# Cryptocaulaceae: a new and deeply diverged branch within the fern tree of life

**DOI:** 10.1007/s10265-026-01698-0

**Published:** 2026-04-17

**Authors:** Tao Fujiwara, Ponpipat Limpanasittichai, Katsuhiro Yoneoka, Shuichiro Tagane, Thyraphon Vongthavone, Takenori Yamamoto, Van-Son Dang, Phetlasy Souladeth, Voradol Chamchumroon, Atsushi Ebihara, Michael Sundue, Li-Yaung Kuo

**Affiliations:** 1 The Mt. Fuji Institute for Nature and Biology, Showa Medical University, 4562 Kamiyoshida, Fujiyoshida, Yamanashi 4030005 Japan; 2 Institute of Molecular and Cellular Biology, National Tsing Hua University, 30013 Hsinchu, Taiwan; 3 Center for Frontier Research, National Institute of Genetics, 1111 Yata, Mishima, Shizuoka, 4118540 Japan; 4 The Kagoshima University Museum, Kagoshima University, 1-21-30, Korimoto, Kagoshima, 890-0065 Japan; 5 Association for Community Development (ACD), Salavan, Salavan Province Laos; 6 Rural Regeneration Research Center, Yamagata University, 1-23 Wakaba- machi, Tsuruoka, 997-8555 Japan; 7 Institute of Life Sciences, Vietnam Academy of Science and Technology, 85 Tran Quoc Toan Street, Xuan Hoa Ward, Ho Chi Minh City, Vietnam; 8 Faculty of Forest Science, National University of Laos, Dongdok Campus, 01170 Xaythany District, Vientiane Capital Laos; 9 Department of National parks, Wildlife and Plant Conservation, The Forest Herbarium (BKF), Chatuchak, Bangkok, 10900 Thailand; 10 Department of Botany, National Museum of Nature and Science, 4-1-1 Amakubo, Tsukuba-shi, Ibaraki 305-0005 Japan; 11 Royal Botanic Garden Edinburgh, 20A Inverleith Row, Edinburgh, EH3 5LR Scotland, UK

**Keywords:** Molecular systematics, Plastome, Pteridophyte, Taxonomy, Tectariaceae, Transcriptome

## Abstract

Advances in phylogenomics have greatly clarified fern evolutionary relationships, yet significant gaps remain. In this study, we performed phylogenetic and phylogenomic analyses of Polypodiineae, one of the most species-rich suborders of Polypodiales, and found that *Tectaria tenerifrons* is a deeply diverged lineage. Our results support it as sister to Nephrolepidaceae in the plastome dataset, whereas the transcriptome dataset recovered it as sister to a clade comprising Nephrolepidaceae, Polypodiaceae, Davalliaceae, Oleandraceae, Tectariaceae s.l. No results support it as a member of the Tectariaceae, where it was previously classified. To reflect this deep divergence, we establish a new family, Cryptocaulaceae fam. nov., and a new monotypic genus, *Cryptocaulon* gen. nov, and propose the new combination *Cryptocaulon tenerifrons* comb. nov. Morphologically, *Cryptocaulon* can be distinguished from *Tectaria* by a combination of characters: long-creeping carnose rhizomes; subclathrate scales with multicellular hairs; anastomosing venation forming multiple rows of areoles without free included veinlets; basal veinlets of pinna lobes on the basiscopic side arising from the pinnule costule; cristate perispores with short, thick ridges; basic chromosome number, *x* = 41. We also designated a lectotype for *C. tenerifrons.*

**Supplementary Information:**

The online version contains supplementary material available at 10.1007/s10265-026-01698-0.

## Introduction

Advances in DNA sequencing technologies and computational power have reshaped our understanding of the tree of life. Across plants, higher classifications are now grounded on inferred phylogenetic relationships (e.g. APG IV 2016; Bechteler et al. 2023; Li et al. 2024; PPG I 2016), with morphological traits serving to diagnose clades in the classification system and assign taxa accordingly. Fern classifications have progressed dramatically during the molecular (Hasebe et al. 1995; Pryer et al. 2001, 2004; Schuettpelz and Pryer 2007; Testo and Sundue 2016) and genomic eras (Fawcett et al. 2021; Kuo et al. 2025), with simultaneous advancement of phylogenetic relationships, and understanding of the evolution of morphological traits across major lineages. This activity has led to the development of phylogenetically-informed fern classifications (e.g. PPG I 2016) that are rich in diagnostic characters. Concurrent with phylogenetic advancement has been a re-evaluation of the diagnostic power of particular characters; many traditionally relied-upon features, such as lamina dissection (Riibe et al. 2021), sorus position and indusia (Schwartsburd et al. 2020) in ferns, are now de-emphasized, while some previously under-utilized features, e.g. stele patterns, perine morphology (Moran et al. 2010; Patel et al. 2019) and rachis-costa architecture have come forward (Labiak et al. 2014; Triana-Moreno et al. 2023). Thanks to this cumulative effort, fern systematists have sampled across morpho-space and most major morphological groups have been incorporated into phylogenetic studies (e.g. Du et al. 2022; Fawcett et al. 2021; Kuo et al. 2024a; Qi et al. 2018; Shen et al. 2018; Tseng et al. 2024; Wang et al. 2022; Wei et al. 2021). As such, many of the most recent insights are among taxa with cryptic placement— lineages whose macromorphology is surprisingly different from their phylogenetic placement. For instance, based upon their linear and occasionally back-to-back sori, *Desmophlebium* Mynssen, A.Vasco, Sylvestre, R.C.Moran & Rouhan, *Hemidictyum* C.Presl, and *Rachidosorus* Ching had been traditionally affiliated to *Diplazium* Sw. and long been treated within the same family Athyriaceae Alston (Kramer and Green 1990). However, phylogenetic evidence resolves them as grade of lineages related to Aspleniaceae Newman (Rothfels et al. 2012). Subsequent morphological comparisons corroborate their phylogenetic placement (Sundue and Rothfels 2014), identifying unique combinations of diagnosable traits and leading to the recognition of several new families within the suborder Aspleniineae (Polypodiales)—Rachidosoraceae X.C.Zhang, Diplaziopsidaceae X.C.Zhang & Christenh., Hemidictyaceae Christenh. & H.Schneid., and Desmophlebiaceae Mynssen, A.Vasco, Sylvestre, R.C.Moran & Rouhan (Christenhusz et al. 2011; Christenhusz and Schneider 2011; Mynssen et al. 2016).

Molecular phylogenetics have also revealed examples of cryptic placement in the suborder Polypodiinae, particularly involving taxa formerly placed in the Tectariaceae Panigrahi. *Tectaria* Cav. has a long history of recognition (often as “*Aspidium*”), based upon round soral morphology, adaxially non-sulcate costae and articulated hairs (Holttum 1984, 1991; Nayar 1970; Pichi Sermolli 1977; Tryon and Stolze 1991; Wang et al. 2014), but the recognition of the family Tectariaceae, and its circumscription have changed considerably. In a series of seven papers on the “fern genera allied to *Tectaria”*, Holttum (1984a 1984b, 1985, 1986a, 1986b, 1988), and Holttum and Edwards (1986) characterized the genera *Ctenitis* (C.Chr.) C.Chr., *Ctenitopsis* Ching, *Pleocnemia* C.Presl, *Pteridrys* C.Chr., *Psomiocarpa* Fée, *Sagenia* Blume, *Tectaria* Cav., and *Triplophyllum* Holttum. Holttum (1991) also include *Tectaridium* Copel., *Chlamydogramme* Holttum, *Heterogonium* Presl., *Aenigmopteris* Holttum, *Cyclopeltis* J.Sm., and *Lastreopsis* Ching in the *Tectaria* group. These studies substantially advanced the systematics of tectarioid ferns; however, subsequent molecular studies later demonstrated that many of these ferns including the genus recognized by Holttum—e.g., *Cyclopeltis*, *Ctenitis*, *Draconopteris* Li Bing Zhang & Liang Zhang, *Dryopsis* Holttum & P.J.Edwards, *Lastreops*,* Malaifilix* Li Bing Zhang & Schuettp., *Pleocnemia*, and *Polydictyum* C.Presl—belong in distantly related families, including Dryopteridaceae Herter, Lomariopsidaceae Alston, or Pteridryaceae Li Bing Zhang, X.M.Zhou, Liang Zhang & T.N.Lu (Christenhusz et al. 2013; Dong et al. 2018a; Hasebe et al. 1995; Liu et al. 2014, 2007; Schuettpelz and Pryer 2007; Zhang et al. 2016; Zhou et al. 2018). *Dracoglossum*, formerly placed in *Tectaria*, for example, was resolved as sister to *Lomariopsis* Fée (Christenhusz et al. 2013); a result that was unexpected given their disparate morphologies. Likewise, *Dryopolystichum* Copel., an essentially unplaced taxon historically allied with *Dryopteris* Adans., *Polystichum* Roth, or *Tectaria* on the basis of its peltate indusia and leaf form, was resolved in Lomariopsidaceae (Chen et al. 2017). These findings indicate that the traditional emphasis on soral shape, articulated hairs, and costa morphology is insufficient to diagnose natural groups within the Tectariaceae, and that additional cryptic lineages may remain hidden within Tectariaceae.

Our current understanding of the fern tree of life is based on phylogenetic investigations encompassing approximately 6,000 species—nearly half of all taxonomically described fern diversity (Nitta et al. 2022; PPG I 2016). By conducting fern floristic surveys globally, we continue to fill the remaining tree “gaps”, enriching the DNA dataset to phylogenetically link the missing tips and branches. In the present study, *Tectaria tenerifrons* (Hook.) Ching, a long-unquestioned relationship to tectarioid ferns and known only from mainland Southeast Asia, represented as a remarkable case. Through in-depth phylogenetic investigation of Tectariaceae, we find that this fern represents a previously unrecognized and deeply diverged lineage in Polypodiineae. To accommodate it while maintaining the monophyly of other taxa, we establish a new genus *Cryptocaulon* and a new family Cryptocaulaceae. Examination of its morphology and chromosome number additionally supports its distinction from *Tectaria*.

## Materials and methods

### Taxon samplings for phylogeny

Our phylogenomic sampling focused on the suborder Polypodiineae encompassing all eleven families in this suborder, that had been recognized in the most recent classification updates (PPG I 2016; https://github.com/pteridogroup/ppg). As for the outgroups, we also sampled two representatives respectively from the sister suborders Pteridineae and Aspleniineae. For the family-level phylogenomics, a total of 38 genera across these Polypodiineae families were included, each with both transcriptomic and plastomic data available. For the species-level phylogeny, we had a much broader sampling across Polypodiineae, including additional 2,239 species and 110 genera using the FTOL 1.7 dataset (Nitta et al. 2022). Detailed information on all these sampled taxa and GenBank accessions of their sequences/reads is provided in Tables S1 and S2.

### Family-level nuclear phylogenomics

To obtain nuclear transcriptomic sequences from newly collected samples, total RNA was extracted from either fresh, RNA-later fixed (RNApreserve; BIONOVAS, Toronto, Canada), or recently silica-dried leaf tissues using the CTAB method in combination with Spectrum Total RNA Kit (Sigma-Aldrich, St. Louis, MO, USA) as described in Pelosi et al. (2024). After extraction, RNA was treated with TURBO DNase (Thermo Fisher Scientific, Waltham, MA, USA), and cDNA libraries were constructed using Zymo-Seq RiboFree Total RNA Library Kit (Zymo Research, Irvine, CA, USA). Transcriptome sequencing was performed on the Illumina NovaSeq platform (Illumina, San Diego, CA, USA), generating 5–12 Gbp PE150 reads per sample. After trimming by fastp (Chen et al. 2018b), PE reads were used for transcriptomic assembling by Trinity (Grabherr et al. 2011), followed by clustering of protein-coding sequences (CDS) with CD-HIT v4.6 (Fu et al. 2012) using a minimum length of 100 bp and a sequence identity threshold of 0.98.

Together with earlier published transcriptomes (Table S1; One Thousand Plant Transcriptomes Initiative 2019; Qi et al. 2018; Shen et al. 2018), CDSs across all assemblies were then assigned to orthologous groups (OGs) using OrthoFinder (Emms and Kelly 2019). To minimize interference due to missing data and paralog inclusion in downstream nuclear phylogenomics, OGs were filtered based on two criteria: (1) more than 80% of Polypodiineae samples contained single or only two copies, and (2) the sample of our new family also contained single or only two copies. In total, 1,711 OGs were retained for the downstream analyses. For each retained OG, samples with more than two copies were thus removed. When a sample contained two copies, only the longer one was retained. Filtered CDS sequences were aligned in a codon-aware manner by MAFFT (Katoh and Standley 2013) with a modified script (https://github.com/lyy005/codon_alignment). Putative 5′UTR regions in these alignments were removed using a customized python script, and the resultant alignments were further trimmed using trimAl (Capella-Gutiérrez et al. 2009) with default settings.

We compiled a concatenated matrix of all selected OGs (i.e. “all”) and two submatrices comprising only third codon positions (“codon3”) and only first and second codon positions (“codon1 + 2”), respectively. Each matrix was analyzed under three substitution models/schemes using IQ-TREE v2.1.3 (Minh et al. 2020; Nguyen et al. 2015): (1) GTR + F+R10 model without any partition, (2) GTR + F+R10 model with partitions by codon positions by genes, (3) ModelFinder-determined model/scheme (i.e. “-m TESTNEWMERGE -rcluster 10”) with partitions by codon positions by gene. For each analysis, a maximum likelihood (ML) phylogenomic tree was reconstructed with 1000 ultrafast bootstrap (UFBS) replicates (Hoang et al. 2018).

We also incorporated the plastome dataset (see below) as a single locus into the “all” nuclear dataset. An ML tree was reconstructed from this combined, concatenated matrix using IQ-TREE with the inferred substitution models and partitioning schemes. To infer individual gene trees for these nuclear OGs and plastome, ML phylogenetic trees were reconstructed, with the best-fit substitution model and partitioning scheme selected by ModelFinder (i.e. “-m TESTNEWMERGE -rcluster 100”), which was first partitioned by codon positions. Resulting gene trees were applied to generate a species tree using ASTRAL-III (Zhang et al. 2018) with local posterior probabilities (LPP) and to infer gene concordance factors (gCF; Minh et al. 2020). The concatenated matrix was also used to calculate site concordance factors (sCF; Minh et al. 2020) to assess support for phylogenetic inferences.

### Family-level plastome phylogenomics

To assemble plastomes of the new family samples, we adopted a genome skimming approach. Total genomic DNA was extracted using the modified CTAB protocol. The fragment size of the extracted DNA was initially assessed using a Qsep100 with an N1 cartridge (BiOptic Inc., New Taipei City, Taiwan). If the average fragment exceeded 600 bp, the DNA was sheared using a Bioruptor Plus (Diagenode, Denville, NJ, USA) until the desired fragment size (~ 600 bp) was reached. DNA libraries were reconstructed using NEBNext Ultra II DNA Library Prep Kit with NEBNext Dual Index Primers Set (New England Biolabs, Ipswich, MA, USA), and were sequenced on the NovaSeq PE150 platform (Illumina, San Diego, CA, USA) generating 5–10 Gbp of PE150 reads per sample. Raw reads were trimmed using fastp (Chen et al. 2018b), and plastome assembly was performed using NOVOplasty (Dierckxsens et al. 2017) with a setting of “Kmer = 39” and a conspecific *rbcL* sequence as the seed. The assembled plastomes were annotated using Geneious (Kearse et al. 2012) with other Polypodiineae plastomes as references. Gene annotations were manually verified and adjusted as necessary.

Because of inconsistent annotation and occasional pseudogenization in other Polypodiineae plasomes, *rps16*, *ycf94*, and *rps12* were excluded from the downstream analyses. A total of 82 plastid CDS were finally included in our plastome datasets. These CDS sequences were aligned using MACSE v. 2.03 (Ranwez et al. 2011). Since all plastid genes in ferns are presumed to be co-transferred and maternally inherited (reviewed in Kuo et al. 2018b), we treated the entire plastome as a single locus, with all CDSs concatenated into a single ‘gene’ alignment. As with the nuclear dataset, we also generate two additional submatrices representing different codon positions. The downstream ML analyses of these plastome matrices followed the same procedures as described above for nuclear phylogenomics, including model testing, partitioning schemes, and UFBS.

To confirm whether these plastid CDS genes yield different gene trees, we further analyzed their individual gene trees and inferred a species tree, following the same methods described above for the nuclear phylogenomic analyses.

### Species-level plastid phylogeny

In addition to the two plastome-sequenced samples (Table S2), four additional collections representing the geographic range of the new family were included in our species-level phylogeny. The total DNA was extracted from silica dried leaves using the CTAB method according to Doyle and Doyle (1987). Five plastid DNA regions, *rbcL*, *atpA*, *atpB*, *rps4*-*trnS* (*rps4* gene + *rps4*-*trnS* intergenic spacer), and *trnL*-*F* (*trnL* gene + *trnL*-*trnF* intergenic spacer) were amplified with the primer sets shown in Table S3. The PCR amplifications were performed using Prime STAR Max DNA Polymerase (Takara, Kyoto, Japan) under the following conditions: 95 °C for 7 min, followed by 35 cycles of 98 °C for 10 s, 55 °C for 15 s, and 72 °C for 10 s, and 72 °C for 7 min. The PCR reactions were carried out in a T100 Thermal Cycler (Bio-Rad, Hercules, CA, USA). The PCR products of the plastid DNA regions were directly purified using the Exo SAP-IT Express PCR Product Cleanup Reagent (USB Products Affymetrix, Inc., Cleveland, OH, USA) and used as templates for Sanger sequencing. The sequencing reactions were prepared using the QuantumDye Terminator Cycle Sequencing Kit (Tomy Digital Biology, Tokyo, Japan) and analyzed on an ABI 3500xl Genetic Analyzer (Applied Biosystems, Foster City, CA, USA). The newly generated plastid sequences were merged into the FTOL v1.7 dataset (Nitta et al. 2022) using the “—addfull” function in MAFFT v7.450 (Katoh and Standley 2013). The single-locus alignments were then concatenated and used to reconstruct a Polypodiineae species-level phylogeny. The concatenated matrix was first partitioned by genic region by codon position. ModelFinder was used to identify the best-fitting substitution model and partitioning scheme (“-m TESTNEWMERGE -rcluster 100”). Based on this, a ML phylogeny was reconstructed with 1000 UFBS replicates using IQ-TREE.

### Morphological observation

Morphological traits were mainly observed with a stereomicroscope and microscope. Spore morphology was examined using a tabletop scanning electron microscope (JCM-6000 Plus, JEOL Ltd., Tokyo, Japan). Spores were mounted directly onto carbon adhesive tape attached to an aluminum stub without additional conductive coating. Observations were conducted under low-vacuum conditions using a backscattered electron detector in composition mode (BED-C) at an accelerating voltage of 15 kV with the probe current set to the standard level (PC-std.). For observation of the gametophyte, spores of *T. teneifrons* were collected from a plant (Voucher number, *Kuo-5171*). Spores were directly sown on soil in a plastic case, and cultivated in a growth chamber. We picked mature gametophytes and then observed them under a microscope.

### Chromosome counting and genome size estimation

For chromosome counting, mitotic chromosome numbers were determined using the hematoxylin squash method (Limpanasittichai and Jaruwattanaphan 2022). Young fronds from a *T. tenerifrons* collection (*Kuo5171*, vouched in the herbarium TAIF) were pretreated with 2 mM 8-hydroxyquinoline for 6–8 h at 16–18 °C, and then were fixed in freshly prepared Carnoy’s solution (ethanol: acetic acid = 3:1) for 24 h, hydrolyzed in 1 M HCl for 5 min at 60 °C, stained with 1% hematoxylin (Guerra 1999) for 10 min, and squashed.

For flow cytometric (FCM) experiments, we followed the protocol developed by Kuo and Huang (2017), used LB01 (Doležel et al. 2007) buffer containing 4% PVP-40, 0.5%(v/v) 2‐mercaptoethanol, and RNase A (0.1 mg/mL). First, the fresh leaf tissues of the same *T. tenerifrons* collection (*Kuo5171*) were placed in a Petri dish on ice, and chopped with buffer to extract the nuclei using a razor. Similarly, we extracted leaf 2 C nuclei of *Vicia faba* L. cv. Inovec (26.9 pg; Doležel et al. 1992), which was used as our internal standard. The extractions were then filtered through 30‐µm nylon meshes (Sysmex Partec, Goerlitz, Germany). Propidium iodide (PI) solution was then added to each sample to a final concentration of 0.04 mg/mL, and then placed in the dark at 4° C for 1 h for PI-staining. Finally, we performed the FCM analyses on a CytoFLEX system (Beckman Coulter, Brea, CA, USA), three replicates per sample, and set criteria to collect > 1,300 particles per peak, each with a coefficient of variation (CV) < 5%.

## Results

### Plastome and transcriptome assemblies

The plastomes of Cryptocaulaceae ranged in size from 151,047 to 152,064 kbp, and each consisted of a single circular chromosome. Genic content and structure of these plastomes were consistent with those of most Polypodiineae members, including the closely related families Nephrolepidaceae and Lomariopsidaceae. We did not find any arrangement and IR (inverted repeat region)-SC (single copy regions) boundary change among these plastomes. A total of 85 CDS (including *rps16* and *ycf94*), 29 tRNA, and four rRNA genes were identified in these plastomes. As in all other Polypodiineae members, the *psaM* gene was absent in Cryptocaulaceae plastomes. The annotation details are provided in gff files deposited at https://github.com/lykuofern/Eu1_new.

The transcriptome assemblies of the Cryptocaulaceae sample contained 67,036 CDS contigs, which were clustered into 3,183 OGs. For the two additional newly sequenced samples, *Lomariopsis* and *Lastreopsis*, transcriptome assemblies contained 58,511 and 55,005 CDS contigs, respectively. These assemblies are also provided at https://github.com/lykuofern/Eu1_new.

### Phylogenetic and phylogenomic patterns

**Fig. 1:**
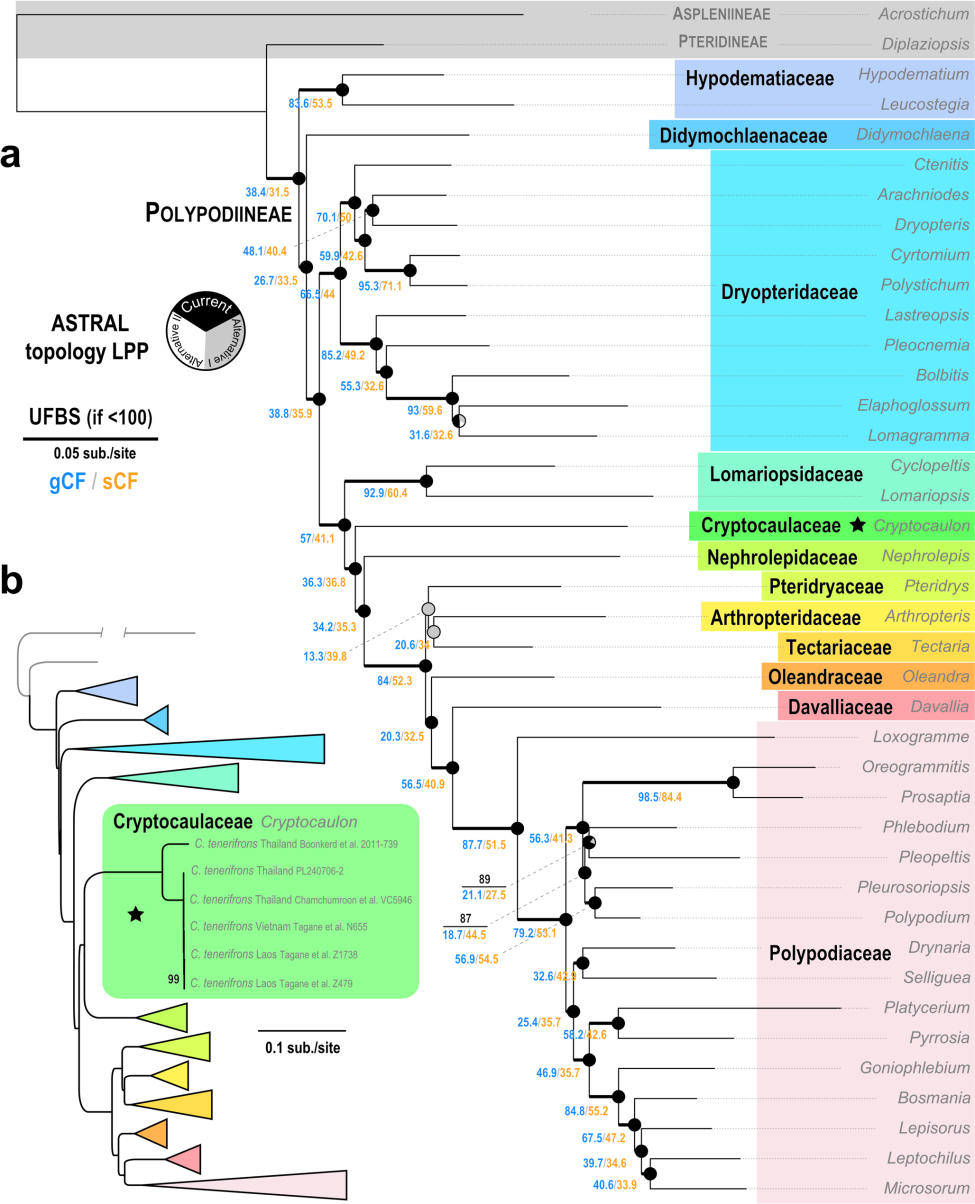
Polypodiineae relationships based on the family-level phylogemonic dataset (**a**) and the five-region species-level plastid dataset (**b**). Thickened branches represent their ultrafast bootstrap (UFBS) value of 100. For branches with UFBS < 100, exact values are shown in black on branches. Tips belonging to different Polypodiineae families are indicated by different colors. In the family-level phylogemonic tree incorporating 1,171 nuclear and 82 plastid CDSs (**a**), additional branch supports are provided, including gene and site concordance factors (gCF and sCF) and ASTRAL local posterior probabilities (LPP). The full species-level plastid tree (**b**) is provided in Fig. S2

**Fig. 2:**
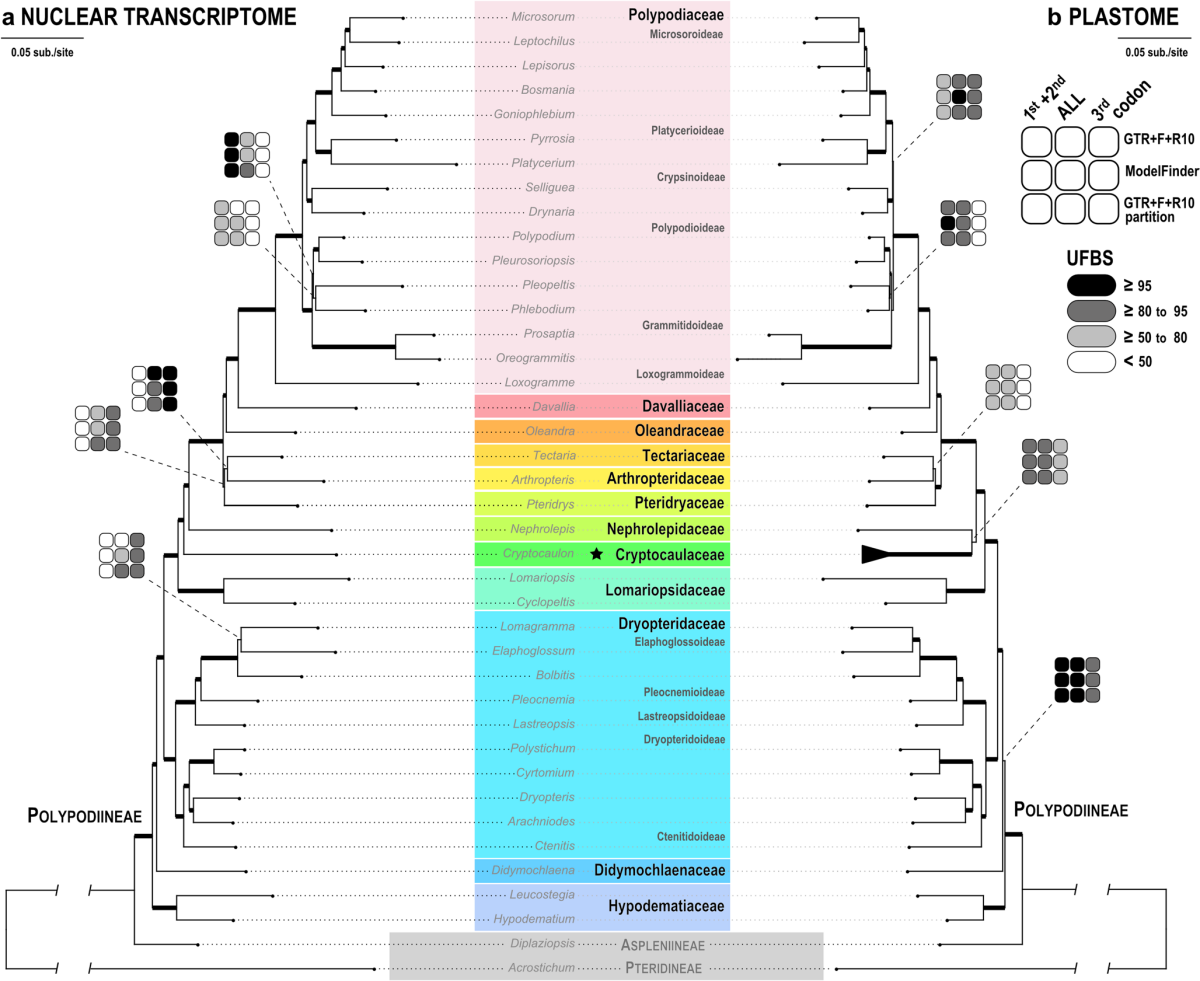
Phylogenomic trees of polypodiineae based on 1,171 nuclear CDSs (**a**) and 82 plastid CDSs (**b**). Tips belonging to different Polypodiineae families are indicated by different colors. Thickened branches indicate their ultrafast bootstrap (UFBS) values of 100 across all analyses. For branches with any UFBS < 100, their individual UFBS from different analyses are indicated in 3 × 3 grids next to branches

The new family Cryptocaulaceae is nested within the suborder Polypodiineae (Figs. 1 and 2). In the phylogenomic tree combining 1,711 nuclear and 83 plastid CDSs, Cryptocaulaceae is strongly supported (UFBS = 100 and ASTRAL LPP = 1.00) as the sister of the lineage comprising Nephrolepidaceae and remaining families, despite the corresponding sCF and gCF values not being very high (36.3 and 36.8, respectively) (Fig. 1a). In contrast, our plastid phylogenies place Cryptocaulaceae sister to Nephrolepidaceae, with high (UFBS = 100) and moderate (UFBS = 50–95 and ASTRAL LPP = 0.74) branch supports in the five-region species-level tree (Fig. 1b; Supplementary file 1) and plastome family-level tree (Figs. 2b, S1), respectively. This incongruence was clear between our nuclear (Fig. 2a) and plastid phylogenomic results (Fig. 2b). Despite this, most other family-level relationships are congruent between nuclear and plastid phylogenomics (Figs. 1 and 2), and are consistent with previous studies (Du et al. 2022; Pelosi et al. 2022; Qi et al. 2018). Notably, a few nodes showed considerable variation in branch support across analyses using different codon partitions (Fig. 2). In particular, the monophyly of Tectariaceae s.l. (Tectariaceae + Arthropteridaceae + Pteridryaceae) and of the subfamily Polypodioideae (Polypodiaceae), as well as the internal relationships within these groups received inconsistent support, also echoing patterns seen in earlier fern phylogenies (Qi et al. 2018). Additionally, we also found two genetically diverged lineages in this monotypic new family (Figs. 1b and 2b), indicating the presence of a cryptic taxon.

### Morphological comparison

**Fig. 3:**
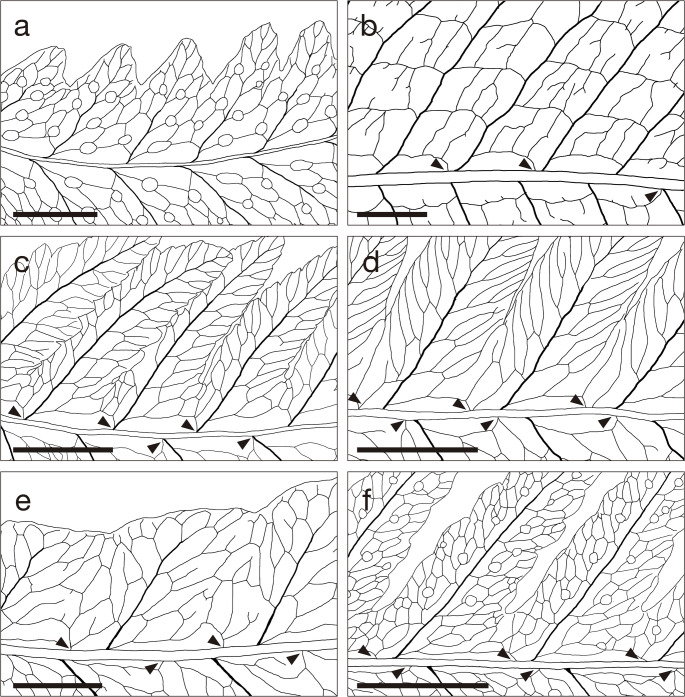
Morphological comparison of venation patterns between *Cryptocaulon* and *Tectaria*. Arrows in each panel indicates the basal veinlets of pinna-lobes on the basiscopic side arising from pinna-rachis, which is a synapomorphy in *Tectaria*. **a**. *C. tenerifrons* (Chamchumroon et al. VC5946); **b**. *T. decurrens* (PL251211-1); **c**. *T. devexa* (Kuo4671-2); **d**. *T. fuscipes* (Kuo5139); **e**. *T. griffithii* (PL251211-2); **f**. *T. mexicana* (Sundue and Torres 3504). Scale 1 cm

**Fig. 4:**
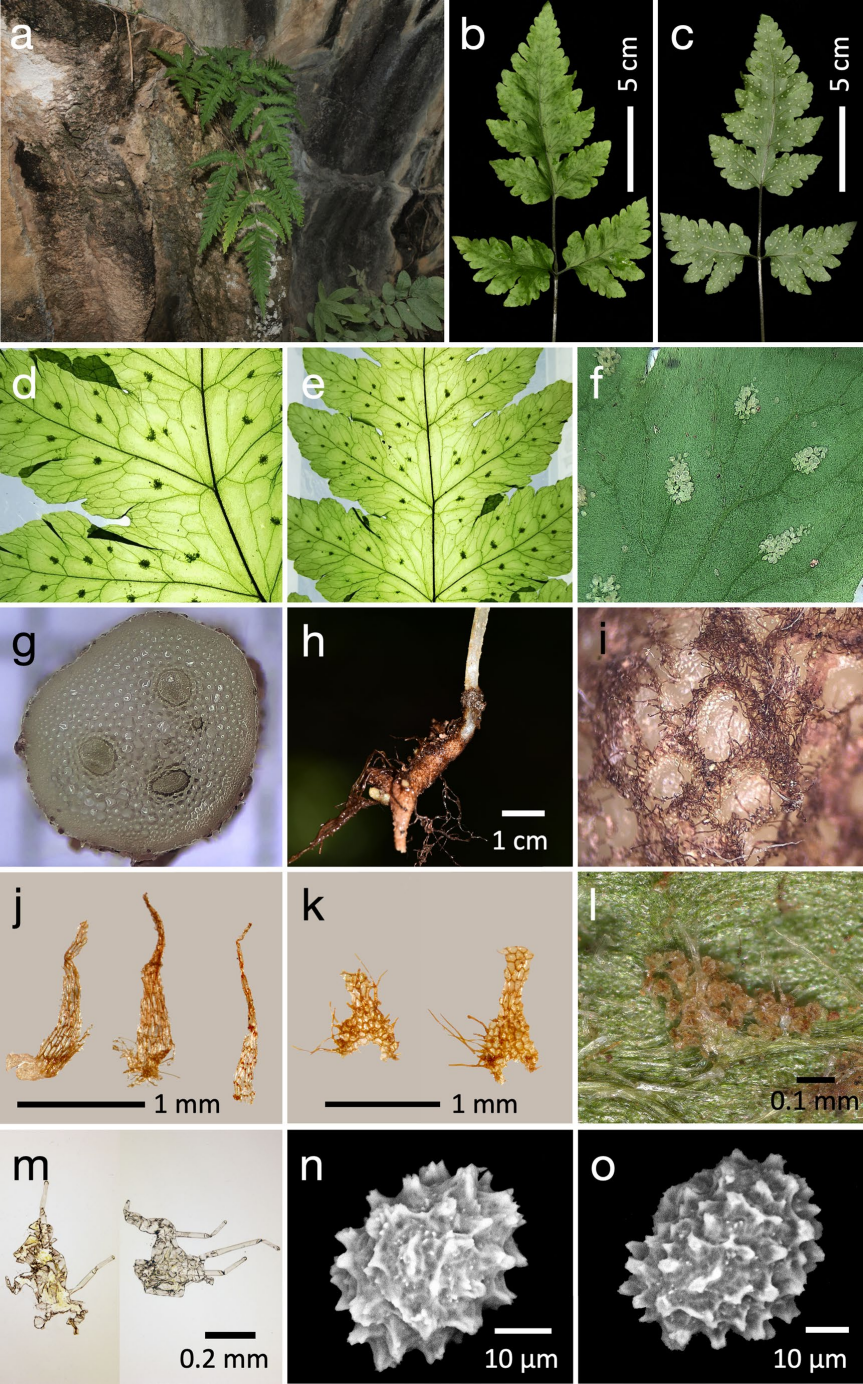
*Cryptocaulon tenerifrons.* **a**. Habit; **b**. Adaxial side of lamina; **c**. Abaxial side of lamina; **d**. Venation pattern of the proximal pinna; **e**. Venation pattern of the terminal pinna; **f**. Young sorus. **g**. Transverse section of stipe base; **h**. Rhizome; **i**. Rhizome indument; **j**. Scales of the stipe; **k**. Scales of the rhizome; **l**. Indusium covering sorus; **m**. Indusia; **n** and **o**. Spores. Photos a from Chamchumroon et al. VC5946; **b**–**i** from Souladeth et al. PPM123 (FOF); **j**, **k**, **l**, **m**, **n,** and o from Chamchumroon et al. VC5946

**Fig. 5:**
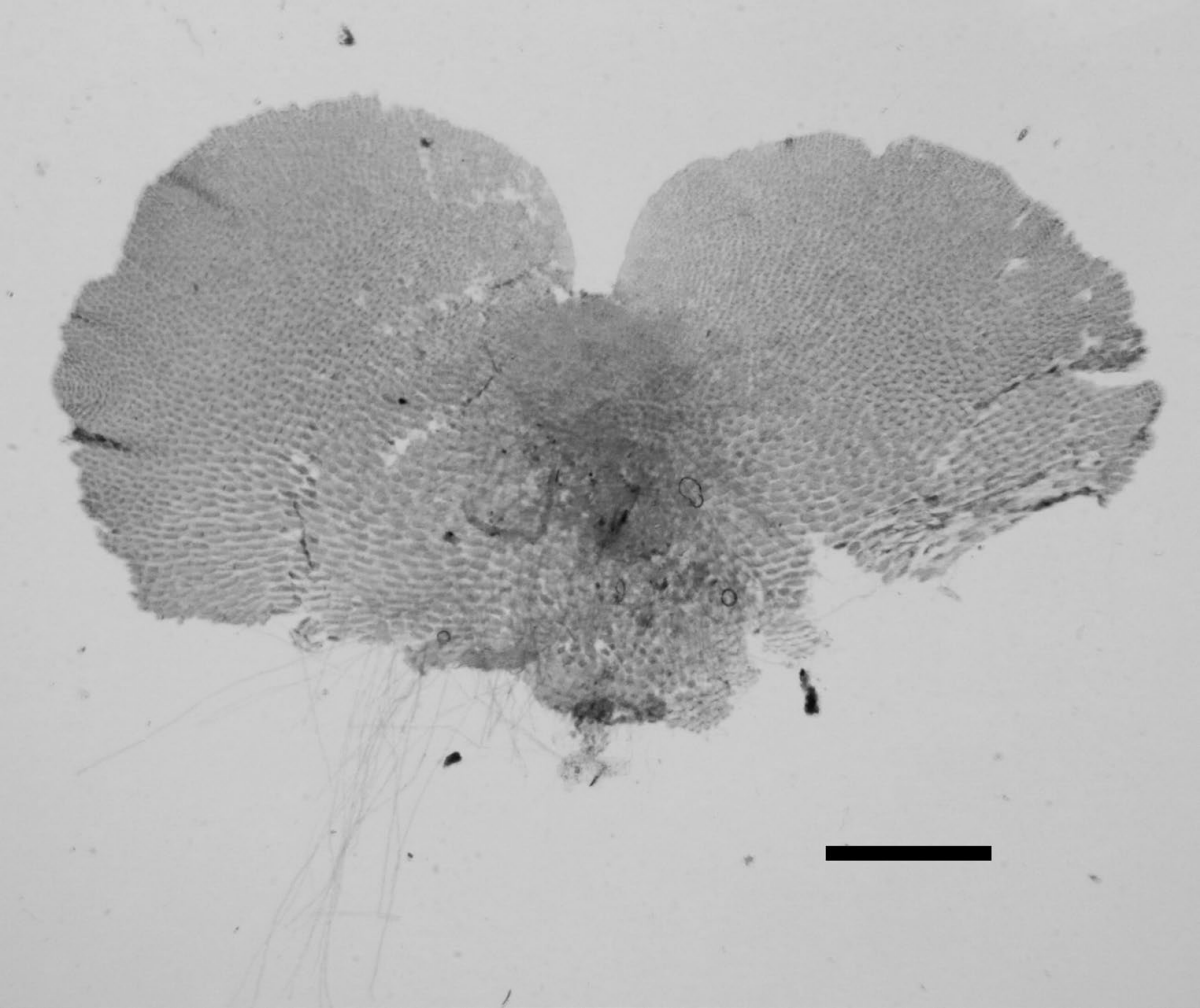
Mature gametophyte of *Cryptocaulon tenerifrons*. Scale 1 mm

We found that the venation patterns are the most prominent diagnostic character to differentiate *Cryptocaulon* from *Tectaria* (Figs. 3 and 4d and e). We also identified additional morphological traits that distinguish the genus from Tectariaceae and related families, including sorus (Fig. 4f), rhizome (Fig. 4h), scale cell pattern and scale shape (Fig. 4i, j), indusia (Fig. 4l, m), and spore ornamentation (Fig. 4n, o). The mature gametophyte was cordate (Fig. 5). The main morphological characteristics among the families in Polypodiineae including Cryptocaulaceae are summarized in Table 1.

**Table 1:** Morphological comparison among Cryptocaulaceae and Polypodiineae families

Family	Rhizome	Rhizome carnose	Scale	Hairs on abaxial surface of scales	Lamina	Leaves articulate	Hairs on fronds	Venation	Indusia	Perispore	Chromosome base number
Cryptocaulaceae fam. nov.	Creeping	Yes	Subclathrate	Present	2–3 pinnatifid	No	Present	Anastomosing	Minute, deltoid tolanceolate	Cristate, forming cristate ridges that vary from short to elongate	x = 41
Didymochlaenaceae Ching ex Li Bing Zhang & Liang Zhang	Erect	No	Concolorous	Absent	2 pinnate	No	Absent	Free	Elliptic	Tuberculate	*x* = 41
Hypodematiaceae Ching	Creeping	No	Concolorous	Absent	2–4 pinnate	Yes or no	Present or absent	Free	Circular or round-reniform	Coarsely verrucate or tuberculate	*x* = 41
Dryopteridaceae Herter	Erect or creeping	Mostly no	Concolorous or clathrate	Absent	Simple to 4 pinnate	Mostly no	Present or absent	Free to anastomosing	Round-reniform or peltate, or exindusiate	Highly variable	*x* = 41
Lomariopsidaceae Alston	Erect or creeping	Yes or no	Concolorous or clathrate	Absent	Simple, pinnate, or pinnate-pinnatifid	No	Present or absent	Free, rarely anastomosing	Round-reniform to reniform, or exindusiate	Highly variable	Mostly *x* = 41
Nephrolepidaceae Pic.Serm.	Erect	No	Concolorous	Absent	1 pinnate	No	Mostly absent	Free	Round-reniform to reniform	Irregularly tuberculate, occasional globules	*x* = 41
Tectariaceae Panigrahi	Erect or creeping	Mostly no	Concolorous	Absent	Simple, pinnatifid, or 1–2 pinnate	No	Mostly present	Free to anastomosing	Round-reniform to reniform, or exindusiate	Broad folds, cristate, echinate or echinulate	*x* = 40, 41
Arthropteridaceae Hong M.Liu, Hovenkamp & H.Schneid.	Creeping	No	Concolorous	Absent	1 pinnate	Yes	Present	Free or rarely anastomosing	Reniform	Erose, fimbriate. cristate, or with broad folds	*x* = 41
Pteridryaceae Li Bing Zhang, X.M.Zhou, Liang Zhang & N.T.Lu	Erect or creeping	No	Concolorous	Absent	1 pinnatifid, or 1–2 pinnate	No	Mostly absent	Free to anastomosing	Rounded or reniform	Broad folds, or cristate	*x* = 39, 41
Oleandraceae Ching ex Pic.Serm.	Creeping	No	Concolorous	Absent	Simple	Yes	Present or absent	Free	Round-reniform	Broad folds, cristate, or echinate	*x* = 41
Davalliaceae M.R.Schomb.	Creeping	Yes or no	Concolorous or clathrate	Mostly absent	1–4 pinnate, rarely simple	Yes	Mostly absent	Free	Cup-shaped, reniform, or lunate	Verrucate or tuberculate	*x* = 40
Polypodiaceae J.Presl & C.Presl	Creeping	Yes or no	Concolorous or clathrate	Mostly absent	mostly simple or pinnatifid	Yes	Present or absent	Free to anastomosing	Exindusiate	Usually smooth and provided with globules, sometimes tuberculate, verrucate or granulate	Mostly x = 35, 36, 37

### Cytological observation and genome size measurement

**Fig. 6:**
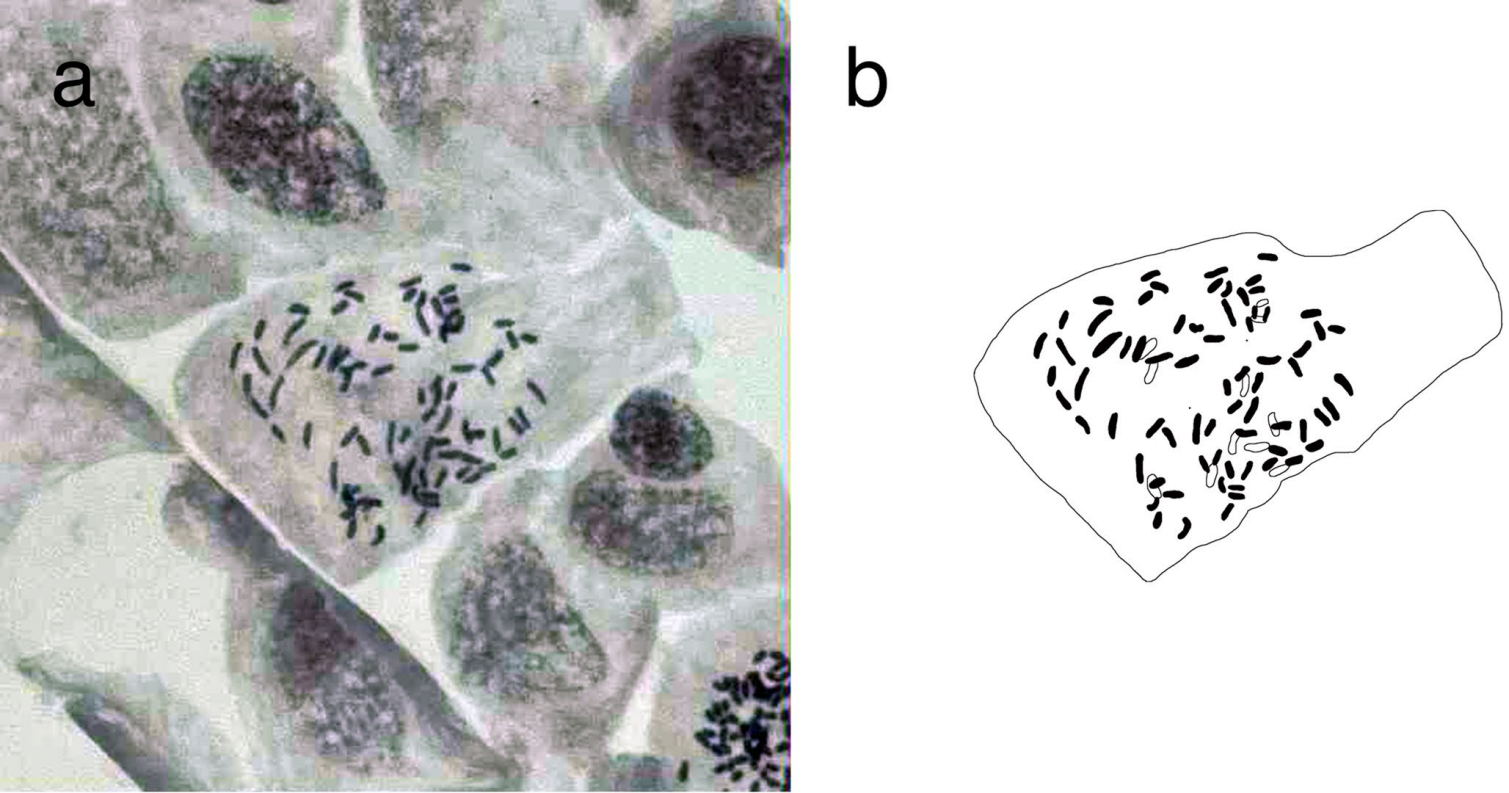
Mitotic metaphase chromosome of *Cryptocaulon tenerifrons*. **a**. Microphotographs and **b**. Explanatory illustrations

The chromosome count is shown in Fig. 6. A chromosome number of 2*n* = 82 was counted for *Cryptocaulon tenerifrons*, indicating diploid cytotype. The relative DNA content values of the same sample was 6.62 pg/C on average.

## Discussion

### Phylogenomic inference and incongruence in a deeply divergent fern lineage

Systematists are tasked with deepening and broadening our evolutionary understanding of the tree of life. With the advancement of phylogenomic tools, many previously unresolved evolutionary nodes have now been substantially clarified. Ferns are the second most diverse branch among extant land plants (PPG I 2016), and our current understanding of the fern tree of life is based on phylogenetic investigations encompassing approximately 6,000 species—nearly half of all taxonomically described fern diversity (Nitta et al. 2022; PPG I 2016). Among these, approximately 1% have available plastome sequences (Nitta et al. 2022), which have been instrumental in reconstructing phylogenomic trees and resolving long-standing ambiguities in fern evolutionary history (Du et al. 2022; Kuo et al. 2024a). Nonetheless, gaps remain, including taxa with unsettled phylogenetic placement (Kuo et al. 2024b). In the present study, we highlight such an example. Through phylogenetic investigation of ferns in one of the biodiversity “dark spots” (Ondo et al. 2024), mainland Southeast Asia, we discovered a previously unrecognized branch in the fern tree of life (Figs. 1 and 2), estimated to have diverged from other Polypodiinae over 120 million years ago (Nitta et al. 2022). Although currently represented by a single species, our results suggest the possibility of an additional morphologically cryptic but genetically distinct taxon within this lineage (Figs. 1b and 2b), warranting additional systematic studies.

Phylogenomics offers valuable insights into the tree of life, particularly for shallow, rapidly bifurcating branches. This is accomplished by the use of greatly expanded character matrices that integrate numerous sites and loci in phylogenomic analyses. Nonetheless, this approach also introduces new phylogenetic challenges (e.g. Smith et al. 2022; Wanke and Wicke 2023). First, individual loci might differ in their genealogies due to their different fates on segregated chromosomes and/or histories of duplication-and-loss (Hime et al. 2021; Huang et al. 2025; Smith et al. 2022). As a result, nuclear genome datasets often harbor incongruent phylogenetic signals, underscoring the importance of examining individual gene trees to assess the robustness of inferred relationships (Kandziora et al. 2022; Morales-Briones et al. 2021; Smith et al. 2015). Second, selecting appropriate substitution models that account for rate heterogeneity across genes and sites is also a challenge (Kapli et al. 2020). Oversimplified, unrealistic substitution models can produce inaccurate branch lengths, which in turn may result in artifacts during tree reconstruction, known as branch attraction issues (Kapli et al. 2020). In ferns, a lineage with evolutionary origins dating back over 400 million years, phylogenomic incongruences have been reported at several deep nodes (Kuo et al. 2018b; Wang et al. 2022). In our phylogenomic results of Polypodiineae, we also found incongruences between nuclear and plastid genes and between different codon positions (Fig. 2). For example, the newly identified clade Cryptocaulaceae was placed differently in our nuclear transcriptome tree and plastome tree (Fig. 2). Gene trees from 1711 nuclear loci recovered Cryptocaulaceae as sister to Nephrolepidaceae + other decedent Polypodiineae families with high supports (ASTRAL LPP = 1.00 and UFBS = 100; Figs. 1a and 2a). In contrast, the plastome phylogeny, that is based on 82 CDSs, resolved Cryptocaulaceae as sister to Nephrolepidaceae, with lower branch support (UFBS = 50–95 and ASTRAL LPP = 0.74; Figs. 2b, S1). Another example involves Tectariaceae s.l. (Tectariaceae + Arthropteridaceae + Pteridryaceae), whose monophyly was weakly supported by nuclear genes (gCF = 13.3, ASTRAL LPP = 0; Fig. 1a), but strongly supported by the plastome phylogenetic tree (UFBS = 100 and ASTRAL LPP = 1.0; Figs. 2b, Fig. S1), coherent to earlier findings (Du et al. 2022; Pelosi et al. 2022; Qi et al. 2018). Incongruencies within our phylogenomic results were most pronounced between different codon positions (Fig. 2), and were somewhat mitigated by the use of partitioned models (i.e. by ModelFinder or GTR + F+R10 with partition; Fig. 2). Such codon-specific discordance has been previously documented (e.g. Kuo et al. 2018a, 2024a) and is largely attributable to variation in evolutionary rates across codon positions (i.e., usually low in the 1st + 2nd codons and high in the 3rd codon) (Kuo et al. 2018a).

### The recognition of Cryptocaulaceae and *Cryptocaulon* as a new family and genus

Recognition of Cryptocaulaceae and *Cryptocaulon* as a distinct family and genus for *C. tenerifrons* accords with the current trend toward rank assignments that faithfully reflect the phylogenetic relationships of ferns and lycophytes (PPG I 2016). *Cryptocaulon* is unequivocally segregated from Tectariaceae and its allied families: plastome data place *Cryptocaulon* in a weakly supported clade with Nephrolepidaceae (Figs. 1b and 2b), but nuclear transcriptome data do not corroborate this relationship and recovered it as sister to a clade comprising Nephrolepidaceae, Polypodiaceae, Davalliaceae, Oleandraceae, Tectariaceae and other closely related families (Fig. 2a), indicating that the species constitutes an independent lineage from all recognized families. To preserve the monophyly of *Tectaria* and Tectariaceae and acknowledge the uniqueness of *Cryptocaulon*, the species should be accommodated in a new genus, *Cryptocaulon* and treated as the sole member of the new family, Cryptocaulaceae. As detailed below, morphological comparison also reveals several unique character states that further distinguish this lineage from all putatively allied families demonstrating that the genus and family can be characterized both phylogenetically and morphologically.

### Morphological and cytological diagnostics of Cryptocaulaceae

*Cryptocaulon* is most similar to *Tectaria*, and has long been assigned to the genus based on several diagnostic characters (Ching 1936; Holttum 1988, 1991). Notably, traits that are widely regarded as typical of *Tectaria*—a pinnate lamina with the basal pinnae basiscopically enlarged, a dense covering of articulated hairs on both stipe and lamina surfaces, and an anastomosing venation pattern—are all conspicuously present in *Cryptocaulon* (Holttum 1991). In light of our results, we interpret these features as homoplastic. A similar interpretation was made for the genera *Ctenitis*, *Lastreopsis*, *Pleocnemia*, and *Dracoglossum*; these were once accommodated within Tectariaceae or the *Tectaria* group on the basis of the similar morphological characters but are now placed in the Dryopteridaceae or Lomariopsidaceae and those characters are interpreted as homoplastic (Liu et al. 2014; Schuettpelz and Pryer 2007).

*Cryptocaulon* lacks several traits regarded as core synapomorphies of *Tectaria.* First, veinlet patterns, specifically, that the basal veinlets of pinna-lobes on the basiscopic side arise from pinna-rachis, is proposed to be a reliable diagnostic character segregating *Tectaria* from the other related genera (Ding et al. 2014). The pattern of veinlets in *Cryptocaulon* is not *Tectaria*-like; the basal veinlets of pinna-lobes on the basiscopic side arise from pinnule costule (Figs. 3 and 4d and e). Second, the combination of the anastomosing veins forming multiple rows of areoles without free included veinlets and sori dorsal on anastomosing veins is also very rare among *Tectaria* (Fig. 3f). There are only two *Tectaria* species with the combination of the characters in Asia, namely *T. chinensis* (Ching & Chu H.Wang) Christenh. and *T. chattagrammica* (C.B.Clarke) Ching (Dong et al. 2018a, b). Third, the chromosome base number is also a distinctive characteristics of *Tectaria* distinguishing from other genera in Polypodiineae, and the number is mostly *x* = 40 (Kramer and Green 1990; Moran et al. 2014). However, the chromosome number of *C. tenerifrons* is counted as 2*n* = 82 (Fig. 6), and thus the chromosome base number of *Crypotocaulon* is *x* = 41. This also supports the segregation of *Crypotocaulon* from *Tectaria*.

Scale morphology is an important character in Cryptocaulaceae. It bears subclathrate scales on the stipes and rhizomes (Fig. 4h–k) with isodiametric cells that bear multicellular hairs both along the margins and across the abaxial surface (Fig. 3k). These characters help distinguish Cryptocaulaceae from *Tectaria*. As noted by Holttum (1983), most species of *Tectaria* possess non-clathrate or concolorous scales, which separates *Tectaria* from *Ctenitis*. Holttum (1991) also noted that although a few *Tectaria* have thin, and translucent scales, these are not truly clathrate and do not exhibit isodiametric cells. Subclathrate to clathrate scales also occur in some Polypodiineae families—Dryopteridaceae, Lomariopsidaceae, Davalliaceae and Polypodiaceae—although non-clathrate or concolorous scales predominate across the suborder (Christenhusz 2007; Kramer and Green 1990) (Table 1). However, scales that bear multicellular hairs over the surface, as in *Crypotocaulon*, appear to be rare in Polypodiineae and have been reported only sporadically (e.g. in some species of *Lepisorus* J.Sm., *Leptochilus* Kaulf., *Microsorum* Link, Zhang et al. 2013; and *Terpsichore* A.R.Sm., Sundue 2010). Taken together, this combination of the subclathrate/clathrate scale with isodiametric cells and multicellular hairs provides a useful diagnostic suite for separating Cryptocaulaceae from the other families of Polypodiineae.

The most distinctive feature of *Crypotocaulon* is its rhizome and growth habit. The rhizome is somewhat carnose, pale in color, and grows deep in limestone fissures (Fig. 4a, h). The rhizomes are missing from most herbarium specimens, likely because most collectors are unable to try it out of the recess in which it is growing. We suggest that the fleshy carnose texture, and habit of growing deep in fissures may be adaptations to the seasonal drought that characterizes its habitat. Carnose rhizomes are relatively uncommon in the Polypodiineae, but are common in epiphytic members of Polypodiaceae and Davalliaceae (Sundue et al. 2015), and in a few Dryopteridaceae such as *Dryopteris watsii* (McKeown et al. 2012). Similar rhizomes are also known from a few *Tectaria*, calciphiles such as *T. manilensis* (C.Presl) Holttum and *T. hymenophylla* (Bedd.) Holttum (Holttum 1988). Although these species share a thinly herbaceous, seasonally deciduous lamina with *C. tenerifrons*, they are readily distinguished by their rhizome‑scale, completely free venation, sori attached at terminal veins, and reniform indusia.

Indusium morphology is particularly useful for discriminating *Crypotocaulon* from the other families of Polypodiineae. The indusium is fugacious, deltoid to lanceolate composed of scarious-greenish-white lobes, with a few articulated hairs (Fig. 4l, m). This indusium is unique within Polypodiineae, others generally being round to reniform (Table 1). Previous authors described *Crypotocaulon* as exindusiate (Ching 1936; Holttum 1986, 1991), but our careful observation located indusia on some sori (Fig. 4l). Thus, the minute scarious and fugacious indusium of *Crypotocaulon* is one of the most useful traits for characterizing the family, Cryptocaulaceae. We note, however, that it is not the only Polypodiinae with minute scarious indusia; similar ones can be found in a few *Tectaria* (e.g. *Tectaria psomiocarpa* S.Y.Dong), which we interpret as another striking homoplastic trait shared between these two lineages.

The perispore of *Crypotocaulon* is decorated with cristate ridges that vary from short to elongate (Fig. 4n, o). This morphology is different from those of Tectariaceae and, Arthropteridaceae and Pteridryaceae which most commonly consists of broad folds, cristate wings, or echinate (Chen et al. 2018a, ; Holttum 1986a; Lugardon 2012; Moran et al. 2014; Tryon and Tryon 2012; Zhang et al. 2016) (Table 1). Although the species is also phylogenetically close to Lomariopsidaceae and Nephrolepidaceae (Figs. 1 and 2), it differs from those as well. Lomariopsidaceae exhibits diverse perine morphology but the structures are mostly broad folds, narrow crests, prominent wing-like folds, tuberculae, or echinae (Chen et al. 2017; Christenhusz 2007; Rouhan et al. 2007; Tryon and Lugardon 2012; Tryon and Tryon 2012; Wu et al. 2021) (Table 1), whereas Nephrolepidaceae possess irregularly tuberculate, sometimes globular ornamentation (Tryon and Lugardon 2012) (Table 1). The cristate perispore of *Crypotocaulon* therefore is an additional diagnostic character that separates it from the closely related families.

### Taxonomic treatments

**Cryptocaulaceae** Sundue, T.Fujiw., Limpanasittichai & L.Y.Kuo **fam. nov.** – Type: *C. tenerifrons* (Hook.) Limpanasittichai, Yoneoka, Ebihara & L.Y.Kuo.

Diagnosis.—Distinguished from all other families of Polypodiineae by the combination of a creeping carnose rhizome, subclathrate scales bearing multicellular hairs, pubescent fronds, anastomosing veins, minute deltoid to lanceolate fugacious indusium composed of scarious-greenish-white lobes, and perispore cristate, forming cristate ridges that vary from short to elongate.

Description. —Rhizome creeping, somewhat carnose, densely scaly, dictyostelic; scales deltoid to lanceolate, basifixed, clathrate with isodiametric cells, with long multicellular hairs on the base of the abaxial surface; fronds remote; stipes stramineous, sparsely hairy with short catenate hairs, the base bearing narrowly lanceolate, clathrate scales, with 2–4 vascular bundles arranged in a semi-circle. Lamina pentagonal, 2-or 3-pinnatifid, thinly herbaceous, covered with short hairs on both surfaces, the hairs, articulate, acicular; free lateral pinnae 1 to 3 pairs, pinna oblong-subdeltoid, falcate, basiscopically strongly produced, terminal pinna oblong-subdeltoid, deeply pinnatifid; costa and costules densely hairy, veins copiously anastomosing, forming multiple rows of areoles along costa and costules, without included veinlets. Sori round to oblong, dorsal upon junctions of anastomosing veins, without paraphyses, indusiate. Indusia minute, fugacious, deltoid to lanceolate, composed of scarious-greenish-white lobes. Sporangia glabrous, each with 64 spores inside. Spores sub-ellipsoid, monolete, perispore cristate, forming cristate ridges that vary from short to elongate, areas between the cristae irregularly reticulate. Chromosome base number, *x* = 41.

Distribution.—Cambodia, Laos, Myanmar, Thailand, and Vietnam.

Etymology.—The name Cryptocaulaceae is taken from the habit of its rhizome, which is hidden deeply inside limestone fissures (Greek *kruptós* = hidden, + *kaulós* = stem, rhizome).

***Cryptocaulon*** Vongthavone, Tagane, Sundue & T.Fujiw., **gen. nov.** –Type: *C. tenerifrons* (Hook.) Limpanasittichai, Yoneoka, Ebihara & L.Y.Kuo, comb. nov. (≡ *Polypodium tenerifrons* Hook.).

Diagnosis.—Distinguished from *Tectaria* by having creeping carnose rhizome, subclathrate rhizome scales with isodiametric cells, bearing multicellular hairs (versus concolorous rhizome scales without hairs), the anastomosing veins forming multiple rows of areoles without free included veinlets (versus free to anastomosing forming areoles with free included veinlets), the basal veinlets of pinna-lobes on the basiscopic side arise from pinnule costule (versus the basal veinlets of pinna-lobes on the basiscopic side arise from pinna-rachis), and cristate perispore forming cristate ridges that vary from short to elongate (versus inflated folds, cristate wings, or echinate), and the basic chromosome number, *x* = 41 (versus *x* = 40).

Description.—Description as for the species.

***Cryptocaulon tenerifrons*** (Hook.) Limpanasittichai, Yoneoka, Ebihara & L.Y.Kuo, **comb. nov.≡** *Polypodium tenerifrons* Hook. in Sp. Fil. 5: 104 (1864) ≡ *Dictyopteris tenerifrons* (Hook.) Bedd. in Ferns Brit. India: t. 4 (1865) ≡ *Aspidium tenerifrons* (Hook.) Diels in H.G.A.Engler & K.A.E.Prantl, Nat. Pflanzenfam. 1(4): 186 (1899) ≡ *Tectaria tenerifrons* (Hook.) Ching in Sinensia 2: 34 (1931) – Type: Myanmar, Moulmeine, 1 Jul. 1860, *C.S.P. Parish 92* (**Lectotype** (**designated here**) K [K001080771 image! ], Isolectotypes E [E00417649 images! ], K [K001080769, K001080770, K001080772, K001080773 image! ]).

Description.—Plants epipetric. Rhizomes long-creeping, 2–3 mm in diameter, whitish, somewhat carnose, densely scaly, dictyostelic; rhizome scales deltoid to lanceolate, up to 2–3 mm long, basifixed, brown, clathrate with isodiametric cells, densely bearing long multicellular hairs along margins and on the adaxial surface, margin entire. Fronds remote, monomorphic, deciduous. Stipes 20–30 cm long, stramineous, sparsely covered with short articulate hairs, scaly near the base, with 2–4 vascular bundles arranged in a semi-circle; stipe scales lanceolate, up to 2–5 mm long, light brown, clathrate with narrow elongate cells, bearing long multicellular hairs along margins and on the adaxial surface. Lamina pentagonal, 2-or 3-pinnatifid, up to 30 cm long, 25 cm wide, thinly herbaceous, light green, sparsely covered with articulate hairs on both surfaces, the hairs acicular, 12-celled, occasionally 4-celled; free lateral pinnae 1 to 3 pairs, nearly opposite; basal pinnae the largest, 5–18 cm long, 6–10 cm wide, asymmetrically oblong-subdeltoid, falcate, basiscopically enlarged, with stalks up to 1 cm long, pinnatifid, apex acuminate, base rounded; basal basiscopic pinnule on basal pinna the largest, falcate, sometimes free, apex acuminate, margin deeply lobed; upper pinnule on basal pinna, falcate to lanceolate, apex acute to acuminate, margin crenate to deeply lobed; upper pinnae, 5–10 cm long, 2.5–4 cm wide, falcate, sessile to decurrent, base rounded to broadly cuneate, apex acute to acuminate, margin deeply lobed; terminal pinna oblong-subdeltoid, widest at the base, 10–15 cm long, 6–8 cm wide, deeply pinnatifid, apex acuminate to caudate, base decurrent; costa and costules densely hairy; veins copiously anastomosing, forming multiple rows of areoles along costa and costules, without free included veinlets, the terminal veins near margin free, densely hairy. Sori round to oblong, up to two times longer than wide, less than 1 mm in diam., dorsal upon junctions of anastomosing veins, in 1–2 rows along the costules in ultimate pinnules or lobes. Indusia attached proximally, minute, deltoid to lanceolate, comprised of scarious-greenish-white lobes, fugacious and only visible in very young leaves. Spores, 64 within sporangium, sub-ellipsoid, monolete, dark brown, perispore cristate, forming cristate ridges that vary from short to elongate, areas between the cristae irregularly reticulate. Chromosome number 2*n* = 82. Gametophyte cordate.

Distribution.—Cambodia, Laos, Myanmar, Thailand, and Vietnam.

Habitat.—On semi-shaded limestone walls.

*Note*.—In the protologue of *P. tenerifrons* (Hooker 1864), only one specimen from Myanmar (C.S.P. Parish 92) was cited. Our examination of the original material revealed that several sheets are deposited in the two herbaria (E [E00417649], K [K001080769, K001080770, K001080771, K001080772, K001080773]), and those are considered as syntypes. To secure the nomenclatural stability of *P. tenerifrons*, we here designate K001080771 as the lectotype, since it is only the sheet that contains a part of rhizome. The remaining sheets of the same specimen are isolectotypes.

Selected other specimens examined. —**CAMBODIA**. Kampot Prov.: 2 km north of Kampong Trach, midsection of Phnom Kampong Trach on East rim, 10.574722°N, 104.473056°E, 22 Jan. 2017, *McDonald et al. 8086* (TEX [TEX00575850]). **LAOS**. Bolikhamxai Prov.: Viengthong Distr., Ban Tha Pha, Nam Kading National Protected Area, 18.50047°N, 104.4659°E, alt 393 m, 6 Sep. 2023, *Tagane et al. Z479* (FOF [FOF0006993], KAG [KAG186913], VNM [VNM00071612]). Khammouane Prov.: Hinboun Distr., Walking trail on limestone hills nearby Khoun Kongleng, 17.6447°N, 104.81186°E, alt. 194 m, 6 Sep. 2024, *Tagane et al. Z1738* (FOF [FOF0007111], KAG [KAG188401], VNM); Khounkham Distr., Ban Khounphet, Phou Hin Poun National Park, on semi-shaded limestone walls, 18.05125°N, 104.44339°E, alt. 173 m, 27 Jun. 2025, *Tagane et al. Z2534* (FOF [FOF0007604], KAG [KAG202113], VNM); Khounkham Distr., in limestone karst, The Rock Viewpoint at Phou Pha Marn, 18.150798°N, 104.453809°E, 22 Jul. 2023, *Souladeth et al. PPM123* (FOF). Vientiane Prov.: Vang Vieng Distr., Ban Phon Nguen, along trail to the Pha Nguen View Point, 18.9225°N, 102.4179°E, alt. 410 m, 14 Sep. 2025, *Tagane et al. Z2766* (FOF, KAG [KAG202848], VNM). **THAILAND**. Chanthaburi Prov : Pong Nam Ron Dist., Nong Tha Kong, Wat Khao Kaeo, alt. 35–600 m, 28 Sep. 2011, *Boonkerd et al. 2011 − 408* (BCU [BCU004675]). Chiang Mai Prov.: Doi Muang Awn, west side, Sahagawn Subdistrict, Mae Awn Branch Dist., alt. 550 m, 29 Jul. 1998, *Palee 391* (L [L3613962]). Doi Muang Awn, west side, Sahagawn Subdistrict, Mae Awn Branch Dist., alt. 550 m, 15 Aug. 1998, *Palee 410* (L [L3613961]). Sankampang Dist., Muang On Cave, alt. 525 m, 29 Jul. 1989,* Maxwell* 89–955 (E [E00748988], L [L3613963]). Kamphaeng Phet Prov.: Phran Kratai Distr., Wat Tam Khao Tarom, 16.67748°N, 99.54092°E, alt. 88 m, 23 Nov. 2023, *Chamchumroon et al. VC5946* (BKF, FOF, KAG [KAG185196], KYO). Lampang Prov.: Doi Pang La, Huay Tak, alt. ca. 400 m, 25 Sep. 1967, *Shimizu et al. T10787* (E [E00748989], L [L3613968]). Tham Pha Thai in Huay Tak, alt. ca. 350 m, 23 Sep. 1967, *Tagawa et al. 10,637* (L [Lamphun Prov.: Doi Kuhn Dehn National Park, ridge above Tah Goo Station (Doi Tah Goo), alt. 550–600 m, 30 Aug. 1994,* Maxwell* 94–951 (L [L3613965]). Maehongson Prov.: 19°15′N, 98°E, alt. 500–600 m, 10 Sep. 1974, *Larsen & Larsen 34,340* (E [E00748990], L [L3613966]). Nan Prov.: Tham Phatup Forest Park, Trail to Phra Cave, 18°51′20″N, 100°44′5″E, alt. 300 m, 16 Aug. 2012, *Middleton et al. 5616* (E [E00726837]). Ratchaburi Prov.: Above Tapoh, on limestone rocks in mixed deciduous forest, alt. 250 m, Jun.-Jul. 1963, *Larsen 10,607* (L [L3613969]). Tak Prov.: Po Tip Tawng Cave Meditation Centre, Mae Kah Sah Subdistrict, alt 250 m, 20 Aug. 1994, *Maxwell 94–912* (L [L3613964]). Uthai Thani Prov.: Lan Sak Dist., Wat Khao Pha Rat, alt. 35–600 m, 24 May 2013, *Boonkerd et al. *2011 − 739 (BCU [BCU004728]). **VIETNAM**. KienGiang Prov.: Kien Luong Dist., Nui Chua Hang (Mt. Chua Hang), 10.1393°N, 104.6401°E, alt. 60 m, 3 Jul. 2024, *Tagane et al. N655* (FOF [FOF0009321], KAG [KAG188758], VNM [VNM00072732]).

## Supplementary Information

Below is the link to the electronic supplementary material.

Supplementary Material 1.
