# Supplementary material for: Cryptocaulaceae: a new and deeply diverged branch within the fern tree of life
Source: J Plant Res. 2026 Apr 17;139(3):365–83. doi: 10.1007/s10265-026-01698-0 (PMC13197324; doi:10.1007/s10265-026-01698-0)

Supporting Information

Cryptocaulaceae: A new and deeply diverged branch within the fern tree of life

Tao Fujiwara[1,*,†], Ponpipat Limpanasittichai[2,†], Katsuhiro Yoneoka[3], Shuichiro Tagane[4], Thyraphon Vongthavone[5], Takenori Yamamoto[6], Van Son Dang[7], Phetlasy Souladeth[8], Voradol Chamchumroon[9], Atsushi Ebihara[10], Michael Sundue[11], Li-Yaung Kuo[2,*]

[1]The Mt. Fuji Institute for Nature and Biology, Showa Medical University, Kamiyoshida 4562, Fujiyoshida, Yamanashi 4030005, Japan.

[2]Institute of Molecular & Cellular Biology, National Tsing Hua University, Hsinchu, 30013, Taiwan

[3]Center for Frontier Research, National Institute of Genetics, 1111 Yata, Mishima, Shizuoka, 4118540, Japan

[4]The Kagoshima University Museum, Kagoshima University, 1-21-30 Korimoto, Kagoshima, 890-0065, Japan.

[5]Association for Community Development (ACD), Salavan Province, Laos.

[6]Rural Regeneration Research Center, Yamagata University, 1-23 Wakaba-machi, Tsuruoka, 997-8555, Japan.

[7]Institute of Life Sciences, Vietnam Academy of Science and Technology, 85 Tran Quoc Toan Street, Xuan Hoa Ward, Ho Chi Minh City, Vietnam.

[8]Faculty of Forest Science, National University of Laos, Dongdok Campus, 01170, Xaythany District, Vientiane Capital, Laos.

[9]The Forest Herbarium (BKF), Department of National parks, Wildlife and Plant Conservation, Chatuchak, Bangkok, 10900, Thailand.

[10]Department of Botany, National Museum of Nature and Science, 4-1-1 Amakubo, Tsukuba-shi, Ibaraki 305-0005, Japan.

[11]Royal Botanic Garden Edinburgh, 20A Inverleith Row, Edinburgh, Scotland EH3 5LR, United Kingdom

† Joint first authors

*Correspondence Tao Fujiwara (tao.fujiwara@gmail.com); Li-Yaung Kuo (lykuo@life.nthu.edu.tw).

Contents

Supplementary Table S1. Phylogenomic sampling of this study. *sequences generated in this study.

Supplementary Table S2. Plastid sequences used in the species-level phylogeny of this study.

Supplementary Table S3. Primer sets used in this study

Supplementary Fig. S1. Species tree inferred from individual gene trees of 86 plastid CDSs.

Supplementary Fig. S2. The full species-level plastid tree based on 6 plastid regions, *atpA*, *atpB*, *rbcL*, *rps4-trnS*, and *trnL-F*.

Table S1. Phylogenomic sampling of this study. *sequences generated in this study.

Suborder	Family (subfamily)	Genus	Plastome GenBank accession (	Transcriptome reference (SRA)	Voucher of newly generatedly sequences: collection no. (herbarium) / locality
Polypodiineae	Cryptocaulaceae	*Cryptocaulon*	PV931327 (SRR34754126)*	This study (SRR34754125)*	P. Limpanasittichai 240706-2 (TAIF) / Nong Ta Khong, Pong Nam Ron Dist., Chanthaburi Prov. Thailand
Polypodiineae	Cryptocaulaceae	*Cryptocaulon*	PV931326 (SRR34754127)*	PV931326 (SRR34754128)*	T. Boonkerd et al. 2011-739 (BCU) / Lan Sak, Thailand
Polypodiineae	Arthropteridaceae	*Arthropteris*	MT130588	Qi et al. (2018) (SRR6920693)	
Polypodiineae	Davalliaceae	*Davallia*	MK761241	Qi et al. (2018) (SRR6920691)	
Polypodiineae	Didymochlaenaceae	*Didymochlaena*	MT130600	Qi et al. (2018) (SRR6920662)	
Polypodiineae	Dryopteridaceae (Ctenitidoideae)	*Ctenitis*	MT130665	Qi et al. (2018) (SRR6920661)	
Polypodiineae	Dryopteridaceae (Dryopteridoideae)	*Dryopteris*	KY427348	Qi et al. (2018) (SRR6920660)	
Polypodiineae	Dryopteridaceae (Dryopteridoideae)	*Cyrtomium*	MT130683	Qi et al. (2018) (SRR6920659)	
Polypodiineae	Dryopteridaceae (Dryopteridoideae)	*Polystichum*	MN712466	Qi et al. (2018) (SRR6920645)	
Polypodiineae	Dryopteridaceae (Dryopteridoideae)	*Arachniodes*	MT130551	Shen et al. (2018) (SRR2103702)	
Polypodiineae	Dryopteridaceae (Elaphoglossoideae)	*Bolbitis*	MT130550	Qi et al. (2018) (SRR6920653)	
Polypodiineae	Dryopteridaceae (Elaphoglossoideae)	*Elaphoglossum*	ON898018	Qi et al. (2018) (SRR6920651)	
Polypodiineae	Dryopteridaceae (Elaphoglossoideae)	*Lomagramma*	OP298004	Shen et al. (2018) (SRR2103704)	
Polypodiineae	Dryopteridaceae (Lastreopsidoideae)	*Lastreopsis*	OR530053	This study (SRR34754124)*	Lu31571 (TAIF) / Nantou County, Taiwan
Polypodiineae	Dryopteridaceae (Pleocnemioideae)	*Pleocnemia*	MT130681	Qi et al. (2018) (SRR6920646)	
Polypodiineae	Hypodematiaceae	*Leucostegia*	MT130653	1KP (2019)	
Polypodiineae	Hypodematiaceae	*Hypodematium*	MT130540	Qi et al. (2018) (SRR6920655)	
Polypodiineae	Lomariopsidaceae	*Cyclopeltis*	MT130541	Qi et al. (2018) (SRR6920654)	
Polypodiineae	Lomariopsidaceae	*Lomariopsis*	MT130608	This study (SRR34754123)*	Kuo4552 (TAIF) / Orchid Is., Taiwan
Polypodiineae	Nephrolepidaceae	*Nephrolepis*	MT130615	Qi et al. (2018) (SRR6920648)	
Polypodiineae	Oleandraceae	*Oleandra*	MT130650	Qi et al. (2018) (SRR6920692)	
Polypodiineae	Polypodiaceae (Crypsinoideae)	*Drynaria*	MK761238	Qi et al. (2018) (SRR6920674)	
Polypodiineae	Polypodiaceae (Crypsinoideae)	*Selliguea*	MT130554	Qi et al. (2018) (SRR6920670)	
Polypodiineae	Polypodiaceae (Grammitidoideae)	*Oreogrammitis*	MT130563	Qi et al. (2018) (SRR6920688)	
Polypodiineae	Polypodiaceae (Grammitidoideae)	*Prosaptia*	MW876357	Qi et al. (2018) (SRR6920687)	
Polypodiineae	Polypodiaceae (Loxogrammoideae)	*Loxogramme*	MT130671	Qi et al. (2018) (SRR6920686)	
Polypodiineae	Polypodiaceae (Microsoroideae)	*Lepisorus*	MT130651	Qi et al. (2018) (SRR6920665)	
Polypodiineae	Polypodiaceae (Microsoroideae)	*Leptochilus*	MT130679	Qi et al. (2018) (SRR6369206)	
Polypodiineae	Polypodiaceae (Microsoroideae)	*Bosmania*	MT130574	Qi et al. (2018) (SRR6920666)	
Polypodiineae	Polypodiaceae (Microsoroideae)	*Microsorum*	MW876342	Qi et al. (2018) (SRR6920668)	
Polypodiineae	Polypodiaceae (Microsoroideae)	*Goniophlebium*	MW876323	Shen et al. (2018) (SRR2103725)	
Polypodiineae	Polypodiaceae (Platycerioideae)	*Platycerium*	OR601546	Qi et al. (2018) (SRR6920672)	
Polypodiineae	Polypodiaceae (Platycerioideae)	*Pyrrosia*	MT130682	Qi et al. (2018) (SRR6920669)	
Polypodiineae	Polypodiaceae (Polypodioideae)	*Polypodium*	MT984517	1KP (2019)	
Polypodiineae	Polypodiaceae (Polypodioideae)	*Pleopeltis*	MW876353	1KP (2019)	
Polypodiineae	Polypodiaceae (Polypodioideae)	*Pleurosoriopsis*	MW876354	Shen et al. (2018) (SRR2103723)	
Polypodiineae	Polypodiaceae (Polypodioideae)	*Phlebodium*	MW876348	1KP (2019)	
Polypodiineae	Pteridryaceae	*Pteridrys*	MT130579	Qi et al. (2018) (SRR6920649)	
Polypodiineae	Tectariaceae	*Tectaria*	MT130620	Qi et al. (2018) (SRR6920694)	
Aspleniineae	Diplaziopsidaceae	*Diplaziopsis*	KY427341	Qi et al. (2018) (SRR6920710)	
Pteridineae	Pteridaceae	*Acrostichum*	MT026711	Qi et al. (2018) (SRR6920722)	

Table S2. Plastid sequences used in the species-levelphylogeny of this study.

Family	Species	Plastid regio	GenBank access	Source / collection no. (herbarium) / locality	Note
Cryptocaulaceae	Cryptocaulon tenerifrons (Hook.)	plastome	PV931327	This study / T. Boonkerd et al. 2011-739 (BCU) / Lan Sak, Thailand	KTHU2823
Cryptocaulaceae	Cryptocaulon tenerifrons (Hook.)	plastome	PV931326	This study / P. Limpanasittichai 240706-2 (TAIF) / Nong Ta Khong, Pong Nam Ron Dist., Chanthaburi Prov. Thailand	KTHU2741
Cryptocaulaceae	Cryptocaulon tenerifrons (Hook.)	atpA	LC907086	This study / Tagane et al. Z655 (KAG, FOF, VNM)/ Nui Chua Hang, Kien Luong Dist., Kien Giang Prov., Vietnam	N655_Vietnam
Cryptocaulaceae	Cryptocaulon tenerifrons (Hook.)	atpB	LC907090	This study / Tagane et al. Z655 (KAG, FOF, VNM)/ Nui Chua Hang, Kien Luong Dist., Kien Giang Prov., Vietnam	N655_Vietnam
Cryptocaulaceae	Cryptocaulon tenerifrons (Hook.)	rbcL	LC907082	This study / Tagane et al. Z655 (KAG, FOF, VNM)/ Nui Chua Hang, Kien Luong Dist., Kien Giang Prov., Vietnam	N655_Vietnam
Cryptocaulaceae	Cryptocaulon tenerifrons (Hook.)	rps4+rps4-tr	LC907094	This study / Tagane et al. Z655 (KAG, FOF, VNM)/ Nui Chua Hang, Kien Luong Dist., Kien Giang Prov., Vietnam	N655_Vietnam
Cryptocaulaceae	Cryptocaulon tenerifrons (Hook.)	trnL-trnF	LC907097	This study / Tagane et al. Z655 (KAG, FOF, VNM)/ Nui Chua Hang, Kien Luong Dist., Kien Giang Prov., Vietnam	N655_Vietnam
Cryptocaulaceae	Cryptocaulon tenerifrons (Hook.)	atpA	LC907087	This study /Chamchumroon et al. VC5946 (BKF, KAG)/ Wat Tam Khao Tarom, Phran Kratai District, Kamphaeng Phet Prov., Thailand	VC4956_Thailand_merged_VC5946_Thailand
Cryptocaulaceae	Cryptocaulon tenerifrons (Hook.)	atpB	LC907091	This study /Chamchumroon et al. VC5946 (BKF, KAG)/ Wat Tam Khao Tarom, Phran Kratai District, Kamphaeng Phet Prov., Thailand	VC4956_Thailand_merged_VC5946_Thailand
Cryptocaulaceae	Cryptocaulon tenerifrons (Hook.)	rbcL	LC907083	This study /Chamchumroon et al. VC5946 (BKF, KAG)/ Wat Tam Khao Tarom, Phran Kratai District, Kamphaeng Phet Prov., Thailand	VC4956_Thailand_merged_VC5946_Thailand
Cryptocaulaceae	Cryptocaulon tenerifrons (Hook.)	rps4+rps4-tr	LC907095	This study /Chamchumroon et al. VC5946 (BKF, KAG)/ Wat Tam Khao Tarom, Phran Kratai District, Kamphaeng Phet Prov., Thailand	VC4956_Thailand_merged_VC5946_Thailand
Cryptocaulaceae	Cryptocaulon tenerifrons (Hook.)	trnL-trnF	LC907098	This study /Chamchumroon et al. VC5946 (BKF, KAG)/ Wat Tam Khao Tarom, Phran Kratai District, Kamphaeng Phet Prov., Thailand	VC4956_Thailand_merged_VC5946_Thailand
Cryptocaulaceae	Cryptocaulon tenerifrons (Hook.)	atpA	LC907088	This study /Tagane et al. Z1738 (KAG, FOF, VNM)/ Walking trail on limestone hills nearby Khoun Kongleng, Hinboun Dist., Khammouan Prov., Laos	Z1738_Laos
Cryptocaulaceae	Cryptocaulon tenerifrons (Hook.)	atpB	LC907092	This study /Tagane et al. Z1738 (KAG, FOF, VNM)/ Walking trail on limestone hills nearby Khoun Kongleng, Hinboun Dist., Khammouan Prov., Laos	Z1738_Laos
Cryptocaulaceae	Cryptocaulon tenerifrons (Hook.)	rbcL	LC907084	This study /Tagane et al. Z1738 (KAG, FOF, VNM)/ Walking trail on limestone hills nearby Khoun Kongleng, Hinboun Dist., Khammouan Prov., Laos	Z1738_Laos
Cryptocaulaceae	Cryptocaulon tenerifrons (Hook.)	rps4+rps4-tr	LC907096	This study /Tagane et al. Z1738 (KAG, FOF, VNM)/ Walking trail on limestone hills nearby Khoun Kongleng, Hinboun Dist., Khammouan Prov., Laos	Z1738_Laos
Cryptocaulaceae	Cryptocaulon tenerifrons (Hook.)	trnL-trnF	LC907099	This study /Tagane et al. Z1738 (KAG, FOF, VNM)/ Walking trail on limestone hills nearby Khoun Kongleng, Hinboun Dist., Khammouan Prov., Laos	Z1738_Laos
Cryptocaulaceae	Cryptocaulon tenerifrons (Hook.)	atpA	LC907085	This study /Tagane et al. Z479 (KAG, FOF, VNM)/ Nam Kading National Protected Area, Ban Tha Pha, Viengthong Dist., Bolikhamxai Prov., Laos	Z479_Laos_merged_z479_Laos
Cryptocaulaceae	Cryptocaulon tenerifrons (Hook.)	atpB	LC907089	This study /Tagane et al. Z479 (KAG, FOF, VNM)/ Nam Kading National Protected Area, Ban Tha Pha, Viengthong Dist., Bolikhamxai Prov., Laos	Z479_Laos_merged_z479_Laos
Cryptocaulaceae	Cryptocaulon tenerifrons (Hook.)	rbcL	LC907081	This study /Tagane et al. Z479 (KAG, FOF, VNM)/ Nam Kading National Protected Area, Ban Tha Pha, Viengthong Dist., Bolikhamxai Prov., Laos	Z479_Laos_merged_z479_Laos
Cryptocaulaceae	Cryptocaulon tenerifrons (Hook.)	rps4+rps4-tr	LC907093	This study /Tagane et al. Z479 (KAG, FOF, VNM)/ Nam Kading National Protected Area, Ban Tha Pha, Viengthong Dist., Bolikhamxai Prov., Laos	Z479_Laos_merged_z479_Laos
Arthropteridacea	Arthropteris altescandens (Colla) J. Sm.	rbcL	KF667641	FTOL v1.7	
Arthropteridacea	Arthropteris altescandens (Colla) J. Sm.	rps4-trnS	KF667554	FTOL v1.7	
Arthropteridacea	Arthropteris altescandens (Colla) J. Sm.	trnL-trnF	KF667610	FTOL v1.7	
Arthropteridacea	Arthropteris articulata (Brack.) C. Chr.	atpA	KC977329	FTOL v1.7	
Arthropteridacea	Arthropteris articulata (Brack.) C. Chr.	atpB	KC977348	FTOL v1.7	
Arthropteridacea	Arthropteris articulata (Brack.) C. Chr.	rbcL	KC977367	FTOL v1.7	
Arthropteridacea	Arthropteris articulata (Brack.) C. Chr.	trnL-trnF	KC977411	FTOL v1.7	
Arthropteridacea	Arthropteris beckleri (Hook.) Mett.	atpB	AB212686	FTOL v1.7	
Arthropteridacea	Arthropteris beckleri (Hook.) Mett.	rbcL	AB212686	FTOL v1.7	
Arthropteridacea	Arthropteris cameroonensis Alston	rbcL	KF667638	FTOL v1.7	
Arthropteridacea	Arthropteris cameroonensis Alston	rps4-trnS	KF667551	FTOL v1.7	
Arthropteridacea	Arthropteris monocarpa (Cordem.) C. Chr.	atpA	KF897993	FTOL v1.7	
Arthropteridacea	Arthropteris monocarpa (Cordem.) C. Chr.	rbcL	KF887155	FTOL v1.7	
Arthropteridacea	Arthropteris monocarpa (Cordem.) C. Chr.	trnL-trnF	KF897941	FTOL v1.7	
Arthropteridacea	Arthropteris orientalis (J. F. Gmel.) Posth.	atpB	KC977357	FTOL v1.7	
Arthropteridacea	Arthropteris orientalis (J. F. Gmel.) Posth.	rbcL	KC977378	FTOL v1.7	
Arthropteridacea	Arthropteris orientalis (J. F. Gmel.) Posth.	trnL-trnF	KC977420	FTOL v1.7	
Arthropteridacea	Arthropteris palisotii (Desv.) Alston	plastome	MT130588	FTOL v1.7	
Arthropteridacea	Arthropteris parallela (Baker) C. Chr.	atpA	EF463862	FTOL v1.7	
Arthropteridacea	Arthropteris parallela (Baker) C. Chr.	atpB	EF463522	FTOL v1.7	
Arthropteridacea	Arthropteris parallela (Baker) C. Chr.	rbcL	EF463266	FTOL v1.7	
Arthropteridacea	Arthropteris parallela (Baker) C. Chr.	trnL-trnF	KC977425	FTOL v1.7	
Arthropteridacea	Arthropteris paucivenia (C. Chr.) H. M. Liu, Hovenkamp	atpA	EF463864	FTOL v1.7	
Arthropteridacea	Arthropteris paucivenia (C. Chr.) H. M. Liu, Hovenkamp	atpB	EF463524	FTOL v1.7	
Arthropteridacea	Arthropteris paucivenia (C. Chr.) H. M. Liu, Hovenkamp	rbcL	EF463268	FTOL v1.7	
Arthropteridacea	Arthropteris paucivenia (C. Chr.) H. M. Liu, Hovenkamp	trnL-trnF	KC977426	FTOL v1.7	
Arthropteridacea	Arthropteris submarginalis Domin	atpA	KC977339	FTOL v1.7	
Arthropteridacea	Arthropteris submarginalis Domin	atpB	KC977360	FTOL v1.7	
Arthropteridacea	Arthropteris submarginalis Domin	rbcL	KC977381	FTOL v1.7	
Arthropteridacea	Arthropteris submarginalis Domin	trnL-trnF	KC977423	FTOL v1.7	
Arthropteridacea	Arthropteris tenella (G. Forst.) J. Sm.	atpA	KF897998	FTOL v1.7	
Arthropteridacea	Arthropteris tenella (G. Forst.) J. Sm.	rbcL	KF887160	FTOL v1.7	
Arthropteridacea	Arthropteris tenella (G. Forst.) J. Sm.	trnL-trnF	KF897946	FTOL v1.7	
Davalliaceae	Davallia assamica (Bedd.) Baker	plastome	MT130637	FTOL v1.7	
Davalliaceae	Davallia beddomei Hope	atpB	MH392510	FTOL v1.7	
Davalliaceae	Davallia beddomei Hope	rbcL	MH392510	FTOL v1.7	
Davalliaceae	Davallia borneensis (Hook.) J. Sm.	atpB	AB212694	FTOL v1.7	
Davalliaceae	Davallia borneensis (Hook.) J. Sm.	rbcL	AB212694	FTOL v1.7	
Davalliaceae	Davallia bullata Wall.	atpB	JX103675	FTOL v1.7	
Davalliaceae	Davallia bullata Wall.	rbcL	JX103717	FTOL v1.7	
Davalliaceae	Davallia bullata Wall.	rps4	JX103759	FTOL v1.7	
Davalliaceae	Davallia bullata Wall.	rps4-trnS	JX103759	FTOL v1.7	
Davalliaceae	Davallia bullata Wall.	trnL-trnF	JX103801	FTOL v1.7	
Davalliaceae	Davallia canariensis (L.) Sm.	atpB	MH392512	FTOL v1.7	
Davalliaceae	Davallia canariensis (L.) Sm.	rbcL	LC177751	FTOL v1.7	
Davalliaceae	Davallia canariensis (L.) Sm.	trnL-trnF	KF601953	FTOL v1.7	
Davalliaceae	Davallia chaerophylloides (Poir.) Steud.	atpB	AB300575	FTOL v1.7	
Davalliaceae	Davallia chaerophylloides (Poir.) Steud.	rbcL	AB300575	FTOL v1.7	
Davalliaceae	Davallia chrysanthemifolia Hayata	atpB	LC177752	FTOL v1.7	
Davalliaceae	Davallia chrysanthemifolia Hayata	rbcL	LC177752	FTOL v1.7	
Davalliaceae	Davallia corniculata T. Moore	atpB	AB212697	FTOL v1.7	
Davalliaceae	Davallia corniculata T. Moore	rbcL	AB212697	FTOL v1.7	
Davalliaceae	Davallia cumingii Hook.	atpB	LC177755	FTOL v1.7	
Davalliaceae	Davallia cumingii Hook.	rbcL	LC177755	FTOL v1.7	
Davalliaceae	Davallia denticulata (Burm. fil.) Mett. ex Kuhn	atpB	AB212698	FTOL v1.7	
Davalliaceae	Davallia denticulata (Burm. fil.) Mett. ex Kuhn	rbcL	AB212698	FTOL v1.7	
Davalliaceae	Davallia divaricata Blume	plastome	MT130639	FTOL v1.7	
Davalliaceae	Davallia embolostegia Copel.	atpB	AB212701	FTOL v1.7	
Davalliaceae	Davallia embolostegia Copel.	rbcL	AB212701	FTOL v1.7	
Davalliaceae	Davallia epiphylla (G. Forst.) Spreng.	atpB	AB212702	FTOL v1.7	
Davalliaceae	Davallia epiphylla (G. Forst.) Spreng.	rbcL	AB212702	FTOL v1.7	
Davalliaceae	Davallia faberiana (C. Chr.) comb. ined.	atpB	AB212688	FTOL v1.7	
Davalliaceae	Davallia faberiana (C. Chr.) comb. ined.	rbcL	AB212688	FTOL v1.7	
Davalliaceae	Davallia fejeensis Hook.	plastome	MK705750	FTOL v1.7	
Davalliaceae	Davallia griffithiana Hook.	atpA	EF463649	FTOL v1.7	
Davalliaceae	Davallia griffithiana Hook.	atpB	EF463371	FTOL v1.7	
Davalliaceae	Davallia griffithiana Hook.	rbcL	EF463165	FTOL v1.7	
Davalliaceae	Davallia heterophylla Sm.	atpB	AB289539	FTOL v1.7	
Davalliaceae	Davallia heterophylla Sm.	rbcL	AB289539	FTOL v1.7	
Davalliaceae	Davallia hookeri (Bedd.) X. C. Zhang	atpB	MH392501	FTOL v1.7	
Davalliaceae	Davallia hookeri (Bedd.) X. C. Zhang	rbcL	MH392501	FTOL v1.7	
Davalliaceae	Davallia hymenophylloides (Blume) Kuhn	atpB	AB212689	FTOL v1.7	
Davalliaceae	Davallia hymenophylloides (Blume) Kuhn	rbcL	AB212689	FTOL v1.7	
Davalliaceae	Davallia kansuensis (Ching) X.D.Ma & F.G.Wang.	atpB	MH392503	FTOL v1.7	
Davalliaceae	Davallia kansuensis (Ching) X.D.Ma & F.G.Wang.	rbcL	MH392503	FTOL v1.7	
Davalliaceae	Davallia membranulosa Wall.	atpB	MH392504	FTOL v1.7	
Davalliaceae	Davallia membranulosa Wall.	rbcL	MH392504	FTOL v1.7	
Davalliaceae	Davallia multidentata Wall. ex Hook. & Baker	atpB	AB289538	FTOL v1.7	
Davalliaceae	Davallia multidentata Wall. ex Hook. & Baker	rbcL	AB289538	FTOL v1.7	
Davalliaceae	Davallia parvula Wall.	atpB	AB212718	FTOL v1.7	
Davalliaceae	Davallia parvula Wall.	rbcL	AB212718	FTOL v1.7	
Davalliaceae	Davallia pectinata Sm.	atpB	LC177753	FTOL v1.7	
Davalliaceae	Davallia pectinata Sm.	rbcL	LC177753	FTOL v1.7	
Davalliaceae	Davallia pentaphylla Blume	atpB	AB212723	FTOL v1.7	
Davalliaceae	Davallia pentaphylla Blume	rbcL	AB212723	FTOL v1.7	
Davalliaceae	Davallia perdurans Christ	plastome	MT130656	FTOL v1.7	
Davalliaceae	Davallia platylepis Baker	atpB	MH392497	FTOL v1.7	
Davalliaceae	Davallia platylepis Baker	rbcL	MH392497	FTOL v1.7	
Davalliaceae	Davallia polypodioides D. Don	atpB	AB212720	FTOL v1.7	
Davalliaceae	Davallia polypodioides D. Don	rbcL	AB212720	FTOL v1.7	
Davalliaceae	Davallia pubescens C. W. Chen	atpB	AB212696	FTOL v1.7	
Davalliaceae	Davallia pubescens C. W. Chen	rbcL	AB212696	FTOL v1.7	
Davalliaceae	Davallia pubescens C. W. Chen	rps4	AY096213	FTOL v1.7	
Davalliaceae	Davallia pubescens C. W. Chen	rps4-trnS	AY096213	FTOL v1.7	
Davalliaceae	Davallia pulchra D. Don	atpB	AB212692	FTOL v1.7	
Davalliaceae	Davallia pulchra D. Don	rbcL	AB212692	FTOL v1.7	
Davalliaceae	Davallia pusilla Mett.	rbcL	MT657553	FTOL v1.7	
Davalliaceae	Davallia pyxidata Cav.	atpB	AB212711	FTOL v1.7	
Davalliaceae	Davallia pyxidata Cav.	rbcL	AB212711	FTOL v1.7	
Davalliaceae	Davallia repens (L. fil.) Kuhn	plastome	MT130578	FTOL v1.7	
Davalliaceae	Davallia sessilifolia Blume	rbcL	ON365802	FTOL v1.7	
Davalliaceae	Davallia sinensis (Christ) Ching	atpB	MH392492	FTOL v1.7	
Davalliaceae	Davallia sinensis (Christ) Ching	rbcL	MH392492	FTOL v1.7	
Davalliaceae	Davallia solida (G. Forst.) Sw.	atpB	AB212710	FTOL v1.7	
Davalliaceae	Davallia solida (G. Forst.) Sw.	rbcL	AB212710	FTOL v1.7	
Davalliaceae	Davallia tasmanii Field	atpB	AB212713	FTOL v1.7	
Davalliaceae	Davallia tasmanii Field	rbcL	AB212713	FTOL v1.7	
Davalliaceae	Davallia trichomanoides Blume	atpB	AB212708	FTOL v1.7	
Davalliaceae	Davallia trichomanoides Blume	rbcL	AB212708	FTOL v1.7	
Davalliaceae	Davallia triphylla Hook.	atpB	AB212725	FTOL v1.7	
Davalliaceae	Davallia triphylla Hook.	rbcL	AB212725	FTOL v1.7	
Davalliaceae	Davallia tyermannii (T. Moore) H. J. Veitch	plastome	MK761241	FTOL v1.7	
Davalliaceae	Davallia yunnanensis Christ	atpB	JX103676	FTOL v1.7	
Davalliaceae	Davallia yunnanensis Christ	rbcL	JX103718	FTOL v1.7	
Davalliaceae	Davallia yunnanensis Christ	rps4	JX103760	FTOL v1.7	
Davalliaceae	Davallia yunnanensis Christ	rps4-trnS	JX103760	FTOL v1.7	
Davalliaceae	Davallia yunnanensis Christ	trnL-trnF	JX103802	FTOL v1.7	
Didymochlaenace	Didymochlaena alpina Li Bing Zhang & H.Shang	atpA	OP595261	FTOL v1.7	
Didymochlaenace	Didymochlaena alpina Li Bing Zhang & H.Shang	atpB	OP595231	FTOL v1.7	
Didymochlaenace	Didymochlaena alpina Li Bing Zhang & H.Shang	rbcL	OP595169	FTOL v1.7	
Didymochlaenace	Didymochlaena alpina Li Bing Zhang & H.Shang	rps4	OP595322	FTOL v1.7	
Didymochlaenace	Didymochlaena alpina Li Bing Zhang & H.Shang	rps4-trnS	OP595322	FTOL v1.7	
Didymochlaenace	Didymochlaena alpina Li Bing Zhang & H.Shang	trnL-trnF	OP595324	FTOL v1.7	
Didymochlaenace	Didymochlaena amazonica Li Bing Zhang & H.Shang	atpA	OP595260	FTOL v1.7	
Didymochlaenace	Didymochlaena amazonica Li Bing Zhang & H.Shang	atpB	OP595230	FTOL v1.7	
Didymochlaenace	Didymochlaena amazonica Li Bing Zhang & H.Shang	rbcL	OP595168	FTOL v1.7	
Didymochlaenace	Didymochlaena amazonica Li Bing Zhang & H.Shang	rps4	OP595293	FTOL v1.7	
Didymochlaenace	Didymochlaena amazonica Li Bing Zhang & H.Shang	rps4-trnS	OP595293	FTOL v1.7	
Didymochlaenace	Didymochlaena amazonica Li Bing Zhang & H.Shang	trnL-trnF	OP595323	FTOL v1.7	
Didymochlaenace	Didymochlaena attenuata (Bonap.) Li Bing Zhang & H.	atpA	OP595279	FTOL v1.7	
Didymochlaenace	Didymochlaena attenuata (Bonap.) Li Bing Zhang & H.	atpB	OP595249	FTOL v1.7	
Didymochlaenace	Didymochlaena attenuata (Bonap.) Li Bing Zhang & H.	rbcL	OP595187	FTOL v1.7	
Didymochlaenace	Didymochlaena attenuata (Bonap.) Li Bing Zhang & H.	rps4	OP595314	FTOL v1.7	
Didymochlaenace	Didymochlaena attenuata (Bonap.) Li Bing Zhang & H.	rps4-trnS	OP595314	FTOL v1.7	
Didymochlaenace	Didymochlaena attenuata (Bonap.) Li Bing Zhang & H.	trnL-trnF	OP595342	FTOL v1.7	
Didymochlaenace	Didymochlaena bipinnatipartita (Bonap.) Li Bing Zhang	atpA	OP595271	FTOL v1.7	
Didymochlaenace	Didymochlaena bipinnatipartita (Bonap.) Li Bing Zhang	atpB	OP595241	FTOL v1.7	
Didymochlaenace	Didymochlaena bipinnatipartita (Bonap.) Li Bing Zhang	rbcL	OP595179	FTOL v1.7	
Didymochlaenace	Didymochlaena bipinnatipartita (Bonap.) Li Bing Zhang	rps4	OP595306	FTOL v1.7	
Didymochlaenace	Didymochlaena bipinnatipartita (Bonap.) Li Bing Zhang	rps4-trnS	OP595306	FTOL v1.7	
Didymochlaenace	Didymochlaena bipinnatipartita (Bonap.) Li Bing Zhang	trnL-trnF	OP595334	FTOL v1.7	
Didymochlaenace	Didymochlaena cameroonensis Li Bing Zhang & H.Shar	atpA	OP595268	FTOL v1.7	
Didymochlaenace	Didymochlaena cameroonensis Li Bing Zhang & H.Shar	atpB	OP595238	FTOL v1.7	
Didymochlaenace	Didymochlaena cameroonensis Li Bing Zhang & H.Shar	rbcL	OP595176	FTOL v1.7	
Didymochlaenace	Didymochlaena cameroonensis Li Bing Zhang & H.Shar	rps4	OP595303	FTOL v1.7	
Didymochlaenace	Didymochlaena cameroonensis Li Bing Zhang & H.Shar	rps4-trnS	OP595303	FTOL v1.7	
Didymochlaenace	Didymochlaena cameroonensis Li Bing Zhang & H.Shar	trnL-trnF	OP595331	FTOL v1.7	
Didymochlaenace	Didymochlaena deltoidea Li Bing Zhang & H. Shang	atpA	OP595275	FTOL v1.7	
Didymochlaenace	Didymochlaena deltoidea Li Bing Zhang & H. Shang	atpB	OP595245	FTOL v1.7	
Didymochlaenace	Didymochlaena deltoidea Li Bing Zhang & H. Shang	rbcL	OP595183	FTOL v1.7	
Didymochlaenace	Didymochlaena deltoidea Li Bing Zhang & H. Shang	rps4	OP595310	FTOL v1.7	
Didymochlaenace	Didymochlaena deltoidea Li Bing Zhang & H. Shang	rps4-trnS	OP595310	FTOL v1.7	
Didymochlaenace	Didymochlaena deltoidea Li Bing Zhang & H. Shang	trnL-trnF	OP595338	FTOL v1.7	
Didymochlaenace	Didymochlaena dimidiata Kunze	atpA	OP595288	FTOL v1.7	
Didymochlaenace	Didymochlaena dimidiata Kunze	rbcL	OP595197	FTOL v1.7	
Didymochlaenace	Didymochlaena fijiensis Li Bing Zhang & H. Shang	atpA	MW323337	FTOL v1.7	
Didymochlaenace	Didymochlaena fijiensis Li Bing Zhang & H. Shang	atpB	MW323324	FTOL v1.7	
Didymochlaenace	Didymochlaena fijiensis Li Bing Zhang & H. Shang	rbcL	MW323312	FTOL v1.7	
Didymochlaenace	Didymochlaena fijiensis Li Bing Zhang & H. Shang	rps4	MW323362	FTOL v1.7	
Didymochlaenace	Didymochlaena fijiensis Li Bing Zhang & H. Shang	rps4-trnS	MW323362	FTOL v1.7	
Didymochlaenace	Didymochlaena fijiensis Li Bing Zhang & H. Shang	trnL-trnF	MW323373	FTOL v1.7	
Didymochlaenace	Didymochlaena madagascariensis Li Bing Zhang & H. S	atpA	OP595277	FTOL v1.7	
Didymochlaenace	Didymochlaena madagascariensis Li Bing Zhang & H. S	atpB	OP595247	FTOL v1.7	
Didymochlaenace	Didymochlaena madagascariensis Li Bing Zhang & H. S	rbcL	OP595185	FTOL v1.7	

Didymochlaenace	Didymochlaena madagascariensis Li Bing Zhang & H. S	rps4	OP595312	FTOL v1.7
Didymochlaenace	Didymochlaena madagascariensis Li Bing Zhang & H. S	rps4-trnS	OP595312	FTOL v1.7
Didymochlaenace	Didymochlaena madagascariensis Li Bing Zhang & H. S	trnL-trnF	OP595340	FTOL v1.7
Didymochlaenace	Didymochlaena mesoamericana Li Bing Zhang & H.Sha	atpA	OP595263	FTOL v1.7
Didymochlaenace	Didymochlaena mesoamericana Li Bing Zhang & H.Sha	atpB	OP595233	FTOL v1.7
Didymochlaenace	Didymochlaena mesoamericana Li Bing Zhang & H.Sha	rbcL	OP595171	FTOL v1.7
Didymochlaenace	Didymochlaena mesoamericana Li Bing Zhang & H.Sha	rps4	OP595298	FTOL v1.7
Didymochlaenace	Didymochlaena mesoamericana Li Bing Zhang & H.Sha	rps4-trnS	OP595298	FTOL v1.7
Didymochlaenace	Didymochlaena mesoamericana Li Bing Zhang & H.Sha	trnL-trnF	OP595326	FTOL v1.7
Didymochlaenace	Didymochlaena microphylla (Bonap.) C.Chr.	atpA	OP595281	FTOL v1.7
Didymochlaenace	Didymochlaena microphylla (Bonap.) C.Chr.	atpB	OP595251	FTOL v1.7
Didymochlaenace	Didymochlaena microphylla (Bonap.) C.Chr.	rbcL	OP595189	FTOL v1.7
Didymochlaenace	Didymochlaena microphylla (Bonap.) C.Chr.	rps4	OP595316	FTOL v1.7
Didymochlaenace	Didymochlaena microphylla (Bonap.) C.Chr.	rps4-trnS	OP595316	FTOL v1.7
Didymochlaenace	Didymochlaena microphylla (Bonap.) C.Chr.	trnL-trnF	OP595344	FTOL v1.7
Didymochlaenace	Didymochlaena oceanica (Rosenstock) Li Bing Zhang &	atpA	MW323338	FTOL v1.7
Didymochlaenace	Didymochlaena oceanica (Rosenstock) Li Bing Zhang &	atpB	MW323325	FTOL v1.7
Didymochlaenace	Didymochlaena oceanica (Rosenstock) Li Bing Zhang &	rbcL	MW323313	FTOL v1.7
Didymochlaenace	Didymochlaena oceanica (Rosenstock) Li Bing Zhang &	rps4	MW323363	FTOL v1.7
Didymochlaenace	Didymochlaena oceanica (Rosenstock) Li Bing Zhang &	rps4-trnS	OP595295	FTOL v1.7
Didymochlaenace	Didymochlaena oceanica (Rosenstock) Li Bing Zhang &	trnL-trnF	MW323374	FTOL v1.7
Didymochlaenace	Didymochlaena philippensis Li Bing Zhang & H. Shang	atpA	MW323342	FTOL v1.7
Didymochlaenace	Didymochlaena philippensis Li Bing Zhang & H. Shang	atpB	MW323330	FTOL v1.7
Didymochlaenace	Didymochlaena philippensis Li Bing Zhang & H. Shang	rbcL	MW323318	FTOL v1.7
Didymochlaenace	Didymochlaena philippensis Li Bing Zhang & H. Shang	rps4	MW323366	FTOL v1.7
Didymochlaenace	Didymochlaena philippensis Li Bing Zhang & H. Shang	rps4-trnS	MW323366	FTOL v1.7
Didymochlaenace	Didymochlaena philippensis Li Bing Zhang & H. Shang	trnL-trnF	MW323377	FTOL v1.7
Didymochlaenace	Didymochlaena punctata Li Bing Zhang & H. Shang	atpA	OP595266	FTOL v1.7
Didymochlaenace	Didymochlaena punctata Li Bing Zhang & H. Shang	atpB	OP595236	FTOL v1.7
Didymochlaenace	Didymochlaena punctata Li Bing Zhang & H. Shang	rbcL	OP595174	FTOL v1.7
Didymochlaenace	Didymochlaena punctata Li Bing Zhang & H. Shang	rps4	OP595301	FTOL v1.7
Didymochlaenace	Didymochlaena punctata Li Bing Zhang & H. Shang	rps4-trnS	OP595301	FTOL v1.7
Didymochlaenace	Didymochlaena punctata Li Bing Zhang & H. Shang	trnL-trnF	OP595329	FTOL v1.7
Didymochlaenace	Didymochlaena sinuosa Desv.	atpA	OP595270	FTOL v1.7
Didymochlaenace	Didymochlaena sinuosa Desv.	atpB	OP595240	FTOL v1.7
Didymochlaenace	Didymochlaena sinuosa Desv.	rbcL	OP595178	FTOL v1.7
Didymochlaenace	Didymochlaena sinuosa Desv.	rps4	OP595305	FTOL v1.7
Didymochlaenace	Didymochlaena sinuosa Desv.	rps4-trnS	OP595305	FTOL v1.7
Didymochlaenace	Didymochlaena sinuosa Desv.	trnL-trnF	OP595333	FTOL v1.7
Didymochlaenace	Didymochlaena solomonensis Li Bing Zhang & H. Shan	atpA	OP595291	FTOL v1.7
Didymochlaenace	Didymochlaena solomonensis Li Bing Zhang & H. Shan	atpB	OP595259	FTOL v1.7
Didymochlaenace	Didymochlaena solomonensis Li Bing Zhang & H. Shan	rbcL	OP595200	FTOL v1.7
Didymochlaenace	Didymochlaena solomonensis Li Bing Zhang & H. Shan	rps4	OP595292	FTOL v1.7
Didymochlaenace	Didymochlaena solomonensis Li Bing Zhang & H. Shan	rps4-trnS	OP595292	FTOL v1.7
Didymochlaenace	Didymochlaena solomonensis Li Bing Zhang & H. Shan	trnL-trnF	OP595353	FTOL v1.7
Didymochlaenace	Didymochlaena spinulosa (Brause) Li Bing Zhang & H. S	atpA	OP595276	FTOL v1.7
Didymochlaenace	Didymochlaena spinulosa (Brause) Li Bing Zhang & H. S	atpB	OP595246	FTOL v1.7
Didymochlaenace	Didymochlaena spinulosa (Brause) Li Bing Zhang & H. S	rbcL	OP595184	FTOL v1.7
Didymochlaenace	Didymochlaena spinulosa (Brause) Li Bing Zhang & H. S	rps4	OP595311	FTOL v1.7
Didymochlaenace	Didymochlaena spinulosa (Brause) Li Bing Zhang & H. S	rps4-trnS	OP595311	FTOL v1.7
Didymochlaenace	Didymochlaena spinulosa (Brause) Li Bing Zhang & H. S	trnL-trnF	OP595339	FTOL v1.7
Didymochlaenace	Didymochlaena truncatula (Sw.) J. Sm.	plastome	MT130600	FTOL v1.7
Dryopteridaceae	Arachniodes amabilis (Blume) Tindale	rbcL	MK013496	FTOL v1.7
Dryopteridaceae	Arachniodes amabilis (Blume) Tindale	rps4	MK031906	FTOL v1.7
Dryopteridaceae	Arachniodes amabilis (Blume) Tindale	rps4-trnS	MK031906	FTOL v1.7
Dryopteridaceae	Arachniodes amabilis (Blume) Tindale	trnL-trnF	MK013693	FTOL v1.7
Dryopteridaceae	Arachniodes amoena (Ching) Ching	atpB	EF450481	FTOL v1.7
Dryopteridaceae	Arachniodes amoena (Ching) Ching	rbcL	EF463109	FTOL v1.7
Dryopteridaceae	Arachniodes aristata (G. Forst.) Tindale	rbcL	MK013356	FTOL v1.7
Dryopteridaceae	Arachniodes aristata (G. Forst.) Tindale	rps4	MK031741	FTOL v1.7
Dryopteridaceae	Arachniodes aristata (G. Forst.) Tindale	rps4-trnS	MK031741	FTOL v1.7
Dryopteridaceae	Arachniodes aristata (G. Forst.) Tindale	trnL-trnF	MK013520	FTOL v1.7
Dryopteridaceae	Arachniodes assamica (Kuhn) Ohwi	atpB	EF450502	FTOL v1.7
Dryopteridaceae	Arachniodes assamica (Kuhn) Ohwi	rbcL	EF463137	FTOL v1.7
Dryopteridaceae	Arachniodes assamica (Kuhn) Ohwi	rps4	EF540706	FTOL v1.7
Dryopteridaceae	Arachniodes assamica (Kuhn) Ohwi	rps4-trnS	EF540706	FTOL v1.7
Dryopteridaceae	Arachniodes assamica (Kuhn) Ohwi	trnL-trnF	EF540701	FTOL v1.7
Dryopteridaceae	Arachniodes blinii (H. Lév.) Nakaike	atpB	EF450503	FTOL v1.7
Dryopteridaceae	Arachniodes blinii (H. Lév.) Nakaike	rbcL	EF463138	FTOL v1.7
Dryopteridaceae	Arachniodes blinii (H. Lév.) Nakaike	rps4	EF540705	FTOL v1.7
Dryopteridaceae	Arachniodes blinii (H. Lév.) Nakaike	rps4-trnS	EF540705	FTOL v1.7
Dryopteridaceae	Arachniodes blinii (H. Lév.) Nakaike	trnL-trnF	EF540702	FTOL v1.7
Dryopteridaceae	Arachniodes carvifolia (Kunze) Ching	rbcL	MK013394	FTOL v1.7
Dryopteridaceae	Arachniodes carvifolia (Kunze) Ching	rps4	MK031782	FTOL v1.7
Dryopteridaceae	Arachniodes carvifolia (Kunze) Ching	rps4-trnS	MK031782	FTOL v1.7
Dryopteridaceae	Arachniodes carvifolia (Kunze) Ching	trnL-trnF	MK013570	FTOL v1.7
Dryopteridaceae	Arachniodes cavaleriei (Christ) Ohwi	rbcL	MK013377	FTOL v1.7
Dryopteridaceae	Arachniodes cavaleriei (Christ) Ohwi	rps4	MK031766	FTOL v1.7
Dryopteridaceae	Arachniodes cavaleriei (Christ) Ohwi	rps4-trnS	MK031766	FTOL v1.7
Dryopteridaceae	Arachniodes cavaleriei (Christ) Ohwi	trnL-trnF	MK013542	FTOL v1.7
Dryopteridaceae	Arachniodes chinensis (Rosenst.) Ching	rbcL	MK013373	FTOL v1.7
Dryopteridaceae	Arachniodes chinensis (Rosenst.) Ching	rps4	MK031762	FTOL v1.7
Dryopteridaceae	Arachniodes chinensis (Rosenst.) Ching	rps4-trnS	MK031762	FTOL v1.7
Dryopteridaceae	Arachniodes chinensis (Rosenst.) Ching	trnL-trnF	MK013546	FTOL v1.7
Dryopteridaceae	Arachniodes coniifolia (T. Moore) Ching	plastome	MT130551	FTOL v1.7
Dryopteridaceae	Arachniodes cornucervi (D. Don) Fraser-Jenk.	rbcL	MK013480	FTOL v1.7
Dryopteridaceae	Arachniodes cornucervi (D. Don) Fraser-Jenk.	rps4	MK031891	FTOL v1.7
Dryopteridaceae	Arachniodes cornucervi (D. Don) Fraser-Jenk.	rps4-trnS	MK031891	FTOL v1.7
Dryopteridaceae	Arachniodes cornucervi (D. Don) Fraser-Jenk.	trnL-trnF	MK013676	FTOL v1.7
Dryopteridaceae	Arachniodes davalliaeformis (Christ) Nakaike	rbcL	MK013387	FTOL v1.7
Dryopteridaceae	Arachniodes davalliaeformis (Christ) Nakaike	rps4	MK031775	FTOL v1.7
Dryopteridaceae	Arachniodes davalliaeformis (Christ) Nakaike	rps4-trnS	MK031775	FTOL v1.7
Dryopteridaceae	Arachniodes davalliaeformis (Christ) Nakaike	trnL-trnF	MK013559	FTOL v1.7
Dryopteridaceae	Arachniodes denticulata (Sw.) Ching	atpA	EF463664	FTOL v1.7
Dryopteridaceae	Arachniodes denticulata (Sw.) Ching	atpB	EF463380	FTOL v1.7
Dryopteridaceae	Arachniodes denticulata (Sw.) Ching	rbcL	MW620380	FTOL v1.7
Dryopteridaceae	Arachniodes denticulata (Sw.) Ching	rps4	MK031779	FTOL v1.7
Dryopteridaceae	Arachniodes denticulata (Sw.) Ching	rps4-trnS	MK031781	FTOL v1.7
Dryopteridaceae	Arachniodes denticulata (Sw.) Ching	trnL-trnF	MK013568	FTOL v1.7
Dryopteridaceae	Arachniodes fengii Ching	rbcL	MK013397	FTOL v1.7
Dryopteridaceae	Arachniodes fengii Ching	rps4	MK031785	FTOL v1.7
Dryopteridaceae	Arachniodes fengii Ching	rps4-trnS	MK031785	FTOL v1.7
Dryopteridaceae	Arachniodes fengii Ching	trnL-trnF	MK013574	FTOL v1.7
Dryopteridaceae	Arachniodes festina (Hance) Ching	rbcL	MK013400	FTOL v1.7
Dryopteridaceae	Arachniodes festina (Hance) Ching	rps4	MK031788	FTOL v1.7
Dryopteridaceae	Arachniodes festina (Hance) Ching	rps4-trnS	MK031788	FTOL v1.7
Dryopteridaceae	Arachniodes festina (Hance) Ching	trnL-trnF	MK013577	FTOL v1.7
Dryopteridaceae	Arachniodes formosissima (Goldm.) Nakaike	rbcL	MK013429	FTOL v1.7
Dryopteridaceae	Arachniodes formosissima (Goldm.) Nakaike	rps4	MK031821	FTOL v1.7
Dryopteridaceae	Arachniodes formosissima (Goldm.) Nakaike	rps4-trnS	MK031821	FTOL v1.7
Dryopteridaceae	Arachniodes formosissima (Goldm.) Nakaike	trnL-trnF	MK013607	FTOL v1.7
Dryopteridaceae	Arachniodes gigantea Ching	rbcL	DQ508761	FTOL v1.7
Dryopteridaceae	Arachniodes gigantea Ching	trnL-trnF	DQ514483	FTOL v1.7
Dryopteridaceae	Arachniodes globisora (Hayata) Ching	rbcL	MK013408	FTOL v1.7
Dryopteridaceae	Arachniodes globisora (Hayata) Ching	rps4	MK031796	FTOL v1.7
Dryopteridaceae	Arachniodes globisora (Hayata) Ching	rps4-trnS	MK031796	FTOL v1.7
Dryopteridaceae	Arachniodes globisora (Hayata) Ching	trnL-trnF	MK013585	FTOL v1.7
Dryopteridaceae	Arachniodes grossa (Tardieu & C. Chr.) Ching	rbcL	MK013415	FTOL v1.7
Dryopteridaceae	Arachniodes grossa (Tardieu & C. Chr.) Ching	rps4	MK031801	FTOL v1.7
Dryopteridaceae	Arachniodes grossa (Tardieu & C. Chr.) Ching	rps4-trnS	MK031802	FTOL v1.7
Dryopteridaceae	Arachniodes grossa (Tardieu & C. Chr.) Ching	trnL-trnF	MK013592	FTOL v1.7
Dryopteridaceae	Arachniodes hainanensis (Ching) Ching	plastome	MT130572	FTOL v1.7
Dryopteridaceae	Arachniodes hekiana Kurata	rbcL	MK013375	FTOL v1.7
Dryopteridaceae	Arachniodes hekiana Kurata	rps4	MK031764	FTOL v1.7
Dryopteridaceae	Arachniodes hekiana Kurata	rps4-trnS	MK031764	FTOL v1.7
Dryopteridaceae	Arachniodes hekiana Kurata	trnL-trnF	MK013540	FTOL v1.7
Dryopteridaceae	Arachniodes henryi (Christ) Ching	rbcL	MK013421	FTOL v1.7
Dryopteridaceae	Arachniodes henryi (Christ) Ching	rps4	MK031806	FTOL v1.7
Dryopteridaceae	Arachniodes henryi (Christ) Ching	rps4-trnS	MK031806	FTOL v1.7
Dryopteridaceae	Arachniodes henryi (Christ) Ching	trnL-trnF	MK013598	FTOL v1.7
Dryopteridaceae	Arachniodes hiugana Sa. Kurata	rbcL	AB575077	FTOL v1.7
Dryopteridaceae	Arachniodes hiugana Sa. Kurata	rps4-trnS	MK031815	FTOL v1.7
Dryopteridaceae	Arachniodes hiugana Sa. Kurata	trnL-trnF	MK013599	FTOL v1.7
Dryopteridaceae	Arachniodes hunanensis Ching	rbcL	MK013422	FTOL v1.7
Dryopteridaceae	Arachniodes insularis W. H. Wagner	rbcL	MT657468	FTOL v1.7
Dryopteridaceae	Arachniodes jinpingensis Y. T. Hsieh	rbcL	MK013361	FTOL v1.7
Dryopteridaceae	Arachniodes jinpingensis Y. T. Hsieh	rps4	MK031750	FTOL v1.7
Dryopteridaceae	Arachniodes jinpingensis Y. T. Hsieh	rps4-trnS	MK031750	FTOL v1.7
Dryopteridaceae	Arachniodes jinpingensis Y. T. Hsieh	trnL-trnF	MK013528	FTOL v1.7
Dryopteridaceae	Arachniodes longipinna Ching	trnL-trnF	MK013610	FTOL v1.7
Dryopteridaceae	Arachniodes miqueliana (Maxim. ex Franch. & Sav.) Ol	atpB	EF450506	FTOL v1.7
Dryopteridaceae	Arachniodes miqueliana (Maxim. ex Franch. & Sav.) Ol	rbcL	EF463132	FTOL v1.7
Dryopteridaceae	Arachniodes miqueliana (Maxim. ex Franch. & Sav.) Ol	rps4	EF540708	FTOL v1.7
Dryopteridaceae	Arachniodes miqueliana (Maxim. ex Franch. & Sav.) Ol	rps4-trnS	EF540708	FTOL v1.7
Dryopteridaceae	Arachniodes mutica (Franch. & Sav.) Ohwi	rbcL	AB575079	FTOL v1.7
Dryopteridaceae	Arachniodes mutica (Franch. & Sav.) Ohwi	trnL-trnF	MK013699	FTOL v1.7
Dryopteridaceae	Arachniodes neopodophylla (Ching) Nakaike	rbcL	MK013342	FTOL v1.7
Dryopteridaceae	Arachniodes neopodophylla (Ching) Nakaike	rps4	MK031730	FTOL v1.7
Dryopteridaceae	Arachniodes neopodophylla (Ching) Nakaike	rps4-trnS	MK031730	FTOL v1.7
Dryopteridaceae	Arachniodes neopodophylla (Ching) Nakaike	trnL-trnF	MK013504	FTOL v1.7
Dryopteridaceae	Arachniodes nigrospinosa (Ching) Ching	rbcL	MK013449	FTOL v1.7
Dryopteridaceae	Arachniodes nigrospinosa (Ching) Ching	rps4	MK031844	FTOL v1.7
Dryopteridaceae	Arachniodes nigrospinosa (Ching) Ching	rps4-trnS	MK031844	FTOL v1.7
Dryopteridaceae	Arachniodes nigrospinosa (Ching) Ching	trnL-trnF	MK013633	FTOL v1.7
Dryopteridaceae	Arachniodes nipponica (Rosenst.) Ohwi	rbcL	AB575080	FTOL v1.7
Dryopteridaceae	Arachniodes nipponica (Rosenst.) Ohwi	rps4-trnS	MK031848	FTOL v1.7
Dryopteridaceae	Arachniodes nipponica (Rosenst.) Ohwi	trnL-trnF	MK013635	FTOL v1.7
Dryopteridaceae	Arachniodes oohorae H.Ito	rbcL	AB575081	FTOL v1.7
Dryopteridaceae	Arachniodes oohorae H.Ito	rps4-trnS	MK031916	FTOL v1.7
Dryopteridaceae	Arachniodes oohorae H.Ito	trnL-trnF	MK013672	FTOL v1.7
Dryopteridaceae	Arachniodes pseudoaristata (Tagawa) Ohwi	rbcL	MK013452	FTOL v1.7
Dryopteridaceae	Arachniodes pseudoaristata (Tagawa) Ohwi	rps4	MK031854	FTOL v1.7
Dryopteridaceae	Arachniodes pseudoaristata (Tagawa) Ohwi	rps4-trnS	MK031854	FTOL v1.7
Dryopteridaceae	Arachniodes pseudoaristata (Tagawa) Ohwi	trnL-trnF	MK013640	FTOL v1.7
Dryopteridaceae	Arachniodes pseudoassamica Ching	rbcL	MK013455	FTOL v1.7
Dryopteridaceae	Arachniodes pseudoassamica Ching	rps4	MK031857	FTOL v1.7
Dryopteridaceae	Arachniodes pseudoassamica Ching	rps4-trnS	MK031857	FTOL v1.7
Dryopteridaceae	Arachniodes pseudoassamica Ching	trnL-trnF	MK013643	FTOL v1.7
Dryopteridaceae	Arachniodes quadripinnata (Hayata) Seriz.	rbcL	MK013458	FTOL v1.7
Dryopteridaceae	Arachniodes quadripinnata (Hayata) Seriz.	rps4	MK031859	FTOL v1.7
Dryopteridaceae	Arachniodes quadripinnata (Hayata) Seriz.	rps4-trnS	MK031859	FTOL v1.7
Dryopteridaceae	Arachniodes quadripinnata (Hayata) Seriz.	trnL-trnF	MK013646	FTOL v1.7
Dryopteridaceae	Arachniodes rhomboidea (Schott) Ching	atpB	EF450487	FTOL v1.7
Dryopteridaceae	Arachniodes rhomboidea (Schott) Ching	rbcL	EF463115	FTOL v1.7
Dryopteridaceae	Arachniodes simplicior (Makino) Ohwi	rbcL	MK013465	FTOL v1.7
Dryopteridaceae	Arachniodes simplicior (Makino) Ohwi	rps4	MK031873	FTOL v1.7
Dryopteridaceae	Arachniodes simplicior (Makino) Ohwi	rps4-trnS	MK031873	FTOL v1.7
Dryopteridaceae	Arachniodes simplicior (Makino) Ohwi	trnL-trnF	MK013657	FTOL v1.7
Dryopteridaceae	Arachniodes simulans (Ching) Ching	rbcL	MK013388	FTOL v1.7
Dryopteridaceae	Arachniodes simulans (Ching) Ching	rps4	MK032311	FTOL v1.7
Dryopteridaceae	Arachniodes simulans (Ching) Ching	rps4-trnS	MK032311	FTOL v1.7
Dryopteridaceae	Arachniodes simulans (Ching) Ching	trnL-trnF	MK013562	FTOL v1.7
Dryopteridaceae	Arachniodes sinomiqueliana (Ching) Ohwi	rbcL	AB575084	FTOL v1.7
Dryopteridaceae	Arachniodes sinorhomboidea Ching	rbcL	MK013423	FTOL v1.7
Dryopteridaceae	Arachniodes sinorhomboidea Ching	rps4	MK031816	FTOL v1.7
Dryopteridaceae	Arachniodes sinorhomboidea Ching	rps4-trnS	MK031816	FTOL v1.7
Dryopteridaceae	Arachniodes sinorhomboidea Ching	trnL-trnF	MK013600	FTOL v1.7

Dryopteridaceae	Arachniodes spectabilis (Ching) Ching	rbcL	MK013473	FTOL v1.7
Dryopteridaceae	Arachniodes spectabilis (Ching) Ching	rps4	MK031884	FTOL v1.7
Dryopteridaceae	Arachniodes spectabilis (Ching) Ching	rps4-trnS	MK031884	FTOL v1.7
Dryopteridaceae	Arachniodes spectabilis (Ching) Ching	trnL-trnF	MK013666	FTOL v1.7
Dryopteridaceae	Arachniodes standishii (T. Moore) Ohwi	atpB	EF450508	FTOL v1.7
Dryopteridaceae	Arachniodes standishii (T. Moore) Ohwi	rbcL	EF540722	FTOL v1.7
Dryopteridaceae	Arachniodes standishii (T. Moore) Ohwi	rps4	EF540709	FTOL v1.7
Dryopteridaceae	Arachniodes standishii (T. Moore) Ohwi	rps4-trnS	EF540709	FTOL v1.7
Dryopteridaceae	Arachniodes standishii (T. Moore) Ohwi	trnL-trnF	EF540700	FTOL v1.7
Dryopteridaceae	Arachniodes superba Fraser-Jenk.	plastome	MT130657	FTOL v1.7
Dryopteridaceae	Arachniodes tonkinensis (Ching) Ching	rbcL	MK013479	FTOL v1.7
Dryopteridaceae	Arachniodes tonkinensis (Ching) Ching	rps4	MK031890	FTOL v1.7
Dryopteridaceae	Arachniodes tonkinensis (Ching) Ching	rps4-trnS	MK031890	FTOL v1.7
Dryopteridaceae	Arachniodes tonkinensis (Ching) Ching	trnL-trnF	MK013675	FTOL v1.7
Dryopteridaceae	Arachniodes tripinnata (Goldm.) Sledge	rbcL	MK188728	FTOL v1.7
Dryopteridaceae	Arachniodes yasu-inouei var. angustipinnula Seriz.	rbcL	LC484409	FTOL v1.7
Dryopteridaceae	Arachniodes ziyunshanensis Y. T. Xie	rbcL	MK013501	FTOL v1.7
Dryopteridaceae	Arachniodes ziyunshanensis Y. T. Xie	rps4	MK031913	FTOL v1.7
Dryopteridaceae	Arachniodes ziyunshanensis Y. T. Xie	rps4-trnS	MK031913	FTOL v1.7
Dryopteridaceae	Arachniodes ziyunshanensis Y. T. Xie	trnL-trnF	MK013703	FTOL v1.7
Dryopteridaceae	Arthrobotrya articulata (J. Sm. ex Fée) J. Sm.	rps4-trnS	GU376714	FTOL v1.7
Dryopteridaceae	Arthrobotrya articulata (J. Sm. ex Fée) J. Sm.	trnL-trnF	GU376565	FTOL v1.7
Dryopteridaceae	Arthrobotrya brightiae (F. Muell.) Pic. Serm.	atpA	KT831778	FTOL v1.7
Dryopteridaceae	Arthrobotrya brightiae (F. Muell.) Pic. Serm.	atpB	KT831810	FTOL v1.7
Dryopteridaceae	Arthrobotrya brightiae (F. Muell.) Pic. Serm.	rbcL	KT831848	FTOL v1.7
Dryopteridaceae	Arthrobotrya wilkesiana (Brack.) Copel.	atpA	EF463730	FTOL v1.7
Dryopteridaceae	Arthrobotrya wilkesiana (Brack.) Copel.	atpB	EF463446	FTOL v1.7
Dryopteridaceae	Arthrobotrya wilkesiana (Brack.) Copel.	rbcL	AB232402	FTOL v1.7
Dryopteridaceae	Arthrobotrya wilkesiana (Brack.) Copel.	rps4-trnS	GU376719	FTOL v1.7
Dryopteridaceae	Arthrobotrya wilkesiana (Brack.) Copel.	trnL-trnF	GU376569	FTOL v1.7
Dryopteridaceae	Bolbitis × laxireticulata K. Iwats.	plastome	OM350395	FTOL v1.7
Dryopteridaceae	Bolbitis × multipinna F. G. Wang, K. Iwats. & F. W. Xin	plastome	OM891302	FTOL v1.7
Dryopteridaceae	Bolbitis × terminans (Wall.) Gandhi & Fraser-Jenk.	rbcL	MK779194	FTOL v1.7
Dryopteridaceae	Bolbitis acrostichoides (Afzel. ex Sw.) Ching	rbcL	KJ464420	FTOL v1.7
Dryopteridaceae	Bolbitis acrostichoides (Afzel. ex Sw.) Ching	rps4-trnS	GU376645	FTOL v1.7
Dryopteridaceae	Bolbitis acrostichoides (Afzel. ex Sw.) Ching	trnL-trnF	GU376501	FTOL v1.7
Dryopteridaceae	Bolbitis aliena (Sw.) Alston	plastome	OP432086	FTOL v1.7
Dryopteridaceae	Bolbitis angustipinna (Hayata) Itô	plastome	PP471972	FTOL v1.7
Dryopteridaceae	Bolbitis appendiculata (Willd.) K. Iwats.	plastome	MT130676	FTOL v1.7
Dryopteridaceae	Bolbitis auriculata (Lam.) Alston	atpA	EF463665	FTOL v1.7
Dryopteridaceae	Bolbitis auriculata (Lam.) Alston	atpB	EF463381	FTOL v1.7
Dryopteridaceae	Bolbitis auriculata (Lam.) Alston	rbcL	EF463170	FTOL v1.7
Dryopteridaceae	Bolbitis bipinnatifida (Mett. ex Kuhn) Ching	rps4-trnS	GU376676	FTOL v1.7
Dryopteridaceae	Bolbitis bipinnatifida (Mett. ex Kuhn) Ching	trnL-trnF	GU376530	FTOL v1.7
Dryopteridaceae	Bolbitis costata (Wall. ex Hook.) Ching	rbcL	KT225395	FTOL v1.7
Dryopteridaceae	Bolbitis crispatula var. copelandii (Ching ex Tardieu &	rbcL	ON820495	FTOL v1.7
Dryopteridaceae	Bolbitis crispatula var. copelandii (Ching ex Tardieu &	rps4	ON820387	FTOL v1.7
Dryopteridaceae	Bolbitis crispatula var. copelandii (Ching ex Tardieu &	rps4-trnS	ON820387	FTOL v1.7
Dryopteridaceae	Bolbitis crispatula var. copelandii (Ching ex Tardieu &	trnL-trnF	ON820275	FTOL v1.7
Dryopteridaceae	Bolbitis deltigera	plastome	MT130603	FTOL v1.7
Dryopteridaceae	Bolbitis fengiana (Ching) S. Y. Dong	rbcL	ON820498	FTOL v1.7
Dryopteridaceae	Bolbitis fengiana (Ching) S. Y. Dong	rps4	ON820390	FTOL v1.7
Dryopteridaceae	Bolbitis fengiana (Ching) S. Y. Dong	rps4-trnS	ON820389	FTOL v1.7
Dryopteridaceae	Bolbitis fengiana (Ching) S. Y. Dong	trnL-trnF	ON820277	FTOL v1.7
Dryopteridaceae	Bolbitis fluviatilis (Hook.) Ching	rps4-trnS	GU376656	FTOL v1.7
Dryopteridaceae	Bolbitis fluviatilis (Hook.) Ching	trnL-trnF	GU376510	FTOL v1.7
Dryopteridaceae	Bolbitis gemmifera (Hieron.) C. Chr.	rps4-trnS	GU376657	FTOL v1.7
Dryopteridaceae	Bolbitis gemmifera (Hieron.) C. Chr.	trnL-trnF	GU376511	FTOL v1.7
Dryopteridaceae	Bolbitis hainanensis Ching & C. H. Wang	rbcL	ON820500	FTOL v1.7
Dryopteridaceae	Bolbitis hainanensis Ching & C. H. Wang	rps4	ON820393	FTOL v1.7
Dryopteridaceae	Bolbitis hainanensis Ching & C. H. Wang	rps4-trnS	ON820393	FTOL v1.7
Dryopteridaceae	Bolbitis hainanensis Ching & C. H. Wang	trnL-trnF	ON820281	FTOL v1.7
Dryopteridaceae	Bolbitis hastata (Liebm. ex E. Fourn.) Hennipman	rbcL	MW620412	FTOL v1.7
Dryopteridaceae	Bolbitis hekouensis Ching	plastome	OP379655	FTOL v1.7
Dryopteridaceae	Bolbitis heteroclita (C. Presl) Ching	plastome	MT130550	FTOL v1.7
Dryopteridaceae	Bolbitis heudelotii (Bory ex Fée) Alston	rps4-trnS	GU376662	FTOL v1.7
Dryopteridaceae	Bolbitis heudelotii (Bory ex Fée) Alston	trnL-trnF	GU376515	FTOL v1.7
Dryopteridaceae	Bolbitis humblotii (Baker) Ching	rbcL	KJ464422	FTOL v1.7
Dryopteridaceae	Bolbitis lianhuachihensis Y. S. Chao, Y. F. Huang & H. Y	rps4-trnS	MK978747	FTOL v1.7
Dryopteridaceae	Bolbitis lianhuachihensis Y. S. Chao, Y. F. Huang & H. Y	trnL-trnF	MK978746	FTOL v1.7
Dryopteridaceae	Bolbitis lonchophora (Kunze) C. Chr.	rbcL	KY099778	FTOL v1.7
Dryopteridaceae	Bolbitis major (Bedd.) Hennipman	rbcL	ON820478	FTOL v1.7
Dryopteridaceae	Bolbitis major (Bedd.) Hennipman	rps4	ON820367	FTOL v1.7
Dryopteridaceae	Bolbitis major (Bedd.) Hennipman	rps4-trnS	ON820367	FTOL v1.7
Dryopteridaceae	Bolbitis major (Bedd.) Hennipman	trnL-trnF	ON820256	FTOL v1.7
Dryopteridaceae	Bolbitis medogensis (Ching & S. K. Wu) S. Y. Dong	rbcL	ON820508	FTOL v1.7
Dryopteridaceae	Bolbitis medogensis (Ching & S. K. Wu) S. Y. Dong	rps4	ON820403	FTOL v1.7
Dryopteridaceae	Bolbitis medogensis (Ching & S. K. Wu) S. Y. Dong	rps4-trnS	ON820403	FTOL v1.7
Dryopteridaceae	Bolbitis nodiflora (Bory) Fraser-Jenk.	rbcL	ON820526	FTOL v1.7
Dryopteridaceae	Bolbitis nodiflora (Bory) Fraser-Jenk.	rps4	ON820422	FTOL v1.7
Dryopteridaceae	Bolbitis nodiflora (Bory) Fraser-Jenk.	rps4-trnS	ON820422	FTOL v1.7
Dryopteridaceae	Bolbitis nodiflora (Bory) Fraser-Jenk.	trnL-trnF	ON820311	FTOL v1.7
Dryopteridaceae	Bolbitis portoricensis (Spreng.) Hennipman	rbcL	KJ628645	FTOL v1.7
Dryopteridaceae	Bolbitis portoricensis (Spreng.) Hennipman	rps4-trnS	GU376670	FTOL v1.7
Dryopteridaceae	Bolbitis portoricensis (Spreng.) Hennipman	trnL-trnF	GU376524	FTOL v1.7
Dryopteridaceae	Bolbitis quoyana (Gaudich.) Ching	rbcL	MT657679	FTOL v1.7
Dryopteridaceae	Bolbitis repanda (Blume) Schott	rbcL	AB232399	FTOL v1.7
Dryopteridaceae	Bolbitis rhizophylla (Kaulf.) Hennipman	rps4-trnS	MK639323	FTOL v1.7
Dryopteridaceae	Bolbitis rhizophylla (Kaulf.) Hennipman	trnL-trnF	MK978742	FTOL v1.7
Dryopteridaceae	Bolbitis salicina (Hook.) Ching	rps4-trnS	GU376671	FTOL v1.7
Dryopteridaceae	Bolbitis salicina (Hook.) Ching	trnL-trnF	GU376525	FTOL v1.7
Dryopteridaceae	Bolbitis scalpturata (Fée) Ching	rps4-trnS	MK639324	FTOL v1.7
Dryopteridaceae	Bolbitis scalpturata (Fée) Ching	trnL-trnF	MK978741	FTOL v1.7
Dryopteridaceae	Bolbitis semicordata (Baker) Ching	rbcL	MK562517	FTOL v1.7
Dryopteridaceae	Bolbitis semipinnatifida (Fée) Alston	rbcL	KJ628646	FTOL v1.7
Dryopteridaceae	Bolbitis serratifolia (Mert. ex Kaulf.) Schott	atpB	MG600314	FTOL v1.7
Dryopteridaceae	Bolbitis serratifolia (Mert. ex Kaulf.) Schott	rps4-trnS	GU376674	FTOL v1.7
Dryopteridaceae	Bolbitis serratifolia (Mert. ex Kaulf.) Schott	trnL-trnF	GU376528	FTOL v1.7
Dryopteridaceae	Bolbitis sinensis (Baker) K. Iwats.	plastome	OP359065	FTOL v1.7
Dryopteridaceae	Bolbitis sinuata (C. Presl) Hennipman	rps4-trnS	GU376675	FTOL v1.7
Dryopteridaceae	Bolbitis sinuata (C. Presl) Hennipman	trnL-trnF	GU376529	FTOL v1.7
Dryopteridaceae	Bolbitis subcordata (Copel.) Ching	plastome	MT130686	FTOL v1.7
Dryopteridaceae	Bolbitis tibetica Ching & S. K. Wu	rbcL	ON820481	FTOL v1.7
Dryopteridaceae	Bolbitis tibetica Ching & S. K. Wu	rps4	ON820372	FTOL v1.7
Dryopteridaceae	Bolbitis tibetica Ching & S. K. Wu	rps4-trnS	ON820372	FTOL v1.7
Dryopteridaceae	Bolbitis tibetica Ching & S. K. Wu	trnL-trnF	ON820259	FTOL v1.7
Dryopteridaceae	Bolbitis tonkinensis (C. Chr. ex Ching) K. Iwats.	plastome	OP432084	FTOL v1.7
Dryopteridaceae	Bolbitis virens var. compacta Hennipman	rbcL	ON820477	FTOL v1.7
Dryopteridaceae	Bolbitis virens var. compacta Hennipman	rps4	ON820365	FTOL v1.7
Dryopteridaceae	Bolbitis virens var. compacta Hennipman	rps4-trnS	ON820365	FTOL v1.7
Dryopteridaceae	Bolbitis virens var. compacta Hennipman	trnL-trnF	ON820254	FTOL v1.7
Dryopteridaceae	Ctenitis aciculata (Baker) Ching	rbcL	KY701622	FTOL v1.7
Dryopteridaceae	Ctenitis aciculata (Baker) Ching	trnL-trnF	KY701668	FTOL v1.7
Dryopteridaceae	Ctenitis analamazaotrensis (Rakotondr.) Hennequin &	rbcL	KY701662	FTOL v1.7
Dryopteridaceae	Ctenitis analamazaotrensis (Rakotondr.) Hennequin &	trnL-trnF	KY701720	FTOL v1.7
Dryopteridaceae	Ctenitis anniesii (Rosenst.) Copel.	trnL-trnF	ON186672	FTOL v1.7
Dryopteridaceae	Ctenitis aspidioides (C. Presl) Copel.	rbcL	OM810244	FTOL v1.7
Dryopteridaceae	Ctenitis aspidioides (C. Presl) Copel.	trnL-trnF	ON186673	FTOL v1.7
Dryopteridaceae	Ctenitis biformis (Mett. ex Kuhn) Tardieu	atpA	KF898003	FTOL v1.7
Dryopteridaceae	Ctenitis biformis (Mett. ex Kuhn) Tardieu	rbcL	KF887166	FTOL v1.7
Dryopteridaceae	Ctenitis biformis (Mett. ex Kuhn) Tardieu	trnL-trnF	KF897951	FTOL v1.7
Dryopteridaceae	Ctenitis bivestita (Mett.) Tardieu	trnL-trnF	KX238782	FTOL v1.7
Dryopteridaceae	Ctenitis borbonica (Baker) Tardieu	rbcL	KY701623	FTOL v1.7
Dryopteridaceae	Ctenitis borbonica (Baker) Tardieu	trnL-trnF	KY701687	FTOL v1.7
Dryopteridaceae	Ctenitis brevipetiolulata Li Bing Zhang & Yi F. Duan	rbcL	KY701653	FTOL v1.7
Dryopteridaceae	Ctenitis brevipetiolulata Li Bing Zhang & Yi F. Duan	trnL-trnF	KY701713	FTOL v1.7
Dryopteridaceae	Ctenitis bullescens Rouhan, Li Bing Zhang & Yi F. Duan	rbcL	KY701647	FTOL v1.7
Dryopteridaceae	Ctenitis bullescens Rouhan, Li Bing Zhang & Yi F. Duan	trnL-trnF	KY701708	FTOL v1.7
Dryopteridaceae	Ctenitis cerea Li Bing Zhang & Yi F. Duan	trnL-trnF	KY701689	FTOL v1.7
Dryopteridaceae	Ctenitis christensenii R. S. Viveros & Salino	rbcL	OM810247	FTOL v1.7
Dryopteridaceae	Ctenitis christensenii R. S. Viveros & Salino	trnL-trnF	ON186676	FTOL v1.7
Dryopteridaceae	Ctenitis cirrhosa (Schum.) Ching	rbcL	KY701626	FTOL v1.7
Dryopteridaceae	Ctenitis cirrhosa (Schum.) Ching	trnL-trnF	KY701670	FTOL v1.7
Dryopteridaceae	Ctenitis comorensis Li Bing Zhang & Yi F. Duan	rbcL	KY701624	FTOL v1.7
Dryopteridaceae	Ctenitis comorensis Li Bing Zhang & Yi F. Duan	trnL-trnF	KY701688	FTOL v1.7
Dryopteridaceae	Ctenitis crinigera (C. Chr.) Hennequin & Rouhan	rbcL	KY701664	FTOL v1.7
Dryopteridaceae	Ctenitis crinigera (C. Chr.) Hennequin & Rouhan	trnL-trnF	KY701722	FTOL v1.7
Dryopteridaceae	Ctenitis crinita (Poir.) Ching	rbcL	KF992457	FTOL v1.7
Dryopteridaceae	Ctenitis crinita (Poir.) Ching	trnL-trnF	KY701695	FTOL v1.7
Dryopteridaceae	Ctenitis cyclochlamys (Fée) Holttum	rbcL	KY701633	FTOL v1.7
Dryopteridaceae	Ctenitis cyclochlamys (Fée) Holttum	trnL-trnF	KX238781	FTOL v1.7
Dryopteridaceae	Ctenitis decaryana (C. Chr.) Hennequin & Rouhan	rbcL	KY701665	FTOL v1.7
Dryopteridaceae	Ctenitis decaryana (C. Chr.) Hennequin & Rouhan	trnL-trnF	KY701723	FTOL v1.7
Dryopteridaceae	Ctenitis decurrentipinnata (Ching) Tardieu & C. Chr.	plastome	MT130665	FTOL v1.7
Dryopteridaceae	Ctenitis deflexa (Kaulf.) Copel.	trnL-trnF	ON502957	FTOL v1.7
Dryopteridaceae	Ctenitis dentata Li Bing Zhang & Yi F. Duan	rbcL	KY701627	FTOL v1.7
Dryopteridaceae	Ctenitis dentata Li Bing Zhang & Yi F. Duan	trnL-trnF	KY701692	FTOL v1.7
Dryopteridaceae	Ctenitis desvauxii (Mett. ex Kuhn) Tardieu	rbcL	KF992459	FTOL v1.7
Dryopteridaceae	Ctenitis desvauxii (Mett. ex Kuhn) Tardieu	trnL-trnF	KY701700	FTOL v1.7
Dryopteridaceae	Ctenitis distans (Brack.) Ching	rbcL	OM810248	FTOL v1.7
Dryopteridaceae	Ctenitis distans (Brack.) Ching	trnL-trnF	ON186678	FTOL v1.7
Dryopteridaceae	Ctenitis eatonii (Baker) Ching	atpA	KF709456	FTOL v1.7
Dryopteridaceae	Ctenitis eatonii (Baker) Ching	atpB	KF709464	FTOL v1.7
Dryopteridaceae	Ctenitis eatonii (Baker) Ching	rbcL	KF709483	FTOL v1.7
Dryopteridaceae	Ctenitis eatonii (Baker) Ching	trnL-trnF	KF709509	FTOL v1.7
Dryopteridaceae	Ctenitis equestris (Kunze) Ching	rbcL	KY701637	FTOL v1.7
Dryopteridaceae	Ctenitis equestris (Kunze) Ching	trnL-trnF	KY701672	FTOL v1.7
Dryopteridaceae	Ctenitis eriocaulis (Fée) Alston	rbcL	KY701638	FTOL v1.7
Dryopteridaceae	Ctenitis eriocaulis (Fée) Alston	trnL-trnF	KY701701	FTOL v1.7
Dryopteridaceae	Ctenitis exaggerata (Baker) Ching	rbcL	KY701639	FTOL v1.7
Dryopteridaceae	Ctenitis exaggerata (Baker) Ching	trnL-trnF	KY701702	FTOL v1.7
Dryopteridaceae	Ctenitis excelsa (Desv.) Proctor	rbcL	KY701641	FTOL v1.7
Dryopteridaceae	Ctenitis excelsa (Desv.) Proctor	trnL-trnF	KY701674	FTOL v1.7
Dryopteridaceae	Ctenitis fibrillosa (Baker) Li Bing Zhang & Yi F. Duan	rbcL	KF887168	FTOL v1.7
Dryopteridaceae	Ctenitis glandulosa R. S. Viveros & Salino	rbcL	KY701642	FTOL v1.7
Dryopteridaceae	Ctenitis glandulosa R. S. Viveros & Salino	trnL-trnF	KY701703	FTOL v1.7
Dryopteridaceae	Ctenitis hemsleyana (Baker ex Hemsl.) Copel.	rbcL	MW620381	FTOL v1.7
Dryopteridaceae	Ctenitis hemsleyana (Baker ex Hemsl.) Copel.	trnL-trnF	KY701704	FTOL v1.7
Dryopteridaceae	Ctenitis hispida (Kuhn) Li Bing Zhang & Yi F. Duan	rbcL	KY701631	FTOL v1.7
Dryopteridaceae	Ctenitis hispida (Kuhn) Li Bing Zhang & Yi F. Duan	trnL-trnF	KY701696	FTOL v1.7
Dryopteridaceae	Ctenitis humida (Cordem.) Holttum	rbcL	KY701645	FTOL v1.7
Dryopteridaceae	Ctenitis humida (Cordem.) Holttum	trnL-trnF	KY701706	FTOL v1.7
Dryopteridaceae	Ctenitis iriomotensis (H. Itô) Nakaike	rbcL	AB575094	FTOL v1.7
Dryopteridaceae	Ctenitis laetevirens (Rosenst.) Salino & P. O. Morais	rbcL	OM810250	FTOL v1.7
Dryopteridaceae	Ctenitis laetevirens (Rosenst.) Salino & P. O. Morais	trnL-trnF	ON186680	FTOL v1.7
Dryopteridaceae	Ctenitis lanceolata (Baker) A. R. Sm.	rbcL	KY701646	FTOL v1.7
Dryopteridaceae	Ctenitis lanceolata (Baker) A. R. Sm.	trnL-trnF	KY701707	FTOL v1.7
Dryopteridaceae	Ctenitis latifrons (Brack.) Copel.	rbcL	MT657492	FTOL v1.7
Dryopteridaceae	Ctenitis latifrons (Brack.) Copel.	trnL-trnF	ON186681	FTOL v1.7
Dryopteridaceae	Ctenitis lorencei Holttum	trnL-trnF	KY701675	FTOL v1.7
Dryopteridaceae	Ctenitis maritima (Cordem.) Tardieu	rbcL	KF992461	FTOL v1.7
Dryopteridaceae	Ctenitis maritima (Cordem.) Tardieu	trnL-trnF	KY701710	FTOL v1.7
Dryopteridaceae	Ctenitis mayottensis Li Bing Zhang & Yi F. Duan	trnL-trnF	KY701690	FTOL v1.7
Dryopteridaceae	Ctenitis melanosticta (Kunze) Copel.	rbcL	MW620252	FTOL v1.7
Dryopteridaceae	Ctenitis microlepigera (Nakai) Ching	rbcL	AB575097	FTOL v1.7
Dryopteridaceae	Ctenitis nervata (Fée) R. S. Viveros & Salino	rbcL	OM810252	FTOL v1.7
Dryopteridaceae	Ctenitis nervata (Fée) R. S. Viveros & Salino	trnL-trnF	ON186682	FTOL v1.7

Dryopteridaceae	Ctenitis nigrovenia (Christ) Copel.	rbcL	OM810253	FTOL v1.7
Dryopteridaceae	Ctenitis nigrovenia (Christ) Copel.	trnL-trnF	ON186683	FTOL v1.7
Dryopteridaceae	Ctenitis pallens (Brack.) M. G. Price	trnL-trnF	KY701679	FTOL v1.7
Dryopteridaceae	Ctenitis pauciflora (Kaulf. ex Spreng.) Holttum	rbcL	KY701651	FTOL v1.7
Dryopteridaceae	Ctenitis pauciflora (Kaulf. ex Spreng.) Holttum	trnL-trnF	KY701680	FTOL v1.7
Dryopteridaceae	Ctenitis refulgens (Klotzsch ex Mett.) Vareschi	atpB	KM114092	FTOL v1.7
Dryopteridaceae	Ctenitis refulgens (Klotzsch ex Mett.) Vareschi	rbcL	KJ628670	FTOL v1.7
Dryopteridaceae	Ctenitis refulgens (Klotzsch ex Mett.) Vareschi	trnL-trnF	KY701714	FTOL v1.7
Dryopteridaceae	Ctenitis reunionensis Hennequin, Li Bing Zhang & Yi F.	trnL-trnF	KY701691	FTOL v1.7
Dryopteridaceae	Ctenitis sciaphila (Maxon) Ching	rbcL	KY099785	FTOL v1.7
Dryopteridaceae	Ctenitis sieberiana (Kaulf. ex Spreng.) Hennequin & Ro	rbcL	KF992495	FTOL v1.7
Dryopteridaceae	Ctenitis sieberiana (Kaulf. ex Spreng.) Hennequin & Ro	trnL-trnF	KY701719	FTOL v1.7
Dryopteridaceae	Ctenitis sinii (Ching) Ohwi	atpB	KJ196464	FTOL v1.7
Dryopteridaceae	Ctenitis sinii (Ching) Ohwi	rbcL	KJ196861	FTOL v1.7
Dryopteridaceae	Ctenitis sinii (Ching) Ohwi	trnL-trnF	KJ196643	FTOL v1.7
Dryopteridaceae	Ctenitis sloanei (Poepp. ex Spreng.) C. V. Morton	atpA	JF832106	FTOL v1.7
Dryopteridaceae	Ctenitis sloanei (Poepp. ex Spreng.) C. V. Morton	atpB	EF463383	FTOL v1.7
Dryopteridaceae	Ctenitis sloanei (Poepp. ex Spreng.) C. V. Morton	rbcL	EF463172	FTOL v1.7
Dryopteridaceae	Ctenitis sloanei (Poepp. ex Spreng.) C. V. Morton	trnL-trnF	KY701715	FTOL v1.7
Dryopteridaceae	Ctenitis spekei (Baker) Li Bing Zhang & Yi F. Duan	trnL-trnF	KY701676	FTOL v1.7
Dryopteridaceae	Ctenitis squamigera (Brack.) Copel.	rbcL	MT657493	FTOL v1.7
Dryopteridaceae	Ctenitis squamigera (Brack.) Copel.	trnL-trnF	ON186685	FTOL v1.7
Dryopteridaceae	Ctenitis subglandulosa (Hance) Ching	atpB	KJ196429	FTOL v1.7
Dryopteridaceae	Ctenitis subglandulosa (Hance) Ching	rbcL	KJ196836	FTOL v1.7
Dryopteridaceae	Ctenitis subglandulosa (Hance) Ching	trnL-trnF	KJ196715	FTOL v1.7
Dryopteridaceae	Ctenitis submarginalis (Langsd. & Fisch.) Ching	atpA	EF463668	FTOL v1.7
Dryopteridaceae	Ctenitis submarginalis (Langsd. & Fisch.) Ching	atpB	EF463384	FTOL v1.7
Dryopteridaceae	Ctenitis submarginalis (Langsd. & Fisch.) Ching	rbcL	EF463173	FTOL v1.7
Dryopteridaceae	Ctenitis tardieu-blotiae Li Bing Zhang & Yi F. Duan	trnL-trnF	KY701671	FTOL v1.7
Dryopteridaceae	Ctenitis vilis (Kunze) Ching	rbcL	KY701659	FTOL v1.7
Dryopteridaceae	Ctenitis vilis (Kunze) Ching	trnL-trnF	KY701683	FTOL v1.7
Dryopteridaceae	Ctenitis warburii (C. Chr.) Tardieu	trnL-trnF	KY701686	FTOL v1.7
Dryopteridaceae	Cyclodium akawaiorum A. R. Sm.	rps4-trnS	MN025363	FTOL v1.7
Dryopteridaceae	Cyclodium calophyllum (C. V. Morton) A. R. Sm.	rbcL	KT272936	FTOL v1.7
Dryopteridaceae	Cyclodium chocoense (A. R. Sm.) Bohn & Labiak	rbcL	MN025355	FTOL v1.7
Dryopteridaceae	Cyclodium chocoense (A. R. Sm.) Bohn & Labiak	rps4-trnS	MN025367	FTOL v1.7
Dryopteridaceae	Cyclodium guianense (Klotzsch) van der Werff ex L. D. (	rbcL	KT272937	FTOL v1.7
Dryopteridaceae	Cyclodium guianense (Klotzsch) van der Werff ex L. D. (	rps4-trnS	KT273010	FTOL v1.7
Dryopteridaceae	Cyclodium guianense (Klotzsch) van der Werff ex L. D. (	trnL-trnF	KT273162	FTOL v1.7
Dryopteridaceae	Cyclodium heterodon (Schrad.) T. Moore	rbcL	KJ464425	FTOL v1.7
Dryopteridaceae	Cyclodium heterodon (Schrad.) T. Moore	rps4-trnS	KJ464666	FTOL v1.7
Dryopteridaceae	Cyclodium heterodon (Schrad.) T. Moore	trnL-trnF	KJ464596	FTOL v1.7
Dryopteridaceae	Cyclodium inerme (Fée) A. R. Sm.	rbcL	MN025357	FTOL v1.7
Dryopteridaceae	Cyclodium inerme (Fée) A. R. Sm.	rps4-trnS	KT273011	FTOL v1.7
Dryopteridaceae	Cyclodium inerme (Fée) A. R. Sm.	trnL-trnF	KT273163	FTOL v1.7
Dryopteridaceae	Cyclodium meniscioides (Willd.) C. Presl	atpB	MN781240	FTOL v1.7
Dryopteridaceae	Cyclodium meniscioides (Willd.) C. Presl	rbcL	MN025359	FTOL v1.7
Dryopteridaceae	Cyclodium meniscioides (Willd.) C. Presl	rps4-trnS	KT273012	FTOL v1.7
Dryopteridaceae	Cyclodium rheophilum A. R. Sm.	rbcL	KJ464426	FTOL v1.7
Dryopteridaceae	Cyclodium rheophilum A. R. Sm.	rps4-trnS	KJ464667	FTOL v1.7
Dryopteridaceae	Cyclodium rheophilum A. R. Sm.	trnL-trnF	KJ464597	FTOL v1.7
Dryopteridaceae	Cyclodium trianae (Mett.) A. R. Sm.	atpA	EF463670	FTOL v1.7
Dryopteridaceae	Cyclodium trianae (Mett.) A. R. Sm.	atpB	EF463386	FTOL v1.7
Dryopteridaceae	Cyclodium trianae (Mett.) A. R. Sm.	rbcL	EF463175	FTOL v1.7
Dryopteridaceae	Cyrtomium aequibasis (C. Chr.) Ching	rbcL	AY694809	FTOL v1.7
Dryopteridaceae	Cyrtomium aequibasis (C. Chr.) Ching	trnL-trnF	AY736346	FTOL v1.7
Dryopteridaceae	Cyrtomium anomophyllum (Zenker) Fraser-Jenk.	rbcL	AB575110	FTOL v1.7
Dryopteridaceae	Cyrtomium atropunctatum Kurata	rbcL	AB575105	FTOL v1.7
Dryopteridaceae	Cyrtomium caryotideum (Wall. ex Hook. & Grev.) C. Pre	plastome	OR051895	FTOL v1.7
Dryopteridaceae	Cyrtomium chingianum P. S. Wang	rbcL	AY694803	FTOL v1.7
Dryopteridaceae	Cyrtomium chingianum P. S. Wang	trnL-trnF	AY736339	FTOL v1.7
Dryopteridaceae	Cyrtomium devexiscapulae (Koidz.) Ching	plastome	KT599100	FTOL v1.7
Dryopteridaceae	Cyrtomium falcatum (L. fil.) C. Presl	plastome	KP189363	FTOL v1.7
Dryopteridaceae	Cyrtomium fortunei J. Sm.	plastome	MG913607	FTOL v1.7
Dryopteridaceae	Cyrtomium grossum Christ	rbcL	AY694805	FTOL v1.7
Dryopteridaceae	Cyrtomium grossum Christ	trnL-trnF	AY736341	FTOL v1.7
Dryopteridaceae	Cyrtomium guizhouense H. S. Kung & P. S. Wang	rbcL	AY694806	FTOL v1.7
Dryopteridaceae	Cyrtomium guizhouense H. S. Kung & P. S. Wang	trnL-trnF	AY736342	FTOL v1.7
Dryopteridaceae	Cyrtomium hemionitis Christ	plastome	MT130568	FTOL v1.7
Dryopteridaceae	Cyrtomium laetevirens (Hiyama) Nakaike	rbcL	AB575108	FTOL v1.7
Dryopteridaceae	Cyrtomium lonchitoides (Christ) Christ	rbcL	AY694800	FTOL v1.7
Dryopteridaceae	Cyrtomium lonchitoides (Christ) Christ	trnL-trnF	AY736336	FTOL v1.7
Dryopteridaceae	Cyrtomium macrophyllum (Makino) Tagawa	plastome	OR051896	FTOL v1.7
Dryopteridaceae	Cyrtomium nephrolepioides (Christ) Copel.	atpB	EF450473	FTOL v1.7
Dryopteridaceae	Cyrtomium nephrolepioides (Christ) Copel.	rbcL	EF394242	FTOL v1.7
Dryopteridaceae	Cyrtomium omeiense China & Shing	rbcL	KU244742	FTOL v1.7
Dryopteridaceae	Cyrtomium omeiense China & Shing	rps4-trnS	KU244914	FTOL v1.7
Dryopteridaceae	Cyrtomium omeiense China & Shing	trnL-trnF	KU245001	FTOL v1.7
Dryopteridaceae	Cyrtomium pachyphyllum (Rosenst.) C. Chr.	atpB	EF450474	FTOL v1.7
Dryopteridaceae	Cyrtomium pachyphyllum (Rosenst.) C. Chr.	rbcL	EF394241	FTOL v1.7
Dryopteridaceae	Cyrtomium shingianum H. S. Kung & P. S. Wang	rbcL	AY694804	FTOL v1.7
Dryopteridaceae	Cyrtomium shingianum H. S. Kung & P. S. Wang	trnL-trnF	AY736340	FTOL v1.7
Dryopteridaceae	Cyrtomium urophyllum Ching	atpB	EF450475	FTOL v1.7
Dryopteridaceae	Cyrtomium urophyllum Ching	rbcL	EF394240	FTOL v1.7
Dryopteridaceae	Cyrtomium yamamotoi Tagawa	atpA	KT831785	FTOL v1.7
Dryopteridaceae	Cyrtomium yamamotoi Tagawa	atpB	KT831817	FTOL v1.7
Dryopteridaceae	Cyrtomium yamamotoi Tagawa	rbcL	KT831855	FTOL v1.7
Dryopteridaceae	Cyrtomium yunnanense Ching	trnL-trnF	AY736345	FTOL v1.7
Dryopteridaceae	Dryopteris × ambroseae Fraser-Jenk. & Jermy	trnL-trnF	MK697576	FTOL v1.7
Dryopteridaceae	Dryopteris × benedictii (Farw.) Wherry	atpA	KF186545	FTOL v1.7
Dryopteridaceae	Dryopteris × benedictii (Farw.) Wherry	rbcL	KF186512	FTOL v1.7
Dryopteridaceae	Dryopteris × deweveri (Jansen) Jansen & Wacht.	trnL-trnF	MK697581	FTOL v1.7
Dryopteridaceae	Dryopteris × ebinoensis Sa.Kurata	rbcL	AB575179	FTOL v1.7
Dryopteridaceae	Dryopteris × holttumii Li Bing Zhang	trnL-trnF	KU500194	FTOL v1.7
Dryopteridaceae	Dryopteris × sarvelae Fraser-Jenk. & Jermy	trnL-trnF	MK697585	FTOL v1.7
Dryopteridaceae	Dryopteris × triploidea Wherry	atpA	KF186546	FTOL v1.7
Dryopteridaceae	Dryopteris × triploidea Wherry	rbcL	KF186513	FTOL v1.7
Dryopteridaceae	Dryopteris acutodentata Ching	rps4-trnS	DQ191874	FTOL v1.7
Dryopteridaceae	Dryopteris adnata (Blume) Alderw.	rps4-trnS	OM950993	FTOL v1.7
Dryopteridaceae	Dryopteris aemula (Aiton) Kuntze	rbcL	AY268881	FTOL v1.7
Dryopteridaceae	Dryopteris aitoniana Pic. Serm.	rbcL	KY073633	FTOL v1.7
Dryopteridaceae	Dryopteris aitoniana Pic. Serm.	trnL-trnF	KY073696	FTOL v1.7
Dryopteridaceae	Dryopteris alpestris Tagawa	rbcL	JN189536	FTOL v1.7
Dryopteridaceae	Dryopteris alpestris Tagawa	rps4-trnS	JN189210	FTOL v1.7
Dryopteridaceae	Dryopteris alpestris Tagawa	trnL-trnF	JN189105	FTOL v1.7
Dryopteridaceae	Dryopteris amurensis (Milde) Christ	rbcL	AY268867	FTOL v1.7
Dryopteridaceae	Dryopteris anadroma Mitsuta	rbcL	AB575113	FTOL v1.7
Dryopteridaceae	Dryopteris annamensis (Tagawa) Li Bing Zhang	atpB	EF450510	FTOL v1.7
Dryopteridaceae	Dryopteris annamensis (Tagawa) Li Bing Zhang	rbcL	EF463125	FTOL v1.7
Dryopteridaceae	Dryopteris annamensis (Tagawa) Li Bing Zhang	trnL-trnF	EF540698	FTOL v1.7
Dryopteridaceae	Dryopteris antarctica (Baker) C. Chr.	rbcL	JN189577	FTOL v1.7
Dryopteridaceae	Dryopteris antarctica (Baker) C. Chr.	rps4-trnS	JN189250	FTOL v1.7
Dryopteridaceae	Dryopteris antarctica (Baker) C. Chr.	trnL-trnF	JN189141	FTOL v1.7
Dryopteridaceae	Dryopteris anthracinisquama Miyam.	rbcL	AB575114	FTOL v1.7
Dryopteridaceae	Dryopteris apiciflora (Wall. ex Mett.) Kuntze	atpB	KJ196463	FTOL v1.7
Dryopteridaceae	Dryopteris apiciflora (Wall. ex Mett.) Kuntze	rbcL	KJ196860	FTOL v1.7
Dryopteridaceae	Dryopteris apiciflora (Wall. ex Mett.) Kuntze	trnL-trnF	KJ196641	FTOL v1.7
Dryopteridaceae	Dryopteris aquilinoides (Desv.) C. Chr.	rbcL	JN189537	FTOL v1.7
Dryopteridaceae	Dryopteris aquilinoides (Desv.) C. Chr.	rps4-trnS	JN189211	FTOL v1.7
Dryopteridaceae	Dryopteris aquilinoides (Desv.) C. Chr.	trnL-trnF	JN189106	FTOL v1.7
Dryopteridaceae	Dryopteris ardechensis Fraser-Jenk.	rbcL	JN189596	FTOL v1.7
Dryopteridaceae	Dryopteris ardechensis Fraser-Jenk.	rps4-trnS	JN189271	FTOL v1.7
Dryopteridaceae	Dryopteris ardechensis Fraser-Jenk.	trnL-trnF	JN189163	FTOL v1.7
Dryopteridaceae	Dryopteris arguta (Kaulf.) Watt.	rbcL	JQ935258	FTOL v1.7
Dryopteridaceae	Dryopteris arguta (Kaulf.) Watt.	rps4-trnS	JQ936838	FTOL v1.7
Dryopteridaceae	Dryopteris arguta (Kaulf.) Watt.	trnL-trnF	AY278397	FTOL v1.7
Dryopteridaceae	Dryopteris athamantica (Kunze) Kuntze	rbcL	KY073636	FTOL v1.7
Dryopteridaceae	Dryopteris athamantica (Kunze) Kuntze	rps4-trnS	JN189212	FTOL v1.7
Dryopteridaceae	Dryopteris athamantica (Kunze) Kuntze	trnL-trnF	KY073698	FTOL v1.7
Dryopteridaceae	Dryopteris atrata (Wall) Ching	rbcL	DQ508771	FTOL v1.7
Dryopteridaceae	Dryopteris atrata (Wall) Ching	trnL-trnF	DQ514493	FTOL v1.7
Dryopteridaceae	Dryopteris barbigera (Hook.) Kuntze	rbcL	JN189539	FTOL v1.7
Dryopteridaceae	Dryopteris barbigera (Hook.) Kuntze	rps4-trnS	JN189213	FTOL v1.7
Dryopteridaceae	Dryopteris barbigera (Hook.) Kuntze	trnL-trnF	JN189108	FTOL v1.7
Dryopteridaceae	Dryopteris basisora Christ	rbcL	AY587119	FTOL v1.7
Dryopteridaceae	Dryopteris basisora Christ	rps4-trnS	DQ191828	FTOL v1.7
Dryopteridaceae	Dryopteris bernieri Tardieu	rbcL	KY073638	FTOL v1.7
Dryopteridaceae	Dryopteris blanfordii (Hope) C. Chr.	plastome	LT827127	FTOL v1.7
Dryopteridaceae	Dryopteris bodinieri (Christ) C. Chr.	rbcL	DQ508772	FTOL v1.7
Dryopteridaceae	Dryopteris bodinieri (Christ) C. Chr.	trnL-trnF	DQ514494	FTOL v1.7
Dryopteridaceae	Dryopteris bojeri (Baker) Kuntze	rbcL	KY073639	FTOL v1.7
Dryopteridaceae	Dryopteris bojeri (Baker) Kuntze	rps4-trnS	KY073663	FTOL v1.7
Dryopteridaceae	Dryopteris borreri (Newman) Oberh. & Tavel	rbcL	JN189557	FTOL v1.7
Dryopteridaceae	Dryopteris borreri (Newman) Oberh. & Tavel	rps4-trnS	JN189231	FTOL v1.7
Dryopteridaceae	Dryopteris borreri (Newman) Oberh. & Tavel	trnL-trnF	JN189126	FTOL v1.7
Dryopteridaceae	Dryopteris cacaina Tagawa	rbcL	AB575180	FTOL v1.7
Dryopteridaceae	Dryopteris cambrensis (Fraser-Jenk.) Beitel & W. R. Bu	trnL-trnF	KY073700	FTOL v1.7
Dryopteridaceae	Dryopteris campyloptera (Kunze) Clarkson	rbcL	JQ935255	FTOL v1.7
Dryopteridaceae	Dryopteris campyloptera (Kunze) Clarkson	rps4-trnS	JQ936819	FTOL v1.7
Dryopteridaceae	Dryopteris campyloptera (Kunze) Clarkson	trnL-trnF	JN105306	FTOL v1.7
Dryopteridaceae	Dryopteris carthusiana (Vill.) H. P. Fuchs	rbcL	JQ935272	FTOL v1.7
Dryopteridaceae	Dryopteris carthusiana (Vill.) H. P. Fuchs	rps4-trnS	JQ936843	FTOL v1.7
Dryopteridaceae	Dryopteris carthusiana (Vill.) H. P. Fuchs	trnL-trnF	JQ682990	FTOL v1.7
Dryopteridaceae	Dryopteris caucasica (A. Braun) Fraser-Jenk. & Corley	rbcL	JN189516	FTOL v1.7
Dryopteridaceae	Dryopteris caucasica (A. Braun) Fraser-Jenk. & Corley	rps4-trnS	JN189190	FTOL v1.7
Dryopteridaceae	Dryopteris caucasica (A. Braun) Fraser-Jenk. & Corley	trnL-trnF	JN189085	FTOL v1.7
Dryopteridaceae	Dryopteris caudipinna Nakai	rbcL	LC043077	FTOL v1.7
Dryopteridaceae	Dryopteris celsa (W. Palmer) Knowlt.	rbcL	JQ935249	FTOL v1.7
Dryopteridaceae	Dryopteris celsa (W. Palmer) Knowlt.	rps4-trnS	JQ936822	FTOL v1.7
Dryopteridaceae	Dryopteris celsa (W. Palmer) Knowlt.	trnL-trnF	JN105314	FTOL v1.7
Dryopteridaceae	Dryopteris championii (Benth.) C. Chr. apud Ching	plastome	OR601539	FTOL v1.7
Dryopteridaceae	Dryopteris chinensis (Baker) Koidz.	rbcL	JX535859	FTOL v1.7
Dryopteridaceae	Dryopteris chinensis (Baker) Koidz.	rps4	JX535819	FTOL v1.7
Dryopteridaceae	Dryopteris chinensis (Baker) Koidz.	rps4-trnS	JX535819	FTOL v1.7
Dryopteridaceae	Dryopteris chinensis (Baker) Koidz.	trnL-trnF	JX535921	FTOL v1.7
Dryopteridaceae	Dryopteris christensenae (Ching) Li Bing Zhang	atpB	KJ196491	FTOL v1.7
Dryopteridaceae	Dryopteris christensenae (Ching) Li Bing Zhang	rbcL	KJ196809	FTOL v1.7
Dryopteridaceae	Dryopteris christensenae (Ching) Li Bing Zhang	trnL-trnF	KJ196679	FTOL v1.7
Dryopteridaceae	Dryopteris chrysocoma (Christ) C. Chr.	rbcL	DQ508773	FTOL v1.7
Dryopteridaceae	Dryopteris chrysocoma (Christ) C. Chr.	trnL-trnF	DQ514495	FTOL v1.7
Dryopteridaceae	Dryopteris cinnamomea (Cav.) C. Chr.	rbcL	JN189528	FTOL v1.7
Dryopteridaceae	Dryopteris cinnamomea (Cav.) C. Chr.	rps4-trnS	JN189202	FTOL v1.7
Dryopteridaceae	Dryopteris cinnamomea (Cav.) C. Chr.	trnL-trnF	JN189097	FTOL v1.7
Dryopteridaceae	Dryopteris clarkei (Baker) Kuntze	rbcL	DQ054525	FTOL v1.7
Dryopteridaceae	Dryopteris clintoniana (D. C. Eaton) Dowell	rbcL	JQ935247	FTOL v1.7
Dryopteridaceae	Dryopteris clintoniana (D. C. Eaton) Dowell	rps4-trnS	JQ936813	FTOL v1.7
Dryopteridaceae	Dryopteris clintoniana (D. C. Eaton) Dowell	trnL-trnF	JQ683004	FTOL v1.7
Dryopteridaceae	Dryopteris cochleata (D. Don) C. Chr.	rbcL	AY587121	FTOL v1.7
Dryopteridaceae	Dryopteris cochleata (D. Don) C. Chr.	rps4-trnS	DQ191833	FTOL v1.7
Dryopteridaceae	Dryopteris cognata (C. Presl) Kuntze	rbcL	KY073640	FTOL v1.7
Dryopteridaceae	Dryopteris commixta Tagawa	rbcL	AB575115	FTOL v1.7
Dryopteridaceae	Dryopteris comorensis (Tardieu) Fraser-Jenk.	rps4-trnS	KY073664	FTOL v1.7
Dryopteridaceae	Dryopteris conjugata Ching	rbcL	AY587116	FTOL v1.7
Dryopteridaceae	Dryopteris conjugata Ching	rps4-trnS	DQ191834	FTOL v1.7
Dryopteridaceae	Dryopteris coreanomontana Nakai	rbcL	AY268869	FTOL v1.7
Dryopteridaceae	Dryopteris coreanomontana Nakai	trnL-trnF	AY268804	FTOL v1.7
Dryopteridaceae	Dryopteris corleyi Fraser-Jenk.	rbcL	AY268873	FTOL v1.7

Dryopteridaceae	Dryopteris corleyi Fraser-Jenk.	trnL-trnF	AY268808	FTOL v1.7
Dryopteridaceae	Dryopteris costalisora Tagawa	rbcL	JN189603	FTOL v1.7
Dryopteridaceae	Dryopteris costalisora Tagawa	rps4-trnS	JN189278	FTOL v1.7
Dryopteridaceae	Dryopteris costalisora Tagawa	trnL-trnF	JN189170	FTOL v1.7
Dryopteridaceae	Dryopteris crassirhizoma Nakai	plastome	MN712465	FTOL v1.7
Dryopteridaceae	Dryopteris crinalis (Hook. & Arn.) C. Chr.	rbcL	AY268835	FTOL v1.7
Dryopteridaceae	Dryopteris crinalis (Hook. & Arn.) C. Chr.	trnL-trnF	AY268774	FTOL v1.7
Dryopteridaceae	Dryopteris crispifolia Rasbach, Reichst. & Vida	rbcL	JN189597	FTOL v1.7
Dryopteridaceae	Dryopteris crispifolia Rasbach, Reichst. & Vida	rps4-trnS	JN189272	FTOL v1.7
Dryopteridaceae	Dryopteris crispifolia Rasbach, Reichst. & Vida	trnL-trnF	JN189164	FTOL v1.7
Dryopteridaceae	Dryopteris cristata (L.) A. Gray	rbcL	JQ935263	FTOL v1.7
Dryopteridaceae	Dryopteris cristata (L.) A. Gray	rps4-trnS	JQ936832	FTOL v1.7
Dryopteridaceae	Dryopteris cristata (L.) A. Gray	trnL-trnF	JQ683002	FTOL v1.7
Dryopteridaceae	Dryopteris cycadina (Franch. & Sav.) C. Chr.	atpA	KT831787	FTOL v1.7
Dryopteridaceae	Dryopteris cycadina (Franch. & Sav.) C. Chr.	atpB	KT831819	FTOL v1.7
Dryopteridaceae	Dryopteris cycadina (Franch. & Sav.) C. Chr.	rbcL	KT831858	FTOL v1.7
Dryopteridaceae	Dryopteris cyclopeltidiformis C. Chr.	rps4-trnS	DQ191836	FTOL v1.7
Dryopteridaceae	Dryopteris decipiens (Hook.) Kuntze	plastome	KY427348	FTOL v1.7
Dryopteridaceae	Dryopteris dehuaensis Ching & K. H. Shing	rps4-trnS	DQ191838	FTOL v1.7
Dryopteridaceae	Dryopteris dehuaensis Ching & K. H. Shing	trnL-trnF	KP686273	FTOL v1.7
Dryopteridaceae	Dryopteris deparioides (T. Moore) Kuntze	plastome	MW796571	FTOL v1.7
Dryopteridaceae	Dryopteris dickinsii (Franch. & Sav.) C. Chr.	rbcL	JN189598	FTOL v1.7
Dryopteridaceae	Dryopteris dickinsii (Franch. & Sav.) C. Chr.	rps4-trnS	JN189273	FTOL v1.7
Dryopteridaceae	Dryopteris dickinsii (Franch. & Sav.) C. Chr.	trnL-trnF	JN189165	FTOL v1.7
Dryopteridaceae	Dryopteris dicksonioides (Mett. ex Kuhn) Copel.	rbcL	KY099798	FTOL v1.7
Dryopteridaceae	Dryopteris diffracta (Baker) C. Chr.	atpB	EF450504	FTOL v1.7
Dryopteridaceae	Dryopteris diffracta (Baker) C. Chr.	rbcL	DQ508758	FTOL v1.7
Dryopteridaceae	Dryopteris diffracta (Baker) C. Chr.	rps4	OQ144946	FTOL v1.7
Dryopteridaceae	Dryopteris diffracta (Baker) C. Chr.	rps4-trnS	OQ144946	FTOL v1.7
Dryopteridaceae	Dryopteris diffracta (Baker) C. Chr.	trnL-trnF	DQ514478	FTOL v1.7
Dryopteridaceae	Dryopteris dilatata (Hoffm.) A. Gray	plastome	MT984525	FTOL v1.7
Dryopteridaceae	Dryopteris dracomontana Schelpe & N. C. Anthony	rbcL	KY073641	FTOL v1.7
Dryopteridaceae	Dryopteris dracomontana Schelpe & N. C. Anthony	rps4-trnS	KY073665	FTOL v1.7
Dryopteridaceae	Dryopteris erythrosora (D. C. Eaton) Kuntze	plastome	MT130675	FTOL v1.7
Dryopteridaceae	Dryopteris expansa (C. Presl) Fraser-Jenk. & Jermy	atpA	EF463674	FTOL v1.7
Dryopteridaceae	Dryopteris expansa (C. Presl) Fraser-Jenk. & Jermy	atpB	EF463390	FTOL v1.7
Dryopteridaceae	Dryopteris expansa (C. Presl) Fraser-Jenk. & Jermy	rbcL	EF463179	FTOL v1.7
Dryopteridaceae	Dryopteris fadenii Pic. Serm.	rbcL	KY073642	FTOL v1.7
Dryopteridaceae	Dryopteris fadenii Pic. Serm.	rps4-trnS	KY073666	FTOL v1.7
Dryopteridaceae	Dryopteris fadenii Pic. Serm.	trnL-trnF	KY073701	FTOL v1.7
Dryopteridaceae	Dryopteris fatuhivensis E. D. Br.	rps4-trnS	JN189275	FTOL v1.7
Dryopteridaceae	Dryopteris fatuhivensis E. D. Br.	trnL-trnF	JN189168	FTOL v1.7
Dryopteridaceae	Dryopteris filix-mas (L.) Schott	plastome	LT618774	FTOL v1.7
Dryopteridaceae	Dryopteris flaccisquama A. Rojas	rbcL	JN189519	FTOL v1.7
Dryopteridaceae	Dryopteris flaccisquama A. Rojas	rps4-trnS	JN189193	FTOL v1.7
Dryopteridaceae	Dryopteris flaccisquama A. Rojas	trnL-trnF	JN189088	FTOL v1.7
Dryopteridaceae	Dryopteris formosana (Christ) C. Chr.	rbcL	JN189545	FTOL v1.7
Dryopteridaceae	Dryopteris formosana (Christ) C. Chr.	rps4-trnS	JN189219	FTOL v1.7
Dryopteridaceae	Dryopteris formosana (Christ) C. Chr.	trnL-trnF	JN189114	FTOL v1.7
Dryopteridaceae	Dryopteris fragrans (L.) Schott	plastome	KX418656	FTOL v1.7
Dryopteridaceae	Dryopteris fuscipes C. Chr.	atpB	OK253038	FTOL v1.7
Dryopteridaceae	Dryopteris fuscipes C. Chr.	rbcL	OK253029	FTOL v1.7
Dryopteridaceae	Dryopteris fuscipes C. Chr.	trnL-trnF	OK253047	FTOL v1.7
Dryopteridaceae	Dryopteris fuscoatra var. lamoureuxii Fraser-Jenk.	rbcL	AY268841	FTOL v1.7
Dryopteridaceae	Dryopteris fuscoatra var. lamoureuxii Fraser-Jenk.	trnL-trnF	AY268783	FTOL v1.7
Dryopteridaceae	Dryopteris futura A. R. Sm.	rbcL	JN189534	FTOL v1.7
Dryopteridaceae	Dryopteris futura A. R. Sm.	rps4-trnS	JN189208	FTOL v1.7
Dryopteridaceae	Dryopteris futura A. R. Sm.	trnL-trnF	JN189103	FTOL v1.7
Dryopteridaceae	Dryopteris gamblei (Hope) C. Chr.	rbcL	AY587112	FTOL v1.7
Dryopteridaceae	Dryopteris gamblei (Hope) C. Chr.	rps4-trnS	DQ191842	FTOL v1.7
Dryopteridaceae	Dryopteris gaoligongensis Z. Y. Zuo, Jin Mei Lu & D. Z. I	plastome	MW796575	FTOL v1.7
Dryopteridaceae	Dryopteris gemmifera S. Y. Dong	atpB	OK253039	FTOL v1.7
Dryopteridaceae	Dryopteris gemmifera S. Y. Dong	rbcL	OK253030	FTOL v1.7
Dryopteridaceae	Dryopteris gemmifera S. Y. Dong	trnL-trnF	OK253048	FTOL v1.7
Dryopteridaceae	Dryopteris glabra var. nuda (Underw.) Fraser-Jenk.	rbcL	AY268843	FTOL v1.7
Dryopteridaceae	Dryopteris glandulosopaleata J. P. Roux	rbcL	KY073643	FTOL v1.7
Dryopteridaceae	Dryopteris glandulosopaleata J. P. Roux	rps4-trnS	KY073667	FTOL v1.7
Dryopteridaceae	Dryopteris glandulosopaleata J. P. Roux	trnL-trnF	KY073702	FTOL v1.7
Dryopteridaceae	Dryopteris goeringiana (Kunze) Koidz.	plastome	MN712463	FTOL v1.7
Dryopteridaceae	Dryopteris goldieana (Hook.) Gray	rbcL	JQ935260	FTOL v1.7
Dryopteridaceae	Dryopteris goldieana (Hook.) Gray	rps4-trnS	JQ936818	FTOL v1.7
Dryopteridaceae	Dryopteris goldieana (Hook.) Gray	trnL-trnF	JQ682993	FTOL v1.7
Dryopteridaceae	Dryopteris guanchica Gibby & Jermy	rbcL	JN189573	FTOL v1.7
Dryopteridaceae	Dryopteris guanchica Gibby & Jermy	rps4-trnS	JN189246	FTOL v1.7
Dryopteridaceae	Dryopteris guanchica Gibby & Jermy	trnL-trnF	JN189137	FTOL v1.7
Dryopteridaceae	Dryopteris guangxiensis S. G. Lu	rbcL	LC371383	FTOL v1.7
Dryopteridaceae	Dryopteris gymnophylla (Baker) C. Chr.	rbcL	JX535860	FTOL v1.7
Dryopteridaceae	Dryopteris gymnophylla (Baker) C. Chr.	rps4	JX535823	FTOL v1.7
Dryopteridaceae	Dryopteris gymnophylla (Baker) C. Chr.	rps4-trnS	JX535823	FTOL v1.7
Dryopteridaceae	Dryopteris gymnophylla (Baker) C. Chr.	trnL-trnF	JX535925	FTOL v1.7
Dryopteridaceae	Dryopteris gymnosora (Makino) C. Chr.	rbcL	JN189546	FTOL v1.7
Dryopteridaceae	Dryopteris gymnosora (Makino) C. Chr.	rps4-trnS	JN189220	FTOL v1.7
Dryopteridaceae	Dryopteris gymnosora (Makino) C. Chr.	trnL-trnF	JN189115	FTOL v1.7
Dryopteridaceae	Dryopteris hadanoi Kurata	rbcL	AB575133	FTOL v1.7
Dryopteridaceae	Dryopteris handeliana C. Chr.	rbcL	AB575134	FTOL v1.7
Dryopteridaceae	Dryopteris hangchowensis Ching	rbcL	LC484413	FTOL v1.7
Dryopteridaceae	Dryopteris hasseltii (Blume) C. Chr.	rbcL	OQ144943	FTOL v1.7
Dryopteridaceae	Dryopteris hasseltii (Blume) C. Chr.	rps4	OQ144947	FTOL v1.7
Dryopteridaceae	Dryopteris hasseltii (Blume) C. Chr.	rps4-trnS	OQ144947	FTOL v1.7
Dryopteridaceae	Dryopteris hasseltii (Blume) C. Chr.	trnL-trnF	OQ144951	FTOL v1.7
Dryopteridaceae	Dryopteris hawaiiensis (Hillebr.) W. J. Rob.	rbcL	JN189580	FTOL v1.7
Dryopteridaceae	Dryopteris hawaiiensis (Hillebr.) W. J. Rob.	rps4-trnS	JN189253	FTOL v1.7
Dryopteridaceae	Dryopteris hawaiiensis (Hillebr.) W. J. Rob.	trnL-trnF	JN189144	FTOL v1.7
Dryopteridaceae	Dryopteris hendersonii (Bedd.) C. Chr.	rbcL	AB575138	FTOL v1.7
Dryopteridaceae	Dryopteris hendersonii (Bedd.) C. Chr.	trnL-trnF	KU500174	FTOL v1.7
Dryopteridaceae	Dryopteris heterolaena C. Chr.	atpB	EF450516	FTOL v1.7
Dryopteridaceae	Dryopteris heterolaena C. Chr.	rbcL	EF460682	FTOL v1.7
Dryopteridaceae	Dryopteris himachalensis Fraser-Jenk.	rps4-trnS	DQ191845	FTOL v1.7
Dryopteridaceae	Dryopteris hirtipes (Blume) Kuntze	rbcL	MT657487	FTOL v1.7
Dryopteridaceae	Dryopteris hondoensis Koidz.	rbcL	JN189583	FTOL v1.7
Dryopteridaceae	Dryopteris hondoensis Koidz.	rps4-trnS	JN189257	FTOL v1.7
Dryopteridaceae	Dryopteris hondoensis Koidz.	trnL-trnF	JN189149	FTOL v1.7
Dryopteridaceae	Dryopteris huberi (Christ) C. Chr.	rbcL	JN189520	FTOL v1.7
Dryopteridaceae	Dryopteris huberi (Christ) C. Chr.	rps4-trnS	JN189194	FTOL v1.7
Dryopteridaceae	Dryopteris huberi (Christ) C. Chr.	trnL-trnF	JN189089	FTOL v1.7
Dryopteridaceae	Dryopteris huongii C.W.Chen	rbcL	OQ144941	FTOL v1.7
Dryopteridaceae	Dryopteris huongii C.W.Chen	rps4	OQ144944	FTOL v1.7
Dryopteridaceae	Dryopteris huongii C.W.Chen	rps4-trnS	OQ144944	FTOL v1.7
Dryopteridaceae	Dryopteris huongii C.W.Chen	trnL-trnF	OQ144948	FTOL v1.7
Dryopteridaceae	Dryopteris hypolepioides Rosenst.	trnL-trnF	KU500186	FTOL v1.7
Dryopteridaceae	Dryopteris inaequalis (Schltdl.) Kuntze	rbcL	JN189548	FTOL v1.7
Dryopteridaceae	Dryopteris inaequalis (Schltdl.) Kuntze	rps4-trnS	JN189222	FTOL v1.7
Dryopteridaceae	Dryopteris inaequalis (Schltdl.) Kuntze	trnL-trnF	JN189117	FTOL v1.7
Dryopteridaceae	Dryopteris indusiata (Makino) Makino & Yamam.	rbcL	LC484414	FTOL v1.7
Dryopteridaceae	Dryopteris insularis var. chichishimensis (Nakai ex Itô)	rbcL	AB575141	FTOL v1.7
Dryopteridaceae	Dryopteris integriloba C. Chr.	atpB	KJ196504	FTOL v1.7
Dryopteridaceae	Dryopteris integriloba C. Chr.	rbcL	KJ196808	FTOL v1.7
Dryopteridaceae	Dryopteris integriloba C. Chr.	trnL-trnF	KJ196701	FTOL v1.7
Dryopteridaceae	Dryopteris intermedia (Muhl. ex Willd.) A. Gray	rbcL	JQ935262	FTOL v1.7
Dryopteridaceae	Dryopteris intermedia (Muhl. ex Willd.) A. Gray	rps4-trnS	JQ936812	FTOL v1.7
Dryopteridaceae	Dryopteris intermedia (Muhl. ex Willd.) A. Gray	trnL-trnF	JQ682992	FTOL v1.7
Dryopteridaceae	Dryopteris jinpingensis Z.Y. Zuo, J. Mei Lu & D.Z.	plastome	PP148899	FTOL v1.7
Dryopteridaceae	Dryopteris jishouensis G. X. Chen & D. G. Zhang	rbcL	MZ444598	FTOL v1.7
Dryopteridaceae	Dryopteris juxtaposita Christ	rbcL	JN189549	FTOL v1.7
Dryopteridaceae	Dryopteris juxtaposita Christ	rps4-trnS	JN189223	FTOL v1.7
Dryopteridaceae	Dryopteris juxtaposita Christ	trnL-trnF	JN189118	FTOL v1.7
Dryopteridaceae	Dryopteris karwinskyana (Mett.) Kuntze	rbcL	JN189521	FTOL v1.7
Dryopteridaceae	Dryopteris karwinskyana (Mett.) Kuntze	rps4-trnS	JN189195	FTOL v1.7
Dryopteridaceae	Dryopteris karwinskyana (Mett.) Kuntze	trnL-trnF	JN189090	FTOL v1.7
Dryopteridaceae	Dryopteris katangaensis J. P. Roux	rbcL	KY073645	FTOL v1.7
Dryopteridaceae	Dryopteris katangaensis J. P. Roux	rps4-trnS	KY073668	FTOL v1.7
Dryopteridaceae	Dryopteris katangaensis J. P. Roux	trnL-trnF	KY073704	FTOL v1.7
Dryopteridaceae	Dryopteris kawakamii Hayata	atpB	KJ196493	FTOL v1.7
Dryopteridaceae	Dryopteris kawakamii Hayata	rbcL	KJ196812	FTOL v1.7
Dryopteridaceae	Dryopteris kawakamii Hayata	trnL-trnF	KJ196686	FTOL v1.7
Dryopteridaceae	Dryopteris kilemensis (Kuhn) Kuntze	rbcL	KY073646	FTOL v1.7
Dryopteridaceae	Dryopteris kilemensis (Kuhn) Kuntze	rps4-trnS	KY073669	FTOL v1.7
Dryopteridaceae	Dryopteris kilemensis (Kuhn) Kuntze	trnL-trnF	KY073705	FTOL v1.7
Dryopteridaceae	Dryopteris kinkiensis Koidz.	rbcL	KC896548	FTOL v1.7
Dryopteridaceae	Dryopteris kinkiensis Koidz.	trnL-trnF	KC896582	FTOL v1.7
Dryopteridaceae	Dryopteris kinokuniensis Kurata	rbcL	AB575145	FTOL v1.7
Dryopteridaceae	Dryopteris knoblochii A. R. Sm.	rbcL	JN189522	FTOL v1.7
Dryopteridaceae	Dryopteris knoblochii A. R. Sm.	rps4-trnS	JN189196	FTOL v1.7
Dryopteridaceae	Dryopteris knoblochii A. R. Sm.	trnL-trnF	JN189091	FTOL v1.7
Dryopteridaceae	Dryopteris koidzumiana Tagawa	rbcL	AB575146	FTOL v1.7
Dryopteridaceae	Dryopteris komarovii Kossinsky	rbcL	JN189550	FTOL v1.7
Dryopteridaceae	Dryopteris komarovii Kossinsky	rps4-trnS	JN189224	FTOL v1.7
Dryopteridaceae	Dryopteris komarovii Kossinsky	trnL-trnF	JN189119	FTOL v1.7
Dryopteridaceae	Dryopteris labordei (Christ) C. Chr.	atpB	EF450493	FTOL v1.7
Dryopteridaceae	Dryopteris labordei (Christ) C. Chr.	rbcL	EF463128	FTOL v1.7
Dryopteridaceae	Dryopteris lacera (Thunb.) Kuntze	rbcL	JN189585	FTOL v1.7
Dryopteridaceae	Dryopteris lacera (Thunb.) Kuntze	rps4-trnS	JN189259	FTOL v1.7
Dryopteridaceae	Dryopteris lacera (Thunb.) Kuntze	trnL-trnF	JN189151	FTOL v1.7
Dryopteridaceae	Dryopteris lachoongensis (Bedd.) B. K. Nayar & Kaur	rps4-trnS	DQ191852	FTOL v1.7
Dryopteridaceae	Dryopteris lepidopoda Hayata	rbcL	AY587117	FTOL v1.7
Dryopteridaceae	Dryopteris lepidopoda Hayata	rps4-trnS	DQ191853	FTOL v1.7
Dryopteridaceae	Dryopteris lepidorachis C. Chr.	rps4-trnS	DQ191854	FTOL v1.7
Dryopteridaceae	Dryopteris lewalleana Pic. Serm.	rbcL	KY073648	FTOL v1.7
Dryopteridaceae	Dryopteris lewalleana Pic. Serm.	rps4-trnS	KY073670	FTOL v1.7
Dryopteridaceae	Dryopteris liangkwangensis Ching	rps4-trnS	DQ191855	FTOL v1.7
Dryopteridaceae	Dryopteris liboensis P. S. Wang, X. Y. Wang & Li Bing Z	rbcL	LC371382	FTOL v1.7
Dryopteridaceae	Dryopteris ludens (Baker) C. Chr.	rbcL	ON437592	FTOL v1.7
Dryopteridaceae	Dryopteris ludens (Baker) C. Chr.	trnL-trnF	ON437596	FTOL v1.7
Dryopteridaceae	Dryopteris ludoviciana (Kunze) Small	rbcL	JQ935277	FTOL v1.7
Dryopteridaceae	Dryopteris ludoviciana (Kunze) Small	rps4-trnS	JQ936815	FTOL v1.7
Dryopteridaceae	Dryopteris ludoviciana (Kunze) Small	trnL-trnF	JN105313	FTOL v1.7
Dryopteridaceae	Dryopteris lunanensis (Christ) C. Chr.	rbcL	AB575150	FTOL v1.7
Dryopteridaceae	Dryopteris macrolepidota Copel.	rbcL	KY099799	FTOL v1.7
Dryopteridaceae	Dryopteris macropholis Lorence & W. L. Wagner	rbcL	MT216025	FTOL v1.7
Dryopteridaceae	Dryopteris manipurensis (Bedd.) C. Chr.	trnL-trnF	KU500187	FTOL v1.7
Dryopteridaceae	Dryopteris manniana (Hook.) C. Chr.	rbcL	KY073650	FTOL v1.7
Dryopteridaceae	Dryopteris manniana (Hook.) C. Chr.	rps4-trnS	KY073672	FTOL v1.7
Dryopteridaceae	Dryopteris manniana (Hook.) C. Chr.	trnL-trnF	KY073708	FTOL v1.7
Dryopteridaceae	Dryopteris marginalis (L.) Gray	atpA	EF463677	FTOL v1.7
Dryopteridaceae	Dryopteris marginalis (L.) Gray	atpB	EF463393	FTOL v1.7
Dryopteridaceae	Dryopteris marginalis (L.) Gray	rbcL	EF463181	FTOL v1.7
Dryopteridaceae	Dryopteris marginata (Wall. ex Christ) Christ	rps4-trnS	DQ191857	FTOL v1.7
Dryopteridaceae	Dryopteris mauiensis C. Chr.	rbcL	AY268833	FTOL v1.7
Dryopteridaceae	Dryopteris mauiensis C. Chr.	trnL-trnF	AY268770	FTOL v1.7
Dryopteridaceae	Dryopteris maxima (Baker) C. Chr.	rbcL	ON437593	FTOL v1.7
Dryopteridaceae	Dryopteris maxima (Baker) C. Chr.	trnL-trnF	ON437597	FTOL v1.7
Dryopteridaceae	Dryopteris maximowicziana (Miq.) C. Chr.	rbcL	EF540719	FTOL v1.7
Dryopteridaceae	Dryopteris maximowiczii (Baker) Kuntze	atpB	EF540727	FTOL v1.7
Dryopteridaceae	Dryopteris maximowiczii (Baker) Kuntze	rbcL	EF540721	FTOL v1.7
Dryopteridaceae	Dryopteris maxonii Underw. & C. Chr.	rbcL	JN189529	FTOL v1.7
Dryopteridaceae	Dryopteris maxonii Underw. & C. Chr.	rps4-trnS	JN189203	FTOL v1.7
Dryopteridaceae	Dryopteris maxonii Underw. & C. Chr.	trnL-trnF	JN189098	FTOL v1.7

Dryopteridaceae	Dryopteris melanocarpa Hayata	rbcL	AB575153	FTOL v1.7
Dryopteridaceae	Dryopteris mindshelkensis Pavlov	rbcL	AF240653	FTOL v1.7
Dryopteridaceae	Dryopteris mindshelkensis Pavlov	trnL-trnF	AF240671	FTOL v1.7
Dryopteridaceae	Dryopteris monticola (Makino) C. Chr.	rbcL	KC896540	FTOL v1.7
Dryopteridaceae	Dryopteris monticola (Makino) C. Chr.	trnL-trnF	KC896574	FTOL v1.7
Dryopteridaceae	Dryopteris munchii A. R. Sm.	rbcL	JN189535	FTOL v1.7
Dryopteridaceae	Dryopteris munchii A. R. Sm.	rps4-trnS	JN189209	FTOL v1.7
Dryopteridaceae	Dryopteris munchii A. R. Sm.	trnL-trnF	JN189104	FTOL v1.7
Dryopteridaceae	Dryopteris namegatae (Kurata) Kurata	rbcL	AB575155	FTOL v1.7
Dryopteridaceae	Dryopteris napoleonis (Bory) Kuntze	rps4-trnS	KY073673	FTOL v1.7
Dryopteridaceae	Dryopteris nidus (Baker) Zhang	atpB	KJ196494	FTOL v1.7
Dryopteridaceae	Dryopteris nidus (Baker) Zhang	rbcL	KJ196813	FTOL v1.7
Dryopteridaceae	Dryopteris nidus (Baker) Zhang	trnL-trnF	KJ196687	FTOL v1.7
Dryopteridaceae	Dryopteris nodosa (C. Presl) Li Bing Zhang	atpB	EF450509	FTOL v1.7
Dryopteridaceae	Dryopteris nodosa (C. Presl) Li Bing Zhang	rbcL	EF463106	FTOL v1.7
Dryopteridaceae	Dryopteris nubigena Maxon & C. V. Morton	rbcL	JN189501	FTOL v1.7
Dryopteridaceae	Dryopteris nubigena Maxon & C. V. Morton	trnL-trnF	JN189070	FTOL v1.7
Dryopteridaceae	Dryopteris occidentalis J. P. Roux	rbcL	KY073651	FTOL v1.7
Dryopteridaceae	Dryopteris occidentalis J. P. Roux	rps4-trnS	KY073674	FTOL v1.7
Dryopteridaceae	Dryopteris occidentalis J. P. Roux	trnL-trnF	KY073709	FTOL v1.7
Dryopteridaceae	Dryopteris odontoloma (T. Moore) C. Chr.	rbcL	JN189590	FTOL v1.7
Dryopteridaceae	Dryopteris odontoloma (T. Moore) C. Chr.	rps4-trnS	JN189265	FTOL v1.7
Dryopteridaceae	Dryopteris odontoloma (T. Moore) C. Chr.	trnL-trnF	JN189157	FTOL v1.7
Dryopteridaceae	Dryopteris oligodonta (Desv.) Pic. Serm.	rbcL	JN189574	FTOL v1.7
Dryopteridaceae	Dryopteris oligodonta (Desv.) Pic. Serm.	rps4-trnS	JN189247	FTOL v1.7
Dryopteridaceae	Dryopteris oligodonta (Desv.) Pic. Serm.	trnL-trnF	JN189138	FTOL v1.7
Dryopteridaceae	Dryopteris oreades Fomin	rbcL	AY268850	FTOL v1.7
Dryopteridaceae	Dryopteris pacifica (Nakai) Tagawa	rbcL	JN189594	FTOL v1.7
Dryopteridaceae	Dryopteris pacifica (Nakai) Tagawa	rps4-trnS	JN189269	FTOL v1.7
Dryopteridaceae	Dryopteris pacifica (Nakai) Tagawa	trnL-trnF	JN189161	FTOL v1.7
Dryopteridaceae	Dryopteris paleolata (Pic. Serm.) Li Bing Zhang	atpA	KT831789	FTOL v1.7
Dryopteridaceae	Dryopteris paleolata (Pic. Serm.) Li Bing Zhang	atpB	KT831822	FTOL v1.7
Dryopteridaceae	Dryopteris paleolata (Pic. Serm.) Li Bing Zhang	rbcL	KT831862	FTOL v1.7
Dryopteridaceae	Dryopteris pallida (Bory) Maire & Petitm.	rbcL	JN189591	FTOL v1.7
Dryopteridaceae	Dryopteris pallida (Bory) Maire & Petitm.	rps4-trnS	JN189266	FTOL v1.7
Dryopteridaceae	Dryopteris pallida (Bory) Maire & Petitm.	trnL-trnF	JN189158	FTOL v1.7
Dryopteridaceae	Dryopteris panda (C. B. Clarke) C. Chr.	rbcL	JN189551	FTOL v1.7
Dryopteridaceae	Dryopteris panda (C. B. Clarke) C. Chr.	rps4-trnS	JN189225	FTOL v1.7
Dryopteridaceae	Dryopteris panda (C. B. Clarke) C. Chr.	trnL-trnF	JN189120	FTOL v1.7
Dryopteridaceae	Dryopteris patula (Sw.) Underw.	rbcL	KJ464427	FTOL v1.7
Dryopteridaceae	Dryopteris patula (Sw.) Underw.	rps4-trnS	KJ464668	FTOL v1.7
Dryopteridaceae	Dryopteris patula (Sw.) Underw.	trnL-trnF	KJ464598	FTOL v1.7
Dryopteridaceae	Dryopteris peninsulae Kitag.	atpB	EF450494	FTOL v1.7
Dryopteridaceae	Dryopteris peninsulae Kitag.	rbcL	EF463129	FTOL v1.7
Dryopteridaceae	Dryopteris pentheri (Krasser) C. Chr.	rbcL	JN189576	FTOL v1.7
Dryopteridaceae	Dryopteris pentheri (Krasser) C. Chr.	rps4-trnS	JN189249	FTOL v1.7
Dryopteridaceae	Dryopteris pentheri (Krasser) C. Chr.	trnL-trnF	JN189140	FTOL v1.7
Dryopteridaceae	Dryopteris peranema Li Bing Zhang	plastome	MT130581	FTOL v1.7
Dryopteridaceae	Dryopteris podophylla (Hook.) Kuntze	plastome	OR601540	FTOL v1.7
Dryopteridaceae	Dryopteris polita Rosenst.	atpB	KJ196503	FTOL v1.7
Dryopteridaceae	Dryopteris polita Rosenst.	rbcL	JN189606	FTOL v1.7
Dryopteridaceae	Dryopteris polita Rosenst.	rps4	OQ144945	FTOL v1.7
Dryopteridaceae	Dryopteris polita Rosenst.	rps4-trnS	OQ144945	FTOL v1.7
Dryopteridaceae	Dryopteris polita Rosenst.	trnL-trnF	OQ144949	FTOL v1.7
Dryopteridaceae	Dryopteris polylepis (Franch. & Sav.) C. Chr.	rbcL	JN189589	FTOL v1.7
Dryopteridaceae	Dryopteris polylepis (Franch. & Sav.) C. Chr.	rps4-trnS	JN189263	FTOL v1.7
Dryopteridaceae	Dryopteris polylepis (Franch. & Sav.) C. Chr.	trnL-trnF	JN189155	FTOL v1.7
Dryopteridaceae	Dryopteris protobissetiana K. Hori & N. Murak.	rbcL	AB575157	FTOL v1.7
Dryopteridaceae	Dryopteris pseudocaenopteris (Kunze) Li Bing Zhang	atpA	KT831790	FTOL v1.7
Dryopteridaceae	Dryopteris pseudocaenopteris (Kunze) Li Bing Zhang	atpB	KT831823	FTOL v1.7
Dryopteridaceae	Dryopteris pseudocaenopteris (Kunze) Li Bing Zhang	rbcL	KT831864	FTOL v1.7
Dryopteridaceae	Dryopteris pseudofilix-mas (Fée) Rothm.	rbcL	JN189523	FTOL v1.7
Dryopteridaceae	Dryopteris pseudofilix-mas (Fée) Rothm.	rps4-trnS	JN189197	FTOL v1.7
Dryopteridaceae	Dryopteris pseudofilix-mas (Fée) Rothm.	trnL-trnF	JN189092	FTOL v1.7
Dryopteridaceae	Dryopteris pseudosparsa Ching	rps4-trnS	DQ191866	FTOL v1.7
Dryopteridaceae	Dryopteris pulvinulifera (Bedd.) Kuntze	atpB	OK253041	FTOL v1.7
Dryopteridaceae	Dryopteris pulvinulifera (Bedd.) Kuntze	rbcL	OK253032	FTOL v1.7
Dryopteridaceae	Dryopteris pulvinulifera (Bedd.) Kuntze	trnL-trnF	OK253050	FTOL v1.7
Dryopteridaceae	Dryopteris pycnopteroides (Christ) C. Chr.	rbcL	JN189584	FTOL v1.7
Dryopteridaceae	Dryopteris pycnopteroides (Christ) C. Chr.	rps4-trnS	JN189258	FTOL v1.7
Dryopteridaceae	Dryopteris pycnopteroides (Christ) C. Chr.	trnL-trnF	JN189150	FTOL v1.7
Dryopteridaceae	Dryopteris raiateensis (J. W. Moore) Li Bing Zhang	rbcL	KY099744	FTOL v1.7
Dryopteridaceae	Dryopteris ramosa (Hope) C. Chr.	rbcL	MF694706	FTOL v1.7
Dryopteridaceae	Dryopteris redactopinnata S.K.Basu & Panigrahi	rbcL	MF786528	FTOL v1.7
Dryopteridaceae	Dryopteris reflexosquamata Hayata	rbcL	JN189604	FTOL v1.7
Dryopteridaceae	Dryopteris reflexosquamata Hayata	rps4-trnS	DQ191870	FTOL v1.7
Dryopteridaceae	Dryopteris reflexosquamata Hayata	trnL-trnF	JN189171	FTOL v1.7
Dryopteridaceae	Dryopteris remota (Döll) Druce	rbcL	JQ935253	FTOL v1.7
Dryopteridaceae	Dryopteris remota (Döll) Druce	rps4-trnS	JQ936826	FTOL v1.7
Dryopteridaceae	Dryopteris remota (Döll) Druce	trnL-trnF	JQ682983	FTOL v1.7
Dryopteridaceae	Dryopteris renchangiana Z. Y. Zuo & D. Z. Li	plastome	MW796570	FTOL v1.7
Dryopteridaceae	Dryopteris rossii C. Chr.	rbcL	JN189531	FTOL v1.7
Dryopteridaceae	Dryopteris rossii C. Chr.	rps4-trnS	JN189205	FTOL v1.7
Dryopteridaceae	Dryopteris rossii C. Chr.	trnL-trnF	JN189100	FTOL v1.7
Dryopteridaceae	Dryopteris rosthornii (Diels) C. Chr.	rbcL	JX535861	FTOL v1.7
Dryopteridaceae	Dryopteris rosthornii (Diels) C. Chr.	rps4	JX535839	FTOL v1.7
Dryopteridaceae	Dryopteris rosthornii (Diels) C. Chr.	rps4-trnS	JX535839	FTOL v1.7
Dryopteridaceae	Dryopteris rosthornii (Diels) C. Chr.	trnL-trnF	JX535941	FTOL v1.7
Dryopteridaceae	Dryopteris rubiginosa (Brack.) Kuntze	rbcL	AY268836	FTOL v1.7
Dryopteridaceae	Dryopteris rubrobrunnea W. M. Chu	plastome	MT130582	FTOL v1.7
Dryopteridaceae	Dryopteris ryo-itoana Kurata	rbcL	AB575161	FTOL v1.7
Dryopteridaceae	Dryopteris sabaei (Franch. & Sav.) C. Chr.	rbcL	AB575162	FTOL v1.7
Dryopteridaceae	Dryopteris sacrosancta Koidz.	rbcL	JN189592	FTOL v1.7
Dryopteridaceae	Dryopteris sacrosancta Koidz.	rps4-trnS	JN189267	FTOL v1.7
Dryopteridaceae	Dryopteris sacrosancta Koidz.	trnL-trnF	JN189159	FTOL v1.7
Dryopteridaceae	Dryopteris saffordii C. Chr.	rbcL	JN189525	FTOL v1.7
Dryopteridaceae	Dryopteris saffordii C. Chr.	rps4-trnS	JN189199	FTOL v1.7
Dryopteridaceae	Dryopteris saffordii C. Chr.	trnL-trnF	JN189094	FTOL v1.7
Dryopteridaceae	Dryopteris salvinii (Baker) Kuntze	rbcL	JN189526	FTOL v1.7
Dryopteridaceae	Dryopteris salvinii (Baker) Kuntze	rps4-trnS	JN189200	FTOL v1.7
Dryopteridaceae	Dryopteris salvinii (Baker) Kuntze	trnL-trnF	JN189095	FTOL v1.7
Dryopteridaceae	Dryopteris sandwicensis (Hook. & Arn.) C. Chr.	rbcL	AY268827	FTOL v1.7
Dryopteridaceae	Dryopteris saxifraga H. Itô	rbcL	KC896563	FTOL v1.7
Dryopteridaceae	Dryopteris saxifraga H. Itô	trnL-trnF	KC896597	FTOL v1.7
Dryopteridaceae	Dryopteris saxifragivaria Nakai	rbcL	KC896566	FTOL v1.7
Dryopteridaceae	Dryopteris saxifragivaria Nakai	trnL-trnF	KC896600	FTOL v1.7
Dryopteridaceae	Dryopteris schimperiana (Hochst. ex A. Braun) C. Chr.	rbcL	KY073655	FTOL v1.7
Dryopteridaceae	Dryopteris schimperiana (Hochst. ex A. Braun) C. Chr.	rps4-trnS	KY073675	FTOL v1.7
Dryopteridaceae	Dryopteris schnellii Tardieu	rbcL	KY073656	FTOL v1.7
Dryopteridaceae	Dryopteris scottii (Bedd.) Ching	rbcL	DQ508775	FTOL v1.7
Dryopteridaceae	Dryopteris scottii (Bedd.) Ching	trnL-trnF	DQ514498	FTOL v1.7
Dryopteridaceae	Dryopteris setosa (Thunb. ex Murray) Akasawa	rbcL	JN189587	FTOL v1.7
Dryopteridaceae	Dryopteris setosa (Thunb. ex Murray) Akasawa	rps4-trnS	JN189261	FTOL v1.7
Dryopteridaceae	Dryopteris setosa (Thunb. ex Murray) Akasawa	trnL-trnF	JN189153	FTOL v1.7
Dryopteridaceae	Dryopteris shibipedis Kurata	rbcL	AB575166	FTOL v1.7
Dryopteridaceae	Dryopteris shikokiana (Makino) C. Chr.	rbcL	AB575167	FTOL v1.7
Dryopteridaceae	Dryopteris shikokiana (Makino) C. Chr.	trnL-trnF	KU500175	FTOL v1.7
Dryopteridaceae	Dryopteris shiroumensis Kurata & Nakam.	rbcL	AB575168	FTOL v1.7
Dryopteridaceae	Dryopteris sieboldii (T. Moore) Kuntze	plastome	MN623354	FTOL v1.7
Dryopteridaceae	Dryopteris simasakii var. paleacea (H. Itô) Sa. Kurata	rbcL	AB575171	FTOL v1.7
Dryopteridaceae	Dryopteris sinonepalensis Z. Y. Zhou & Fraser-Jenk.	plastome	MW796578	FTOL v1.7
Dryopteridaceae	Dryopteris sordidipes Tagawa	rbcL	JN189605	FTOL v1.7
Dryopteridaceae	Dryopteris sordidipes Tagawa	rps4-trnS	JN189280	FTOL v1.7
Dryopteridaceae	Dryopteris sordidipes Tagawa	trnL-trnF	JN189172	FTOL v1.7
Dryopteridaceae	Dryopteris sororia (Maxon) M. A. Mc Henry, Sundue & I	rbcL	KC878849	FTOL v1.7
Dryopteridaceae	Dryopteris sororia (Maxon) M. A. Mc Henry, Sundue & I	trnL-trnF	KC878866	FTOL v1.7
Dryopteridaceae	Dryopteris sparsa (D. Don) Kuntze	plastome	MZ960495	FTOL v1.7
Dryopteridaceae	Dryopteris spinosa Copel.	trnL-trnF	JN189145	FTOL v1.7
Dryopteridaceae	Dryopteris squamiseta (Hook.) Kuntze	atpA	EF463678	FTOL v1.7
Dryopteridaceae	Dryopteris squamiseta (Hook.) Kuntze	atpB	EF463394	FTOL v1.7
Dryopteridaceae	Dryopteris squamiseta (Hook.) Kuntze	rbcL	EF463182	FTOL v1.7
Dryopteridaceae	Dryopteris squamiseta (Hook.) Kuntze	trnL-trnF	KU500177	FTOL v1.7
Dryopteridaceae	Dryopteris stenolepis (Baker) C. Chr.	rbcL	JN189553	FTOL v1.7
Dryopteridaceae	Dryopteris stenolepis (Baker) C. Chr.	rps4-trnS	JN189227	FTOL v1.7
Dryopteridaceae	Dryopteris stenolepis (Baker) C. Chr.	trnL-trnF	JN189122	FTOL v1.7
Dryopteridaceae	Dryopteris stewartii Fraser-Jenk.	rbcL	JN189586	FTOL v1.7
Dryopteridaceae	Dryopteris stewartii Fraser-Jenk.	rps4-trnS	JN189260	FTOL v1.7
Dryopteridaceae	Dryopteris stewartii Fraser-Jenk.	trnL-trnF	JN189152	FTOL v1.7
Dryopteridaceae	Dryopteris subarborea (Baker) C. Chr.	trnL-trnF	OM782321	FTOL v1.7
Dryopteridaceae	Dryopteris subbipinnata W. H. Wagner & Hobdy	rbcL	JN189579	FTOL v1.7
Dryopteridaceae	Dryopteris subbipinnata W. H. Wagner & Hobdy	rps4-trnS	JN189252	FTOL v1.7
Dryopteridaceae	Dryopteris subbipinnata W. H. Wagner & Hobdy	trnL-trnF	JN189143	FTOL v1.7
Dryopteridaceae	Dryopteris subcrenulata (Baker) C. Chr.	rbcL	KY073658	FTOL v1.7
Dryopteridaceae	Dryopteris subcrenulata (Baker) C. Chr.	rps4-trnS	KY073677	FTOL v1.7
Dryopteridaceae	Dryopteris subexaltata (Christ) C. Chr.	rbcL	KC896546	FTOL v1.7
Dryopteridaceae	Dryopteris subexaltata (Christ) C. Chr.	trnL-trnF	KC896580	FTOL v1.7
Dryopteridaceae	Dryopteris sublacera Christ	rbcL	DQ508778	FTOL v1.7
Dryopteridaceae	Dryopteris sublacera Christ	trnL-trnF	DQ514501	FTOL v1.7
Dryopteridaceae	Dryopteris submarginata Rosenst.	rps4-trnS	DQ191879	FTOL v1.7
Dryopteridaceae	Dryopteris subpycnopteroides Ching ex Fraser-Jenk.	rbcL	AY587113	FTOL v1.7
Dryopteridaceae	Dryopteris subpycnopteroides Ching ex Fraser-Jenk.	rps4-trnS	DQ191880	FTOL v1.7
Dryopteridaceae	Dryopteris subreflexipinna Ogata	rps4-trnS	EU797688	FTOL v1.7
Dryopteridaceae	Dryopteris subreflexipinna Ogata	trnL-trnF	EU797678	FTOL v1.7
Dryopteridaceae	Dryopteris subtriangularis (Hope) C. Chr.	rbcL	AY587110	FTOL v1.7
Dryopteridaceae	Dryopteris tenuicula Matthew & Christ	rbcL	AB575147	FTOL v1.7
Dryopteridaceae	Dryopteris tetrapinnata W. H. Wagner & Hobdy	rbcL	AY268838	FTOL v1.7
Dryopteridaceae	Dryopteris tokyoensis (Matsum.) C. Chr.	rbcL	JN189578	FTOL v1.7
Dryopteridaceae	Dryopteris tokyoensis (Matsum.) C. Chr.	rps4-trnS	JN189251	FTOL v1.7
Dryopteridaceae	Dryopteris tokyoensis (Matsum.) C. Chr.	trnL-trnF	JN189142	FTOL v1.7
Dryopteridaceae	Dryopteris transmorrisonensis (Hayata) Hayata	trnL-trnF	KU500195	FTOL v1.7
Dryopteridaceae	Dryopteris triangularis Herter	rbcL	JN189600	FTOL v1.7
Dryopteridaceae	Dryopteris triangularis Herter	trnL-trnF	JN189167	FTOL v1.7
Dryopteridaceae	Dryopteris tricellularis J. P. Roux	rbcL	KY073659	FTOL v1.7
Dryopteridaceae	Dryopteris tricellularis J. P. Roux	rps4-trnS	KY073678	FTOL v1.7
Dryopteridaceae	Dryopteris tricellularis J. P. Roux	trnL-trnF	KY073715	FTOL v1.7
Dryopteridaceae	Dryopteris tsoongii Ching	rps4-trnS	DQ191882	FTOL v1.7
Dryopteridaceae	Dryopteris tsugiwoi Kurata	rbcL	AB575175	FTOL v1.7
Dryopteridaceae	Dryopteris tsutsuiana Kurata	rbcL	AB575176	FTOL v1.7
Dryopteridaceae	Dryopteris tyrrhena Fraser-Jenk. & Reichst.	rbcL	KY073660	FTOL v1.7
Dryopteridaceae	Dryopteris tyrrhena Fraser-Jenk. & Reichst.	rps4-trnS	KY073679	FTOL v1.7
Dryopteridaceae	Dryopteris tyrrhena Fraser-Jenk. & Reichst.	trnL-trnF	KY073716	FTOL v1.7
Dryopteridaceae	Dryopteris unidentata var. paleacea (Hillebr.) Fraser-Je	rbcL	AY268832	FTOL v1.7
Dryopteridaceae	Dryopteris uniformis (Makino) Makino	atpA	EF463679	FTOL v1.7
Dryopteridaceae	Dryopteris uniformis (Makino) Makino	atpB	EF463395	FTOL v1.7
Dryopteridaceae	Dryopteris uniformis (Makino) Makino	rbcL	EF463183	FTOL v1.7
Dryopteridaceae	Dryopteris varia (L.) Kuntze	rbcL	AY736329	FTOL v1.7
Dryopteridaceae	Dryopteris varia (L.) Kuntze	trnL-trnF	AY736355	FTOL v1.7
Dryopteridaceae	Dryopteris villarii (Bell) Woyn. ex Schinz & Thell.	plastome	LT905144	FTOL v1.7
Dryopteridaceae	Dryopteris wallichiana (Spreng.) Hyl.	rbcL	KJ464428	FTOL v1.7
Dryopteridaceae	Dryopteris wallichiana (Spreng.) Hyl.	rps4-trnS	KJ464669	FTOL v1.7
Dryopteridaceae	Dryopteris wallichiana (Spreng.) Hyl.	trnL-trnF	KJ464599	FTOL v1.7
Dryopteridaceae	Dryopteris wattsii Mc Keown, Sundue & Barrington	atpA	KP164481	FTOL v1.7
Dryopteridaceae	Dryopteris wattsii Mc Keown, Sundue & Barrington	atpB	KP164488	FTOL v1.7
Dryopteridaceae	Dryopteris wattsii Mc Keown, Sundue & Barrington	rbcL	KP164495	FTOL v1.7
Dryopteridaceae	Dryopteris wulingshanensis J. P. Shu, Y. H. Yan & R. J. V	rbcL	MZ444607	FTOL v1.7
Dryopteridaceae	Dryopteris wuzhaohongii Li Bing Zhang	trnL-trnF	KU500178	FTOL v1.7
Dryopteridaceae	Dryopteris x makabensis Ka.Yamam. & N.Murak.	rbcL	LC010782	FTOL v1.7

Dryopteridaceae	Dryopteris xanthomelas (Christ) C. Chr.	rbcL	AY587118	FTOL v1.7
Dryopteridaceae	Dryopteris xanthomelas (Christ) C. Chr.	rps4-trnS	DQ151857	FTOL v1.7
Dryopteridaceae	Dryopteris xanthomelas (Christ) C. Chr.	trnL-trnF	DQ150394	FTOL v1.7
Dryopteridaceae	Dryopteris yenpingensis C.Chr. & Ching	atpB	OK253044	FTOL v1.7
Dryopteridaceae	Dryopteris yenpingensis C.Chr. & Ching	rbcL	OK253035	FTOL v1.7
Dryopteridaceae	Dryopteris yenpingensis C.Chr. & Ching	trnL-trnF	OK253053	FTOL v1.7
Dryopteridaceae	Dryopteris yongdeensis W. M. Chu ex S. G. Lu	atpB	OK253042	FTOL v1.7
Dryopteridaceae	Dryopteris yongdeensis W. M. Chu ex S. G. Lu	rbcL	OK253033	FTOL v1.7
Dryopteridaceae	Dryopteris yongdeensis W. M. Chu ex S. G. Lu	trnL-trnF	OK253051	FTOL v1.7
Dryopteridaceae	Dryopteris yoroii Seriz.	plastome	MW796577	FTOL v1.7
Dryopteridaceae	Dryopteris zayuensis Ching & S. K. Wu	rbcL	DQ508777	FTOL v1.7
Dryopteridaceae	Dryopteris zayuensis Ching & S. K. Wu	trnL-trnF	DQ514500	FTOL v1.7
Dryopteridaceae	Elaphoglossum achroalepis (Baker) C. Chr.	atpB	EF040636	FTOL v1.7
Dryopteridaceae	Elaphoglossum achroalepis (Baker) C. Chr.	rps4-trnS	AY540225	FTOL v1.7
Dryopteridaceae	Elaphoglossum achroalepis (Baker) C. Chr.	trnL-trnF	AY536288	FTOL v1.7
Dryopteridaceae	Elaphoglossum acrostichoides (Hook. & Grev.) Schelpe	rbcL	AY818680	FTOL v1.7
Dryopteridaceae	Elaphoglossum aemulum (Kaulf.) Brack.	rbcL	MT657681	FTOL v1.7
Dryopteridaceae	Elaphoglossum aemulum (Kaulf.) Brack.	rps4-trnS	AY540227	FTOL v1.7
Dryopteridaceae	Elaphoglossum aemulum (Kaulf.) Brack.	trnL-trnF	AY536290	FTOL v1.7
Dryopteridaceae	Elaphoglossum affine (M. Martens & Galeotti) T. Moor	rbcL	AY534861	FTOL v1.7
Dryopteridaceae	Elaphoglossum affine (M. Martens & Galeotti) T. Moor	rps4-trnS	AY536169	FTOL v1.7
Dryopteridaceae	Elaphoglossum affine (M. Martens & Galeotti) T. Moor	trnL-trnF	AY534841	FTOL v1.7
Dryopteridaceae	Elaphoglossum alansmithii Mickel	rbcL	MW620280	FTOL v1.7
Dryopteridaceae	Elaphoglossum albomarginatum A. R. Sm.	atpB	MG600320	FTOL v1.7
Dryopteridaceae	Elaphoglossum albomarginatum A. R. Sm.	rps4-trnS	MG600350	FTOL v1.7
Dryopteridaceae	Elaphoglossum albomarginatum A. R. Sm.	trnL-trnF	MG600378	FTOL v1.7
Dryopteridaceae	Elaphoglossum alpestre (Gardner) T. Moore	atpB	MG600302	FTOL v1.7
Dryopteridaceae	Elaphoglossum alpestre (Gardner) T. Moore	rps4-trnS	MG600331	FTOL v1.7
Dryopteridaceae	Elaphoglossum alpestre (Gardner) T. Moore	trnL-trnF	MG600360	FTOL v1.7
Dryopteridaceae	Elaphoglossum alvaradoanum A. Rojas	atpB	EF040651	FTOL v1.7
Dryopteridaceae	Elaphoglossum alvaradoanum A. Rojas	rps4-trnS	EF040625	FTOL v1.7
Dryopteridaceae	Elaphoglossum alvaradoanum A. Rojas	trnL-trnF	EF040611	FTOL v1.7
Dryopteridaceae	Elaphoglossum amygdalifolium (Mett. ex Kuhn) Christ	atpA	EF463680	FTOL v1.7
Dryopteridaceae	Elaphoglossum amygdalifolium (Mett. ex Kuhn) Christ	atpB	EF463396	FTOL v1.7
Dryopteridaceae	Elaphoglossum amygdalifolium (Mett. ex Kuhn) Christ	rbcL	EF463184	FTOL v1.7
Dryopteridaceae	Elaphoglossum andicola (Fée) T. Moore	atpA	EF463681	FTOL v1.7
Dryopteridaceae	Elaphoglossum andicola (Fée) T. Moore	atpB	EF463397	FTOL v1.7
Dryopteridaceae	Elaphoglossum andicola (Fée) T. Moore	rbcL	EF463185	FTOL v1.7
Dryopteridaceae	Elaphoglossum andreanum Christ	atpB	KJ528046	FTOL v1.7
Dryopteridaceae	Elaphoglossum andreanum Christ	rps4-trnS	KJ528132	FTOL v1.7
Dryopteridaceae	Elaphoglossum andreanum Christ	trnL-trnF	KJ528168	FTOL v1.7
Dryopteridaceae	Elaphoglossum angulatum (Blume) T. Moore	rbcL	KF992484	FTOL v1.7
Dryopteridaceae	Elaphoglossum anthracinum A. Vasco, Mickel & R. C. M	rps4-trnS	KJ528116	FTOL v1.7
Dryopteridaceae	Elaphoglossum anthracinum A. Vasco, Mickel & R. C. M	trnL-trnF	KJ528172	FTOL v1.7
Dryopteridaceae	Elaphoglossum apodum (Kaulf.) Schott	rbcL	AY818681	FTOL v1.7
Dryopteridaceae	Elaphoglossum asterolepis (Baker) C. Chr.	atpB	EF040642	FTOL v1.7
Dryopteridaceae	Elaphoglossum asterolepis (Baker) C. Chr.	rps4-trnS	AY540231	FTOL v1.7
Dryopteridaceae	Elaphoglossum asterolepis (Baker) C. Chr.	trnL-trnF	AY536294	FTOL v1.7
Dryopteridaceae	Elaphoglossum aubertii (Desv.) T. Moore	atpA	EF463682	FTOL v1.7
Dryopteridaceae	Elaphoglossum aubertii (Desv.) T. Moore	atpB	EF463398	FTOL v1.7
Dryopteridaceae	Elaphoglossum aubertii (Desv.) T. Moore	rbcL	EF463186	FTOL v1.7
Dryopteridaceae	Elaphoglossum auricomum (Kunze) T. Moore	rbcL	AY818682	FTOL v1.7
Dryopteridaceae	Elaphoglossum auricomum (Kunze) T. Moore	rps4-trnS	AY536145	FTOL v1.7
Dryopteridaceae	Elaphoglossum auricomum (Kunze) T. Moore	trnL-trnF	AY534817	FTOL v1.7
Dryopteridaceae	Elaphoglossum auripilum Christ	rbcL	AY534849	FTOL v1.7
Dryopteridaceae	Elaphoglossum austromarquesense Rouhan & Lorence	rbcL	MT216028	FTOL v1.7
Dryopteridaceae	Elaphoglossum avaratraense Rakotondr.	rps4-trnS	AY540233	FTOL v1.7
Dryopteridaceae	Elaphoglossum avaratraense Rakotondr.	trnL-trnF	AY536296	FTOL v1.7
Dryopteridaceae	Elaphoglossum backhouseanum T. Moore	atpA	EF463683	FTOL v1.7
Dryopteridaceae	Elaphoglossum backhouseanum T. Moore	atpB	EF463399	FTOL v1.7
Dryopteridaceae	Elaphoglossum backhouseanum T. Moore	rbcL	EF463187	FTOL v1.7
Dryopteridaceae	Elaphoglossum beaurepairei (Fée) Brade	atpB	MG600304	FTOL v1.7
Dryopteridaceae	Elaphoglossum beaurepairei (Fée) Brade	rps4-trnS	MG600333	FTOL v1.7
Dryopteridaceae	Elaphoglossum beaurepairei (Fée) Brade	trnL-trnF	MG600361	FTOL v1.7
Dryopteridaceae	Elaphoglossum bellermannianum (Klotzsch) T. Moore	atpB	KJ528040	FTOL v1.7
Dryopteridaceae	Elaphoglossum bellermannianum (Klotzsch) T. Moore	rps4-trnS	KJ528161	FTOL v1.7
Dryopteridaceae	Elaphoglossum bellermannianum (Klotzsch) T. Moore	trnL-trnF	KJ528173	FTOL v1.7
Dryopteridaceae	Elaphoglossum bifurcatum (Jacq.) Sw.	trnL-trnF	AY194070	FTOL v1.7
Dryopteridaceae	Elaphoglossum biolleyi Christ	rps4-trnS	AY540235	FTOL v1.7
Dryopteridaceae	Elaphoglossum biolleyi Christ	trnL-trnF	AY536298	FTOL v1.7
Dryopteridaceae	Elaphoglossum blepharis A. Vasco, Mickel & R. C. Mora	rps4-trnS	KJ528145	FTOL v1.7
Dryopteridaceae	Elaphoglossum blepharis A. Vasco, Mickel & R. C. Mora	trnL-trnF	KJ528175	FTOL v1.7
Dryopteridaceae	Elaphoglossum boryanum (Fée) T. Moore	rbcL	AY534846	FTOL v1.7
Dryopteridaceae	Elaphoglossum boryanum (Fée) T. Moore	rps4-trnS	AY536133	FTOL v1.7
Dryopteridaceae	Elaphoglossum boryanum (Fée) T. Moore	trnL-trnF	AY534804	FTOL v1.7
Dryopteridaceae	Elaphoglossum brenesii Mickel	rbcL	MW138163	FTOL v1.7
Dryopteridaceae	Elaphoglossum brevipetiolatum F. B. Matos & Mickel	rps4-trnS	MG600334	FTOL v1.7
Dryopteridaceae	Elaphoglossum brevipetiolatum F. B. Matos & Mickel	trnL-trnF	MG600362	FTOL v1.7
Dryopteridaceae	Elaphoglossum bryogenes Mickel	rps4-trnS	KJ528135	FTOL v1.7
Dryopteridaceae	Elaphoglossum bryogenes Mickel	trnL-trnF	KJ528176	FTOL v1.7
Dryopteridaceae	Elaphoglossum burchellii (Baker) C. Chr.	rbcL	AY818683	FTOL v1.7
Dryopteridaceae	Elaphoglossum callifolium (Blume) T. Moore	rbcL	AB232400	FTOL v1.7
Dryopteridaceae	Elaphoglossum cardenasii Wagner	rbcL	AY818684	FTOL v1.7
Dryopteridaceae	Elaphoglossum cardenasii Wagner	rps4-trnS	AY536131	FTOL v1.7
Dryopteridaceae	Elaphoglossum cardenasii Wagner	trnL-trnF	AY534802	FTOL v1.7
Dryopteridaceae	Elaphoglossum cardiophyllum (Hook.) T. Moore	rbcL	AY818685	FTOL v1.7
Dryopteridaceae	Elaphoglossum cardiophyllum (Hook.) T. Moore	rps4-trnS	AY536171	FTOL v1.7
Dryopteridaceae	Elaphoglossum cardiophyllum (Hook.) T. Moore	trnL-trnF	AY534842	FTOL v1.7
Dryopteridaceae	Elaphoglossum caricifolium Mickel	rbcL	AY818708	FTOL v1.7
Dryopteridaceae	Elaphoglossum caricifolium Mickel	rps4-trnS	AY536143	FTOL v1.7
Dryopteridaceae	Elaphoglossum caricifolium Mickel	trnL-trnF	AY534814	FTOL v1.7
Dryopteridaceae	Elaphoglossum carolinense Hosok.	rbcL	MT657690	FTOL v1.7
Dryopteridaceae	Elaphoglossum christianeae Mickel	rbcL	KJ628703	FTOL v1.7
Dryopteridaceae	Elaphoglossum ciliatum (C. Presl) T. Moore	rbcL	MW138220	FTOL v1.7
Dryopteridaceae	Elaphoglossum cismense Rosenst.	atpB	MG600319	FTOL v1.7
Dryopteridaceae	Elaphoglossum cismense Rosenst.	rps4-trnS	MG600349	FTOL v1.7
Dryopteridaceae	Elaphoglossum cismense Rosenst.	trnL-trnF	MG600377	FTOL v1.7
Dryopteridaceae	Elaphoglossum cocosense Mickel	rbcL	KM495087	FTOL v1.7
Dryopteridaceae	Elaphoglossum concinnum Mickel	rps4-trnS	KJ528151	FTOL v1.7
Dryopteridaceae	Elaphoglossum concinnum Mickel	trnL-trnF	KJ528179	FTOL v1.7
Dryopteridaceae	Elaphoglossum conforme (Sw.) Schott	atpB	MZ957367	FTOL v1.7
Dryopteridaceae	Elaphoglossum conforme (Sw.) Schott	rbcL	MZ957133	FTOL v1.7
Dryopteridaceae	Elaphoglossum conforme (Sw.) Schott	rps4	MZ957581	FTOL v1.7
Dryopteridaceae	Elaphoglossum conforme (Sw.) Schott	rps4-trnS	MZ957581	FTOL v1.7
Dryopteridaceae	Elaphoglossum conforme (Sw.) Schott	trnL-trnF	MZ957251	FTOL v1.7
Dryopteridaceae	Elaphoglossum conspersum Christ	rps4-trnS	AY540238	FTOL v1.7
Dryopteridaceae	Elaphoglossum conspersum Christ	trnL-trnF	AY536301	FTOL v1.7
Dryopteridaceae	Elaphoglossum coriaceum Bonap.	atpB	EF040653	FTOL v1.7
Dryopteridaceae	Elaphoglossum coriaceum Bonap.	rps4-trnS	EF040627	FTOL v1.7
Dryopteridaceae	Elaphoglossum coriaceum Bonap.	trnL-trnF	EF040613	FTOL v1.7
Dryopteridaceae	Elaphoglossum costaricense Christ	rbcL	AY818687	FTOL v1.7
Dryopteridaceae	Elaphoglossum costaricense Christ	rps4-trnS	AY536128	FTOL v1.7
Dryopteridaceae	Elaphoglossum costaricense Christ	trnL-trnF	AY534799	FTOL v1.7
Dryopteridaceae	Elaphoglossum coursii Tardieu	rbcL	KF992525	FTOL v1.7
Dryopteridaceae	Elaphoglossum craspedariiforme (Fée) Brade ex Alstor	rbcL	AY818688	FTOL v1.7
Dryopteridaceae	Elaphoglossum craspedariiforme (Fée) Brade ex Alstor	rps4-trnS	AY536158	FTOL v1.7
Dryopteridaceae	Elaphoglossum craspedariiforme (Fée) Brade ex Alstor	trnL-trnF	AY534830	FTOL v1.7
Dryopteridaceae	Elaphoglossum crassicaule Copel.	rbcL	MT657686	FTOL v1.7
Dryopteridaceae	Elaphoglossum crassifolium (Gaudich.) W. R. Andersor	atpA	EF463685	FTOL v1.7
Dryopteridaceae	Elaphoglossum crassifolium (Gaudich.) W. R. Andersor	atpB	EF463401	FTOL v1.7
Dryopteridaceae	Elaphoglossum crassifolium (Gaudich.) W. R. Andersor	rbcL	EF463188	FTOL v1.7
Dryopteridaceae	Elaphoglossum crinitum (L.) Christ	atpA	EF463686	FTOL v1.7
Dryopteridaceae	Elaphoglossum crinitum (L.) Christ	atpB	EF463402	FTOL v1.7
Dryopteridaceae	Elaphoglossum crinitum (L.) Christ	rbcL	EF463189	FTOL v1.7
Dryopteridaceae	Elaphoglossum croatii Mickel	rps4-trnS	AY540241	FTOL v1.7
Dryopteridaceae	Elaphoglossum croatii Mickel	trnL-trnF	AY536304	FTOL v1.7
Dryopteridaceae	Elaphoglossum cubense (Mett.) C. Chr.	atpB	KF212378	FTOL v1.7
Dryopteridaceae	Elaphoglossum cubense (Mett.) C. Chr.	rps4-trnS	KF212429	FTOL v1.7
Dryopteridaceae	Elaphoglossum cubense (Mett.) C. Chr.	trnL-trnF	KF212405	FTOL v1.7
Dryopteridaceae	Elaphoglossum curvans (Kunze) A. Rojas	rbcL	AY818700	FTOL v1.7
Dryopteridaceae	Elaphoglossum curvans (Kunze) A. Rojas	rps4-trnS	AY536152	FTOL v1.7
Dryopteridaceae	Elaphoglossum curvans (Kunze) A. Rojas	trnL-trnF	AY534824	FTOL v1.7
Dryopteridaceae	Elaphoglossum cuspidatum (Willd.) T. Moore	atpB	EU907675	FTOL v1.7
Dryopteridaceae	Elaphoglossum cuspidatum (Willd.) T. Moore	rbcL	AY534856	FTOL v1.7
Dryopteridaceae	Elaphoglossum cuspidatum (Willd.) T. Moore	rps4-trnS	EU907750	FTOL v1.7
Dryopteridaceae	Elaphoglossum cuspidatum (Willd.) T. Moore	trnL-trnF	EU907815	FTOL v1.7
Dryopteridaceae	Elaphoglossum davidsei Mickel	rps4-trnS	AY540242	FTOL v1.7
Dryopteridaceae	Elaphoglossum davidsei Mickel	trnL-trnF	AY536305	FTOL v1.7
Dryopteridaceae	Elaphoglossum decaryanum Tardieu	atpB	EF040658	FTOL v1.7
Dryopteridaceae	Elaphoglossum decaryanum Tardieu	rps4-trnS	AY540243	FTOL v1.7
Dryopteridaceae	Elaphoglossum decaryanum Tardieu	trnL-trnF	AY536306	FTOL v1.7
Dryopteridaceae	Elaphoglossum deckenii (Kuhn) C. Chr.	trnL-trnF	AY194072	FTOL v1.7
Dryopteridaceae	Elaphoglossum decoratum (Kunze) T. Moore	rbcL	KJ464429	FTOL v1.7
Dryopteridaceae	Elaphoglossum decoratum (Kunze) T. Moore	rps4-trnS	KJ464670	FTOL v1.7
Dryopteridaceae	Elaphoglossum decoratum (Kunze) T. Moore	trnL-trnF	KJ464600	FTOL v1.7
Dryopteridaceae	Elaphoglossum decursivum Mickel	atpB	MG600322	FTOL v1.7
Dryopteridaceae	Elaphoglossum decursivum Mickel	rps4-trnS	MG600352	FTOL v1.7
Dryopteridaceae	Elaphoglossum decursivum Mickel	trnL-trnF	MG600380	FTOL v1.7
Dryopteridaceae	Elaphoglossum delgadilloanum A. Rojas	rbcL	KM583470	FTOL v1.7
Dryopteridaceae	Elaphoglossum deltoideum (Sodiro) Christ	atpA	EF463687	FTOL v1.7
Dryopteridaceae	Elaphoglossum deltoideum (Sodiro) Christ	atpB	EF463403	FTOL v1.7
Dryopteridaceae	Elaphoglossum deltoideum (Sodiro) Christ	rbcL	EF463190	FTOL v1.7
Dryopteridaceae	Elaphoglossum dendricola (Baker) Christ	atpB	EU907676	FTOL v1.7
Dryopteridaceae	Elaphoglossum dendricola (Baker) Christ	rps4-trnS	EU907751	FTOL v1.7
Dryopteridaceae	Elaphoglossum dendricola (Baker) Christ	trnL-trnF	EU907816	FTOL v1.7
Dryopteridaceae	Elaphoglossum dimorphum (Hook. & Grev.) T. Moore	atpB	EU907678	FTOL v1.7
Dryopteridaceae	Elaphoglossum dimorphum (Hook. & Grev.) T. Moore	rps4-trnS	EU907753	FTOL v1.7
Dryopteridaceae	Elaphoglossum dimorphum (Hook. & Grev.) T. Moore	trnL-trnF	EU907817	FTOL v1.7
Dryopteridaceae	Elaphoglossum discolor (Kuhn) C. Chr.	rbcL	KJ628704	FTOL v1.7
Dryopteridaceae	Elaphoglossum doanense L. D. Gómez	rbcL	MW138165	FTOL v1.7
Dryopteridaceae	Elaphoglossum dussii Underw. ex Maxon	atpB	EU907681	FTOL v1.7
Dryopteridaceae	Elaphoglossum dussii Underw. ex Maxon	rps4-trnS	EU907755	FTOL v1.7
Dryopteridaceae	Elaphoglossum dussii Underw. ex Maxon	trnL-trnF	EU907819	FTOL v1.7
Dryopteridaceae	Elaphoglossum edwallii Rosenst.	rbcL	AY818689	FTOL v1.7
Dryopteridaceae	Elaphoglossum edwallii Rosenst.	rps4-trnS	AY536144	FTOL v1.7
Dryopteridaceae	Elaphoglossum edwallii Rosenst.	trnL-trnF	AY534816	FTOL v1.7
Dryopteridaceae	Elaphoglossum eggersii (Baker) Christ	atpB	KF212381	FTOL v1.7
Dryopteridaceae	Elaphoglossum eggersii (Baker) Christ	rps4-trnS	KF212431	FTOL v1.7
Dryopteridaceae	Elaphoglossum eggersii (Baker) Christ	trnL-trnF	KF212406	FTOL v1.7
Dryopteridaceae	Elaphoglossum engelii (H. Karst.) Christ	atpB	KJ528065	FTOL v1.7
Dryopteridaceae	Elaphoglossum engelii (H. Karst.) Christ	rps4-trnS	KJ528162	FTOL v1.7
Dryopteridaceae	Elaphoglossum engelii (H. Karst.) Christ	trnL-trnF	KJ528183	FTOL v1.7
Dryopteridaceae	Elaphoglossum erinaceum (Fée) T. Moore	atpA	EF463688	FTOL v1.7
Dryopteridaceae	Elaphoglossum erinaceum (Fée) T. Moore	atpB	EF463404	FTOL v1.7
Dryopteridaceae	Elaphoglossum erinaceum (Fée) T. Moore	rbcL	EF463191	FTOL v1.7
Dryopteridaceae	Elaphoglossum eximium (Mett.) Christ	atpB	MG600327	FTOL v1.7
Dryopteridaceae	Elaphoglossum eximium (Mett.) Christ	rbcL	KM495092	FTOL v1.7
Dryopteridaceae	Elaphoglossum eximium (Mett.) Christ	rps4-trnS	MG600356	FTOL v1.7
Dryopteridaceae	Elaphoglossum eximium (Mett.) Christ	trnL-trnF	MG600384	FTOL v1.7
Dryopteridaceae	Elaphoglossum feei (Bory ex Fée) T. Moore	atpB	KJ528072	FTOL v1.7
Dryopteridaceae	Elaphoglossum feei (Bory ex Fée) T. Moore	rps4-trnS	KJ528120	FTOL v1.7
Dryopteridaceae	Elaphoglossum feei (Bory ex Fée) T. Moore	trnL-trnF	KJ528184	FTOL v1.7
Dryopteridaceae	Elaphoglossum forsythii-majoris Christ	atpB	EF040644	FTOL v1.7
Dryopteridaceae	Elaphoglossum forsythii-majoris Christ	rps4-trnS	EF040620	FTOL v1.7
Dryopteridaceae	Elaphoglossum forsythii-majoris Christ	trnL-trnF	EF040606	FTOL v1.7
Dryopteridaceae	Elaphoglossum fournierianum L. D. Gómez	rps4-trnS	AY540248	FTOL v1.7
Dryopteridaceae	Elaphoglossum fournierianum L. D. Gómez	trnL-trnF	AY536311	FTOL v1.7

Dryopteridaceae	Elaphoglossum furfuraceum (Mett. ex Kuhn) Christ	rps4-trnS	AY540249	FTOL v1.7
Dryopteridaceae	Elaphoglossum furfuraceum (Mett. ex Kuhn) Christ	trnL-trnF	AY536312	FTOL v1.7
Dryopteridaceae	Elaphoglossum gayanum (Fée) T. Moore	atpB	MG600318	FTOL v1.7
Dryopteridaceae	Elaphoglossum gayanum (Fée) T. Moore	rps4-trnS	MG600348	FTOL v1.7
Dryopteridaceae	Elaphoglossum gayanum (Fée) T. Moore	trnL-trnF	MG600376	FTOL v1.7
Dryopteridaceae	Elaphoglossum glabellum J. Sm.	rbcL	AY818691	FTOL v1.7
Dryopteridaceae	Elaphoglossum glaucum T. Moore	rbcL	AY818692	FTOL v1.7
Dryopteridaceae	Elaphoglossum glaucum T. Moore	rps4-trnS	AY536172	FTOL v1.7
Dryopteridaceae	Elaphoglossum glaucum T. Moore	trnL-trnF	AY534844	FTOL v1.7
Dryopteridaceae	Elaphoglossum gomezianum A. Rojas	rbcL	KM495100	FTOL v1.7
Dryopteridaceae	Elaphoglossum gramineum (Jenman) Urb.	atpB	EU907682	FTOL v1.7
Dryopteridaceae	Elaphoglossum gramineum (Jenman) Urb.	rps4-trnS	EU907757	FTOL v1.7
Dryopteridaceae	Elaphoglossum gramineum (Jenman) Urb.	trnL-trnF	EU907820	FTOL v1.7
Dryopteridaceae	Elaphoglossum grayumii Mickel	rps4-trnS	AY540250	FTOL v1.7
Dryopteridaceae	Elaphoglossum grayumii Mickel	trnL-trnF	AY536313	FTOL v1.7
Dryopteridaceae	Elaphoglossum guatemalense (Klotzsch) T. Moore	rbcL	AY818693	FTOL v1.7
Dryopteridaceae	Elaphoglossum guatemalense (Klotzsch) T. Moore	rps4-trnS	AY536164	FTOL v1.7
Dryopteridaceae	Elaphoglossum guatemalense (Klotzsch) T. Moore	trnL-trnF	AY534836	FTOL v1.7
Dryopteridaceae	Elaphoglossum guentheri Rosenst.	atpB	KJ528048	FTOL v1.7
Dryopteridaceae	Elaphoglossum guentheri Rosenst.	rps4-trnS	KJ528159	FTOL v1.7
Dryopteridaceae	Elaphoglossum guentheri Rosenst.	trnL-trnF	KJ528189	FTOL v1.7
Dryopteridaceae	Elaphoglossum heterochroum Mickel	rbcL	MW138228	FTOL v1.7
Dryopteridaceae	Elaphoglossum heterolepis (Fée) T. Moore	atpA	EF463691	FTOL v1.7
Dryopteridaceae	Elaphoglossum heterolepis (Fée) T. Moore	atpB	EF463407	FTOL v1.7
Dryopteridaceae	Elaphoglossum heterolepis (Fée) T. Moore	rbcL	EF463194	FTOL v1.7
Dryopteridaceae	Elaphoglossum hirtum (Sw.) C. Chr.	trnL-trnF	AY194071	FTOL v1.7
Dryopteridaceae	Elaphoglossum hoffmannii (Mett. ex Kuhn) Christ	atpB	KF212382	FTOL v1.7
Dryopteridaceae	Elaphoglossum hoffmannii (Mett. ex Kuhn) Christ	rps4-trnS	KF212433	FTOL v1.7
Dryopteridaceae	Elaphoglossum hoffmannii (Mett. ex Kuhn) Christ	trnL-trnF	KF212408	FTOL v1.7
Dryopteridaceae	Elaphoglossum hornei C. Chr.	rps4-trnS	AY540253	FTOL v1.7
Dryopteridaceae	Elaphoglossum hornei C. Chr.	trnL-trnF	AY536316	FTOL v1.7
Dryopteridaceae	Elaphoglossum huacsaro (Ruiz) Christ	atpA	EF463692	FTOL v1.7
Dryopteridaceae	Elaphoglossum huacsaro (Ruiz) Christ	atpB	EF463408	FTOL v1.7
Dryopteridaceae	Elaphoglossum huacsaro (Ruiz) Christ	rbcL	EF463195	FTOL v1.7
Dryopteridaceae	Elaphoglossum humbertii C. Chr.	atpB	EU907696	FTOL v1.7
Dryopteridaceae	Elaphoglossum humbertii C. Chr.	rps4-trnS	EU907771	FTOL v1.7
Dryopteridaceae	Elaphoglossum humbertii C. Chr.	trnL-trnF	EU907834	FTOL v1.7
Dryopteridaceae	Elaphoglossum hybridum (Bory) Brack.	atpA	EF463693	FTOL v1.7
Dryopteridaceae	Elaphoglossum hybridum (Bory) Brack.	atpB	EF463409	FTOL v1.7
Dryopteridaceae	Elaphoglossum hybridum (Bory) Brack.	rbcL	EF463196	FTOL v1.7
Dryopteridaceae	Elaphoglossum ipshookense Mickel, Mickel & Beitel	atpB	EU907698	FTOL v1.7
Dryopteridaceae	Elaphoglossum ipshookense Mickel, Mickel & Beitel	rps4-trnS	EU907773	FTOL v1.7
Dryopteridaceae	Elaphoglossum ipshookense Mickel, Mickel & Beitel	trnL-trnF	EU907836	FTOL v1.7
Dryopteridaceae	Elaphoglossum killipii Mickel	rps4-trnS	AY540257	FTOL v1.7
Dryopteridaceae	Elaphoglossum killipii Mickel	trnL-trnF	AY536320	FTOL v1.7
Dryopteridaceae	Elaphoglossum lalitae L. D. Gómez	atpB	MG600317	FTOL v1.7
Dryopteridaceae	Elaphoglossum lalitae L. D. Gómez	rps4-trnS	MG600347	FTOL v1.7
Dryopteridaceae	Elaphoglossum lalitae L. D. Gómez	trnL-trnF	MG600375	FTOL v1.7
Dryopteridaceae	Elaphoglossum lanatum (Bojer ex Baker) Lorence	rps4-trnS	AY540258	FTOL v1.7
Dryopteridaceae	Elaphoglossum lanatum (Bojer ex Baker) Lorence	trnL-trnF	AY536321	FTOL v1.7
Dryopteridaceae	Elaphoglossum lanceiforme Mickel	rbcL	MW138225	FTOL v1.7
Dryopteridaceae	Elaphoglossum lancifolium (Desv.) C. V. Morton	atpB	EF040640	FTOL v1.7
Dryopteridaceae	Elaphoglossum lancifolium (Desv.) C. V. Morton	rps4-trnS	EF040619	FTOL v1.7
Dryopteridaceae	Elaphoglossum lancifolium (Desv.) C. V. Morton	trnL-trnF	EF040605	FTOL v1.7
Dryopteridaceae	Elaphoglossum langsdorffii (Hook. & Grev.) T. Moore	atpB	MG600307	FTOL v1.7
Dryopteridaceae	Elaphoglossum langsdorffii (Hook. & Grev.) T. Moore	rps4-trnS	GU376683	FTOL v1.7
Dryopteridaceae	Elaphoglossum langsdorffii (Hook. & Grev.) T. Moore	trnL-trnF	GU376536	FTOL v1.7
Dryopteridaceae	Elaphoglossum lankesteri Mickel	rbcL	MW138130	FTOL v1.7
Dryopteridaceae	Elaphoglossum lastii (Baker) C. Chr.	trnL-trnF	AY194077	FTOL v1.7
Dryopteridaceae	Elaphoglossum latifolium (Sw.) J. Sm.	rbcL	MW138227	FTOL v1.7
Dryopteridaceae	Elaphoglossum lechlerianum (Mett.) T. Moore	atpA	EF463694	FTOL v1.7
Dryopteridaceae	Elaphoglossum lechlerianum (Mett.) T. Moore	atpB	EF463410	FTOL v1.7
Dryopteridaceae	Elaphoglossum lechlerianum (Mett.) T. Moore	rbcL	EF463197	FTOL v1.7
Dryopteridaceae	Elaphoglossum lehmannianum Christ	atpB	KJ528049	FTOL v1.7
Dryopteridaceae	Elaphoglossum lehmannianum Christ	rps4-trnS	KJ528163	FTOL v1.7
Dryopteridaceae	Elaphoglossum lehmannianum Christ	trnL-trnF	KJ528190	FTOL v1.7
Dryopteridaceae	Elaphoglossum lepervanchii (Bory ex Fée) T. Moore	rbcL	KF992526	FTOL v1.7
Dryopteridaceae	Elaphoglossum leucolepis (Baker) Krajina ex Tardieu	atpB	EF040638	FTOL v1.7
Dryopteridaceae	Elaphoglossum leucolepis (Baker) Krajina ex Tardieu	rps4-trnS	AY540261	FTOL v1.7
Dryopteridaceae	Elaphoglossum leucolepis (Baker) Krajina ex Tardieu	trnL-trnF	AY536324	FTOL v1.7
Dryopteridaceae	Elaphoglossum lherminieri (Bory ex Fée) T. Moore	atpA	EF463690	FTOL v1.7
Dryopteridaceae	Elaphoglossum lherminieri (Bory ex Fée) T. Moore	atpB	EF463406	FTOL v1.7
Dryopteridaceae	Elaphoglossum lherminieri (Bory ex Fée) T. Moore	rbcL	EF463193	FTOL v1.7
Dryopteridaceae	Elaphoglossum lindenii (Bory ex Fée) T. Moore	rbcL	AY818696	FTOL v1.7
Dryopteridaceae	Elaphoglossum lingua (Raddi) Brack.	rbcL	AY818697	FTOL v1.7
Dryopteridaceae	Elaphoglossum litanum (Sodiro) C. Chr.	atpB	MG600309	FTOL v1.7
Dryopteridaceae	Elaphoglossum litanum (Sodiro) C. Chr.	rps4-trnS	MG600338	FTOL v1.7
Dryopteridaceae	Elaphoglossum litanum (Sodiro) C. Chr.	trnL-trnF	MG600366	FTOL v1.7
Dryopteridaceae	Elaphoglossum lloense (Hook.) T. Moore	atpB	KJ528084	FTOL v1.7
Dryopteridaceae	Elaphoglossum lloense (Hook.) T. Moore	rps4-trnS	KJ528144	FTOL v1.7
Dryopteridaceae	Elaphoglossum lloense (Hook.) T. Moore	trnL-trnF	KJ528198	FTOL v1.7
Dryopteridaceae	Elaphoglossum lonchophyllum (Fée) T. Moore	rbcL	AY818698	FTOL v1.7
Dryopteridaceae	Elaphoglossum lonchophyllum (Fée) T. Moore	rps4-trnS	AY536136	FTOL v1.7
Dryopteridaceae	Elaphoglossum lonchophyllum (Fée) T. Moore	trnL-trnF	AY534807	FTOL v1.7
Dryopteridaceae	Elaphoglossum longicrure Christ	rbcL	MW138185	FTOL v1.7
Dryopteridaceae	Elaphoglossum longistipitatum A. Rojas	rbcL	KM583469	FTOL v1.7
Dryopteridaceae	Elaphoglossum luridum (Fée) Christ	rbcL	AY534859	FTOL v1.7
Dryopteridaceae	Elaphoglossum luzonicum var. mcclurei (Ching) F. G. W	plastome	MT130636	FTOL v1.7
Dryopteridaceae	Elaphoglossum macropodium (Fée) T. Moore	rbcL	KF992527	FTOL v1.7
Dryopteridaceae	Elaphoglossum marginatum (Wall. ex Fée) T. Moore	plastome	MT130569	FTOL v1.7
Dryopteridaceae	Elaphoglossum marojejyense Tardieu	atpB	EF040630	FTOL v1.7
Dryopteridaceae	Elaphoglossum marojejyense Tardieu	rps4-trnS	AY540266	FTOL v1.7
Dryopteridaceae	Elaphoglossum marojejyense Tardieu	trnL-trnF	AY536329	FTOL v1.7
Dryopteridaceae	Elaphoglossum marquisearum Bonap.	rbcL	MT216031	FTOL v1.7
Dryopteridaceae	Elaphoglossum marquisearum Bonap.	rps4-trnS	AY540267	FTOL v1.7
Dryopteridaceae	Elaphoglossum marquisearum Bonap.	trnL-trnF	AY536330	FTOL v1.7
Dryopteridaceae	Elaphoglossum martinicense (Desv.) T. Moore	atpB	KF212387	FTOL v1.7
Dryopteridaceae	Elaphoglossum martinicense (Desv.) T. Moore	rps4-trnS	KF212437	FTOL v1.7
Dryopteridaceae	Elaphoglossum martinicense (Desv.) T. Moore	trnL-trnF	KF212411	FTOL v1.7
Dryopteridaceae	Elaphoglossum maxonii Underw. ex Morton	atpB	KF212388	FTOL v1.7
Dryopteridaceae	Elaphoglossum maxonii Underw. ex Morton	rps4-trnS	KF212438	FTOL v1.7
Dryopteridaceae	Elaphoglossum maxonii Underw. ex Morton	trnL-trnF	KF212413	FTOL v1.7
Dryopteridaceae	Elaphoglossum mesoamericanum A. Rojas	rbcL	OP710928	FTOL v1.7
Dryopteridaceae	Elaphoglossum metallicum Mickel	rbcL	AY534858	FTOL v1.7
Dryopteridaceae	Elaphoglossum metallicum Mickel	rps4-trnS	AY536160	FTOL v1.7
Dryopteridaceae	Elaphoglossum metallicum Mickel	trnL-trnF	AY534832	FTOL v1.7
Dryopteridaceae	Elaphoglossum meyeri Rouhan	rps4-trnS	AY540287	FTOL v1.7
Dryopteridaceae	Elaphoglossum meyeri Rouhan	trnL-trnF	AY536350	FTOL v1.7
Dryopteridaceae	Elaphoglossum micradenium (Fée) T. Moore	rbcL	MT657684	FTOL v1.7
Dryopteridaceae	Elaphoglossum micropogon Mickel	atpB	EF040643	FTOL v1.7
Dryopteridaceae	Elaphoglossum micropogon Mickel	rps4-trnS	AY540268	FTOL v1.7
Dryopteridaceae	Elaphoglossum micropogon Mickel	trnL-trnF	AY536331	FTOL v1.7
Dryopteridaceae	Elaphoglossum miersii (Baker) C. Chr.	atpB	MG600323	FTOL v1.7
Dryopteridaceae	Elaphoglossum miersii (Baker) C. Chr.	rps4-trnS	MG600353	FTOL v1.7
Dryopteridaceae	Elaphoglossum miersii (Baker) C. Chr.	trnL-trnF	MG600381	FTOL v1.7
Dryopteridaceae	Elaphoglossum minutissimum R. C. Moran & Mickel	rps4-trnS	AY540293	FTOL v1.7
Dryopteridaceae	Elaphoglossum minutissimum R. C. Moran & Mickel	trnL-trnF	AY536356	FTOL v1.7
Dryopteridaceae	Elaphoglossum minutum (Pohl ex Fée) T. Moore	rbcL	AY818699	FTOL v1.7
Dryopteridaceae	Elaphoglossum mitorrhizum Mickel	rbcL	MW138226	FTOL v1.7
Dryopteridaceae	Elaphoglossum moorei (E. Britton) Christ	atpA	EF463698	FTOL v1.7
Dryopteridaceae	Elaphoglossum moorei (E. Britton) Christ	atpB	EF463414	FTOL v1.7
Dryopteridaceae	Elaphoglossum moorei (E. Britton) Christ	rbcL	EF463198	FTOL v1.7
Dryopteridaceae	Elaphoglossum moorei (E. Britton) Christ	rps4-trnS	KJ528154	FTOL v1.7
Dryopteridaceae	Elaphoglossum moorei (E. Britton) Christ	trnL-trnF	KJ528209	FTOL v1.7
Dryopteridaceae	Elaphoglossum moranii Mickel	rbcL	KM495101	FTOL v1.7
Dryopteridaceae	Elaphoglossum nervosum (Bory) Christ	atpB	EU907701	FTOL v1.7
Dryopteridaceae	Elaphoglossum nervosum (Bory) Christ	rps4-trnS	EU907775	FTOL v1.7
Dryopteridaceae	Elaphoglossum nervosum (Bory) Christ	trnL-trnF	EU907837	FTOL v1.7
Dryopteridaceae	Elaphoglossum nidiforme Mickel	atpB	EF040662	FTOL v1.7
Dryopteridaceae	Elaphoglossum nidiforme Mickel	rps4-trnS	EF040629	FTOL v1.7
Dryopteridaceae	Elaphoglossum nidiforme Mickel	trnL-trnF	EF040615	FTOL v1.7
Dryopteridaceae	Elaphoglossum nidusoides Rouhan & Rakotondr.	atpB	EF040634	FTOL v1.7
Dryopteridaceae	Elaphoglossum nidusoides Rouhan & Rakotondr.	rps4-trnS	EF040618	FTOL v1.7
Dryopteridaceae	Elaphoglossum nidusoides Rouhan & Rakotondr.	trnL-trnF	EF040604	FTOL v1.7
Dryopteridaceae	Elaphoglossum nigrescens (Hook.) T. Moore ex Diels	rbcL	AY818710	FTOL v1.7
Dryopteridaceae	Elaphoglossum notatum (Fée) T. Moore	atpB	KJ528069	FTOL v1.7
Dryopteridaceae	Elaphoglossum notatum (Fée) T. Moore	rps4-trnS	GU376678	FTOL v1.7
Dryopteridaceae	Elaphoglossum notatum (Fée) T. Moore	trnL-trnF	GU376532	FTOL v1.7
Dryopteridaceae	Elaphoglossum oblanceolatum C. Chr.	rbcL	KM583468	FTOL v1.7
Dryopteridaceae	Elaphoglossum obovatum Mickel	rbcL	KJ628708	FTOL v1.7
Dryopteridaceae	Elaphoglossum obscurum (E. Fourn.) C. Chr.	rbcL	MW620315	FTOL v1.7
Dryopteridaceae	Elaphoglossum ocoense C. Chr.	rps4-trnS	KF212441	FTOL v1.7
Dryopteridaceae	Elaphoglossum ocoense C. Chr.	trnL-trnF	KF212414	FTOL v1.7
Dryopteridaceae	Elaphoglossum orbignyanum (Fée) T. Moore	atpB	EU907710	FTOL v1.7
Dryopteridaceae	Elaphoglossum orbignyanum (Fée) T. Moore	rbcL	AY818701	FTOL v1.7
Dryopteridaceae	Elaphoglossum orbignyanum (Fée) T. Moore	rps4-trnS	EU907783	FTOL v1.7
Dryopteridaceae	Elaphoglossum orbignyanum (Fée) T. Moore	trnL-trnF	AY534819	FTOL v1.7
Dryopteridaceae	Elaphoglossum ovalilimbatum Bonap.	rps4-trnS	AY540272	FTOL v1.7
Dryopteridaceae	Elaphoglossum ovalilimbatum Bonap.	trnL-trnF	AY536335	FTOL v1.7
Dryopteridaceae	Elaphoglossum ovatum (Hook. & Grev.) T. Moore	atpB	EF040641	FTOL v1.7
Dryopteridaceae	Elaphoglossum ovatum (Hook. & Grev.) T. Moore	rbcL	AY818702	FTOL v1.7
Dryopteridaceae	Elaphoglossum ovatum (Hook. & Grev.) T. Moore	rps4-trnS	AY540273	FTOL v1.7
Dryopteridaceae	Elaphoglossum ovatum (Hook. & Grev.) T. Moore	trnL-trnF	AY536336	FTOL v1.7
Dryopteridaceae	Elaphoglossum paleaceum (Hook. & Grev.) Sledge	atpA	EF463699	FTOL v1.7
Dryopteridaceae	Elaphoglossum paleaceum (Hook. & Grev.) Sledge	atpB	EF463415	FTOL v1.7
Dryopteridaceae	Elaphoglossum paleaceum (Hook. & Grev.) Sledge	rbcL	EF463199	FTOL v1.7
Dryopteridaceae	Elaphoglossum palmeri Underw. & Maxon	atpB	KF212391	FTOL v1.7
Dryopteridaceae	Elaphoglossum palmeri Underw. & Maxon	rps4-trnS	KF212442	FTOL v1.7
Dryopteridaceae	Elaphoglossum palmeri Underw. & Maxon	trnL-trnF	KF212415	FTOL v1.7
Dryopteridaceae	Elaphoglossum papillosum (Baker) Christ	atpA	EF463700	FTOL v1.7
Dryopteridaceae	Elaphoglossum papillosum (Baker) Christ	atpB	EF463416	FTOL v1.7
Dryopteridaceae	Elaphoglossum papillosum (Baker) Christ	rbcL	EF463200	FTOL v1.7
Dryopteridaceae	Elaphoglossum papillosum (Baker) Christ	rps4-trnS	AY536129	FTOL v1.7
Dryopteridaceae	Elaphoglossum papillosum (Baker) Christ	trnL-trnF	AY534800	FTOL v1.7
Dryopteridaceae	Elaphoglossum parvisquameum Skottsb.	rbcL	MT657687	FTOL v1.7
Dryopteridaceae	Elaphoglossum peltatum (Sw.) Urb.	atpA	EF463701	FTOL v1.7
Dryopteridaceae	Elaphoglossum peltatum (Sw.) Urb.	atpB	EF463417	FTOL v1.7
Dryopteridaceae	Elaphoglossum peltatum (Sw.) Urb.	rbcL	EF463201	FTOL v1.7
Dryopteridaceae	Elaphoglossum pendulum A. Rojas	rps4-trnS	MG600355	FTOL v1.7
Dryopteridaceae	Elaphoglossum pendulum A. Rojas	trnL-trnF	MG600383	FTOL v1.7
Dryopteridaceae	Elaphoglossum petiolatum (Sw.) Urb.	rbcL	MW620390	FTOL v1.7
Dryopteridaceae	Elaphoglossum petiolosum (Desv.) T. Moore	atpB	KJ528052	FTOL v1.7
Dryopteridaceae	Elaphoglossum petiolosum (Desv.) T. Moore	rps4-trnS	KJ528160	FTOL v1.7
Dryopteridaceae	Elaphoglossum petiolosum (Desv.) T. Moore	trnL-trnF	KJ528230	FTOL v1.7
Dryopteridaceae	Elaphoglossum phanerophlebium C. Chr.	atpB	EF040646	FTOL v1.7
Dryopteridaceae	Elaphoglossum phanerophlebium C. Chr.	rps4-trnS	AY540276	FTOL v1.7
Dryopteridaceae	Elaphoglossum phanerophlebium C. Chr.	trnL-trnF	AY536339	FTOL v1.7
Dryopteridaceae	Elaphoglossum phoras Mickel	rbcL	MW138198	FTOL v1.7
Dryopteridaceae	Elaphoglossum piloselloides (C. Presl) T. Moore	atpA	EF463702	FTOL v1.7
Dryopteridaceae	Elaphoglossum piloselloides (C. Presl) T. Moore	atpB	EF463418	FTOL v1.7
Dryopteridaceae	Elaphoglossum piloselloides (C. Presl) T. Moore	rbcL	EF463202	FTOL v1.7
Dryopteridaceae	Elaphoglossum pilosius Mickel	atpB	MG600330	FTOL v1.7
Dryopteridaceae	Elaphoglossum pilosius Mickel	rps4-trnS	MG600359	FTOL v1.7
Dryopteridaceae	Elaphoglossum pilosius Mickel	trnL-trnF	MG600387	FTOL v1.7
Dryopteridaceae	Elaphoglossum plumosum (Fée) T. Moore	rbcL	KJ628709	FTOL v1.7
Dryopteridaceae	Elaphoglossum poolii (Baker) Christ	atpB	EF040639	FTOL v1.7
Dryopteridaceae	Elaphoglossum poolii (Baker) Christ	rps4-trnS	AY540278	FTOL v1.7

Dryopteridaceae	Elaphoglossum poolii (Baker) Christ	trnL-trnF	AY536341	FTOL v1.7
Dryopteridaceae	Elaphoglossum potosianum Christ	rbcL	MW620297	FTOL v1.7
Dryopteridaceae	Elaphoglossum pringlei (Davenp.) C. Chr.	rbcL	AY534855	FTOL v1.7
Dryopteridaceae	Elaphoglossum procurrens (Mett.) T. Moore	atpB	MG600311	FTOL v1.7
Dryopteridaceae	Elaphoglossum procurrens (Mett.) T. Moore	rps4-trnS	MG600340	FTOL v1.7
Dryopteridaceae	Elaphoglossum procurrens (Mett.) T. Moore	trnL-trnF	MG600368	FTOL v1.7
Dryopteridaceae	Elaphoglossum productum Rosenst.	rps4-trnS	AY540279	FTOL v1.7
Dryopteridaceae	Elaphoglossum productum Rosenst.	trnL-trnF	AY536342	FTOL v1.7
Dryopteridaceae	Elaphoglossum pteropus C. Chr.	rps4-trnS	AY540280	FTOL v1.7
Dryopteridaceae	Elaphoglossum pteropus C. Chr.	trnL-trnF	AY536343	FTOL v1.7
Dryopteridaceae	Elaphoglossum pusillum (Mett. ex Kuhn) C. Chr.	atpB	KF212394	FTOL v1.7
Dryopteridaceae	Elaphoglossum pusillum (Mett. ex Kuhn) C. Chr.	rps4-trnS	HG428762	FTOL v1.7
Dryopteridaceae	Elaphoglossum pusillum (Mett. ex Kuhn) C. Chr.	trnL-trnF	KF212420	FTOL v1.7
Dryopteridaceae	Elaphoglossum pygmaeum (Mett.) Christ	rps4-trnS	AY540281	FTOL v1.7
Dryopteridaceae	Elaphoglossum pygmaeum (Mett.) Christ	trnL-trnF	AY536344	FTOL v1.7
Dryopteridaceae	Elaphoglossum randii Alston & Schelpe	rps4-trnS	AY540282	FTOL v1.7
Dryopteridaceae	Elaphoglossum randii Alston & Schelpe	trnL-trnF	AY536345	FTOL v1.7
Dryopteridaceae	Elaphoglossum rapense Copel.	rps4-trnS	AY540283	FTOL v1.7
Dryopteridaceae	Elaphoglossum rapense Copel.	trnL-trnF	AY536346	FTOL v1.7
Dryopteridaceae	Elaphoglossum raywaense (Jenman) Alston	rps4	MN781373	FTOL v1.7
Dryopteridaceae	Elaphoglossum raywaense (Jenman) Alston	rps4-trnS	MN781373	FTOL v1.7
Dryopteridaceae	Elaphoglossum rhomboideum A. Vasco, Mickel & R. C.	rps4-trnS	KJ528137	FTOL v1.7
Dryopteridaceae	Elaphoglossum rhomboideum A. Vasco, Mickel & R. C.	trnL-trnF	KJ528231	FTOL v1.7
Dryopteridaceae	Elaphoglossum richardii (Bory) Christ	rbcL	KF992486	FTOL v1.7
Dryopteridaceae	Elaphoglossum rigidum (Aubl.) Urb.	atpA	EF463689	FTOL v1.7
Dryopteridaceae	Elaphoglossum rigidum (Aubl.) Urb.	atpB	EF463405	FTOL v1.7
Dryopteridaceae	Elaphoglossum rigidum (Aubl.) Urb.	rbcL	EF463192	FTOL v1.7
Dryopteridaceae	Elaphoglossum rufidulum (Willd.) C. Chr.	rbcL	KF992528	FTOL v1.7
Dryopteridaceae	Elaphoglossum russelliae Mickel	rps4-trnS	AY540286	FTOL v1.7
Dryopteridaceae	Elaphoglossum russelliae Mickel	trnL-trnF	AY536349	FTOL v1.7
Dryopteridaceae	Elaphoglossum rzedowskii Mickel	atpB	EU907718	FTOL v1.7
Dryopteridaceae	Elaphoglossum rzedowskii Mickel	rps4-trnS	EU907788	FTOL v1.7
Dryopteridaceae	Elaphoglossum rzedowskii Mickel	trnL-trnF	EU907851	FTOL v1.7
Dryopteridaceae	Elaphoglossum samoense Brack.	atpA	EF463703	FTOL v1.7
Dryopteridaceae	Elaphoglossum samoense Brack.	atpB	EF463419	FTOL v1.7
Dryopteridaceae	Elaphoglossum samoense Brack.	rbcL	KY099800	FTOL v1.7
Dryopteridaceae	Elaphoglossum sartorii (Liebm.) Mickel, Mickel & Beite	rbcL	AY818705	FTOL v1.7
Dryopteridaceae	Elaphoglossum sartorii (Liebm.) Mickel, Mickel & Beite	rps4-trnS	AY536161	FTOL v1.7
Dryopteridaceae	Elaphoglossum sartorii (Liebm.) Mickel, Mickel & Beite	trnL-trnF	AY534833	FTOL v1.7
Dryopteridaceae	Elaphoglossum savaiense (Baker) Diels	rbcL	KY099801	FTOL v1.7
Dryopteridaceae	Elaphoglossum scolopendrifolium (Raddi) J. Sm.	rbcL	AY818704	FTOL v1.7
Dryopteridaceae	Elaphoglossum scolopendrifolium (Raddi) J. Sm.	rps4-trnS	AY536139	FTOL v1.7
Dryopteridaceae	Elaphoglossum scolopendrifolium (Raddi) J. Sm.	trnL-trnF	AY534810	FTOL v1.7
Dryopteridaceae	Elaphoglossum scolopendriforme Tardieu	rps4-trnS	AY540288	FTOL v1.7
Dryopteridaceae	Elaphoglossum scolopendriforme Tardieu	trnL-trnF	AY536351	FTOL v1.7
Dryopteridaceae	Elaphoglossum setigerum (Sodiro) Diels	rps4-trnS	AY540289	FTOL v1.7
Dryopteridaceae	Elaphoglossum setigerum (Sodiro) Diels	trnL-trnF	AY536352	FTOL v1.7
Dryopteridaceae	Elaphoglossum sieberi (Hook. & Grev.) T. Moore	rps4-trnS	AY540290	FTOL v1.7
Dryopteridaceae	Elaphoglossum sieberi (Hook. & Grev.) T. Moore	trnL-trnF	AY536353	FTOL v1.7
Dryopteridaceae	Elaphoglossum siliquoides (Jenman) C. Chr.	rbcL	AY818706	FTOL v1.7
Dryopteridaceae	Elaphoglossum smithii (Baker) Christ	rbcL	KM495095	FTOL v1.7
Dryopteridaceae	Elaphoglossum spatulatum (Bory) T. Moore	rbcL	KF992529	FTOL v1.7
Dryopteridaceae	Elaphoglossum splendens (Bory ex Willd.) Brack.	rps4-trnS	AY540296	FTOL v1.7
Dryopteridaceae	Elaphoglossum splendens (Bory ex Willd.) Brack.	trnL-trnF	AY536359	FTOL v1.7
Dryopteridaceae	Elaphoglossum squamipes (Hook.) T. Moore	rbcL	AY818707	FTOL v1.7
Dryopteridaceae	Elaphoglossum squarrosum (Klotzsch) T. Moore	atpB	KJ528070	FTOL v1.7
Dryopteridaceae	Elaphoglossum squarrosum (Klotzsch) T. Moore	rps4-trnS	KJ528166	FTOL v1.7
Dryopteridaceae	Elaphoglossum squarrosum (Klotzsch) T. Moore	trnL-trnF	KJ528238	FTOL v1.7
Dryopteridaceae	Elaphoglossum stelligerum (Wall. ex Baker) T. Moore e	plastome	MT130690	FTOL v1.7
Dryopteridaceae	Elaphoglossum stipitatum (Bory) T. Moore	rbcL	KF992487	FTOL v1.7
Dryopteridaceae	Elaphoglossum styriacum Mickel	atpB	MN781290	FTOL v1.7
Dryopteridaceae	Elaphoglossum styriacum Mickel	rbcL	MN781339	FTOL v1.7
Dryopteridaceae	Elaphoglossum styriacum Mickel	rps4	MN781374	FTOL v1.7
Dryopteridaceae	Elaphoglossum styriacum Mickel	rps4-trnS	MN781374	FTOL v1.7
Dryopteridaceae	Elaphoglossum subcinnamomeum Hieron.	trnL-trnF	AY194073	FTOL v1.7
Dryopteridaceae	Elaphoglossum subsessile (Baker) C. Chr.	atpB	EF040660	FTOL v1.7
Dryopteridaceae	Elaphoglossum subsessile (Baker) C. Chr.	rps4-trnS	AY540298	FTOL v1.7
Dryopteridaceae	Elaphoglossum subsessile (Baker) C. Chr.	trnL-trnF	AY536361	FTOL v1.7
Dryopteridaceae	Elaphoglossum succisifolium (Thouars) T. Moore	atpB	KJ528047	FTOL v1.7
Dryopteridaceae	Elaphoglossum succisifolium (Thouars) T. Moore	rps4-trnS	AY540299	FTOL v1.7
Dryopteridaceae	Elaphoglossum succisifolium (Thouars) T. Moore	trnL-trnF	AY536362	FTOL v1.7
Dryopteridaceae	Elaphoglossum tectum (Humb. & Bonpl. ex Willd.) T. M	rbcL	AY534852	FTOL v1.7
Dryopteridaceae	Elaphoglossum tectum (Humb. & Bonpl. ex Willd.) T. M	rps4-trnS	AY536142	FTOL v1.7
Dryopteridaceae	Elaphoglossum tectum (Humb. & Bonpl. ex Willd.) T. M	trnL-trnF	AY534813	FTOL v1.7
Dryopteridaceae	Elaphoglossum tenuiculum (Fée) T. Moore ex C. Chr.	atpB	EU907722	FTOL v1.7
Dryopteridaceae	Elaphoglossum tenuiculum (Fée) T. Moore ex C. Chr.	trnL-trnF	EU907852	FTOL v1.7
Dryopteridaceae	Elaphoglossum tomentosum (Bory) Christ	rps4-trnS	AY540300	FTOL v1.7
Dryopteridaceae	Elaphoglossum tomentosum (Bory) Christ	trnL-trnF	AY536363	FTOL v1.7
Dryopteridaceae	Elaphoglossum tonduzii Christ	atpB	MG600312	FTOL v1.7
Dryopteridaceae	Elaphoglossum tonduzii Christ	rps4-trnS	MG600341	FTOL v1.7
Dryopteridaceae	Elaphoglossum tonduzii Christ	trnL-trnF	MG600370	FTOL v1.7
Dryopteridaceae	Elaphoglossum tosaense (Yatabe) Makino	rbcL	AB575181	FTOL v1.7
Dryopteridaceae	Elaphoglossum tovii E. D. Br.	rbcL	MT216032	FTOL v1.7
Dryopteridaceae	Elaphoglossum tripartitum (Hook. & Grev.) Mickel	atpA	EF463704	FTOL v1.7
Dryopteridaceae	Elaphoglossum tripartitum (Hook. & Grev.) Mickel	atpB	EF463420	FTOL v1.7
Dryopteridaceae	Elaphoglossum tripartitum (Hook. & Grev.) Mickel	rbcL	EF463204	FTOL v1.7
Dryopteridaceae	Elaphoglossum tripartitum (Hook. & Grev.) Mickel	rps4-trnS	KJ528148	FTOL v1.7
Dryopteridaceae	Elaphoglossum tripartitum (Hook. & Grev.) Mickel	trnL-trnF	KJ528240	FTOL v1.7
Dryopteridaceae	Elaphoglossum vagans (Mett.) Hieron.	atpB	MG600313	FTOL v1.7
Dryopteridaceae	Elaphoglossum vagans (Mett.) Hieron.	rps4-trnS	MG600343	FTOL v1.7
Dryopteridaceae	Elaphoglossum vagans (Mett.) Hieron.	trnL-trnF	MG600371	FTOL v1.7
Dryopteridaceae	Elaphoglossum vestitum (Schltdl. & Cham.) Schott	rbcL	AY534853	FTOL v1.7
Dryopteridaceae	Elaphoglossum vieillardii (Mett.) T. Moore	rps4-trnS	AY540301	FTOL v1.7
Dryopteridaceae	Elaphoglossum vieillardii (Mett.) T. Moore	trnL-trnF	AY536364	FTOL v1.7
Dryopteridaceae	Elaphoglossum vulcanicum Christ	atpB	EU907729	FTOL v1.7
Dryopteridaceae	Elaphoglossum vulcanicum Christ	rps4-trnS	EU907794	FTOL v1.7
Dryopteridaceae	Elaphoglossum vulcanicum Christ	trnL-trnF	EU907857	FTOL v1.7
Dryopteridaceae	Elaphoglossum wawrae (Luerss.) C. Chr.	rbcL	MT657685	FTOL v1.7
Dryopteridaceae	Elaphoglossum welwitschii (Baker) C. Chr.	rps4-trnS	AY540303	FTOL v1.7
Dryopteridaceae	Elaphoglossum welwitschii (Baker) C. Chr.	trnL-trnF	AY536366	FTOL v1.7
Dryopteridaceae	Elaphoglossum wrightii (Mett.) T. Moore	atpB	KJ528075	FTOL v1.7
Dryopteridaceae	Elaphoglossum wrightii (Mett.) T. Moore	rps4-trnS	KJ528086	FTOL v1.7
Dryopteridaceae	Elaphoglossum wrightii (Mett.) T. Moore	trnL-trnF	KJ528243	FTOL v1.7
Dryopteridaceae	Elaphoglossum xanthopodum Mickel	rbcL	MZ771316	FTOL v1.7
Dryopteridaceae	Elaphoglossum yoshinagae (Yatabe) Makino	plastome	ON898018	FTOL v1.7
Dryopteridaceae	Elaphoglossum yungense de la Sota	atpB	EU907733	FTOL v1.7
Dryopteridaceae	Elaphoglossum yungense de la Sota	rps4-trnS	EU907798	FTOL v1.7
Dryopteridaceae	Elaphoglossum yungense de la Sota	trnL-trnF	EU907861	FTOL v1.7
Dryopteridaceae	Lastreopsis abscondita Perrie & Amice	rbcL	MW183340	FTOL v1.7
Dryopteridaceae	Lastreopsis abscondita Perrie & Amice	trnL-trnF	MW183345	FTOL v1.7
Dryopteridaceae	Lastreopsis amplissima (C. Presl) Tindale	rbcL	KJ464434	FTOL v1.7
Dryopteridaceae	Lastreopsis amplissima (C. Presl) Tindale	rps4-trnS	KJ464676	FTOL v1.7
Dryopteridaceae	Lastreopsis amplissima (C. Presl) Tindale	trnL-trnF	KJ464606	FTOL v1.7
Dryopteridaceae	Lastreopsis davallioides (Brack.) Tindale	rbcL	KY886934	FTOL v1.7
Dryopteridaceae	Lastreopsis davallioides (Brack.) Tindale	trnL-trnF	MW183354	FTOL v1.7
Dryopteridaceae	Lastreopsis decomposita (R. Br.) Tindale	rbcL	KJ464439	FTOL v1.7
Dryopteridaceae	Lastreopsis decomposita (R. Br.) Tindale	rps4-trnS	KJ464684	FTOL v1.7
Dryopteridaceae	Lastreopsis hispida (Sw.) Tindale	atpA	EF463708	FTOL v1.7
Dryopteridaceae	Lastreopsis hispida (Sw.) Tindale	atpB	EF463424	FTOL v1.7
Dryopteridaceae	Lastreopsis hispida (Sw.) Tindale	rbcL	EF463207	FTOL v1.7
Dryopteridaceae	Lastreopsis hispida (Sw.) Tindale	rps4-trnS	KJ464692	FTOL v1.7
Dryopteridaceae	Lastreopsis hispida (Sw.) Tindale	trnL-trnF	KJ464615	FTOL v1.7
Dryopteridaceae	Lastreopsis killipii (C. Chr. & Maxon) Tindale	rbcL	KJ464448	FTOL v1.7
Dryopteridaceae	Lastreopsis killipii (C. Chr. & Maxon) Tindale	rps4-trnS	KJ464693	FTOL v1.7
Dryopteridaceae	Lastreopsis marginans (F. Muell.) D. A. Sm. & Tindale e	rbcL	KJ464450	FTOL v1.7
Dryopteridaceae	Lastreopsis marginans (F. Muell.) D. A. Sm. & Tindale e	rps4-trnS	KJ464695	FTOL v1.7
Dryopteridaceae	Lastreopsis marginans (F. Muell.) D. A. Sm. & Tindale e	trnL-trnF	KJ464616	FTOL v1.7
Dryopteridaceae	Lastreopsis poecilophlebia (Hook.) Labiak, Sundue & R.	atpA	KP164480	FTOL v1.7
Dryopteridaceae	Lastreopsis poecilophlebia (Hook.) Labiak, Sundue & R.	atpB	KP164487	FTOL v1.7
Dryopteridaceae	Lastreopsis poecilophlebia (Hook.) Labiak, Sundue & R.	rbcL	KJ464424	FTOL v1.7
Dryopteridaceae	Lastreopsis poecilophlebia (Hook.) Labiak, Sundue & R.	rps4-trnS	KJ464665	FTOL v1.7
Dryopteridaceae	Lastreopsis poecilophlebia (Hook.) Labiak, Sundue & R.	trnL-trnF	KJ464595	FTOL v1.7
Dryopteridaceae	Lastreopsis silvestris D. A. Sm. ex Tindale	rps4-trnS	KJ464713	FTOL v1.7
Dryopteridaceae	Lastreopsis subrecedens Ching	atpB	EF450531	FTOL v1.7
Dryopteridaceae	Lastreopsis subrecedens Ching	rbcL	EF460685	FTOL v1.7
Dryopteridaceae	Lastreopsis subrecedens Ching	trnL-trnF	KJ196646	FTOL v1.7
Dryopteridaceae	Lastreopsis subsericea (Mett.) Tindale	rbcL	KY886941	FTOL v1.7
Dryopteridaceae	Lastreopsis subsericea (Mett.) Tindale	rps4-trnS	KJ464715	FTOL v1.7
Dryopteridaceae	Lastreopsis subsericea (Mett.) Tindale	trnL-trnF	MW183351	FTOL v1.7
Dryopteridaceae	Lastreopsis tenera (R. Br.) Tindale	rbcL	KJ464467	FTOL v1.7
Dryopteridaceae	Lastreopsis tenera (R. Br.) Tindale	rps4-trnS	KJ464717	FTOL v1.7
Dryopteridaceae	Lastreopsis tenera (R. Br.) Tindale	trnL-trnF	KJ464636	FTOL v1.7
Dryopteridaceae	Lastreopsis tripinnata (F. Muell. ex Benth.) Labiak, Sun	atpA	KP164482	FTOL v1.7
Dryopteridaceae	Lastreopsis tripinnata (F. Muell. ex Benth.) Labiak, Sun	atpB	KP164489	FTOL v1.7
Dryopteridaceae	Lastreopsis tripinnata (F. Muell. ex Benth.) Labiak, Sun	rbcL	KJ464492	FTOL v1.7
Dryopteridaceae	Lastreopsis tripinnata (F. Muell. ex Benth.) Labiak, Sun	rps4-trnS	KJ464740	FTOL v1.7
Dryopteridaceae	Lastreopsis velutina (A. Rich.) Tindale	rbcL	KY886943	FTOL v1.7
Dryopteridaceae	Lastreopsis velutina (A. Rich.) Tindale	trnL-trnF	KY886965	FTOL v1.7
Dryopteridaceae	Lastreopsis vieillardii (Mett.) Tindale	rbcL	MW183341	FTOL v1.7
Dryopteridaceae	Lastreopsis vieillardii (Mett.) Tindale	trnL-trnF	MW183348	FTOL v1.7
Dryopteridaceae	Lastreopsis walleri Tindale	rbcL	KJ464472	FTOL v1.7
Dryopteridaceae	Lastreopsis walleri Tindale	rps4-trnS	KJ464723	FTOL v1.7
Dryopteridaceae	Lastreopsis wurunuran (Domin) Tindale	rbcL	KJ464475	FTOL v1.7
Dryopteridaceae	Lastreopsis wurunuran (Domin) Tindale	rps4-trnS	KJ464725	FTOL v1.7
Dryopteridaceae	Lomagramma brooksii Copel.	rps4-trnS	GU376691	FTOL v1.7
Dryopteridaceae	Lomagramma brooksii Copel.	trnL-trnF	GU376542	FTOL v1.7
Dryopteridaceae	Lomagramma cordipinna Holttum	rbcL	MT216056	FTOL v1.7
Dryopteridaceae	Lomagramma cordipinna Holttum	rps4-trnS	GU376692	FTOL v1.7
Dryopteridaceae	Lomagramma cordipinna Holttum	trnL-trnF	GU376545	FTOL v1.7
Dryopteridaceae	Lomagramma lomarioides (Blume) J. Sm.	rps4-trnS	GU376699	FTOL v1.7
Dryopteridaceae	Lomagramma lomarioides (Blume) J. Sm.	trnL-trnF	GU376550	FTOL v1.7
Dryopteridaceae	Lomagramma perakensis Bedd.	rps4-trnS	GU376703	FTOL v1.7
Dryopteridaceae	Lomagramma perakensis Bedd.	trnL-trnF	GU376553	FTOL v1.7
Dryopteridaceae	Lomagramma polyphylla Brack.	rbcL	MT657680	FTOL v1.7
Dryopteridaceae	Lomagramma pteroides J. Sm.	rps4-trnS	GU376704	FTOL v1.7
Dryopteridaceae	Lomagramma pteroides J. Sm.	trnL-trnF	GU376555	FTOL v1.7
Dryopteridaceae	Lomagramma sinuata C. Chr.	rps4-trnS	GU376705	FTOL v1.7
Dryopteridaceae	Lomagramma sinuata C. Chr.	trnL-trnF	GU376557	FTOL v1.7
Dryopteridaceae	Lomagramma sorbifolia (Willd.) Ching	plastome	OP298004	FTOL v1.7
Dryopteridaceae	Lomagramma sumatrana Alderw.	rps4-trnS	GU376708	FTOL v1.7
Dryopteridaceae	Lomagramma sumatrana Alderw.	trnL-trnF	GU376559	FTOL v1.7
Dryopteridaceae	Lomagramma tahitensis Holttum	rbcL	KY099821	FTOL v1.7
Dryopteridaceae	Maxonia apiifolia (Sw.) C. Chr.	atpA	EF463711	FTOL v1.7
Dryopteridaceae	Maxonia apiifolia (Sw.) C. Chr.	atpB	EF463427	FTOL v1.7
Dryopteridaceae	Maxonia apiifolia (Sw.) C. Chr.	rbcL	EF463209	FTOL v1.7
Dryopteridaceae	Maxonia apiifolia (Sw.) C. Chr.	rps4	KJ628941	FTOL v1.7
Dryopteridaceae	Maxonia apiifolia (Sw.) C. Chr.	rps4-trnS	KJ628941	FTOL v1.7
Dryopteridaceae	Maxonia apiifolia (Sw.) C. Chr.	trnL-trnF	KJ464641	FTOL v1.7
Dryopteridaceae	Megalastrum abundans (Rosenst.) A. R. Sm. & R. C. M	rbcL	KJ464478	FTOL v1.7
Dryopteridaceae	Megalastrum abundans (Rosenst.) A. R. Sm. & R. C. M	rps4-trnS	KJ464729	FTOL v1.7
Dryopteridaceae	Megalastrum abundans (Rosenst.) A. R. Sm. & R. C. M	trnL-trnF	KJ464642	FTOL v1.7
Dryopteridaceae	Megalastrum apicale R. C. Moran & J. Prado	rbcL	MW138204	FTOL v1.7
Dryopteridaceae	Megalastrum atrogriseum (C. Chr.) A. R. Sm. & R. C. M	rbcL	KJ464479	FTOL v1.7
Dryopteridaceae	Megalastrum atrogriseum (C. Chr.) A. R. Sm. & R. C. M	rps4-trnS	KJ464730	FTOL v1.7
Dryopteridaceae	Megalastrum atrogriseum (C. Chr.) A. R. Sm. & R. C. M	trnL-trnF	KJ464643	FTOL v1.7
Dryopteridaceae	Megalastrum biseriale (Baker) A. R. Sm. & R. C. Morar	atpA	EF463712	FTOL v1.7
Dryopteridaceae	Megalastrum biseriale (Baker) A. R. Sm. & R. C. Morar	atpB	EF463428	FTOL v1.7

Dryopteridaceae	Megalastrum biseriale (Baker) A. R. Sm. & R. C. Moran	rbcL	EF463210	FTOL v1.7
Dryopteridaceae	Megalastrum connexum (Kaulf.) A. R. Sm. & R. C. Mora	rbcL	KJ464481	FTOL v1.7
Dryopteridaceae	Megalastrum connexum (Kaulf.) A. R. Sm. & R. C. Mora	rps4-trnS	KJ464732	FTOL v1.7
Dryopteridaceae	Megalastrum connexum (Kaulf.) A. R. Sm. & R. C. Mora	trnL-trnF	KJ464645	FTOL v1.7
Dryopteridaceae	Megalastrum fugaceum R. C. Moran, J. Prado & Sundu	rbcL	KJ464482	FTOL v1.7
Dryopteridaceae	Megalastrum fugaceum R. C. Moran, J. Prado & Sundu	rps4-trnS	KJ464733	FTOL v1.7
Dryopteridaceae	Megalastrum fugaceum R. C. Moran, J. Prado & Sundu	trnL-trnF	KJ464646	FTOL v1.7
Dryopteridaceae	Megalastrum lanatum (Fée) Holttum	rbcL	KJ464483	FTOL v1.7
Dryopteridaceae	Megalastrum lanatum (Fée) Holttum	rps4-trnS	KJ464734	FTOL v1.7
Dryopteridaceae	Megalastrum lanatum (Fée) Holttum	trnL-trnF	KJ464647	FTOL v1.7
Dryopteridaceae	Megalastrum lanuginosum (Kaulf.) Holttum	rbcL	KF992531	FTOL v1.7
Dryopteridaceae	Megalastrum littorale R. C. Moran, J. Prado & Labiak	rps4-trnS	GU376710	FTOL v1.7
Dryopteridaceae	Megalastrum littorale R. C. Moran, J. Prado & Labiak	trnL-trnF	GU376561	FTOL v1.7
Dryopteridaceae	Megalastrum longipilosum A. Rojas	rbcL	MW138268	FTOL v1.7
Dryopteridaceae	Megalastrum lunense (Christ) A. R. Sm. & R. C. Moran	rbcL	MW138166	FTOL v1.7
Dryopteridaceae	Megalastrum macrotheca (Fée) A. R. Sm. & R. C. Mora	atpA	EF463713	FTOL v1.7
Dryopteridaceae	Megalastrum macrotheca (Fée) A. R. Sm. & R. C. Mora	atpB	EF463429	FTOL v1.7
Dryopteridaceae	Megalastrum macrotheca (Fée) A. R. Sm. & R. C. Mora	rbcL	EF463211	FTOL v1.7
Dryopteridaceae	Megalastrum macrotheca (Fée) A. R. Sm. & R. C. Mora	rps4-trnS	KJ464735	FTOL v1.7
Dryopteridaceae	Megalastrum macrotheca (Fée) A. R. Sm. & R. C. Mora	trnL-trnF	KJ464648	FTOL v1.7
Dryopteridaceae	Megalastrum oppositum (Kaulf. ex Spreng.) Li Bing Zha	rbcL	KJ464480	FTOL v1.7
Dryopteridaceae	Megalastrum oppositum (Kaulf. ex Spreng.) Li Bing Zha	rps4-trnS	KJ464731	FTOL v1.7
Dryopteridaceae	Megalastrum oppositum (Kaulf. ex Spreng.) Li Bing Zha	trnL-trnF	KJ464644	FTOL v1.7
Dryopteridaceae	Megalastrum retrorsum R. C. Moran, J. Prado & Labiak	rbcL	KJ464485	FTOL v1.7
Dryopteridaceae	Megalastrum retrorsum R. C. Moran, J. Prado & Labiak	rps4-trnS	GU376711	FTOL v1.7
Dryopteridaceae	Megalastrum retrorsum R. C. Moran, J. Prado & Labiak	trnL-trnF	GU376562	FTOL v1.7
Dryopteridaceae	Megalastrum subincisum (Willd.) A. R. Sm. & R. C. Mo	atpA	EF463714	FTOL v1.7
Dryopteridaceae	Megalastrum subincisum (Willd.) A. R. Sm. & R. C. Mo	atpB	EF463430	FTOL v1.7
Dryopteridaceae	Megalastrum subincisum (Willd.) A. R. Sm. & R. C. Mo	rbcL	EF463212	FTOL v1.7
Dryopteridaceae	Megalastrum subtile R. C. Moran, J. Prado & Sundue	rbcL	KJ464486	FTOL v1.7
Dryopteridaceae	Megalastrum subtile R. C. Moran, J. Prado & Sundue	rps4-trnS	KJ464737	FTOL v1.7
Dryopteridaceae	Megalastrum subtile R. C. Moran, J. Prado & Sundue	trnL-trnF	KJ464650	FTOL v1.7
Dryopteridaceae	Megalastrum vastum (Kunze) A. R. Sm. & R. C. Moran	rbcL	KJ464487	FTOL v1.7
Dryopteridaceae	Megalastrum vastum (Kunze) A. R. Sm. & R. C. Moran	rps4-trnS	KJ464738	FTOL v1.7
Dryopteridaceae	Megalastrum vastum (Kunze) A. R. Sm. & R. C. Moran	trnL-trnF	KJ464651	FTOL v1.7
Dryopteridaceae	Mickelia bernoullii (Kuhn ex Christ) R. C. Moran, Labiak	rbcL	ON820457	FTOL v1.7
Dryopteridaceae	Mickelia bernoullii (Kuhn ex Christ) R. C. Moran, Labiak	rps4	ON820346	FTOL v1.7
Dryopteridaceae	Mickelia bernoullii (Kuhn ex Christ) R. C. Moran, Labiak	rps4-trnS	ON820346	FTOL v1.7
Dryopteridaceae	Mickelia bernoullii (Kuhn ex Christ) R. C. Moran, Labiak	trnL-trnF	ON820238	FTOL v1.7
Dryopteridaceae	Mickelia guianensis (Aubl.) R. C. Moran, Labiak & Sund	atpA	EF463710	FTOL v1.7
Dryopteridaceae	Mickelia guianensis (Aubl.) R. C. Moran, Labiak & Sund	atpB	EF463426	FTOL v1.7
Dryopteridaceae	Mickelia guianensis (Aubl.) R. C. Moran, Labiak & Sund	rbcL	EF463208	FTOL v1.7
Dryopteridaceae	Mickelia hemiotis (Maxon) R. C. Moran, Labiak & Sund	rps4-trnS	GU376658	FTOL v1.7
Dryopteridaceae	Mickelia hemiotis (Maxon) R. C. Moran, Labiak & Sund	trnL-trnF	GU376512	FTOL v1.7
Dryopteridaceae	Mickelia nicotianifolia (Sw.) R. C. Moran, Labiak & Sun	atpA	EF463666	FTOL v1.7
Dryopteridaceae	Mickelia nicotianifolia (Sw.) R. C. Moran, Labiak & Sun	atpB	EF463382	FTOL v1.7
Dryopteridaceae	Mickelia nicotianifolia (Sw.) R. C. Moran, Labiak & Sun	rbcL	EF463171	FTOL v1.7
Dryopteridaceae	Mickelia oligarchica (Baker) R. C. Moran, Labiak & Sun	rbcL	MW138214	FTOL v1.7
Dryopteridaceae	Mickelia oligarchica (Baker) R. C. Moran, Labiak & Sun	rps4-trnS	GU376667	FTOL v1.7
Dryopteridaceae	Mickelia oligarchica (Baker) R. C. Moran, Labiak & Sun	trnL-trnF	GU376521	FTOL v1.7
Dryopteridaceae	Mickelia pergamentacea (Maxon) R. C. Moran, Labiak &	rbcL	KJ464490	FTOL v1.7
Dryopteridaceae	Mickelia scandens (Raddi) R. C. Moran, Labiak & Sundu	rbcL	KJ464488	FTOL v1.7
Dryopteridaceae	Mickelia scandens (Raddi) R. C. Moran, Labiak & Sundu	rps4-trnS	GU376696	FTOL v1.7
Dryopteridaceae	Mickelia scandens (Raddi) R. C. Moran, Labiak & Sundu	trnL-trnF	GU376547	FTOL v1.7
Dryopteridaceae	Olfersia alata C. Sánchez & Caluff	rbcL	MN025361	FTOL v1.7
Dryopteridaceae	Olfersia alata C. Sánchez & Caluff	rps4-trnS	KT273018	FTOL v1.7
Dryopteridaceae	Olfersia alata C. Sánchez & Caluff	trnL-trnF	KT273168	FTOL v1.7
Dryopteridaceae	Olfersia cervina (L.) Kunze	atpA	EF463715	FTOL v1.7
Dryopteridaceae	Olfersia cervina (L.) Kunze	atpB	EF463431	FTOL v1.7
Dryopteridaceae	Olfersia cervina (L.) Kunze	rbcL	EF463213	FTOL v1.7
Dryopteridaceae	Olfersia macrostegia (Hook.) comb. ined.	rps4-trnS	KT273003	FTOL v1.7
Dryopteridaceae	Olfersia macrostegia (Hook.) comb. ined.	trnL-trnF	KT273158	FTOL v1.7
Dryopteridaceae	Olfersia ochropteroides (Baker) comb. ined.	rbcL	KT272933	FTOL v1.7
Dryopteridaceae	Olfersia ochropteroides (Baker) comb. ined.	rps4-trnS	KT273007	FTOL v1.7
Dryopteridaceae	Olfersia ochropteroides (Baker) comb. ined.	trnL-trnF	KT273159	FTOL v1.7
Dryopteridaceae	Parapolystichum acuminatum (Houlston) Labiak, Sundu	rbcL	KJ464430	FTOL v1.7
Dryopteridaceae	Parapolystichum acuminatum (Houlston) Labiak, Sundu	rps4-trnS	GU376688	FTOL v1.7
Dryopteridaceae	Parapolystichum acuminatum (Houlston) Labiak, Sundu	trnL-trnF	GU376540	FTOL v1.7
Dryopteridaceae	Parapolystichum acutum (Kuntze) Labiak, Sundue & R.	rps4-trnS	KJ464673	FTOL v1.7
Dryopteridaceae	Parapolystichum acutum (Kuntze) Labiak, Sundue & R.	trnL-trnF	KJ464603	FTOL v1.7
Dryopteridaceae	Parapolystichum barterianum (Hook.) Rouhan	rps4-trnS	KJ464677	FTOL v1.7
Dryopteridaceae	Parapolystichum boivinii (Baker) Rouhan	rbcL	KJ464435	FTOL v1.7
Dryopteridaceae	Parapolystichum boivinii (Baker) Rouhan	rps4-trnS	KJ464678	FTOL v1.7
Dryopteridaceae	Parapolystichum boivinii (Baker) Rouhan	trnL-trnF	KJ464607	FTOL v1.7
Dryopteridaceae	Parapolystichum calanthum (Endl.) J. J. S. Gardner & N	trnL-trnF	KY886955	FTOL v1.7
Dryopteridaceae	Parapolystichum confine (C. Chr.) Labiak, Sundue & R. (	rbcL	KJ464438	FTOL v1.7
Dryopteridaceae	Parapolystichum confine (C. Chr.) Labiak, Sundue & R. (	rps4-trnS	KJ464681	FTOL v1.7
Dryopteridaceae	Parapolystichum confine (C. Chr.) Labiak, Sundue & R. (	trnL-trnF	KJ464610	FTOL v1.7
Dryopteridaceae	Parapolystichum currorii (Mett. ex Kuhn) Rouhan	rps4-trnS	KJ464683	FTOL v1.7
Dryopteridaceae	Parapolystichum currorii (Mett. ex Kuhn) Rouhan	trnL-trnF	KJ464612	FTOL v1.7
Dryopteridaceae	Parapolystichum effusum var. divergens (Willd. ex Schl	rbcL	KJ464440	FTOL v1.7
Dryopteridaceae	Parapolystichum effusum var. divergens (Willd. ex Schl	rps4-trnS	KJ464685	FTOL v1.7
Dryopteridaceae	Parapolystichum excultum (Mett.) Labiak, Sundue & R.	rbcL	KJ464444	FTOL v1.7
Dryopteridaceae	Parapolystichum excultum (Mett.) Labiak, Sundue & R.	rps4-trnS	GU376690	FTOL v1.7
Dryopteridaceae	Parapolystichum excultum (Mett.) Labiak, Sundue & R.	trnL-trnF	GU376541	FTOL v1.7
Dryopteridaceae	Parapolystichum glabellum (A. Cunn.) Labiak, Sundue &	atpA	EF463707	FTOL v1.7
Dryopteridaceae	Parapolystichum glabellum (A. Cunn.) Labiak, Sundue &	atpB	EF463423	FTOL v1.7
Dryopteridaceae	Parapolystichum glabellum (A. Cunn.) Labiak, Sundue &	rbcL	EF463206	FTOL v1.7
Dryopteridaceae	Parapolystichum grayi (D. L. Jones) J. J. S. Gardner & N	rbcL	KY886929	FTOL v1.7
Dryopteridaceae	Parapolystichum grayi (D. L. Jones) J. J. S. Gardner & N	rps4-trnS	KY886946	FTOL v1.7
Dryopteridaceae	Parapolystichum kermadecense (Perrie & Brownsey) P	rbcL	KY886937	FTOL v1.7
Dryopteridaceae	Parapolystichum kermadecense (Perrie & Brownsey) P	trnL-trnF	KY886959	FTOL v1.7
Dryopteridaceae	Parapolystichum microsorum (Endl.) Labiak, Sundue &	rbcL	KJ464454	FTOL v1.7
Dryopteridaceae	Parapolystichum microsorum (Endl.) Labiak, Sundue &	rps4-trnS	KJ464699	FTOL v1.7
Dryopteridaceae	Parapolystichum microsorum (Endl.) Labiak, Sundue &	trnL-trnF	KJ464619	FTOL v1.7
Dryopteridaceae	Parapolystichum munitum (Mett.) Labiak, Sundue & R.	rps4-trnS	KJ464702	FTOL v1.7
Dryopteridaceae	Parapolystichum munitum (Mett.) Labiak, Sundue & R.	trnL-trnF	KJ464622	FTOL v1.7
Dryopteridaceae	Parapolystichum nigritianum (Baker) Rouhan	rps4-trnS	KJ464703	FTOL v1.7
Dryopteridaceae	Parapolystichum novoguineensis (Holttum) Sundue & T	rps4-trnS	KU295570	FTOL v1.7
Dryopteridaceae	Parapolystichum novoguineensis (Holttum) Sundue & T	trnL-trnF	KU295571	FTOL v1.7
Dryopteridaceae	Parapolystichum pacificum (Tindale) J. J. S. Gardner &	rbcL	KY886930	FTOL v1.7
Dryopteridaceae	Parapolystichum pacificum (Tindale) J. J. S. Gardner &	rps4-trnS	KY886947	FTOL v1.7
Dryopteridaceae	Parapolystichum pacificum (Tindale) J. J. S. Gardner &	trnL-trnF	KY886953	FTOL v1.7
Dryopteridaceae	Parapolystichum perrierianum (C. Chr.) Rouhan	rbcL	KJ464455	FTOL v1.7
Dryopteridaceae	Parapolystichum perrierianum (C. Chr.) Rouhan	rps4-trnS	KJ464704	FTOL v1.7
Dryopteridaceae	Parapolystichum perrierianum (C. Chr.) Rouhan	trnL-trnF	KJ464623	FTOL v1.7
Dryopteridaceae	Parapolystichum pseudoperrierianum (Tardieu) Rouhar	rbcL	KJ464457	FTOL v1.7
Dryopteridaceae	Parapolystichum pseudoperrierianum (Tardieu) Rouhar	rps4-trnS	KJ464706	FTOL v1.7
Dryopteridaceae	Parapolystichum pseudoperrierianum (Tardieu) Rouhar	trnL-trnF	KJ464625	FTOL v1.7
Dryopteridaceae	Parapolystichum rufescens (Blume) Labiak, Sundue & F	rbcL	KJ464462	FTOL v1.7
Dryopteridaceae	Parapolystichum rufescens (Blume) Labiak, Sundue & F	rps4-trnS	KJ464711	FTOL v1.7
Dryopteridaceae	Parapolystichum rufescens (Blume) Labiak, Sundue & F	trnL-trnF	KJ464630	FTOL v1.7
Dryopteridaceae	Parapolystichum smithianum (Tindale) Labiak, Sundue	rbcL	KJ464464	FTOL v1.7
Dryopteridaceae	Parapolystichum smithianum (Tindale) Labiak, Sundue	trnL-trnF	KJ464632	FTOL v1.7
Dryopteridaceae	Parapolystichum subsimile (Hook.) Rouhan	rps4-trnS	KJ464716	FTOL v1.7
Dryopteridaceae	Parapolystichum tinarooense (Tindale) Labiak, Sundue	rbcL	KJ464469	FTOL v1.7
Dryopteridaceae	Parapolystichum tinarooense (Tindale) Labiak, Sundue	rps4-trnS	KJ464720	FTOL v1.7
Dryopteridaceae	Parapolystichum tinarooense (Tindale) Labiak, Sundue	trnL-trnF	KJ464638	FTOL v1.7
Dryopteridaceae	Parapolystichum vogelii (Hook.) Rouhan	rbcL	KJ464470	FTOL v1.7
Dryopteridaceae	Parapolystichum vogelii (Hook.) Rouhan	rps4-trnS	KJ464721	FTOL v1.7
Dryopteridaceae	Parapolystichum windsorensis (D.L.Jones & B.Gray) Lal	rbcL	KJ464473	FTOL v1.7
Dryopteridaceae	Parapolystichum windsorensis (D.L.Jones & B.Gray) Lal	rps4-trnS	KJ464724	FTOL v1.7
Dryopteridaceae	Parapolystichum windsorensis (D.L.Jones & B.Gray) Lal	trnL-trnF	KJ464639	FTOL v1.7
Dryopteridaceae	Phanerophlebia gastonyi Yatsk.	rbcL	OP711699	FTOL v1.7
Dryopteridaceae	Phanerophlebia juglandifolia (Humb. & Bonpl. ex Willd	trnL-trnF	DQ514510	FTOL v1.7
Dryopteridaceae	Phanerophlebia nobilis (Schltdl. & Cham.) C. Presl	atpA	EF463716	FTOL v1.7
Dryopteridaceae	Phanerophlebia nobilis (Schltdl. & Cham.) C. Presl	atpB	EF463432	FTOL v1.7
Dryopteridaceae	Phanerophlebia nobilis (Schltdl. & Cham.) C. Presl	rbcL	AF537231	FTOL v1.7
Dryopteridaceae	Phanerophlebia remotispora E. Fourn.	rbcL	MW620302	FTOL v1.7
Dryopteridaceae	Phanerophlebia umbonata Underw.	rbcL	AF537233	FTOL v1.7
Dryopteridaceae	Phanerophlebia umbonata Underw.	rps4	EU031783	FTOL v1.7
Dryopteridaceae	Phanerophlebia umbonata Underw.	rps4-trnS	EU031783	FTOL v1.7
Dryopteridaceae	Phanerophlebia umbonata Underw.	trnL-trnF	DQ514513	FTOL v1.7
Dryopteridaceae	Pleocnemia conjugata (Blume) C. Presl	trnL-trnF	KF709510	FTOL v1.7
Dryopteridaceae	Pleocnemia cumingiana C. Presl	atpB	KJ196506	FTOL v1.7
Dryopteridaceae	Pleocnemia cumingiana C. Presl	rbcL	KJ196828	FTOL v1.7
Dryopteridaceae	Pleocnemia cumingiana C. Presl	trnL-trnF	KJ196705	FTOL v1.7
Dryopteridaceae	Pleocnemia dahlii (Hieron.) Holttum	atpB	KJ196507	FTOL v1.7
Dryopteridaceae	Pleocnemia dahlii (Hieron.) Holttum	rbcL	KJ196829	FTOL v1.7
Dryopteridaceae	Pleocnemia dahlii (Hieron.) Holttum	trnL-trnF	KJ196706	FTOL v1.7
Dryopteridaceae	Pleocnemia hemiteliiformis (Racib.) Holttum	atpA	KF709455	FTOL v1.7
Dryopteridaceae	Pleocnemia hemiteliiformis (Racib.) Holttum	atpB	KF709465	FTOL v1.7
Dryopteridaceae	Pleocnemia hemiteliiformis (Racib.) Holttum	rbcL	KF709482	FTOL v1.7
Dryopteridaceae	Pleocnemia hemiteliiformis (Racib.) Holttum	trnL-trnF	KF709511	FTOL v1.7
Dryopteridaceae	Pleocnemia irregularis (C. Presl) Holttum	atpB	KJ196439	FTOL v1.7
Dryopteridaceae	Pleocnemia irregularis (C. Presl) Holttum	rbcL	KJ196856	FTOL v1.7
Dryopteridaceae	Pleocnemia irregularis (C. Presl) Holttum	trnL-trnF	KJ196659	FTOL v1.7
Dryopteridaceae	Pleocnemia leuzeana (Gaudich.) C. Presl	atpB	KJ196508	FTOL v1.7
Dryopteridaceae	Pleocnemia leuzeana (Gaudich.) C. Presl	rbcL	KJ196830	FTOL v1.7
Dryopteridaceae	Pleocnemia olivacea (Copel.) Holttum	rbcL	KJ464495	FTOL v1.7
Dryopteridaceae	Pleocnemia olivacea (Copel.) Holttum	rps4-trnS	KJ464743	FTOL v1.7
Dryopteridaceae	Pleocnemia presliana Holttum	atpB	KT831830	FTOL v1.7
Dryopteridaceae	Pleocnemia presliana Holttum	rbcL	KJ464496	FTOL v1.7
Dryopteridaceae	Pleocnemia presliana Holttum	rps4-trnS	KJ464744	FTOL v1.7
Dryopteridaceae	Pleocnemia submembranacea (Hayata) Tagawa & K. Iv	plastome	MT130681	FTOL v1.7
Dryopteridaceae	Polybotrya aequatoriana R. C. Moran	rbcL	KT272946	FTOL v1.7
Dryopteridaceae	Polybotrya aequatoriana R. C. Moran	rps4-trnS	KT273022	FTOL v1.7
Dryopteridaceae	Polybotrya alfredii Brade	atpA	EF463717	FTOL v1.7
Dryopteridaceae	Polybotrya alfredii Brade	atpB	EF463433	FTOL v1.7
Dryopteridaceae	Polybotrya alfredii Brade	rbcL	EF463215	FTOL v1.7
Dryopteridaceae	Polybotrya alfredii Brade	rps4-trnS	KJ464745	FTOL v1.7
Dryopteridaceae	Polybotrya alfredii Brade	trnL-trnF	KJ464653	FTOL v1.7
Dryopteridaceae	Polybotrya altescandens C. Chr.	rbcL	KT272949	FTOL v1.7
Dryopteridaceae	Polybotrya altescandens C. Chr.	rps4-trnS	KT273025	FTOL v1.7
Dryopteridaceae	Polybotrya altescandens C. Chr.	trnL-trnF	KT273174	FTOL v1.7
Dryopteridaceae	Polybotrya andina C. Chr.	rbcL	KJ464498	FTOL v1.7
Dryopteridaceae	Polybotrya andina C. Chr.	rps4-trnS	KJ464746	FTOL v1.7
Dryopteridaceae	Polybotrya andina C. Chr.	trnL-trnF	KJ464654	FTOL v1.7
Dryopteridaceae	Polybotrya appressa R. C. Moran	rbcL	KT272953	FTOL v1.7
Dryopteridaceae	Polybotrya appressa R. C. Moran	rps4-trnS	KT273029	FTOL v1.7
Dryopteridaceae	Polybotrya appressa R. C. Moran	trnL-trnF	KT273178	FTOL v1.7
Dryopteridaceae	Polybotrya caudata Kunze	rbcL	KT272957	FTOL v1.7
Dryopteridaceae	Polybotrya caudata Kunze	rps4-trnS	KT273034	FTOL v1.7
Dryopteridaceae	Polybotrya caudata Kunze	trnL-trnF	KT273183	FTOL v1.7
Dryopteridaceae	Polybotrya crassirhizoma Lellinger	rbcL	KT272958	FTOL v1.7
Dryopteridaceae	Polybotrya crassirhizoma Lellinger	rps4-trnS	KT273035	FTOL v1.7
Dryopteridaceae	Polybotrya crassirhizoma Lellinger	trnL-trnF	KT273184	FTOL v1.7
Dryopteridaceae	Polybotrya cylindrica Kaulf.	rbcL	KT272960	FTOL v1.7
Dryopteridaceae	Polybotrya cylindrica Kaulf.	rps4-trnS	KT273037	FTOL v1.7
Dryopteridaceae	Polybotrya cylindrica Kaulf.	trnL-trnF	KT273186	FTOL v1.7
Dryopteridaceae	Polybotrya espiritosantensis Brade	rbcL	KT272963	FTOL v1.7
Dryopteridaceae	Polybotrya espiritosantensis Brade	rps4-trnS	KT273040	FTOL v1.7
Dryopteridaceae	Polybotrya espiritosantensis Brade	trnL-trnF	KT273189	FTOL v1.7
Dryopteridaceae	Polybotrya fractiserialis (Baker) J. Sm.	atpB	KM114098	FTOL v1.7
Dryopteridaceae	Polybotrya fractiserialis (Baker) J. Sm.	rbcL	KT272965	FTOL v1.7
Dryopteridaceae	Polybotrya fractiserialis (Baker) J. Sm.	rps4-trnS	KT273042	FTOL v1.7
Dryopteridaceae	Polybotrya fractiserialis (Baker) J. Sm.	trnL-trnF	KT273191	FTOL v1.7

Dryopteridaceae	Polybotrya glandulosa Mett. ex Kuhn	rbcL	KJ628799	FTOL v1.7
Dryopteridaceae	Polybotrya gomezii R. C. Moran	rbcL	KT272966	FTOL v1.7
Dryopteridaceae	Polybotrya gomezii R. C. Moran	rps4-trnS	KT273043	FTOL v1.7
Dryopteridaceae	Polybotrya gomezii R. C. Moran	trnL-trnF	KT273192	FTOL v1.7
Dryopteridaceae	Polybotrya goyazensis Brade	rbcL	KT272969	FTOL v1.7
Dryopteridaceae	Polybotrya goyazensis Brade	rps4-trnS	KT273046	FTOL v1.7
Dryopteridaceae	Polybotrya goyazensis Brade	trnL-trnF	KT273193	FTOL v1.7
Dryopteridaceae	Polybotrya hickeyi R. C. Moran	rps4-trnS	KT273049	FTOL v1.7
Dryopteridaceae	Polybotrya insularis A. Rojas	rps4-trnS	KT273050	FTOL v1.7
Dryopteridaceae	Polybotrya insularis A. Rojas	trnL-trnF	KT273196	FTOL v1.7
Dryopteridaceae	Polybotrya lechleriana Mett.	rbcL	KT272972	FTOL v1.7
Dryopteridaceae	Polybotrya lechleriana Mett.	rps4-trnS	KT273051	FTOL v1.7
Dryopteridaceae	Polybotrya lechleriana Mett.	trnL-trnF	KT273197	FTOL v1.7
Dryopteridaceae	Polybotrya lourteigiana Lellinger	rps4-trnS	KT273054	FTOL v1.7
Dryopteridaceae	Polybotrya matosii Canestraro & Labiak	rbcL	KT272974	FTOL v1.7
Dryopteridaceae	Polybotrya matosii Canestraro & Labiak	rps4-trnS	KT273055	FTOL v1.7
Dryopteridaceae	Polybotrya matosii Canestraro & Labiak	trnL-trnF	KT273199	FTOL v1.7
Dryopteridaceae	Polybotrya osmundacea Humb. & Bonpl. ex Willd.	rbcL	KT272975	FTOL v1.7
Dryopteridaceae	Polybotrya osmundacea Humb. & Bonpl. ex Willd.	rps4-trnS	KT273056	FTOL v1.7
Dryopteridaceae	Polybotrya osmundacea Humb. & Bonpl. ex Willd.	trnL-trnF	KT273200	FTOL v1.7
Dryopteridaceae	Polybotrya pittieri Lellinger	rbcL	KT272979	FTOL v1.7
Dryopteridaceae	Polybotrya pittieri Lellinger	rps4-trnS	KT273060	FTOL v1.7
Dryopteridaceae	Polybotrya pittieri Lellinger	trnL-trnF	KT273204	FTOL v1.7
Dryopteridaceae	Polybotrya polybotryoides (Baker) Christ	rbcL	KT272980	FTOL v1.7
Dryopteridaceae	Polybotrya polybotryoides (Baker) Christ	rps4-trnS	KT273061	FTOL v1.7
Dryopteridaceae	Polybotrya polybotryoides (Baker) Christ	trnL-trnF	KT273205	FTOL v1.7
Dryopteridaceae	Polybotrya pubens Mart. ex Kunze	rbcL	KT272982	FTOL v1.7
Dryopteridaceae	Polybotrya pubens Mart. ex Kunze	rps4-trnS	KT273063	FTOL v1.7
Dryopteridaceae	Polybotrya pubens Mart. ex Kunze	trnL-trnF	KT273207	FTOL v1.7
Dryopteridaceae	Polybotrya semipinnata Fée	rbcL	KT272984	FTOL v1.7
Dryopteridaceae	Polybotrya semipinnata Fée	rps4-trnS	KT273065	FTOL v1.7
Dryopteridaceae	Polybotrya semipinnata Fée	trnL-trnF	KT273209	FTOL v1.7
Dryopteridaceae	Polybotrya sessilisora R. C. Moran	atpB	MN781239	FTOL v1.7
Dryopteridaceae	Polybotrya sessilisora R. C. Moran	rbcL	KT272988	FTOL v1.7
Dryopteridaceae	Polybotrya sessilisora R. C. Moran	rps4	KJ628945	FTOL v1.7
Dryopteridaceae	Polybotrya sessilisora R. C. Moran	rps4-trnS	KJ628945	FTOL v1.7
Dryopteridaceae	Polybotrya sessilisora R. C. Moran	trnL-trnF	KT273213	FTOL v1.7
Dryopteridaceae	Polybotrya sorbifolia Mett. ex Kuhn	rps4-trnS	KT273070	FTOL v1.7
Dryopteridaceae	Polybotrya sorbifolia Mett. ex Kuhn	trnL-trnF	KT273214	FTOL v1.7
Dryopteridaceae	Polybotrya speciosa Schott	rbcL	KT272989	FTOL v1.7
Dryopteridaceae	Polybotrya speciosa Schott	rps4-trnS	KT273072	FTOL v1.7
Dryopteridaceae	Polybotrya speciosa Schott	trnL-trnF	KT273216	FTOL v1.7
Dryopteridaceae	Polybotrya stolzei R. C. Moran	rbcL	KT272992	FTOL v1.7
Dryopteridaceae	Polybotrya stolzei R. C. Moran	rps4-trnS	KT273075	FTOL v1.7
Dryopteridaceae	Polybotrya suberecta (Baker) C. Chr.	rbcL	KT272993	FTOL v1.7
Dryopteridaceae	Polybotrya suberecta (Baker) C. Chr.	rps4-trnS	KT273076	FTOL v1.7
Dryopteridaceae	Polybotrya suberecta (Baker) C. Chr.	trnL-trnF	KT273219	FTOL v1.7
Dryopteridaceae	Polystichopsis chaerophylloides (Poir.) Morton	atpA	EF463718	FTOL v1.7
Dryopteridaceae	Polystichopsis chaerophylloides (Poir.) Morton	atpB	EF463434	FTOL v1.7
Dryopteridaceae	Polystichopsis chaerophylloides (Poir.) Morton	rbcL	EF463216	FTOL v1.7
Dryopteridaceae	Polystichopsis chaerophylloides (Poir.) Morton	rps4-trnS	KT273080	FTOL v1.7
Dryopteridaceae	Polystichopsis chaerophylloides (Poir.) Morton	trnL-trnF	KT273222	FTOL v1.7
Dryopteridaceae	Polystichopsis muscosa (M. Vahl) Proctor	rbcL	KT272998	FTOL v1.7
Dryopteridaceae	Polystichopsis muscosa (M. Vahl) Proctor	rps4-trnS	KT273081	FTOL v1.7
Dryopteridaceae	Polystichopsis muscosa (M. Vahl) Proctor	trnL-trnF	KT273223	FTOL v1.7
Dryopteridaceae	Polystichopsis pubescens (L.) Morton	rbcL	KT273001	FTOL v1.7
Dryopteridaceae	Polystichopsis pubescens (L.) Morton	rps4-trnS	KT273084	FTOL v1.7
Dryopteridaceae	Polystichopsis pubescens (L.) Morton	trnL-trnF	KT273226	FTOL v1.7
Dryopteridaceae	Polystichum acanthophyllum (Franch.) Christ	rbcL	KU244712	FTOL v1.7
Dryopteridaceae	Polystichum acanthophyllum (Franch.) Christ	rps4-trnS	KU244873	FTOL v1.7
Dryopteridaceae	Polystichum acanthophyllum (Franch.) Christ	trnL-trnF	KU244959	FTOL v1.7
Dryopteridaceae	Polystichum acrostichoides (Michx.) Schott	atpA	KF186548	FTOL v1.7
Dryopteridaceae	Polystichum acrostichoides (Michx.) Schott	rbcL	KF186516	FTOL v1.7
Dryopteridaceae	Polystichum aculeatum (L.) Roth	rbcL	KX768041	FTOL v1.7
Dryopteridaceae	Polystichum aculeatum (L.) Roth	rps4-trnS	KX768072	FTOL v1.7
Dryopteridaceae	Polystichum aculeatum (L.) Roth	trnL-trnF	KX768064	FTOL v1.7
Dryopteridaceae	Polystichum acutidens Christ	atpA	KT831794	FTOL v1.7
Dryopteridaceae	Polystichum acutidens Christ	atpB	KT831832	FTOL v1.7
Dryopteridaceae	Polystichum acutidens Christ	rbcL	KT831872	FTOL v1.7
Dryopteridaceae	Polystichum acutipinnulum Ching & K. H. Shing	rbcL	KU244732	FTOL v1.7
Dryopteridaceae	Polystichum acutipinnulum Ching & K. H. Shing	rps4-trnS	KU244900	FTOL v1.7
Dryopteridaceae	Polystichum acutipinnulum Ching & K. H. Shing	trnL-trnF	KU244987	FTOL v1.7
Dryopteridaceae	Polystichum albomarginatum M. Kessler & A. R. Sm.	rbcL	KC819964	FTOL v1.7
Dryopteridaceae	Polystichum albomarginatum M. Kessler & A. R. Sm.	rps4	KC819994	FTOL v1.7
Dryopteridaceae	Polystichum albomarginatum M. Kessler & A. R. Sm.	rps4-trnS	KC819979	FTOL v1.7
Dryopteridaceae	Polystichum albomarginatum M. Kessler & A. R. Sm.	trnL-trnF	KC819949	FTOL v1.7
Dryopteridaceae	Polystichum alcicorne (Baker) Diels	rbcL	KU244723	FTOL v1.7
Dryopteridaceae	Polystichum alcicorne (Baker) Diels	rps4-trnS	KU244883	FTOL v1.7
Dryopteridaceae	Polystichum alcicorne (Baker) Diels	trnL-trnF	KU244970	FTOL v1.7
Dryopteridaceae	Polystichum alfaroi (Christ) Barrington	rbcL	MW000297	FTOL v1.7
Dryopteridaceae	Polystichum alfaroi (Christ) Barrington	rps4	MW000289	FTOL v1.7
Dryopteridaceae	Polystichum alfaroi (Christ) Barrington	rps4-trnS	MW000265	FTOL v1.7
Dryopteridaceae	Polystichum alfaroi (Christ) Barrington	trnL-trnF	MW000256	FTOL v1.7
Dryopteridaceae	Polystichum altum Ching ex Li Bing Zhang & H. S. Kung	rbcL	MK174897	FTOL v1.7
Dryopteridaceae	Polystichum altum Ching ex Li Bing Zhang & H. S. Kung	rps4	MK214695	FTOL v1.7
Dryopteridaceae	Polystichum altum Ching ex Li Bing Zhang & H. S. Kung	rps4-trnS	MK214695	FTOL v1.7
Dryopteridaceae	Polystichum altum Ching ex Li Bing Zhang & H. S. Kung	trnL-trnF	DQ150395	FTOL v1.7
Dryopteridaceae	Polystichum amboroense M. Kessler & A. R. Sm.	rbcL	KF020328	FTOL v1.7
Dryopteridaceae	Polystichum amboroense M. Kessler & A. R. Sm.	rps4	KF020447	FTOL v1.7
Dryopteridaceae	Polystichum amboroense M. Kessler & A. R. Sm.	rps4-trnS	KF020406	FTOL v1.7
Dryopteridaceae	Polystichum amboroense M. Kessler & A. R. Sm.	trnL-trnF	KF020353	FTOL v1.7
Dryopteridaceae	Polystichum ammifolium (Desv.) C. Chr.	rbcL	KU244752	FTOL v1.7
Dryopteridaceae	Polystichum ammifolium (Desv.) C. Chr.	rps4-trnS	KU244925	FTOL v1.7
Dryopteridaceae	Polystichum ammifolium (Desv.) C. Chr.	trnL-trnF	KU245013	FTOL v1.7
Dryopteridaceae	Polystichum andersonii Hopkins	rbcL	JN189510	FTOL v1.7
Dryopteridaceae	Polystichum andersonii Hopkins	rps4-trnS	JN189183	FTOL v1.7
Dryopteridaceae	Polystichum andersonii Hopkins	trnL-trnF	JN189078	FTOL v1.7
Dryopteridaceae	Polystichum andinum Phil.	rbcL	MK174898	FTOL v1.7
Dryopteridaceae	Polystichum andinum Phil.	rps4	MK214696	FTOL v1.7
Dryopteridaceae	Polystichum andinum Phil.	rps4-trnS	MK214696	FTOL v1.7
Dryopteridaceae	Polystichum andinum Phil.	trnL-trnF	MK035852	FTOL v1.7
Dryopteridaceae	Polystichum anomalum (Hook. & Arn.) J. Sm.	atpB	EF450460	FTOL v1.7
Dryopteridaceae	Polystichum anomalum (Hook. & Arn.) J. Sm.	rbcL	EF394248	FTOL v1.7
Dryopteridaceae	Polystichum atkinsonii Bedd.	atpB	EF450461	FTOL v1.7
Dryopteridaceae	Polystichum atkinsonii Bedd.	rbcL	EF394250	FTOL v1.7
Dryopteridaceae	Polystichum attenuatum Tagawa & K. Iwats.	plastome	MT130606	FTOL v1.7
Dryopteridaceae	Polystichum auriculum Ching	rbcL	KU244758	FTOL v1.7
Dryopteridaceae	Polystichum auriculum Ching	rps4-trnS	KU244931	FTOL v1.7
Dryopteridaceae	Polystichum auriculum Ching	trnL-trnF	KU245020	FTOL v1.7
Dryopteridaceae	Polystichum australiense Tindale	rps4-trnS	AY164624	FTOL v1.7
Dryopteridaceae	Polystichum bakerianum (Atk.) Diels	rbcL	KC878851	FTOL v1.7
Dryopteridaceae	Polystichum bakerianum (Atk.) Diels	rps4	KC890809	FTOL v1.7
Dryopteridaceae	Polystichum bakerianum (Atk.) Diels	rps4-trnS	KF020392	FTOL v1.7
Dryopteridaceae	Polystichum bakerianum (Atk.) Diels	trnL-trnF	KC878859	FTOL v1.7
Dryopteridaceae	Polystichum balansae Christ	atpA	KT831795	FTOL v1.7
Dryopteridaceae	Polystichum balansae Christ	atpB	KT831833	FTOL v1.7
Dryopteridaceae	Polystichum balansae Christ	rbcL	KT831873	FTOL v1.7
Dryopteridaceae	Polystichum basipinnatum (Baker) Diels	atpB	EF450478	FTOL v1.7
Dryopteridaceae	Polystichum basipinnatum (Baker) Diels	rbcL	EF463122	FTOL v1.7
Dryopteridaceae	Polystichum basipinnatum (Baker) Diels	rps4	EF540712	FTOL v1.7
Dryopteridaceae	Polystichum basipinnatum (Baker) Diels	rps4-trnS	EF540712	FTOL v1.7
Dryopteridaceae	Polystichum biaristatum (Blume) T. Moore	rbcL	EF177342	FTOL v1.7
Dryopteridaceae	Polystichum bifidum Ching	rbcL	KU244757	FTOL v1.7
Dryopteridaceae	Polystichum bifidum Ching	rps4-trnS	KU244930	FTOL v1.7
Dryopteridaceae	Polystichum bifidum Ching	trnL-trnF	KU245019	FTOL v1.7
Dryopteridaceae	Polystichum bonseyi W. H. Wagner & Hobdy	rbcL	KX768043	FTOL v1.7
Dryopteridaceae	Polystichum bonseyi W. H. Wagner & Hobdy	rps4-trnS	KX768075	FTOL v1.7
Dryopteridaceae	Polystichum bonseyi W. H. Wagner & Hobdy	trnL-trnF	KX768063	FTOL v1.7
Dryopteridaceae	Polystichum braunii (Spenn.) Fée	rbcL	KX768058	FTOL v1.7
Dryopteridaceae	Polystichum braunii (Spenn.) Fée	rps4-trnS	KX768079	FTOL v1.7
Dryopteridaceae	Polystichum braunii (Spenn.) Fée	trnL-trnF	KX768061	FTOL v1.7
Dryopteridaceae	Polystichum bulbiferum Barrington	rbcL	KF020329	FTOL v1.7
Dryopteridaceae	Polystichum bulbiferum Barrington	rps4	KF020449	FTOL v1.7
Dryopteridaceae	Polystichum bulbiferum Barrington	rps4-trnS	KF020408	FTOL v1.7
Dryopteridaceae	Polystichum bulbiferum Barrington	trnL-trnF	KF020355	FTOL v1.7
Dryopteridaceae	Polystichum californicum (D. C. Eaton) Diels	rbcL	KU244745	FTOL v1.7
Dryopteridaceae	Polystichum californicum (D. C. Eaton) Diels	rps4-trnS	KU244918	FTOL v1.7
Dryopteridaceae	Polystichum californicum (D. C. Eaton) Diels	trnL-trnF	KU245005	FTOL v1.7
Dryopteridaceae	Polystichum capillipes (Baker) Diels	atpB	EF450462	FTOL v1.7
Dryopteridaceae	Polystichum capillipes (Baker) Diels	rbcL	EF394249	FTOL v1.7
Dryopteridaceae	Polystichum caruifolium (Baker) Diels	rbcL	KU244724	FTOL v1.7
Dryopteridaceae	Polystichum caruifolium (Baker) Diels	rps4-trnS	KU244884	FTOL v1.7
Dryopteridaceae	Polystichum caruifolium (Baker) Diels	trnL-trnF	KU244971	FTOL v1.7
Dryopteridaceae	Polystichum caudescens Dutra	rbcL	MK174899	FTOL v1.7
Dryopteridaceae	Polystichum caudescens Dutra	rps4	MK214697	FTOL v1.7
Dryopteridaceae	Polystichum caudescens Dutra	rps4-trnS	MK214697	FTOL v1.7
Dryopteridaceae	Polystichum caudescens Dutra	trnL-trnF	MK035862	FTOL v1.7
Dryopteridaceae	Polystichum cavernicola L. B. Zhang & H. He	trnL-trnF	JF713056	FTOL v1.7
Dryopteridaceae	Polystichum chilense (Christ) Diels	rbcL	MK174900	FTOL v1.7
Dryopteridaceae	Polystichum chilense (Christ) Diels	rps4	MK214698	FTOL v1.7
Dryopteridaceae	Polystichum chilense (Christ) Diels	rps4-trnS	MK214698	FTOL v1.7
Dryopteridaceae	Polystichum chilense (Christ) Diels	trnL-trnF	MK035855	FTOL v1.7
Dryopteridaceae	Polystichum chingiae Ching	rbcL	KU244720	FTOL v1.7
Dryopteridaceae	Polystichum chingiae Ching	rps4-trnS	DQ151861	FTOL v1.7
Dryopteridaceae	Polystichum chingiae Ching	trnL-trnF	KU244967	FTOL v1.7
Dryopteridaceae	Polystichum christii Ching	rbcL	AY545486	FTOL v1.7
Dryopteridaceae	Polystichum concinnum Lellinger ex Barrington	rbcL	MW000298	FTOL v1.7
Dryopteridaceae	Polystichum concinnum Lellinger ex Barrington	rps4	MW000290	FTOL v1.7
Dryopteridaceae	Polystichum concinnum Lellinger ex Barrington	rps4-trnS	MW000266	FTOL v1.7
Dryopteridaceae	Polystichum concinnum Lellinger ex Barrington	trnL-trnF	MW000257	FTOL v1.7
Dryopteridaceae	Polystichum conjunctum (Ching) Li Bing Zhang	atpB	EF540725	FTOL v1.7
Dryopteridaceae	Polystichum conjunctum (Ching) Li Bing Zhang	rbcL	EF463123	FTOL v1.7
Dryopteridaceae	Polystichum conjunctum (Ching) Li Bing Zhang	rps4	EF540711	FTOL v1.7
Dryopteridaceae	Polystichum conjunctum (Ching) Li Bing Zhang	rps4-trnS	EF540711	FTOL v1.7
Dryopteridaceae	Polystichum craspedosorum (Maxim.) Diels	rbcL	KU244761	FTOL v1.7
Dryopteridaceae	Polystichum craspedosorum (Maxim.) Diels	rps4-trnS	KU244935	FTOL v1.7
Dryopteridaceae	Polystichum craspedosorum (Maxim.) Diels	trnL-trnF	KU245024	FTOL v1.7
Dryopteridaceae	Polystichum crinigerum (C. Chr.) Ching	rbcL	AY694813	FTOL v1.7
Dryopteridaceae	Polystichum crinigerum (C. Chr.) Ching	rps4-trnS	DQ202455	FTOL v1.7
Dryopteridaceae	Polystichum crinigerum (C. Chr.) Ching	trnL-trnF	AY736352	FTOL v1.7
Dryopteridaceae	Polystichum cyclolobum C. Chr.	rbcL	MH159212	FTOL v1.7
Dryopteridaceae	Polystichum cyclolobum C. Chr.	rps4	MH175171	FTOL v1.7
Dryopteridaceae	Polystichum cystostegia (Hook.) Armstr.	rbcL	AF208392	FTOL v1.7
Dryopteridaceae	Polystichum deltodon (Baker) Diels	plastome	MN640792	FTOL v1.7
Dryopteridaceae	Polystichum dielsii Christ	rbcL	KU244709	FTOL v1.7
Dryopteridaceae	Polystichum dielsii Christ	rps4-trnS	KU244871	FTOL v1.7
Dryopteridaceae	Polystichum dielsii Christ	trnL-trnF	KU244956	FTOL v1.7
Dryopteridaceae	Polystichum discretum (D. Don) J. Sm.	rbcL	KX768056	FTOL v1.7
Dryopteridaceae	Polystichum discretum (D. Don) J. Sm.	rps4-trnS	DQ151864	FTOL v1.7
Dryopteridaceae	Polystichum discretum (D. Don) J. Sm.	trnL-trnF	DQ150401	FTOL v1.7
Dryopteridaceae	Polystichum disjunctum Ching ex W. M. Chu & Z. R. He	rbcL	MK174902	FTOL v1.7
Dryopteridaceae	Polystichum disjunctum Ching ex W. M. Chu & Z. R. He	rps4	MK214701	FTOL v1.7
Dryopteridaceae	Polystichum distans E. Fourn.	rbcL	KC819971	FTOL v1.7
Dryopteridaceae	Polystichum distans E. Fourn.	rps4	KC820001	FTOL v1.7
Dryopteridaceae	Polystichum distans E. Fourn.	rps4-trnS	KC819986	FTOL v1.7
Dryopteridaceae	Polystichum distans E. Fourn.	trnL-trnF	KC819956	FTOL v1.7
Dryopteridaceae	Polystichum dracomontanum Schelpe & N. C. Anthony	rbcL	AF537240	FTOL v1.7

Dryopteridaceae	Polystichum drepanum (Sw.) C. Presl	rbcL	KC878852	FTOL v1.7
Dryopteridaceae	Polystichum drepanum (Sw.) C. Presl	rps4	KC890811	FTOL v1.7
Dryopteridaceae	Polystichum drepanum (Sw.) C. Presl	rps4-trnS	KF020387	FTOL v1.7
Dryopteridaceae	Polystichum drepanum (Sw.) C. Presl	trnL-trnF	KC878861	FTOL v1.7
Dryopteridaceae	Polystichum dubium (H. Karst.) Diels	rbcL	KF020330	FTOL v1.7
Dryopteridaceae	Polystichum dubium (H. Karst.) Diels	rps4	KF020450	FTOL v1.7
Dryopteridaceae	Polystichum dubium (H. Karst.) Diels	rps4-trnS	KF020410	FTOL v1.7
Dryopteridaceae	Polystichum dubium (H. Karst.) Diels	trnL-trnF	KF020357	FTOL v1.7
Dryopteridaceae	Polystichum dudleyi Maxon	rbcL	AF537241	FTOL v1.7
Dryopteridaceae	Polystichum dudleyi Maxon	rps4	MK214702	FTOL v1.7
Dryopteridaceae	Polystichum dudleyi Maxon	rps4-trnS	MK214702	FTOL v1.7
Dryopteridaceae	Polystichum dudleyi Maxon	trnL-trnF	MK776837	FTOL v1.7
Dryopteridaceae	Polystichum duthiei (Hope) C. Chr.	atpB	EF450464	FTOL v1.7
Dryopteridaceae	Polystichum duthiei (Hope) C. Chr.	rbcL	EF394252	FTOL v1.7
Dryopteridaceae	Polystichum echinatum (J. F. Gmel.) C. Chr.	rbcL	AF537242	FTOL v1.7
Dryopteridaceae	Polystichum erosum Ching & K. H. Shing	rbcL	KU244763	FTOL v1.7
Dryopteridaceae	Polystichum erosum Ching & K. H. Shing	rps4-trnS	KU244937	FTOL v1.7
Dryopteridaceae	Polystichum erosum Ching & K. H. Shing	trnL-trnF	KU245026	FTOL v1.7
Dryopteridaceae	Polystichum falcatilobum Ching ex W. M. Chu & Z. R. H	rbcL	OR543834	FTOL v1.7
Dryopteridaceae	Polystichum falcinellum (Sw.) C. Presl	rbcL	KX768055	FTOL v1.7
Dryopteridaceae	Polystichum falcinellum (Sw.) C. Presl	rps4-trnS	KX768087	FTOL v1.7
Dryopteridaceae	Polystichum fallax Tindale	rbcL	AY163865	FTOL v1.7
Dryopteridaceae	Polystichum fallax Tindale	rps4-trnS	AY164625	FTOL v1.7
Dryopteridaceae	Polystichum fengshanense L. B. Zhang & H. He	trnL-trnF	JF827030	FTOL v1.7
Dryopteridaceae	Polystichum fibrillosopaleaceum (Kodama) Tagawa	rbcL	AB232394	FTOL v1.7
Dryopteridaceae	Polystichum fimbriatum Christ	rps4-trnS	KU244859	FTOL v1.7
Dryopteridaceae	Polystichum fimbriatum Christ	trnL-trnF	KU244944	FTOL v1.7
Dryopteridaceae	Polystichum formosanum Rosenst.	rbcL	EF177337	FTOL v1.7
Dryopteridaceae	Polystichum formosanum Rosenst.	trnL-trnF	EF177307	FTOL v1.7
Dryopteridaceae	Polystichum formosum Tindale	rps4-trnS	AY164626	FTOL v1.7
Dryopteridaceae	Polystichum fournieri A. R. Sm.	rbcL	KF020331	FTOL v1.7
Dryopteridaceae	Polystichum fournieri A. R. Sm.	rps4-trnS	KF020401	FTOL v1.7
Dryopteridaceae	Polystichum fournieri A. R. Sm.	trnL-trnF	KF020362	FTOL v1.7
Dryopteridaceae	Polystichum fraxinellum (Christ) Diels	plastome	MT130647	FTOL v1.7
Dryopteridaceae	Polystichum fugongense Ching & W. M. Chu	rps4-trnS	DQ202458	FTOL v1.7
Dryopteridaceae	Polystichum fugongense Ching & W. M. Chu	trnL-trnF	DQ202426	FTOL v1.7
Dryopteridaceae	Polystichum gelidum (Kunze ex Klotzsch) Fée	rbcL	KF020332	FTOL v1.7
Dryopteridaceae	Polystichum gelidum (Kunze ex Klotzsch) Fée	rps4	KF020460	FTOL v1.7
Dryopteridaceae	Polystichum gelidum (Kunze ex Klotzsch) Fée	rps4-trnS	KF020423	FTOL v1.7
Dryopteridaceae	Polystichum gelidum (Kunze ex Klotzsch) Fée	trnL-trnF	KF020359	FTOL v1.7
Dryopteridaceae	Polystichum glaciale Christ	atpB	KY241400	FTOL v1.7
Dryopteridaceae	Polystichum glaciale Christ	rbcL	KY241390	FTOL v1.7
Dryopteridaceae	Polystichum glaciale Christ	trnL-trnF	KY241387	FTOL v1.7
Dryopteridaceae	Polystichum glandulosum C. Presl	rbcL	KC878850	FTOL v1.7
Dryopteridaceae	Polystichum glandulosum C. Presl	trnL-trnF	KC878869	FTOL v1.7
Dryopteridaceae	Polystichum grandifrons C. Chr.	rbcL	KU244719	FTOL v1.7
Dryopteridaceae	Polystichum grandifrons C. Chr.	rps4-trnS	KU244879	FTOL v1.7
Dryopteridaceae	Polystichum grandifrons C. Chr.	trnL-trnF	KU244966	FTOL v1.7
Dryopteridaceae	Polystichum haleakalense Brack.	rbcL	EF177322	FTOL v1.7
Dryopteridaceae	Polystichum haleakalense Brack.	rps4-trnS	KX768088	FTOL v1.7
Dryopteridaceae	Polystichum haleakalense Brack.	trnL-trnF	EF177278	FTOL v1.7
Dryopteridaceae	Polystichum hancockii (Hance) Diels	atpA	KT831796	FTOL v1.7
Dryopteridaceae	Polystichum hancockii (Hance) Diels	atpB	KT831836	FTOL v1.7
Dryopteridaceae	Polystichum hancockii (Hance) Diels	rbcL	KT831876	FTOL v1.7
Dryopteridaceae	Polystichum hartwegii (Klotzsch) Hieron.	rbcL	KF020335	FTOL v1.7
Dryopteridaceae	Polystichum hartwegii (Klotzsch) Hieron.	rps4	KF020444	FTOL v1.7
Dryopteridaceae	Polystichum hartwegii (Klotzsch) Hieron.	rps4-trnS	KF020400	FTOL v1.7
Dryopteridaceae	Polystichum hartwegii (Klotzsch) Hieron.	trnL-trnF	KF020363	FTOL v1.7
Dryopteridaceae	Polystichum hecatopterum Diels	rps4-trnS	KU244939	FTOL v1.7
Dryopteridaceae	Polystichum hecatopterum Diels	trnL-trnF	KU245030	FTOL v1.7
Dryopteridaceae	Polystichum herbaceum Ching & Z. Y. Liu	rbcL	MH159214	FTOL v1.7
Dryopteridaceae	Polystichum herbaceum Ching & Z. Y. Liu	rps4	MH175175	FTOL v1.7
Dryopteridaceae	Polystichum herbaceum Ching & Z. Y. Liu	rps4-trnS	MH175175	FTOL v1.7
Dryopteridaceae	Polystichum hillebrandii Carruth.	atpA	EF463720	FTOL v1.7
Dryopteridaceae	Polystichum hillebrandii Carruth.	atpB	EF463436	FTOL v1.7
Dryopteridaceae	Polystichum hillebrandii Carruth.	rbcL	EF463217	FTOL v1.7
Dryopteridaceae	Polystichum holttumii C. Chr.	rbcL	KX768059	FTOL v1.7
Dryopteridaceae	Polystichum holttumii C. Chr.	rps4-trnS	KX768090	FTOL v1.7
Dryopteridaceae	Polystichum hookerianum (C. Presl) C. Chr.	rbcL	OR543841	FTOL v1.7
Dryopteridaceae	Polystichum hookerianum (C. Presl) C. Chr.	rps4	OR562007	FTOL v1.7
Dryopteridaceae	Polystichum hookerianum (C. Presl) C. Chr.	rps4-trnS	OR562007	FTOL v1.7
Dryopteridaceae	Polystichum hookerianum (C. Presl) C. Chr.	trnL-trnF	OR543851	FTOL v1.7
Dryopteridaceae	Polystichum hubeiense Liang Zhang & Li Bing Zhang	trnL-trnF	KU245029	FTOL v1.7
Dryopteridaceae	Polystichum igaense Tagawa	rbcL	AB575199	FTOL v1.7
Dryopteridaceae	Polystichum imbricans (D. C. Eaton) D. H. Wagner	rbcL	KU244746	FTOL v1.7
Dryopteridaceae	Polystichum imbricans (D. C. Eaton) D. H. Wagner	rps4-trnS	KU244919	FTOL v1.7
Dryopteridaceae	Polystichum imbricans (D. C. Eaton) D. H. Wagner	trnL-trnF	KU245006	FTOL v1.7
Dryopteridaceae	Polystichum incongruum J. P. Roux	rbcL	KU244749	FTOL v1.7
Dryopteridaceae	Polystichum incongruum J. P. Roux	rps4-trnS	KU244922	FTOL v1.7
Dryopteridaceae	Polystichum incongruum J. P. Roux	trnL-trnF	KU245009	FTOL v1.7
Dryopteridaceae	Polystichum jiucaipingense P. S. Wang & Q. Luo	rbcL	KU244702	FTOL v1.7
Dryopteridaceae	Polystichum jiucaipingense P. S. Wang & Q. Luo	rps4-trnS	KU244856	FTOL v1.7
Dryopteridaceae	Polystichum jiucaipingense P. S. Wang & Q. Luo	trnL-trnF	KU244941	FTOL v1.7
Dryopteridaceae	Polystichum jiulaodongense W. M. Chu & Z. R. He	rps4-trnS	KU244938	FTOL v1.7
Dryopteridaceae	Polystichum jiulaodongense W. M. Chu & Z. R. He	trnL-trnF	KU245027	FTOL v1.7
Dryopteridaceae	Polystichum kenwoodii Lorence & W. L. Wagner	rbcL	MT216090	FTOL v1.7
Dryopteridaceae	Polystichum kruckebergii W. H. Wagner	rbcL	MG215518	FTOL v1.7
Dryopteridaceae	Polystichum kungianum H. He & Li Bing Zhang	rbcL	KU244764	FTOL v1.7
Dryopteridaceae	Polystichum kungianum H. He & Li Bing Zhang	trnL-trnF	KU245028	FTOL v1.7
Dryopteridaceae	Polystichum lachenense (Hook.) Bedd.	rbcL	KU662013	FTOL v1.7
Dryopteridaceae	Polystichum lachenense (Hook.) Bedd.	rps4-trnS	KU662031	FTOL v1.7
Dryopteridaceae	Polystichum lachenense (Hook.) Bedd.	trnL-trnF	KU662020	FTOL v1.7
Dryopteridaceae	Polystichum lehmannii Hieron.	rbcL	KF020336	FTOL v1.7
Dryopteridaceae	Polystichum lehmannii Hieron.	rps4	KF020451	FTOL v1.7
Dryopteridaceae	Polystichum lehmannii Hieron.	rps4-trnS	KF020412	FTOL v1.7
Dryopteridaceae	Polystichum lehmannii Hieron.	trnL-trnF	KF020364	FTOL v1.7
Dryopteridaceae	Polystichum lemmonii Underw.	atpA	EF463721	FTOL v1.7
Dryopteridaceae	Polystichum lemmonii Underw.	atpB	EF463437	FTOL v1.7
Dryopteridaceae	Polystichum lemmonii Underw.	rbcL	EF463218	FTOL v1.7
Dryopteridaceae	Polystichum lentum (D. Don) T. Moore	rbcL	OR543837	FTOL v1.7
Dryopteridaceae	Polystichum lentum (D. Don) T. Moore	trnL-trnF	OR543856	FTOL v1.7
Dryopteridaceae	Polystichum lepidocaulon (Hook.) J. Sm.	atpA	KT831797	FTOL v1.7
Dryopteridaceae	Polystichum lepidocaulon (Hook.) J. Sm.	atpB	KT831837	FTOL v1.7
Dryopteridaceae	Polystichum lepidocaulon (Hook.) J. Sm.	rbcL	KT831877	FTOL v1.7
Dryopteridaceae	Polystichum lepidotum Sundue & M. Kessler	rbcL	KF020338	FTOL v1.7
Dryopteridaceae	Polystichum lepidotum Sundue & M. Kessler	rps4	KF020477	FTOL v1.7
Dryopteridaceae	Polystichum lepidotum Sundue & M. Kessler	rps4-trnS	KF020415	FTOL v1.7
Dryopteridaceae	Polystichum lepidotum Sundue & M. Kessler	trnL-trnF	KF020367	FTOL v1.7
Dryopteridaceae	Polystichum levingei C. Hope ex Christ	rbcL	MK174915	FTOL v1.7
Dryopteridaceae	Polystichum levingei C. Hope ex Christ	rps4	MK214720	FTOL v1.7
Dryopteridaceae	Polystichum levingei C. Hope ex Christ	rps4-trnS	MK214720	FTOL v1.7
Dryopteridaceae	Polystichum lilianae Barrington	rbcL	MW000300	FTOL v1.7
Dryopteridaceae	Polystichum lilianae Barrington	rps4	MW000292	FTOL v1.7
Dryopteridaceae	Polystichum lilianae Barrington	rps4-trnS	MW000268	FTOL v1.7
Dryopteridaceae	Polystichum lilianae Barrington	trnL-trnF	MW000259	FTOL v1.7
Dryopteridaceae	Polystichum liui Ching	rps4	EF540715	FTOL v1.7
Dryopteridaceae	Polystichum liui Ching	rps4-trnS	EF540715	FTOL v1.7
Dryopteridaceae	Polystichum liui Ching	trnL-trnF	EF540703	FTOL v1.7
Dryopteridaceae	Polystichum lonchitis (L.) Roth	rbcL	MK174903	FTOL v1.7
Dryopteridaceae	Polystichum lonchitis (L.) Roth	rps4	MK214705	FTOL v1.7
Dryopteridaceae	Polystichum lonchitis (L.) Roth	rps4-trnS	MK214705	FTOL v1.7
Dryopteridaceae	Polystichum lonchitis (L.) Roth	trnL-trnF	MK035863	FTOL v1.7
Dryopteridaceae	Polystichum longifrons Kurata	rbcL	AB575204	FTOL v1.7
Dryopteridaceae	Polystichum longipaleatum Christ	rbcL	AY694814	FTOL v1.7
Dryopteridaceae	Polystichum longipaleatum Christ	trnL-trnF	AY736353	FTOL v1.7
Dryopteridaceae	Polystichum longispinosum Ching ex Li Bing Zhang & H	rbcL	KU244706	FTOL v1.7
Dryopteridaceae	Polystichum longispinosum Ching ex Li Bing Zhang & H	rps4-trnS	KU244867	FTOL v1.7
Dryopteridaceae	Polystichum longispinosum Ching ex Li Bing Zhang & H	trnL-trnF	KU244951	FTOL v1.7
Dryopteridaceae	Polystichum luctuosum (Kunze) T. Moore	atpA	KT831800	FTOL v1.7
Dryopteridaceae	Polystichum luctuosum (Kunze) T. Moore	atpB	KT831840	FTOL v1.7
Dryopteridaceae	Polystichum luctuosum (Kunze) T. Moore	rbcL	KT831881	FTOL v1.7
Dryopteridaceae	Polystichum macleaii (Baker) Diels	rbcL	AF537249	FTOL v1.7
Dryopteridaceae	Polystichum maevaranense Tardieu	rbcL	KU244747	FTOL v1.7
Dryopteridaceae	Polystichum maevaranense Tardieu	trnL-trnF	KU245008	FTOL v1.7
Dryopteridaceae	Polystichum makinoi (Tagawa) Tagawa	atpB	EF450467	FTOL v1.7
Dryopteridaceae	Polystichum makinoi (Tagawa) Tagawa	rbcL	EF394247	FTOL v1.7
Dryopteridaceae	Polystichum manmeiense (Christ) Nakaike	rbcL	KU244717	FTOL v1.7
Dryopteridaceae	Polystichum manmeiense (Christ) Nakaike	rps4-trnS	KU244877	FTOL v1.7
Dryopteridaceae	Polystichum manmeiense (Christ) Nakaike	trnL-trnF	KU244964	FTOL v1.7
Dryopteridaceae	Polystichum marquesense E. D. Br.	rbcL	MT216092	FTOL v1.7
Dryopteridaceae	Polystichum marquesense E. D. Br.	trnL-trnF	EF177281	FTOL v1.7
Dryopteridaceae	Polystichum maximum M. Kessler & A. R. Sm.	rbcL	KC819965	FTOL v1.7
Dryopteridaceae	Polystichum maximum M. Kessler & A. R. Sm.	rps4	KC819995	FTOL v1.7
Dryopteridaceae	Polystichum maximum M. Kessler & A. R. Sm.	rps4-trnS	KC819980	FTOL v1.7
Dryopteridaceae	Polystichum maximum M. Kessler & A. R. Sm.	trnL-trnF	KC819950	FTOL v1.7
Dryopteridaceae	Polystichum mayebarae Tagawa	rbcL	MH159216	FTOL v1.7
Dryopteridaceae	Polystichum microchlamys (Christ) Matsum.	rbcL	AB575188	FTOL v1.7
Dryopteridaceae	Polystichum microchlamys (Christ) Matsum.	rps4-trnS	KX856982	FTOL v1.7
Dryopteridaceae	Polystichum microchlamys (Christ) Matsum.	trnL-trnF	KX856981	FTOL v1.7
Dryopteridaceae	Polystichum minimum (Y. T. Hsieh) Li Bing Zhang	rbcL	AY694812	FTOL v1.7
Dryopteridaceae	Polystichum minimum (Y. T. Hsieh) Li Bing Zhang	trnL-trnF	AY736351	FTOL v1.7
Dryopteridaceae	Polystichum minutissimum L. B. Zhang & H. He	trnL-trnF	JF827028	FTOL v1.7
Dryopteridaceae	Polystichum mohrioides (Bory) C. Presl	rbcL	MK174905	FTOL v1.7
Dryopteridaceae	Polystichum mohrioides (Bory) C. Presl	rps4	MK214708	FTOL v1.7
Dryopteridaceae	Polystichum mohrioides (Bory) C. Presl	rps4-trnS	MK214708	FTOL v1.7
Dryopteridaceae	Polystichum mohrioides (Bory) C. Presl	trnL-trnF	MK035847	FTOL v1.7
Dryopteridaceae	Polystichum moluccense (Blume) T. Moore	rbcL	KX768050	FTOL v1.7
Dryopteridaceae	Polystichum moluccense (Blume) T. Moore	rps4-trnS	KX768091	FTOL v1.7
Dryopteridaceae	Polystichum montevidense (Spreng.) Rosenst.	rbcL	MK174907	FTOL v1.7
Dryopteridaceae	Polystichum montevidense (Spreng.) Rosenst.	rps4	MK214710	FTOL v1.7
Dryopteridaceae	Polystichum montevidense (Spreng.) Rosenst.	rps4-trnS	MK214710	FTOL v1.7
Dryopteridaceae	Polystichum montevidense (Spreng.) Rosenst.	trnL-trnF	MK035865	FTOL v1.7
Dryopteridaceae	Polystichum moorei Christ	trnL-trnF	KU245033	FTOL v1.7
Dryopteridaceae	Polystichum mucronifolium (Blume) B. K. Nayar & Kau	rbcL	EF177340	FTOL v1.7
Dryopteridaceae	Polystichum mucronifolium (Blume) B. K. Nayar & Kau	rps4-trnS	KU244875	FTOL v1.7
Dryopteridaceae	Polystichum mucronifolium (Blume) B. K. Nayar & Kau	trnL-trnF	KU244962	FTOL v1.7
Dryopteridaceae	Polystichum multifidum (Mett.) T. Moore	rbcL	MK174909	FTOL v1.7
Dryopteridaceae	Polystichum multifidum (Mett.) T. Moore	rps4	MK214712	FTOL v1.7
Dryopteridaceae	Polystichum multifidum (Mett.) T. Moore	rps4-trnS	MK214712	FTOL v1.7
Dryopteridaceae	Polystichum multifidum (Mett.) T. Moore	trnL-trnF	MK035856	FTOL v1.7
Dryopteridaceae	Polystichum munitum (Kaulf.) C. Presl	rbcL	JN189508	FTOL v1.7
Dryopteridaceae	Polystichum munitum (Kaulf.) C. Presl	rps4-trnS	JN189182	FTOL v1.7
Dryopteridaceae	Polystichum munitum (Kaulf.) C. Presl	trnL-trnF	JN189076	FTOL v1.7
Dryopteridaceae	Polystichum muricatum (L.) Fée	rbcL	AF537251	FTOL v1.7
Dryopteridaceae	Polystichum neolobatum Nakai	atpA	KT831798	FTOL v1.7
Dryopteridaceae	Polystichum neolobatum Nakai	atpB	KT831838	FTOL v1.7
Dryopteridaceae	Polystichum neolobatum Nakai	rbcL	KT831878	FTOL v1.7
Dryopteridaceae	Polystichum neozelandicum Fée	rbcL	AF208394	FTOL v1.7
Dryopteridaceae	Polystichum nepalense (Spreng.) C. Chr.	rbcL	KU244713	FTOL v1.7
Dryopteridaceae	Polystichum nepalense (Spreng.) C. Chr.	trnL-trnF	KU244960	FTOL v1.7
Dryopteridaceae	Polystichum normale Ching ex P. S. Wang & Li Bing Zha	trnL-trnF	JF713068	FTOL v1.7
Dryopteridaceae	Polystichum nudicaule Rosenst.	rbcL	KF020321	FTOL v1.7
Dryopteridaceae	Polystichum nudicaule Rosenst.	rps4	KF020454	FTOL v1.7
Dryopteridaceae	Polystichum nudicaule Rosenst.	rps4-trnS	KF020417	FTOL v1.7
Dryopteridaceae	Polystichum nudicaule Rosenst.	trnL-trnF	KF020369	FTOL v1.7
Dryopteridaceae	Polystichum obai Tagawa	rbcL	AB575209	FTOL v1.7
Dryopteridaceae	Polystichum obliquum (D. Don) T. Moore	rbcL	OR543846	FTOL v1.7

Dryopteridaceae	Polystichum obliquum (D. Don) T. Moore	rps4	OR562012	FTOL v1.7
Dryopteridaceae	Polystichum obliquum (D. Don) T. Moore	rps4-trnS	OR562012	FTOL v1.7
Dryopteridaceae	Polystichum obliquum (D. Don) T. Moore	trnL-trnF	OR543847	FTOL v1.7
Dryopteridaceae	Polystichum oculatum Armstr.	rps4-trnS	AY164633	FTOL v1.7
Dryopteridaceae	Polystichum ohmurae Kurata	rbcL	AB575210	FTOL v1.7
Dryopteridaceae	Polystichum orbiculatum (Desv.) J. Rémy & Fée	rbcL	KF020340	FTOL v1.7
Dryopteridaceae	Polystichum orbiculatum (Desv.) J. Rémy & Fée	rps4	KF020458	FTOL v1.7
Dryopteridaceae	Polystichum orbiculatum (Desv.) J. Rémy & Fée	rps4-trnS	KF020421	FTOL v1.7
Dryopteridaceae	Polystichum orbiculatum (Desv.) J. Rémy & Fée	trnL-trnF	KF020372	FTOL v1.7
Dryopteridaceae	Polystichum oreodoxa H. S. Kung & L. B. Zhang ex Ching	rps4-trnS	DQ202467	FTOL v1.7
Dryopteridaceae	Polystichum oreodoxa H. S. Kung & L. B. Zhang ex Ching	trnL-trnF	DQ202435	FTOL v1.7
Dryopteridaceae	Polystichum otomasui Kurata	rbcL	AB575211	FTOL v1.7
Dryopteridaceae	Polystichum otophorum (Franch.) Bedd.	rbcL	KU244711	FTOL v1.7
Dryopteridaceae	Polystichum otophorum (Franch.) Bedd.	trnL-trnF	KU244958	FTOL v1.7
Dryopteridaceae	Polystichum ovatopaleaceum (Kodama) Kurata	rbcL	AB575213	FTOL v1.7
Dryopteridaceae	Polystichum paleatum Copel.	rbcL	KY099848	FTOL v1.7
Dryopteridaceae	Polystichum paramicola M. Kessler & A. R. Sm.	rbcL	MW000303	FTOL v1.7
Dryopteridaceae	Polystichum paramicola M. Kessler & A. R. Sm.	trnL-trnF	MW000264	FTOL v1.7
Dryopteridaceae	Polystichum parvifoliolatum W. M. Chu	rps4-trnS	DQ151873	FTOL v1.7
Dryopteridaceae	Polystichum parvifoliolatum W. M. Chu	trnL-trnF	DQ150411	FTOL v1.7
Dryopteridaceae	Polystichum parvipinnulum Tagawa	rbcL	KU244739	FTOL v1.7
Dryopteridaceae	Polystichum parvipinnulum Tagawa	rps4-trnS	KU244910	FTOL v1.7
Dryopteridaceae	Polystichum parvipinnulum Tagawa	trnL-trnF	KU244997	FTOL v1.7
Dryopteridaceae	Polystichum perpusillum Li Bing Zhang & H. He	trnL-trnF	JF713058	FTOL v1.7
Dryopteridaceae	Polystichum piceopaleaceum Tagawa	plastome	PP236762	FTOL v1.7
Dryopteridaceae	Polystichum platylepis Fée	rbcL	KC819974	FTOL v1.7
Dryopteridaceae	Polystichum platylepis Fée	rps4	KC820004	FTOL v1.7
Dryopteridaceae	Polystichum platylepis Fée	rps4-trnS	KC819989	FTOL v1.7
Dryopteridaceae	Polystichum platylepis Fée	trnL-trnF	KC819959	FTOL v1.7
Dryopteridaceae	Polystichum platyphyllum (Willd.) C. Presl	rbcL	KF020325	FTOL v1.7
Dryopteridaceae	Polystichum platyphyllum (Willd.) C. Presl	rps4	KF020464	FTOL v1.7
Dryopteridaceae	Polystichum platyphyllum (Willd.) C. Presl	rps4-trnS	KF020428	FTOL v1.7
Dryopteridaceae	Polystichum platyphyllum (Willd.) C. Presl	trnL-trnF	KF020373	FTOL v1.7
Dryopteridaceae	Polystichum plicatum (Poepp. ex Kunze) Hicken ex Hos	rbcL	MK174911	FTOL v1.7
Dryopteridaceae	Polystichum plicatum (Poepp. ex Kunze) Hicken ex Hos	rps4	MK214715	FTOL v1.7
Dryopteridaceae	Polystichum plicatum (Poepp. ex Kunze) Hicken ex Hos	rps4-trnS	MK214715	FTOL v1.7
Dryopteridaceae	Polystichum plicatum (Poepp. ex Kunze) Hicken ex Hos	trnL-trnF	MK035853	FTOL v1.7
Dryopteridaceae	Polystichum polyblepharum (Roem. ex Kunze) C. Presl	rbcL	AB575216	FTOL v1.7
Dryopteridaceae	Polystichum polyphyllum (C. Presl) C. Presl	rbcL	KF020342	FTOL v1.7
Dryopteridaceae	Polystichum polyphyllum (C. Presl) C. Presl	rps4	KF020455	FTOL v1.7
Dryopteridaceae	Polystichum polyphyllum (C. Presl) C. Presl	rps4-trnS	KF020418	FTOL v1.7
Dryopteridaceae	Polystichum polyphyllum (C. Presl) C. Presl	trnL-trnF	KF020374	FTOL v1.7
Dryopteridaceae	Polystichum prescottianum (Wall. ex Mett.) T. Moore	rbcL	KU662014	FTOL v1.7
Dryopteridaceae	Polystichum prescottianum (Wall. ex Mett.) T. Moore	rps4-trnS	KU662032	FTOL v1.7
Dryopteridaceae	Polystichum prescottianum (Wall. ex Mett.) T. Moore	trnL-trnF	KU662021	FTOL v1.7
Dryopteridaceae	Polystichum proliferum (R. Br.) C. Presl	rbcL	AF208393	FTOL v1.7
Dryopteridaceae	Polystichum pseudomakinoi Tagawa	rbcL	KX768053	FTOL v1.7
Dryopteridaceae	Polystichum pseudomakinoi Tagawa	rps4-trnS	KX768100	FTOL v1.7
Dryopteridaceae	Polystichum pseudostenophyllum Tagawa	rps4-trnS	KU662036	FTOL v1.7
Dryopteridaceae	Polystichum pseudostenophyllum Tagawa	trnL-trnF	KU662025	FTOL v1.7
Dryopteridaceae	Polystichum punctiferum C. Chr.	rbcL	MK174914	FTOL v1.7
Dryopteridaceae	Polystichum punctiferum C. Chr.	rps4	MK214718	FTOL v1.7
Dryopteridaceae	Polystichum punctiferum C. Chr.	rps4-trnS	MK214718	FTOL v1.7
Dryopteridaceae	Polystichum punctiferum C. Chr.	trnL-trnF	DQ150413	FTOL v1.7
Dryopteridaceae	Polystichum pungens (Kaulf.) C. Presl	rbcL	AF537253	FTOL v1.7
Dryopteridaceae	Polystichum puteicola L. B. Zhang, H. He & Q. Luo	trnL-trnF	GQ244335	FTOL v1.7
Dryopteridaceae	Polystichum putuoense Li Bing Zhang	atpB	EF450479	FTOL v1.7
Dryopteridaceae	Polystichum putuoense Li Bing Zhang	rbcL	EF463124	FTOL v1.7
Dryopteridaceae	Polystichum putuoense Li Bing Zhang	rps4	EF540710	FTOL v1.7
Dryopteridaceae	Polystichum putuoense Li Bing Zhang	rps4-trnS	EF540710	FTOL v1.7
Dryopteridaceae	Polystichum putuoense Li Bing Zhang	trnL-trnF	EF540697	FTOL v1.7
Dryopteridaceae	Polystichum pycnolepis (Kunze ex Klotzsch) T. Moore	rbcL	KF020324	FTOL v1.7
Dryopteridaceae	Polystichum pycnolepis (Kunze ex Klotzsch) T. Moore	rps4	KF020467	FTOL v1.7
Dryopteridaceae	Polystichum pycnolepis (Kunze ex Klotzsch) T. Moore	rps4-trnS	KF020432	FTOL v1.7
Dryopteridaceae	Polystichum pycnolepis (Kunze ex Klotzsch) T. Moore	trnL-trnF	KF020376	FTOL v1.7
Dryopteridaceae	Polystichum pycnopterum (Christ) Ching ex W. M. Chu	rbcL	KU244714	FTOL v1.7
Dryopteridaceae	Polystichum pycnopterum (Christ) Ching ex W. M. Chu	rps4-trnS	KU244874	FTOL v1.7
Dryopteridaceae	Polystichum pycnopterum (Christ) Ching ex W. M. Chu	trnL-trnF	KU244961	FTOL v1.7
Dryopteridaceae	Polystichum rapense E. D. Br.	rbcL	KC896009	FTOL v1.7
Dryopteridaceae	Polystichum rapense E. D. Br.	rps4-trnS	KX768102	FTOL v1.7
Dryopteridaceae	Polystichum rapense E. D. Br.	trnL-trnF	KC896008	FTOL v1.7
Dryopteridaceae	Polystichum retrosopaleaceum (Kodama) Tagawa	rbcL	KX768052	FTOL v1.7
Dryopteridaceae	Polystichum retrosopaleaceum (Kodama) Tagawa	rps4-trnS	DQ202470	FTOL v1.7
Dryopteridaceae	Polystichum retrosopaleaceum (Kodama) Tagawa	trnL-trnF	DQ202438	FTOL v1.7
Dryopteridaceae	Polystichum revolutum P. S. Wang	rbcL	KU244708	FTOL v1.7
Dryopteridaceae	Polystichum revolutum P. S. Wang	rps4-trnS	KU244869	FTOL v1.7
Dryopteridaceae	Polystichum revolutum P. S. Wang	trnL-trnF	KU244953	FTOL v1.7
Dryopteridaceae	Polystichum rhizophyllum (Sw.) C. Presl	rbcL	KC878855	FTOL v1.7
Dryopteridaceae	Polystichum rhizophyllum (Sw.) C. Presl	rps4	KC890821	FTOL v1.7
Dryopteridaceae	Polystichum rhizophyllum (Sw.) C. Presl	rps4-trnS	KF020394	FTOL v1.7
Dryopteridaceae	Polystichum rhizophyllum (Sw.) C. Presl	trnL-trnF	KC878867	FTOL v1.7
Dryopteridaceae	Polystichum rigens Tagawa	atpA	KT831799	FTOL v1.7
Dryopteridaceae	Polystichum rigens Tagawa	atpB	KT831839	FTOL v1.7
Dryopteridaceae	Polystichum rigens Tagawa	rbcL	KT831879	FTOL v1.7
Dryopteridaceae	Polystichum rochaleanum Glaz. ex Fée	rbcL	KC819976	FTOL v1.7
Dryopteridaceae	Polystichum rochaleanum Glaz. ex Fée	rps4	KC820006	FTOL v1.7
Dryopteridaceae	Polystichum rochaleanum Glaz. ex Fée	rps4-trnS	KC819991	FTOL v1.7
Dryopteridaceae	Polystichum rochaleanum Glaz. ex Fée	trnL-trnF	KC819961	FTOL v1.7
Dryopteridaceae	Polystichum rufum M. Kessler & A. R. Sm.	rbcL	KC819977	FTOL v1.7
Dryopteridaceae	Polystichum rufum M. Kessler & A. R. Sm.	rps4	KC820007	FTOL v1.7
Dryopteridaceae	Polystichum rufum M. Kessler & A. R. Sm.	rps4-trnS	KC819992	FTOL v1.7
Dryopteridaceae	Polystichum rufum M. Kessler & A. R. Sm.	trnL-trnF	KC819962	FTOL v1.7
Dryopteridaceae	Polystichum scopulinum (D. C. Eaton) Maxon	rbcL	KX768047	FTOL v1.7
Dryopteridaceae	Polystichum scopulinum (D. C. Eaton) Maxon	rps4	KC890817	FTOL v1.7
Dryopteridaceae	Polystichum scopulinum (D. C. Eaton) Maxon	rps4-trnS	KX768104	FTOL v1.7
Dryopteridaceae	Polystichum scopulinum (D. C. Eaton) Maxon	trnL-trnF	KC878860	FTOL v1.7
Dryopteridaceae	Polystichum semifertile (C. B. Clarke) Ching	plastome	MT130677	FTOL v1.7
Dryopteridaceae	Polystichum setiferum (Forssk.) T. Moore ex Woyn.	rbcL	AF537254	FTOL v1.7
Dryopteridaceae	Polystichum setigerum (C. Presl) C. Presl	rbcL	KX768039	FTOL v1.7
Dryopteridaceae	Polystichum setigerum (C. Presl) C. Presl	rps4-trnS	KX768105	FTOL v1.7
Dryopteridaceae	Polystichum shensiense Christ	rbcL	KU244728	FTOL v1.7
Dryopteridaceae	Polystichum shensiense Christ	rps4-trnS	KU244894	FTOL v1.7
Dryopteridaceae	Polystichum shensiense Christ	trnL-trnF	KU244981	FTOL v1.7
Dryopteridaceae	Polystichum shimurae Sa. Kurata ex Seriz.	rbcL	AB575220	FTOL v1.7
Dryopteridaceae	Polystichum silvaticum (Colenso) Diels	rps4-trnS	AY164634	FTOL v1.7
Dryopteridaceae	Polystichum sinense (Christ) Christ	rbcL	KU244755	FTOL v1.7
Dryopteridaceae	Polystichum sinense (Christ) Christ	rps4-trnS	KU244928	FTOL v1.7
Dryopteridaceae	Polystichum sinense (Christ) Christ	trnL-trnF	KU245016	FTOL v1.7
Dryopteridaceae	Polystichum sinotsus-simense Ching & Z. Y. Liu	rbcL	KC878857	FTOL v1.7
Dryopteridaceae	Polystichum sinotsus-simense Ching & Z. Y. Liu	rps4	KC890820	FTOL v1.7
Dryopteridaceae	Polystichum sinotsus-simense Ching & Z. Y. Liu	rps4-trnS	DQ151878	FTOL v1.7
Dryopteridaceae	Polystichum sinotsus-simense Ching & Z. Y. Liu	trnL-trnF	DQ150416	FTOL v1.7
Dryopteridaceae	Polystichum sodiroi Christ	rbcL	KF020347	FTOL v1.7
Dryopteridaceae	Polystichum sodiroi Christ	rps4	KF020470	FTOL v1.7
Dryopteridaceae	Polystichum sodiroi Christ	rps4-trnS	KF020436	FTOL v1.7
Dryopteridaceae	Polystichum sodiroi Christ	trnL-trnF	KF020379	FTOL v1.7
Dryopteridaceae	Polystichum solomonii M. Kessler & A. R. Sm.	rbcL	KC819978	FTOL v1.7
Dryopteridaceae	Polystichum solomonii M. Kessler & A. R. Sm.	rps4	KC820008	FTOL v1.7
Dryopteridaceae	Polystichum solomonii M. Kessler & A. R. Sm.	rps4-trnS	KC819993	FTOL v1.7
Dryopteridaceae	Polystichum solomonii M. Kessler & A. R. Sm.	trnL-trnF	KC819963	FTOL v1.7
Dryopteridaceae	Polystichum speciosissimum (A. Braun ex Kunze) Copel	rbcL	AF537255	FTOL v1.7
Dryopteridaceae	Polystichum speluncicola L. B. Zhang & H. He	trnL-trnF	GQ244334	FTOL v1.7
Dryopteridaceae	Polystichum squarrosum (D. Don) Fée	rbcL	EF177339	FTOL v1.7
Dryopteridaceae	Polystichum stimulans (Kunze ex Mett.) Bedd.	rbcL	MH159213	FTOL v1.7
Dryopteridaceae	Polystichum stimulans (Kunze ex Mett.) Bedd.	rps4	MH175172	FTOL v1.7
Dryopteridaceae	Polystichum stimulans (Kunze ex Mett.) Bedd.	rps4-trnS	MH175172	FTOL v1.7
Dryopteridaceae	Polystichum stimulans (Kunze ex Mett.) Bedd.	trnL-trnF	DQ150417	FTOL v1.7
Dryopteridaceae	Polystichum stuebelii Hieron.	rbcL	KF020323	FTOL v1.7
Dryopteridaceae	Polystichum stuebelii Hieron.	rps4	KF020472	FTOL v1.7
Dryopteridaceae	Polystichum stuebelii Hieron.	rps4-trnS	KF020438	FTOL v1.7
Dryopteridaceae	Polystichum stuebelii Hieron.	trnL-trnF	KF020380	FTOL v1.7
Dryopteridaceae	Polystichum subacutidens L. L. Xiang ex Ching	rbcL	DQ508787	FTOL v1.7
Dryopteridaceae	Polystichum subacutidens L. L. Xiang ex Ching	trnL-trnF	DQ514518	FTOL v1.7
Dryopteridaceae	Polystichum subfimbriatum W. M. Chu & Z. R. He	rbcL	KU244721	FTOL v1.7
Dryopteridaceae	Polystichum subfimbriatum W. M. Chu & Z. R. He	rps4-trnS	KU244881	FTOL v1.7
Dryopteridaceae	Polystichum subfimbriatum W. M. Chu & Z. R. He	trnL-trnF	KU244968	FTOL v1.7
Dryopteridaceae	Polystichum subintegerrimum (Hook. & Arn.) R. Rodr.	rbcL	MK174916	FTOL v1.7
Dryopteridaceae	Polystichum subintegerrimum (Hook. & Arn.) R. Rodr.	rps4	MK214723	FTOL v1.7
Dryopteridaceae	Polystichum subintegerrimum (Hook. & Arn.) R. Rodr.	rps4-trnS	MK214723	FTOL v1.7
Dryopteridaceae	Polystichum subintegerrimum (Hook. & Arn.) R. Rodr.	trnL-trnF	MK035858	FTOL v1.7
Dryopteridaceae	Polystichum submarginale (Baker) L. L. Xiang ex Ching	rbcL	KU244707	FTOL v1.7
Dryopteridaceae	Polystichum submarginale (Baker) L. L. Xiang ex Ching	rps4-trnS	KU244868	FTOL v1.7
Dryopteridaceae	Polystichum submarginale (Baker) L. L. Xiang ex Ching	trnL-trnF	KU244952	FTOL v1.7
Dryopteridaceae	Polystichum sunhangii Liang Zhang, Yong L.Qiu & Li Bin	rbcL	OR543843	FTOL v1.7
Dryopteridaceae	Polystichum sunhangii Liang Zhang, Yong L.Qiu & Li Bin	rps4	OR562004	FTOL v1.7
Dryopteridaceae	Polystichum sunhangii Liang Zhang, Yong L.Qiu & Li Bin	rps4-trnS	OR562004	FTOL v1.7
Dryopteridaceae	Polystichum sunhangii Liang Zhang, Yong L.Qiu & Li Bin	trnL-trnF	OR543849	FTOL v1.7
Dryopteridaceae	Polystichum tagawanum Kurata	rbcL	AB575221	FTOL v1.7
Dryopteridaceae	Polystichum tagawanum Kurata	trnL-trnF	EF177302	FTOL v1.7
Dryopteridaceae	Polystichum talamancanum Barrington	rbcL	MW000306	FTOL v1.7
Dryopteridaceae	Polystichum talamancanum Barrington	rps4	MW000296	FTOL v1.7
Dryopteridaceae	Polystichum talamancanum Barrington	rps4-trnS	MW000272	FTOL v1.7
Dryopteridaceae	Polystichum talamancanum Barrington	trnL-trnF	MW000263	FTOL v1.7
Dryopteridaceae	Polystichum tenuius (Ching) Li Bing Zhang	rbcL	KU244705	FTOL v1.7
Dryopteridaceae	Polystichum tenuius (Ching) Li Bing Zhang	rps4-trnS	KU244865	FTOL v1.7
Dryopteridaceae	Polystichum tenuius (Ching) Li Bing Zhang	trnL-trnF	KU244949	FTOL v1.7
Dryopteridaceae	Polystichum tetragonum Fée	rbcL	MK174918	FTOL v1.7
Dryopteridaceae	Polystichum tetragonum Fée	rps4	MK214724	FTOL v1.7
Dryopteridaceae	Polystichum tetragonum Fée	rps4-trnS	MK214724	FTOL v1.7
Dryopteridaceae	Polystichum tetragonum Fée	trnL-trnF	MK035854	FTOL v1.7
Dryopteridaceae	Polystichum thomsonii (Hook. fil.) Bedd.	rbcL	KU244744	FTOL v1.7
Dryopteridaceae	Polystichum thomsonii (Hook. fil.) Bedd.	rps4-trnS	KU244916	FTOL v1.7
Dryopteridaceae	Polystichum thomsonii (Hook. fil.) Bedd.	trnL-trnF	KU245003	FTOL v1.7
Dryopteridaceae	Polystichum tiankengicola Li Bing Zhang, Q. Luo & P. S.	trnL-trnF	JF713062	FTOL v1.7
Dryopteridaceae	Polystichum tonkinense (Christ) W. M. Chu & Z. R. He	atpB	EF540728	FTOL v1.7
Dryopteridaceae	Polystichum tonkinense (Christ) W. M. Chu & Z. R. He	rbcL	EF540724	FTOL v1.7
Dryopteridaceae	Polystichum tonkinense (Christ) W. M. Chu & Z. R. He	rps4	EF540714	FTOL v1.7
Dryopteridaceae	Polystichum tonkinense (Christ) W. M. Chu & Z. R. He	rps4-trnS	EF540714	FTOL v1.7
Dryopteridaceae	Polystichum transkeiense W. Jacobsen	atpA	EF463724	FTOL v1.7
Dryopteridaceae	Polystichum transkeiense W. Jacobsen	atpB	EF463440	FTOL v1.7
Dryopteridaceae	Polystichum transkeiense W. Jacobsen	rbcL	AF537257	FTOL v1.7
Dryopteridaceae	Polystichum transkeiense W. Jacobsen	rps4	MK214725	FTOL v1.7
Dryopteridaceae	Polystichum transkeiense W. Jacobsen	rps4-trnS	MK214725	FTOL v1.7
Dryopteridaceae	Polystichum transkeiense W. Jacobsen	trnL-trnF	EF177297	FTOL v1.7
Dryopteridaceae	Polystichum tripteron (Kunze) C. Presl	plastome	MN712466	FTOL v1.7
Dryopteridaceae	Polystichum turrialbae Christ	rbcL	AF537259	FTOL v1.7
Dryopteridaceae	Polystichum underwoodii Maxon	rbcL	AF537260	FTOL v1.7
Dryopteridaceae	Polystichum underwoodii Maxon	rps4	KC890819	FTOL v1.7
Dryopteridaceae	Polystichum underwoodii Maxon	rps4-trnS	KC890819	FTOL v1.7
Dryopteridaceae	Polystichum underwoodii Maxon	trnL-trnF	KC878868	FTOL v1.7
Dryopteridaceae	Polystichum uniseriale (Ching ex K. H. Shing) Li Bing Zh	rbcL	AY694794	FTOL v1.7
Dryopteridaceae	Polystichum uniseriale (Ching ex K. H. Shing) Li Bing Zh	trnL-trnF	AY736330	FTOL v1.7
Dryopteridaceae	Polystichum vestitum (G. Forst.) C. Presl	rbcL	AY300099	FTOL v1.7
Dryopteridaceae	Polystichum wawranum (Szyszyl.) Perrie	trnL-trnF	KU245032	FTOL v1.7
Dryopteridaceae	Polystichum weimingii L. B. Zhang & H. He	rbcL	KU253818	FTOL v1.7
Dryopteridaceae	Polystichum weimingii L. B. Zhang & H. He	rps4-trnS	KU244909	FTOL v1.7
Dryopteridaceae	Polystichum weimingii L. B. Zhang & H. He	trnL-trnF	KU244996	FTOL v1.7

Dryopteridaceae	Polystichum whiteleggii Watts	rps4-trnS	AY164629	FTOL v1.7
Dryopteridaceae	Polystichum wusugongii Liang Zhang, X.M.Zhou & Li Bi	rbcL	OR543842	FTOL v1.7
Dryopteridaceae	Polystichum wusugongii Liang Zhang, X.M.Zhou & Li Bi	rps4	OR562008	FTOL v1.7
Dryopteridaceae	Polystichum wusugongii Liang Zhang, X.M.Zhou & Li Bi	rps4-trnS	OR562008	FTOL v1.7
Dryopteridaceae	Polystichum wusugongii Liang Zhang, X.M.Zhou & Li Bi	trnL-trnF	OR543850	FTOL v1.7
Dryopteridaceae	Polystichum xichouense (S. K. Wu & Mitsuta) Li Bing Zl	rbcL	DQ054515	FTOL v1.7
Dryopteridaceae	Polystichum xinfeniae Liang Zhang, Yong L.Qiu & Li Bin	rbcL	OR543840	FTOL v1.7
Dryopteridaceae	Polystichum xinfeniae Liang Zhang, Yong L.Qiu & Li Bin	rps4	OR562006	FTOL v1.7
Dryopteridaceae	Polystichum xinfeniae Liang Zhang, Yong L.Qiu & Li Bin	rps4-trnS	OR562006	FTOL v1.7
Dryopteridaceae	Polystichum xinfeniae Liang Zhang, Yong L.Qiu & Li Bin	trnL-trnF	OR543852	FTOL v1.7
Dryopteridaceae	Polystichum xiphophyllum (Baker) Diels	rbcL	KU244701	FTOL v1.7
Dryopteridaceae	Polystichum xiphophyllum (Baker) Diels	rps4-trnS	KU244855	FTOL v1.7
Dryopteridaceae	Polystichum xiphophyllum (Baker) Diels	trnL-trnF	KU244940	FTOL v1.7
Dryopteridaceae	Polystichum yaanense Liang Zhang & Li Bing Zhang	plastome	MT773344	FTOL v1.7
Dryopteridaceae	Polystichum yaeyamense (Makino) Makino	rbcL	AB575225	FTOL v1.7
Dryopteridaceae	Polystichum yuanum Ching	rbcL	KU244737	FTOL v1.7
Dryopteridaceae	Polystichum yuanum Ching	rps4-trnS	KU244907	FTOL v1.7
Dryopteridaceae	Polystichum yuanum Ching	trnL-trnF	KU244994	FTOL v1.7
Dryopteridaceae	Polystichum yunnanense Christ	atpA	EF463726	FTOL v1.7
Dryopteridaceae	Polystichum yunnanense Christ	atpB	EF463442	FTOL v1.7
Dryopteridaceae	Polystichum yunnanense Christ	rbcL	EF463220	FTOL v1.7
Dryopteridaceae	Rumohra adiantiformis (G. Forst.) Ching	atpA	MF158004	FTOL v1.7
Dryopteridaceae	Rumohra adiantiformis (G. Forst.) Ching	atpB	MF157746	FTOL v1.7
Dryopteridaceae	Rumohra adiantiformis (G. Forst.) Ching	rbcL	MF157818	FTOL v1.7
Dryopteridaceae	Rumohra adiantiformis (G. Forst.) Ching	rps4-trnS	MF157860	FTOL v1.7
Dryopteridaceae	Rumohra adiantiformis (G. Forst.) Ching	trnL-trnF	MF157966	FTOL v1.7
Dryopteridaceae	Rumohra berteroana (Colla) R. R. Rodriguez	atpA	MF158019	FTOL v1.7
Dryopteridaceae	Rumohra berteroana (Colla) R. R. Rodriguez	atpB	MF157762	FTOL v1.7
Dryopteridaceae	Rumohra berteroana (Colla) R. R. Rodriguez	rbcL	KJ464503	FTOL v1.7
Dryopteridaceae	Rumohra berteroana (Colla) R. R. Rodriguez	rps4-trnS	KJ464750	FTOL v1.7
Dryopteridaceae	Rumohra berteroana (Colla) R. R. Rodriguez	trnL-trnF	KJ464657	FTOL v1.7
Dryopteridaceae	Rumohra glandulosissima Sundue & J. Prado	atpA	MF158021	FTOL v1.7
Dryopteridaceae	Rumohra glandulosissima Sundue & J. Prado	atpB	MF157763	FTOL v1.7
Dryopteridaceae	Rumohra glandulosissima Sundue & J. Prado	rbcL	MF157838	FTOL v1.7
Dryopteridaceae	Rumohra glandulosissima Sundue & J. Prado	rps4-trnS	MF157877	FTOL v1.7
Dryopteridaceae	Rumohra glandulosissima Sundue & J. Prado	trnL-trnF	MF157986	FTOL v1.7
Dryopteridaceae	Rumohra linearisquamata Rakotondr.	atpA	MF158022	FTOL v1.7
Dryopteridaceae	Rumohra linearisquamata Rakotondr.	atpB	MF157764	FTOL v1.7
Dryopteridaceae	Rumohra linearisquamata Rakotondr.	rbcL	MF157839	FTOL v1.7
Dryopteridaceae	Rumohra linearisquamata Rakotondr.	rps4-trnS	MF157878	FTOL v1.7
Dryopteridaceae	Rumohra linearisquamata Rakotondr.	trnL-trnF	MF157987	FTOL v1.7
Dryopteridaceae	Rumohra lokohensis Tardieu	atpA	MF158024	FTOL v1.7
Dryopteridaceae	Rumohra lokohensis Tardieu	atpB	MF157767	FTOL v1.7
Dryopteridaceae	Rumohra lokohensis Tardieu	rbcL	MF157841	FTOL v1.7
Dryopteridaceae	Rumohra lokohensis Tardieu	rps4-trnS	MF157881	FTOL v1.7
Dryopteridaceae	Rumohra lokohensis Tardieu	trnL-trnF	MF157990	FTOL v1.7
Dryopteridaceae	Rumohra madagascarica (Bonap.) Tardieu	atpA	MF158031	FTOL v1.7
Dryopteridaceae	Rumohra madagascarica (Bonap.) Tardieu	atpB	MF157774	FTOL v1.7
Dryopteridaceae	Rumohra madagascarica (Bonap.) Tardieu	rbcL	MF157848	FTOL v1.7
Dryopteridaceae	Rumohra madagascarica (Bonap.) Tardieu	rps4-trnS	MF157888	FTOL v1.7
Dryopteridaceae	Rumohra madagascarica (Bonap.) Tardieu	trnL-trnF	MF157997	FTOL v1.7
Dryopteridaceae	Rumohra quadrangularis (Fée) Brade	atpA	MF158032	FTOL v1.7
Dryopteridaceae	Rumohra quadrangularis (Fée) Brade	atpB	MF157775	FTOL v1.7
Dryopteridaceae	Rumohra quadrangularis (Fée) Brade	rbcL	MF157849	FTOL v1.7
Dryopteridaceae	Rumohra quadrangularis (Fée) Brade	rps4-trnS	MF157889	FTOL v1.7
Dryopteridaceae	Rumohra quadrangularis (Fée) Brade	trnL-trnF	MF157998	FTOL v1.7
Dryopteridaceae	Stigmatopteris brevinervis (Fée) R. C. Moran	rbcL	KU521866	FTOL v1.7
Dryopteridaceae	Stigmatopteris brevinervis (Fée) R. C. Moran	rps4-trnS	KU521899	FTOL v1.7
Dryopteridaceae	Stigmatopteris brevinervis (Fée) R. C. Moran	trnL-trnF	KU521967	FTOL v1.7
Dryopteridaceae	Stigmatopteris bulbifera R. C. Moran	rbcL	KU521868	FTOL v1.7
Dryopteridaceae	Stigmatopteris bulbifera R. C. Moran	rps4-trnS	KU521901	FTOL v1.7
Dryopteridaceae	Stigmatopteris bulbifera R. C. Moran	trnL-trnF	KU521969	FTOL v1.7
Dryopteridaceae	Stigmatopteris caudata (Raddi) C. Chr.	rbcL	KU521872	FTOL v1.7
Dryopteridaceae	Stigmatopteris caudata (Raddi) C. Chr.	rps4-trnS	KU521905	FTOL v1.7
Dryopteridaceae	Stigmatopteris caudata (Raddi) C. Chr.	trnL-trnF	KU521973	FTOL v1.7
Dryopteridaceae	Stigmatopteris contracta (Christ) C. Chr.	rbcL	KU521874	FTOL v1.7
Dryopteridaceae	Stigmatopteris contracta (Christ) C. Chr.	rps4-trnS	KU521907	FTOL v1.7
Dryopteridaceae	Stigmatopteris contracta (Christ) C. Chr.	trnL-trnF	KU521975	FTOL v1.7
Dryopteridaceae	Stigmatopteris heterocarpa (Fée) Rosenst.	rbcL	KU521875	FTOL v1.7
Dryopteridaceae	Stigmatopteris heterocarpa (Fée) Rosenst.	rps4-trnS	KU521908	FTOL v1.7
Dryopteridaceae	Stigmatopteris heterocarpa (Fée) Rosenst.	trnL-trnF	KU521976	FTOL v1.7
Dryopteridaceae	Stigmatopteris heterophlebia (Baker) R. C. Moran	rbcL	KU521878	FTOL v1.7
Dryopteridaceae	Stigmatopteris heterophlebia (Baker) R. C. Moran	rps4-trnS	KU521911	FTOL v1.7
Dryopteridaceae	Stigmatopteris heterophlebia (Baker) R. C. Moran	trnL-trnF	KU521979	FTOL v1.7
Dryopteridaceae	Stigmatopteris ichthiosma (Sodiro) C.Chr.	rbcL	KU521881	FTOL v1.7
Dryopteridaceae	Stigmatopteris ichthiosma (Sodiro) C.Chr.	rps4-trnS	KU521914	FTOL v1.7
Dryopteridaceae	Stigmatopteris ichthiosma (Sodiro) C.Chr.	trnL-trnF	KU521982	FTOL v1.7
Dryopteridaceae	Stigmatopteris jamaicensis (Desv.) Proctor	rbcL	KU521884	FTOL v1.7
Dryopteridaceae	Stigmatopteris jamaicensis (Desv.) Proctor	rps4-trnS	KU521917	FTOL v1.7
Dryopteridaceae	Stigmatopteris jamaicensis (Desv.) Proctor	trnL-trnF	KU521985	FTOL v1.7
Dryopteridaceae	Stigmatopteris killipiana Lellinger	rbcL	KJ464505	FTOL v1.7
Dryopteridaceae	Stigmatopteris killipiana Lellinger	rps4-trnS	KU521918	FTOL v1.7
Dryopteridaceae	Stigmatopteris killipiana Lellinger	trnL-trnF	KU521986	FTOL v1.7
Dryopteridaceae	Stigmatopteris lechleri (Mett.) C. Chr.	atpA	EF463728	FTOL v1.7
Dryopteridaceae	Stigmatopteris lechleri (Mett.) C. Chr.	atpB	EF463444	FTOL v1.7
Dryopteridaceae	Stigmatopteris lechleri (Mett.) C. Chr.	rbcL	EF463221	FTOL v1.7
Dryopteridaceae	Stigmatopteris lechleri (Mett.) C. Chr.	rps4-trnS	KU521919	FTOL v1.7
Dryopteridaceae	Stigmatopteris lechleri (Mett.) C. Chr.	trnL-trnF	KU521987	FTOL v1.7
Dryopteridaceae	Stigmatopteris longicaudata (Liebm.) C. Chr.	atpA	EF463729	FTOL v1.7
Dryopteridaceae	Stigmatopteris longicaudata (Liebm.) C. Chr.	atpB	EF463445	FTOL v1.7
Dryopteridaceae	Stigmatopteris longicaudata (Liebm.) C. Chr.	rbcL	DQ508792	FTOL v1.7
Dryopteridaceae	Stigmatopteris longicaudata (Liebm.) C. Chr.	trnL-trnF	DQ514523	FTOL v1.7
Dryopteridaceae	Stigmatopteris michaelis (Baker) C. Chr.	rbcL	KU521889	FTOL v1.7
Dryopteridaceae	Stigmatopteris michaelis (Baker) C. Chr.	rps4-trnS	KU521922	FTOL v1.7
Dryopteridaceae	Stigmatopteris michaelis (Baker) C. Chr.	trnL-trnF	KU521988	FTOL v1.7
Dryopteridaceae	Stigmatopteris nephrodioides (Klotzsch) C. Chr.	rbcL	KU521890	FTOL v1.7
Dryopteridaceae	Stigmatopteris nephrodioides (Klotzsch) C. Chr.	rps4-trnS	KU521923	FTOL v1.7
Dryopteridaceae	Stigmatopteris nephrodioides (Klotzsch) C. Chr.	trnL-trnF	KU521989	FTOL v1.7
Dryopteridaceae	Stigmatopteris opaca (Baker) C. Chr.	rbcL	KU521892	FTOL v1.7
Dryopteridaceae	Stigmatopteris opaca (Baker) C. Chr.	rps4-trnS	KU521925	FTOL v1.7
Dryopteridaceae	Stigmatopteris opaca (Baker) C. Chr.	trnL-trnF	KU521991	FTOL v1.7
Dryopteridaceae	Stigmatopteris pellucidopunctata (C. Chr.) C. Chr.	rps4-trnS	KU521926	FTOL v1.7
Dryopteridaceae	Stigmatopteris pellucidopunctata (C. Chr.) C. Chr.	trnL-trnF	KU521992	FTOL v1.7
Dryopteridaceae	Stigmatopteris prionites (Kunze) C. Chr.	rbcL	KU521893	FTOL v1.7
Dryopteridaceae	Stigmatopteris prionites (Kunze) C. Chr.	rps4-trnS	GU376713	FTOL v1.7
Dryopteridaceae	Stigmatopteris prionites (Kunze) C. Chr.	trnL-trnF	GU376564	FTOL v1.7
Dryopteridaceae	Stigmatopteris pterorhachis R. C. Moran	rps4-trnS	KU521930	FTOL v1.7
Dryopteridaceae	Stigmatopteris rotundata (Humb. & Bonpl. Willd.) C. Cl	rps4-trnS	KU521931	FTOL v1.7
Dryopteridaceae	Stigmatopteris rotundata (Humb. & Bonpl. Willd.) C. Cl	trnL-trnF	KU521995	FTOL v1.7
Dryopteridaceae	Stigmatopteris sordida (Maxon) C. Chr.	rbcL	KJ464507	FTOL v1.7
Dryopteridaceae	Stigmatopteris sordida (Maxon) C. Chr.	rps4-trnS	KJ464754	FTOL v1.7
Dryopteridaceae	Stigmatopteris sordida (Maxon) C. Chr.	trnL-trnF	KJ464661	FTOL v1.7
Dryopteridaceae	Stigmatopteris tijuccana (Raddi) C. Chr.	rbcL	KU521895	FTOL v1.7
Dryopteridaceae	Stigmatopteris tijuccana (Raddi) C. Chr.	rps4-trnS	KU521932	FTOL v1.7
Dryopteridaceae	Stigmatopteris tijuccana (Raddi) C. Chr.	trnL-trnF	KU521996	FTOL v1.7
Dryopteridaceae	Stigmatopteris ulei (C. Chr.) Sehnem	rbcL	KU521897	FTOL v1.7
Dryopteridaceae	Stigmatopteris ulei (C. Chr.) Sehnem	rps4-trnS	KU521934	FTOL v1.7
Dryopteridaceae	Stigmatopteris ulei (C. Chr.) Sehnem	trnL-trnF	KU521998	FTOL v1.7
Dryopteridaceae	Teratophyllum koordersii Holttum	rps4-trnS	GU376715	FTOL v1.7
Dryopteridaceae	Teratophyllum koordersii Holttum	trnL-trnF	GU376566	FTOL v1.7
Dryopteridaceae	Teratophyllum ludens (Fée) Holttum	rps4-trnS	GU376717	FTOL v1.7
Dryopteridaceae	Teratophyllum ludens (Fée) Holttum	trnL-trnF	GU376568	FTOL v1.7
Dryopteridaceae	Teratophyllum rotundifoliatum (R. Bonap.) Holttum	rps4-trnS	GU376718	FTOL v1.7
Dryopteridaceae	Trichoneuron microlepioides Ching	atpA	KT831802	FTOL v1.7
Dryopteridaceae	Trichoneuron microlepioides Ching	atpB	KT831841	FTOL v1.7
Dryopteridaceae	Trichoneuron microlepioides Ching	rbcL	KT831884	FTOL v1.7
Hypodematiaceae	Hypodematium angustifolium C.S.Lee & K.Lee	atpB	MZ957914	FTOL v1.7
Hypodematiaceae	Hypodematium angustifolium C.S.Lee & K.Lee	rbcL	MZ957782	FTOL v1.7
Hypodematiaceae	Hypodematium angustifolium C.S.Lee & K.Lee	rps4	MZ957737	FTOL v1.7
Hypodematiaceae	Hypodematium angustifolium C.S.Lee & K.Lee	rps4-trnS	MZ957737	FTOL v1.7
Hypodematiaceae	Hypodematium angustifolium C.S.Lee & K.Lee	trnL-trnF	MZ957691	FTOL v1.7
Hypodematiaceae	Hypodematium boonkerdii Pongkai, Li Bing Zhang & Po	atpB	MZ957451	FTOL v1.7
Hypodematiaceae	Hypodematium boonkerdii Pongkai, Li Bing Zhang & Po	rbcL	MZ957219	FTOL v1.7
Hypodematiaceae	Hypodematium boonkerdii Pongkai, Li Bing Zhang & Po	rps4	MZ957668	FTOL v1.7
Hypodematiaceae	Hypodematium boonkerdii Pongkai, Li Bing Zhang & Po	rps4-trnS	MZ957668	FTOL v1.7
Hypodematiaceae	Hypodematium boonkerdii Pongkai, Li Bing Zhang & Po	trnL-trnF	MZ957341	FTOL v1.7
Hypodematiaceae	Hypodematium brevipilosum Li Bing Zhang, X. P. Fan &	atpB	MZ957089	FTOL v1.7
Hypodematiaceae	Hypodematium brevipilosum Li Bing Zhang, X. P. Fan &	rbcL	MZ957203	FTOL v1.7
Hypodematiaceae	Hypodematium brevipilosum Li Bing Zhang, X. P. Fan &	rps4	MZ957651	FTOL v1.7
Hypodematiaceae	Hypodematium brevipilosum Li Bing Zhang, X. P. Fan &	rps4-trnS	MZ957651	FTOL v1.7
Hypodematiaceae	Hypodematium brevipilosum Li Bing Zhang, X. P. Fan &	trnL-trnF	MZ957324	FTOL v1.7
Hypodematiaceae	Hypodematium chrysolepis (Fée) X. P. Fan & Li Bing Zh	plastome	MT130540	FTOL v1.7
Hypodematiaceae	Hypodematium confertivillosum J. X. Li, F. Q. Zhou & X.	atpB	MZ957059	FTOL v1.7
Hypodematiaceae	Hypodematium confertivillosum J. X. Li, F. Q. Zhou & X.	rbcL	MZ957168	FTOL v1.7
Hypodematiaceae	Hypodematium confertivillosum J. X. Li, F. Q. Zhou & X.	rps4	MZ957615	FTOL v1.7
Hypodematiaceae	Hypodematium confertivillosum J. X. Li, F. Q. Zhou & X.	rps4-trnS	MZ957615	FTOL v1.7
Hypodematiaceae	Hypodematium confertivillosum J. X. Li, F. Q. Zhou & X.	trnL-trnF	MZ957286	FTOL v1.7
Hypodematiaceae	Hypodematium crenatum subsp. fauriei (Kodama) K.Iw	rbcL	AB232414	FTOL v1.7
Hypodematiaceae	Hypodematium daochengense K. H. Shing	atpB	MZ957072	FTOL v1.7
Hypodematiaceae	Hypodematium daochengense K. H. Shing	rbcL	MZ957187	FTOL v1.7
Hypodematiaceae	Hypodematium daochengense K. H. Shing	rps4	MZ957634	FTOL v1.7
Hypodematiaceae	Hypodematium daochengense K. H. Shing	rps4-trnS	MZ957634	FTOL v1.7
Hypodematiaceae	Hypodematium daochengense K. H. Shing	trnL-trnF	MZ957306	FTOL v1.7
Hypodematiaceae	Hypodematium delicatulum Rakotondr.	atpB	MZ957066	FTOL v1.7
Hypodematiaceae	Hypodematium delicatulum Rakotondr.	rbcL	MZ957176	FTOL v1.7
Hypodematiaceae	Hypodematium delicatulum Rakotondr.	rps4	MZ957623	FTOL v1.7
Hypodematiaceae	Hypodematium delicatulum Rakotondr.	rps4-trnS	MZ957623	FTOL v1.7
Hypodematiaceae	Hypodematium delicatulum Rakotondr.	trnL-trnF	MZ957294	FTOL v1.7
Hypodematiaceae	Hypodematium eglandulosum X. P. Fan, Liang Zhang &	atpB	MZ957097	FTOL v1.7
Hypodematiaceae	Hypodematium eglandulosum X. P. Fan, Liang Zhang &	rbcL	MZ957211	FTOL v1.7
Hypodematiaceae	Hypodematium eglandulosum X. P. Fan, Liang Zhang &	rps4	MZ957659	FTOL v1.7
Hypodematiaceae	Hypodematium eglandulosum X. P. Fan, Liang Zhang &	rps4-trnS	MZ957659	FTOL v1.7
Hypodematiaceae	Hypodematium eglandulosum X. P. Fan, Liang Zhang &	trnL-trnF	MZ957332	FTOL v1.7
Hypodematiaceae	Hypodematium fordii (Baker) Ching	atpB	MZ957064	FTOL v1.7
Hypodematiaceae	Hypodematium fordii (Baker) Ching	rbcL	MZ957174	FTOL v1.7
Hypodematiaceae	Hypodematium fordii (Baker) Ching	rps4	MZ957621	FTOL v1.7
Hypodematiaceae	Hypodematium fordii (Baker) Ching	rps4-trnS	MZ957621	FTOL v1.7
Hypodematiaceae	Hypodematium fordii (Baker) Ching	trnL-trnF	MZ957292	FTOL v1.7
Hypodematiaceae	Hypodematium glabrum Ching ex K. H. Shing	atpB	MZ957091	FTOL v1.7
Hypodematiaceae	Hypodematium glabrum Ching ex K. H. Shing	rbcL	MZ957205	FTOL v1.7
Hypodematiaceae	Hypodematium glabrum Ching ex K. H. Shing	rps4	MZ957653	FTOL v1.7
Hypodematiaceae	Hypodematium glabrum Ching ex K. H. Shing	rps4-trnS	MZ957653	FTOL v1.7
Hypodematiaceae	Hypodematium glabrum Ching ex K. H. Shing	trnL-trnF	MZ957326	FTOL v1.7
Hypodematiaceae	Hypodematium glanduloso-pilosum (Tagawa) Ohwi	atpB	MZ957040	FTOL v1.7
Hypodematiaceae	Hypodematium glanduloso-pilosum (Tagawa) Ohwi	rbcL	MZ957148	FTOL v1.7
Hypodematiaceae	Hypodematium glanduloso-pilosum (Tagawa) Ohwi	rps4	MZ957596	FTOL v1.7
Hypodematiaceae	Hypodematium glanduloso-pilosum (Tagawa) Ohwi	rps4-trnS	MZ957596	FTOL v1.7
Hypodematiaceae	Hypodematium glanduloso-pilosum (Tagawa) Ohwi	trnL-trnF	MZ957266	FTOL v1.7
Hypodematiaceae	Hypodematium glandulosum Ching ex K. H. Shing	atpB	MZ957051	FTOL v1.7
Hypodematiaceae	Hypodematium glandulosum Ching ex K. H. Shing	rbcL	MZ957159	FTOL v1.7
Hypodematiaceae	Hypodematium glandulosum Ching ex K. H. Shing	rps4	MZ957606	FTOL v1.7
Hypodematiaceae	Hypodematium glandulosum Ching ex K. H. Shing	rps4-trnS	MZ957606	FTOL v1.7
Hypodematiaceae	Hypodematium glandulosum Ching ex K. H. Shing	trnL-trnF	MZ957277	FTOL v1.7
Hypodematiaceae	Hypodematium gracile Ching	atpB	MZ957057	FTOL v1.7
Hypodematiaceae	Hypodematium gracile Ching	rbcL	MZ957166	FTOL v1.7
Hypodematiaceae	Hypodematium gracile Ching	rps4	MZ957613	FTOL v1.7
Hypodematiaceae	Hypodematium gracile Ching	rps4-trnS	MZ957613	FTOL v1.7

Hypodematiacea	Hypodematium gracile Ching	trnL-trnF	MZ957284	FTOL v1.7
Hypodematiacea	Hypodematium hirsutum (D. Don) Ching	atpB	MZ957928	FTOL v1.7
Hypodematiacea	Hypodematium hirsutum (D. Don) Ching	rbcL	MZ957796	FTOL v1.7
Hypodematiacea	Hypodematium hirsutum (D. Don) Ching	rps4	MZ957751	FTOL v1.7
Hypodematiacea	Hypodematium hirsutum (D. Don) Ching	rps4-trnS	MZ957751	FTOL v1.7
Hypodematiacea	Hypodematium hirsutum (D. Don) Ching	trnL-trnF	MZ957705	FTOL v1.7
Hypodematiacea	Hypodematium parvisorum Li Bing Zhang, X. P. Fan &	atpB	MZ957094	FTOL v1.7
Hypodematiacea	Hypodematium parvisorum Li Bing Zhang, X. P. Fan &	rbcL	MZ957208	FTOL v1.7
Hypodematiacea	Hypodematium parvisorum Li Bing Zhang, X. P. Fan &	rps4	MZ957656	FTOL v1.7
Hypodematiacea	Hypodematium parvisorum Li Bing Zhang, X. P. Fan &	rps4-trnS	MZ957656	FTOL v1.7
Hypodematiacea	Hypodematium parvisorum Li Bing Zhang, X. P. Fan &	trnL-trnF	MZ957329	FTOL v1.7
Hypodematiacea	Hypodematium ryukyuense Li Bing Zhang, X. P. Fan &	atpB	MZ957049	FTOL v1.7
Hypodematiacea	Hypodematium ryukyuense Li Bing Zhang, X. P. Fan &	rbcL	MZ957157	FTOL v1.7
Hypodematiacea	Hypodematium ryukyuense Li Bing Zhang, X. P. Fan &	rps4	MZ957605	FTOL v1.7
Hypodematiacea	Hypodematium ryukyuense Li Bing Zhang, X. P. Fan &	rps4-trnS	MZ957605	FTOL v1.7
Hypodematiacea	Hypodematium ryukyuense Li Bing Zhang, X. P. Fan &	trnL-trnF	MZ957275	FTOL v1.7
Hypodematiacea	Hypodematium shingii Li Bing Zhang, X. P. Fan & X. F.	atpB	MZ957107	FTOL v1.7
Hypodematiacea	Hypodematium shingii Li Bing Zhang, X. P. Fan & X. F.	rbcL	MZ957221	FTOL v1.7
Hypodematiacea	Hypodematium shingii Li Bing Zhang, X. P. Fan & X. F.	rps4	MZ957671	FTOL v1.7
Hypodematiacea	Hypodematium shingii Li Bing Zhang, X. P. Fan & X. F.	rps4-trnS	MZ957671	FTOL v1.7
Hypodematiacea	Hypodematium shingii Li Bing Zhang, X. P. Fan & X. F.	trnL-trnF	MZ957344	FTOL v1.7
Hypodematiacea	Hypodematium sinense K. Iwats.	atpB	MZ957923	FTOL v1.7
Hypodematiacea	Hypodematium sinense K. Iwats.	rbcL	MZ957791	FTOL v1.7
Hypodematiacea	Hypodematium sinense K. Iwats.	rps4	MZ957746	FTOL v1.7
Hypodematiacea	Hypodematium sinense K. Iwats.	rps4-trnS	MZ957746	FTOL v1.7
Hypodematiacea	Hypodematium sinense K. Iwats.	trnL-trnF	MZ957700	FTOL v1.7
Hypodematiacea	Hypodematium squamulosopilosum Ching	atpB	MZ957920	FTOL v1.7
Hypodematiacea	Hypodematium squamulosopilosum Ching	rbcL	MZ957788	FTOL v1.7
Hypodematiacea	Hypodematium squamulosopilosum Ching	rps4	MZ957743	FTOL v1.7
Hypodematiacea	Hypodematium squamulosopilosum Ching	rps4-trnS	MZ957743	FTOL v1.7
Hypodematiacea	Hypodematium squamulosopilosum Ching	trnL-trnF	MZ957697	FTOL v1.7
Hypodematiacea	Hypodematium subglabrum Li Bing Zhang, X. P. Fan &	atpB	MZ957093	FTOL v1.7
Hypodematiacea	Hypodematium subglabrum Li Bing Zhang, X. P. Fan &	rbcL	MZ957207	FTOL v1.7
Hypodematiacea	Hypodematium subglabrum Li Bing Zhang, X. P. Fan &	rps4	MZ957655	FTOL v1.7
Hypodematiacea	Hypodematium subglabrum Li Bing Zhang, X. P. Fan &	rps4-trnS	MZ957655	FTOL v1.7
Hypodematiacea	Hypodematium subglabrum Li Bing Zhang, X. P. Fan &	trnL-trnF	MZ957328	FTOL v1.7
Hypodematiacea	Hypodematium truncatum Li Bing Zhang, X. P. Fan & X	atpB	MZ957090	FTOL v1.7
Hypodematiacea	Hypodematium truncatum Li Bing Zhang, X. P. Fan & X	rbcL	MZ957204	FTOL v1.7
Hypodematiacea	Hypodematium truncatum Li Bing Zhang, X. P. Fan & X	rps4	MZ957652	FTOL v1.7
Hypodematiacea	Hypodematium truncatum Li Bing Zhang, X. P. Fan & X	rps4-trnS	MZ957652	FTOL v1.7
Hypodematiacea	Hypodematium truncatum Li Bing Zhang, X. P. Fan & X	trnL-trnF	MZ957325	FTOL v1.7
Hypodematiacea	Hypodematium villosum F. G. Wang & F. W. Xing	atpB	MZ957054	FTOL v1.7
Hypodematiacea	Hypodematium villosum F. G. Wang & F. W. Xing	rbcL	MZ957162	FTOL v1.7
Hypodematiacea	Hypodematium villosum F. G. Wang & F. W. Xing	rps4	MZ957609	FTOL v1.7
Hypodematiacea	Hypodematium villosum F. G. Wang & F. W. Xing	rps4-trnS	MZ957609	FTOL v1.7
Hypodematiacea	Hypodematium villosum F. G. Wang & F. W. Xing	trnL-trnF	MZ957280	FTOL v1.7
Hypodematiacea	Leucostegia amplissima (Christ) C.W.Chen	atpB	MZ957032	FTOL v1.7
Hypodematiacea	Leucostegia amplissima (Christ) C.W.Chen	rbcL	MZ957135	FTOL v1.7
Hypodematiacea	Leucostegia amplissima (Christ) C.W.Chen	rps4	MZ957583	FTOL v1.7
Hypodematiacea	Leucostegia amplissima (Christ) C.W.Chen	rps4-trnS	MZ957583	FTOL v1.7
Hypodematiacea	Leucostegia amplissima (Christ) C.W.Chen	trnL-trnF	MZ957253	FTOL v1.7
Hypodematiacea	Leucostegia pallida (Mett. ex Kuhn) Copel.	atpB	MZ957368	FTOL v1.7
Hypodematiacea	Leucostegia pallida (Mett. ex Kuhn) Copel.	rbcL	MZ957134	FTOL v1.7
Hypodematiacea	Leucostegia pallida (Mett. ex Kuhn) Copel.	rps4	MZ957582	FTOL v1.7
Hypodematiacea	Leucostegia pallida (Mett. ex Kuhn) Copel.	rps4-trnS	MZ957582	FTOL v1.7
Hypodematiacea	Leucostegia pallida (Mett. ex Kuhn) Copel.	trnL-trnF	MZ957252	FTOL v1.7
Hypodematiacea	Leucostegia truncata (D. Don) Fraser-Jenk.	plastome	MT130653	FTOL v1.7
Lomariopsidacea	Cyclopeltis crenata (Fée) C. Chr.	plastome	MT130541	FTOL v1.7
Lomariopsidacea	Cyclopeltis novoguineensis Rosenst.	rbcL	KY397974	FTOL v1.7
Lomariopsidacea	Cyclopeltis novoguineensis Rosenst.	rps4	KY397978	FTOL v1.7
Lomariopsidacea	Cyclopeltis novoguineensis Rosenst.	rps4-trnS	KY397978	FTOL v1.7
Lomariopsidacea	Cyclopeltis novoguineensis Rosenst.	trnL-trnF	KY397970	FTOL v1.7
Lomariopsidacea	Cyclopeltis semicordata (Sw.) J. Sm.	atpA	JF832107	FTOL v1.7
Lomariopsidacea	Cyclopeltis semicordata (Sw.) J. Sm.	atpB	EF463480	FTOL v1.7
Lomariopsidacea	Cyclopeltis semicordata (Sw.) J. Sm.	rbcL	EF463234	FTOL v1.7
Lomariopsidacea	Cyclopeltis semicordata (Sw.) J. Sm.	rps4	KY397977	FTOL v1.7
Lomariopsidacea	Cyclopeltis semicordata (Sw.) J. Sm.	rps4-trnS	KY397977	FTOL v1.7
Lomariopsidacea	Cyclopeltis semicordata (Sw.) J. Sm.	trnL-trnF	KY397969	FTOL v1.7
Lomariopsidacea	Dracoglossum plantagineum (Jacq.) Christenh.	atpA	KC914562	FTOL v1.7
Lomariopsidacea	Dracoglossum plantagineum (Jacq.) Christenh.	atpB	KC914563	FTOL v1.7
Lomariopsidacea	Dracoglossum plantagineum (Jacq.) Christenh.	rbcL	KC914564	FTOL v1.7
Lomariopsidacea	Dracoglossum plantagineum (Jacq.) Christenh.	rps4	KC914565	FTOL v1.7
Lomariopsidacea	Dracoglossum plantagineum (Jacq.) Christenh.	rps4-trnS	KC914565	FTOL v1.7
Lomariopsidacea	Dracoglossum sinuatum (Fée) Christenh.	atpA	OR087148	FTOL v1.7
Lomariopsidacea	Dracoglossum sinuatum (Fée) Christenh.	rbcL	OR087014	FTOL v1.7
Lomariopsidacea	Dryopolystichum phaeostigma (Ces.) Copel.	rbcL	KY397972	FTOL v1.7
Lomariopsidacea	Dryopolystichum phaeostigma (Ces.) Copel.	rps4	KY397976	FTOL v1.7
Lomariopsidacea	Dryopolystichum phaeostigma (Ces.) Copel.	rps4-trnS	KY397976	FTOL v1.7
Lomariopsidacea	Dryopolystichum phaeostigma (Ces.) Copel.	trnL-trnF	KY397968	FTOL v1.7
Lomariopsidacea	Lomariopsis amydrophlebia (Sloss. ex Maxon) Holttum	trnL-trnF	DQ396555	FTOL v1.7
Lomariopsidacea	Lomariopsis boivinii Holttum	trnL-trnF	DQ396557	FTOL v1.7
Lomariopsidacea	Lomariopsis boninensis Nakai	rbcL	OL420738	FTOL v1.7
Lomariopsidacea	Lomariopsis boninensis Nakai	rps4	OL473645	FTOL v1.7
Lomariopsidacea	Lomariopsis boninensis Nakai	rps4-trnS	OL473645	FTOL v1.7
Lomariopsidacea	Lomariopsis boninensis Nakai	trnL-trnF	OL473669	FTOL v1.7
Lomariopsidacea	Lomariopsis brackenridgei Carruth.	rbcL	OL420741	FTOL v1.7
Lomariopsidacea	Lomariopsis brackenridgei Carruth.	rps4	OL473649	FTOL v1.7
Lomariopsidacea	Lomariopsis brackenridgei Carruth.	rps4-trnS	OL473649	FTOL v1.7
Lomariopsidacea	Lomariopsis brackenridgei Carruth.	trnL-trnF	OL473673	FTOL v1.7
Lomariopsidacea	Lomariopsis cordata (Bonap.) Alston	trnL-trnF	DQ396558	FTOL v1.7
Lomariopsidacea	Lomariopsis crassifolia Holttum	trnL-trnF	DQ396559	FTOL v1.7
Lomariopsidacea	Lomariopsis fendleri D. C. Eaton	atpB	MN781292	FTOL v1.7
Lomariopsidacea	Lomariopsis fendleri D. C. Eaton	rbcL	MN781336	FTOL v1.7
Lomariopsidacea	Lomariopsis fendleri D. C. Eaton	rps4	MN781376	FTOL v1.7
Lomariopsidacea	Lomariopsis fendleri D. C. Eaton	rps4-trnS	MN781376	FTOL v1.7
Lomariopsidacea	Lomariopsis guineensis (Underw.) Alston	trnL-trnF	DQ396560	FTOL v1.7
Lomariopsidacea	Lomariopsis hederacea Alston	trnL-trnF	DQ396561	FTOL v1.7
Lomariopsidacea	Lomariopsis jamaicensis (Underw.) Holttum	trnL-trnF	DQ396562	FTOL v1.7
Lomariopsidacea	Lomariopsis japurensis (Mart.) J. Sm.	plastome	MK705752	FTOL v1.7
Lomariopsidacea	Lomariopsis kingii (Copel.) Holttum	rbcL	OL420744	FTOL v1.7
Lomariopsidacea	Lomariopsis kingii (Copel.) Holttum	rps4	OL473650	FTOL v1.7
Lomariopsidacea	Lomariopsis kingii (Copel.) Holttum	rps4-trnS	OL473650	FTOL v1.7
Lomariopsidacea	Lomariopsis kingii (Copel.) Holttum	trnL-trnF	OL473676	FTOL v1.7
Lomariopsidacea	Lomariopsis kunzeana (C. Presl ex Underw.) Holttum	trnL-trnF	DQ396569	FTOL v1.7
Lomariopsidacea	Lomariopsis latipinna Stolze	atpB	MN781293	FTOL v1.7
Lomariopsidacea	Lomariopsis latipinna Stolze	rbcL	MN781337	FTOL v1.7
Lomariopsidacea	Lomariopsis latipinna Stolze	rps4	MN781377	FTOL v1.7
Lomariopsidacea	Lomariopsis latipinna Stolze	rps4-trnS	MN781377	FTOL v1.7
Lomariopsidacea	Lomariopsis leptocarpa Fée	rbcL	OL420745	FTOL v1.7
Lomariopsidacea	Lomariopsis leptocarpa Fée	rps4	OL473652	FTOL v1.7
Lomariopsidacea	Lomariopsis leptocarpa Fée	rps4-trnS	OL473652	FTOL v1.7
Lomariopsidacea	Lomariopsis leptocarpa Fée	trnL-trnF	OL473677	FTOL v1.7
Lomariopsidacea	Lomariopsis lineata (C. Presl) Holttum	plastome	MT130608	FTOL v1.7
Lomariopsidacea	Lomariopsis longicaudata (Bonap.) Holttum	trnL-trnF	DQ396574	FTOL v1.7
Lomariopsidacea	Lomariopsis longini L.Y.Kuo & Y.H.Wu	rbcL	OL420749	FTOL v1.7
Lomariopsidacea	Lomariopsis longini L.Y.Kuo & Y.H.Wu	rps4	OL473658	FTOL v1.7
Lomariopsidacea	Lomariopsis longini L.Y.Kuo & Y.H.Wu	rps4-trnS	OL473658	FTOL v1.7
Lomariopsidacea	Lomariopsis longini L.Y.Kuo & Y.H.Wu	trnL-trnF	OL473684	FTOL v1.7
Lomariopsidacea	Lomariopsis madagascarica (Bonap.) Alston	trnL-trnF	DQ396576	FTOL v1.7
Lomariopsidacea	Lomariopsis mannii (Underw.) Alston	trnL-trnF	DQ396577	FTOL v1.7
Lomariopsidacea	Lomariopsis marginata (Schrad.) Kuhn	rbcL	AY818677	FTOL v1.7
Lomariopsidacea	Lomariopsis marginata (Schrad.) Kuhn	rps4-trnS	AY540049	FTOL v1.7
Lomariopsidacea	Lomariopsis maxonii (Underw.) Holttum	rbcL	MW138155	FTOL v1.7
Lomariopsidacea	Lomariopsis moorei Y.H.Wu & L.Y.Kuo	rbcL	OL420753	FTOL v1.7
Lomariopsidacea	Lomariopsis moorei Y.H.Wu & L.Y.Kuo	rps4	OL473662	FTOL v1.7
Lomariopsidacea	Lomariopsis moorei Y.H.Wu & L.Y.Kuo	rps4-trnS	OL473662	FTOL v1.7
Lomariopsidacea	Lomariopsis moorei Y.H.Wu & L.Y.Kuo	trnL-trnF	OL473691	FTOL v1.7
Lomariopsidacea	Lomariopsis muriculata Holttum	trnL-trnF	DQ396582	FTOL v1.7
Lomariopsidacea	Lomariopsis nigropaleata Holttum	atpB	MN781294	FTOL v1.7
Lomariopsidacea	Lomariopsis nigropaleata Holttum	rbcL	MN781334	FTOL v1.7
Lomariopsidacea	Lomariopsis nigropaleata Holttum	rps4	MN781378	FTOL v1.7
Lomariopsidacea	Lomariopsis nigropaleata Holttum	rps4-trnS	MN781378	FTOL v1.7
Lomariopsidacea	Lomariopsis nigropaleata Holttum	trnL-trnF	DQ396584	FTOL v1.7
Lomariopsidacea	Lomariopsis novae-caledoniae Mett.	rps4	OL473655	FTOL v1.7
Lomariopsidacea	Lomariopsis novae-caledoniae Mett.	rps4-trnS	OL473655	FTOL v1.7
Lomariopsidacea	Lomariopsis novae-caledoniae Mett.	trnL-trnF	OL473681	FTOL v1.7
Lomariopsidacea	Lomariopsis oleandrifolia Mett. ex Kuhn	rbcL	MT657693	FTOL v1.7
Lomariopsidacea	Lomariopsis palustris (Hook.) Mett.	trnL-trnF	DQ396585	FTOL v1.7
Lomariopsidacea	Lomariopsis pervillei Mett. ex Kuhn	trnL-trnF	DQ396587	FTOL v1.7
Lomariopsidacea	Lomariopsis pollicina (Willemet) Mett. ex Kuhn	atpA	EF463776	FTOL v1.7
Lomariopsidacea	Lomariopsis pollicina (Willemet) Mett. ex Kuhn	atpB	EF463481	FTOL v1.7
Lomariopsidacea	Lomariopsis pollicina (Willemet) Mett. ex Kuhn	rbcL	EF463235	FTOL v1.7
Lomariopsidacea	Lomariopsis pollicina (Willemet) Mett. ex Kuhn	trnL-trnF	DQ396588	FTOL v1.7
Lomariopsidacea	Lomariopsis prieuriana Fée	rbcL	KJ628765	FTOL v1.7
Lomariopsidacea	Lomariopsis recurvata Fée	trnL-trnF	DQ396592	FTOL v1.7
Lomariopsidacea	Lomariopsis rossii Holttum	trnL-trnF	DQ396594	FTOL v1.7
Lomariopsidacea	Lomariopsis salicifolia (Kunze) Lellinger	trnL-trnF	DQ396596	FTOL v1.7
Lomariopsidacea	Lomariopsis sorbifolia (L.) Fée	atpA	EF463777	FTOL v1.7
Lomariopsidacea	Lomariopsis sorbifolia (L.) Fée	atpB	EF463482	FTOL v1.7
Lomariopsidacea	Lomariopsis sorbifolia (L.) Fée	rbcL	EF463236	FTOL v1.7
Lomariopsidacea	Lomariopsis sorbifolia (L.) Fée	rps4	OL473656	FTOL v1.7
Lomariopsidacea	Lomariopsis sorbifolia (L.) Fée	rps4-trnS	OL473656	FTOL v1.7
Lomariopsidacea	Lomariopsis sorbifolia (L.) Fée	trnL-trnF	OL473682	FTOL v1.7
Lomariopsidacea	Lomariopsis spectabilis (Kunze) Mett.	atpB	KJ196492	FTOL v1.7
Lomariopsidacea	Lomariopsis spectabilis (Kunze) Mett.	rbcL	KJ196811	FTOL v1.7
Lomariopsidacea	Lomariopsis spectabilis (Kunze) Mett.	trnL-trnF	KJ196685	FTOL v1.7
Lomariopsidacea	Lomariopsis vestita E. Fourn.	rbcL	KJ628767	FTOL v1.7
Lomariopsidacea	Lomariopsis wrightii Mett ex D. C. Eaton	trnL-trnF	DQ396600	FTOL v1.7
Nephrolepidacea	Nephrolepis abrupta (Bory) Mett.	rbcL	HM748138	FTOL v1.7
Nephrolepidacea	Nephrolepis abrupta (Bory) Mett.	rps4	HM748164	FTOL v1.7
Nephrolepidacea	Nephrolepis abrupta (Bory) Mett.	rps4-trnS	HM748164	FTOL v1.7
Nephrolepidacea	Nephrolepis acutifolia (Desv.) Christ	rbcL	HM748139	FTOL v1.7
Nephrolepidacea	Nephrolepis acutifolia (Desv.) Christ	rps4	HM748165	FTOL v1.7
Nephrolepidacea	Nephrolepis acutifolia (Desv.) Christ	rps4-trnS	HM748165	FTOL v1.7
Nephrolepidacea	Nephrolepis biserrata (Sw.) Schott	plastome	MT130615	FTOL v1.7
Nephrolepidacea	Nephrolepis brownii (Desv.) Hovenkamp & Miyam.	rbcL	KR816691	FTOL v1.7
Nephrolepidacea	Nephrolepis cordifolia (L.) C. Presl	plastome	OL876381	FTOL v1.7
Nephrolepidacea	Nephrolepis davalliae Alderw.	rbcL	HM748147	FTOL v1.7
Nephrolepidacea	Nephrolepis davalliae Alderw.	rps4	HM748174	FTOL v1.7
Nephrolepidacea	Nephrolepis davalliae Alderw.	rps4-trnS	HM748174	FTOL v1.7
Nephrolepidacea	Nephrolepis davallioides (Sw.) Kunze	atpA	JF832131	FTOL v1.7
Nephrolepidacea	Nephrolepis davallioides (Sw.) Kunze	atpB	JF832172	FTOL v1.7
Nephrolepidacea	Nephrolepis davallioides (Sw.) Kunze	rbcL	JF832075	FTOL v1.7
Nephrolepidacea	Nephrolepis exaltata (L.) Schott	rbcL	HM748149	FTOL v1.7
Nephrolepidacea	Nephrolepis exaltata (L.) Schott	rps4	HM748176	FTOL v1.7
Nephrolepidacea	Nephrolepis exaltata (L.) Schott	rps4-trnS	HM748176	FTOL v1.7
Nephrolepidacea	Nephrolepis falcata (Cav.) C. Chr.	atpB	DQ646105	FTOL v1.7
Nephrolepidacea	Nephrolepis falcata (Cav.) C. Chr.	rbcL	HM748150	FTOL v1.7
Nephrolepidacea	Nephrolepis falcata (Cav.) C. Chr.	rps4	HM748177	FTOL v1.7
Nephrolepidacea	Nephrolepis falcata (Cav.) C. Chr.	rps4-trnS	HM748177	FTOL v1.7
Nephrolepidacea	Nephrolepis falciformis J. Sm.	rbcL	AB232404	FTOL v1.7
Nephrolepidacea	Nephrolepis falciformis J. Sm.	rps4	HM748178	FTOL v1.7
Nephrolepidacea	Nephrolepis falciformis J. Sm.	rps4-trnS	HM748178	FTOL v1.7
Nephrolepidacea	Nephrolepis flexuosa Colenso	rbcL	MK959215	FTOL v1.7
Nephrolepidacea	Nephrolepis hirsutula (G. Forst.) C. Presl	atpA	EF463778	FTOL v1.7
Nephrolepidacea	Nephrolepis hirsutula (G. Forst.) C. Presl	atpB	EF463483	FTOL v1.7
Nephrolepidacea	Nephrolepis hirsutula (G. Forst.) C. Presl	rbcL	EF463237	FTOL v1.7

Nephrolepidacea	Nephrolepis lauterbachii (Christ) Christ	rbcL	HM748153	FTOL v1.7
Nephrolepidacea	Nephrolepis lauterbachii (Christ) Christ	rps4	HM748180	FTOL v1.7
Nephrolepidacea	Nephrolepis lauterbachii (Christ) Christ	rps4-trnS	HM748180	FTOL v1.7
Nephrolepidacea	Nephrolepis obliterata (R. Br.) J. Sm.	rbcL	MT657675	FTOL v1.7
Nephrolepidacea	Nephrolepis pectinata (Willd.) Schott	rbcL	MW138231	FTOL v1.7
Nephrolepidacea	Nephrolepis pectinata (Willd.) Schott	rps4	HM748181	FTOL v1.7
Nephrolepidacea	Nephrolepis pectinata (Willd.) Schott	rps4-trnS	HM748181	FTOL v1.7
Nephrolepidacea	Nephrolepis pendula (Raddi) J. Sm.	rbcL	HM748156	FTOL v1.7
Nephrolepidacea	Nephrolepis pendula (Raddi) J. Sm.	rps4	HM748182	FTOL v1.7
Nephrolepidacea	Nephrolepis pendula (Raddi) J. Sm.	rps4-trnS	HM748182	FTOL v1.7
Nephrolepidacea	Nephrolepis radicans (Burm.) Kuhn	rbcL	HM748157	FTOL v1.7
Nephrolepidacea	Nephrolepis radicans (Burm.) Kuhn	rps4	HM748183	FTOL v1.7
Nephrolepidacea	Nephrolepis radicans (Burm.) Kuhn	rps4-trnS	HM748183	FTOL v1.7
Nephrolepidacea	Nephrolepis rivularis (Vahl) Mett. ex Krug	rbcL	HM748158	FTOL v1.7
Nephrolepidacea	Nephrolepis rivularis (Vahl) Mett. ex Krug	rps4	HM748184	FTOL v1.7
Nephrolepidacea	Nephrolepis rivularis (Vahl) Mett. ex Krug	rps4-trnS	HM748184	FTOL v1.7
Nephrolepidacea	Nephrolepis undulata (Afzel. ex Sw.) J. Sm.	rbcL	HM748159	FTOL v1.7
Nephrolepidacea	Nephrolepis undulata (Afzel. ex Sw.) J. Sm.	rps4	HM748185	FTOL v1.7
Nephrolepidacea	Nephrolepis undulata (Afzel. ex Sw.) J. Sm.	rps4-trnS	HM748186	FTOL v1.7
Oleandraceae	Oleandra articulata (Sw.) C. Presl	plastome	MK705754	FTOL v1.7
Oleandraceae	Oleandra cumingii J. Sm.	atpB	KJ196497	FTOL v1.7
Oleandraceae	Oleandra cumingii J. Sm.	rbcL	KJ196816	FTOL v1.7
Oleandraceae	Oleandra cumingii J. Sm.	trnL-trnF	KJ196690	FTOL v1.7
Oleandraceae	Oleandra decurrens Maxon	rbcL	MW138210	FTOL v1.7
Oleandraceae	Oleandra distenta Kunze	rbcL	KF992505	FTOL v1.7
Oleandraceae	Oleandra musifolia (Blume) C. Presl	atpB	EF450536	FTOL v1.7
Oleandraceae	Oleandra neriiformis Cav.	atpB	KJ196496	FTOL v1.7
Oleandraceae	Oleandra neriiformis Cav.	rbcL	KJ196815	FTOL v1.7
Oleandraceae	Oleandra neriiformis Cav.	trnL-trnF	KJ196689	FTOL v1.7
Oleandraceae	Oleandra pilosa Hook.	rbcL	KF667646	FTOL v1.7
Oleandraceae	Oleandra pilosa Hook.	rps4-trnS	KF667559	FTOL v1.7
Oleandraceae	Oleandra pilosa Hook.	trnL-trnF	KF667615	FTOL v1.7
Oleandraceae	Oleandra pistillaris (Sw.) C. Chr.	rbcL	AB232405	FTOL v1.7
Oleandraceae	Oleandra sibbaldii Grev.	rbcL	MT216070	FTOL v1.7
Oleandraceae	Oleandra wallichii (Hook.) C. Presl	plastome	MT130650	FTOL v1.7
Polypodiaceae	Adenophorus abietinus (D. C. Eaton) K. A. Wilson	atpB	AF469778	FTOL v1.7
Polypodiaceae	Adenophorus abietinus (D. C. Eaton) K. A. Wilson	rbcL	AF468202	FTOL v1.7
Polypodiaceae	Adenophorus abietinus (D. C. Eaton) K. A. Wilson	trnL-trnF	AF469791	FTOL v1.7
Polypodiaceae	Adenophorus epigaeus (L. E. Bishop) W. H. Wagner	atpB	AF469779	FTOL v1.7
Polypodiaceae	Adenophorus epigaeus (L. E. Bishop) W. H. Wagner	rbcL	AF468203	FTOL v1.7
Polypodiaceae	Adenophorus epigaeus (L. E. Bishop) W. H. Wagner	trnL-trnF	AF469792	FTOL v1.7
Polypodiaceae	Adenophorus haaliloanus (Brack.) K.A.Wilson	atpB	AF469775	FTOL v1.7
Polypodiaceae	Adenophorus haaliloanus (Brack.) K.A.Wilson	rbcL	AF468200	FTOL v1.7
Polypodiaceae	Adenophorus haaliloanus (Brack.) K.A.Wilson	trnL-trnF	AF469788	FTOL v1.7
Polypodiaceae	Adenophorus hymenophylloides (Kaulf.) Hook. & Grev.	atpB	AF469780	FTOL v1.7
Polypodiaceae	Adenophorus hymenophylloides (Kaulf.) Hook. & Grev.	rbcL	AF468204	FTOL v1.7
Polypodiaceae	Adenophorus hymenophylloides (Kaulf.) Hook. & Grev.	trnL-trnF	AF469793	FTOL v1.7
Polypodiaceae	Adenophorus oahuensis (Copel.) L. E. Bishop	atpB	AF469776	FTOL v1.7
Polypodiaceae	Adenophorus oahuensis (Copel.) L. E. Bishop	rbcL	AY057382	FTOL v1.7
Polypodiaceae	Adenophorus oahuensis (Copel.) L. E. Bishop	trnL-trnF	AF469789	FTOL v1.7
Polypodiaceae	Adenophorus periens L. E. Bishop	atpB	AF469774	FTOL v1.7
Polypodiaceae	Adenophorus periens L. E. Bishop	rbcL	AF468199	FTOL v1.7
Polypodiaceae	Adenophorus periens L. E. Bishop	trnL-trnF	AF469787	FTOL v1.7
Polypodiaceae	Adenophorus sarmentosus (Brack.) K. A. Wilson	atpA	EF463798	FTOL v1.7
Polypodiaceae	Adenophorus sarmentosus (Brack.) K. A. Wilson	atpB	AF469777	FTOL v1.7
Polypodiaceae	Adenophorus sarmentosus (Brack.) K. A. Wilson	rbcL	AF468201	FTOL v1.7
Polypodiaceae	Adenophorus sarmentosus (Brack.) K. A. Wilson	trnL-trnF	AF469790	FTOL v1.7
Polypodiaceae	Adenophorus tamariscinus var. montanus (Hillebr.) Bis	atpB	AF469781	FTOL v1.7
Polypodiaceae	Adenophorus tamariscinus var. montanus (Hillebr.) Bis	rbcL	AF468205	FTOL v1.7
Polypodiaceae	Adenophorus tamariscinus var. montanus (Hillebr.) Bis	trnL-trnF	AF469794	FTOL v1.7
Polypodiaceae	Adenophorus tenellus (Kaulf.) Ranker	atpA	EF463820	FTOL v1.7
Polypodiaceae	Adenophorus tenellus (Kaulf.) Ranker	atpB	AF469773	FTOL v1.7
Polypodiaceae	Adenophorus tenellus (Kaulf.) Ranker	rbcL	AF468198	FTOL v1.7
Polypodiaceae	Adenophorus tenellus (Kaulf.) Ranker	trnL-trnF	AF469786	FTOL v1.7
Polypodiaceae	Adenophorus tripinnatifidus Gaudich.	atpB	AF469783	FTOL v1.7
Polypodiaceae	Adenophorus tripinnatifidus Gaudich.	rbcL	AF468207	FTOL v1.7
Polypodiaceae	Adenophorus tripinnatifidus Gaudich.	trnL-trnF	AF469796	FTOL v1.7
Polypodiaceae	Adetogramma chrysolepis (Hook.) T. E. Almeida	plastome	MW876307	FTOL v1.7
Polypodiaceae	Aenigmatogrammitis stenophylla (Parris) Parris	atpB	JX499239	FTOL v1.7
Polypodiaceae	Aenigmatogrammitis stenophylla (Parris) Parris	rbcL	JQ904084	FTOL v1.7
Polypodiaceae	Aenigmatogrammitis stenophylla (Parris) Parris	trnL-trnF	JQ911714	FTOL v1.7
Polypodiaceae	Alansmia cultrata (Bory ex Willd.) Moguel & M. Kessle	atpB	GU376476	FTOL v1.7
Polypodiaceae	Alansmia cultrata (Bory ex Willd.) Moguel & M. Kessle	rbcL	GU376496	FTOL v1.7
Polypodiaceae	Alansmia cultrata (Bory ex Willd.) Moguel & M. Kessle	rps4-trnS	JN654943	FTOL v1.7
Polypodiaceae	Alansmia cultrata (Bory ex Willd.) Moguel & M. Kessle	trnL-trnF	JN654969	FTOL v1.7
Polypodiaceae	Alansmia elastica (Bory ex Willd.) Moguel & M. Kessle	atpB	KY711748	FTOL v1.7
Polypodiaceae	Alansmia elastica (Bory ex Willd.) Moguel & M. Kessle	rbcL	KY711922	FTOL v1.7
Polypodiaceae	Alansmia elastica (Bory ex Willd.) Moguel & M. Kessle	rps4-trnS	KY712249	FTOL v1.7
Polypodiaceae	Alansmia elastica (Bory ex Willd.) Moguel & M. Kessle	trnL-trnF	KY711575	FTOL v1.7
Polypodiaceae	Alansmia heteromorpha (Hook. & Grev.) Moguel & M.	rbcL	KM218803	FTOL v1.7
Polypodiaceae	Alansmia lanigera (Desv.) Moguel & M. Kessler	atpB	GU476809	FTOL v1.7
Polypodiaceae	Alansmia lanigera (Desv.) Moguel & M. Kessler	rbcL	GU476844	FTOL v1.7
Polypodiaceae	Alansmia lanigera (Desv.) Moguel & M. Kessler	trnL-trnF	GU476718	FTOL v1.7
Polypodiaceae	Alansmia senilis (Fée) Moguel & M. Kessler	atpB	GU376474	FTOL v1.7
Polypodiaceae	Alansmia senilis (Fée) Moguel & M. Kessler	rbcL	GU376498	FTOL v1.7
Polypodiaceae	Alansmia senilis (Fée) Moguel & M. Kessler	rps4-trnS	JN654941	FTOL v1.7
Polypodiaceae	Alansmia senilis (Fée) Moguel & M. Kessler	trnL-trnF	JN654967	FTOL v1.7
Polypodiaceae	Alansmia smithii (A. Rojas) Moguel & M. Kessler	rbcL	MK319096	FTOL v1.7
Polypodiaceae	Alansmia smithii (A. Rojas) Moguel & M. Kessler	trnL-trnF	MK318994	FTOL v1.7
Polypodiaceae	Alansmia stella (Copel.) Moguel & M. Kessler	atpB	GU376473	FTOL v1.7
Polypodiaceae	Alansmia stella (Copel.) Moguel & M. Kessler	rbcL	GU376499	FTOL v1.7
Polypodiaceae	Alansmia stella (Copel.) Moguel & M. Kessler	rps4-trnS	JN654939	FTOL v1.7
Polypodiaceae	Alansmia stella (Copel.) Moguel & M. Kessler	trnL-trnF	JN654965	FTOL v1.7
Polypodiaceae	Alansmia turrialbae (Christ) Moguel & M. Kessler	atpB	GU376472	FTOL v1.7
Polypodiaceae	Alansmia turrialbae (Christ) Moguel & M. Kessler	rbcL	GU376497	FTOL v1.7
Polypodiaceae	Alansmia turrialbae (Christ) Moguel & M. Kessler	rps4-trnS	JN654944	FTOL v1.7
Polypodiaceae	Alansmia turrialbae (Christ) Moguel & M. Kessler	trnL-trnF	JN654970	FTOL v1.7
Polypodiaceae	Archigrammitis friderici-et-pauli (Christ) Parris	atpB	KM218817	FTOL v1.7
Polypodiaceae	Archigrammitis friderici-et-pauli (Christ) Parris	rbcL	KM218751	FTOL v1.7
Polypodiaceae	Archigrammitis friderici-et-pauli (Christ) Parris	rps4-trnS	KY712388	FTOL v1.7
Polypodiaceae	Archigrammitis friderici-et-pauli (Christ) Parris	trnL-trnF	KM106046	FTOL v1.7
Polypodiaceae	Archigrammitis marquesensis (Parris) Parris	rbcL	MT215977	FTOL v1.7
Polypodiaceae	Archigrammitis ponapensis (Copel.) Parris	rbcL	MT657611	FTOL v1.7
Polypodiaceae	Archigrammitis tahitensis (C. Chr.) Parris	rbcL	KY099756	FTOL v1.7
Polypodiaceae	Ascogrammitis anfractuosa (Kunze ex Klotzsch) Sundu	atpB	MG948936	FTOL v1.7
Polypodiaceae	Ascogrammitis anfractuosa (Kunze ex Klotzsch) Sundu	rbcL	MG948938	FTOL v1.7
Polypodiaceae	Ascogrammitis anfractuosa (Kunze ex Klotzsch) Sundu	rps4-trnS	MG966515	FTOL v1.7
Polypodiaceae	Ascogrammitis anfractuosa (Kunze ex Klotzsch) Sundu	trnL-trnF	MG966517	FTOL v1.7
Polypodiaceae	Ascogrammitis angustipes (Copel.) Sundue	atpB	KM218837	FTOL v1.7
Polypodiaceae	Ascogrammitis angustipes (Copel.) Sundue	rbcL	GU476891	FTOL v1.7
Polypodiaceae	Ascogrammitis angustipes (Copel.) Sundue	rps4-trnS	KM106109	FTOL v1.7
Polypodiaceae	Ascogrammitis angustipes (Copel.) Sundue	trnL-trnF	GU476703	FTOL v1.7
Polypodiaceae	Ascogrammitis athyrioides (Hook.) Sundue	atpB	KM218840	FTOL v1.7
Polypodiaceae	Ascogrammitis athyrioides (Hook.) Sundue	rbcL	GU476856	FTOL v1.7
Polypodiaceae	Ascogrammitis athyrioides (Hook.) Sundue	rps4-trnS	KM106110	FTOL v1.7
Polypodiaceae	Ascogrammitis athyrioides (Hook.) Sundue	trnL-trnF	GU476704	FTOL v1.7
Polypodiaceae	Ascogrammitis clathrata (Sundue & M. Kessler) Sundu	atpB	KM218838	FTOL v1.7
Polypodiaceae	Ascogrammitis clathrata (Sundue & M. Kessler) Sundu	rbcL	GU476843	FTOL v1.7
Polypodiaceae	Ascogrammitis clathrata (Sundue & M. Kessler) Sundu	rps4-trnS	KM106111	FTOL v1.7
Polypodiaceae	Ascogrammitis clathrata (Sundue & M. Kessler) Sundu	trnL-trnF	GU476708	FTOL v1.7
Polypodiaceae	Ascogrammitis clavigera A. R. Sm. ex Sundue	atpB	KM218839	FTOL v1.7
Polypodiaceae	Ascogrammitis clavigera A. R. Sm. ex Sundue	rbcL	GU476925	FTOL v1.7
Polypodiaceae	Ascogrammitis clavigera A. R. Sm. ex Sundue	rps4-trnS	KM106112	FTOL v1.7
Polypodiaceae	Ascogrammitis clavigera A. R. Sm. ex Sundue	trnL-trnF	GU476709	FTOL v1.7
Polypodiaceae	Ascogrammitis colombiensis Sundue	atpB	GU476805	FTOL v1.7
Polypodiaceae	Ascogrammitis colombiensis Sundue	rbcL	GU476827	FTOL v1.7
Polypodiaceae	Ascogrammitis colombiensis Sundue	rps4-trnS	KM106113	FTOL v1.7
Polypodiaceae	Ascogrammitis colombiensis Sundue	trnL-trnF	GU476711	FTOL v1.7
Polypodiaceae	Ascogrammitis cuencana (Hieron.) Sundue	rbcL	GU476851	FTOL v1.7
Polypodiaceae	Ascogrammitis cuencana (Hieron.) Sundue	rps4-trnS	KM106114	FTOL v1.7
Polypodiaceae	Ascogrammitis cuencana (Hieron.) Sundue	trnL-trnF	GU476714	FTOL v1.7
Polypodiaceae	Ascogrammitis david-smithii (Stolze) Sundue	atpB	GU476794	FTOL v1.7
Polypodiaceae	Ascogrammitis david-smithii (Stolze) Sundue	rbcL	GU476911	FTOL v1.7
Polypodiaceae	Ascogrammitis david-smithii (Stolze) Sundue	rps4-trnS	GU387122	FTOL v1.7
Polypodiaceae	Ascogrammitis david-smithii (Stolze) Sundue	trnL-trnF	GU387284	FTOL v1.7
Polypodiaceae	Ascogrammitis dilatata (Sundue & M. Kessler) Sundue	atpB	GU376640	FTOL v1.7
Polypodiaceae	Ascogrammitis dilatata (Sundue & M. Kessler) Sundue	rbcL	GU387033	FTOL v1.7
Polypodiaceae	Ascogrammitis dilatata (Sundue & M. Kessler) Sundue	rps4-trnS	GU387124	FTOL v1.7
Polypodiaceae	Ascogrammitis dilatata (Sundue & M. Kessler) Sundue	trnL-trnF	GU387285	FTOL v1.7
Polypodiaceae	Ascogrammitis lehnertii Sundue	atpB	MG948937	FTOL v1.7
Polypodiaceae	Ascogrammitis lehnertii Sundue	rbcL	MG948939	FTOL v1.7
Polypodiaceae	Ascogrammitis lehnertii Sundue	rps4-trnS	MG966516	FTOL v1.7
Polypodiaceae	Ascogrammitis lehnertii Sundue	trnL-trnF	MG966518	FTOL v1.7
Polypodiaceae	Ascogrammitis loxensis Sundue	atpB	GU376641	FTOL v1.7
Polypodiaceae	Ascogrammitis loxensis Sundue	rbcL	GU386995	FTOL v1.7
Polypodiaceae	Ascogrammitis loxensis Sundue	rps4-trnS	GU387125	FTOL v1.7
Polypodiaceae	Ascogrammitis loxensis Sundue	trnL-trnF	GU387286	FTOL v1.7
Polypodiaceae	Ascogrammitis nana (Sundue & M. Kessler) Sundue	atpB	GU376642	FTOL v1.7
Polypodiaceae	Ascogrammitis nana (Sundue & M. Kessler) Sundue	rbcL	GU387031	FTOL v1.7
Polypodiaceae	Ascogrammitis nana (Sundue & M. Kessler) Sundue	rps4-trnS	GU387126	FTOL v1.7
Polypodiaceae	Ascogrammitis nana (Sundue & M. Kessler) Sundue	trnL-trnF	GU387287	FTOL v1.7
Polypodiaceae	Ascogrammitis pichinchae (Sodiro) Sundue	rbcL	GU476928	FTOL v1.7
Polypodiaceae	Ascogrammitis pichinchae (Sodiro) Sundue	trnL-trnF	GU476730	FTOL v1.7
Polypodiaceae	Ascogrammitis pichinchensis (Hieron.) Sundue	atpB	GU476816	FTOL v1.7
Polypodiaceae	Ascogrammitis pichinchensis (Hieron.) Sundue	rbcL	GU476854	FTOL v1.7
Polypodiaceae	Ascogrammitis pichinchensis (Hieron.) Sundue	rps4-trnS	KM106116	FTOL v1.7
Polypodiaceae	Ascogrammitis pichinchensis (Hieron.) Sundue	trnL-trnF	GU476732	FTOL v1.7
Polypodiaceae	Boonkerdia khaoluangensis (Tagawa & K.Iwats.) Li Bin	atpB	OQ702709	FTOL v1.7
Polypodiaceae	Boonkerdia khaoluangensis (Tagawa & K.Iwats.) Li Bin	rps4	OQ717278	FTOL v1.7
Polypodiaceae	Boonkerdia khaoluangensis (Tagawa & K.Iwats.) Li Bin	rps4-trnS	OQ717282	FTOL v1.7
Polypodiaceae	Boonkerdia khaoluangensis (Tagawa & K.Iwats.) Li Bin	trnL-trnF	OQ717097	FTOL v1.7
Polypodiaceae	Bosmania lastii (Baker) Testo	rbcL	EU482961	FTOL v1.7
Polypodiaceae	Bosmania lastii (Baker) Testo	trnL-trnF	EU483058	FTOL v1.7
Polypodiaceae	Bosmania membranacea (D. Don) Testo	plastome	MT130574	FTOL v1.7
Polypodiaceae	Calligrammitis beddomeana (Alderw.) Parris, Sundue,	atpB	OQ702725	FTOL v1.7
Polypodiaceae	Calligrammitis beddomeana (Alderw.) Parris, Sundue,	rbcL	OK514679	FTOL v1.7
Polypodiaceae	Calligrammitis beddomeana (Alderw.) Parris, Sundue,	rps4	OQ717273	FTOL v1.7
Polypodiaceae	Calligrammitis beddomeana (Alderw.) Parris, Sundue,	rps4-trnS	OQ717273	FTOL v1.7
Polypodiaceae	Calymmodon asiaticus Copel.	rbcL	MF450450	FTOL v1.7
Polypodiaceae	Calymmodon clavifer (Hook.) T. Moore	atpB	KM218818	FTOL v1.7
Polypodiaceae	Calymmodon clavifer (Hook.) T. Moore	rbcL	KM218804	FTOL v1.7
Polypodiaceae	Calymmodon clavifer (Hook.) T. Moore	rps4-trnS	KM106117	FTOL v1.7
Polypodiaceae	Calymmodon clavifer (Hook.) T. Moore	trnL-trnF	KM106049	FTOL v1.7
Polypodiaceae	Calymmodon cucullatus (Nees & Blume) C. Presl	plastome	OQ968267	FTOL v1.7
Polypodiaceae	Calymmodon curtus Parris	rps4	AY362688	FTOL v1.7
Polypodiaceae	Calymmodon gracilis (Fée) Copel.	atpA	EF463801	FTOL v1.7
Polypodiaceae	Calymmodon gracilis (Fée) Copel.	atpB	AY459451	FTOL v1.7
Polypodiaceae	Calymmodon gracilis (Fée) Copel.	rbcL	AY362341	FTOL v1.7
Polypodiaceae	Calymmodon gracilis (Fée) Copel.	rps4	OQ717230	FTOL v1.7
Polypodiaceae	Calymmodon gracilis (Fée) Copel.	rps4-trnS	OQ717233	FTOL v1.7
Polypodiaceae	Calymmodon gracilis (Fée) Copel.	trnL-trnF	OQ717104	FTOL v1.7
Polypodiaceae	Calymmodon latealatus Copel.	rbcL	MT657579	FTOL v1.7
Polypodiaceae	Calymmodon luerssenianus (Domin) Copel.	atpB	AY459452	FTOL v1.7
Polypodiaceae	Calymmodon luerssenianus (Domin) Copel.	rbcL	AY460618	FTOL v1.7
Polypodiaceae	Calymmodon mnioides Copel.	atpB	KM218820	FTOL v1.7
Polypodiaceae	Calymmodon mnioides Copel.	rbcL	KM218807	FTOL v1.7
Polypodiaceae	Calymmodon mnioides Copel.	rps4-trnS	KM106120	FTOL v1.7

Polypodiaceae	Calymmodon mnioides Copel.	trnL-trnF	KM106052	FTOL v1.7
Polypodiaceae	Calymmodon orientalis Copel.	rbcL	KY099779	FTOL v1.7
Polypodiaceae	Calymmodon ponapensis Copel.	rbcL	MT657577	FTOL v1.7
Polypodiaceae	Campyloneurum abruptum (Lindm.) B. León	rbcL	MF318031	FTOL v1.7
Polypodiaceae	Campyloneurum abruptum (Lindm.) B. León	rps4	MT806105	FTOL v1.7
Polypodiaceae	Campyloneurum abruptum (Lindm.) B. León	rps4-trnS	MT806105	FTOL v1.7
Polypodiaceae	Campyloneurum abruptum (Lindm.) B. León	trnL-trnF	MT568797	FTOL v1.7
Polypodiaceae	Campyloneurum aglaolepis (Alston) de la Sota	plastome	MW876310	FTOL v1.7
Polypodiaceae	Campyloneurum amphostenon (Kunze ex Klotzsch) Fée	rbcL	MF318050	FTOL v1.7
Polypodiaceae	Campyloneurum amphostenon (Kunze ex Klotzsch) Fée	rps4-trnS	MF318070	FTOL v1.7
Polypodiaceae	Campyloneurum amphostenon (Kunze ex Klotzsch) Fée	trnL-trnF	MF318324	FTOL v1.7
Polypodiaceae	Campyloneurum anetioides (Christ) R. M. Tryon & A. F.	rbcL	MF317991	FTOL v1.7
Polypodiaceae	Campyloneurum anetioides (Christ) R. M. Tryon & A. F.	rps4-trnS	MF318071	FTOL v1.7
Polypodiaceae	Campyloneurum anetioides (Christ) R. M. Tryon & A. F.	trnL-trnF	MF318325	FTOL v1.7
Polypodiaceae	Campyloneurum angustifolium (Sw.) Fée	rbcL	MF317986	FTOL v1.7
Polypodiaceae	Campyloneurum angustifolium (Sw.) Fée	rps4-trnS	MF318076	FTOL v1.7
Polypodiaceae	Campyloneurum angustifolium (Sw.) Fée	trnL-trnF	MF318327	FTOL v1.7
Polypodiaceae	Campyloneurum angustipaleatum (Alston) M. Mey. ex	rbcL	MF318017	FTOL v1.7
Polypodiaceae	Campyloneurum angustipaleatum (Alston) M. Mey. ex	rps4-trnS	MF318078	FTOL v1.7
Polypodiaceae	Campyloneurum angustipaleatum (Alston) M. Mey. ex	trnL-trnF	MF318329	FTOL v1.7
Polypodiaceae	Campyloneurum aphanophlebium (Kunze) T. Moore	rbcL	EU250346	FTOL v1.7
Polypodiaceae	Campyloneurum aphanophlebium (Kunze) T. Moore	rps4	EU250352	FTOL v1.7
Polypodiaceae	Campyloneurum aphanophlebium (Kunze) T. Moore	rps4-trnS	EU250352	FTOL v1.7
Polypodiaceae	Campyloneurum aphanophlebium (Kunze) T. Moore	trnL-trnF	EF104511	FTOL v1.7
Polypodiaceae	Campyloneurum asplundii (C. Chr.) Ching	rbcL	EU250347	FTOL v1.7
Polypodiaceae	Campyloneurum asplundii (C. Chr.) Ching	rps4	EU250353	FTOL v1.7
Polypodiaceae	Campyloneurum asplundii (C. Chr.) Ching	rps4-trnS	EU250353	FTOL v1.7
Polypodiaceae	Campyloneurum asplundii (C. Chr.) Ching	trnL-trnF	EF104512	FTOL v1.7
Polypodiaceae	Campyloneurum atlanticum R. C. Moran & Labiak	rbcL	MF317962	FTOL v1.7
Polypodiaceae	Campyloneurum atlanticum R. C. Moran & Labiak	rps4-trnS	MF318085	FTOL v1.7
Polypodiaceae	Campyloneurum atlanticum R. C. Moran & Labiak	trnL-trnF	MF318336	FTOL v1.7
Polypodiaceae	Campyloneurum austrobrasilianum (Alston) de la Sota	rbcL	MF318036	FTOL v1.7
Polypodiaceae	Campyloneurum austrobrasilianum (Alston) de la Sota	rps4	MT806107	FTOL v1.7
Polypodiaceae	Campyloneurum austrobrasilianum (Alston) de la Sota	rps4-trnS	MT806107	FTOL v1.7
Polypodiaceae	Campyloneurum austrobrasilianum (Alston) de la Sota	trnL-trnF	MT568799	FTOL v1.7
Polypodiaceae	Campyloneurum brevifolium (Lodd. ex Link) Link	atpA	EF463802	FTOL v1.7
Polypodiaceae	Campyloneurum brevifolium (Lodd. ex Link) Link	atpB	EF463491	FTOL v1.7
Polypodiaceae	Campyloneurum brevifolium (Lodd. ex Link) Link	rbcL	EF463245	FTOL v1.7
Polypodiaceae	Campyloneurum centrobrasilianum Lellinger	rbcL	MF317968	FTOL v1.7
Polypodiaceae	Campyloneurum centrobrasilianum Lellinger	rps4-trnS	MF318094	FTOL v1.7
Polypodiaceae	Campyloneurum centrobrasilianum Lellinger	trnL-trnF	MF318346	FTOL v1.7
Polypodiaceae	Campyloneurum chlorolepis Alston	rbcL	MF318062	FTOL v1.7
Polypodiaceae	Campyloneurum chlorolepis Alston	rps4-trnS	MF318098	FTOL v1.7
Polypodiaceae	Campyloneurum chlorolepis Alston	trnL-trnF	MF318349	FTOL v1.7
Polypodiaceae	Campyloneurum chrysopodum (Klotzsch) Fée	rbcL	MF318016	FTOL v1.7
Polypodiaceae	Campyloneurum chrysopodum (Klotzsch) Fée	rps4-trnS	MF318099	FTOL v1.7
Polypodiaceae	Campyloneurum chrysopodum (Klotzsch) Fée	trnL-trnF	MF318350	FTOL v1.7
Polypodiaceae	Campyloneurum coarctatum (Kunze) Fée	rbcL	MF318008	FTOL v1.7
Polypodiaceae	Campyloneurum coarctatum (Kunze) Fée	rps4-trnS	MF318102	FTOL v1.7
Polypodiaceae	Campyloneurum coarctatum (Kunze) Fée	trnL-trnF	MF318351	FTOL v1.7
Polypodiaceae	Campyloneurum cochense (Hieron.) Ching	rbcL	MF318041	FTOL v1.7
Polypodiaceae	Campyloneurum cochense (Hieron.) Ching	rps4-trnS	MF318106	FTOL v1.7
Polypodiaceae	Campyloneurum cochense (Hieron.) Ching	trnL-trnF	MF318356	FTOL v1.7
Polypodiaceae	Campyloneurum costatum (Kunze) C. Presl	rbcL	KJ628651	FTOL v1.7
Polypodiaceae	Campyloneurum costatum (Kunze) C. Presl	rps4	MT806108	FTOL v1.7
Polypodiaceae	Campyloneurum costatum (Kunze) C. Presl	rps4-trnS	MT806108	FTOL v1.7
Polypodiaceae	Campyloneurum costatum (Kunze) C. Presl	trnL-trnF	MT568800	FTOL v1.7
Polypodiaceae	Campyloneurum decurrens (Raddi) C. Presl	rbcL	MF318033	FTOL v1.7
Polypodiaceae	Campyloneurum decurrens (Raddi) C. Presl	rps4	MT806109	FTOL v1.7
Polypodiaceae	Campyloneurum decurrens (Raddi) C. Presl	rps4-trnS	MT806109	FTOL v1.7
Polypodiaceae	Campyloneurum decurrens (Raddi) C. Presl	trnL-trnF	MT568801	FTOL v1.7
Polypodiaceae	Campyloneurum densifolium (Hieron.) Lellinger	rbcL	MF318064	FTOL v1.7
Polypodiaceae	Campyloneurum densifolium (Hieron.) Lellinger	rps4-trnS	MF318116	FTOL v1.7
Polypodiaceae	Campyloneurum densifolium (Hieron.) Lellinger	trnL-trnF	MF318363	FTOL v1.7
Polypodiaceae	Campyloneurum ensifolium (Willd.) J. Sm.	rbcL	MF318010	FTOL v1.7
Polypodiaceae	Campyloneurum ensifolium (Willd.) J. Sm.	rps4-trnS	MF318194	FTOL v1.7
Polypodiaceae	Campyloneurum ensifolium (Willd.) J. Sm.	trnL-trnF	MF318440	FTOL v1.7
Polypodiaceae	Campyloneurum falcoideum (Kuhn ex Hieron.) M. Mey.	rbcL	MF318049	FTOL v1.7
Polypodiaceae	Campyloneurum falcoideum (Kuhn ex Hieron.) M. Mey.	rps4-trnS	MF318118	FTOL v1.7
Polypodiaceae	Campyloneurum falcoideum (Kuhn ex Hieron.) M. Mey.	trnL-trnF	MF318365	FTOL v1.7
Polypodiaceae	Campyloneurum fallax Fée	rbcL	MF318024	FTOL v1.7
Polypodiaceae	Campyloneurum fallax Fée	rps4-trnS	MF318119	FTOL v1.7
Polypodiaceae	Campyloneurum fallax Fée	trnL-trnF	MF318366	FTOL v1.7
Polypodiaceae	Campyloneurum filiforme Labiak & R. C. Moran	rbcL	MF318000	FTOL v1.7
Polypodiaceae	Campyloneurum filiforme Labiak & R. C. Moran	rps4-trnS	MF318121	FTOL v1.7
Polypodiaceae	Campyloneurum filiforme Labiak & R. C. Moran	trnL-trnF	MF318368	FTOL v1.7
Polypodiaceae	Campyloneurum fuscosquamatum Lellinger	rbcL	MF317951	FTOL v1.7
Polypodiaceae	Campyloneurum fuscosquamatum Lellinger	rps4-trnS	MF318123	FTOL v1.7
Polypodiaceae	Campyloneurum fuscosquamatum Lellinger	trnL-trnF	MF318370	FTOL v1.7
Polypodiaceae	Campyloneurum gracile A. Rojas	rbcL	KY847860	FTOL v1.7
Polypodiaceae	Campyloneurum gracile A. Rojas	rps4	KY847864	FTOL v1.7
Polypodiaceae	Campyloneurum gracile A. Rojas	rps4-trnS	KY847864	FTOL v1.7
Polypodiaceae	Campyloneurum jamaicense Labiak & R. C. Moran	rbcL	MF317984	FTOL v1.7
Polypodiaceae	Campyloneurum jamaicense Labiak & R. C. Moran	rps4-trnS	MF318157	FTOL v1.7
Polypodiaceae	Campyloneurum jamaicense Labiak & R. C. Moran	trnL-trnF	MF318401	FTOL v1.7
Polypodiaceae	Campyloneurum lorentzii (Hieron.) Ching	rbcL	MF317971	FTOL v1.7
Polypodiaceae	Campyloneurum lorentzii (Hieron.) Ching	rps4-trnS	MF318127	FTOL v1.7
Polypodiaceae	Campyloneurum lorentzii (Hieron.) Ching	trnL-trnF	MF318374	FTOL v1.7
Polypodiaceae	Campyloneurum macrosorum Fée	rbcL	MF317976	FTOL v1.7
Polypodiaceae	Campyloneurum macrosorum Fée	rps4-trnS	MF318132	FTOL v1.7
Polypodiaceae	Campyloneurum macrosorum Fée	trnL-trnF	MF318377	FTOL v1.7
Polypodiaceae	Campyloneurum magnificum T. Moore	rbcL	MF318039	FTOL v1.7
Polypodiaceae	Campyloneurum magnificum T. Moore	rps4-trnS	MF318134	FTOL v1.7
Polypodiaceae	Campyloneurum magnificum T. Moore	trnL-trnF	MF318379	FTOL v1.7
Polypodiaceae	Campyloneurum major (Hieron. ex Hicken) Lellinger	rbcL	MF318011	FTOL v1.7
Polypodiaceae	Campyloneurum major (Hieron. ex Hicken) Lellinger	rps4-trnS	MF318126	FTOL v1.7
Polypodiaceae	Campyloneurum major (Hieron. ex Hicken) Lellinger	trnL-trnF	MF318373	FTOL v1.7
Polypodiaceae	Campyloneurum nitidissimum (Mett.) Ching	rbcL	MF318007	FTOL v1.7
Polypodiaceae	Campyloneurum nitidissimum (Mett.) Ching	rps4-trnS	MF318176	FTOL v1.7
Polypodiaceae	Campyloneurum nitidissimum (Mett.) Ching	trnL-trnF	MF318419	FTOL v1.7
Polypodiaceae	Campyloneurum nitidum (Kaulf.) C. Presl	rbcL	MF318025	FTOL v1.7
Polypodiaceae	Campyloneurum nitidum (Kaulf.) C. Presl	rps4-trnS	MF318137	FTOL v1.7
Polypodiaceae	Campyloneurum nitidum (Kaulf.) C. Presl	trnL-trnF	MF318382	FTOL v1.7
Polypodiaceae	Campyloneurum ophiocaulon (Klotzsch) Fée	rbcL	MF317964	FTOL v1.7
Polypodiaceae	Campyloneurum ophiocaulon (Klotzsch) Fée	rps4-trnS	MF318143	FTOL v1.7
Polypodiaceae	Campyloneurum ophiocaulon (Klotzsch) Fée	trnL-trnF	MF318389	FTOL v1.7
Polypodiaceae	Campyloneurum parvisquama Labiak & R. C. Moran	rps4-trnS	MF318144	FTOL v1.7
Polypodiaceae	Campyloneurum pascoense R. M. Tryon & A. F. Tryon	rbcL	MF318037	FTOL v1.7
Polypodiaceae	Campyloneurum pascoense R. M. Tryon & A. F. Tryon	rps4-trnS	MF318146	FTOL v1.7
Polypodiaceae	Campyloneurum pascoense R. M. Tryon & A. F. Tryon	trnL-trnF	MF318391	FTOL v1.7
Polypodiaceae	Campyloneurum pentaphyllum (Willd.) Pic. Serm.	rps4-trnS	MF318149	FTOL v1.7
Polypodiaceae	Campyloneurum phyllitidis (L.) C. Presl	rbcL	MF317975	FTOL v1.7
Polypodiaceae	Campyloneurum phyllitidis (L.) C. Presl	rps4-trnS	MF318151	FTOL v1.7
Polypodiaceae	Campyloneurum phyllitidis (L.) C. Presl	trnL-trnF	MF318395	FTOL v1.7
Polypodiaceae	Campyloneurum repens (Aubl.) C. Presl	rbcL	MF317965	FTOL v1.7
Polypodiaceae	Campyloneurum repens (Aubl.) C. Presl	rps4-trnS	MF318154	FTOL v1.7
Polypodiaceae	Campyloneurum repens (Aubl.) C. Presl	trnL-trnF	MF318398	FTOL v1.7
Polypodiaceae	Campyloneurum rigidum J. Sm.	rbcL	MF318032	FTOL v1.7
Polypodiaceae	Campyloneurum rigidum J. Sm.	rps4-trnS	MF318160	FTOL v1.7
Polypodiaceae	Campyloneurum rigidum J. Sm.	trnL-trnF	MF318404	FTOL v1.7
Polypodiaceae	Campyloneurum serpentinum (Christ) Ching	rbcL	MF318046	FTOL v1.7
Polypodiaceae	Campyloneurum serpentinum (Christ) Ching	rps4-trnS	MF318158	FTOL v1.7
Polypodiaceae	Campyloneurum serpentinum (Christ) Ching	trnL-trnF	MF318402	FTOL v1.7
Polypodiaceae	Campyloneurum solutum (Klotzsch) Fée	rbcL	MF318038	FTOL v1.7
Polypodiaceae	Campyloneurum solutum (Klotzsch) Fée	rps4-trnS	MF318164	FTOL v1.7
Polypodiaceae	Campyloneurum solutum (Klotzsch) Fée	trnL-trnF	MF318408	FTOL v1.7
Polypodiaceae	Campyloneurum sphenodes (Kunze ex Klotzsch) Fée	rbcL	MW138261	FTOL v1.7
Polypodiaceae	Campyloneurum tenuipes Maxon	rbcL	MF317987	FTOL v1.7
Polypodiaceae	Campyloneurum tenuipes Maxon	rps4-trnS	MF318173	FTOL v1.7
Polypodiaceae	Campyloneurum tenuipes Maxon	trnL-trnF	MF318417	FTOL v1.7
Polypodiaceae	Campyloneurum tucumanense (Hieron.) Ching	rbcL	MF317983	FTOL v1.7
Polypodiaceae	Campyloneurum tucumanense (Hieron.) Ching	rps4-trnS	MF318145	FTOL v1.7
Polypodiaceae	Campyloneurum tucumanense (Hieron.) Ching	trnL-trnF	MF318390	FTOL v1.7
Polypodiaceae	Campyloneurum vexatum (D. C. Eaton) Ching	rbcL	MF317996	FTOL v1.7
Polypodiaceae	Campyloneurum vexatum (D. C. Eaton) Ching	rps4-trnS	MF318177	FTOL v1.7
Polypodiaceae	Campyloneurum vexatum (D. C. Eaton) Ching	trnL-trnF	MF318421	FTOL v1.7
Polypodiaceae	Campyloneurum vulpinum (Lindm.) Ching	rbcL	MF317995	FTOL v1.7
Polypodiaceae	Campyloneurum vulpinum (Lindm.) Ching	rps4-trnS	MF318178	FTOL v1.7
Polypodiaceae	Campyloneurum vulpinum (Lindm.) Ching	trnL-trnF	MF318422	FTOL v1.7
Polypodiaceae	Campyloneurum wurdackii B. León	rbcL	MF318058	FTOL v1.7
Polypodiaceae	Campyloneurum wurdackii B. León	rps4-trnS	MF318181	FTOL v1.7
Polypodiaceae	Campyloneurum wurdackii B. León	trnL-trnF	MF318426	FTOL v1.7
Polypodiaceae	Campyloneurum xalapense Fée	rbcL	MF318009	FTOL v1.7
Polypodiaceae	Campyloneurum xalapense Fée	rps4-trnS	MF318193	FTOL v1.7
Polypodiaceae	Campyloneurum xalapense Fée	trnL-trnF	MF318439	FTOL v1.7
Polypodiaceae	Ceradenia argyrata (Bory ex Willd.) Parris	atpB	KY711752	FTOL v1.7
Polypodiaceae	Ceradenia argyrata (Bory ex Willd.) Parris	rbcL	KY711926	FTOL v1.7
Polypodiaceae	Ceradenia argyrata (Bory ex Willd.) Parris	rps4-trnS	KY712253	FTOL v1.7
Polypodiaceae	Ceradenia argyrata (Bory ex Willd.) Parris	trnL-trnF	KY711579	FTOL v1.7
Polypodiaceae	Ceradenia aulaeifolia L. E. Bishop ex A. R. Sm.	atpB	AY459453	FTOL v1.7
Polypodiaceae	Ceradenia aulaeifolia L. E. Bishop ex A. R. Sm.	rbcL	AY460619	FTOL v1.7
Polypodiaceae	Ceradenia ayopayana M. Kessler & A. R. Sm.	rbcL	KM218811	FTOL v1.7
Polypodiaceae	Ceradenia ayopayana M. Kessler & A. R. Sm.	rps4-trnS	KM106123	FTOL v1.7
Polypodiaceae	Ceradenia ayopayana M. Kessler & A. R. Sm.	trnL-trnF	KM106053	FTOL v1.7
Polypodiaceae	Ceradenia comorensis (Baker) Parris	atpB	KY711760	FTOL v1.7
Polypodiaceae	Ceradenia comorensis (Baker) Parris	rbcL	KY711935	FTOL v1.7
Polypodiaceae	Ceradenia comorensis (Baker) Parris	rps4-trnS	KY712262	FTOL v1.7
Polypodiaceae	Ceradenia comorensis (Baker) Parris	trnL-trnF	KY711588	FTOL v1.7
Polypodiaceae	Ceradenia curvata (Sw.) L. E. Bishop	atpB	KM218821	FTOL v1.7
Polypodiaceae	Ceradenia curvata (Sw.) L. E. Bishop	rbcL	KM218788	FTOL v1.7
Polypodiaceae	Ceradenia curvata (Sw.) L. E. Bishop	trnL-trnF	KM106054	FTOL v1.7
Polypodiaceae	Ceradenia deltodon (Baker) Parris	atpB	KY711757	FTOL v1.7
Polypodiaceae	Ceradenia deltodon (Baker) Parris	rbcL	KY711931	FTOL v1.7
Polypodiaceae	Ceradenia deltodon (Baker) Parris	rps4-trnS	KY712257	FTOL v1.7
Polypodiaceae	Ceradenia deltodon (Baker) Parris	trnL-trnF	KY711580	FTOL v1.7
Polypodiaceae	Ceradenia farinosa (Hook.) L. E. Bishop	atpB	KM218823	FTOL v1.7
Polypodiaceae	Ceradenia farinosa (Hook.) L. E. Bishop	rbcL	KM218790	FTOL v1.7
Polypodiaceae	Ceradenia farinosa (Hook.) L. E. Bishop	rps4-trnS	KM106124	FTOL v1.7
Polypodiaceae	Ceradenia farinosa (Hook.) L. E. Bishop	trnL-trnF	KM106055	FTOL v1.7
Polypodiaceae	Ceradenia fucoides (Christ) L. E. Bishop	atpB	GU476749	FTOL v1.7
Polypodiaceae	Ceradenia fucoides (Christ) L. E. Bishop	rbcL	GU476907	FTOL v1.7
Polypodiaceae	Ceradenia fucoides (Christ) L. E. Bishop	rps4-trnS	KM106125	FTOL v1.7
Polypodiaceae	Ceradenia fucoides (Christ) L. E. Bishop	trnL-trnF	GU476625	FTOL v1.7
Polypodiaceae	Ceradenia intonsa L. E. Bishop ex León-Parra & Mostac	atpB	GU476750	FTOL v1.7
Polypodiaceae	Ceradenia intonsa L. E. Bishop ex León-Parra & Mostac	rbcL	GU476901	FTOL v1.7
Polypodiaceae	Ceradenia intonsa L. E. Bishop ex León-Parra & Mostac	trnL-trnF	GU476626	FTOL v1.7
Polypodiaceae	Ceradenia intricata (C. V. Morton) L. E. Bishop ex A. R.	atpB	KM218833	FTOL v1.7
Polypodiaceae	Ceradenia intricata (C. V. Morton) L. E. Bishop ex A. R.	rbcL	KM218791	FTOL v1.7
Polypodiaceae	Ceradenia intricata (C. V. Morton) L. E. Bishop ex A. R.	rps4-trnS	KM106127	FTOL v1.7
Polypodiaceae	Ceradenia intricata (C. V. Morton) L. E. Bishop ex A. R.	trnL-trnF	KM106056	FTOL v1.7
Polypodiaceae	Ceradenia jungermannioides (Klotzsch) L. E. Bishop	atpB	AY459454	FTOL v1.7
Polypodiaceae	Ceradenia jungermannioides (Klotzsch) L. E. Bishop	rbcL	AY460620	FTOL v1.7
Polypodiaceae	Ceradenia kalbreyeri (Baker) L. E. Bishop	atpB	GU476745	FTOL v1.7
Polypodiaceae	Ceradenia kalbreyeri (Baker) L. E. Bishop	rbcL	GU476887	FTOL v1.7
Polypodiaceae	Ceradenia kalbreyeri (Baker) L. E. Bishop	trnL-trnF	GU476619	FTOL v1.7
Polypodiaceae	Ceradenia knightii (Copel.) L. E. Bishop	rbcL	MK319106	FTOL v1.7
Polypodiaceae	Ceradenia kookenamae (Jenman) L. E. Bishop	rbcL	MK319100	FTOL v1.7

Polypodiaceae	Ceradenia kookenamae (Jenman) L. E. Bishop	trnL-trnF	MK318998	FTOL v1.7
Polypodiaceae	Ceradenia leucosora (Bojer ex Hook.) Parris	atpB	KY711758	FTOL v1.7
Polypodiaceae	Ceradenia leucosora (Bojer ex Hook.) Parris	rbcL	KF992464	FTOL v1.7
Polypodiaceae	Ceradenia leucosora (Bojer ex Hook.) Parris	rps4-trnS	KY712261	FTOL v1.7
Polypodiaceae	Ceradenia leucosora (Bojer ex Hook.) Parris	trnL-trnF	KY711587	FTOL v1.7
Polypodiaceae	Ceradenia madidiensis M. Kessler & A. R. Sm.	atpB	KM218834	FTOL v1.7
Polypodiaceae	Ceradenia madidiensis M. Kessler & A. R. Sm.	rps4-trnS	KM106129	FTOL v1.7
Polypodiaceae	Ceradenia madidiensis M. Kessler & A. R. Sm.	trnL-trnF	KM106057	FTOL v1.7
Polypodiaceae	Ceradenia meridensis (Klotzsch) L. E. Bishop	rbcL	MK319130	FTOL v1.7
Polypodiaceae	Ceradenia meridensis (Klotzsch) L. E. Bishop	trnL-trnF	MK318999	FTOL v1.7
Polypodiaceae	Ceradenia pearcei (Baker) L. E. Bishop	atpB	KM218822	FTOL v1.7
Polypodiaceae	Ceradenia pearcei (Baker) L. E. Bishop	rbcL	KM218789	FTOL v1.7
Polypodiaceae	Ceradenia pearcei (Baker) L. E. Bishop	rps4-trnS	KM106130	FTOL v1.7
Polypodiaceae	Ceradenia pearcei (Baker) L. E. Bishop	trnL-trnF	KM106058	FTOL v1.7
Polypodiaceae	Ceradenia pilipes (Hook.) L. E. Bishop	atpA	EF463804	FTOL v1.7
Polypodiaceae	Ceradenia pilipes (Hook.) L. E. Bishop	atpB	AY459456	FTOL v1.7
Polypodiaceae	Ceradenia pilipes (Hook.) L. E. Bishop	rbcL	AY460622	FTOL v1.7
Polypodiaceae	Ceradenia pilipes (Hook.) L. E. Bishop	trnL-trnF	GU476621	FTOL v1.7
Polypodiaceae	Ceradenia sechellarum (Baker) Parris	atpB	KY711886	FTOL v1.7
Polypodiaceae	Ceradenia sechellarum (Baker) Parris	rbcL	KY712060	FTOL v1.7
Polypodiaceae	Ceradenia sechellarum (Baker) Parris	rps4-trnS	KY712378	FTOL v1.7
Polypodiaceae	Ceradenia sechellarum (Baker) Parris	trnL-trnF	KY711706	FTOL v1.7
Polypodiaceae	Ceradenia spixiana (M. Martens ex Mett.) L. E. Bishop	atpB	AY459457	FTOL v1.7
Polypodiaceae	Ceradenia spixiana (M. Martens ex Mett.) L. E. Bishop	rbcL	AY460623	FTOL v1.7
Polypodiaceae	Chrysogrammitis glandulosa (J. Sm.) Parris	atpB	JF514082	FTOL v1.7
Polypodiaceae	Chrysogrammitis glandulosa (J. Sm.) Parris	rbcL	JF514014	FTOL v1.7
Polypodiaceae	Chrysogrammitis glandulosa (J. Sm.) Parris	trnL-trnF	JF514048	FTOL v1.7
Polypodiaceae	Chrysogrammitis musgraviana (Baker) Parris	atpB	KM218825	FTOL v1.7
Polypodiaceae	Chrysogrammitis musgraviana (Baker) Parris	rbcL	KM218797	FTOL v1.7
Polypodiaceae	Chrysogrammitis musgraviana (Baker) Parris	rps4-trnS	KM106132	FTOL v1.7
Polypodiaceae	Chrysogrammitis musgraviana (Baker) Parris	trnL-trnF	KM106059	FTOL v1.7
Polypodiaceae	Cochlidium jungens L. E. Bishop	rbcL	MK319131	FTOL v1.7
Polypodiaceae	Cochlidium jungens L. E. Bishop	trnL-trnF	MK319000	FTOL v1.7
Polypodiaceae	Cochlidium linearifolium (Desv.) Maxon	rbcL	MK319103	FTOL v1.7
Polypodiaceae	Cochlidium linearifolium (Desv.) Maxon	trnL-trnF	MK319002	FTOL v1.7
Polypodiaceae	Cochlidium punctatum (Raddi) L. E. Bishop	atpB	JF514057	FTOL v1.7
Polypodiaceae	Cochlidium punctatum (Raddi) L. E. Bishop	rbcL	AY460625	FTOL v1.7
Polypodiaceae	Cochlidium punctatum (Raddi) L. E. Bishop	trnL-trnF	GU476631	FTOL v1.7
Polypodiaceae	Cochlidium rostratum (Hook.) Maxon ex C. Chr.	atpB	AY459459	FTOL v1.7
Polypodiaceae	Cochlidium rostratum (Hook.) Maxon ex C. Chr.	rbcL	AY460626	FTOL v1.7
Polypodiaceae	Cochlidium seminudum (Willd.) Maxon	atpB	AY459460	FTOL v1.7
Polypodiaceae	Cochlidium seminudum (Willd.) Maxon	rbcL	AY460627	FTOL v1.7
Polypodiaceae	Cochlidium serrulatum (Sw.) L. E. Bishop	atpB	KY711765	FTOL v1.7
Polypodiaceae	Cochlidium serrulatum (Sw.) L. E. Bishop	rbcL	KY711940	FTOL v1.7
Polypodiaceae	Cochlidium serrulatum (Sw.) L. E. Bishop	rps4-trnS	KY712267	FTOL v1.7
Polypodiaceae	Cochlidium serrulatum (Sw.) L. E. Bishop	trnL-trnF	KY711592	FTOL v1.7
Polypodiaceae	Ctenopterella blechnoides (Grev.) Parris	rbcL	MT657582	FTOL v1.7
Polypodiaceae	Ctenopterella denticulata (Blume) Parris	atpB	JF514081	FTOL v1.7
Polypodiaceae	Ctenopterella denticulata (Blume) Parris	rbcL	JF514013	FTOL v1.7
Polypodiaceae	Ctenopterella denticulata (Blume) Parris	trnL-trnF	JF514047	FTOL v1.7
Polypodiaceae	Ctenopterella lasiostipes (Mett.) Parris	atpA	EF463807	FTOL v1.7
Polypodiaceae	Ctenopterella lasiostipes (Mett.) Parris	atpB	KY711899	FTOL v1.7
Polypodiaceae	Ctenopterella lasiostipes (Mett.) Parris	rbcL	AY460630	FTOL v1.7
Polypodiaceae	Ctenopterella lasiostipes (Mett.) Parris	rps4	OQ717222	FTOL v1.7
Polypodiaceae	Ctenopterella lasiostipes (Mett.) Parris	rps4-trnS	KY712393	FTOL v1.7
Polypodiaceae	Ctenopterella lasiostipes (Mett.) Parris	trnL-trnF	OQ717113	FTOL v1.7
Polypodiaceae	Ctenopterella nhatrangensis (C. Chr. & Tardieu) Parris	atpB	OQ702683	FTOL v1.7
Polypodiaceae	Ctenopterella nhatrangensis (C. Chr. & Tardieu) Parris	rps4	OQ717216	FTOL v1.7
Polypodiaceae	Ctenopterella nhatrangensis (C. Chr. & Tardieu) Parris	rps4-trnS	OQ717232	FTOL v1.7
Polypodiaceae	Ctenopterella nhatrangensis (C. Chr. & Tardieu) Parris	trnL-trnF	OQ717115	FTOL v1.7
Polypodiaceae	Ctenopterella parvula (Bory ex Willd.) Parris	atpB	KY711770	FTOL v1.7
Polypodiaceae	Ctenopterella parvula (Bory ex Willd.) Parris	rbcL	KY711945	FTOL v1.7
Polypodiaceae	Ctenopterella parvula (Bory ex Willd.) Parris	rps4-trnS	KY712272	FTOL v1.7
Polypodiaceae	Ctenopterella parvula (Bory ex Willd.) Parris	trnL-trnF	KY711597	FTOL v1.7
Polypodiaceae	Ctenopterella zenkeri (Hieron.) Parris	atpB	KY711774	FTOL v1.7
Polypodiaceae	Ctenopterella zenkeri (Hieron.) Parris	rbcL	KY711949	FTOL v1.7
Polypodiaceae	Ctenopterella zenkeri (Hieron.) Parris	rps4-trnS	KY712276	FTOL v1.7
Polypodiaceae	Ctenopterella zenkeri (Hieron.) Parris	trnL-trnF	KY711601	FTOL v1.7
Polypodiaceae	Dasygrammitis brevivenosa (Alderw.) Parris	atpB	KM218829	FTOL v1.7
Polypodiaceae	Dasygrammitis brevivenosa (Alderw.) Parris	rbcL	KM218775	FTOL v1.7
Polypodiaceae	Dasygrammitis brevivenosa (Alderw.) Parris	rps4-trnS	KM106136	FTOL v1.7
Polypodiaceae	Dasygrammitis brevivenosa (Alderw.) Parris	trnL-trnF	KM106061	FTOL v1.7
Polypodiaceae	Dasygrammitis crassifrons (Baker) Parris	atpB	JF514062	FTOL v1.7
Polypodiaceae	Dasygrammitis crassifrons (Baker) Parris	rbcL	JF513992	FTOL v1.7
Polypodiaceae	Dasygrammitis crassifrons (Baker) Parris	trnL-trnF	JF514026	FTOL v1.7
Polypodiaceae	Dasygrammitis fuscata (Blume) Parris	rbcL	KM218778	FTOL v1.7
Polypodiaceae	Dasygrammitis fuscata (Blume) Parris	rps4-trnS	KM106138	FTOL v1.7
Polypodiaceae	Dasygrammitis malaccana (Baker) Parris	rbcL	KM218779	FTOL v1.7
Polypodiaceae	Dasygrammitis malaccana (Baker) Parris	rps4-trnS	KM106139	FTOL v1.7
Polypodiaceae	Dasygrammitis mollicoma (Nees & Blume) Parris	rbcL	KM218776	FTOL v1.7
Polypodiaceae	Dasygrammitis mollicoma (Nees & Blume) Parris	trnL-trnF	KM106063	FTOL v1.7
Polypodiaceae	Dasygrammitis purpurascens (Nadeaud) Parris	rbcL	MT216011	FTOL v1.7
Polypodiaceae	Dendroconche ampla (F. Muell. ex Benth.) Testo, Sund	rbcL	KF570108	FTOL v1.7
Polypodiaceae	Dendroconche ampla (F. Muell. ex Benth.) Testo, Sund	rps4	KF570109	FTOL v1.7
Polypodiaceae	Dendroconche ampla (F. Muell. ex Benth.) Testo, Sund	rps4-trnS	KF570109	FTOL v1.7
Polypodiaceae	Dendroconche ampla (F. Muell. ex Benth.) Testo, Sund	trnL-trnF	KF570110	FTOL v1.7
Polypodiaceae	Dendroconche annabellae (H. O. Forbes) Copel.	atpB	MN018179	FTOL v1.7
Polypodiaceae	Dendroconche annabellae (H. O. Forbes) Copel.	rbcL	MN018180	FTOL v1.7
Polypodiaceae	Dendroconche linguiforme (Mett.) Testo, Sundue & A.	atpA	MH113540	FTOL v1.7
Polypodiaceae	Dendroconche linguiforme (Mett.) Testo, Sundue & A.	atpB	MH113573	FTOL v1.7
Polypodiaceae	Dendroconche linguiforme (Mett.) Testo, Sundue & A.	rbcL	MH051174	FTOL v1.7
Polypodiaceae	Dendroconche linguiforme (Mett.) Testo, Sundue & A.	rps4	MH113473	FTOL v1.7
Polypodiaceae	Dendroconche linguiforme (Mett.) Testo, Sundue & A.	rps4-trnS	MH113473	FTOL v1.7
Polypodiaceae	Dendroconche linguiforme (Mett.) Testo, Sundue & A.	trnL-trnF	MH113506	FTOL v1.7
Polypodiaceae	Dendroconche sayeri (F. Muell. & Baker) Testo, Sundue	rbcL	MN018176	FTOL v1.7
Polypodiaceae	Dendroconche sayeri (F. Muell. & Baker) Testo, Sundue	rps4	MN018177	FTOL v1.7
Polypodiaceae	Dendroconche sayeri (F. Muell. & Baker) Testo, Sundue	rps4-trnS	MN018177	FTOL v1.7
Polypodiaceae	Dendroconche sayeri (F. Muell. & Baker) Testo, Sundue	trnL-trnF	MN018178	FTOL v1.7
Polypodiaceae	Dendroconche scandens (G. Forst.) Testo, Sundue & A.	atpA	MH113547	FTOL v1.7
Polypodiaceae	Dendroconche scandens (G. Forst.) Testo, Sundue & A.	atpB	MH113581	FTOL v1.7
Polypodiaceae	Dendroconche scandens (G. Forst.) Testo, Sundue & A.	rbcL	DQ212057	FTOL v1.7
Polypodiaceae	Dendroconche scandens (G. Forst.) Testo, Sundue & A.	rps4	DQ401128	FTOL v1.7
Polypodiaceae	Dendroconche scandens (G. Forst.) Testo, Sundue & A.	rps4-trnS	MH113481	FTOL v1.7
Polypodiaceae	Dendroconche scandens (G. Forst.) Testo, Sundue & A.	trnL-trnF	DQ401125	FTOL v1.7
Polypodiaceae	Dendroconche varians (Mett.) Testo, Sundue & A. R. Fi	rbcL	AY362566	FTOL v1.7
Polypodiaceae	Dendroconche varians (Mett.) Testo, Sundue & A. R. Fi	rps4-trnS	AY362638	FTOL v1.7
Polypodiaceae	Devolia orientalis (T.C.Hsu) Li Bing Zhang, X.M.Zhou, Ji	atpB	OQ702730	FTOL v1.7
Polypodiaceae	Devolia orientalis (T.C.Hsu) Li Bing Zhang, X.M.Zhou, Ji	rps4	OQ717274	FTOL v1.7
Polypodiaceae	Devolia orientalis (T.C.Hsu) Li Bing Zhang, X.M.Zhou, Ji	rps4-trnS	OQ717274	FTOL v1.7
Polypodiaceae	Dictymia brownii (Wikstr.) Copel.	plastome	MW876312	FTOL v1.7
Polypodiaceae	Dictymia mckeei Tindale	atpA	EF463810	FTOL v1.7
Polypodiaceae	Dictymia mckeei Tindale	atpB	EF463492	FTOL v1.7
Polypodiaceae	Dictymia mckeei Tindale	rbcL	DQ164441	FTOL v1.7
Polypodiaceae	Dictymia mckeei Tindale	rps4	DQ164472	FTOL v1.7
Polypodiaceae	Dictymia mckeei Tindale	rps4-trnS	DQ164472	FTOL v1.7
Polypodiaceae	Dictymia mckeei Tindale	trnL-trnF	DQ164504	FTOL v1.7
Polypodiaceae	Drynaria baronii (Christ) Diels	plastome	MK789653	FTOL v1.7
Polypodiaceae	Drynaria bonii Christ	plastome	MK761229	FTOL v1.7
Polypodiaceae	Drynaria coronans (Wall. ex Mett.) J. Sm.	plastome	MK761244	FTOL v1.7
Polypodiaceae	Drynaria delavayi Christ	plastome	MK761240	FTOL v1.7
Polypodiaceae	Drynaria descensa Copel.	rbcL	AY529159	FTOL v1.7
Polypodiaceae	Drynaria descensa Copel.	rps4	AY529183	FTOL v1.7
Polypodiaceae	Drynaria descensa Copel.	rps4-trnS	AY529183	FTOL v1.7
Polypodiaceae	Drynaria descensa Copel.	trnL-trnF	AY529476	FTOL v1.7
Polypodiaceae	Drynaria drynarioides (Hook.) Christenh.	rbcL	AY529149	FTOL v1.7
Polypodiaceae	Drynaria drynarioides (Hook.) Christenh.	rps4	AY529174	FTOL v1.7
Polypodiaceae	Drynaria drynarioides (Hook.) Christenh.	rps4-trnS	AY529174	FTOL v1.7
Polypodiaceae	Drynaria drynarioides (Hook.) Christenh.	trnL-trnF	AY529465	FTOL v1.7
Polypodiaceae	Drynaria fortunei (Kunze ex Mett.) J. Sm.	plastome	MK761238	FTOL v1.7
Polypodiaceae	Drynaria heraclea (Kunze) T. Moore	plastome	MW876314	FTOL v1.7
Polypodiaceae	Drynaria hieronymi (Brause) Christenh.	rbcL	AY529151	FTOL v1.7
Polypodiaceae	Drynaria hieronymi (Brause) Christenh.	rps4	AY529176	FTOL v1.7
Polypodiaceae	Drynaria hieronymi (Brause) Christenh.	rps4-trnS	AY529176	FTOL v1.7
Polypodiaceae	Drynaria hieronymi (Brause) Christenh.	trnL-trnF	AY529467	FTOL v1.7
Polypodiaceae	Drynaria latipinna (C. Chr.) Christenh.	trnL-trnF	AY529468	FTOL v1.7
Polypodiaceae	Drynaria laurentii (Christ) Hieron.	rbcL	AY529161	FTOL v1.7
Polypodiaceae	Drynaria laurentii (Christ) Hieron.	rps4	AY529185	FTOL v1.7
Polypodiaceae	Drynaria laurentii (Christ) Hieron.	rps4-trnS	AY529185	FTOL v1.7
Polypodiaceae	Drynaria laurentii (Christ) Hieron.	trnL-trnF	AY529478	FTOL v1.7
Polypodiaceae	Drynaria leporella K.I.Goebel	rbcL	AY529152	FTOL v1.7
Polypodiaceae	Drynaria leporella K.I.Goebel	rps4	AY529177	FTOL v1.7
Polypodiaceae	Drynaria leporella K.I.Goebel	rps4-trnS	AY529177	FTOL v1.7
Polypodiaceae	Drynaria leporella K.I.Goebel	trnL-trnF	AY529469	FTOL v1.7
Polypodiaceae	Drynaria meyeniana (Schott) Christenh.	rbcL	AY529153	FTOL v1.7
Polypodiaceae	Drynaria meyeniana (Schott) Christenh.	trnL-trnF	AY529470	FTOL v1.7
Polypodiaceae	Drynaria mollis Bedd.	rbcL	AY529162	FTOL v1.7
Polypodiaceae	Drynaria mollis Bedd.	rps4	AY529186	FTOL v1.7
Polypodiaceae	Drynaria mollis Bedd.	rps4-trnS	AY529186	FTOL v1.7
Polypodiaceae	Drynaria mollis Bedd.	trnL-trnF	EU571225	FTOL v1.7
Polypodiaceae	Drynaria novoguineensis (Brause) Christenh.	rbcL	AY529154	FTOL v1.7
Polypodiaceae	Drynaria novoguineensis (Brause) Christenh.	rps4	AY529178	FTOL v1.7
Polypodiaceae	Drynaria novoguineensis (Brause) Christenh.	rps4-trnS	AY529178	FTOL v1.7
Polypodiaceae	Drynaria novoguineensis (Brause) Christenh.	trnL-trnF	AY529471	FTOL v1.7
Polypodiaceae	Drynaria parishii (Bedd.) Bedd.	plastome	MW876315	FTOL v1.7
Polypodiaceae	Drynaria parkinsonii (Baker) Diels	rbcL	AY529155	FTOL v1.7
Polypodiaceae	Drynaria parkinsonii (Baker) Diels	rps4	AY529179	FTOL v1.7
Polypodiaceae	Drynaria parkinsonii (Baker) Diels	rps4-trnS	AY529179	FTOL v1.7
Polypodiaceae	Drynaria parkinsonii (Baker) Diels	trnL-trnF	AY529472	FTOL v1.7
Polypodiaceae	Drynaria pilosa (J. Sm.) Christenh.	rbcL	AY529156	FTOL v1.7
Polypodiaceae	Drynaria pilosa (J. Sm.) Christenh.	rps4	AY529180	FTOL v1.7
Polypodiaceae	Drynaria pilosa (J. Sm.) Christenh.	rps4-trnS	AY529180	FTOL v1.7
Polypodiaceae	Drynaria pilosa (J. Sm.) Christenh.	trnL-trnF	AY529473	FTOL v1.7
Polypodiaceae	Drynaria propinqua (Wall. ex Mett.) J. Sm. ex Bedd.	plastome	MT130648	FTOL v1.7
Polypodiaceae	Drynaria quercifolia (L.) J. Sm.	plastome	MT130596	FTOL v1.7
Polypodiaceae	Drynaria rigidula (Sw.) Bedd.	atpA	EF463811	FTOL v1.7
Polypodiaceae	Drynaria rigidula (Sw.) Bedd.	atpB	EF463493	FTOL v1.7
Polypodiaceae	Drynaria rigidula (Sw.) Bedd.	rbcL	AF470339	FTOL v1.7
Polypodiaceae	Drynaria rigidula (Sw.) Bedd.	rps4	JX103733	FTOL v1.7
Polypodiaceae	Drynaria rigidula (Sw.) Bedd.	rps4-trnS	AY529188	FTOL v1.7
Polypodiaceae	Drynaria rigidula (Sw.) Bedd.	trnL-trnF	EU571223	FTOL v1.7
Polypodiaceae	Drynaria sagitta (Sw.) Christenh.	rbcL	EU128498	FTOL v1.7
Polypodiaceae	Drynaria sagitta (Sw.) Christenh.	rps4	EU128505	FTOL v1.7
Polypodiaceae	Drynaria sagitta (Sw.) Christenh.	rps4-trnS	EU128505	FTOL v1.7
Polypodiaceae	Drynaria sagitta (Sw.) Christenh.	trnL-trnF	EU128512	FTOL v1.7
Polypodiaceae	Drynaria sparsisora (Desv.) T. Moore	rbcL	AY529167	FTOL v1.7
Polypodiaceae	Drynaria sparsisora (Desv.) T. Moore	rps4	AY529189	FTOL v1.7
Polypodiaceae	Drynaria sparsisora (Desv.) T. Moore	rps4-trnS	AY529189	FTOL v1.7
Polypodiaceae	Drynaria sparsisora (Desv.) T. Moore	trnL-trnF	AY529482	FTOL v1.7
Polypodiaceae	Drynaria speciosa (Blume) Christenh.	plastome	MW876316	FTOL v1.7
Polypodiaceae	Drynaria splendens (J. Sm.) Bedd.	rbcL	AY529157	FTOL v1.7
Polypodiaceae	Drynaria splendens (J. Sm.) Bedd.	rps4	AY529181	FTOL v1.7
Polypodiaceae	Drynaria splendens (J. Sm.) Bedd.	rps4-trnS	AY529181	FTOL v1.7
Polypodiaceae	Drynaria splendens (J. Sm.) Bedd.	trnL-trnF	AY529474	FTOL v1.7
Polypodiaceae	Drynaria tricuspis (Hook.) Christenh.	trnL-trnF	EU128514	FTOL v1.7
Polypodiaceae	Drynaria volkensii Hieron.	rbcL	AY529168	FTOL v1.7
Polypodiaceae	Drynaria volkensii Hieron.	rps4	AY529190	FTOL v1.7
Polypodiaceae	Drynaria volkensii Hieron.	rps4-trnS	AY529190	FTOL v1.7

Polypodiaceae	Drynaria willdenowii (Bory) T. Moore	plastome	MW876317	FTOL v1.7
Polypodiaceae	Enterosora barbatula (Baker) Parris	atpB	KY711781	FTOL v1.7
Polypodiaceae	Enterosora barbatula (Baker) Parris	rbcL	KY711956	FTOL v1.7
Polypodiaceae	Enterosora barbatula (Baker) Parris	rps4-trnS	KY712281	FTOL v1.7
Polypodiaceae	Enterosora barbatula (Baker) Parris	trnL-trnF	KY711607	FTOL v1.7
Polypodiaceae	Enterosora campbellii Baker	rbcL	MK319107	FTOL v1.7
Polypodiaceae	Enterosora campbellii Baker	trnL-trnF	MK319005	FTOL v1.7
Polypodiaceae	Enterosora cornuta (Lellinger) Shalisko & Sundue	rbcL	MK319139	FTOL v1.7
Polypodiaceae	Enterosora cornuta (Lellinger) Shalisko & Sundue	trnL-trnF	MK319043	FTOL v1.7
Polypodiaceae	Enterosora devoluta (Baker) Shalisko & Sundue	atpB	KY711855	FTOL v1.7
Polypodiaceae	Enterosora devoluta (Baker) Shalisko & Sundue	rbcL	KY712030	FTOL v1.7
Polypodiaceae	Enterosora devoluta (Baker) Shalisko & Sundue	rps4-trnS	KY712347	FTOL v1.7
Polypodiaceae	Enterosora devoluta (Baker) Shalisko & Sundue	trnL-trnF	KY711679	FTOL v1.7
Polypodiaceae	Enterosora enterosoroides (Christ) A. Rojas	atpB	GU476756	FTOL v1.7
Polypodiaceae	Enterosora enterosoroides (Christ) A. Rojas	rps4-trnS	KM106140	FTOL v1.7
Polypodiaceae	Enterosora enterosoroides (Christ) A. Rojas	trnL-trnF	GU476634	FTOL v1.7
Polypodiaceae	Enterosora forsythiana (Baker) Shalisko & Sundue	atpB	KY711858	FTOL v1.7
Polypodiaceae	Enterosora forsythiana (Baker) Shalisko & Sundue	rbcL	KY712033	FTOL v1.7
Polypodiaceae	Enterosora forsythiana (Baker) Shalisko & Sundue	rps4-trnS	KY712350	FTOL v1.7
Polypodiaceae	Enterosora forsythiana (Baker) Shalisko & Sundue	trnL-trnF	KY711682	FTOL v1.7
Polypodiaceae	Enterosora goodmanii (Rakotondr.) Shalisko & Sundue	atpB	KY711863	FTOL v1.7
Polypodiaceae	Enterosora goodmanii (Rakotondr.) Shalisko & Sundue	rbcL	KY712038	FTOL v1.7
Polypodiaceae	Enterosora goodmanii (Rakotondr.) Shalisko & Sundue	rps4-trnS	KY712355	FTOL v1.7
Polypodiaceae	Enterosora goodmanii (Rakotondr.) Shalisko & Sundue	trnL-trnF	KY711686	FTOL v1.7
Polypodiaceae	Enterosora humbertii (C. Chr.) Shalisko & Sundue	atpB	KY711866	FTOL v1.7
Polypodiaceae	Enterosora humbertii (C. Chr.) Shalisko & Sundue	rbcL	KY712041	FTOL v1.7
Polypodiaceae	Enterosora humbertii (C. Chr.) Shalisko & Sundue	rps4-trnS	KY712358	FTOL v1.7
Polypodiaceae	Enterosora humbertii (C. Chr.) Shalisko & Sundue	trnL-trnF	KY711688	FTOL v1.7
Polypodiaceae	Enterosora mathewsii (Kunze ex Mett.) Shalisko & Sun	atpB	KY775306	FTOL v1.7
Polypodiaceae	Enterosora mathewsii (Kunze ex Mett.) Shalisko & Sun	rbcL	GU476895	FTOL v1.7
Polypodiaceae	Enterosora mathewsii (Kunze ex Mett.) Shalisko & Sun	rps4-trnS	KY775305	FTOL v1.7
Polypodiaceae	Enterosora mathewsii (Kunze ex Mett.) Shalisko & Sun	trnL-trnF	GU476743	FTOL v1.7
Polypodiaceae	Enterosora percrassa (Baker) L. E. Bishop	atpB	AY459468	FTOL v1.7
Polypodiaceae	Enterosora percrassa (Baker) L. E. Bishop	rbcL	GU476882	FTOL v1.7
Polypodiaceae	Enterosora percrassa (Baker) L. E. Bishop	rps4-trnS	KM106141	FTOL v1.7
Polypodiaceae	Enterosora percrassa (Baker) L. E. Bishop	trnL-trnF	GU476636	FTOL v1.7
Polypodiaceae	Enterosora sectifrons (Kunze ex Mett.) Shalisko & Sunc	rbcL	MK319140	FTOL v1.7
Polypodiaceae	Enterosora sectifrons (Kunze ex Mett.) Shalisko & Sunc	rps4-trnS	KY775304	FTOL v1.7
Polypodiaceae	Enterosora sectifrons (Kunze ex Mett.) Shalisko & Sunc	trnL-trnF	MK319044	FTOL v1.7
Polypodiaceae	Enterosora subpinnata (Baker) Shalisko & Sundue	atpB	KY711875	FTOL v1.7
Polypodiaceae	Enterosora subpinnata (Baker) Shalisko & Sundue	rbcL	KY712050	FTOL v1.7
Polypodiaceae	Enterosora subpinnata (Baker) Shalisko & Sundue	rps4-trnS	KY712367	FTOL v1.7
Polypodiaceae	Enterosora subpinnata (Baker) Shalisko & Sundue	trnL-trnF	KY711697	FTOL v1.7
Polypodiaceae	Enterosora torulosa (Baker) Shalisko & Sundue	atpB	KY711884	FTOL v1.7
Polypodiaceae	Enterosora torulosa (Baker) Shalisko & Sundue	rbcL	KF992465	FTOL v1.7
Polypodiaceae	Enterosora torulosa (Baker) Shalisko & Sundue	rps4-trnS	KY712376	FTOL v1.7
Polypodiaceae	Enterosora torulosa (Baker) Shalisko & Sundue	trnL-trnF	KY711701	FTOL v1.7
Polypodiaceae	Enterosora trichosora (Hook.) L. E. Bishop	rbcL	GU476920	FTOL v1.7
Polypodiaceae	Enterosora trichosora (Hook.) L. E. Bishop	trnL-trnF	GU476637	FTOL v1.7
Polypodiaceae	Enterosora trifurcata (L.) L. E. Bishop	atpB	GU476754	FTOL v1.7
Polypodiaceae	Enterosora trifurcata (L.) L. E. Bishop	rbcL	GU476909	FTOL v1.7
Polypodiaceae	Enterosora trifurcata (L.) L. E. Bishop	trnL-trnF	GU476632	FTOL v1.7
Polypodiaceae	Enterosora villosissima (Hook.) Shalisko & Sundue	atpB	KY711892	FTOL v1.7
Polypodiaceae	Enterosora villosissima (Hook.) Shalisko & Sundue	rbcL	KY712066	FTOL v1.7
Polypodiaceae	Enterosora villosissima (Hook.) Shalisko & Sundue	rps4-trnS	KY712385	FTOL v1.7
Polypodiaceae	Enterosora villosissima (Hook.) Shalisko & Sundue	trnL-trnF	KY711713	FTOL v1.7
Polypodiaceae	Galactodenia delicatula (M. Martens & Galeotti) Sundı	atpB	GU386961	FTOL v1.7
Polypodiaceae	Galactodenia delicatula (M. Martens & Galeotti) Sundı	rbcL	MK319102	FTOL v1.7
Polypodiaceae	Galactodenia delicatula (M. Martens & Galeotti) Sundı	rps4-trnS	GU387123	FTOL v1.7
Polypodiaceae	Galactodenia delicatula (M. Martens & Galeotti) Sundı	trnL-trnF	GU476715	FTOL v1.7
Polypodiaceae	Galactodenia parrisiae Sundue & Labiak	rbcL	KM218794	FTOL v1.7
Polypodiaceae	Galactodenia subscabra (Klotzsch) Sundue & Labiak	atpB	GU476821	FTOL v1.7
Polypodiaceae	Galactodenia subscabra (Klotzsch) Sundue & Labiak	rbcL	GU476860	FTOL v1.7
Polypodiaceae	Galactodenia subscabra (Klotzsch) Sundue & Labiak	rps4-trnS	GU387127	FTOL v1.7
Polypodiaceae	Galactodenia subscabra (Klotzsch) Sundue & Labiak	trnL-trnF	GU476739	FTOL v1.7
Polypodiaceae	Goniophlebium amoenum (Wall. ex Mett) J. Sm.	plastome	MT130635	FTOL v1.7
Polypodiaceae	Goniophlebium argutum (Wall. ex Hook.) J. Sm.	plastome	MW876319	FTOL v1.7
Polypodiaceae	Goniophlebium fieldingianum (Kunze ex Mett.) T. Moo	rbcL	DQ078627	FTOL v1.7
Polypodiaceae	Goniophlebium fieldingianum (Kunze ex Mett.) T. Moo	rps4	DQ078632	FTOL v1.7
Polypodiaceae	Goniophlebium fieldingianum (Kunze ex Mett.) T. Moo	rps4-trnS	DQ078632	FTOL v1.7
Polypodiaceae	Goniophlebium formosanum (Baker) Rödl-Linder	plastome	MW876320	FTOL v1.7
Polypodiaceae	Goniophlebium manmeiense (Christ) Rödl-Linder	plastome	MW876321	FTOL v1.7
Polypodiaceae	Goniophlebium mehibitense (C.Chr.) Parris	rbcL	EU482932	FTOL v1.7
Polypodiaceae	Goniophlebium mehibitense (C.Chr.) Parris	trnL-trnF	EU483026	FTOL v1.7
Polypodiaceae	Goniophlebium mengtzeense (Christ) Rödl-Linder	plastome	MW876322	FTOL v1.7
Polypodiaceae	Goniophlebium niponicum (Mett.) Bedd.	plastome	MW876323	FTOL v1.7
Polypodiaceae	Goniophlebium percussum (Cav.) Wagner & Grether	rbcL	AY362561	FTOL v1.7
Polypodiaceae	Goniophlebium persicifolium (Desv.) Bedd.	plastome	MT130634	FTOL v1.7
Polypodiaceae	Goniophlebium pseudoconnatum (Copel.) Copel.	rbcL	EU482934	FTOL v1.7
Polypodiaceae	Goniophlebium pseudoconnatum (Copel.) Copel.	trnL-trnF	EU483029	FTOL v1.7
Polypodiaceae	Goniophlebium someyae var. awaense (Tagawa) Naka	rbcL	AB575291	FTOL v1.7
Polypodiaceae	Goniophlebium subamoenum (C. B. Clarke) Bedd.	rbcL	DQ078629	FTOL v1.7
Polypodiaceae	Goniophlebium subauriculatum (Blume) C. Presl	rbcL	AF470342	FTOL v1.7
Polypodiaceae	Goniophlebium wattii (Bedd.) Panigrahi & Sarn. Singh	rbcL	DQ078625	FTOL v1.7
Polypodiaceae	Goniophlebium wattii (Bedd.) Panigrahi & Sarn. Singh	rps4	DQ078636	FTOL v1.7
Polypodiaceae	Goniophlebium wattii (Bedd.) Panigrahi & Sarn. Singh	rps4-trnS	DQ078636	FTOL v1.7
Polypodiaceae	Goniophlebium yunnanense (Franch.) Bedd.	plastome	MT130566	FTOL v1.7
Polypodiaceae	Grammitastrum pseudaustrale (E.Fourn.) Parris, Sundı	atpB	KY711867	FTOL v1.7
Polypodiaceae	Grammitastrum pseudaustrale (E.Fourn.) Parris, Sundı	rbcL	KY712042	FTOL v1.7
Polypodiaceae	Grammitastrum pseudaustrale (E.Fourn.) Parris, Sundı	rps4-trnS	KY712359	FTOL v1.7
Polypodiaceae	Grammitastrum pseudaustrale (E.Fourn.) Parris, Sundı	trnL-trnF	KM106068	FTOL v1.7
Polypodiaceae	Grammitis bryophila (Maxon) F.Seym.	atpB	AF469784	FTOL v1.7
Polypodiaceae	Grammitis bryophila (Maxon) F.Seym.	rbcL	AF468208	FTOL v1.7
Polypodiaceae	Grammitis bryophila (Maxon) F.Seym.	trnL-trnF	AF469797	FTOL v1.7
Polypodiaceae	Grammitis bufonis L.D.Go?mez	rbcL	MK319109	FTOL v1.7
Polypodiaceae	Grammitis bufonis L.D.Go?mez	trnL-trnF	MK319007	FTOL v1.7
Polypodiaceae	Grammitis cincta Copel. ex Parris	rbcL	MT216033	FTOL v1.7
Polypodiaceae	Grammitis copelandii Tardieu	atpB	KY711882	FTOL v1.7
Polypodiaceae	Grammitis copelandii Tardieu	rbcL	KY712057	FTOL v1.7
Polypodiaceae	Grammitis copelandii Tardieu	rps4-trnS	KY712374	FTOL v1.7
Polypodiaceae	Grammitis copelandii Tardieu	trnL-trnF	KY711703	FTOL v1.7
Polypodiaceae	Grammitis coriaceifolia Rakotondr. & Parris	atpB	KY711796	FTOL v1.7
Polypodiaceae	Grammitis coriaceifolia Rakotondr. & Parris	rbcL	KY711971	FTOL v1.7
Polypodiaceae	Grammitis coriaceifolia Rakotondr. & Parris	rps4-trnS	KY712294	FTOL v1.7
Polypodiaceae	Grammitis coriaceifolia Rakotondr. & Parris	trnL-trnF	KY711620	FTOL v1.7
Polypodiaceae	Grammitis cryptophlebia (Baker) Copel.	atpB	KY711885	FTOL v1.7
Polypodiaceae	Grammitis cryptophlebia (Baker) Copel.	rbcL	KY712059	FTOL v1.7
Polypodiaceae	Grammitis cryptophlebia (Baker) Copel.	rps4-trnS	KY712377	FTOL v1.7
Polypodiaceae	Grammitis cryptophlebia (Baker) Copel.	trnL-trnF	KY711705	FTOL v1.7
Polypodiaceae	Grammitis ebenina (Maxon) Tardieu	atpB	KY711825	FTOL v1.7
Polypodiaceae	Grammitis ebenina (Maxon) Tardieu	rbcL	KY712001	FTOL v1.7
Polypodiaceae	Grammitis ebenina (Maxon) Tardieu	rps4-trnS	KY712320	FTOL v1.7
Polypodiaceae	Grammitis ebenina (Maxon) Tardieu	trnL-trnF	KY711650	FTOL v1.7
Polypodiaceae	Grammitis kyimbilensis (Brause) Copel.	atpB	KY711814	FTOL v1.7
Polypodiaceae	Grammitis kyimbilensis (Brause) Copel.	rbcL	KY711989	FTOL v1.7
Polypodiaceae	Grammitis kyimbilensis (Brause) Copel.	rps4-trnS	KY712311	FTOL v1.7
Polypodiaceae	Grammitis kyimbilensis (Brause) Copel.	trnL-trnF	KY711638	FTOL v1.7
Polypodiaceae	Grammitis limbata Fe?e	rbcL	MK319141	FTOL v1.7
Polypodiaceae	Grammitis limbata Fe?e	trnL-trnF	MK319008	FTOL v1.7
Polypodiaceae	Grammitis marginella (Sw.) Sw.	plastome	OQ968266	FTOL v1.7
Polypodiaceae	Grammitis marginelloides (J.W.Moore) Copel.	rbcL	KY099803	FTOL v1.7
Polypodiaceae	Grammitis melanoloma (Cordem.) Tardieu	atpB	AY459475	FTOL v1.7
Polypodiaceae	Grammitis melanoloma (Cordem.) Tardieu	rbcL	AY460643	FTOL v1.7
Polypodiaceae	Grammitis melanoloma (Cordem.) Tardieu	rps4-trnS	KY712313	FTOL v1.7
Polypodiaceae	Grammitis melanoloma (Cordem.) Tardieu	trnL-trnF	GU476641	FTOL v1.7
Polypodiaceae	Grammitis nudicarpa Copel.	rbcL	JF950810	FTOL v1.7
Polypodiaceae	Grammitis nudicarpa Copel.	trnL-trnF	JF950911	FTOL v1.7
Polypodiaceae	Grammitis paramicola L.E.Bishop	atpB	KM218816	FTOL v1.7
Polypodiaceae	Grammitis paramicola L.E.Bishop	rbcL	KM218801	FTOL v1.7
Polypodiaceae	Grammitis paramicola L.E.Bishop	rps4-trnS	KM106149	FTOL v1.7
Polypodiaceae	Grammitis paramicola L.E.Bishop	trnL-trnF	KM106067	FTOL v1.7
Polypodiaceae	Gymnogrammitis dareiformis (Hook.) Ching ex Tardieu	plastome	MW876368	FTOL v1.7
Polypodiaceae	Hovenkampia liebuschii (Hieron.) Li Bing Zhang & X. M	trnL-trnF	DQ642253	FTOL v1.7
Polypodiaceae	Hovenkampia schimperiana (Mett. ex Kuhn) Li Bing Zha	plastome	MW876325	FTOL v1.7
Polypodiaceae	Howeogrammitis diminuta (Baker) Parris, Sundue, Li B	rbcL	JF950809	FTOL v1.7
Polypodiaceae	Howeogrammitis diminuta (Baker) Parris, Sundue, Li B	trnL-trnF	JF950910	FTOL v1.7
Polypodiaceae	Lecanopteris balgooyi Hennipman	rbcL	AF470328	FTOL v1.7
Polypodiaceae	Lecanopteris balgooyi Hennipman	trnL-trnF	AY083631	FTOL v1.7
Polypodiaceae	Lecanopteris carnosa (Reinw.) Blume	rbcL	AF470322	FTOL v1.7
Polypodiaceae	Lecanopteris carnosa (Reinw.) Blume	trnL-trnF	AY083625	FTOL v1.7
Polypodiaceae	Lecanopteris celebica Hennipman	rbcL	AF470323	FTOL v1.7
Polypodiaceae	Lecanopteris celebica Hennipman	trnL-trnF	AY083626	FTOL v1.7
Polypodiaceae	Lecanopteris crustacea Copel.	rbcL	AF470329	FTOL v1.7
Polypodiaceae	Lecanopteris deparioides (Ces.) Baker	rbcL	AF470324	FTOL v1.7
Polypodiaceae	Lecanopteris deparioides (Ces.) Baker	trnL-trnF	AY083627	FTOL v1.7
Polypodiaceae	Lecanopteris luzonensis Hennipman	rbcL	AF470325	FTOL v1.7
Polypodiaceae	Lecanopteris luzonensis Hennipman	trnL-trnF	AY083628	FTOL v1.7
Polypodiaceae	Lecanopteris mirabilis Copel.	rbcL	AF470330	FTOL v1.7
Polypodiaceae	Lecanopteris mirabilis Copel.	trnL-trnF	AY083633	FTOL v1.7
Polypodiaceae	Lecanopteris pumila Blume	rbcL	AF470331	FTOL v1.7
Polypodiaceae	Lecanopteris pumila Blume	trnL-trnF	AY083634	FTOL v1.7
Polypodiaceae	Lecanopteris sarcopus (Teijsm. & Binn.) Copel.	rbcL	EU482935	FTOL v1.7
Polypodiaceae	Lecanopteris sarcopus (Teijsm. & Binn.) Copel.	trnL-trnF	EU483030	FTOL v1.7
Polypodiaceae	Lecanopteris sinuosa (Wall. ex Hook.) Copel.	rbcL	AF470321	FTOL v1.7
Polypodiaceae	Lecanopteris sinuosa (Wall. ex Hook.) Copel.	trnL-trnF	AY083624	FTOL v1.7
Polypodiaceae	Lecanopteris spinosa Jermy & T. Walker	rbcL	AF470327	FTOL v1.7
Polypodiaceae	Lecanopteris spinosa Jermy & T. Walker	trnL-trnF	AY083630	FTOL v1.7
Polypodiaceae	Lellingeria affinis Labiak	atpB	GU376570	FTOL v1.7
Polypodiaceae	Lellingeria affinis Labiak	rbcL	GU386982	FTOL v1.7
Polypodiaceae	Lellingeria affinis Labiak	rps4-trnS	GU387044	FTOL v1.7
Polypodiaceae	Lellingeria affinis Labiak	trnL-trnF	GU387212	FTOL v1.7
Polypodiaceae	Lellingeria apiculata (Kunze ex Klotzsch) A. R. Sm. & R.	atpB	GU376573	FTOL v1.7
Polypodiaceae	Lellingeria apiculata (Kunze ex Klotzsch) A. R. Sm. & R.	rbcL	GU387025	FTOL v1.7
Polypodiaceae	Lellingeria apiculata (Kunze ex Klotzsch) A. R. Sm. & R.	rps4-trnS	GU387047	FTOL v1.7
Polypodiaceae	Lellingeria apiculata (Kunze ex Klotzsch) A. R. Sm. & R.	trnL-trnF	GU387215	FTOL v1.7
Polypodiaceae	Lellingeria bishopii Labiak	atpB	GU376574	FTOL v1.7
Polypodiaceae	Lellingeria bishopii Labiak	rbcL	GU476878	FTOL v1.7
Polypodiaceae	Lellingeria bishopii Labiak	rps4-trnS	GU387048	FTOL v1.7
Polypodiaceae	Lellingeria bishopii Labiak	trnL-trnF	GU387216	FTOL v1.7
Polypodiaceae	Lellingeria brevistipes (Mett. ex Kuhn) A. R. Sm. & R. C	plastome	MZ357088	FTOL v1.7
Polypodiaceae	Lellingeria calolepis Labiak	atpB	GU376577	FTOL v1.7
Polypodiaceae	Lellingeria calolepis Labiak	rbcL	GU386979	FTOL v1.7
Polypodiaceae	Lellingeria calolepis Labiak	rps4-trnS	GU387051	FTOL v1.7
Polypodiaceae	Lellingeria calolepis Labiak	trnL-trnF	GU387219	FTOL v1.7
Polypodiaceae	Lellingeria cantarensis B. K. Black & Sundue	atpB	MZ147036	FTOL v1.7
Polypodiaceae	Lellingeria cantarensis B. K. Black & Sundue	rbcL	MZ147035	FTOL v1.7
Polypodiaceae	Lellingeria cantarensis B. K. Black & Sundue	rps4-trnS	MZ147034	FTOL v1.7
Polypodiaceae	Lellingeria cantarensis B. K. Black & Sundue	trnL-trnF	MZ147039	FTOL v1.7
Polypodiaceae	Lellingeria ciliolepis (C. Chr.) A. R. Sm.	atpB	GU376578	FTOL v1.7
Polypodiaceae	Lellingeria ciliolepis (C. Chr.) A. R. Sm.	rbcL	GU387016	FTOL v1.7
Polypodiaceae	Lellingeria ciliolepis (C. Chr.) A. R. Sm.	rps4-trnS	GU387052	FTOL v1.7
Polypodiaceae	Lellingeria ciliolepis (C. Chr.) A. R. Sm.	trnL-trnF	GU387220	FTOL v1.7
Polypodiaceae	Lellingeria depressa (C. Chr.) A. R. Sm. & R. C. Moran	atpB	GU376579	FTOL v1.7
Polypodiaceae	Lellingeria depressa (C. Chr.) A. R. Sm. & R. C. Moran	rbcL	GU387017	FTOL v1.7
Polypodiaceae	Lellingeria depressa (C. Chr.) A. R. Sm. & R. C. Moran	rps4-trnS	GU387053	FTOL v1.7
Polypodiaceae	Lellingeria depressa (C. Chr.) A. R. Sm. & R. C. Moran	trnL-trnF	GU387221	FTOL v1.7
Polypodiaceae	Lellingeria dissimulans (Maxon) A. R. Sm.	atpB	GU376580	FTOL v1.7

Polypodiaceae	Lellingeria dissimulans (Maxon) A. R. Sm.	rbcL	GU387043	FTOL v1.7
Polypodiaceae	Lellingeria dissimulans (Maxon) A. R. Sm.	rps4-trnS	GU387054	FTOL v1.7
Polypodiaceae	Lellingeria dissimulans (Maxon) A. R. Sm.	trnL-trnF	GU387222	FTOL v1.7
Polypodiaceae	Lellingeria flagellipinnata M. Kessler & A. R. Sm.	atpB	GU376581	FTOL v1.7
Polypodiaceae	Lellingeria flagellipinnata M. Kessler & A. R. Sm.	rbcL	GU387015	FTOL v1.7
Polypodiaceae	Lellingeria flagellipinnata M. Kessler & A. R. Sm.	rps4-trnS	GU387055	FTOL v1.7
Polypodiaceae	Lellingeria flagellipinnata M. Kessler & A. R. Sm.	trnL-trnF	GU387223	FTOL v1.7
Polypodiaceae	Lellingeria hombersleyi (Maxon) A. R. Sm.	rbcL	MW138141	FTOL v1.7
Polypodiaceae	Lellingeria humilis (Mett.) A. R. Sm. & R. C. Moran	atpB	GU476763	FTOL v1.7
Polypodiaceae	Lellingeria humilis (Mett.) A. R. Sm. & R. C. Moran	rbcL	GU476897	FTOL v1.7
Polypodiaceae	Lellingeria humilis (Mett.) A. R. Sm. & R. C. Moran	trnL-trnF	GU476649	FTOL v1.7
Polypodiaceae	Lellingeria isidrensis (Maxon ex Copel.) A. R. Sm. & R. (	atpB	GU376586	FTOL v1.7
Polypodiaceae	Lellingeria isidrensis (Maxon ex Copel.) A. R. Sm. & R. (	rbcL	GU387042	FTOL v1.7
Polypodiaceae	Lellingeria isidrensis (Maxon ex Copel.) A. R. Sm. & R. (	rps4-trnS	GU387061	FTOL v1.7
Polypodiaceae	Lellingeria isidrensis (Maxon ex Copel.) A. R. Sm. & R. (	trnL-trnF	GU387228	FTOL v1.7
Polypodiaceae	Lellingeria itatimensis (C. Chr.) A. R. Sm. & R. C. Morar	atpB	GU376587	FTOL v1.7
Polypodiaceae	Lellingeria itatimensis (C. Chr.) A. R. Sm. & R. C. Morar	rbcL	GU387020	FTOL v1.7
Polypodiaceae	Lellingeria itatimensis (C. Chr.) A. R. Sm. & R. C. Morar	rps4-trnS	GU387062	FTOL v1.7
Polypodiaceae	Lellingeria itatimensis (C. Chr.) A. R. Sm. & R. C. Morar	trnL-trnF	GU387229	FTOL v1.7
Polypodiaceae	Lellingeria jimenezii Labiak	atpB	GU376589	FTOL v1.7
Polypodiaceae	Lellingeria jimenezii Labiak	rbcL	GU386986	FTOL v1.7
Polypodiaceae	Lellingeria jimenezii Labiak	rps4-trnS	GU387064	FTOL v1.7
Polypodiaceae	Lellingeria jimenezii Labiak	trnL-trnF	GU387230	FTOL v1.7
Polypodiaceae	Lellingeria kaieteura (Jenman) Labiak	atpB	GU376590	FTOL v1.7
Polypodiaceae	Lellingeria kaieteura (Jenman) Labiak	rbcL	GU386978	FTOL v1.7
Polypodiaceae	Lellingeria kaieteura (Jenman) Labiak	rps4-trnS	GU387065	FTOL v1.7
Polypodiaceae	Lellingeria kaieteura (Jenman) Labiak	trnL-trnF	GU387231	FTOL v1.7
Polypodiaceae	Lellingeria major (Copel.) A. R. Sm. & R. C. Moran	atpB	GU376592	FTOL v1.7
Polypodiaceae	Lellingeria major (Copel.) A. R. Sm. & R. C. Moran	rbcL	GU476876	FTOL v1.7
Polypodiaceae	Lellingeria major (Copel.) A. R. Sm. & R. C. Moran	rps4-trnS	GU387069	FTOL v1.7
Polypodiaceae	Lellingeria major (Copel.) A. R. Sm. & R. C. Moran	trnL-trnF	GU387233	FTOL v1.7
Polypodiaceae	Lellingeria melanotrichia (Baker) A. R. Sm. & R. C. Mor	atpB	GU376593	FTOL v1.7
Polypodiaceae	Lellingeria melanotrichia (Baker) A. R. Sm. & R. C. Mor	rbcL	GU386973	FTOL v1.7
Polypodiaceae	Lellingeria melanotrichia (Baker) A. R. Sm. & R. C. Mor	rps4-trnS	GU387070	FTOL v1.7
Polypodiaceae	Lellingeria melanotrichia (Baker) A. R. Sm. & R. C. Mor	trnL-trnF	GU387234	FTOL v1.7
Polypodiaceae	Lellingeria oreophila (Maxon) A. R. Sm. & R. C. Moran	atpB	GU386919	FTOL v1.7
Polypodiaceae	Lellingeria oreophila (Maxon) A. R. Sm. & R. C. Moran	rbcL	GU386974	FTOL v1.7
Polypodiaceae	Lellingeria oreophila (Maxon) A. R. Sm. & R. C. Moran	rps4-trnS	GU387080	FTOL v1.7
Polypodiaceae	Lellingeria oreophila (Maxon) A. R. Sm. & R. C. Moran	trnL-trnF	GU387243	FTOL v1.7
Polypodiaceae	Lellingeria paramicola Labiak	atpB	GU376601	FTOL v1.7
Polypodiaceae	Lellingeria paramicola Labiak	rbcL	GU387014	FTOL v1.7
Polypodiaceae	Lellingeria paramicola Labiak	rps4-trnS	GU387081	FTOL v1.7
Polypodiaceae	Lellingeria paramicola Labiak	trnL-trnF	GU387244	FTOL v1.7
Polypodiaceae	Lellingeria pendula (Sw.) A. R. Sm. & R. C. Moran	atpB	GU376602	FTOL v1.7
Polypodiaceae	Lellingeria pendula (Sw.) A. R. Sm. & R. C. Moran	rbcL	GU387036	FTOL v1.7
Polypodiaceae	Lellingeria pendula (Sw.) A. R. Sm. & R. C. Moran	rps4-trnS	GU387083	FTOL v1.7
Polypodiaceae	Lellingeria pendula (Sw.) A. R. Sm. & R. C. Moran	trnL-trnF	GU387246	FTOL v1.7
Polypodiaceae	Lellingeria phlegmaria (J. Sm.) A. R. Sm. & R. C. Morar	atpB	GU376603	FTOL v1.7
Polypodiaceae	Lellingeria phlegmaria (J. Sm.) A. R. Sm. & R. C. Morar	rbcL	GU387008	FTOL v1.7
Polypodiaceae	Lellingeria phlegmaria (J. Sm.) A. R. Sm. & R. C. Morar	rps4-trnS	GU387084	FTOL v1.7
Polypodiaceae	Lellingeria phlegmaria (J. Sm.) A. R. Sm. & R. C. Morar	trnL-trnF	GU387247	FTOL v1.7
Polypodiaceae	Lellingeria pseudocapillaris (Rosenst.) A. R. Sm. & R. C	atpB	GU376607	FTOL v1.7
Polypodiaceae	Lellingeria pseudocapillaris (Rosenst.) A. R. Sm. & R. C	rbcL	GU386983	FTOL v1.7
Polypodiaceae	Lellingeria pseudocapillaris (Rosenst.) A. R. Sm. & R. C	rps4-trnS	GU387088	FTOL v1.7
Polypodiaceae	Lellingeria pseudocapillaris (Rosenst.) A. R. Sm. & R. C	trnL-trnF	GU387251	FTOL v1.7
Polypodiaceae	Lellingeria randallii (Maxon) A. R. Sm. & R. C. Moran	atpB	GU376609	FTOL v1.7
Polypodiaceae	Lellingeria randallii (Maxon) A. R. Sm. & R. C. Moran	rbcL	GU387039	FTOL v1.7
Polypodiaceae	Lellingeria randallii (Maxon) A. R. Sm. & R. C. Moran	rps4-trnS	GU387091	FTOL v1.7
Polypodiaceae	Lellingeria randallii (Maxon) A. R. Sm. & R. C. Moran	trnL-trnF	GU387254	FTOL v1.7
Polypodiaceae	Lellingeria simacensis (Rosenst.) A. R. Sm. & R. C. Mor	atpB	GU376610	FTOL v1.7
Polypodiaceae	Lellingeria simacensis (Rosenst.) A. R. Sm. & R. C. Mor	rbcL	GU387001	FTOL v1.7
Polypodiaceae	Lellingeria simacensis (Rosenst.) A. R. Sm. & R. C. Mor	rps4-trnS	GU387092	FTOL v1.7
Polypodiaceae	Lellingeria simacensis (Rosenst.) A. R. Sm. & R. C. Mor	trnL-trnF	GU387255	FTOL v1.7
Polypodiaceae	Lellingeria subimpressa (Copel.) Labiak	atpB	GU376611	FTOL v1.7
Polypodiaceae	Lellingeria subimpressa (Copel.) Labiak	rbcL	GU386967	FTOL v1.7
Polypodiaceae	Lellingeria subimpressa (Copel.) Labiak	rps4-trnS	GU387093	FTOL v1.7
Polypodiaceae	Lellingeria subimpressa (Copel.) Labiak	trnL-trnF	GU387256	FTOL v1.7
Polypodiaceae	Lellingeria subsessilis (Baker) A. R. Sm. & R. C. Moran	atpB	GU376613	FTOL v1.7
Polypodiaceae	Lellingeria subsessilis (Baker) A. R. Sm. & R. C. Moran	rbcL	GU386997	FTOL v1.7
Polypodiaceae	Lellingeria subsessilis (Baker) A. R. Sm. & R. C. Moran	rps4-trnS	GU387095	FTOL v1.7
Polypodiaceae	Lellingeria subsessilis (Baker) A. R. Sm. & R. C. Moran	trnL-trnF	GU387258	FTOL v1.7
Polypodiaceae	Lellingeria suprasculpta (Christ) A. R. Sm. & R. C. Mora	atpB	GU376614	FTOL v1.7
Polypodiaceae	Lellingeria suprasculpta (Christ) A. R. Sm. & R. C. Mora	rbcL	GU386987	FTOL v1.7
Polypodiaceae	Lellingeria suprasculpta (Christ) A. R. Sm. & R. C. Mora	rps4-trnS	GU387096	FTOL v1.7
Polypodiaceae	Lellingeria suprasculpta (Christ) A. R. Sm. & R. C. Mora	trnL-trnF	GU387259	FTOL v1.7
Polypodiaceae	Lellingeria suspensa (L.) A. R. Sm. & R. C. Moran	atpB	GU376617	FTOL v1.7
Polypodiaceae	Lellingeria suspensa (L.) A. R. Sm. & R. C. Moran	rbcL	GU386976	FTOL v1.7
Polypodiaceae	Lellingeria suspensa (L.) A. R. Sm. & R. C. Moran	rps4-trnS	GU387099	FTOL v1.7
Polypodiaceae	Lellingeria suspensa (L.) A. R. Sm. & R. C. Moran	trnL-trnF	GU387262	FTOL v1.7
Polypodiaceae	Lellingeria tamandarei (Rosenst.) A. R. Sm. & R. C. Mo	atpB	GU376621	FTOL v1.7
Polypodiaceae	Lellingeria tamandarei (Rosenst.) A. R. Sm. & R. C. Mo	rbcL	GU387027	FTOL v1.7
Polypodiaceae	Lellingeria tamandarei (Rosenst.) A. R. Sm. & R. C. Mo	rps4-trnS	GU387103	FTOL v1.7
Polypodiaceae	Lellingeria tamandarei (Rosenst.) A. R. Sm. & R. C. Mo	trnL-trnF	GU387266	FTOL v1.7
Polypodiaceae	Lellingeria tenuicula (Fée) A. R. Sm. & R. C. Moran	atpB	GU376622	FTOL v1.7
Polypodiaceae	Lellingeria tenuicula (Fée) A. R. Sm. & R. C. Moran	rbcL	GU386977	FTOL v1.7
Polypodiaceae	Lellingeria tenuicula (Fée) A. R. Sm. & R. C. Moran	rps4-trnS	GU387104	FTOL v1.7
Polypodiaceae	Lellingeria tenuicula (Fée) A. R. Sm. & R. C. Moran	trnL-trnF	GU387267	FTOL v1.7
Polypodiaceae	Lellingeria tmesipteris (Copel.) A. R. Sm. & R. C. Morar	rbcL	MK319112	FTOL v1.7
Polypodiaceae	Lellingeria tmesipteris (Copel.) A. R. Sm. & R. C. Morar	trnL-trnF	MK319010	FTOL v1.7
Polypodiaceae	Lepisorus accedens (Blume) Hosok.	atpB	KX891382	FTOL v1.7
Polypodiaceae	Lepisorus accedens (Blume) Hosok.	rbcL	KX891368	FTOL v1.7
Polypodiaceae	Lepisorus accedens (Blume) Hosok.	trnL-trnF	KX891354	FTOL v1.7
Polypodiaceae	Lepisorus affinis Ching	plastome	MT130664	FTOL v1.7
Polypodiaceae	Lepisorus albertii (Regel) Ching	atpB	GQ256130	FTOL v1.7
Polypodiaceae	Lepisorus albertii (Regel) Ching	rbcL	GQ256292	FTOL v1.7
Polypodiaceae	Lepisorus albertii (Regel) Ching	rps4	GQ256366	FTOL v1.7
Polypodiaceae	Lepisorus albertii (Regel) Ching	rps4-trnS	GQ256366	FTOL v1.7
Polypodiaceae	Lepisorus albertii (Regel) Ching	trnL-trnF	GQ256217	FTOL v1.7
Polypodiaceae	Lepisorus angustus Ching	atpB	GQ256127	FTOL v1.7
Polypodiaceae	Lepisorus angustus Ching	rbcL	GQ256290	FTOL v1.7
Polypodiaceae	Lepisorus angustus Ching	rps4	GQ256364	FTOL v1.7
Polypodiaceae	Lepisorus angustus Ching	rps4-trnS	GQ256364	FTOL v1.7
Polypodiaceae	Lepisorus angustus Ching	trnL-trnF	GQ256214	FTOL v1.7
Polypodiaceae	Lepisorus annamensis (C. Chr.) Li Wang	atpB	GQ256079	FTOL v1.7
Polypodiaceae	Lepisorus annamensis (C. Chr.) Li Wang	rbcL	GQ256252	FTOL v1.7
Polypodiaceae	Lepisorus annamensis (C. Chr.) Li Wang	rps4	GQ256324	FTOL v1.7
Polypodiaceae	Lepisorus annamensis (C. Chr.) Li Wang	rps4-trnS	GQ256324	FTOL v1.7
Polypodiaceae	Lepisorus annamensis (C. Chr.) Li Wang	trnL-trnF	GQ256166	FTOL v1.7
Polypodiaceae	Lepisorus annuifrons (Makino) Ching	atpB	GQ256089	FTOL v1.7
Polypodiaceae	Lepisorus annuifrons (Makino) Ching	rbcL	GQ256258	FTOL v1.7
Polypodiaceae	Lepisorus annuifrons (Makino) Ching	rps4	GQ256331	FTOL v1.7
Polypodiaceae	Lepisorus annuifrons (Makino) Ching	rps4-trnS	GQ256331	FTOL v1.7
Polypodiaceae	Lepisorus annuifrons (Makino) Ching	trnL-trnF	GQ256176	FTOL v1.7
Polypodiaceae	Lepisorus asterolepis (Baker) Ching	atpB	GQ256090	FTOL v1.7
Polypodiaceae	Lepisorus asterolepis (Baker) Ching	rbcL	GQ256259	FTOL v1.7
Polypodiaceae	Lepisorus asterolepis (Baker) Ching	rps4	GQ256332	FTOL v1.7
Polypodiaceae	Lepisorus asterolepis (Baker) Ching	rps4-trnS	GQ256332	FTOL v1.7
Polypodiaceae	Lepisorus asterolepis (Baker) Ching	trnL-trnF	GQ256177	FTOL v1.7
Polypodiaceae	Lepisorus bicolor (Takeda) Ching	atpB	GQ256092	FTOL v1.7
Polypodiaceae	Lepisorus bicolor (Takeda) Ching	rbcL	GQ256261	FTOL v1.7
Polypodiaceae	Lepisorus bicolor (Takeda) Ching	rps4	GQ256334	FTOL v1.7
Polypodiaceae	Lepisorus bicolor (Takeda) Ching	rps4-trnS	GQ256334	FTOL v1.7
Polypodiaceae	Lepisorus bicolor (Takeda) Ching	trnL-trnF	GQ256179	FTOL v1.7
Polypodiaceae	Lepisorus boninensis Ching	atpB	GQ256093	FTOL v1.7
Polypodiaceae	Lepisorus boninensis Ching	rbcL	GQ256262	FTOL v1.7
Polypodiaceae	Lepisorus boninensis Ching	rps4	GQ256335	FTOL v1.7
Polypodiaceae	Lepisorus boninensis Ching	rps4-trnS	GQ256335	FTOL v1.7
Polypodiaceae	Lepisorus boninensis Ching	trnL-trnF	GQ256180	FTOL v1.7
Polypodiaceae	Lepisorus buergerianus (Miq.) C. F. Zhao, R. Wei & X. C	plastome	MN623364	FTOL v1.7
Polypodiaceae	Lepisorus carnosus (J. Sm.) C. F. Zhao, R. Wei & X. C. Zl	plastome	MT130559	FTOL v1.7
Polypodiaceae	Lepisorus cespitosus Y. X. Lin	atpB	LC524158	FTOL v1.7
Polypodiaceae	Lepisorus cespitosus Y. X. Lin	rbcL	LC524157	FTOL v1.7
Polypodiaceae	Lepisorus cespitosus Y. X. Lin	rps4	LC524159	FTOL v1.7
Polypodiaceae	Lepisorus cespitosus Y. X. Lin	rps4-trnS	LC524159	FTOL v1.7
Polypodiaceae	Lepisorus cespitosus Y. X. Lin	trnL-trnF	LC524160	FTOL v1.7
Polypodiaceae	Lepisorus clathratus (C. B. Clarke) Ching	plastome	KY419704	FTOL v1.7
Polypodiaceae	Lepisorus confluens W. M. Chu	plastome	MT130651	FTOL v1.7
Polypodiaceae	Lepisorus contortus (Christ) Ching	atpB	GQ256148	FTOL v1.7
Polypodiaceae	Lepisorus contortus (Christ) Ching	rbcL	GQ256308	FTOL v1.7
Polypodiaceae	Lepisorus contortus (Christ) Ching	rps4	GQ256384	FTOL v1.7
Polypodiaceae	Lepisorus contortus (Christ) Ching	rps4-trnS	GQ256384	FTOL v1.7
Polypodiaceae	Lepisorus contortus (Christ) Ching	trnL-trnF	GQ256235	FTOL v1.7
Polypodiaceae	Lepisorus crassipes Ching & Y. X. Lin	atpB	GQ256105	FTOL v1.7
Polypodiaceae	Lepisorus crassipes Ching & Y. X. Lin	rbcL	GQ256271	FTOL v1.7
Polypodiaceae	Lepisorus crassipes Ching & Y. X. Lin	rps4	GQ256345	FTOL v1.7
Polypodiaceae	Lepisorus crassipes Ching & Y. X. Lin	rps4-trnS	GQ256345	FTOL v1.7
Polypodiaceae	Lepisorus crassipes Ching & Y. X. Lin	trnL-trnF	GQ256192	FTOL v1.7
Polypodiaceae	Lepisorus distans (Makino) Ching	atpB	GQ256152	FTOL v1.7
Polypodiaceae	Lepisorus distans (Makino) Ching	rbcL	GQ256312	FTOL v1.7
Polypodiaceae	Lepisorus distans (Makino) Ching	rps4	GQ256388	FTOL v1.7
Polypodiaceae	Lepisorus distans (Makino) Ching	rps4-trnS	GQ256388	FTOL v1.7
Polypodiaceae	Lepisorus distans (Makino) Ching	trnL-trnF	GQ256239	FTOL v1.7
Polypodiaceae	Lepisorus diversus (Rosenst.) comb. ined.	plastome	MT968973	FTOL v1.7
Polypodiaceae	Lepisorus drymoglossoides (Baker) comb. ined.	plastome	OP369280	FTOL v1.7
Polypodiaceae	Lepisorus elegans Ching & W. M. Chu	atpB	GQ256100	FTOL v1.7
Polypodiaceae	Lepisorus elegans Ching & W. M. Chu	rbcL	GQ256268	FTOL v1.7
Polypodiaceae	Lepisorus elegans Ching & W. M. Chu	rps4	GQ256342	FTOL v1.7
Polypodiaceae	Lepisorus elegans Ching & W. M. Chu	rps4-trnS	GQ256342	FTOL v1.7
Polypodiaceae	Lepisorus elegans Ching & W. M. Chu	trnL-trnF	GQ256187	FTOL v1.7
Polypodiaceae	Lepisorus ensatus (Thunb.) C. F. Zhao, R. Wei & X. C. Zl	atpA	MH113549	FTOL v1.7
Polypodiaceae	Lepisorus ensatus (Thunb.) C. F. Zhao, R. Wei & X. C. Zl	atpB	MH113583	FTOL v1.7
Polypodiaceae	Lepisorus ensatus (Thunb.) C. F. Zhao, R. Wei & X. C. Zl	rbcL	MH051184	FTOL v1.7
Polypodiaceae	Lepisorus ensatus (Thunb.) C. F. Zhao, R. Wei & X. C. Zl	rps4	MH113483	FTOL v1.7
Polypodiaceae	Lepisorus ensatus (Thunb.) C. F. Zhao, R. Wei & X. C. Zl	rps4-trnS	MH113483	FTOL v1.7
Polypodiaceae	Lepisorus ensatus (Thunb.) C. F. Zhao, R. Wei & X. C. Zl	trnL-trnF	MH113516	FTOL v1.7
Polypodiaceae	Lepisorus excavatus (Bory ex Willd.) Ching	atpB	GQ256091	FTOL v1.7
Polypodiaceae	Lepisorus excavatus (Bory ex Willd.) Ching	rbcL	GQ256260	FTOL v1.7
Polypodiaceae	Lepisorus excavatus (Bory ex Willd.) Ching	rps4	GQ256333	FTOL v1.7
Polypodiaceae	Lepisorus excavatus (Bory ex Willd.) Ching	rps4-trnS	GQ256333	FTOL v1.7
Polypodiaceae	Lepisorus excavatus (Bory ex Willd.) Ching	trnL-trnF	GQ256178	FTOL v1.7
Polypodiaceae	Lepisorus fortunei (T. Moore) C. M. Kuo	plastome	MT373087	FTOL v1.7
Polypodiaceae	Lepisorus hachijoensis Sa. Kurata	atpB	GQ256103	FTOL v1.7
Polypodiaceae	Lepisorus hachijoensis Sa. Kurata	rbcL	GQ256269	FTOL v1.7
Polypodiaceae	Lepisorus hachijoensis Sa. Kurata	rps4	GQ256343	FTOL v1.7
Polypodiaceae	Lepisorus hachijoensis Sa. Kurata	rps4-trnS	GQ256343	FTOL v1.7
Polypodiaceae	Lepisorus hachijoensis Sa. Kurata	trnL-trnF	GQ256190	FTOL v1.7
Polypodiaceae	Lepisorus henryi (Hieron. ex C. Chr.) Li Wang	atpB	GQ256080	FTOL v1.7
Polypodiaceae	Lepisorus henryi (Hieron. ex C. Chr.) Li Wang	rbcL	GQ256253	FTOL v1.7
Polypodiaceae	Lepisorus henryi (Hieron. ex C. Chr.) Li Wang	rps4	GQ256325	FTOL v1.7
Polypodiaceae	Lepisorus henryi (Hieron. ex C. Chr.) Li Wang	rps4-trnS	GQ256325	FTOL v1.7
Polypodiaceae	Lepisorus henryi (Hieron. ex C. Chr.) Li Wang	trnL-trnF	GQ256167	FTOL v1.7
Polypodiaceae	Lepisorus heterolepis (Rosenst.) Ching	atpB	GQ256104	FTOL v1.7
Polypodiaceae	Lepisorus heterolepis (Rosenst.) Ching	rbcL	GQ256270	FTOL v1.7
Polypodiaceae	Lepisorus heterolepis (Rosenst.) Ching	rps4	GQ256344	FTOL v1.7
Polypodiaceae	Lepisorus heterolepis (Rosenst.) Ching	rps4-trnS	GQ256344	FTOL v1.7
Polypodiaceae	Lepisorus heterolepis (Rosenst.) Ching	trnL-trnF	GQ256191	FTOL v1.7
Polypodiaceae	Lepisorus jakonensis (Blanf.) Fraser-Jenk. & J. Krieg.	plastome	MW876326	FTOL v1.7

Polypodiaceae	Lepisorus kawakamii (Hayata) Tagawa	atpB	GQ256106	FTOL v1.7
Polypodiaceae	Lepisorus kawakamii (Hayata) Tagawa	rbcL	EU482940	FTOL v1.7
Polypodiaceae	Lepisorus kawakamii (Hayata) Tagawa	trnL-trnF	GQ256193	FTOL v1.7
Polypodiaceae	Lepisorus kuchenensis (Y. C. Wu) Ching	atpB	GQ256107	FTOL v1.7
Polypodiaceae	Lepisorus kuchenensis (Y. C. Wu) Ching	rbcL	GQ256272	FTOL v1.7
Polypodiaceae	Lepisorus kuchenensis (Y. C. Wu) Ching	rps4	GQ256346	FTOL v1.7
Polypodiaceae	Lepisorus kuchenensis (Y. C. Wu) Ching	rps4-trnS	GQ256346	FTOL v1.7
Polypodiaceae	Lepisorus kuchenensis (Y. C. Wu) Ching	trnL-trnF	GQ256194	FTOL v1.7
Polypodiaceae	Lepisorus kuratae T. Fujiw. & Seriz.	rbcL	LC331937	FTOL v1.7
Polypodiaceae	Lepisorus kuratae T. Fujiw. & Seriz.	rps4	LC332106	FTOL v1.7
Polypodiaceae	Lepisorus kuratae T. Fujiw. & Seriz.	rps4-trnS	LC332106	FTOL v1.7
Polypodiaceae	Lepisorus lewisii (Baker) Ching	atpB	GQ256108	FTOL v1.7
Polypodiaceae	Lepisorus lewisii (Baker) Ching	rbcL	GQ256273	FTOL v1.7
Polypodiaceae	Lepisorus lewisii (Baker) Ching	rps4	GQ256347	FTOL v1.7
Polypodiaceae	Lepisorus lewisii (Baker) Ching	rps4-trnS	GQ256347	FTOL v1.7
Polypodiaceae	Lepisorus lewisii (Baker) Ching	trnL-trnF	GQ256195	FTOL v1.7
Polypodiaceae	Lepisorus likiangensis Ching & S. K. Wu	atpB	GQ256099	FTOL v1.7
Polypodiaceae	Lepisorus likiangensis Ching & S. K. Wu	rbcL	GQ256267	FTOL v1.7
Polypodiaceae	Lepisorus likiangensis Ching & S. K. Wu	rps4	GQ256341	FTOL v1.7
Polypodiaceae	Lepisorus likiangensis Ching & S. K. Wu	rps4-trnS	GQ256341	FTOL v1.7
Polypodiaceae	Lepisorus likiangensis Ching & S. K. Wu	trnL-trnF	GQ256186	FTOL v1.7
Polypodiaceae	Lepisorus lineariformis Ching & S. K. Wu	atpB	GQ256112	FTOL v1.7
Polypodiaceae	Lepisorus lineariformis Ching & S. K. Wu	rbcL	GQ256277	FTOL v1.7
Polypodiaceae	Lepisorus lineariformis Ching & S. K. Wu	rps4	GQ256351	FTOL v1.7
Polypodiaceae	Lepisorus lineariformis Ching & S. K. Wu	rps4-trnS	GQ256351	FTOL v1.7
Polypodiaceae	Lepisorus lineariformis Ching & S. K. Wu	trnL-trnF	GQ256199	FTOL v1.7
Polypodiaceae	Lepisorus longifolius (Blume) Holttum	plastome	MW876327	FTOL v1.7
Polypodiaceae	Lepisorus loriformis (Wall. ex Mett.) Ching	atpB	GQ256153	FTOL v1.7
Polypodiaceae	Lepisorus loriformis (Wall. ex Mett.) Ching	rbcL	GQ256313	FTOL v1.7
Polypodiaceae	Lepisorus loriformis (Wall. ex Mett.) Ching	rps4	GQ256389	FTOL v1.7
Polypodiaceae	Lepisorus loriformis (Wall. ex Mett.) Ching	rps4-trnS	GQ256389	FTOL v1.7
Polypodiaceae	Lepisorus loriformis (Wall. ex Mett.) Ching	trnL-trnF	GQ256240	FTOL v1.7
Polypodiaceae	Lepisorus luchunensis Y. X. Lin	atpB	HQ712007	FTOL v1.7
Polypodiaceae	Lepisorus luchunensis Y. X. Lin	rbcL	HQ712000	FTOL v1.7
Polypodiaceae	Lepisorus luchunensis Y. X. Lin	rps4	HQ712008	FTOL v1.7
Polypodiaceae	Lepisorus luchunensis Y. X. Lin	rps4-trnS	HQ712008	FTOL v1.7
Polypodiaceae	Lepisorus luchunensis Y. X. Lin	trnL-trnF	HQ712019	FTOL v1.7
Polypodiaceae	Lepisorus macrosphaerus (Baker) Ching	atpB	GQ256116	FTOL v1.7
Polypodiaceae	Lepisorus macrosphaerus (Baker) Ching	rbcL	GQ256280	FTOL v1.7
Polypodiaceae	Lepisorus macrosphaerus (Baker) Ching	rps4	GQ256354	FTOL v1.7
Polypodiaceae	Lepisorus macrosphaerus (Baker) Ching	rps4-trnS	GQ256354	FTOL v1.7
Polypodiaceae	Lepisorus macrosphaerus (Baker) Ching	trnL-trnF	GQ256203	FTOL v1.7
Polypodiaceae	Lepisorus maculosus (Christ) C. F. Zhao, R. Wei & X. C.	atpB	GQ256165	FTOL v1.7
Polypodiaceae	Lepisorus maculosus (Christ) C. F. Zhao, R. Wei & X. C.	rbcL	GQ256323	FTOL v1.7
Polypodiaceae	Lepisorus maculosus (Christ) C. F. Zhao, R. Wei & X. C.	rps4	GQ256401	FTOL v1.7
Polypodiaceae	Lepisorus maculosus (Christ) C. F. Zhao, R. Wei & X. C.	rps4-trnS	GQ256401	FTOL v1.7
Polypodiaceae	Lepisorus maculosus (Christ) C. F. Zhao, R. Wei & X. C.	trnL-trnF	GQ256251	FTOL v1.7
Polypodiaceae	Lepisorus marginatus Ching	atpB	GQ256117	FTOL v1.7
Polypodiaceae	Lepisorus marginatus Ching	rbcL	GQ256281	FTOL v1.7
Polypodiaceae	Lepisorus marginatus Ching	rps4	GQ256355	FTOL v1.7
Polypodiaceae	Lepisorus marginatus Ching	rps4-trnS	GQ256355	FTOL v1.7
Polypodiaceae	Lepisorus marginatus Ching	trnL-trnF	GQ256204	FTOL v1.7
Polypodiaceae	Lepisorus medioximus T.Fujiw., K.Hori & Khine	atpB	LC710541	FTOL v1.7
Polypodiaceae	Lepisorus medioximus T.Fujiw., K.Hori & Khine	rbcL	LC710540	FTOL v1.7
Polypodiaceae	Lepisorus medioximus T.Fujiw., K.Hori & Khine	rps4	LC710544	FTOL v1.7
Polypodiaceae	Lepisorus medioximus T.Fujiw., K.Hori & Khine	rps4-trnS	LC710544	FTOL v1.7
Polypodiaceae	Lepisorus medioximus T.Fujiw., K.Hori & Khine	trnL-trnF	LC710546	FTOL v1.7
Polypodiaceae	Lepisorus medogensis Ching & Y. X. Lin	atpB	GQ256118	FTOL v1.7
Polypodiaceae	Lepisorus medogensis Ching & Y. X. Lin	rbcL	GQ256282	FTOL v1.7
Polypodiaceae	Lepisorus medogensis Ching & Y. X. Lin	rps4	GQ256356	FTOL v1.7
Polypodiaceae	Lepisorus medogensis Ching & Y. X. Lin	rps4-trnS	GQ256356	FTOL v1.7
Polypodiaceae	Lepisorus medogensis Ching & Y. X. Lin	trnL-trnF	GQ256205	FTOL v1.7
Polypodiaceae	Lepisorus megasorus (C. Chr.) Ching	rbcL	DQ642158	FTOL v1.7
Polypodiaceae	Lepisorus megasorus (C. Chr.) Ching	rps4	DQ642196	FTOL v1.7
Polypodiaceae	Lepisorus megasorus (C. Chr.) Ching	rps4-trnS	DQ642196	FTOL v1.7
Polypodiaceae	Lepisorus megasorus (C. Chr.) Ching	trnL-trnF	DQ642240	FTOL v1.7
Polypodiaceae	Lepisorus microphyllus (C. Presl) comb. ined.	plastome	MN623356	FTOL v1.7
Polypodiaceae	Lepisorus mikawanus Kurata	rbcL	LC331943	FTOL v1.7
Polypodiaceae	Lepisorus mikawanus Kurata	rps4	LC332112	FTOL v1.7
Polypodiaceae	Lepisorus mikawanus Kurata	rps4-trnS	LC332112	FTOL v1.7
Polypodiaceae	Lepisorus miyoshianus (Makino) Fraser-Jenk. & Subh. C	plastome	MW876328	FTOL v1.7
Polypodiaceae	Lepisorus monilisorus (Hayata) Tagawa	atpB	GQ256120	FTOL v1.7
Polypodiaceae	Lepisorus monilisorus (Hayata) Tagawa	rbcL	GQ256283	FTOL v1.7
Polypodiaceae	Lepisorus monilisorus (Hayata) Tagawa	rps4	GQ256357	FTOL v1.7
Polypodiaceae	Lepisorus monilisorus (Hayata) Tagawa	rps4-trnS	GQ256357	FTOL v1.7
Polypodiaceae	Lepisorus monilisorus (Hayata) Tagawa	trnL-trnF	GQ256207	FTOL v1.7
Polypodiaceae	Lepisorus morrisonensis (Hayata) H. Itô	atpB	GQ256122	FTOL v1.7
Polypodiaceae	Lepisorus morrisonensis (Hayata) H. Itô	rbcL	GQ256285	FTOL v1.7
Polypodiaceae	Lepisorus morrisonensis (Hayata) H. Itô	rps4	GQ256359	FTOL v1.7
Polypodiaceae	Lepisorus morrisonensis (Hayata) H. Itô	rps4-trnS	GQ256359	FTOL v1.7
Polypodiaceae	Lepisorus morrisonensis (Hayata) H. Itô	trnL-trnF	GQ256209	FTOL v1.7
Polypodiaceae	Lepisorus mucronatus (Fée) Li Wang	atpB	MT169854	FTOL v1.7
Polypodiaceae	Lepisorus mucronatus (Fée) Li Wang	rbcL	MT169814	FTOL v1.7
Polypodiaceae	Lepisorus mucronatus (Fée) Li Wang	rps4	MT169884	FTOL v1.7
Polypodiaceae	Lepisorus mucronatus (Fée) Li Wang	rps4-trnS	MT169884	FTOL v1.7
Polypodiaceae	Lepisorus mucronatus (Fée) Li Wang	trnL-trnF	MT169922	FTOL v1.7
Polypodiaceae	Lepisorus nigripes T. Fujiw. & Seriz.	rbcL	LC331927	FTOL v1.7
Polypodiaceae	Lepisorus nigripes T. Fujiw. & Seriz.	rps4	LC332095	FTOL v1.7
Polypodiaceae	Lepisorus nigripes T. Fujiw. & Seriz.	rps4-trnS	LC332095	FTOL v1.7
Polypodiaceae	Lepisorus normalis (D. Don) C. F. Zhao, R. Wei & X. C. Z	atpB	MT169864	FTOL v1.7
Polypodiaceae	Lepisorus normalis (D. Don) C. F. Zhao, R. Wei & X. C. Z	rbcL	MT169824	FTOL v1.7
Polypodiaceae	Lepisorus normalis (D. Don) C. F. Zhao, R. Wei & X. C. Z	rps4	MT169894	FTOL v1.7
Polypodiaceae	Lepisorus normalis (D. Don) C. F. Zhao, R. Wei & X. C. Z	rps4-trnS	MT169894	FTOL v1.7
Polypodiaceae	Lepisorus normalis (D. Don) C. F. Zhao, R. Wei & X. C. Z	trnL-trnF	MT169932	FTOL v1.7
Polypodiaceae	Lepisorus nudus (Hook.) Ching	atpB	MT169858	FTOL v1.7
Polypodiaceae	Lepisorus nudus (Hook.) Ching	rbcL	MT169818	FTOL v1.7
Polypodiaceae	Lepisorus nudus (Hook.) Ching	rps4	MT169888	FTOL v1.7
Polypodiaceae	Lepisorus nudus (Hook.) Ching	rps4-trnS	MT169888	FTOL v1.7
Polypodiaceae	Lepisorus nudus (Hook.) Ching	trnL-trnF	MT169926	FTOL v1.7
Polypodiaceae	Lepisorus obscurevenulosus (Hayata) Ching	atpB	GQ256123	FTOL v1.7
Polypodiaceae	Lepisorus obscurevenulosus (Hayata) Ching	rbcL	GQ256286	FTOL v1.7
Polypodiaceae	Lepisorus obscurevenulosus (Hayata) Ching	rps4	GQ256360	FTOL v1.7
Polypodiaceae	Lepisorus obscurevenulosus (Hayata) Ching	rps4-trnS	GQ256360	FTOL v1.7
Polypodiaceae	Lepisorus obscurevenulosus (Hayata) Ching	trnL-trnF	GQ256210	FTOL v1.7
Polypodiaceae	Lepisorus oligolepidus (Baker) Ching	atpB	GQ256124	FTOL v1.7
Polypodiaceae	Lepisorus oligolepidus (Baker) Ching	rbcL	GQ256287	FTOL v1.7
Polypodiaceae	Lepisorus oligolepidus (Baker) Ching	rps4	GQ256361	FTOL v1.7
Polypodiaceae	Lepisorus oligolepidus (Baker) Ching	rps4-trnS	GQ256361	FTOL v1.7
Polypodiaceae	Lepisorus oligolepidus (Baker) Ching	trnL-trnF	GQ256211	FTOL v1.7
Polypodiaceae	Lepisorus onoei (Franch. & Sav.) Ching	atpB	GQ256125	FTOL v1.7
Polypodiaceae	Lepisorus onoei (Franch. & Sav.) Ching	rbcL	GQ256288	FTOL v1.7
Polypodiaceae	Lepisorus onoei (Franch. & Sav.) Ching	rps4	GQ256362	FTOL v1.7
Polypodiaceae	Lepisorus onoei (Franch. & Sav.) Ching	rps4-trnS	GQ256362	FTOL v1.7
Polypodiaceae	Lepisorus onoei (Franch. & Sav.) Ching	trnL-trnF	GQ256212	FTOL v1.7
Polypodiaceae	Lepisorus ovatus (C. Presl) C. F. Zhao, R. Wei & X. C. Zh	plastome	MT130595	FTOL v1.7
Polypodiaceae	Lepisorus palmatopedatus (Baker) C. F. Zhao, R. Wei &	plastome	MH707375	FTOL v1.7
Polypodiaceae	Lepisorus perrierianus (C. Chr.) Ching	atpB	HQ712003	FTOL v1.7
Polypodiaceae	Lepisorus perrierianus (C. Chr.) Ching	rbcL	HQ711995	FTOL v1.7
Polypodiaceae	Lepisorus perrierianus (C. Chr.) Ching	trnL-trnF	HQ712017	FTOL v1.7
Polypodiaceae	Lepisorus platyrhynchos (J. Sm. ex Kunze) Li Wang	rbcL	DQ642152	FTOL v1.7
Polypodiaceae	Lepisorus platyrhynchos (J. Sm. ex Kunze) Li Wang	rps4	DQ642190	FTOL v1.7
Polypodiaceae	Lepisorus platyrhynchos (J. Sm. ex Kunze) Li Wang	rps4-trnS	DQ642190	FTOL v1.7
Polypodiaceae	Lepisorus platyrhynchos (J. Sm. ex Kunze) Li Wang	trnL-trnF	DQ642233	FTOL v1.7
Polypodiaceae	Lepisorus pseudonudus Ching	atpB	GQ256128	FTOL v1.7
Polypodiaceae	Lepisorus pseudonudus Ching	rbcL	GQ256291	FTOL v1.7
Polypodiaceae	Lepisorus pseudonudus Ching	rps4	GQ256365	FTOL v1.7
Polypodiaceae	Lepisorus pseudonudus Ching	rps4-trnS	GQ256365	FTOL v1.7
Polypodiaceae	Lepisorus pseudonudus Ching	trnL-trnF	GQ256215	FTOL v1.7
Polypodiaceae	Lepisorus pseudoussuriensis Tagawa	rbcL	EU482943	FTOL v1.7
Polypodiaceae	Lepisorus pseudoussuriensis Tagawa	trnL-trnF	EU483038	FTOL v1.7
Polypodiaceae	Lepisorus pyriformis (Ching) C. F. Zhao, R. Wei & X. C. Z	atpB	GU126703	FTOL v1.7
Polypodiaceae	Lepisorus pyriformis (Ching) C. F. Zhao, R. Wei & X. C. Z	rbcL	GU126695	FTOL v1.7
Polypodiaceae	Lepisorus pyriformis (Ching) C. F. Zhao, R. Wei & X. C. Z	rps4	GU126714	FTOL v1.7
Polypodiaceae	Lepisorus pyriformis (Ching) C. F. Zhao, R. Wei & X. C. Z	rps4-trnS	GU126714	FTOL v1.7
Polypodiaceae	Lepisorus pyriformis (Ching) C. F. Zhao, R. Wei & X. C. Z	trnL-trnF	GU126725	FTOL v1.7
Polypodiaceae	Lepisorus rostratus (Bedd.) C. F. Zhao, R. Wei & X. C. Zh	atpB	GU126705	FTOL v1.7
Polypodiaceae	Lepisorus rostratus (Bedd.) C. F. Zhao, R. Wei & X. C. Zh	rbcL	GU126697	FTOL v1.7
Polypodiaceae	Lepisorus rostratus (Bedd.) C. F. Zhao, R. Wei & X. C. Zh	rps4	GU126716	FTOL v1.7
Polypodiaceae	Lepisorus rostratus (Bedd.) C. F. Zhao, R. Wei & X. C. Zh	rps4-trnS	GU126716	FTOL v1.7
Polypodiaceae	Lepisorus rostratus (Bedd.) C. F. Zhao, R. Wei & X. C. Zh	trnL-trnF	GU126727	FTOL v1.7
Polypodiaceae	Lepisorus rufofuscus T.Fujiwara	atpB	LC732056	FTOL v1.7
Polypodiaceae	Lepisorus rufofuscus T.Fujiwara	rbcL	LC732052	FTOL v1.7
Polypodiaceae	Lepisorus rufofuscus T.Fujiwara	rps4	LC732061	FTOL v1.7
Polypodiaceae	Lepisorus rufofuscus T.Fujiwara	rps4-trnS	LC732061	FTOL v1.7
Polypodiaceae	Lepisorus rufofuscus T.Fujiwara	trnL-trnF	LC732064	FTOL v1.7
Polypodiaceae	Lepisorus schraderi (Mett.) Ching	plastome	MW876329	FTOL v1.7
Polypodiaceae	Lepisorus scolopendrium (Ching) Mehra & Bir	atpB	GQ256132	FTOL v1.7
Polypodiaceae	Lepisorus scolopendrium (Ching) Mehra & Bir	rbcL	GQ256294	FTOL v1.7
Polypodiaceae	Lepisorus scolopendrium (Ching) Mehra & Bir	rps4	GQ256368	FTOL v1.7
Polypodiaceae	Lepisorus scolopendrium (Ching) Mehra & Bir	rps4-trnS	GQ256368	FTOL v1.7
Polypodiaceae	Lepisorus scolopendrium (Ching) Mehra & Bir	trnL-trnF	GQ256219	FTOL v1.7
Polypodiaceae	Lepisorus sinensis (Christ) Ching	atpB	GQ256134	FTOL v1.7
Polypodiaceae	Lepisorus sinensis (Christ) Ching	rbcL	MT169819	FTOL v1.7
Polypodiaceae	Lepisorus sinensis (Christ) Ching	rps4	MT169889	FTOL v1.7
Polypodiaceae	Lepisorus sinensis (Christ) Ching	rps4-trnS	GQ256370	FTOL v1.7
Polypodiaceae	Lepisorus sinensis (Christ) Ching	trnL-trnF	MT169927	FTOL v1.7
Polypodiaceae	Lepisorus sordidus (C. Chr.) Ching	atpB	GQ256136	FTOL v1.7
Polypodiaceae	Lepisorus sordidus (C. Chr.) Ching	rbcL	GQ256298	FTOL v1.7
Polypodiaceae	Lepisorus sordidus (C. Chr.) Ching	rps4	GQ256372	FTOL v1.7
Polypodiaceae	Lepisorus sordidus (C. Chr.) Ching	rps4-trnS	GQ256372	FTOL v1.7
Polypodiaceae	Lepisorus sordidus (C. Chr.) Ching	trnL-trnF	GQ256223	FTOL v1.7
Polypodiaceae	Lepisorus soulieanus (Christ) Ching & S. K. Wu	atpB	GQ256163	FTOL v1.7
Polypodiaceae	Lepisorus soulieanus (Christ) Ching & S. K. Wu	rbcL	GQ256321	FTOL v1.7
Polypodiaceae	Lepisorus soulieanus (Christ) Ching & S. K. Wu	rps4	GQ256399	FTOL v1.7
Polypodiaceae	Lepisorus soulieanus (Christ) Ching & S. K. Wu	rps4-trnS	GQ256399	FTOL v1.7
Polypodiaceae	Lepisorus soulieanus (Christ) Ching & S. K. Wu	trnL-trnF	GQ256249	FTOL v1.7
Polypodiaceae	Lepisorus spicatus (L. fil.) Li Wang	plastome	MW876330	FTOL v1.7
Polypodiaceae	Lepisorus squamatus (A. R. Sm. & X. C. Zhang) C. F. Zh	atpB	KX891392	FTOL v1.7
Polypodiaceae	Lepisorus squamatus (A. R. Sm. & X. C. Zhang) C. F. Zh	rbcL	GU126692	FTOL v1.7
Polypodiaceae	Lepisorus squamatus (A. R. Sm. & X. C. Zhang) C. F. Zh	rps4	GU126710	FTOL v1.7
Polypodiaceae	Lepisorus squamatus (A. R. Sm. & X. C. Zhang) C. F. Zh	rps4-trnS	GU126710	FTOL v1.7
Polypodiaceae	Lepisorus squamatus (A. R. Sm. & X. C. Zhang) C. F. Zh	trnL-trnF	GU126721	FTOL v1.7
Polypodiaceae	Lepisorus subconfluens Ching	atpB	GQ256137	FTOL v1.7
Polypodiaceae	Lepisorus subconfluens Ching	rbcL	GQ256299	FTOL v1.7
Polypodiaceae	Lepisorus subconfluens Ching	rps4	GQ256373	FTOL v1.7
Polypodiaceae	Lepisorus subconfluens Ching	rps4-trnS	GQ256373	FTOL v1.7
Polypodiaceae	Lepisorus subconfluens Ching	trnL-trnF	GQ256224	FTOL v1.7
Polypodiaceae	Lepisorus subhemionitideus (Christ) C. F. Zhao, R. Wei	atpB	GU126701	FTOL v1.7
Polypodiaceae	Lepisorus subhemionitideus (Christ) C. F. Zhao, R. Wei	rbcL	GU126693	FTOL v1.7
Polypodiaceae	Lepisorus subhemionitideus (Christ) C. F. Zhao, R. Wei	rps4	GU126712	FTOL v1.7
Polypodiaceae	Lepisorus subhemionitideus (Christ) C. F. Zhao, R. Wei	rps4-trnS	GU126712	FTOL v1.7
Polypodiaceae	Lepisorus subhemionitideus (Christ) C. F. Zhao, R. Wei	trnL-trnF	GU126723	FTOL v1.7
Polypodiaceae	Lepisorus sublinearis (Baker ex Takeda) Ching	atpB	GQ256139	FTOL v1.7
Polypodiaceae	Lepisorus sublinearis (Baker ex Takeda) Ching	rbcL	GQ256301	FTOL v1.7
Polypodiaceae	Lepisorus sublinearis (Baker ex Takeda) Ching	rps4	GQ256375	FTOL v1.7
Polypodiaceae	Lepisorus sublinearis (Baker ex Takeda) Ching	rps4-trnS	GQ256375	FTOL v1.7
Polypodiaceae	Lepisorus sublinearis (Baker ex Takeda) Ching	trnL-trnF	GQ256226	FTOL v1.7

Polypodiaceae	Lepisorus suboligolepidus Ching	plastome	MT130652	FTOL v1.7
Polypodiaceae	Lepisorus subsessilis Ching & Y. X. Lin	atpB	GQ256141	FTOL v1.7
Polypodiaceae	Lepisorus subsessilis Ching & Y. X. Lin	rps4	GQ256377	FTOL v1.7
Polypodiaceae	Lepisorus subsessilis Ching & Y. X. Lin	rps4-trnS	GQ256377	FTOL v1.7
Polypodiaceae	Lepisorus subsessilis Ching & Y. X. Lin	trnL-trnF	GQ256228	FTOL v1.7
Polypodiaceae	Lepisorus superficialis (Blume) C. F. Zhao, R. Wei & X.	plastome	MT130546	FTOL v1.7
Polypodiaceae	Lepisorus thaipaiensis Ching & S. K. Wu	atpB	GQ256142	FTOL v1.7
Polypodiaceae	Lepisorus thaipaiensis Ching & S. K. Wu	rbcL	GQ256302	FTOL v1.7
Polypodiaceae	Lepisorus thaipaiensis Ching & S. K. Wu	rps4	GQ256378	FTOL v1.7
Polypodiaceae	Lepisorus thaipaiensis Ching & S. K. Wu	rps4-trnS	GQ256378	FTOL v1.7
Polypodiaceae	Lepisorus thaipaiensis Ching & S. K. Wu	trnL-trnF	GQ256229	FTOL v1.7
Polypodiaceae	Lepisorus thunbergianus (Kaulf.) Ching	atpA	MT137061	FTOL v1.7
Polypodiaceae	Lepisorus thunbergianus (Kaulf.) Ching	atpB	MT137062	FTOL v1.7
Polypodiaceae	Lepisorus thunbergianus (Kaulf.) Ching	rbcL	MT137054	FTOL v1.7
Polypodiaceae	Lepisorus thunbergianus (Kaulf.) Ching	rps4	MT137057	FTOL v1.7
Polypodiaceae	Lepisorus thunbergianus (Kaulf.) Ching	rps4-trnS	MT137057	FTOL v1.7
Polypodiaceae	Lepisorus thunbergianus (Kaulf.) Ching	trnL-trnF	MT137059	FTOL v1.7
Polypodiaceae	Lepisorus tibeticus Ching & S. K. Wu	atpB	GQ256147	FTOL v1.7
Polypodiaceae	Lepisorus tibeticus Ching & S. K. Wu	rbcL	GQ256307	FTOL v1.7
Polypodiaceae	Lepisorus tibeticus Ching & S. K. Wu	rps4	GQ256383	FTOL v1.7
Polypodiaceae	Lepisorus tibeticus Ching & S. K. Wu	rps4-trnS	GQ256383	FTOL v1.7
Polypodiaceae	Lepisorus tibeticus Ching & S. K. Wu	trnL-trnF	GQ256234	FTOL v1.7
Polypodiaceae	Lepisorus tosaensis (Makino) H. Itô	atpB	GQ256149	FTOL v1.7
Polypodiaceae	Lepisorus tosaensis (Makino) H. Itô	rbcL	GQ256309	FTOL v1.7
Polypodiaceae	Lepisorus tosaensis (Makino) H. Itô	rps4	GQ256385	FTOL v1.7
Polypodiaceae	Lepisorus tosaensis (Makino) H. Itô	rps4-trnS	GQ256385	FTOL v1.7
Polypodiaceae	Lepisorus tosaensis (Makino) H. Itô	trnL-trnF	GQ256236	FTOL v1.7
Polypodiaceae	Lepisorus triglossus (Baker) C. F. Zhao, R. Wei & X. C. Z	atpB	HQ597024	FTOL v1.7
Polypodiaceae	Lepisorus triglossus (Baker) C. F. Zhao, R. Wei & X. C. Z	rbcL	HQ597010	FTOL v1.7
Polypodiaceae	Lepisorus triglossus (Baker) C. F. Zhao, R. Wei & X. C. Z	trnL-trnF	HQ597019	FTOL v1.7
Polypodiaceae	Lepisorus uchiyamae (Makino) H. Itô	atpB	GQ256150	FTOL v1.7
Polypodiaceae	Lepisorus uchiyamae (Makino) H. Itô	rbcL	GQ256310	FTOL v1.7
Polypodiaceae	Lepisorus uchiyamae (Makino) H. Itô	rps4	GQ256386	FTOL v1.7
Polypodiaceae	Lepisorus uchiyamae (Makino) H. Itô	rps4-trnS	GQ256386	FTOL v1.7
Polypodiaceae	Lepisorus uchiyamae (Makino) H. Itô	trnL-trnF	GQ256237	FTOL v1.7
Polypodiaceae	Lepisorus ussuriensis (Regel & Mack.) Ching	atpB	GQ256151	FTOL v1.7
Polypodiaceae	Lepisorus ussuriensis (Regel & Mack.) Ching	rbcL	GQ256311	FTOL v1.7
Polypodiaceae	Lepisorus ussuriensis (Regel & Mack.) Ching	rps4	GQ256387	FTOL v1.7
Polypodiaceae	Lepisorus ussuriensis (Regel & Mack.) Ching	rps4-trnS	GQ256387	FTOL v1.7
Polypodiaceae	Lepisorus ussuriensis (Regel & Mack.) Ching	trnL-trnF	GQ256238	FTOL v1.7
Polypodiaceae	Lepisorus validinervis (Kunze) Li Wang	atpA	MH664989	FTOL v1.7
Polypodiaceae	Lepisorus validinervis (Kunze) Li Wang	atpB	MH665005	FTOL v1.7
Polypodiaceae	Lepisorus validinervis (Kunze) Li Wang	rbcL	MH665031	FTOL v1.7
Polypodiaceae	Lepisorus validinervis (Kunze) Li Wang	rps4	MH665093	FTOL v1.7
Polypodiaceae	Lepisorus validinervis (Kunze) Li Wang	rps4-trnS	MH665093	FTOL v1.7
Polypodiaceae	Lepisorus validinervis (Kunze) Li Wang	trnL-trnF	MH665160	FTOL v1.7
Polypodiaceae	Lepisorus venosus (Ching) comb. ined.	rbcL	MT169838	FTOL v1.7
Polypodiaceae	Lepisorus venosus (Ching) comb. ined.	rps4	MT169907	FTOL v1.7
Polypodiaceae	Lepisorus venosus (Ching) comb. ined.	rps4-trnS	MT169907	FTOL v1.7
Polypodiaceae	Lepisorus venosus (Ching) comb. ined.	trnL-trnF	MT169946	FTOL v1.7
Polypodiaceae	Lepisorus waltonii (Ching) S. L. Yu	plastome	MK287776	FTOL v1.7
Polypodiaceae	Lepisorus yamaokae Seriz. ined.	rbcL	AB575271	FTOL v1.7
Polypodiaceae	Lepisorus zippelii (Blume) C. F. Zhao, R. Wei & X. C. Zh	atpA	MH113553	FTOL v1.7
Polypodiaceae	Lepisorus zippelii (Blume) C. F. Zhao, R. Wei & X. C. Zh	atpB	MH175523	FTOL v1.7
Polypodiaceae	Lepisorus zippelii (Blume) C. F. Zhao, R. Wei & X. C. Zh	rbcL	MH051188	FTOL v1.7
Polypodiaceae	Lepisorus zippelii (Blume) C. F. Zhao, R. Wei & X. C. Zh	rps4	MH113487	FTOL v1.7
Polypodiaceae	Lepisorus zippelii (Blume) C. F. Zhao, R. Wei & X. C. Zh	rps4-trnS	MH113487	FTOL v1.7
Polypodiaceae	Lepisorus zippelii (Blume) C. F. Zhao, R. Wei & X. C. Zh	trnL-trnF	MH113520	FTOL v1.7
Polypodiaceae	Leptochilus × shintenensis (Hayata) Nakaike	atpA	MH113535	FTOL v1.7
Polypodiaceae	Leptochilus × shintenensis (Hayata) Nakaike	atpB	MH113568	FTOL v1.7
Polypodiaceae	Leptochilus × shintenensis (Hayata) Nakaike	rbcL	MH051169	FTOL v1.7
Polypodiaceae	Leptochilus × shintenensis (Hayata) Nakaike	rps4	MH113468	FTOL v1.7
Polypodiaceae	Leptochilus × shintenensis (Hayata) Nakaike	rps4-trnS	MH113468	FTOL v1.7
Polypodiaceae	Leptochilus × shintenensis (Hayata) Nakaike	trnL-trnF	MH113501	FTOL v1.7
Polypodiaceae	Leptochilus axillaris (Cav.) Kaulf.	atpB	JX103657	FTOL v1.7
Polypodiaceae	Leptochilus axillaris (Cav.) Kaulf.	rbcL	JX103699	FTOL v1.7
Polypodiaceae	Leptochilus axillaris (Cav.) Kaulf.	rps4	JX103741	FTOL v1.7
Polypodiaceae	Leptochilus axillaris (Cav.) Kaulf.	rps4-trnS	JX103741	FTOL v1.7
Polypodiaceae	Leptochilus axillaris (Cav.) Kaulf.	trnL-trnF	JX103783	FTOL v1.7
Polypodiaceae	Leptochilus cantoniensis (Baker) Ching	rbcL	MT137055	FTOL v1.7
Polypodiaceae	Leptochilus cantoniensis (Baker) Ching	rps4	MH665095	FTOL v1.7
Polypodiaceae	Leptochilus cantoniensis (Baker) Ching	rps4-trnS	MH665095	FTOL v1.7
Polypodiaceae	Leptochilus cantoniensis (Baker) Ching	trnL-trnF	MH665162	FTOL v1.7
Polypodiaceae	Leptochilus chingii Liang Zhang & Li Bing Zhang	atpB	MH768380	FTOL v1.7
Polypodiaceae	Leptochilus chingii Liang Zhang & Li Bing Zhang	rbcL	MH768437	FTOL v1.7
Polypodiaceae	Leptochilus chingii Liang Zhang & Li Bing Zhang	rps4	MH768502	FTOL v1.7
Polypodiaceae	Leptochilus chingii Liang Zhang & Li Bing Zhang	rps4-trnS	MH768502	FTOL v1.7
Polypodiaceae	Leptochilus chingii Liang Zhang & Li Bing Zhang	trnL-trnF	MH768565	FTOL v1.7
Polypodiaceae	Leptochilus decurrens Blume	plastome	MN044573	FTOL v1.7
Polypodiaceae	Leptochilus digitatus (Baker) Noot.	atpA	MH113528	FTOL v1.7
Polypodiaceae	Leptochilus digitatus (Baker) Noot.	atpB	MH113561	FTOL v1.7
Polypodiaceae	Leptochilus digitatus (Baker) Noot.	rbcL	MH051162	FTOL v1.7
Polypodiaceae	Leptochilus digitatus (Baker) Noot.	rps4	MH113461	FTOL v1.7
Polypodiaceae	Leptochilus digitatus (Baker) Noot.	rps4-trnS	MH113461	FTOL v1.7
Polypodiaceae	Leptochilus digitatus (Baker) Noot.	trnL-trnF	MH113495	FTOL v1.7
Polypodiaceae	Leptochilus dissimilialatus (Bonap.) Liang Zhang & Li B	atpB	MH768359	FTOL v1.7
Polypodiaceae	Leptochilus dissimilialatus (Bonap.) Liang Zhang & Li B	rbcL	MH768419	FTOL v1.7
Polypodiaceae	Leptochilus dissimilialatus (Bonap.) Liang Zhang & Li B	rps4	MH768481	FTOL v1.7
Polypodiaceae	Leptochilus dissimilialatus (Bonap.) Liang Zhang & Li B	rps4-trnS	MH768481	FTOL v1.7
Polypodiaceae	Leptochilus dissimilialatus (Bonap.) Liang Zhang & Li B	trnL-trnF	MH768547	FTOL v1.7
Polypodiaceae	Leptochilus ellipticus (Thunb.) Noot.	plastome	MT130679	FTOL v1.7
Polypodiaceae	Leptochilus evrardii (Tardieu) Liang Zhang & Li Bing Zh	atpB	MH768399	FTOL v1.7
Polypodiaceae	Leptochilus evrardii (Tardieu) Liang Zhang & Li Bing Zh	rbcL	MH768460	FTOL v1.7
Polypodiaceae	Leptochilus evrardii (Tardieu) Liang Zhang & Li Bing Zh	rps4	MH768525	FTOL v1.7
Polypodiaceae	Leptochilus evrardii (Tardieu) Liang Zhang & Li Bing Zh	rps4-trnS	MH768525	FTOL v1.7
Polypodiaceae	Leptochilus evrardii (Tardieu) Liang Zhang & Li Bing Zh	trnL-trnF	MH768584	FTOL v1.7
Polypodiaceae	Leptochilus flexilobus (Christ) Liang Zhang & Li Bing Zh	atpB	MH768397	FTOL v1.7
Polypodiaceae	Leptochilus flexilobus (Christ) Liang Zhang & Li Bing Zh	rbcL	MH768458	FTOL v1.7
Polypodiaceae	Leptochilus flexilobus (Christ) Liang Zhang & Li Bing Zh	rps4	MH768523	FTOL v1.7
Polypodiaceae	Leptochilus flexilobus (Christ) Liang Zhang & Li Bing Zh	rps4-trnS	MH768523	FTOL v1.7
Polypodiaceae	Leptochilus flexilobus (Christ) Liang Zhang & Li Bing Zh	trnL-trnF	MH768582	FTOL v1.7
Polypodiaceae	Leptochilus gracilis Z. L. Liang, Liang Zhang & Li Bing Z	rps4	MW142229	FTOL v1.7
Polypodiaceae	Leptochilus gracilis Z. L. Liang, Liang Zhang & Li Bing Z	rps4-trnS	MW142229	FTOL v1.7
Polypodiaceae	Leptochilus gracilis Z. L. Liang, Liang Zhang & Li Bing Z	trnL-trnF	MW142228	FTOL v1.7
Polypodiaceae	Leptochilus hemionitideus (C. Presl) Noot.	plastome	MH319943	FTOL v1.7
Polypodiaceae	Leptochilus henryi (Baker) X. C. Zhang	plastome	MW876333	FTOL v1.7
Polypodiaceae	Leptochilus heterophyllus (S. K. Wu & P. K. Lôc) comb. i	atpB	JX103646	FTOL v1.7
Polypodiaceae	Leptochilus heterophyllus (S. K. Wu & P. K. Lôc) comb. i	rbcL	JX103688	FTOL v1.7
Polypodiaceae	Leptochilus heterophyllus (S. K. Wu & P. K. Lôc) comb. i	rps4	JX103730	FTOL v1.7
Polypodiaceae	Leptochilus heterophyllus (S. K. Wu & P. K. Lôc) comb. i	rps4-trnS	JX103730	FTOL v1.7
Polypodiaceae	Leptochilus heterophyllus (S. K. Wu & P. K. Lôc) comb. i	trnL-trnF	JX103772	FTOL v1.7
Polypodiaceae	Leptochilus leveillei (Ching) X. C. Zhang	atpA	MH113532	FTOL v1.7
Polypodiaceae	Leptochilus leveillei (Ching) X. C. Zhang	atpB	MH113565	FTOL v1.7
Polypodiaceae	Leptochilus leveillei (Ching) X. C. Zhang	rbcL	MH051166	FTOL v1.7
Polypodiaceae	Leptochilus leveillei (Ching) X. C. Zhang	rps4	MH113465	FTOL v1.7
Polypodiaceae	Leptochilus leveillei (Ching) X. C. Zhang	rps4-trnS	MH113465	FTOL v1.7
Polypodiaceae	Leptochilus leveillei (Ching) X. C. Zhang	trnL-trnF	MH113498	FTOL v1.7
Polypodiaceae	Leptochilus macrophyllus (Blume) Noot.	plastome	MW876334	FTOL v1.7
Polypodiaceae	Leptochilus mengsongensis M. X. Zhao	rbcL	KY963656	FTOL v1.7
Polypodiaceae	Leptochilus oblongus Li Bing Zhang, Liang Zhang & N. T	atpB	MH768369	FTOL v1.7
Polypodiaceae	Leptochilus oblongus Li Bing Zhang, Liang Zhang & N. T	rbcL	MH768429	FTOL v1.7
Polypodiaceae	Leptochilus oblongus Li Bing Zhang, Liang Zhang & N. T	rps4	MH768491	FTOL v1.7
Polypodiaceae	Leptochilus oblongus Li Bing Zhang, Liang Zhang & N. T	rps4-trnS	MH768491	FTOL v1.7
Polypodiaceae	Leptochilus oblongus Li Bing Zhang, Liang Zhang & N. T	trnL-trnF	MH768557	FTOL v1.7
Polypodiaceae	Leptochilus pedunculatus (Hook. & Grev.) Fraser-Jenk.	atpA	MH113534	FTOL v1.7
Polypodiaceae	Leptochilus pedunculatus (Hook. & Grev.) Fraser-Jenk.	atpB	MH113567	FTOL v1.7
Polypodiaceae	Leptochilus pedunculatus (Hook. & Grev.) Fraser-Jenk.	rbcL	MH051168	FTOL v1.7
Polypodiaceae	Leptochilus pedunculatus (Hook. & Grev.) Fraser-Jenk.	rps4	MH113467	FTOL v1.7
Polypodiaceae	Leptochilus pedunculatus (Hook. & Grev.) Fraser-Jenk.	rps4-trnS	MH113467	FTOL v1.7
Polypodiaceae	Leptochilus pedunculatus (Hook. & Grev.) Fraser-Jenk.	trnL-trnF	MH113500	FTOL v1.7
Polypodiaceae	Leptochilus pentaphyllus (Baker) Liang Zhang & Li Bing	atpB	MH768411	FTOL v1.7
Polypodiaceae	Leptochilus pentaphyllus (Baker) Liang Zhang & Li Bing	rbcL	MH768474	FTOL v1.7
Polypodiaceae	Leptochilus pentaphyllus (Baker) Liang Zhang & Li Bing	rps4	MH768539	FTOL v1.7
Polypodiaceae	Leptochilus pentaphyllus (Baker) Liang Zhang & Li Bing	rps4-trnS	MH768539	FTOL v1.7
Polypodiaceae	Leptochilus pentaphyllus (Baker) Liang Zhang & Li Bing	trnL-trnF	MH768599	FTOL v1.7
Polypodiaceae	Leptochilus pothifolius (Buch.-Ham. ex D. Don) Fraser-J	atpA	MH113529	FTOL v1.7
Polypodiaceae	Leptochilus pothifolius (Buch.-Ham. ex D. Don) Fraser-J	atpB	MH113562	FTOL v1.7
Polypodiaceae	Leptochilus pothifolius (Buch.-Ham. ex D. Don) Fraser-J	rbcL	MH051163	FTOL v1.7
Polypodiaceae	Leptochilus pothifolius (Buch.-Ham. ex D. Don) Fraser-J	rps4	MH113462	FTOL v1.7
Polypodiaceae	Leptochilus pothifolius (Buch.-Ham. ex D. Don) Fraser-J	rps4-trnS	MH113462	FTOL v1.7
Polypodiaceae	Leptochilus pothifolius (Buch.-Ham. ex D. Don) Fraser-J	trnL-trnF	MH113496	FTOL v1.7
Polypodiaceae	Leptochilus pteropus (Blume) Fraser-Jenk.	plastome	MW876341	FTOL v1.7
Polypodiaceae	Leptochilus saxicola (H. G. Zhou & Hua Li) Liang Zhang	atpB	MH768410	FTOL v1.7
Polypodiaceae	Leptochilus saxicola (H. G. Zhou & Hua Li) Liang Zhang	rbcL	MH768471	FTOL v1.7
Polypodiaceae	Leptochilus saxicola (H. G. Zhou & Hua Li) Liang Zhang	rps4	MH768536	FTOL v1.7
Polypodiaceae	Leptochilus saxicola (H. G. Zhou & Hua Li) Liang Zhang	rps4-trnS	MH768536	FTOL v1.7
Polypodiaceae	Leptochilus saxicola (H. G. Zhou & Hua Li) Liang Zhang	trnL-trnF	MH768595	FTOL v1.7
Polypodiaceae	Leptochilus wrightii (Hook.) X. C. Zhang	atpA	MH113536	FTOL v1.7
Polypodiaceae	Leptochilus wrightii (Hook.) X. C. Zhang	atpB	MH113569	FTOL v1.7
Polypodiaceae	Leptochilus wrightii (Hook.) X. C. Zhang	rbcL	MH051170	FTOL v1.7
Polypodiaceae	Leptochilus wrightii (Hook.) X. C. Zhang	rps4	MH113469	FTOL v1.7
Polypodiaceae	Leptochilus wrightii (Hook.) X. C. Zhang	rps4-trnS	MH113469	FTOL v1.7
Polypodiaceae	Leptochilus wrightii (Hook.) X. C. Zhang	trnL-trnF	MH113502	FTOL v1.7
Polypodiaceae	Leucotrichum madagascariense Rakotondr. & Rouhan	atpB	JN654923	FTOL v1.7
Polypodiaceae	Leucotrichum madagascariense Rakotondr. & Rouhan	rbcL	JN654924	FTOL v1.7
Polypodiaceae	Leucotrichum madagascariense Rakotondr. & Rouhan	rps4-trnS	JN654949	FTOL v1.7
Polypodiaceae	Leucotrichum madagascariense Rakotondr. & Rouhan	trnL-trnF	JN654975	FTOL v1.7
Polypodiaceae	Leucotrichum mitchelliae (Baker) Labiak	atpB	GU376480	FTOL v1.7
Polypodiaceae	Leucotrichum mitchelliae (Baker) Labiak	rbcL	GU376487	FTOL v1.7
Polypodiaceae	Leucotrichum mitchelliae (Baker) Labiak	rps4-trnS	JN654938	FTOL v1.7
Polypodiaceae	Leucotrichum mitchelliae (Baker) Labiak	trnL-trnF	JN654964	FTOL v1.7
Polypodiaceae	Leucotrichum mortonii (Copel.) Labiak	atpB	GU376478	FTOL v1.7
Polypodiaceae	Leucotrichum mortonii (Copel.) Labiak	rbcL	GU376489	FTOL v1.7
Polypodiaceae	Leucotrichum organense (Gardner) Labiak	atpB	GU376484	FTOL v1.7
Polypodiaceae	Leucotrichum organense (Gardner) Labiak	rbcL	GU376491	FTOL v1.7
Polypodiaceae	Leucotrichum organense (Gardner) Labiak	rps4-trnS	JN654947	FTOL v1.7
Polypodiaceae	Leucotrichum organense (Gardner) Labiak	trnL-trnF	JN654973	FTOL v1.7
Polypodiaceae	Leucotrichum pseudomitchelliae (Lellinger) Labiak	atpB	AY459484	FTOL v1.7
Polypodiaceae	Leucotrichum pseudomitchelliae (Lellinger) Labiak	rbcL	AY460652	FTOL v1.7
Polypodiaceae	Leucotrichum schenckii (Hieron.) Labiak	atpB	AY459483	FTOL v1.7
Polypodiaceae	Leucotrichum schenckii (Hieron.) Labiak	rbcL	AY460651	FTOL v1.7
Polypodiaceae	Lomaphlebia linearis (Sw.) J. Sm.	rbcL	MK319113	FTOL v1.7
Polypodiaceae	Lomaphlebia linearis (Sw.) J. Sm.	trnL-trnF	MK319011	FTOL v1.7
Polypodiaceae	Lomaphlebia turquina (Maxon) Sundue & Ranker ined.	atpB	KM218814	FTOL v1.7
Polypodiaceae	Lomaphlebia turquina (Maxon) Sundue & Ranker ined.	rbcL	KM218800	FTOL v1.7
Polypodiaceae	Lomaphlebia turquina (Maxon) Sundue & Ranker ined.	trnL-trnF	KM106069	FTOL v1.7
Polypodiaceae	Loxogramme abyssinica (Baker) M. G. Price	atpA	EF463826	FTOL v1.7
Polypodiaceae	Loxogramme abyssinica (Baker) M. G. Price	atpB	EF463498	FTOL v1.7
Polypodiaceae	Loxogramme abyssinica (Baker) M. G. Price	rbcL	EF463252	FTOL v1.7
Polypodiaceae	Loxogramme abyssinica (Baker) M. G. Price	rps4	DQ164474	FTOL v1.7
Polypodiaceae	Loxogramme abyssinica (Baker) M. G. Price	rps4-trnS	DQ164474	FTOL v1.7
Polypodiaceae	Loxogramme abyssinica (Baker) M. G. Price	trnL-trnF	DQ164506	FTOL v1.7
Polypodiaceae	Loxogramme acroscopa (Christ) C. Chr.	plastome	MW876336	FTOL v1.7
Polypodiaceae	Loxogramme avenia (Blume) C. Presl	plastome	MW876335	FTOL v1.7
Polypodiaceae	Loxogramme chinensis Ching	plastome	MT130671	FTOL v1.7
Polypodiaceae	Loxogramme cuspidata (Zenker) M. G. Price	rbcL	MF450443	FTOL v1.7
Polypodiaceae	Loxogramme cuspidata (Zenker) M. G. Price	rps4	MF450464	FTOL v1.7

Polypodiaceae	Loxogramme cuspidata (Zenker) M. G. Price	rps4-trnS	MF450464	FTOL v1.7
Polypodiaceae	Loxogramme cuspidata (Zenker) M. G. Price	trnL-trnF	MN912806	FTOL v1.7
Polypodiaceae	Loxogramme dictyopteris (Mett.) Copel.	rbcL	KT626735	FTOL v1.7
Polypodiaceae	Loxogramme dictyopteris (Mett.) Copel.	rps4	AY096214	FTOL v1.7
Polypodiaceae	Loxogramme dictyopteris (Mett.) Copel.	rps4-trnS	AY096214	FTOL v1.7
Polypodiaceae	Loxogramme dictyopteris (Mett.) Copel.	trnL-trnF	DQ227303	FTOL v1.7
Polypodiaceae	Loxogramme grammitoides (Baker) C. Chr.	rbcL	MF450445	FTOL v1.7
Polypodiaceae	Loxogramme grammitoides (Baker) C. Chr.	rps4	MF450466	FTOL v1.7
Polypodiaceae	Loxogramme grammitoides (Baker) C. Chr.	rps4-trnS	MF450466	FTOL v1.7
Polypodiaceae	Loxogramme involuta (D. Don) C. Presl	plastome	MT130617	FTOL v1.7
Polypodiaceae	Loxogramme lanceolata (Sw.) C. Presl	rbcL	KF992501	FTOL v1.7
Polypodiaceae	Loxogramme mexicana (Fée) C. Chr.	trnL-trnF	DQ227301	FTOL v1.7
Polypodiaceae	Loxogramme parksii Copel.	rbcL	MT657668	FTOL v1.7
Polypodiaceae	Loxogramme salicifolia (Makino) Makino	rbcL	DQ227294	FTOL v1.7
Polypodiaceae	Loxogramme salicifolia (Makino) Makino	rps4	DQ227297	FTOL v1.7
Polypodiaceae	Loxogramme salicifolia (Makino) Makino	rps4-trnS	DQ227297	FTOL v1.7
Polypodiaceae	Loxogramme salicifolia (Makino) Makino	trnL-trnF	DQ227302	FTOL v1.7
Polypodiaceae	Melpomene albicans Lehnert	atpB	FJ011009	FTOL v1.7
Polypodiaceae	Melpomene albicans Lehnert	rbcL	FJ011046	FTOL v1.7
Polypodiaceae	Melpomene allosuroides (Rosenst.) A. R. Sm. & R. C. M	atpB	GU376628	FTOL v1.7
Polypodiaceae	Melpomene allosuroides (Rosenst.) A. R. Sm. & R. C. M	rbcL	GU387038	FTOL v1.7
Polypodiaceae	Melpomene allosuroides (Rosenst.) A. R. Sm. & R. C. M	rps4-trnS	GU387109	FTOL v1.7
Polypodiaceae	Melpomene allosuroides (Rosenst.) A. R. Sm. & R. C. M	trnL-trnF	GU387273	FTOL v1.7
Polypodiaceae	Melpomene anazalea Sundue & Lehnert	atpB	GU476773	FTOL v1.7
Polypodiaceae	Melpomene anazalea Sundue & Lehnert	rbcL	GU476898	FTOL v1.7
Polypodiaceae	Melpomene anazalea Sundue & Lehnert	trnL-trnF	GU476662	FTOL v1.7
Polypodiaceae	Melpomene caput-gorgonis Lehnert	atpB	FJ011010	FTOL v1.7
Polypodiaceae	Melpomene caput-gorgonis Lehnert	rbcL	FJ011047	FTOL v1.7
Polypodiaceae	Melpomene erecta (C. V. Morton) A. R. Sm. & R. C. Mo	atpB	GU376629	FTOL v1.7
Polypodiaceae	Melpomene erecta (C. V. Morton) A. R. Sm. & R. C. Mo	rbcL	GU387013	FTOL v1.7
Polypodiaceae	Melpomene erecta (C. V. Morton) A. R. Sm. & R. C. Mo	rps4-trnS	GU387110	FTOL v1.7
Polypodiaceae	Melpomene erecta (C. V. Morton) A. R. Sm. & R. C. Mo	trnL-trnF	GU387274	FTOL v1.7
Polypodiaceae	Melpomene firma (J. Sm.) A. R. Sm. & R. C. Moran	atpB	GU376630	FTOL v1.7
Polypodiaceae	Melpomene firma (J. Sm.) A. R. Sm. & R. C. Moran	rbcL	GU387035	FTOL v1.7
Polypodiaceae	Melpomene firma (J. Sm.) A. R. Sm. & R. C. Moran	rps4-trnS	GU387112	FTOL v1.7
Polypodiaceae	Melpomene firma (J. Sm.) A. R. Sm. & R. C. Moran	trnL-trnF	GU387276	FTOL v1.7
Polypodiaceae	Melpomene flabelliformis (Poir.) A. R. Sm. & R. C. Mor	plastome	MZ357089	FTOL v1.7
Polypodiaceae	Melpomene flagellata Lehnert	rbcL	FJ011084	FTOL v1.7
Polypodiaceae	Melpomene gracilis (Hook.) A. R. Sm.	atpB	FJ011006	FTOL v1.7
Polypodiaceae	Melpomene gracilis (Hook.) A. R. Sm.	rbcL	FJ011043	FTOL v1.7
Polypodiaceae	Melpomene jimenezii Lehnert	rbcL	FJ011085	FTOL v1.7
Polypodiaceae	Melpomene leptostoma (Fée) A. R. Sm. & R. C. Moran	rbcL	MW620278	FTOL v1.7
Polypodiaceae	Melpomene melanosticta (Kunze) A. R. Sm. & R. C. Mo	atpB	GU376633	FTOL v1.7
Polypodiaceae	Melpomene melanosticta (Kunze) A. R. Sm. & R. C. Mo	rbcL	GU387024	FTOL v1.7
Polypodiaceae	Melpomene melanosticta (Kunze) A. R. Sm. & R. C. Mo	rps4-trnS	GU387115	FTOL v1.7
Polypodiaceae	Melpomene melanosticta (Kunze) A. R. Sm. & R. C. Mo	trnL-trnF	GU387279	FTOL v1.7
Polypodiaceae	Melpomene michaelis Lehnert	rbcL	FJ011086	FTOL v1.7
Polypodiaceae	Melpomene moniliformis (Lag. ex Sw.) A. R. Sm. & R.	atpB	AY459487	FTOL v1.7
Polypodiaceae	Melpomene moniliformis (Lag. ex Sw.) A. R. Sm. & R.	rbcL	AY460655	FTOL v1.7
Polypodiaceae	Melpomene occidentalis Lehnert	atpB	GU476776	FTOL v1.7
Polypodiaceae	Melpomene occidentalis Lehnert	rbcL	GU476877	FTOL v1.7
Polypodiaceae	Melpomene occidentalis Lehnert	trnL-trnF	GU476666	FTOL v1.7
Polypodiaceae	Melpomene paradoxa Lehnert	atpB	FJ011025	FTOL v1.7
Polypodiaceae	Melpomene paradoxa Lehnert	rbcL	FJ011062	FTOL v1.7
Polypodiaceae	Melpomene personata Lehnert	atpB	GU376634	FTOL v1.7
Polypodiaceae	Melpomene personata Lehnert	rbcL	GU387007	FTOL v1.7
Polypodiaceae	Melpomene personata Lehnert	rps4-trnS	GU387116	FTOL v1.7
Polypodiaceae	Melpomene personata Lehnert	trnL-trnF	GU387280	FTOL v1.7
Polypodiaceae	Melpomene peruviana	atpB	FJ011030	FTOL v1.7
Polypodiaceae	Melpomene peruviana	rbcL	FJ011067	FTOL v1.7
Polypodiaceae	Melpomene pilosissima (M. Martens & Galeotti) A. R.	atpB	GU376636	FTOL v1.7
Polypodiaceae	Melpomene pilosissima (M. Martens & Galeotti) A. R.	rbcL	GU386993	FTOL v1.7
Polypodiaceae	Melpomene pilosissima (M. Martens & Galeotti) A. R.	rps4-trnS	GU387118	FTOL v1.7
Polypodiaceae	Melpomene pilosissima (M. Martens & Galeotti) A. R.	trnL-trnF	GU387281	FTOL v1.7
Polypodiaceae	Melpomene pseudonutans (Christ & Rosenst.) A. R. Sm	atpB	AY459489	FTOL v1.7
Polypodiaceae	Melpomene pseudonutans (Christ & Rosenst.) A. R. Sm	rbcL	AY460657	FTOL v1.7
Polypodiaceae	Melpomene sklenarii Lehnert	atpB	FJ011034	FTOL v1.7
Polypodiaceae	Melpomene sklenarii Lehnert	rbcL	FJ011071	FTOL v1.7
Polypodiaceae	Melpomene sodiroi (Christ & Rosenst.) A. R. Sm. & R.	atpB	FJ011035	FTOL v1.7
Polypodiaceae	Melpomene sodiroi (Christ & Rosenst.) A. R. Sm. & R.	rbcL	FJ011072	FTOL v1.7
Polypodiaceae	Melpomene vernicosa (Copel.) A. R. Sm. & R. C. Moran	atpB	GU476778	FTOL v1.7
Polypodiaceae	Melpomene vernicosa (Copel.) A. R. Sm. & R. C. Moran	rbcL	GU476867	FTOL v1.7
Polypodiaceae	Melpomene vernicosa (Copel.) A. R. Sm. & R. C. Moran	trnL-trnF	GU476668	FTOL v1.7
Polypodiaceae	Melpomene vulcanica Lehnert	atpB	FJ011037	FTOL v1.7
Polypodiaceae	Melpomene vulcanica Lehnert	rbcL	FJ011074	FTOL v1.7
Polypodiaceae	Melpomene wolfii (Hieron.) A. R. Sm. & R. C. Moran	atpB	FJ011039	FTOL v1.7
Polypodiaceae	Melpomene wolfii (Hieron.) A. R. Sm. & R. C. Moran	rbcL	FJ011076	FTOL v1.7
Polypodiaceae	Melpomene xiphopteroides (Liebm.) A. R. Sm. & R. C.	atpB	GU376638	FTOL v1.7
Polypodiaceae	Melpomene xiphopteroides (Liebm.) A. R. Sm. & R. C.	rbcL	GU387040	FTOL v1.7
Polypodiaceae	Melpomene xiphopteroides (Liebm.) A. R. Sm. & R. C.	rps4-trnS	GU387120	FTOL v1.7
Polypodiaceae	Melpomene xiphopteroides (Liebm.) A. R. Sm. & R. C.	trnL-trnF	GU387283	FTOL v1.7
Polypodiaceae	Melpomene zempoaltepetlensis (Mickel & Beitel) A. R	rbcL	MK319126	FTOL v1.7
Polypodiaceae	Melpomene zempoaltepetlensis (Mickel & Beitel) A. R	trnL-trnF	MK319024	FTOL v1.7
Polypodiaceae	Microgramma baldwinii Brade	rbcL	KY847861	FTOL v1.7
Polypodiaceae	Microgramma baldwinii Brade	rps4	KY847866	FTOL v1.7
Polypodiaceae	Microgramma baldwinii Brade	rps4-trnS	KY847866	FTOL v1.7
Polypodiaceae	Microgramma bifrons (Hook.) Lellinger	atpA	EF463828	FTOL v1.7
Polypodiaceae	Microgramma bifrons (Hook.) Lellinger	atpB	EF463499	FTOL v1.7
Polypodiaceae	Microgramma bifrons (Hook.) Lellinger	rbcL	AY362582	FTOL v1.7
Polypodiaceae	Microgramma bifrons (Hook.) Lellinger	rps4	AY362654	FTOL v1.7
Polypodiaceae	Microgramma bifrons (Hook.) Lellinger	rps4-trnS	AY362654	FTOL v1.7
Polypodiaceae	Microgramma bifrons (Hook.) Lellinger	trnL-trnF	DQ642224	FTOL v1.7
Polypodiaceae	Microgramma brunei (Wercklé ex Christ) Lellinger	rbcL	MT583743	FTOL v1.7
Polypodiaceae	Microgramma brunei (Wercklé ex Christ) Lellinger	rps4	MT806111	FTOL v1.7
Polypodiaceae	Microgramma brunei (Wercklé ex Christ) Lellinger	trnL-trnF	MT568806	FTOL v1.7
Polypodiaceae	Microgramma crispata (Fée) R. M. Tryon & A. F. Tryon	rbcL	MT583745	FTOL v1.7
Polypodiaceae	Microgramma dictyophylla (Kunze ex Mett.) de la Sota	atpB	MN781295	FTOL v1.7
Polypodiaceae	Microgramma dictyophylla (Kunze ex Mett.) de la Sota	rbcL	MN781335	FTOL v1.7
Polypodiaceae	Microgramma dictyophylla (Kunze ex Mett.) de la Sota	rps4	MN781379	FTOL v1.7
Polypodiaceae	Microgramma dictyophylla (Kunze ex Mett.) de la Sota	rps4-trnS	MN781379	FTOL v1.7
Polypodiaceae	Microgramma geminata (Schrad.) R. M. Tryon & A. F.	rbcL	MT583747	FTOL v1.7
Polypodiaceae	Microgramma geminata (Schrad.) R. M. Tryon & A. F.	rps4	MT806115	FTOL v1.7
Polypodiaceae	Microgramma geminata (Schrad.) R. M. Tryon & A. F.	rps4-trnS	MT806115	FTOL v1.7
Polypodiaceae	Microgramma geminata (Schrad.) R. M. Tryon & A. F.	trnL-trnF	MT568810	FTOL v1.7
Polypodiaceae	Microgramma heterophylla (L.) Wherry	rbcL	MT583749	FTOL v1.7
Polypodiaceae	Microgramma heterophylla (L.) Wherry	rps4	MT806116	FTOL v1.7
Polypodiaceae	Microgramma heterophylla (L.) Wherry	rps4-trnS	MT806116	FTOL v1.7
Polypodiaceae	Microgramma heterophylla (L.) Wherry	trnL-trnF	MT568811	FTOL v1.7
Polypodiaceae	Microgramma latevagans (Maxon & C. Chr.) Lellinger	rps4	EU250356	FTOL v1.7
Polypodiaceae	Microgramma latevagans (Maxon & C. Chr.) Lellinger	rps4-trnS	EU250356	FTOL v1.7
Polypodiaceae	Microgramma latevagans (Maxon & C. Chr.) Lellinger	trnL-trnF	EF104517	FTOL v1.7
Polypodiaceae	Microgramma lindbergii (Mett.) de la Sota	rbcL	MF317957	FTOL v1.7
Polypodiaceae	Microgramma lindbergii (Mett.) de la Sota	rps4	MT806119	FTOL v1.7
Polypodiaceae	Microgramma lindbergii (Mett.) de la Sota	rps4-trnS	MT806119	FTOL v1.7
Polypodiaceae	Microgramma lindbergii (Mett.) de la Sota	trnL-trnF	MT568813	FTOL v1.7
Polypodiaceae	Microgramma lycopodioides (L.) Copel.	plastome	MT130703	FTOL v1.7
Polypodiaceae	Microgramma mauritiana (Willd.) Tardieu	rbcL	DQ642148	FTOL v1.7
Polypodiaceae	Microgramma mauritiana (Willd.) Tardieu	rps4	DQ642186	FTOL v1.7
Polypodiaceae	Microgramma mauritiana (Willd.) Tardieu	rps4-trnS	DQ642186	FTOL v1.7
Polypodiaceae	Microgramma mauritiana (Willd.) Tardieu	trnL-trnF	DQ642226	FTOL v1.7
Polypodiaceae	Microgramma megalophylla (Desv.) de la Sota	rbcL	AY362578	FTOL v1.7
Polypodiaceae	Microgramma megalophylla (Desv.) de la Sota	rps4	AY362650	FTOL v1.7
Polypodiaceae	Microgramma megalophylla (Desv.) de la Sota	rps4-trnS	AY362650	FTOL v1.7
Polypodiaceae	Microgramma megalophylla (Desv.) de la Sota	trnL-trnF	MT568818	FTOL v1.7
Polypodiaceae	Microgramma microsoroides Salino, T. E. Almeida & A	rbcL	EU292730	FTOL v1.7
Polypodiaceae	Microgramma microsoroides Salino, T. E. Almeida & A	rps4	EU292731	FTOL v1.7
Polypodiaceae	Microgramma microsoroides Salino, T. E. Almeida & A	rps4-trnS	EU292731	FTOL v1.7
Polypodiaceae	Microgramma microsoroides Salino, T. E. Almeida & A	trnL-trnF	EU292732	FTOL v1.7
Polypodiaceae	Microgramma mortoniana de la Sota	rbcL	MT583757	FTOL v1.7
Polypodiaceae	Microgramma mortoniana de la Sota	rps4	MT806126	FTOL v1.7
Polypodiaceae	Microgramma mortoniana de la Sota	rps4-trnS	MT806126	FTOL v1.7
Polypodiaceae	Microgramma mortoniana de la Sota	trnL-trnF	MT568819	FTOL v1.7
Polypodiaceae	Microgramma nitida (J. Sm.) A. R. Sm.	rbcL	EU250350	FTOL v1.7
Polypodiaceae	Microgramma nitida (J. Sm.) A. R. Sm.	rps4	EU250357	FTOL v1.7
Polypodiaceae	Microgramma nitida (J. Sm.) A. R. Sm.	rps4-trnS	EU250357	FTOL v1.7
Polypodiaceae	Microgramma nitida (J. Sm.) A. R. Sm.	trnL-trnF	EF104518	FTOL v1.7
Polypodiaceae	Microgramma percussa (Cav.) de la Sota	atpB	MN781296	FTOL v1.7
Polypodiaceae	Microgramma percussa (Cav.) de la Sota	rbcL	MN781332	FTOL v1.7
Polypodiaceae	Microgramma percussa (Cav.) de la Sota	rps4	MN781380	FTOL v1.7
Polypodiaceae	Microgramma percussa (Cav.) de la Sota	rps4-trnS	MN781380	FTOL v1.7
Polypodiaceae	Microgramma persicariifolia (Schrad.) C. Presl	rbcL	KT780753	FTOL v1.7
Polypodiaceae	Microgramma persicariifolia (Schrad.) C. Presl	rps4	KT794133	FTOL v1.7
Polypodiaceae	Microgramma persicariifolia (Schrad.) C. Presl	rps4-trnS	KT794133	FTOL v1.7
Polypodiaceae	Microgramma persicariifolia (Schrad.) C. Presl	trnL-trnF	MT568824	FTOL v1.7
Polypodiaceae	Microgramma piloselloides (L.) Copel.	rbcL	MT583763	FTOL v1.7
Polypodiaceae	Microgramma reptans (Cav.) A. R. Sm.	rbcL	MT708071	FTOL v1.7
Polypodiaceae	Microgramma rosmarinifolia (Kunth) R. M. Tryon & A.	rbcL	MT583766	FTOL v1.7
Polypodiaceae	Microgramma rosmarinifolia (Kunth) R. M. Tryon & A.	rps4	MT806135	FTOL v1.7
Polypodiaceae	Microgramma rosmarinifolia (Kunth) R. M. Tryon & A.	rps4-trnS	MT806135	FTOL v1.7
Polypodiaceae	Microgramma rosmarinifolia (Kunth) R. M. Tryon & A.	trnL-trnF	MT568829	FTOL v1.7
Polypodiaceae	Microgramma squamulosa (Kaulf.) de la Sota	rbcL	DQ642150	FTOL v1.7
Polypodiaceae	Microgramma squamulosa (Kaulf.) de la Sota	rps4	DQ642188	FTOL v1.7
Polypodiaceae	Microgramma squamulosa (Kaulf.) de la Sota	rps4-trnS	DQ642188	FTOL v1.7
Polypodiaceae	Microgramma squamulosa (Kaulf.) de la Sota	trnL-trnF	DQ642228	FTOL v1.7
Polypodiaceae	Microgramma tecta (Kaulf.) Alston	rbcL	AY362580	FTOL v1.7
Polypodiaceae	Microgramma thurnii (Baker) R. M. Tryon & Stolze	rbcL	MT583769	FTOL v1.7
Polypodiaceae	Microgramma thurnii (Baker) R. M. Tryon & Stolze	rps4	MT806137	FTOL v1.7
Polypodiaceae	Microgramma thurnii (Baker) R. M. Tryon & Stolze	rps4-trnS	MT806137	FTOL v1.7
Polypodiaceae	Microgramma thurnii (Baker) R. M. Tryon & Stolze	trnL-trnF	MT568832	FTOL v1.7
Polypodiaceae	Microgramma tobagensis (C. Chr.) C. D. Adams & Baks	rbcL	MF318001	FTOL v1.7
Polypodiaceae	Microgramma tobagensis (C. Chr.) C. D. Adams & Baks	rps4-trnS	MF318182	FTOL v1.7
Polypodiaceae	Microgramma tobagensis (C. Chr.) C. D. Adams & Baks	trnL-trnF	MF318427	FTOL v1.7
Polypodiaceae	Microgramma vacciniifolia (Langsd. & Fisch.) Copel.	plastome	MW876337	FTOL v1.7
Polypodiaceae	Micropolypodium okuboi (Yatabe) Hayata	plastome	MW876338	FTOL v1.7
Polypodiaceae	Micropolypodium sikkimense (Hieron.) X. C. Zhang	plastome	MW876339	FTOL v1.7
Polypodiaceae	Microsorum × tohieaense J. H. Nitta	rbcL	MG452032	FTOL v1.7
Polypodiaceae	Microsorum × tohieaense J. H. Nitta	trnL-trnF	MG427077	FTOL v1.7
Polypodiaceae	Microsorum biseriatum (Bosman) Noot.	atpA	MH113554	FTOL v1.7
Polypodiaceae	Microsorum biseriatum (Bosman) Noot.	atpB	MH113587	FTOL v1.7
Polypodiaceae	Microsorum biseriatum (Bosman) Noot.	rbcL	MH051189	FTOL v1.7
Polypodiaceae	Microsorum biseriatum (Bosman) Noot.	rps4	MH113488	FTOL v1.7
Polypodiaceae	Microsorum biseriatum (Bosman) Noot.	rps4-trnS	MH113488	FTOL v1.7
Polypodiaceae	Microsorum biseriatum (Bosman) Noot.	trnL-trnF	MH113521	FTOL v1.7
Polypodiaceae	Microsorum commutatum (Blume) Copel.	atpA	MH113537	FTOL v1.7
Polypodiaceae	Microsorum commutatum (Blume) Copel.	atpB	MH113570	FTOL v1.7
Polypodiaceae	Microsorum commutatum (Blume) Copel.	rbcL	MH051171	FTOL v1.7
Polypodiaceae	Microsorum commutatum (Blume) Copel.	rps4	MH113470	FTOL v1.7
Polypodiaceae	Microsorum commutatum (Blume) Copel.	rps4-trnS	MH113470	FTOL v1.7
Polypodiaceae	Microsorum commutatum (Blume) Copel.	trnL-trnF	MH113503	FTOL v1.7
Polypodiaceae	Microsorum cuspidatum (D. Don) Tagawa	plastome	MT364353	FTOL v1.7
Polypodiaceae	Microsorum glossophyllum (Copel.) Copel.	atpA	MH175522	FTOL v1.7
Polypodiaceae	Microsorum glossophyllum (Copel.) Copel.	atpB	MH113578	FTOL v1.7
Polypodiaceae	Microsorum glossophyllum (Copel.) Copel.	rbcL	MH051180	FTOL v1.7
Polypodiaceae	Microsorum glossophyllum (Copel.) Copel.	rps4	MH113478	FTOL v1.7
Polypodiaceae	Microsorum glossophyllum (Copel.) Copel.	rps4-trnS	MH113478	FTOL v1.7
Polypodiaceae	Microsorum glossophyllum (Copel.) Copel.	trnL-trnF	MH113512	FTOL v1.7
Polypodiaceae	Microsorum grossum (Langsd. & Fisch.) S. B. Andrews	atpA	EF463831	FTOL v1.7

Polypodiaceae	Microsorum grossum (Langsd. & Fisch.) S. B. Andrews	atpB	EF463500	FTOL v1.7
Polypodiaceae	Microsorum grossum (Langsd. & Fisch.) S. B. Andrews	rbcL	EF463253	FTOL v1.7
Polypodiaceae	Microsorum hainanense Noot.	rbcL	EU482960	FTOL v1.7
Polypodiaceae	Microsorum hainanense Noot.	rps4	EU363263	FTOL v1.7
Polypodiaceae	Microsorum hainanense Noot.	rps4-trnS	EU363263	FTOL v1.7
Polypodiaceae	Microsorum hainanense Noot.	trnL-trnF	EU483057	FTOL v1.7
Polypodiaceae	Microsorum heterocarpum (Blume) Ching	atpA	MH664993	FTOL v1.7
Polypodiaceae	Microsorum heterocarpum (Blume) Ching	atpB	MH665008	FTOL v1.7
Polypodiaceae	Microsorum heterocarpum (Blume) Ching	rbcL	MH665072	FTOL v1.7
Polypodiaceae	Microsorum heterocarpum (Blume) Ching	rps4	MH665138	FTOL v1.7
Polypodiaceae	Microsorum heterocarpum (Blume) Ching	rps4-trnS	MH665137	FTOL v1.7
Polypodiaceae	Microsorum heterocarpum (Blume) Ching	trnL-trnF	MH665204	FTOL v1.7
Polypodiaceae	Microsorum insigne (Blume) Copel.	plastome	MW876340	FTOL v1.7
Polypodiaceae	Microsorum maximum (Brack.) Copel.	rbcL	KY099833	FTOL v1.7
Polypodiaceae	Microsorum maximum (Brack.) Copel.	trnL-trnF	MG427066	FTOL v1.7
Polypodiaceae	Microsorum membranifolium (R. Br.) Ching	atpA	MH664996	FTOL v1.7
Polypodiaceae	Microsorum membranifolium (R. Br.) Ching	atpB	MH665011	FTOL v1.7
Polypodiaceae	Microsorum membranifolium (R. Br.) Ching	rbcL	MH665077	FTOL v1.7
Polypodiaceae	Microsorum membranifolium (R. Br.) Ching	rps4	MH665143	FTOL v1.7
Polypodiaceae	Microsorum membranifolium (R. Br.) Ching	rps4-trnS	MH665143	FTOL v1.7
Polypodiaceae	Microsorum membranifolium (R. Br.) Ching	trnL-trnF	MH665209	FTOL v1.7
Polypodiaceae	Microsorum musifolium (Blume) Copel.	atpA	MH664998	FTOL v1.7
Polypodiaceae	Microsorum musifolium (Blume) Copel.	atpB	MH665013	FTOL v1.7
Polypodiaceae	Microsorum musifolium (Blume) Copel.	rbcL	MH665079	FTOL v1.7
Polypodiaceae	Microsorum musifolium (Blume) Copel.	rps4	MH665145	FTOL v1.7
Polypodiaceae	Microsorum musifolium (Blume) Copel.	rps4-trnS	MH665145	FTOL v1.7
Polypodiaceae	Microsorum musifolium (Blume) Copel.	trnL-trnF	MH665211	FTOL v1.7
Polypodiaceae	Microsorum pappei (Mett. ex Kuhn) Tardieu	rbcL	AF470336	FTOL v1.7
Polypodiaceae	Microsorum pappei (Mett. ex Kuhn) Tardieu	trnL-trnF	AY083639	FTOL v1.7
Polypodiaceae	Microsorum papuanum (Baker) Parris	rbcL	DQ642162	FTOL v1.7
Polypodiaceae	Microsorum papuanum (Baker) Parris	trnL-trnF	DQ642246	FTOL v1.7
Polypodiaceae	Microsorum punctatum (L.) Copel.	plastome	MW876342	FTOL v1.7
Polypodiaceae	Microsorum rubidum (J. Sm.) Copel.	plastome	MT130640	FTOL v1.7
Polypodiaceae	Microsorum scolopendria (Burm. fil.) Copel.	atpA	MH113555	FTOL v1.7
Polypodiaceae	Microsorum scolopendria (Burm. fil.) Copel.	atpB	MH113588	FTOL v1.7
Polypodiaceae	Microsorum scolopendria (Burm. fil.) Copel.	rbcL	MH051190	FTOL v1.7
Polypodiaceae	Microsorum scolopendria (Burm. fil.) Copel.	rps4	MH113489	FTOL v1.7
Polypodiaceae	Microsorum scolopendria (Burm. fil.) Copel.	rps4-trnS	MH113489	FTOL v1.7
Polypodiaceae	Microsorum scolopendria (Burm. fil.) Copel.	trnL-trnF	MH113522	FTOL v1.7
Polypodiaceae	Microsorum siamense Boonkerd	plastome	MT801156	FTOL v1.7
Polypodiaceae	Microsorum spectrum (Kaulf.) Copel.	rbcL	MT657633	FTOL v1.7
Polypodiaceae	Microsorum spectrum (Kaulf.) Copel.	trnL-trnF	MG427075	FTOL v1.7
Polypodiaceae	Microsorum steerei (Harr.) Ching	plastome	MW876343	FTOL v1.7
Polypodiaceae	Microsorum thailandicum Boonkerd & Noot.	rbcL	EU482969	FTOL v1.7
Polypodiaceae	Microsorum thailandicum Boonkerd & Noot.	trnL-trnF	EU483066	FTOL v1.7
Polypodiaceae	Microsorum whiteheadii A. R. Sm. & Hoshiz.	rbcL	EU482970	FTOL v1.7
Polypodiaceae	Microsorum whiteheadii A. R. Sm. & Hoshiz.	trnL-trnF	EU483067	FTOL v1.7
Polypodiaceae	Moranopteris achilleifolia (Kaulf.) R. Y. Hirai & J. Prado	atpB	AY459499	FTOL v1.7
Polypodiaceae	Moranopteris achilleifolia (Kaulf.) R. Y. Hirai & J. Prado	rbcL	AY460666	FTOL v1.7
Polypodiaceae	Moranopteris aphelolepis (C. V. Morton) R. Y. Hirai & J.	atpB	JF514066	FTOL v1.7
Polypodiaceae	Moranopteris aphelolepis (C. V. Morton) R. Y. Hirai & J.	rbcL	JF513996	FTOL v1.7
Polypodiaceae	Moranopteris aphelolepis (C. V. Morton) R. Y. Hirai & J.	trnL-trnF	JF514030	FTOL v1.7
Polypodiaceae	Moranopteris basiattenuata (Jenman) R. Y. Hirai & J. P	atpB	JF514058	FTOL v1.7
Polypodiaceae	Moranopteris basiattenuata (Jenman) R. Y. Hirai & J. P	rbcL	JF513988	FTOL v1.7
Polypodiaceae	Moranopteris basiattenuata (Jenman) R. Y. Hirai & J. P	trnL-trnF	JF514022	FTOL v1.7
Polypodiaceae	Moranopteris blepharidea (Copel.) R. Y. Hirai & J. Prad	atpB	JF514065	FTOL v1.7
Polypodiaceae	Moranopteris blepharidea (Copel.) R. Y. Hirai & J. Prad	rbcL	JF513995	FTOL v1.7
Polypodiaceae	Moranopteris blepharidea (Copel.) R. Y. Hirai & J. Prad	rps4-trnS	KM106154	FTOL v1.7
Polypodiaceae	Moranopteris blepharidea (Copel.) R. Y. Hirai & J. Prad	trnL-trnF	JF514029	FTOL v1.7
Polypodiaceae	Moranopteris caucana (Hieron.) R. Y. Hirai & J. Prado	atpB	JF514071	FTOL v1.7
Polypodiaceae	Moranopteris caucana (Hieron.) R. Y. Hirai & J. Prado	rbcL	JF514002	FTOL v1.7
Polypodiaceae	Moranopteris caucana (Hieron.) R. Y. Hirai & J. Prado	rps4-trnS	KM106155	FTOL v1.7
Polypodiaceae	Moranopteris caucana (Hieron.) R. Y. Hirai & J. Prado	trnL-trnF	JF514035	FTOL v1.7
Polypodiaceae	Moranopteris cookii (Underw. & Maxon) R. Y. Hirai & J.	atpB	JF514076	FTOL v1.7
Polypodiaceae	Moranopteris cookii (Underw. & Maxon) R. Y. Hirai & J.	rbcL	JF514007	FTOL v1.7
Polypodiaceae	Moranopteris cookii (Underw. & Maxon) R. Y. Hirai & J.	rps4-trnS	KM106156	FTOL v1.7
Polypodiaceae	Moranopteris cookii (Underw. & Maxon) R. Y. Hirai & J.	trnL-trnF	JF514040	FTOL v1.7
Polypodiaceae	Moranopteris gradata (Baker) R. Y. Hirai & J. Prado	atpB	JF514077	FTOL v1.7
Polypodiaceae	Moranopteris gradata (Baker) R. Y. Hirai & J. Prado	rbcL	JF514009	FTOL v1.7
Polypodiaceae	Moranopteris gradata (Baker) R. Y. Hirai & J. Prado	trnL-trnF	JF514043	FTOL v1.7
Polypodiaceae	Moranopteris grisebachii (C. Chr.) R. Y. Hirai & J. Prado	rbcL	JF514008	FTOL v1.7
Polypodiaceae	Moranopteris grisebachii (C. Chr.) R. Y. Hirai & J. Prado	trnL-trnF	JF514041	FTOL v1.7
Polypodiaceae	Moranopteris hyalina (Maxon) R. Y. Hirai & J. Prado	atpB	JF514070	FTOL v1.7
Polypodiaceae	Moranopteris hyalina (Maxon) R. Y. Hirai & J. Prado	rbcL	JF514001	FTOL v1.7
Polypodiaceae	Moranopteris hyalina (Maxon) R. Y. Hirai & J. Prado	rps4-trnS	KM106157	FTOL v1.7
Polypodiaceae	Moranopteris hyalina (Maxon) R. Y. Hirai & J. Prado	trnL-trnF	JF514034	FTOL v1.7
Polypodiaceae	Moranopteris inaccessa Sundue & Sylvester	atpB	KP050355	FTOL v1.7
Polypodiaceae	Moranopteris inaccessa Sundue & Sylvester	rbcL	KP027642	FTOL v1.7
Polypodiaceae	Moranopteris inaccessa Sundue & Sylvester	trnL-trnF	KP050357	FTOL v1.7
Polypodiaceae	Moranopteris longisetosa (Hook.) R. Y. Hirai & J. Prado	atpB	JF514072	FTOL v1.7
Polypodiaceae	Moranopteris longisetosa (Hook.) R. Y. Hirai & J. Prado	rbcL	JF514003	FTOL v1.7
Polypodiaceae	Moranopteris longisetosa (Hook.) R. Y. Hirai & J. Prado	rps4-trnS	KM106158	FTOL v1.7
Polypodiaceae	Moranopteris longisetosa (Hook.) R. Y. Hirai & J. Prado	trnL-trnF	JF514036	FTOL v1.7
Polypodiaceae	Moranopteris microlepis (Rosenst.) R. Y. Hirai & J. Pra	atpB	JF514056	FTOL v1.7
Polypodiaceae	Moranopteris microlepis (Rosenst.) R. Y. Hirai & J. Pra	rbcL	JF513997	FTOL v1.7
Polypodiaceae	Moranopteris microlepis (Rosenst.) R. Y. Hirai & J. Pra	trnL-trnF	JF514031	FTOL v1.7
Polypodiaceae	Moranopteris nana (Fée) R. Y. Hirai & J. Prado	rbcL	JF513990	FTOL v1.7
Polypodiaceae	Moranopteris perpusilla (Maxon) R. Y. Hirai & J. Prado	atpB	JF514079	FTOL v1.7
Polypodiaceae	Moranopteris perpusilla (Maxon) R. Y. Hirai & J. Prado	rbcL	JF514011	FTOL v1.7
Polypodiaceae	Moranopteris perpusilla (Maxon) R. Y. Hirai & J. Prado	trnL-trnF	JF514045	FTOL v1.7
Polypodiaceae	Moranopteris plicata (A. R. Sm.) R. Y. Hirai & J. Prado	atpB	JF514074	FTOL v1.7
Polypodiaceae	Moranopteris plicata (A. R. Sm.) R. Y. Hirai & J. Prado	rbcL	JF514005	FTOL v1.7
Polypodiaceae	Moranopteris plicata (A. R. Sm.) R. Y. Hirai & J. Prado	rps4-trnS	KM106159	FTOL v1.7
Polypodiaceae	Moranopteris plicata (A. R. Sm.) R. Y. Hirai & J. Prado	trnL-trnF	JF514038	FTOL v1.7
Polypodiaceae	Moranopteris serricula (Fée) R. Y. Hirai & J. Prado	atpB	JF514085	FTOL v1.7
Polypodiaceae	Moranopteris serricula (Fée) R. Y. Hirai & J. Prado	rbcL	JF514017	FTOL v1.7
Polypodiaceae	Moranopteris serricula (Fée) R. Y. Hirai & J. Prado	trnL-trnF	JF514052	FTOL v1.7
Polypodiaceae	Moranopteris setosa (Kaulf.) R. Y. Hirai & J. Prado	atpB	JF514080	FTOL v1.7
Polypodiaceae	Moranopteris setosa (Kaulf.) R. Y. Hirai & J. Prado	rbcL	JF514012	FTOL v1.7
Polypodiaceae	Moranopteris setosa (Kaulf.) R. Y. Hirai & J. Prado	trnL-trnF	JF514046	FTOL v1.7
Polypodiaceae	Moranopteris setulosa (Rosenst.) A. Rojas	rbcL	MK319143	FTOL v1.7
Polypodiaceae	Moranopteris setulosa (Rosenst.) A. Rojas	trnL-trnF	MK319025	FTOL v1.7
Polypodiaceae	Moranopteris sherringii (Baker) R. Y. Hirai & J. Prado	rbcL	KP027643	FTOL v1.7
Polypodiaceae	Moranopteris sherringii (Baker) R. Y. Hirai & J. Prado	trnL-trnF	KP050356	FTOL v1.7
Polypodiaceae	Moranopteris taenifolia (Jenman) R. Y. Hirai & J. Prado	atpB	JF514083	FTOL v1.7
Polypodiaceae	Moranopteris taenifolia (Jenman) R. Y. Hirai & J. Prado	rbcL	JF514015	FTOL v1.7
Polypodiaceae	Moranopteris taenifolia (Jenman) R. Y. Hirai & J. Prado	trnL-trnF	JF514050	FTOL v1.7
Polypodiaceae	Moranopteris trichomanoides (Sw.) R. Y. Hirai & J. Pra	atpB	JF514063	FTOL v1.7
Polypodiaceae	Moranopteris trichomanoides (Sw.) R. Y. Hirai & J. Pra	rbcL	JF513993	FTOL v1.7
Polypodiaceae	Moranopteris trichomanoides (Sw.) R. Y. Hirai & J. Pra	trnL-trnF	JF514027	FTOL v1.7
Polypodiaceae	Moranopteris truncicola (Klotzsch) R. Y. Hirai & J. Prad	atpB	JF514084	FTOL v1.7
Polypodiaceae	Moranopteris truncicola (Klotzsch) R. Y. Hirai & J. Prad	rbcL	JF514016	FTOL v1.7
Polypodiaceae	Moranopteris truncicola (Klotzsch) R. Y. Hirai & J. Prad	rps4-trnS	KM106160	FTOL v1.7
Polypodiaceae	Moranopteris truncicola (Klotzsch) R. Y. Hirai & J. Prad	trnL-trnF	JF514051	FTOL v1.7
Polypodiaceae	Moranopteris williamsii (Maxon) R. Y. Hirai & J. Prado	atpB	JF514069	FTOL v1.7
Polypodiaceae	Moranopteris williamsii (Maxon) R. Y. Hirai & J. Prado	rbcL	JF514000	FTOL v1.7
Polypodiaceae	Moranopteris williamsii (Maxon) R. Y. Hirai & J. Prado	trnL-trnF	JF514033	FTOL v1.7
Polypodiaceae	Moranopteris zurquina (Copel.) R. Y. Hirai & J. Prado	atpB	AY459492	FTOL v1.7
Polypodiaceae	Moranopteris zurquina (Copel.) R. Y. Hirai & J. Prado	rbcL	AY460659	FTOL v1.7
Polypodiaceae	Mycopteris alsopteris (C. V. Morton) Sundue	atpB	AY459500	FTOL v1.7
Polypodiaceae	Mycopteris alsopteris (C. V. Morton) Sundue	rbcL	AY460667	FTOL v1.7
Polypodiaceae	Mycopteris amphidasyon (Mett.) Sundue	atpB	GU476759	FTOL v1.7
Polypodiaceae	Mycopteris amphidasyon (Mett.) Sundue	rbcL	GU476922	FTOL v1.7
Polypodiaceae	Mycopteris amphidasyon (Mett.) Sundue	rps4-trnS	KM106161	FTOL v1.7
Polypodiaceae	Mycopteris amphidasyon (Mett.) Sundue	trnL-trnF	GU476638	FTOL v1.7
Polypodiaceae	Mycopteris attenuatissima (Copel.) Sundue	atpB	GU476801	FTOL v1.7
Polypodiaceae	Mycopteris attenuatissima (Copel.) Sundue	rbcL	GU476866	FTOL v1.7
Polypodiaceae	Mycopteris attenuatissima (Copel.) Sundue	rps4-trnS	GU387121	FTOL v1.7
Polypodiaceae	Mycopteris attenuatissima (Copel.) Sundue	trnL-trnF	GU476705	FTOL v1.7
Polypodiaceae	Mycopteris costaricensis (Rosenst.) Sundue	rbcL	MW138273	FTOL v1.7
Polypodiaceae	Mycopteris cretata (Maxon) Sundue	rbcL	GU476823	FTOL v1.7
Polypodiaceae	Mycopteris cretata (Maxon) Sundue	trnL-trnF	GU476713	FTOL v1.7
Polypodiaceae	Mycopteris leucosticta (J. Sm.) Sundue	atpB	GU476811	FTOL v1.7
Polypodiaceae	Mycopteris leucosticta (J. Sm.) Sundue	rbcL	GU476848	FTOL v1.7
Polypodiaceae	Mycopteris leucosticta (J. Sm.) Sundue	rps4-trnS	KM106162	FTOL v1.7
Polypodiaceae	Mycopteris leucosticta (J. Sm.) Sundue	trnL-trnF	GU476720	FTOL v1.7
Polypodiaceae	Mycopteris longicaulis (Sundue & M. Kessler) Sundue	atpB	GU476813	FTOL v1.7
Polypodiaceae	Mycopteris longicaulis (Sundue & M. Kessler) Sundue	rbcL	GU476840	FTOL v1.7
Polypodiaceae	Mycopteris longicaulis (Sundue & M. Kessler) Sundue	rps4-trnS	KM106163	FTOL v1.7
Polypodiaceae	Mycopteris longicaulis (Sundue & M. Kessler) Sundue	trnL-trnF	GU476724	FTOL v1.7
Polypodiaceae	Mycopteris longipilosa Sundue	atpB	GU476814	FTOL v1.7
Polypodiaceae	Mycopteris longipilosa Sundue	rbcL	GU476861	FTOL v1.7
Polypodiaceae	Mycopteris longipilosa Sundue	rps4-trnS	KM106164	FTOL v1.7
Polypodiaceae	Mycopteris longipilosa Sundue	trnL-trnF	GU476725	FTOL v1.7
Polypodiaceae	Mycopteris praeceps (Sundue & M. Kessler) Sundue	atpB	GU476817	FTOL v1.7
Polypodiaceae	Mycopteris praeceps (Sundue & M. Kessler) Sundue	rbcL	GU476839	FTOL v1.7
Polypodiaceae	Mycopteris praeceps (Sundue & M. Kessler) Sundue	rps4-trnS	KM106165	FTOL v1.7
Polypodiaceae	Mycopteris praeceps (Sundue & M. Kessler) Sundue	trnL-trnF	GU476734	FTOL v1.7
Polypodiaceae	Mycopteris semihirsuta (Klotzsch) Sundue	atpB	GU476818	FTOL v1.7
Polypodiaceae	Mycopteris semihirsuta (Klotzsch) Sundue	rbcL	GU476894	FTOL v1.7
Polypodiaceae	Mycopteris semihirsuta (Klotzsch) Sundue	trnL-trnF	GU476735	FTOL v1.7
Polypodiaceae	Mycopteris subtilis (Kunze ex Klotzsch) Sundue	atpB	GU376643	FTOL v1.7
Polypodiaceae	Mycopteris subtilis (Kunze ex Klotzsch) Sundue	rbcL	GU386984	FTOL v1.7
Polypodiaceae	Mycopteris subtilis (Kunze ex Klotzsch) Sundue	rps4-trnS	GU387128	FTOL v1.7
Polypodiaceae	Mycopteris subtilis (Kunze ex Klotzsch) Sundue	trnL-trnF	GU387288	FTOL v1.7
Polypodiaceae	Mycopteris taxifolia (L.) Sundue	atpB	GU476800	FTOL v1.7
Polypodiaceae	Mycopteris taxifolia (L.) Sundue	rbcL	GU476914	FTOL v1.7
Polypodiaceae	Mycopteris taxifolia (L.) Sundue	rps4-trnS	KM106167	FTOL v1.7
Polypodiaceae	Mycopteris taxifolia (L.) Sundue	trnL-trnF	GU476699	FTOL v1.7
Polypodiaceae	Mycopteris zeledoniana (Lellinger) Sundue	rbcL	GU476910	FTOL v1.7
Polypodiaceae	Mycopteris zeledoniana (Lellinger) Sundue	rps4-trnS	GU387129	FTOL v1.7
Polypodiaceae	Nanogrammitis holophlebia (Baker) Parris, Li Bing Zha	atpB	KY711808	FTOL v1.7
Polypodiaceae	Nanogrammitis holophlebia (Baker) Parris, Li Bing Zha	rbcL	KY711983	FTOL v1.7
Polypodiaceae	Nanogrammitis holophlebia (Baker) Parris, Li Bing Zha	rps4-trnS	KY712306	FTOL v1.7
Polypodiaceae	Nanogrammitis holophlebia (Baker) Parris, Li Bing Zha	trnL-trnF	KY711632	FTOL v1.7
Polypodiaceae	Nanogrammitis obtusa (Willd. ex Kaulf.) Parris, Li Bing	atpB	KY711818	FTOL v1.7
Polypodiaceae	Nanogrammitis obtusa (Willd. ex Kaulf.) Parris, Li Bing	rbcL	KF992493	FTOL v1.7
Polypodiaceae	Nanogrammitis obtusa (Willd. ex Kaulf.) Parris, Li Bing	rps4-trnS	KY712315	FTOL v1.7
Polypodiaceae	Nanogrammitis obtusa (Willd. ex Kaulf.) Parris, Li Bing	trnL-trnF	KY711641	FTOL v1.7
Polypodiaceae	Nanogrammitis pellucidovenosa (Bonap.) Parris, Li Bin	atpB	KY711820	FTOL v1.7
Polypodiaceae	Nanogrammitis pellucidovenosa (Bonap.) Parris, Li Bin	rbcL	KY711996	FTOL v1.7
Polypodiaceae	Nanogrammitis pellucidovenosa (Bonap.) Parris, Li Bin	trnL-trnF	KY711645	FTOL v1.7
Polypodiaceae	Nanogrammitis pervillei (Mett. ex Kuhn) Parris, Li Bing	atpB	KY711887	FTOL v1.7
Polypodiaceae	Nanogrammitis pervillei (Mett. ex Kuhn) Parris, Li Bing	rbcL	KY712061	FTOL v1.7
Polypodiaceae	Nanogrammitis pervillei (Mett. ex Kuhn) Parris, Li Bing	rps4-trnS	KY712379	FTOL v1.7
Polypodiaceae	Nanogrammitis pervillei (Mett. ex Kuhn) Parris, Li Bing	trnL-trnF	KY711707	FTOL v1.7
Polypodiaceae	Nanogrammitis synsora (Baker) Parris, Li Bing Zhang,	atpB	KY711826	FTOL v1.7
Polypodiaceae	Nanogrammitis synsora (Baker) Parris, Li Bing Zhang,	rbcL	KY712006	FTOL v1.7
Polypodiaceae	Nanogrammitis synsora (Baker) Parris, Li Bing Zhang,	trnL-trnF	KY711654	FTOL v1.7
Polypodiaceae	Niphidium albopunctatissimum (J. Sm.) Lellinger	rbcL	MF317952	FTOL v1.7
Polypodiaceae	Niphidium albopunctatissimum (J. Sm.) Lellinger	rps4-trnS	MF318187	FTOL v1.7
Polypodiaceae	Niphidium albopunctatissimum (J. Sm.) Lellinger	trnL-trnF	MF318432	FTOL v1.7
Polypodiaceae	Niphidium crassifolium (L.) Lellinger	plastome	MW876344	FTOL v1.7
Polypodiaceae	Niphidium longifolium (Cav.) C. V. Morton & Lellinger	rbcL	KF667663	FTOL v1.7
Polypodiaceae	Niphidium longifolium (Cav.) C. V. Morton & Lellinger	rps4-trnS	KF667578	FTOL v1.7
Polypodiaceae	Niphidium longifolium (Cav.) C. V. Morton & Lellinger	trnL-trnF	MF318434	FTOL v1.7
Polypodiaceae	Niphidium nidulare (Rosenst.) Lellinger	rbcL	MF317988	FTOL v1.7

Polypodiaceae	Niphidium nidulare (Rosenst.) Lellinger	rps4-trnS	MF318189	FTOL v1.7
Polypodiaceae	Niphidium nidulare (Rosenst.) Lellinger	trnL-trnF	MF318435	FTOL v1.7
Polypodiaceae	Notogrammitis angustifolia (Jacq.) Parris	atpB	KM218824	FTOL v1.7
Polypodiaceae	Notogrammitis angustifolia (Jacq.) Parris	rbcL	KM218770	FTOL v1.7
Polypodiaceae	Notogrammitis angustifolia (Jacq.) Parris	rps4-trnS	KM106168	FTOL v1.7
Polypodiaceae	Notogrammitis angustifolia (Jacq.) Parris	trnL-trnF	KM106074	FTOL v1.7
Polypodiaceae	Notogrammitis billardierei (Willd.) Parris	atpB	AY459469	FTOL v1.7
Polypodiaceae	Notogrammitis billardierei (Willd.) Parris	rbcL	AY460637	FTOL v1.7
Polypodiaceae	Notogrammitis ciliata (Colenso) Parris	atpB	AY459470	FTOL v1.7
Polypodiaceae	Notogrammitis ciliata (Colenso) Parris	rbcL	AY460638	FTOL v1.7
Polypodiaceae	Notogrammitis crassior (Kirk) Parris	atpB	KY711810	FTOL v1.7
Polypodiaceae	Notogrammitis crassior (Kirk) Parris	rbcL	KY711985	FTOL v1.7
Polypodiaceae	Notogrammitis crassior (Kirk) Parris	rps4-trnS	KY712307	FTOL v1.7
Polypodiaceae	Notogrammitis crassior (Kirk) Parris	trnL-trnF	KY711634	FTOL v1.7
Polypodiaceae	Notogrammitis givenii (Parris) Parris	atpB	JX499242	FTOL v1.7
Polypodiaceae	Notogrammitis givenii (Parris) Parris	rbcL	JQ904079	FTOL v1.7
Polypodiaceae	Notogrammitis givenii (Parris) Parris	trnL-trnF	JQ904112	FTOL v1.7
Polypodiaceae	Notogrammitis heterophylla (Labill.) Parris	atpB	JX499237	FTOL v1.7
Polypodiaceae	Notogrammitis heterophylla (Labill.) Parris	rbcL	AY460628	FTOL v1.7
Polypodiaceae	Notogrammitis heterophylla (Labill.) Parris	trnL-trnF	JQ904099	FTOL v1.7
Polypodiaceae	Notogrammitis patagonica (C. Chr.) Parris	rbcL	JQ904088	FTOL v1.7
Polypodiaceae	Notogrammitis patagonica (C. Chr.) Parris	trnL-trnF	JQ904075	FTOL v1.7
Polypodiaceae	Notogrammitis pseudociliata (Parris) Parris	atpB	AY459477	FTOL v1.7
Polypodiaceae	Notogrammitis pseudociliata (Parris) Parris	rbcL	AY460645	FTOL v1.7
Polypodiaceae	Notogrammitis rawlingsii (Parris) Parris	atpB	JX499240	FTOL v1.7
Polypodiaceae	Notogrammitis rawlingsii (Parris) Parris	rbcL	JQ904086	FTOL v1.7
Polypodiaceae	Notogrammitis rawlingsii (Parris) Parris	trnL-trnF	JQ904118	FTOL v1.7
Polypodiaceae	Notogrammitis rigida (Hombr.) Parris	atpB	JX499238	FTOL v1.7
Polypodiaceae	Notogrammitis rigida (Hombr.) Parris	rbcL	JQ904082	FTOL v1.7
Polypodiaceae	Notogrammitis rigida (Hombr.) Parris	trnL-trnF	JQ904121	FTOL v1.7
Polypodiaceae	Oreogrammitis adspersa (Blume) Parris	plastome	MT130563	FTOL v1.7
Polypodiaceae	Oreogrammitis archboldii (C. Chr.) Parris	rbcL	OK514682	FTOL v1.7
Polypodiaceae	Oreogrammitis baldwinii (Baker) Parris	atpB	EF178633	FTOL v1.7
Polypodiaceae	Oreogrammitis baldwinii (Baker) Parris	rbcL	EF178616	FTOL v1.7
Polypodiaceae	Oreogrammitis baldwinii (Baker) Parris	trnL-trnF	EF178649	FTOL v1.7
Polypodiaceae	Oreogrammitis caespitosa (Blume) Parris	rbcL	OK514670	FTOL v1.7
Polypodiaceae	Oreogrammitis cheesemanii (Parris) Parris & Sundue	atpB	KM218861	FTOL v1.7
Polypodiaceae	Oreogrammitis cheesemanii (Parris) Parris & Sundue	rps4-trnS	KM106183	FTOL v1.7
Polypodiaceae	Oreogrammitis cheesemanii (Parris) Parris & Sundue	trnL-trnF	KM106087	FTOL v1.7
Polypodiaceae	Oreogrammitis conformis (Brack.) Parris	rbcL	MT657610	FTOL v1.7
Polypodiaceae	Oreogrammitis congener (Blume) Parris	atpB	KM218845	FTOL v1.7
Polypodiaceae	Oreogrammitis congener (Blume) Parris	rbcL	KM218762	FTOL v1.7
Polypodiaceae	Oreogrammitis congener (Blume) Parris	rps4-trnS	KM106172	FTOL v1.7
Polypodiaceae	Oreogrammitis congener (Blume) Parris	trnL-trnF	KY711570	FTOL v1.7
Polypodiaceae	Oreogrammitis conjunctisora (Baker) Parris & Sundue	atpB	AY459514	FTOL v1.7
Polypodiaceae	Oreogrammitis conjunctisora (Baker) Parris & Sundue	rbcL	AY460680	FTOL v1.7
Polypodiaceae	Oreogrammitis ctenoidea (Brause) Parris & Sundue	rbcL	OK514689	FTOL v1.7
Polypodiaceae	Oreogrammitis curtipila (Parris) Parris	rps4-trnS	MT648255	FTOL v1.7
Polypodiaceae	Oreogrammitis curtisii (Baker) Parris & Sundue	rbcL	KM218758	FTOL v1.7
Polypodiaceae	Oreogrammitis curtisii (Baker) Parris & Sundue	rps4-trnS	KM106193	FTOL v1.7
Polypodiaceae	Oreogrammitis curtisii (Baker) Parris & Sundue	trnL-trnF	KM106093	FTOL v1.7
Polypodiaceae	Oreogrammitis dolichosora (Copel.) Parris	atpB	EF178635	FTOL v1.7
Polypodiaceae	Oreogrammitis dolichosora (Copel.) Parris	rbcL	EF178618	FTOL v1.7
Polypodiaceae	Oreogrammitis dorsipila (Christ) Parris	rbcL	AB575257	FTOL v1.7
Polypodiaceae	Oreogrammitis fasciata (Blume) Parris	rbcL	OK514675	FTOL v1.7
Polypodiaceae	Oreogrammitis fenicis (Copel.) Parris	atpB	OQ702686	FTOL v1.7
Polypodiaceae	Oreogrammitis fenicis (Copel.) Parris	rps4	OQ717223	FTOL v1.7
Polypodiaceae	Oreogrammitis fenicis (Copel.) Parris	rps4-trnS	OQ717223	FTOL v1.7
Polypodiaceae	Oreogrammitis fenicis (Copel.) Parris	trnL-trnF	OQ717130	FTOL v1.7
Polypodiaceae	Oreogrammitis flavovirens (Alston) Parris	rbcL	MH826903	FTOL v1.7
Polypodiaceae	Oreogrammitis forbesiana (W. H. Wagner) Parris	atpB	AY459472	FTOL v1.7
Polypodiaceae	Oreogrammitis forbesiana (W. H. Wagner) Parris	rbcL	AY460640	FTOL v1.7
Polypodiaceae	Oreogrammitis frigida (Ridl.) Parris	rbcL	OK514681	FTOL v1.7
Polypodiaceae	Oreogrammitis graminella (C. Chr.) Parris & Sundue	rbcL	OK514662	FTOL v1.7
Polypodiaceae	Oreogrammitis halconensis (Copel.) Parris & Sundue	atpB	KM218844	FTOL v1.7
Polypodiaceae	Oreogrammitis halconensis (Copel.) Parris & Sundue	rps4-trnS	KM106194	FTOL v1.7
Polypodiaceae	Oreogrammitis halconensis (Copel.) Parris & Sundue	trnL-trnF	KM106094	FTOL v1.7
Polypodiaceae	Oreogrammitis havilandii (Baker) Parris & Sundue	atpB	EF178636	FTOL v1.7
Polypodiaceae	Oreogrammitis havilandii (Baker) Parris & Sundue	rbcL	EF178619	FTOL v1.7
Polypodiaceae	Oreogrammitis havilandii (Baker) Parris & Sundue	trnL-trnF	EF178652	FTOL v1.7
Polypodiaceae	Oreogrammitis hirtella (Blume) Parris & Sundue	atpB	OQ702736	FTOL v1.7
Polypodiaceae	Oreogrammitis hirtella (Blume) Parris & Sundue	rbcL	KM218772	FTOL v1.7
Polypodiaceae	Oreogrammitis hirtella (Blume) Parris & Sundue	rps4	OQ717285	FTOL v1.7
Polypodiaceae	Oreogrammitis hirtella (Blume) Parris & Sundue	rps4-trnS	OQ717285	FTOL v1.7
Polypodiaceae	Oreogrammitis hirtella (Blume) Parris & Sundue	trnL-trnF	OQ717131	FTOL v1.7
Polypodiaceae	Oreogrammitis hirtelloides (Copel.) Parris & Sundue	atpB	KY711841	FTOL v1.7
Polypodiaceae	Oreogrammitis hirtelloides (Copel.) Parris & Sundue	rbcL	KY712017	FTOL v1.7
Polypodiaceae	Oreogrammitis hirtelloides (Copel.) Parris & Sundue	rps4-trnS	KY712333	FTOL v1.7
Polypodiaceae	Oreogrammitis hirtelloides (Copel.) Parris & Sundue	trnL-trnF	KY711666	FTOL v1.7
Polypodiaceae	Oreogrammitis holttumii (Copel.) Parris & Sundue	atpB	KM218856	FTOL v1.7
Polypodiaceae	Oreogrammitis holttumii (Copel.) Parris & Sundue	rbcL	KM218771	FTOL v1.7
Polypodiaceae	Oreogrammitis holttumii (Copel.) Parris & Sundue	rps4-trnS	KM106184	FTOL v1.7
Polypodiaceae	Oreogrammitis hookeri (Brack.) Parris	atpB	AY459473	FTOL v1.7
Polypodiaceae	Oreogrammitis hookeri (Brack.) Parris	rbcL	AY460642	FTOL v1.7
Polypodiaceae	Oreogrammitis jagoriana (Mett. ex Kuhn) Parris & Sun	atpB	EF178639	FTOL v1.7
Polypodiaceae	Oreogrammitis jagoriana (Mett. ex Kuhn) Parris & Sun	rbcL	EF178622	FTOL v1.7
Polypodiaceae	Oreogrammitis jagoriana (Mett. ex Kuhn) Parris & Sun	trnL-trnF	EF178656	FTOL v1.7
Polypodiaceae	Oreogrammitis kinabaluensis (Copel.) Parris & Sundue	atpB	KM218855	FTOL v1.7
Polypodiaceae	Oreogrammitis kinabaluensis (Copel.) Parris & Sundue	rbcL	KM218773	FTOL v1.7
Polypodiaceae	Oreogrammitis kinabaluensis (Copel.) Parris & Sundue	rps4-trnS	KM106185	FTOL v1.7
Polypodiaceae	Oreogrammitis kinabaluensis (Copel.) Parris & Sundue	trnL-trnF	KM106088	FTOL v1.7
Polypodiaceae	Oreogrammitis knutsfordiana (Baker) Parris	atpB	AY459474	FTOL v1.7
Polypodiaceae	Oreogrammitis knutsfordiana (Baker) Parris	rbcL	AY362342	FTOL v1.7
Polypodiaceae	Oreogrammitis ligulata (Baker) Parris & Sundue	rbcL	MT657606	FTOL v1.7
Polypodiaceae	Oreogrammitis locellata (Baker) Parris	rbcL	MT657592	FTOL v1.7
Polypodiaceae	Oreogrammitis locellata (Baker) Parris	rps4-trnS	KM106173	FTOL v1.7
Polypodiaceae	Oreogrammitis locellata (Baker) Parris	trnL-trnF	KM106079	FTOL v1.7
Polypodiaceae	Oreogrammitis longiceps (Rosenst.) Parris	rbcL	OK514691	FTOL v1.7
Polypodiaceae	Oreogrammitis malayensis Parris	rbcL	OK514667	FTOL v1.7
Polypodiaceae	Oreogrammitis marivelesensis (Copel.) Parris	rbcL	OK514661	FTOL v1.7
Polypodiaceae	Oreogrammitis medialis (Baker) Parris	rps4-trnS	KY712397	FTOL v1.7
Polypodiaceae	Oreogrammitis medialis (Baker) Parris	trnL-trnF	KY711730	FTOL v1.7
Polypodiaceae	Oreogrammitis mollipila (Baker) Parris	rbcL	OK514686	FTOL v1.7
Polypodiaceae	Oreogrammitis multifolia (Copel.) Parris & Sundue	rps4	AY362689	FTOL v1.7
Polypodiaceae	Oreogrammitis nana (Fée) Parris	rbcL	OK514693	FTOL v1.7
Polypodiaceae	Oreogrammitis neocaledonica (Copel.) Parris	atpB	KY711843	FTOL v1.7
Polypodiaceae	Oreogrammitis neocaledonica (Copel.) Parris	rbcL	KY712019	FTOL v1.7
Polypodiaceae	Oreogrammitis neocaledonica (Copel.) Parris	rps4-trnS	KY712335	FTOL v1.7
Polypodiaceae	Oreogrammitis neocaledonica (Copel.) Parris	trnL-trnF	KY711668	FTOL v1.7
Polypodiaceae	Oreogrammitis padangensis (Baker) Parris	atpB	EF178642	FTOL v1.7
Polypodiaceae	Oreogrammitis padangensis (Baker) Parris	rbcL	EF178625	FTOL v1.7
Polypodiaceae	Oreogrammitis padangensis (Baker) Parris	trnL-trnF	EF178660	FTOL v1.7
Polypodiaceae	Oreogrammitis parva (Brause) Parris & Sundue	atpB	AY459476	FTOL v1.7
Polypodiaceae	Oreogrammitis parva (Brause) Parris & Sundue	rbcL	AY460644	FTOL v1.7
Polypodiaceae	Oreogrammitis parvula Parris	rbcL	OK514659	FTOL v1.7
Polypodiaceae	Oreogrammitis pilosiuscula (Blume) Parris	rps4-trnS	MT648248	FTOL v1.7
Polypodiaceae	Oreogrammitis pleurogrammoides (Rosenst.) Parris	rbcL	OK514663	FTOL v1.7
Polypodiaceae	Oreogrammitis raiateensis (J. W. Moore) Parris	rbcL	KY099840	FTOL v1.7
Polypodiaceae	Oreogrammitis reinwardtii (Blume) Parris	plastome	MW876347	FTOL v1.7
Polypodiaceae	Oreogrammitis reinwardtioides (Copel.) Parris	atpB	KM218847	FTOL v1.7
Polypodiaceae	Oreogrammitis reinwardtioides (Copel.) Parris	rbcL	KM218756	FTOL v1.7
Polypodiaceae	Oreogrammitis reinwardtioides (Copel.) Parris	trnL-trnF	KM106080	FTOL v1.7
Polypodiaceae	Oreogrammitis scabristipes (Baker) Parris	rbcL	OK514687	FTOL v1.7
Polypodiaceae	Oreogrammitis scleroglossoides (Copel.) Parris	rbcL	MT657612	FTOL v1.7
Polypodiaceae	Oreogrammitis setigera (Blume) T. C. Hsu	atpB	OQ702701	FTOL v1.7
Polypodiaceae	Oreogrammitis setigera (Blume) T. C. Hsu	rbcL	KM218759	FTOL v1.7
Polypodiaceae	Oreogrammitis setigera (Blume) T. C. Hsu	rps4	OQ717281	FTOL v1.7
Polypodiaceae	Oreogrammitis setigera (Blume) T. C. Hsu	rps4-trnS	OQ717281	FTOL v1.7
Polypodiaceae	Oreogrammitis setigera (Blume) T. C. Hsu	trnL-trnF	KM106089	FTOL v1.7
Polypodiaceae	Oreogrammitis sinohirtella Parris	rbcL	OK514666	FTOL v1.7
Polypodiaceae	Oreogrammitis subevenosa (Baker) Parris	rbcL	OK514692	FTOL v1.7
Polypodiaceae	Oreogrammitis subevenosa (Baker) Parris	rps4-trnS	MT648269	FTOL v1.7
Polypodiaceae	Oreogrammitis subpinnatifida (Blume) Parris & Sundu	rbcL	OK514683	FTOL v1.7
Polypodiaceae	Oreogrammitis subspathulata (Brack.) Parris	rbcL	KY099841	FTOL v1.7
Polypodiaceae	Oreogrammitis sumatrana (Baker) Parris	rbcL	OK514685	FTOL v1.7
Polypodiaceae	Oreogrammitis taiwanensis (Parris & Ralf Knapp) T. C.	atpB	OQ702687	FTOL v1.7
Polypodiaceae	Oreogrammitis taiwanensis (Parris & Ralf Knapp) T. C.	rbcL	OK514665	FTOL v1.7
Polypodiaceae	Oreogrammitis taiwanensis (Parris & Ralf Knapp) T. C.	rps4	OQ717198	FTOL v1.7
Polypodiaceae	Oreogrammitis taiwanensis (Parris & Ralf Knapp) T. C.	rps4-trnS	OQ717198	FTOL v1.7
Polypodiaceae	Oreogrammitis tenuisecta (Blume) T. C. Hsu	plastome	OQ968265	FTOL v1.7
Polypodiaceae	Oreogrammitis torricelliana (Brause) Parris	rbcL	OK514658	FTOL v1.7
Polypodiaceae	Oreogrammitis tuberculata (Parris) Parris & Sundue	rbcL	OK514688	FTOL v1.7
Polypodiaceae	Oreogrammitis tuyamae (H. Ohba) Parris	rbcL	AB575258	FTOL v1.7
Polypodiaceae	Oreogrammitis uapensis (E. D. Br.) Parris	rbcL	MT216076	FTOL v1.7
Polypodiaceae	Oreogrammitis uapensis (E. D. Br.) Parris	rps4-trnS	MT648270	FTOL v1.7
Polypodiaceae	Oreogrammitis uapensis (E. D. Br.) Parris	trnL-trnF	KM106070	FTOL v1.7
Polypodiaceae	Oreogrammitis wurunuran (Parris) Parris	atpB	KM218843	FTOL v1.7
Polypodiaceae	Oreogrammitis wurunuran (Parris) Parris	rbcL	KM218761	FTOL v1.7
Polypodiaceae	Oreogrammitis wurunuran (Parris) Parris	rps4-trnS	KM106175	FTOL v1.7
Polypodiaceae	Oreogrammitis yoderi (Copel.) Parris & Sundue	rbcL	OK514673	FTOL v1.7
Polypodiaceae	Parrisia parietina (Klotzsch) Shalisko & Sundue	atpA	EF463812	FTOL v1.7
Polypodiaceae	Parrisia parietina (Klotzsch) Shalisko & Sundue	atpB	EF463494	FTOL v1.7
Polypodiaceae	Parrisia parietina (Klotzsch) Shalisko & Sundue	rbcL	EF463248	FTOL v1.7
Polypodiaceae	Pecluma alfredii (Rosenst.) M. G. Price	rbcL	AY096206	FTOL v1.7
Polypodiaceae	Pecluma alfredii (Rosenst.) M. G. Price	rps4	AY096232	FTOL v1.7
Polypodiaceae	Pecluma alfredii (Rosenst.) M. G. Price	rps4-trnS	AY096232	FTOL v1.7
Polypodiaceae	Pecluma barituensis O. G. Martínez & de la Sota	rbcL	KT780725	FTOL v1.7
Polypodiaceae	Pecluma barituensis O. G. Martínez & de la Sota	rps4	KT794100	FTOL v1.7
Polypodiaceae	Pecluma barituensis O. G. Martínez & de la Sota	rps4-trnS	KT794100	FTOL v1.7
Polypodiaceae	Pecluma barituensis O. G. Martínez & de la Sota	trnL-trnF	KT780782	FTOL v1.7
Polypodiaceae	Pecluma bourgeauana (E. Fourn.) L. A. Triana	rbcL	KT780726	FTOL v1.7
Polypodiaceae	Pecluma bourgeauana (E. Fourn.) L. A. Triana	rps4	KT794101	FTOL v1.7
Polypodiaceae	Pecluma bourgeauana (E. Fourn.) L. A. Triana	rps4-trnS	KT794101	FTOL v1.7
Polypodiaceae	Pecluma bourgeauana (E. Fourn.) L. A. Triana	trnL-trnF	KT780783	FTOL v1.7
Polypodiaceae	Pecluma camptophyllaria (Fée) M. G. Price	rbcL	KT780727	FTOL v1.7
Polypodiaceae	Pecluma camptophyllaria (Fée) M. G. Price	rps4	KT794102	FTOL v1.7
Polypodiaceae	Pecluma camptophyllaria (Fée) M. G. Price	rps4-trnS	KT794102	FTOL v1.7
Polypodiaceae	Pecluma camptophyllaria (Fée) M. G. Price	trnL-trnF	KT780784	FTOL v1.7
Polypodiaceae	Pecluma chnoophora (Kunze) Salino & F. C. Assis	rbcL	KT780728	FTOL v1.7
Polypodiaceae	Pecluma chnoophora (Kunze) Salino & F. C. Assis	rps4	KT794103	FTOL v1.7
Polypodiaceae	Pecluma chnoophora (Kunze) Salino & F. C. Assis	rps4-trnS	KT794103	FTOL v1.7
Polypodiaceae	Pecluma chnoophora (Kunze) Salino & F. C. Assis	trnL-trnF	KT780785	FTOL v1.7
Polypodiaceae	Pecluma choquetangensis (Rosenst.) M. G. Price	rbcL	KT780729	FTOL v1.7
Polypodiaceae	Pecluma choquetangensis (Rosenst.) M. G. Price	rps4	KT794104	FTOL v1.7
Polypodiaceae	Pecluma choquetangensis (Rosenst.) M. G. Price	rps4-trnS	KT794104	FTOL v1.7
Polypodiaceae	Pecluma choquetangensis (Rosenst.) M. G. Price	trnL-trnF	KT780786	FTOL v1.7
Polypodiaceae	Pecluma consimilis (D. C. Eaton ex Mett.) M. G. Price	rbcL	KT780730	FTOL v1.7
Polypodiaceae	Pecluma consimilis (D. C. Eaton ex Mett.) M. G. Price	rps4	KT794105	FTOL v1.7
Polypodiaceae	Pecluma consimilis (D. C. Eaton ex Mett.) M. G. Price	rps4-trnS	KT794105	FTOL v1.7
Polypodiaceae	Pecluma consimilis (D. C. Eaton ex Mett.) M. G. Price	trnL-trnF	KT780787	FTOL v1.7
Polypodiaceae	Pecluma curvans (Mett.) M. G. Price	rbcL	KT780731	FTOL v1.7
Polypodiaceae	Pecluma curvans (Mett.) M. G. Price	rps4	KT794106	FTOL v1.7
Polypodiaceae	Pecluma curvans (Mett.) M. G. Price	rps4-trnS	KT794106	FTOL v1.7
Polypodiaceae	Pecluma curvans (Mett.) M. G. Price	trnL-trnF	KT780788	FTOL v1.7
Polypodiaceae	Pecluma dispersa (A. M. Evans) M. G. Price	rps4	KT794107	FTOL v1.7
Polypodiaceae	Pecluma dispersa (A. M. Evans) M. G. Price	rps4-trnS	KT794107	FTOL v1.7
Polypodiaceae	Pecluma dispersa (A. M. Evans) M. G. Price	trnL-trnF	KT780789	FTOL v1.7

Polypodiaceae	Pecluma divaricata (E. Fourn.) Mickel & Beitel	rbcL	KT780732	FTOL v1.7
Polypodiaceae	Pecluma divaricata (E. Fourn.) Mickel & Beitel	rps4	KT794108	FTOL v1.7
Polypodiaceae	Pecluma divaricata (E. Fourn.) Mickel & Beitel	rps4-trnS	KT794108	FTOL v1.7
Polypodiaceae	Pecluma divaricata (E. Fourn.) Mickel & Beitel	trnL-trnF	KT780790	FTOL v1.7
Polypodiaceae	Pecluma dulcis (Poir.) F. C. Assis & Salino	plastome	MK705755	FTOL v1.7
Polypodiaceae	Pecluma eurybasis (C. Chr.) M. G. Price	atpA	EF463835	FTOL v1.7
Polypodiaceae	Pecluma eurybasis (C. Chr.) M. G. Price	atpB	EF463504	FTOL v1.7
Polypodiaceae	Pecluma eurybasis (C. Chr.) M. G. Price	rbcL	EF463255	FTOL v1.7
Polypodiaceae	Pecluma filicula (Kaulf.) M. G. Price	rbcL	KT780735	FTOL v1.7
Polypodiaceae	Pecluma filicula (Kaulf.) M. G. Price	rps4	KT794111	FTOL v1.7
Polypodiaceae	Pecluma filicula (Kaulf.) M. G. Price	rps4-trnS	KT794111	FTOL v1.7
Polypodiaceae	Pecluma filicula (Kaulf.) M. G. Price	trnL-trnF	KT780793	FTOL v1.7
Polypodiaceae	Pecluma hartwegiana (Hook.) F. C. Assis & Salino	rbcL	AY362596	FTOL v1.7
Polypodiaceae	Pecluma hygrometrica (Splitg.) M. G. Price	rbcL	KT780736	FTOL v1.7
Polypodiaceae	Pecluma hygrometrica (Splitg.) M. G. Price	rps4	KT794113	FTOL v1.7
Polypodiaceae	Pecluma hygrometrica (Splitg.) M. G. Price	rps4-trnS	KT794113	FTOL v1.7
Polypodiaceae	Pecluma hygrometrica (Splitg.) M. G. Price	trnL-trnF	KT780795	FTOL v1.7
Polypodiaceae	Pecluma insularis (Brade) Salino	rbcL	KT780737	FTOL v1.7
Polypodiaceae	Pecluma insularis (Brade) Salino	rps4	KT794114	FTOL v1.7
Polypodiaceae	Pecluma insularis (Brade) Salino	rps4-trnS	KT794114	FTOL v1.7
Polypodiaceae	Pecluma insularis (Brade) Salino	trnL-trnF	KT780796	FTOL v1.7
Polypodiaceae	Pecluma longepinnulata (E. Fourn.) F. C. Assis & Salino	rbcL	AY362605	FTOL v1.7
Polypodiaceae	Pecluma macedoi (Brade) M. Kessler & A. R. Sm.	rbcL	KT780738	FTOL v1.7
Polypodiaceae	Pecluma macedoi (Brade) M. Kessler & A. R. Sm.	rps4	KT794116	FTOL v1.7
Polypodiaceae	Pecluma macedoi (Brade) M. Kessler & A. R. Sm.	rps4-trnS	KT794116	FTOL v1.7
Polypodiaceae	Pecluma macedoi (Brade) M. Kessler & A. R. Sm.	trnL-trnF	KT780798	FTOL v1.7
Polypodiaceae	Pecluma oranensis (de la Sota) de la Sota	rbcL	KT780739	FTOL v1.7
Polypodiaceae	Pecluma oranensis (de la Sota) de la Sota	rps4	KT794117	FTOL v1.7
Polypodiaceae	Pecluma oranensis (de la Sota) de la Sota	rps4-trnS	KT794117	FTOL v1.7
Polypodiaceae	Pecluma oranensis (de la Sota) de la Sota	trnL-trnF	KT780799	FTOL v1.7
Polypodiaceae	Pecluma paradiseae (Langsd. & Fisch.) M. G. Price	rbcL	KT780740	FTOL v1.7
Polypodiaceae	Pecluma paradiseae (Langsd. & Fisch.) M. G. Price	rps4	KT794118	FTOL v1.7
Polypodiaceae	Pecluma paradiseae (Langsd. & Fisch.) M. G. Price	rps4-trnS	KT794118	FTOL v1.7
Polypodiaceae	Pecluma paradiseae (Langsd. & Fisch.) M. G. Price	trnL-trnF	KT780800	FTOL v1.7
Polypodiaceae	Pecluma pastazensis (Hieron.) R. C. Moran	rps4	KT794119	FTOL v1.7
Polypodiaceae	Pecluma pastazensis (Hieron.) R. C. Moran	rps4-trnS	KT794119	FTOL v1.7
Polypodiaceae	Pecluma pectinata (L.) M. G. Price	rbcL	KT780741	FTOL v1.7
Polypodiaceae	Pecluma pectinata (L.) M. G. Price	rps4	KT794120	FTOL v1.7
Polypodiaceae	Pecluma pectinata (L.) M. G. Price	rps4-trnS	KT794120	FTOL v1.7
Polypodiaceae	Pecluma pectinata (L.) M. G. Price	trnL-trnF	KT780801	FTOL v1.7
Polypodiaceae	Pecluma pectinatiformis (Lindm.) M. G. Price	rbcL	KT780742	FTOL v1.7
Polypodiaceae	Pecluma pectinatiformis (Lindm.) M. G. Price	rps4	KT794121	FTOL v1.7
Polypodiaceae	Pecluma pectinatiformis (Lindm.) M. G. Price	rps4-trnS	KT794121	FTOL v1.7
Polypodiaceae	Pecluma pectinatiformis (Lindm.) M. G. Price	trnL-trnF	KT780802	FTOL v1.7
Polypodiaceae	Pecluma perpinnata M. Kessler & A. R. Sm.	rbcL	KT780743	FTOL v1.7
Polypodiaceae	Pecluma perpinnata M. Kessler & A. R. Sm.	rps4	KT794122	FTOL v1.7
Polypodiaceae	Pecluma perpinnata M. Kessler & A. R. Sm.	rps4-trnS	KT794122	FTOL v1.7
Polypodiaceae	Pecluma perpinnata M. Kessler & A. R. Sm.	trnL-trnF	KT780803	FTOL v1.7
Polypodiaceae	Pecluma pilosa (A. M. Evans) M. Kessler & A. R. Sm.	rbcL	KT780744	FTOL v1.7
Polypodiaceae	Pecluma pilosa (A. M. Evans) M. Kessler & A. R. Sm.	rps4	KT794123	FTOL v1.7
Polypodiaceae	Pecluma pilosa (A. M. Evans) M. Kessler & A. R. Sm.	rps4-trnS	KT794123	FTOL v1.7
Polypodiaceae	Pecluma pilosa (A. M. Evans) M. Kessler & A. R. Sm.	trnL-trnF	KT780804	FTOL v1.7
Polypodiaceae	Pecluma plumula (Humb. & Bonpl. ex Willd.) M. G. Pri	rbcL	KR816693	FTOL v1.7
Polypodiaceae	Pecluma plumula (Humb. & Bonpl. ex Willd.) M. G. Pri	rps4	KT794124	FTOL v1.7
Polypodiaceae	Pecluma plumula (Humb. & Bonpl. ex Willd.) M. G. Pri	rps4-trnS	KT794124	FTOL v1.7
Polypodiaceae	Pecluma plumula (Humb. & Bonpl. ex Willd.) M. G. Pri	trnL-trnF	KT780805	FTOL v1.7
Polypodiaceae	Pecluma ptilotos (Kunze) M.G.Price	rbcL	KT780746	FTOL v1.7
Polypodiaceae	Pecluma ptilotos (Kunze) M.G.Price	rps4	KT794125	FTOL v1.7
Polypodiaceae	Pecluma ptilotos (Kunze) M.G.Price	rps4-trnS	KT794125	FTOL v1.7
Polypodiaceae	Pecluma ptilotos (Kunze) M.G.Price	trnL-trnF	KT780806	FTOL v1.7
Polypodiaceae	Pecluma recurvata (Kaulf.) M. G. Price	rbcL	KT780747	FTOL v1.7
Polypodiaceae	Pecluma recurvata (Kaulf.) M. G. Price	rps4	KT794126	FTOL v1.7
Polypodiaceae	Pecluma recurvata (Kaulf.) M. G. Price	rps4-trnS	KT794126	FTOL v1.7
Polypodiaceae	Pecluma recurvata (Kaulf.) M. G. Price	trnL-trnF	KT780807	FTOL v1.7
Polypodiaceae	Pecluma rhachipterygia (Liebm.) F. C. Assis & Salino	rbcL	FJ825694	FTOL v1.7
Polypodiaceae	Pecluma rhachipterygia (Liebm.) F. C. Assis & Salino	rps4	FJ825665	FTOL v1.7
Polypodiaceae	Pecluma rhachipterygia (Liebm.) F. C. Assis & Salino	rps4-trnS	FJ825665	FTOL v1.7
Polypodiaceae	Pecluma rhachipterygia (Liebm.) F. C. Assis & Salino	trnL-trnF	FJ825679	FTOL v1.7
Polypodiaceae	Pecluma robusta (Fée) M. Kessler & A. R. Sm.	trnL-trnF	AF159191	FTOL v1.7
Polypodiaceae	Pecluma sicca (Lindm.) M. G. Price	rbcL	KT780748	FTOL v1.7
Polypodiaceae	Pecluma sicca (Lindm.) M. G. Price	rps4	KT794127	FTOL v1.7
Polypodiaceae	Pecluma sicca (Lindm.) M. G. Price	rps4-trnS	KT794127	FTOL v1.7
Polypodiaceae	Pecluma sicca (Lindm.) M. G. Price	trnL-trnF	KT780808	FTOL v1.7
Polypodiaceae	Pecluma singeri (de la Sota) M. G. Price	rbcL	KT780749	FTOL v1.7
Polypodiaceae	Pecluma singeri (de la Sota) M. G. Price	rps4	KT794128	FTOL v1.7
Polypodiaceae	Pecluma singeri (de la Sota) M. G. Price	rps4-trnS	KT794128	FTOL v1.7
Polypodiaceae	Pecluma singeri (de la Sota) M. G. Price	trnL-trnF	KT780809	FTOL v1.7
Polypodiaceae	Pecluma sursumcurrens (Copel.) M. G. Price	rps4	KT794129	FTOL v1.7
Polypodiaceae	Pecluma sursumcurrens (Copel.) M. G. Price	rps4-trnS	KT794129	FTOL v1.7
Polypodiaceae	Pecluma sursumcurrens (Copel.) M. G. Price	trnL-trnF	KT780810	FTOL v1.7
Polypodiaceae	Pecluma truncorum (Lindm.) M. G. Price	rbcL	KT780750	FTOL v1.7
Polypodiaceae	Pecluma truncorum (Lindm.) M. G. Price	rps4	KT794130	FTOL v1.7
Polypodiaceae	Pecluma truncorum (Lindm.) M. G. Price	rps4-trnS	KT794130	FTOL v1.7
Polypodiaceae	Pecluma truncorum (Lindm.) M. G. Price	trnL-trnF	KT780811	FTOL v1.7
Polypodiaceae	Pecluma venturii (de la Sota) M. G. Price	rbcL	KT780751	FTOL v1.7
Polypodiaceae	Pecluma venturii (de la Sota) M. G. Price	rps4	KT794131	FTOL v1.7
Polypodiaceae	Pecluma venturii (de la Sota) M. G. Price	rps4-trnS	KT794131	FTOL v1.7
Polypodiaceae	Pecluma venturii (de la Sota) M. G. Price	trnL-trnF	KT780812	FTOL v1.7
Polypodiaceae	Phlebodium areolatum (Humb. & Bonpl. ex Willd.) J. Sı	plastome	MW876348	FTOL v1.7
Polypodiaceae	Phlebodium aureum (L.) J. Sm.	rbcL	KR816694	FTOL v1.7
Polypodiaceae	Phlebodium decumanum (Willd.) J. Sm.	atpA	EF463836	FTOL v1.7
Polypodiaceae	Phlebodium decumanum (Willd.) J. Sm.	atpB	EF463505	FTOL v1.7
Polypodiaceae	Phlebodium decumanum (Willd.) J. Sm.	rbcL	EF463256	FTOL v1.7
Polypodiaceae	Phlebodium decumanum (Willd.) J. Sm.	rps4	MN781381	FTOL v1.7
Polypodiaceae	Phlebodium decumanum (Willd.) J. Sm.	rps4-trnS	MN781381	FTOL v1.7
Polypodiaceae	Pichisermollodes albopes (C. Chr. & Ching) Fraser-Jenk	rbcL	KT289163	FTOL v1.7
Polypodiaceae	Pichisermollodes albopes (C. Chr. & Ching) Fraser-Jenk	trnL-trnF	KT289304	FTOL v1.7
Polypodiaceae	Pichisermollodes connexa (Ching) Fraser-Jenk.	plastome	MT130564	FTOL v1.7
Polypodiaceae	Pichisermollodes crenatopinnata (C. B. Clarke) Fraser-J	plastome	MW876367	FTOL v1.7
Polypodiaceae	Pichisermollodes ebenipes (Hook.) Fraser-Jenk.	plastome	OM419351	FTOL v1.7
Polypodiaceae	Pichisermollodes malacodon (Hook.) Fraser-Jenk.	plastome	MW876360	FTOL v1.7
Polypodiaceae	Pichisermollodes quasidivaricata (Hayata) Fraser-Jenk.	rbcL	KT289193	FTOL v1.7
Polypodiaceae	Pichisermollodes quasidivaricata (Hayata) Fraser-Jenk.	trnL-trnF	KT289333	FTOL v1.7
Polypodiaceae	Pichisermollodes stewartii (Bedd.) Fraser-Jenk.	rbcL	JQ685433	FTOL v1.7
Polypodiaceae	Pichisermollodes tibetana (Ching & S. K. Wu) Fraser-Je	rbcL	KT289195	FTOL v1.7
Polypodiaceae	Pichisermollodes tibetana (Ching & S. K. Wu) Fraser-Je	trnL-trnF	KT289335	FTOL v1.7
Polypodiaceae	Pichisermollodes veitchii (Baker) Fraser-Jenk.	rbcL	KT289196	FTOL v1.7
Polypodiaceae	Pichisermollodes veitchii (Baker) Fraser-Jenk.	trnL-trnF	KT289337	FTOL v1.7
Polypodiaceae	Platycerium alcicorne Desv.	plastome	OR601548	FTOL v1.7
Polypodiaceae	Platycerium andinum Baker	plastome	OR601544	FTOL v1.7
Polypodiaceae	Platycerium bifurcatum (Cav.) C. Chr.	plastome	MN623367	FTOL v1.7
Polypodiaceae	Platycerium coronarium (D. Koenig ex O. F. M羹ll.) Des	plastome	OR601553	FTOL v1.7
Polypodiaceae	Platycerium elephantotis Schweinf.	plastome	OR601546	FTOL v1.7
Polypodiaceae	Platycerium ellisii Baker	plastome	OR601547	FTOL v1.7
Polypodiaceae	Platycerium grande (Fée) Kunze	plastome	OR601557	FTOL v1.7
Polypodiaceae	Platycerium hillii T. Moore	plastome	OR601558	FTOL v1.7
Polypodiaceae	Platycerium holttumii de Jonch. & Hennipman	plastome	OR601556	FTOL v1.7
Polypodiaceae	Platycerium madagascariense Baker	plastome	OR601550	FTOL v1.7
Polypodiaceae	Platycerium quadridichotomum (Bonap.) Tardieu	plastome	OR601549	FTOL v1.7
Polypodiaceae	Platycerium ridleyi Christ	plastome	OR601554	FTOL v1.7
Polypodiaceae	Platycerium stemaria (P. Beauv.) Desv.	plastome	OR601545	FTOL v1.7
Polypodiaceae	Platycerium superbum de Jonch. & Hennipman	plastome	OR601551	FTOL v1.7
Polypodiaceae	Platycerium veitchii (Underw.) C. Chr.	plastome	OR601561	FTOL v1.7
Polypodiaceae	Platycerium wallichii Hook.	plastome	MT130688	FTOL v1.7
Polypodiaceae	Platycerium wandae Racib.	plastome	OR601552	FTOL v1.7
Polypodiaceae	Pleopeltis acicularis (Weath.) A. R. Sm. & T. Krömer	rbcL	EU650125	FTOL v1.7
Polypodiaceae	Pleopeltis acicularis (Weath.) A. R. Sm. & T. Krömer	rps4	EU650164	FTOL v1.7
Polypodiaceae	Pleopeltis acicularis (Weath.) A. R. Sm. & T. Krömer	rps4-trnS	EU650164	FTOL v1.7
Polypodiaceae	Pleopeltis acicularis (Weath.) A. R. Sm. & T. Krömer	trnL-trnF	EU650086	FTOL v1.7
Polypodiaceae	Pleopeltis angusta var. stenoloma (Fée) Farw.	rbcL	EU650123	FTOL v1.7
Polypodiaceae	Pleopeltis angusta var. stenoloma (Fée) Farw.	rps4	EU650162	FTOL v1.7
Polypodiaceae	Pleopeltis angusta var. stenoloma (Fée) Farw.	rps4-trnS	EU650162	FTOL v1.7
Polypodiaceae	Pleopeltis angusta var. stenoloma (Fée) Farw.	trnL-trnF	EU650084	FTOL v1.7
Polypodiaceae	Pleopeltis appressa M. Kessler & A. R. Sm.	rbcL	DQ642172	FTOL v1.7
Polypodiaceae	Pleopeltis appressa M. Kessler & A. R. Sm.	rps4	DQ642211	FTOL v1.7
Polypodiaceae	Pleopeltis appressa M. Kessler & A. R. Sm.	rps4-trnS	DQ642211	FTOL v1.7
Polypodiaceae	Pleopeltis appressa M. Kessler & A. R. Sm.	trnL-trnF	DQ642259	FTOL v1.7
Polypodiaceae	Pleopeltis astrolepis (Liebm.) E. Fourn.	rbcL	EU650106	FTOL v1.7
Polypodiaceae	Pleopeltis astrolepis (Liebm.) E. Fourn.	rps4	EU650145	FTOL v1.7
Polypodiaceae	Pleopeltis astrolepis (Liebm.) E. Fourn.	rps4-trnS	EU650145	FTOL v1.7
Polypodiaceae	Pleopeltis astrolepis (Liebm.) E. Fourn.	trnL-trnF	EU650067	FTOL v1.7
Polypodiaceae	Pleopeltis balaonensis (Hieron.) A. R. Sm.	rbcL	EU650137	FTOL v1.7
Polypodiaceae	Pleopeltis balaonensis (Hieron.) A. R. Sm.	rps4	EU650176	FTOL v1.7
Polypodiaceae	Pleopeltis balaonensis (Hieron.) A. R. Sm.	rps4-trnS	EU650176	FTOL v1.7
Polypodiaceae	Pleopeltis balaonensis (Hieron.) A. R. Sm.	trnL-trnF	EU650098	FTOL v1.7
Polypodiaceae	Pleopeltis bolivianii (Rosenst.) A. R. Sm.	rbcL	DQ642173	FTOL v1.7
Polypodiaceae	Pleopeltis bolivianii (Rosenst.) A. R. Sm.	rps4	DQ642212	FTOL v1.7
Polypodiaceae	Pleopeltis bolivianii (Rosenst.) A. R. Sm.	rps4-trnS	DQ642212	FTOL v1.7
Polypodiaceae	Pleopeltis bolivianii (Rosenst.) A. R. Sm.	trnL-trnF	DQ642260	FTOL v1.7
Polypodiaceae	Pleopeltis bombycina (Maxon) A. R. Sm.	rbcL	EU650136	FTOL v1.7
Polypodiaceae	Pleopeltis bombycina (Maxon) A. R. Sm.	rps4	EU650175	FTOL v1.7
Polypodiaceae	Pleopeltis bombycina (Maxon) A. R. Sm.	rps4-trnS	EU650175	FTOL v1.7
Polypodiaceae	Pleopeltis bombycina (Maxon) A. R. Sm.	trnL-trnF	EU650097	FTOL v1.7
Polypodiaceae	Pleopeltis bradeorum (Rosenst.) A. R. Sm. & Tejero	rbcL	AY362614	FTOL v1.7
Polypodiaceae	Pleopeltis bradeorum (Rosenst.) A. R. Sm. & Tejero	rps4-trnS	AY362686	FTOL v1.7
Polypodiaceae	Pleopeltis buchtienii (Christ & Rosenst.) A. R. Sm.	rbcL	DQ642175	FTOL v1.7
Polypodiaceae	Pleopeltis buchtienii (Christ & Rosenst.) A. R. Sm.	rps4	DQ642214	FTOL v1.7
Polypodiaceae	Pleopeltis buchtienii (Christ & Rosenst.) A. R. Sm.	rps4-trnS	DQ642214	FTOL v1.7
Polypodiaceae	Pleopeltis buchtienii (Christ & Rosenst.) A. R. Sm.	trnL-trnF	DQ642262	FTOL v1.7
Polypodiaceae	Pleopeltis burchellii (Baker) Hickey & Sprunt ex A. R. Sı	trnL-trnF	HQ641029	FTOL v1.7
Polypodiaceae	Pleopeltis christensenii A. R. Sm.	atpB	KM114060	FTOL v1.7
Polypodiaceae	Pleopeltis christensenii A. R. Sm.	rbcL	KJ628697	FTOL v1.7
Polypodiaceae	Pleopeltis christensenii A. R. Sm.	rps4	EU650138	FTOL v1.7
Polypodiaceae	Pleopeltis christensenii A. R. Sm.	rps4-trnS	EU650138	FTOL v1.7
Polypodiaceae	Pleopeltis collinsii (Maxon) A. R. Sm. & Tejero	rbcL	EU650130	FTOL v1.7
Polypodiaceae	Pleopeltis collinsii (Maxon) A. R. Sm. & Tejero	rps4	EU650169	FTOL v1.7
Polypodiaceae	Pleopeltis collinsii (Maxon) A. R. Sm. & Tejero	rps4-trnS	EU650169	FTOL v1.7
Polypodiaceae	Pleopeltis collinsii (Maxon) A. R. Sm. & Tejero	trnL-trnF	EU650091	FTOL v1.7
Polypodiaceae	Pleopeltis complanata (Weath.) E. A. Hooper	rbcL	MW138186	FTOL v1.7
Polypodiaceae	Pleopeltis conzattii (Weath.) R. M. Tryon & A. F. Tryon	rbcL	EU650103	FTOL v1.7
Polypodiaceae	Pleopeltis conzattii (Weath.) R. M. Tryon & A. F. Tryon	rps4	EU650142	FTOL v1.7
Polypodiaceae	Pleopeltis conzattii (Weath.) R. M. Tryon & A. F. Tryon	rps4-trnS	EU650142	FTOL v1.7
Polypodiaceae	Pleopeltis conzattii (Weath.) R. M. Tryon & A. F. Tryon	trnL-trnF	EU650064	FTOL v1.7
Polypodiaceae	Pleopeltis crassinervata (Fée) T. Moore	rbcL	EU650105	FTOL v1.7
Polypodiaceae	Pleopeltis crassinervata (Fée) T. Moore	rps4	EU650144	FTOL v1.7
Polypodiaceae	Pleopeltis crassinervata (Fée) T. Moore	rps4-trnS	EU650144	FTOL v1.7
Polypodiaceae	Pleopeltis crassinervata (Fée) T. Moore	trnL-trnF	EU650066	FTOL v1.7
Polypodiaceae	Pleopeltis desvauxii (Klotzsch) Salino	rbcL	AY362584	FTOL v1.7
Polypodiaceae	Pleopeltis disjuncta M. Kessler & A. R. Sm.	rbcL	EU650127	FTOL v1.7
Polypodiaceae	Pleopeltis disjuncta M. Kessler & A. R. Sm.	rps4	EU650166	FTOL v1.7
Polypodiaceae	Pleopeltis disjuncta M. Kessler & A. R. Sm.	rps4-trnS	EU650166	FTOL v1.7
Polypodiaceae	Pleopeltis disjuncta M. Kessler & A. R. Sm.	trnL-trnF	EU650088	FTOL v1.7
Polypodiaceae	Pleopeltis ecklonii (Kunze) A. R. Sm.	trnL-trnF	HQ641002	FTOL v1.7
Polypodiaceae	Pleopeltis fallax (Schltdl. & Cham.) Mickel & Beitel	rbcL	MW620401	FTOL v1.7
Polypodiaceae	Pleopeltis fallax (Schltdl. & Cham.) Mickel & Beitel	rps4	EU650147	FTOL v1.7
Polypodiaceae	Pleopeltis fallax (Schltdl. & Cham.) Mickel & Beitel	rps4-trnS	EU650147	FTOL v1.7
Polypodiaceae	Pleopeltis fallax (Schltdl. & Cham.) Mickel & Beitel	trnL-trnF	EU650069	FTOL v1.7

Polypodiaceae	Pleopeltis fraseri (Kuhn) A. R. Sm.	rbcL	DQ642176	FTOL v1.7
Polypodiaceae	Pleopeltis fraseri (Kuhn) A. R. Sm.	rps4	DQ642215	FTOL v1.7
Polypodiaceae	Pleopeltis fraseri (Kuhn) A. R. Sm.	rps4-trnS	DQ642215	FTOL v1.7
Polypodiaceae	Pleopeltis fraseri (Kuhn) A. R. Sm.	trnL-trnF	DQ642263	FTOL v1.7
Polypodiaceae	Pleopeltis friedrichsthaliana (Kunze) A. R. Sm. & Tejero	rbcL	EU650128	FTOL v1.7
Polypodiaceae	Pleopeltis friedrichsthaliana (Kunze) A. R. Sm. & Tejero	rps4	EU650167	FTOL v1.7
Polypodiaceae	Pleopeltis friedrichsthaliana (Kunze) A. R. Sm. & Tejero	rps4-trnS	EU650167	FTOL v1.7
Polypodiaceae	Pleopeltis friedrichsthaliana (Kunze) A. R. Sm. & Tejero	trnL-trnF	EU650089	FTOL v1.7
Polypodiaceae	Pleopeltis fructuosa (Maxon & Weath.) Lellinger	rbcL	EU650101	FTOL v1.7
Polypodiaceae	Pleopeltis fructuosa (Maxon & Weath.) Lellinger	rps4	EU650140	FTOL v1.7
Polypodiaceae	Pleopeltis fructuosa (Maxon & Weath.) Lellinger	rps4-trnS	EU650140	FTOL v1.7
Polypodiaceae	Pleopeltis fructuosa (Maxon & Weath.) Lellinger	trnL-trnF	EU650062	FTOL v1.7
Polypodiaceae	Pleopeltis furfuracea (Schltdl. & Cham.) A. R. Sm. & Te	rbcL	EU650126	FTOL v1.7
Polypodiaceae	Pleopeltis furfuracea (Schltdl. & Cham.) A. R. Sm. & Te	rps4	EU650165	FTOL v1.7
Polypodiaceae	Pleopeltis furfuracea (Schltdl. & Cham.) A. R. Sm. & Te	rps4-trnS	EU650165	FTOL v1.7
Polypodiaceae	Pleopeltis furfuracea (Schltdl. & Cham.) A. R. Sm. & Te	trnL-trnF	EU650087	FTOL v1.7
Polypodiaceae	Pleopeltis guttata (Maxon) E. G. Andrews & Windham	rbcL	EU650111	FTOL v1.7
Polypodiaceae	Pleopeltis guttata (Maxon) E. G. Andrews & Windham	rps4	EU650150	FTOL v1.7
Polypodiaceae	Pleopeltis guttata (Maxon) E. G. Andrews & Windham	rps4-trnS	EU650150	FTOL v1.7
Polypodiaceae	Pleopeltis guttata (Maxon) E. G. Andrews & Windham	trnL-trnF	EU650072	FTOL v1.7
Polypodiaceae	Pleopeltis hirsutissima (Raddi) de la Sota	rbcL	EU650135	FTOL v1.7
Polypodiaceae	Pleopeltis hirsutissima (Raddi) de la Sota	rps4	EU650174	FTOL v1.7
Polypodiaceae	Pleopeltis hirsutissima (Raddi) de la Sota	rps4-trnS	EU650174	FTOL v1.7
Polypodiaceae	Pleopeltis hirsutissima (Raddi) de la Sota	trnL-trnF	EU650096	FTOL v1.7
Polypodiaceae	Pleopeltis intermedia M. Kessler & A. R. Sm.	rbcL	EU650107	FTOL v1.7
Polypodiaceae	Pleopeltis intermedia M. Kessler & A. R. Sm.	rps4	EU650146	FTOL v1.7
Polypodiaceae	Pleopeltis intermedia M. Kessler & A. R. Sm.	rps4-trnS	EU650146	FTOL v1.7
Polypodiaceae	Pleopeltis intermedia M. Kessler & A. R. Sm.	trnL-trnF	EU650068	FTOL v1.7
Polypodiaceae	Pleopeltis lepidotricha (Fée) A. R. Sm. & Tejero	rbcL	EU650129	FTOL v1.7
Polypodiaceae	Pleopeltis lepidotricha (Fée) A. R. Sm. & Tejero	rps4	EU650168	FTOL v1.7
Polypodiaceae	Pleopeltis lepidotricha (Fée) A. R. Sm. & Tejero	rps4-trnS	EU650168	FTOL v1.7
Polypodiaceae	Pleopeltis lepidotricha (Fée) A. R. Sm. & Tejero	trnL-trnF	EU650090	FTOL v1.7
Polypodiaceae	Pleopeltis macrocarpa (Bory ex Willd.) Kaulf.	plastome	MW876352	FTOL v1.7
Polypodiaceae	Pleopeltis macrolepis (Maxon) A. R. Sm. & Tejero	rbcL	EU650134	FTOL v1.7
Polypodiaceae	Pleopeltis macrolepis (Maxon) A. R. Sm. & Tejero	rps4	EU650173	FTOL v1.7
Polypodiaceae	Pleopeltis macrolepis (Maxon) A. R. Sm. & Tejero	rps4-trnS	EU650173	FTOL v1.7
Polypodiaceae	Pleopeltis macrolepis (Maxon) A. R. Sm. & Tejero	trnL-trnF	EU650095	FTOL v1.7
Polypodiaceae	Pleopeltis madrensis (J. Sm.) A. R. Sm. & Tejero	rbcL	EU650110	FTOL v1.7
Polypodiaceae	Pleopeltis madrensis (J. Sm.) A. R. Sm. & Tejero	rps4	EU650149	FTOL v1.7
Polypodiaceae	Pleopeltis madrensis (J. Sm.) A. R. Sm. & Tejero	rps4-trnS	EU650149	FTOL v1.7
Polypodiaceae	Pleopeltis madrensis (J. Sm.) A. R. Sm. & Tejero	trnL-trnF	EU650071	FTOL v1.7
Polypodiaceae	Pleopeltis marginata A. R. Sm. & Tejero	rbcL	AY362583	FTOL v1.7
Polypodiaceae	Pleopeltis marginata A. R. Sm. & Tejero	rps4	EU650139	FTOL v1.7
Polypodiaceae	Pleopeltis marginata A. R. Sm. & Tejero	rps4-trnS	EU650139	FTOL v1.7
Polypodiaceae	Pleopeltis marginata A. R. Sm. & Tejero	trnL-trnF	EU650061	FTOL v1.7
Polypodiaceae	Pleopeltis mexicana (Fée) Mickel & Beitel	rbcL	EU650102	FTOL v1.7
Polypodiaceae	Pleopeltis mexicana (Fée) Mickel & Beitel	rps4	EU650141	FTOL v1.7
Polypodiaceae	Pleopeltis mexicana (Fée) Mickel & Beitel	rps4-trnS	EU650141	FTOL v1.7
Polypodiaceae	Pleopeltis mexicana (Fée) Mickel & Beitel	trnL-trnF	EU650063	FTOL v1.7
Polypodiaceae	Pleopeltis michauxiana (Weath.) Hickey & Sprunt	rbcL	MH549950	FTOL v1.7
Polypodiaceae	Pleopeltis minima (Bory) J. Prado & R. Y. Hirai	trnL-trnF	HQ641018	FTOL v1.7
Polypodiaceae	Pleopeltis monosora (Desv.) A. R. Sm.	rbcL	EU650118	FTOL v1.7
Polypodiaceae	Pleopeltis monosora (Desv.) A. R. Sm.	rps4	EU650157	FTOL v1.7
Polypodiaceae	Pleopeltis monosora (Desv.) A. R. Sm.	rps4-trnS	EU650157	FTOL v1.7
Polypodiaceae	Pleopeltis monosora (Desv.) A. R. Sm.	trnL-trnF	EU650079	FTOL v1.7
Polypodiaceae	Pleopeltis montigena (Maxon) A. R. Sm. & Tejero	rbcL	EU650114	FTOL v1.7
Polypodiaceae	Pleopeltis montigena (Maxon) A. R. Sm. & Tejero	rps4	EU650153	FTOL v1.7
Polypodiaceae	Pleopeltis montigena (Maxon) A. R. Sm. & Tejero	rps4-trnS	EU650153	FTOL v1.7
Polypodiaceae	Pleopeltis montigena (Maxon) A. R. Sm. & Tejero	trnL-trnF	EU650075	FTOL v1.7
Polypodiaceae	Pleopeltis munchii (Christ) A. R. Sm.	rbcL	EU650113	FTOL v1.7
Polypodiaceae	Pleopeltis munchii (Christ) A. R. Sm.	rps4	EU650152	FTOL v1.7
Polypodiaceae	Pleopeltis munchii (Christ) A. R. Sm.	rps4-trnS	EU650152	FTOL v1.7
Polypodiaceae	Pleopeltis munchii (Christ) A. R. Sm.	trnL-trnF	EU650074	FTOL v1.7
Polypodiaceae	Pleopeltis murorum (Hook.) A.R.Sm. & Tejero	rbcL	EU650119	FTOL v1.7
Polypodiaceae	Pleopeltis murorum (Hook.) A.R.Sm. & Tejero	rps4	EU650158	FTOL v1.7
Polypodiaceae	Pleopeltis murorum (Hook.) A.R.Sm. & Tejero	rps4-trnS	EU650158	FTOL v1.7
Polypodiaceae	Pleopeltis murorum (Hook.) A.R.Sm. & Tejero	trnL-trnF	EU650080	FTOL v1.7
Polypodiaceae	Pleopeltis myriolepis (Christ) A. R. Sm. & Tejero	rbcL	EU650131	FTOL v1.7
Polypodiaceae	Pleopeltis myriolepis (Christ) A. R. Sm. & Tejero	rps4	EU650170	FTOL v1.7
Polypodiaceae	Pleopeltis myriolepis (Christ) A. R. Sm. & Tejero	rps4-trnS	EU650170	FTOL v1.7
Polypodiaceae	Pleopeltis myriolepis (Christ) A. R. Sm. & Tejero	trnL-trnF	EU650092	FTOL v1.7
Polypodiaceae	Pleopeltis platylepis (Mett. ex Kuhn) A. R. Sm. & Tejero	rbcL	EU650115	FTOL v1.7
Polypodiaceae	Pleopeltis platylepis (Mett. ex Kuhn) A. R. Sm. & Tejero	rps4	EU650154	FTOL v1.7
Polypodiaceae	Pleopeltis platylepis (Mett. ex Kuhn) A. R. Sm. & Tejero	rps4-trnS	EU650154	FTOL v1.7
Polypodiaceae	Pleopeltis platylepis (Mett. ex Kuhn) A. R. Sm. & Tejero	trnL-trnF	EU650076	FTOL v1.7
Polypodiaceae	Pleopeltis plebeia (Schltdl. & Cham.) A. R. Sm. & Tejer	rbcL	EU650116	FTOL v1.7
Polypodiaceae	Pleopeltis plebeia (Schltdl. & Cham.) A. R. Sm. & Tejer	rps4	EU650155	FTOL v1.7
Polypodiaceae	Pleopeltis plebeia (Schltdl. & Cham.) A. R. Sm. & Tejer	rps4-trnS	EU650155	FTOL v1.7
Polypodiaceae	Pleopeltis plebeia (Schltdl. & Cham.) A. R. Sm. & Tejer	trnL-trnF	EU650077	FTOL v1.7
Polypodiaceae	Pleopeltis pleopeltifolia (Raddi) Alston	rbcL	EU650121	FTOL v1.7
Polypodiaceae	Pleopeltis pleopeltifolia (Raddi) Alston	rps4	EU650160	FTOL v1.7
Polypodiaceae	Pleopeltis pleopeltifolia (Raddi) Alston	rps4-trnS	EU650160	FTOL v1.7
Polypodiaceae	Pleopeltis pleopeltifolia (Raddi) Alston	trnL-trnF	EU650082	FTOL v1.7
Polypodiaceae	Pleopeltis polylepis (Roem. ex Kunze) T. Moore	rbcL	EU650104	FTOL v1.7
Polypodiaceae	Pleopeltis polylepis (Roem. ex Kunze) T. Moore	rps4	EU650143	FTOL v1.7
Polypodiaceae	Pleopeltis polylepis (Roem. ex Kunze) T. Moore	rps4-trnS	EU650143	FTOL v1.7
Polypodiaceae	Pleopeltis polylepis (Roem. ex Kunze) T. Moore	trnL-trnF	EU650065	FTOL v1.7
Polypodiaceae	Pleopeltis polypodioides (L.) E. G. Andrews & Windhan	plastome	MW876353	FTOL v1.7
Polypodiaceae	Pleopeltis pycnocarpa (C. Chr.) A. R. Sm.	rbcL	EU650120	FTOL v1.7
Polypodiaceae	Pleopeltis pycnocarpa (C. Chr.) A. R. Sm.	rps4	EU650159	FTOL v1.7
Polypodiaceae	Pleopeltis pycnocarpa (C. Chr.) A. R. Sm.	rps4-trnS	EU650159	FTOL v1.7
Polypodiaceae	Pleopeltis pycnocarpa (C. Chr.) A. R. Sm.	trnL-trnF	EU650081	FTOL v1.7
Polypodiaceae	Pleopeltis pyrrholepis (Fée) A. R. Sm. & Tejero	rbcL	EU650133	FTOL v1.7
Polypodiaceae	Pleopeltis pyrrholepis (Fée) A. R. Sm. & Tejero	rps4	EU650172	FTOL v1.7
Polypodiaceae	Pleopeltis pyrrholepis (Fée) A. R. Sm. & Tejero	rps4-trnS	EU650172	FTOL v1.7
Polypodiaceae	Pleopeltis pyrrholepis (Fée) A. R. Sm. & Tejero	trnL-trnF	EU650094	FTOL v1.7
Polypodiaceae	Pleopeltis remota (Desv.) A. R. Sm.	rbcL	DQ642180	FTOL v1.7
Polypodiaceae	Pleopeltis remota (Desv.) A. R. Sm.	rps4	DQ642220	FTOL v1.7
Polypodiaceae	Pleopeltis remota (Desv.) A. R. Sm.	rps4-trnS	DQ642220	FTOL v1.7
Polypodiaceae	Pleopeltis remota (Desv.) A. R. Sm.	trnL-trnF	DQ642268	FTOL v1.7
Polypodiaceae	Pleopeltis rosei (Maxon) A. R. Sm. & Tejero	rbcL	AY362608	FTOL v1.7
Polypodiaceae	Pleopeltis rosei (Maxon) A. R. Sm. & Tejero	rps4-trnS	AY362680	FTOL v1.7
Polypodiaceae	Pleopeltis rzedowskiana (Mickel) A. R. Sm. & Tejero	rbcL	EU650117	FTOL v1.7
Polypodiaceae	Pleopeltis rzedowskiana (Mickel) A. R. Sm. & Tejero	rps4	EU650156	FTOL v1.7
Polypodiaceae	Pleopeltis rzedowskiana (Mickel) A. R. Sm. & Tejero	rps4-trnS	EU650156	FTOL v1.7
Polypodiaceae	Pleopeltis rzedowskiana (Mickel) A. R. Sm. & Tejero	trnL-trnF	EU650078	FTOL v1.7
Polypodiaceae	Pleopeltis sanctae-rosae (Maxon) A. R. Sm. & Tejero	atpA	EF463840	FTOL v1.7
Polypodiaceae	Pleopeltis sanctae-rosae (Maxon) A. R. Sm. & Tejero	atpB	EF463509	FTOL v1.7
Polypodiaceae	Pleopeltis sanctae-rosae (Maxon) A. R. Sm. & Tejero	rbcL	EF463258	FTOL v1.7
Polypodiaceae	Pleopeltis thyssanolepis (A. Braun ex Klotzsch) E. G. Ar	rbcL	EU650112	FTOL v1.7
Polypodiaceae	Pleopeltis thyssanolepis (A. Braun ex Klotzsch) E. G. Ar	rps4	EU650151	FTOL v1.7
Polypodiaceae	Pleopeltis thyssanolepis (A. Braun ex Klotzsch) E. G. Ar	rps4-trnS	EU650151	FTOL v1.7
Polypodiaceae	Pleopeltis thyssanolepis (A. Braun ex Klotzsch) E. G. Ar	trnL-trnF	EU650073	FTOL v1.7
Polypodiaceae	Pleopeltis tweediana (Hook.) A. R. Sm.	rbcL	DQ642182	FTOL v1.7
Polypodiaceae	Pleopeltis tweediana (Hook.) A. R. Sm.	rps4	DQ642222	FTOL v1.7
Polypodiaceae	Pleopeltis tweediana (Hook.) A. R. Sm.	rps4-trnS	DQ642222	FTOL v1.7
Polypodiaceae	Pleopeltis tweediana (Hook.) A. R. Sm.	trnL-trnF	DQ642270	FTOL v1.7
Polypodiaceae	Pleopeltis villagranii (Copel.) A. R. Sm. & Tejero	rbcL	MW620404	FTOL v1.7
Polypodiaceae	Pleopeltis wiesbaurii (Sodiro) Lellinger	rbcL	EU650109	FTOL v1.7
Polypodiaceae	Pleopeltis wiesbaurii (Sodiro) Lellinger	rps4	EU650148	FTOL v1.7
Polypodiaceae	Pleopeltis wiesbaurii (Sodiro) Lellinger	rps4-trnS	EU650148	FTOL v1.7
Polypodiaceae	Pleopeltis wiesbaurii (Sodiro) Lellinger	trnL-trnF	EU650070	FTOL v1.7
Polypodiaceae	Pleurosoriopsis makinoi (Maxim.) Fomin	plastome	MW876354	FTOL v1.7
Polypodiaceae	Polypodium × vianei Shmakov	rbcL	OR805769	FTOL v1.7
Polypodiaceae	Polypodium amorphum Suksd.	rbcL	U21142	FTOL v1.7
Polypodiaceae	Polypodium appalachianum Haufler & Windham	rbcL	U21141	FTOL v1.7
Polypodiaceae	Polypodium arcanum Maxon	plastome	MZ357090	FTOL v1.7
Polypodiaceae	Polypodium californicum Kaulf.	rbcL	KF909040	FTOL v1.7
Polypodiaceae	Polypodium californicum Kaulf.	trnL-trnF	AF159189	FTOL v1.7
Polypodiaceae	Polypodium cambricum L.	rbcL	HE963605	FTOL v1.7
Polypodiaceae	Polypodium colpodes Kunze	rbcL	KF909044	FTOL v1.7
Polypodiaceae	Polypodium conterminans Liebm.	rbcL	OP711662	FTOL v1.7
Polypodiaceae	Polypodium eatonii Baker	rbcL	MW620246	FTOL v1.7
Polypodiaceae	Polypodium echinolepis Fée	rbcL	FJ825701	FTOL v1.7
Polypodiaceae	Polypodium echinolepis Fée	rps4	FJ825672	FTOL v1.7
Polypodiaceae	Polypodium echinolepis Fée	rps4-trnS	FJ825672	FTOL v1.7
Polypodiaceae	Polypodium echinolepis Fée	trnL-trnF	FJ825686	FTOL v1.7
Polypodiaceae	Polypodium ensiforme Thunb.	rbcL	AY362598	FTOL v1.7
Polypodiaceae	Polypodium ensiforme Thunb.	rps4-trnS	AY362671	FTOL v1.7
Polypodiaceae	Polypodium fauriei Christ	rbcL	U21148	FTOL v1.7
Polypodiaceae	Polypodium glycyrrhiza D. C. Eaton	plastome	KP136832	FTOL v1.7
Polypodiaceae	Polypodium hesperium Maxon	atpB	EU352282	FTOL v1.7
Polypodiaceae	Polypodium hesperium Maxon	rbcL	EU352309	FTOL v1.7
Polypodiaceae	Polypodium interjectum Shivas	rbcL	HE963606	FTOL v1.7
Polypodiaceae	Polypodium kamelinii Shmakov	rbcL	OR805770	FTOL v1.7
Polypodiaceae	Polypodium macaronesicum Bobrov	rbcL	U21151	FTOL v1.7
Polypodiaceae	Polypodium martensii Mett.	rbcL	MW620351	FTOL v1.7
Polypodiaceae	Polypodium pellucidum Kaulf.	rbcL	U21149	FTOL v1.7
Polypodiaceae	Polypodium pellucidum Kaulf.	rps4	AY096234	FTOL v1.7
Polypodiaceae	Polypodium pellucidum Kaulf.	rps4-trnS	AY096234	FTOL v1.7
Polypodiaceae	Polypodium pellucidum Kaulf.	trnL-trnF	AF159190	FTOL v1.7
Polypodiaceae	Polypodium plesiosorum Kunze	rbcL	U21144	FTOL v1.7
Polypodiaceae	Polypodium puberulum Schltdl. & Cham.	rbcL	U21143	FTOL v1.7
Polypodiaceae	Polypodium rhodopleuron Kunze	plastome	MZ357091	FTOL v1.7
Polypodiaceae	Polypodium scouleri Hook. & Grev.	rbcL	U21150	FTOL v1.7
Polypodiaceae	Polypodium sibiricum Sipliv.	plastome	MW876355	FTOL v1.7
Polypodiaceae	Polypodium subpetiolatum Hook.	plastome	MZ357092	FTOL v1.7
Polypodiaceae	Polypodium virginianum L.	atpA	KF186559	FTOL v1.7
Polypodiaceae	Polypodium virginianum L.	rbcL	KF186527	FTOL v1.7
Polypodiaceae	Polypodium vulgare L.	plastome	MZ357093	FTOL v1.7
Polypodiaceae	Prosaptia alata (Blume) Christ	atpB	KM218848	FTOL v1.7
Polypodiaceae	Prosaptia alata (Blume) Christ	rbcL	KM218764	FTOL v1.7
Polypodiaceae	Prosaptia alata (Blume) Christ	rps4-trnS	KM106177	FTOL v1.7
Polypodiaceae	Prosaptia alata (Blume) Christ	trnL-trnF	KM106082	FTOL v1.7
Polypodiaceae	Prosaptia barathrophylla (Baker) M. G. Price	atpB	OQ702696	FTOL v1.7
Polypodiaceae	Prosaptia barathrophylla (Baker) M. G. Price	rps4	OQ717238	FTOL v1.7
Polypodiaceae	Prosaptia barathrophylla (Baker) M. G. Price	rps4-trnS	OQ717238	FTOL v1.7
Polypodiaceae	Prosaptia barathrophylla (Baker) M. G. Price	trnL-trnF	OQ717155	FTOL v1.7
Polypodiaceae	Prosaptia celebica (Blume) Tagawa & K. Iwats.	atpB	KM218849	FTOL v1.7
Polypodiaceae	Prosaptia celebica (Blume) Tagawa & K. Iwats.	rbcL	KM218786	FTOL v1.7
Polypodiaceae	Prosaptia celebica (Blume) Tagawa & K. Iwats.	rps4-trnS	KM106178	FTOL v1.7
Polypodiaceae	Prosaptia contigua (G. Forst.) C. Presl	atpB	KY711838	FTOL v1.7
Polypodiaceae	Prosaptia contigua (G. Forst.) C. Presl	rbcL	KY712014	FTOL v1.7
Polypodiaceae	Prosaptia contigua (G. Forst.) C. Presl	rps4-trnS	KY712330	FTOL v1.7
Polypodiaceae	Prosaptia contigua (G. Forst.) C. Presl	trnL-trnF	KY711663	FTOL v1.7
Polypodiaceae	Prosaptia davalliacea (F. Muell. & Baker) Copel.	atpB	KM218852	FTOL v1.7
Polypodiaceae	Prosaptia davalliacea (F. Muell. & Baker) Copel.	rbcL	KM218798	FTOL v1.7
Polypodiaceae	Prosaptia davalliacea (F. Muell. & Baker) Copel.	rps4-trnS	KM106179	FTOL v1.7
Polypodiaceae	Prosaptia davalliacea (F. Muell. & Baker) Copel.	trnL-trnF	KM106084	FTOL v1.7
Polypodiaceae	Prosaptia formosana (Hayata) T. C. Hsu	atpB	OQ702706	FTOL v1.7
Polypodiaceae	Prosaptia formosana (Hayata) T. C. Hsu	rps4	OQ717217	FTOL v1.7
Polypodiaceae	Prosaptia formosana (Hayata) T. C. Hsu	rps4-trnS	OQ717217	FTOL v1.7
Polypodiaceae	Prosaptia formosana (Hayata) T. C. Hsu	trnL-trnF	OQ717160	FTOL v1.7
Polypodiaceae	Prosaptia kanashiroi (Hayata) Nakai	rbcL	AB575253	FTOL v1.7
Polypodiaceae	Prosaptia khasyana (Hook.) C. Chr. & Tardieu	plastome	MW876356	FTOL v1.7
Polypodiaceae	Prosaptia maidenii (Watts) Parris	rbcL	KM218766	FTOL v1.7
Polypodiaceae	Prosaptia nutans (Blume) Mett.	atpB	AY459464	FTOL v1.7

Polypodiaceae	Prosaptia nutans (Blume) Mett.	rbcL	AY460631	FTOL v1.7
Polypodiaceae	Prosaptia obliquata (Blume) Mett.	plastome	MW876357	FTOL v1.7
Polypodiaceae	Prosaptia palauensis Hosok.	rbcL	AY460662	FTOL v1.7
Polypodiaceae	Prosaptia pectinata T. Moore	atpB	OQ702707	FTOL v1.7
Polypodiaceae	Prosaptia pectinata T. Moore	rps4	OQ717235	FTOL v1.7
Polypodiaceae	Prosaptia pectinata T. Moore	rps4-trnS	OQ717235	FTOL v1.7
Polypodiaceae	Prosaptia pectinata T. Moore	trnL-trnF	OQ717168	FTOL v1.7
Polypodiaceae	Prosaptia pubipes Copel.	atpB	AY459496	FTOL v1.7
Polypodiaceae	Prosaptia pubipes Copel.	rbcL	AY460663	FTOL v1.7
Polypodiaceae	Prosaptia rhodocarpa (Copel.) Parris	atpB	AY459467	FTOL v1.7
Polypodiaceae	Prosaptia rhodocarpa (Copel.) Parris	rbcL	AY460634	FTOL v1.7
Polypodiaceae	Prosaptia samoensis (C. Chr.) Parris	rbcL	MT657598	FTOL v1.7
Polypodiaceae	Prosaptia subnuda (Mett. ex Kuhn) Copel.	rbcL	KY099850	FTOL v1.7
Polypodiaceae	Prosaptia urceolaris (Hayata) Copel.	atpB	OQ702749	FTOL v1.7
Polypodiaceae	Prosaptia urceolaris (Hayata) Copel.	rps4	OQ717265	FTOL v1.7
Polypodiaceae	Prosaptia urceolaris (Hayata) Copel.	rps4-trnS	OQ717265	FTOL v1.7
Polypodiaceae	Prosaptia venulosa (Blume) M. G. Price	atpB	KM218853	FTOL v1.7
Polypodiaceae	Prosaptia venulosa (Blume) M. G. Price	rbcL	KM218769	FTOL v1.7
Polypodiaceae	Prosaptia venulosa (Blume) M. G. Price	rps4-trnS	KM106182	FTOL v1.7
Polypodiaceae	Prosaptia venulosa (Blume) M. G. Price	trnL-trnF	KM106086	FTOL v1.7
Polypodiaceae	Pyrrosia abbreviata (Zoll. & Moritz) Tagawa	rbcL	KY632745	FTOL v1.7
Polypodiaceae	Pyrrosia adnascens (Sw.) Ching	plastome	OR051897	FTOL v1.7
Polypodiaceae	Pyrrosia albicans (Blume) Ching	rbcL	KY931040	FTOL v1.7
Polypodiaceae	Pyrrosia albicans (Blume) Ching	rps4	KY931191	FTOL v1.7
Polypodiaceae	Pyrrosia albicans (Blume) Ching	rps4-trnS	KY931191	FTOL v1.7
Polypodiaceae	Pyrrosia albicans (Blume) Ching	trnL-trnF	KY931341	FTOL v1.7
Polypodiaceae	Pyrrosia angustata (Sw.) Ching	plastome	MW876358	FTOL v1.7
Polypodiaceae	Pyrrosia angustissima (Giesenh. ex Diels) Tagawa & K.	plastome	MT210543	FTOL v1.7
Polypodiaceae	Pyrrosia assimilis (Baker) Ching	plastome	MN617019	FTOL v1.7
Polypodiaceae	Pyrrosia bonii (Christ) Ching	plastome	MH352390	FTOL v1.7
Polypodiaceae	Pyrrosia boothii (Hook.) Ching	rbcL	KY632775	FTOL v1.7
Polypodiaceae	Pyrrosia christii (Giesenh.) Ching	rbcL	DQ164465	FTOL v1.7
Polypodiaceae	Pyrrosia christii (Giesenh.) Ching	rps4	KY931210	FTOL v1.7
Polypodiaceae	Pyrrosia christii (Giesenh.) Ching	rps4-trnS	DQ164496	FTOL v1.7
Polypodiaceae	Pyrrosia christii (Giesenh.) Ching	trnL-trnF	KY931355	FTOL v1.7
Polypodiaceae	Pyrrosia confluens (R. Br.) Ching	atpB	KY064551	FTOL v1.7
Polypodiaceae	Pyrrosia confluens (R. Br.) Ching	rbcL	MT226743	FTOL v1.7
Polypodiaceae	Pyrrosia confluens (R. Br.) Ching	rps4	MT226744	FTOL v1.7
Polypodiaceae	Pyrrosia confluens (R. Br.) Ching	rps4-trnS	KY064576	FTOL v1.7
Polypodiaceae	Pyrrosia costata (C. Presl ex Bedd.) Tagawa & K. Iwats.	plastome	MT130646	FTOL v1.7
Polypodiaceae	Pyrrosia davidii (Baker) Ching	rbcL	KY931067	FTOL v1.7
Polypodiaceae	Pyrrosia davidii (Baker) Ching	rps4	KY931218	FTOL v1.7
Polypodiaceae	Pyrrosia davidii (Baker) Ching	rps4-trnS	KY931218	FTOL v1.7
Polypodiaceae	Pyrrosia davidii (Baker) Ching	trnL-trnF	KY931358	FTOL v1.7
Polypodiaceae	Pyrrosia distichocarpa (Mett.) K. H. Shing	atpB	KY064549	FTOL v1.7
Polypodiaceae	Pyrrosia distichocarpa (Mett.) K. H. Shing	rbcL	KY064520	FTOL v1.7
Polypodiaceae	Pyrrosia distichocarpa (Mett.) K. H. Shing	rps4-trnS	KY064572	FTOL v1.7
Polypodiaceae	Pyrrosia drakeana (Franch.) Ching	plastome	MT210542	FTOL v1.7
Polypodiaceae	Pyrrosia eberhardtii (Christ) Ching	rbcL	KY931121	FTOL v1.7
Polypodiaceae	Pyrrosia eberhardtii (Christ) Ching	rps4	KY931273	FTOL v1.7
Polypodiaceae	Pyrrosia eberhardtii (Christ) Ching	rps4-trnS	KY931273	FTOL v1.7
Polypodiaceae	Pyrrosia eberhardtii (Christ) Ching	trnL-trnF	KY931402	FTOL v1.7
Polypodiaceae	Pyrrosia eleagnifolia (Bory) Hovenkamp	rbcL	DQ642166	FTOL v1.7
Polypodiaceae	Pyrrosia eleagnifolia (Bory) Hovenkamp	rps4	DQ642205	FTOL v1.7
Polypodiaceae	Pyrrosia eleagnifolia (Bory) Hovenkamp	rps4-trnS	DQ642205	FTOL v1.7
Polypodiaceae	Pyrrosia eleagnifolia (Bory) Hovenkamp	trnL-trnF	DQ642251	FTOL v1.7
Polypodiaceae	Pyrrosia ensata Ching & K. H. Shing	atpB	KY064548	FTOL v1.7
Polypodiaceae	Pyrrosia ensata Ching & K. H. Shing	rbcL	KY064523	FTOL v1.7
Polypodiaceae	Pyrrosia ensata Ching & K. H. Shing	rps4-trnS	KY064571	FTOL v1.7
Polypodiaceae	Pyrrosia fallax (Alderw.) M. G. Price	rbcL	KY931081	FTOL v1.7
Polypodiaceae	Pyrrosia fallax (Alderw.) M. G. Price	rps4	KY931232	FTOL v1.7
Polypodiaceae	Pyrrosia fallax (Alderw.) M. G. Price	rps4-trnS	KY931232	FTOL v1.7
Polypodiaceae	Pyrrosia fallax (Alderw.) M. G. Price	trnL-trnF	KY931368	FTOL v1.7
Polypodiaceae	Pyrrosia flocculosa (D. Don) Ching	rbcL	KY931047	FTOL v1.7
Polypodiaceae	Pyrrosia flocculosa (D. Don) Ching	rps4	KY931198	FTOL v1.7
Polypodiaceae	Pyrrosia flocculosa (D. Don) Ching	rps4-trnS	KY931198	FTOL v1.7
Polypodiaceae	Pyrrosia flocculosa (D. Don) Ching	trnL-trnF	KY931346	FTOL v1.7
Polypodiaceae	Pyrrosia foveolata (Alston) C. V. Morton	rbcL	DQ642167	FTOL v1.7
Polypodiaceae	Pyrrosia foveolata (Alston) C. V. Morton	rps4	DQ642206	FTOL v1.7
Polypodiaceae	Pyrrosia foveolata (Alston) C. V. Morton	rps4-trnS	DQ642206	FTOL v1.7
Polypodiaceae	Pyrrosia foveolata (Alston) C. V. Morton	trnL-trnF	DQ642252	FTOL v1.7
Polypodiaceae	Pyrrosia glabra (Desv.) Fraser-Jenk.	rbcL	KY931116	FTOL v1.7
Polypodiaceae	Pyrrosia glabra (Desv.) Fraser-Jenk.	rps4	KY931267	FTOL v1.7
Polypodiaceae	Pyrrosia glabra (Desv.) Fraser-Jenk.	rps4-trnS	KY931267	FTOL v1.7
Polypodiaceae	Pyrrosia glabra (Desv.) Fraser-Jenk.	trnL-trnF	KY931398	FTOL v1.7
Polypodiaceae	Pyrrosia hastata (Houtt.) Ching	plastome	OR051898	FTOL v1.7
Polypodiaceae	Pyrrosia heteractis (Mett. ex Kuhn) Ching	plastome	MT130592	FTOL v1.7
Polypodiaceae	Pyrrosia heterophylla (L.) M. G. Price	rbcL	ON453836	FTOL v1.7
Polypodiaceae	Pyrrosia kinabaluensis Hovenkamp	rbcL	KY931085	FTOL v1.7
Polypodiaceae	Pyrrosia kinabaluensis Hovenkamp	rps4	KY931237	FTOL v1.7
Polypodiaceae	Pyrrosia kinabaluensis Hovenkamp	rps4-trnS	KY931237	FTOL v1.7
Polypodiaceae	Pyrrosia kinabaluensis Hovenkamp	trnL-trnF	KY931373	FTOL v1.7
Polypodiaceae	Pyrrosia laevis (J. Sm.) Ching	rbcL	KY632785	FTOL v1.7
Polypodiaceae	Pyrrosia lanceolata (L.) Farw.	rbcL	KY931087	FTOL v1.7
Polypodiaceae	Pyrrosia lanceolata (L.) Farw.	rps4	KY931239	FTOL v1.7
Polypodiaceae	Pyrrosia lanceolata (L.) Farw.	rps4-trnS	KY931239	FTOL v1.7
Polypodiaceae	Pyrrosia lanceolata (L.) Farw.	trnL-trnF	KY931375	FTOL v1.7
Polypodiaceae	Pyrrosia linearifolia (Hook.) Ching	rbcL	KY931090	FTOL v1.7
Polypodiaceae	Pyrrosia linearifolia (Hook.) Ching	rps4	KY931242	FTOL v1.7
Polypodiaceae	Pyrrosia linearifolia (Hook.) Ching	rps4-trnS	KY931242	FTOL v1.7
Polypodiaceae	Pyrrosia linearifolia (Hook.) Ching	trnL-trnF	KY931378	FTOL v1.7
Polypodiaceae	Pyrrosia lingua (Thunb.) Farw.	plastome	MT210540	FTOL v1.7
Polypodiaceae	Pyrrosia longifolia (Burm. fil.) C. V. Morton	rbcL	KY931103	FTOL v1.7
Polypodiaceae	Pyrrosia longifolia (Burm. fil.) C. V. Morton	rps4	KY931254	FTOL v1.7
Polypodiaceae	Pyrrosia longifolia (Burm. fil.) C. V. Morton	rps4-trnS	KY931254	FTOL v1.7
Polypodiaceae	Pyrrosia longifolia (Burm. fil.) C. V. Morton	trnL-trnF	KY931388	FTOL v1.7
Polypodiaceae	Pyrrosia mannii (Giesenh.) Ching	rbcL	KY931105	FTOL v1.7
Polypodiaceae	Pyrrosia mannii (Giesenh.) Ching	rps4	KY931256	FTOL v1.7
Polypodiaceae	Pyrrosia mannii (Giesenh.) Ching	rps4-trnS	KY931256	FTOL v1.7
Polypodiaceae	Pyrrosia mannii (Giesenh.) Ching	trnL-trnF	KY931390	FTOL v1.7
Polypodiaceae	Pyrrosia matsudai (Hayata) Tagawa	rbcL	KY931107	FTOL v1.7
Polypodiaceae	Pyrrosia matsudai (Hayata) Tagawa	rps4	KY931259	FTOL v1.7
Polypodiaceae	Pyrrosia matsudai (Hayata) Tagawa	rps4-trnS	KY931259	FTOL v1.7
Polypodiaceae	Pyrrosia niphoboloides (Luerss.) M. G. Price	rbcL	DQ642168	FTOL v1.7
Polypodiaceae	Pyrrosia niphoboloides (Luerss.) M. G. Price	rps4-trnS	DQ642207	FTOL v1.7
Polypodiaceae	Pyrrosia niphoboloides (Luerss.) M. G. Price	trnL-trnF	DQ642254	FTOL v1.7
Polypodiaceae	Pyrrosia nummulariifolia (Sw.) Ching	atpB	JX103671	FTOL v1.7
Polypodiaceae	Pyrrosia nummulariifolia (Sw.) Ching	rbcL	JX103713	FTOL v1.7
Polypodiaceae	Pyrrosia nummulariifolia (Sw.) Ching	rps4	JX103755	FTOL v1.7
Polypodiaceae	Pyrrosia nummulariifolia (Sw.) Ching	rps4-trnS	JX103755	FTOL v1.7
Polypodiaceae	Pyrrosia nummulariifolia (Sw.) Ching	trnL-trnF	JX103797	FTOL v1.7
Polypodiaceae	Pyrrosia pannosa (Mett. ex Kuhn) Ching	atpB	KY064556	FTOL v1.7
Polypodiaceae	Pyrrosia pannosa (Mett. ex Kuhn) Ching	rbcL	KY064529	FTOL v1.7
Polypodiaceae	Pyrrosia pannosa (Mett. ex Kuhn) Ching	rps4-trnS	KY064577	FTOL v1.7
Polypodiaceae	Pyrrosia penangiana (Hook.) Holttum	plastome	MW876359	FTOL v1.7
Polypodiaceae	Pyrrosia petiolosa (Christ) Ching	plastome	MT210541	FTOL v1.7
Polypodiaceae	Pyrrosia piloselloides (L.) M. G. Price	rbcL	KY931128	FTOL v1.7
Polypodiaceae	Pyrrosia piloselloides (L.) M. G. Price	rps4	KY931281	FTOL v1.7
Polypodiaceae	Pyrrosia piloselloides (L.) M. G. Price	rps4-trnS	KY931281	FTOL v1.7
Polypodiaceae	Pyrrosia piloselloides (L.) M. G. Price	trnL-trnF	KY931408	FTOL v1.7
Polypodiaceae	Pyrrosia polydactyla (Hance) Ching	atpA	EF463844	FTOL v1.7
Polypodiaceae	Pyrrosia polydactyla (Hance) Ching	atpB	EF463511	FTOL v1.7
Polypodiaceae	Pyrrosia polydactyla (Hance) Ching	rbcL	EF463259	FTOL v1.7
Polypodiaceae	Pyrrosia porosa (C. Presl) Hovenkamp	rbcL	KY931135	FTOL v1.7
Polypodiaceae	Pyrrosia porosa (C. Presl) Hovenkamp	rps4	KY931288	FTOL v1.7
Polypodiaceae	Pyrrosia porosa (C. Presl) Hovenkamp	rps4-trnS	KY931288	FTOL v1.7
Polypodiaceae	Pyrrosia porosa (C. Presl) Hovenkamp	trnL-trnF	KY931412	FTOL v1.7
Polypodiaceae	Pyrrosia rasamalae (Racib.) K. H. Shing	rbcL	KY931082	FTOL v1.7
Polypodiaceae	Pyrrosia rasamalae (Racib.) K. H. Shing	rps4	KY931233	FTOL v1.7
Polypodiaceae	Pyrrosia rasamalae (Racib.) K. H. Shing	rps4-trnS	KY931233	FTOL v1.7
Polypodiaceae	Pyrrosia rasamalae (Racib.) K. H. Shing	trnL-trnF	KY931369	FTOL v1.7
Polypodiaceae	Pyrrosia rhodesiana (C. Chr.) Schelpe	rbcL	KY931104	FTOL v1.7
Polypodiaceae	Pyrrosia rhodesiana (C. Chr.) Schelpe	rps4	KY931255	FTOL v1.7
Polypodiaceae	Pyrrosia rhodesiana (C. Chr.) Schelpe	rps4-trnS	KY931255	FTOL v1.7
Polypodiaceae	Pyrrosia rhodesiana (C. Chr.) Schelpe	trnL-trnF	KY931389	FTOL v1.7
Polypodiaceae	Pyrrosia rupestris (R. Br.) Ching	rbcL	AY362558	FTOL v1.7
Polypodiaceae	Pyrrosia samarensis (Mett.) Ching	rbcL	DQ642170	FTOL v1.7
Polypodiaceae	Pyrrosia samarensis (Mett.) Ching	rps4	DQ642209	FTOL v1.7
Polypodiaceae	Pyrrosia samarensis (Mett.) Ching	rps4-trnS	DQ642209	FTOL v1.7
Polypodiaceae	Pyrrosia samarensis (Mett.) Ching	trnL-trnF	DQ642256	FTOL v1.7
Polypodiaceae	Pyrrosia serpens (G. Forst.) Ching	atpA	EF463845	FTOL v1.7
Polypodiaceae	Pyrrosia serpens (G. Forst.) Ching	atpB	EF463512	FTOL v1.7
Polypodiaceae	Pyrrosia serpens (G. Forst.) Ching	rbcL	EF463260	FTOL v1.7
Polypodiaceae	Pyrrosia sheareri (Baker) Ching	plastome	MT130682	FTOL v1.7
Polypodiaceae	Pyrrosia similis Ching	rbcL	KY931147	FTOL v1.7
Polypodiaceae	Pyrrosia similis Ching	rps4	KY931300	FTOL v1.7
Polypodiaceae	Pyrrosia similis Ching	rps4-trnS	KY931300	FTOL v1.7
Polypodiaceae	Pyrrosia similis Ching	trnL-trnF	KY931422	FTOL v1.7
Polypodiaceae	Pyrrosia sphaerosticha (Mett.) Ching	rbcL	KY931152	FTOL v1.7
Polypodiaceae	Pyrrosia sphaerosticha (Mett.) Ching	rps4	KY931304	FTOL v1.7
Polypodiaceae	Pyrrosia sphaerosticha (Mett.) Ching	rps4-trnS	KY931304	FTOL v1.7
Polypodiaceae	Pyrrosia sphaerosticha (Mett.) Ching	trnL-trnF	KY931427	FTOL v1.7
Polypodiaceae	Pyrrosia stenophylla (Bedd.) Ching	rbcL	KY931172	FTOL v1.7
Polypodiaceae	Pyrrosia stenophylla (Bedd.) Ching	rps4	KY931324	FTOL v1.7
Polypodiaceae	Pyrrosia stenophylla (Bedd.) Ching	rps4-trnS	KY931324	FTOL v1.7
Polypodiaceae	Pyrrosia stenophylla (Bedd.) Ching	trnL-trnF	KY931446	FTOL v1.7
Polypodiaceae	Pyrrosia stigmosa (Sw.) Ching	atpB	JX103674	FTOL v1.7
Polypodiaceae	Pyrrosia stigmosa (Sw.) Ching	rbcL	JX103716	FTOL v1.7
Polypodiaceae	Pyrrosia stigmosa (Sw.) Ching	rps4	JX103758	FTOL v1.7
Polypodiaceae	Pyrrosia stigmosa (Sw.) Ching	rps4-trnS	JX103758	FTOL v1.7
Polypodiaceae	Pyrrosia stigmosa (Sw.) Ching	trnL-trnF	JX103800	FTOL v1.7
Polypodiaceae	Pyrrosia stolzii (Hieron.) Schelpe	rbcL	KY931157	FTOL v1.7
Polypodiaceae	Pyrrosia stolzii (Hieron.) Schelpe	rps4	KY931310	FTOL v1.7
Polypodiaceae	Pyrrosia stolzii (Hieron.) Schelpe	rps4-trnS	KY931310	FTOL v1.7
Polypodiaceae	Pyrrosia stolzii (Hieron.) Schelpe	trnL-trnF	KY931433	FTOL v1.7
Polypodiaceae	Pyrrosia subfurfuracea (Hook.) Ching	plastome	MK679765	FTOL v1.7
Polypodiaceae	Rouhania pygmaea (Bory ex Willd.) Li Bing Zhang, X.M	atpB	KY711888	FTOL v1.7
Polypodiaceae	Rouhania pygmaea (Bory ex Willd.) Li Bing Zhang, X.M	rbcL	KY712062	FTOL v1.7
Polypodiaceae	Rouhania pygmaea (Bory ex Willd.) Li Bing Zhang, X.M	rps4-trnS	KY712318	FTOL v1.7
Polypodiaceae	Rouhania pygmaea (Bory ex Willd.) Li Bing Zhang, X.M	trnL-trnF	KY711647	FTOL v1.7
Polypodiaceae	Scleroglossum pusillum (Blume) Alderw.	atpB	KY711911	FTOL v1.7
Polypodiaceae	Scleroglossum pusillum (Blume) Alderw.	rbcL	KY712079	FTOL v1.7
Polypodiaceae	Scleroglossum pusillum (Blume) Alderw.	rps4-trnS	KY712404	FTOL v1.7
Polypodiaceae	Scleroglossum pusillum (Blume) Alderw.	trnL-trnF	KY711741	FTOL v1.7
Polypodiaceae	Scleroglossum pyxidatum Alderw.	atpB	OQ702674	FTOL v1.7
Polypodiaceae	Scleroglossum pyxidatum Alderw.	rps4	OQ717185	FTOL v1.7
Polypodiaceae	Scleroglossum pyxidatum Alderw.	rps4-trnS	OQ717185	FTOL v1.7
Polypodiaceae	Scleroglossum pyxidatum Alderw.	trnL-trnF	OQ717172	FTOL v1.7
Polypodiaceae	Scleroglossum sulcatum (Mett. ex Kuhn) Alderw.	atpB	AY459498	FTOL v1.7
Polypodiaceae	Scleroglossum sulcatum (Mett. ex Kuhn) Alderw.	rbcL	AY460665	FTOL v1.7
Polypodiaceae	Scleroglossum wooroonooran (Bailey) C. Chr.	atpB	KM218835	FTOL v1.7
Polypodiaceae	Scleroglossum wooroonooran (Bailey) C. Chr.	rbcL	KM218809	FTOL v1.7
Polypodiaceae	Scleroglossum wooroonooran (Bailey) C. Chr.	rps4-trnS	KM106189	FTOL v1.7
Polypodiaceae	Scleroglossum wooroonooran (Bailey) C. Chr.	trnL-trnF	KM106091	FTOL v1.7
Polypodiaceae	Selliguea albidosquamata (Blume) Parris	atpA	KT289108	FTOL v1.7
Polypodiaceae	Selliguea albidosquamata (Blume) Parris	atpB	KT289124	FTOL v1.7
Polypodiaceae	Selliguea albidosquamata (Blume) Parris	rbcL	KT289200	FTOL v1.7

Polypodiaceae	Selliguea albidosquamata (Blume) Parris	trnL-trnF	KT289338	FTOL v1.7
Polypodiaceae	Selliguea brachypodia (Copel.) X. C. Zhang & L. J. He	rbcL	AY362557	FTOL v1.7
Polypodiaceae	Selliguea brachypodia (Copel.) X. C. Zhang & L. J. He	rps4-trnS	AY362621	FTOL v1.7
Polypodiaceae	Selliguea capitellata (Wall.) X. C. Zhang & L. J. He	plastome	MW876361	FTOL v1.7
Polypodiaceae	Selliguea caudiformis (Blume) J. Sm.	atpA	KT289109	FTOL v1.7
Polypodiaceae	Selliguea caudiformis (Blume) J. Sm.	atpB	KT289125	FTOL v1.7
Polypodiaceae	Selliguea caudiformis (Blume) J. Sm.	rbcL	KT289201	FTOL v1.7
Polypodiaceae	Selliguea caudiformis (Blume) J. Sm.	trnL-trnF	KT289340	FTOL v1.7
Polypodiaceae	Selliguea chenopus (Christ) S. G. Lu, Hovenkamp & M.	plastome	MW876366	FTOL v1.7
Polypodiaceae	Selliguea chrysotricha (C. Chr.) Fraser-Jenk.	plastome	OM419376	FTOL v1.7
Polypodiaceae	Selliguea conjuncta (Ching) S. G. Lu, Hovenkamp & M.	rbcL	JQ685397	FTOL v1.7
Polypodiaceae	Selliguea conjuncta (Ching) S. G. Lu, Hovenkamp & M.	rps4-trnS	JQ685461	FTOL v1.7
Polypodiaceae	Selliguea conjuncta (Ching) S. G. Lu, Hovenkamp & M.	trnL-trnF	JQ700474	FTOL v1.7
Polypodiaceae	Selliguea conmixta (Ching) S. G. Lu, Hovenkamp & M. (	rbcL	JQ685398	FTOL v1.7
Polypodiaceae	Selliguea conmixta (Ching) S. G. Lu, Hovenkamp & M. (	rps4-trnS	JQ685462	FTOL v1.7
Polypodiaceae	Selliguea conmixta (Ching) S. G. Lu, Hovenkamp & M. (	trnL-trnF	JQ700475	FTOL v1.7
Polypodiaceae	Selliguea cyrtomioides (S. G. Lu & C. D. Xu) X. C. Zhang	rbcL	JQ685377	FTOL v1.7
Polypodiaceae	Selliguea cyrtomioides (S. G. Lu & C. D. Xu) X. C. Zhang	rps4-trnS	JQ685441	FTOL v1.7
Polypodiaceae	Selliguea cyrtomioides (S. G. Lu & C. D. Xu) X. C. Zhang	trnL-trnF	JQ700454	FTOL v1.7
Polypodiaceae	Selliguea dactylina (Christ) S. G. Lu, Hovenkamp & M. (	rbcL	JQ685403	FTOL v1.7
Polypodiaceae	Selliguea dactylina (Christ) S. G. Lu, Hovenkamp & M. (	rps4	JQ685467	FTOL v1.7
Polypodiaceae	Selliguea dactylina (Christ) S. G. Lu, Hovenkamp & M. (	rps4-trnS	JQ685467	FTOL v1.7
Polypodiaceae	Selliguea dactylina (Christ) S. G. Lu, Hovenkamp & M. (	trnL-trnF	JQ700480	FTOL v1.7
Polypodiaceae	Selliguea digitata (Ching) S. G. Lu, Hovenkamp & M. G.	trnL-trnF	KT289307	FTOL v1.7
Polypodiaceae	Selliguea enervis (Cav.) Ching	atpA	KT289110	FTOL v1.7
Polypodiaceae	Selliguea enervis (Cav.) Ching	atpB	KT289126	FTOL v1.7
Polypodiaceae	Selliguea enervis (Cav.) Ching	rbcL	KT289202	FTOL v1.7
Polypodiaceae	Selliguea enervis (Cav.) Ching	trnL-trnF	KT289341	FTOL v1.7
Polypodiaceae	Selliguea engleri (Luerss.) Fraser-Jenk.	rbcL	AB575248	FTOL v1.7
Polypodiaceae	Selliguea feei Bory	rbcL	AY529170	FTOL v1.7
Polypodiaceae	Selliguea feei Bory	rps4	AY529192	FTOL v1.7
Polypodiaceae	Selliguea feei Bory	rps4-trnS	AY529192	FTOL v1.7
Polypodiaceae	Selliguea feeoides Copel.	rbcL	MT657616	FTOL v1.7
Polypodiaceae	Selliguea glauca (J. Sm. ex Brack.) Hovenkamp	atpA	KT289111	FTOL v1.7
Polypodiaceae	Selliguea glauca (J. Sm. ex Brack.) Hovenkamp	atpB	KT289127	FTOL v1.7
Polypodiaceae	Selliguea glauca (J. Sm. ex Brack.) Hovenkamp	trnL-trnF	KT289342	FTOL v1.7
Polypodiaceae	Selliguea griffithiana (Hook.) Fraser-Jenk.	plastome	MW876369	FTOL v1.7
Polypodiaceae	Selliguea hastata (Thunb.) Fraser-Jenk.	plastome	OM419379	FTOL v1.7
Polypodiaceae	Selliguea hellwigii (Diels) Hovenkamp	rbcL	EU128501	FTOL v1.7
Polypodiaceae	Selliguea hellwigii (Diels) Hovenkamp	rps4	EU128508	FTOL v1.7
Polypodiaceae	Selliguea hellwigii (Diels) Hovenkamp	rps4-trnS	EU128508	FTOL v1.7
Polypodiaceae	Selliguea hellwigii (Diels) Hovenkamp	trnL-trnF	EU128518	FTOL v1.7
Polypodiaceae	Selliguea heterocarpa (Blume) Blume	plastome	MW876362	FTOL v1.7
Polypodiaceae	Selliguea incisocrenata (Ching ex W. M. Chu & S. G. Lu	rbcL	KT289180	FTOL v1.7
Polypodiaceae	Selliguea incisocrenata (Ching ex W. M. Chu & S. G. Lu	trnL-trnF	KT289321	FTOL v1.7
Polypodiaceae	Selliguea laciniata (C. Presl) Hovenkamp	rbcL	AY529171	FTOL v1.7
Polypodiaceae	Selliguea laciniata (C. Presl) Hovenkamp	rps4	AY529193	FTOL v1.7
Polypodiaceae	Selliguea laciniata (C. Presl) Hovenkamp	rps4-trnS	AY529193	FTOL v1.7
Polypodiaceae	Selliguea laciniata (C. Presl) Hovenkamp	trnL-trnF	AY529484	FTOL v1.7
Polypodiaceae	Selliguea lagunensis (Christ) Hovenkamp	rbcL	KT289203	FTOL v1.7
Polypodiaceae	Selliguea lagunensis (Christ) Hovenkamp	trnL-trnF	KT289343	FTOL v1.7
Polypodiaceae	Selliguea lanceola (Mett.) E. Fourn.	rbcL	AY459173	FTOL v1.7
Polypodiaceae	Selliguea lanceola (Mett.) E. Fourn.	rps4	AY459186	FTOL v1.7
Polypodiaceae	Selliguea lanceola (Mett.) E. Fourn.	rps4-trnS	AY459186	FTOL v1.7
Polypodiaceae	Selliguea lanceola (Mett.) E. Fourn.	trnL-trnF	AY459181	FTOL v1.7
Polypodiaceae	Selliguea lanceolata (Sw.) Fée	atpA	EF463847	FTOL v1.7
Polypodiaceae	Selliguea lanceolata (Sw.) Fée	atpB	EF463513	FTOL v1.7
Polypodiaceae	Selliguea lanceolata (Sw.) Fée	rbcL	EF463261	FTOL v1.7
Polypodiaceae	Selliguea lateritia (Baker) Hovenkamp	rbcL	EU128502	FTOL v1.7
Polypodiaceae	Selliguea lateritia (Baker) Hovenkamp	rps4	EU128509	FTOL v1.7
Polypodiaceae	Selliguea lateritia (Baker) Hovenkamp	rps4-trnS	EU128509	FTOL v1.7
Polypodiaceae	Selliguea lateritia (Baker) Hovenkamp	trnL-trnF	EU128519	FTOL v1.7
Polypodiaceae	Selliguea lehmannii (Mett.) X. C. Zhang & L. J. He	plastome	MT130554	FTOL v1.7
Polypodiaceae	Selliguea majoensis (C. Chr.) Fraser-Jenk.	rbcL	JQ685420	FTOL v1.7
Polypodiaceae	Selliguea metacoela (Alderw.) Parris	plastome	MW876365	FTOL v1.7
Polypodiaceae	Selliguea moulmeinensis (Bedd.) X. C. Zhang & L. J. He	plastome	MW876364	FTOL v1.7
Polypodiaceae	Selliguea oxyloba (Wall. ex Kunze) Fraser-Jenk.	plastome	MT130663	FTOL v1.7
Polypodiaceae	Selliguea pianmaensis (W. M. Chu) S. G. Lu, Hovenkam	rps4-trnS	JQ685490	FTOL v1.7
Polypodiaceae	Selliguea pianmaensis (W. M. Chu) S. G. Lu, Hovenkam	trnL-trnF	JQ700495	FTOL v1.7
Polypodiaceae	Selliguea plantaginea Brack.	atpA	EF463848	FTOL v1.7
Polypodiaceae	Selliguea plantaginea Brack.	atpB	EF463514	FTOL v1.7
Polypodiaceae	Selliguea plantaginea Brack.	rbcL	EF463262	FTOL v1.7
Polypodiaceae	Selliguea platyphylla (Sw.) Ching	rbcL	KT289206	FTOL v1.7
Polypodiaceae	Selliguea platyphylla (Sw.) Ching	trnL-trnF	KT289346	FTOL v1.7
Polypodiaceae	Selliguea pyrolifolia (Goldm.) Hovenkamp	atpA	KT289112	FTOL v1.7
Polypodiaceae	Selliguea pyrolifolia (Goldm.) Hovenkamp	atpB	KT289128	FTOL v1.7
Polypodiaceae	Selliguea pyrolifolia (Goldm.) Hovenkamp	rbcL	KT289207	FTOL v1.7
Polypodiaceae	Selliguea pyrolifolia (Goldm.) Hovenkamp	trnL-trnF	KT289347	FTOL v1.7
Polypodiaceae	Selliguea rhynchophylla (Hook.) Fraser-Jenk.	atpA	KT289107	FTOL v1.7
Polypodiaceae	Selliguea rhynchophylla (Hook.) Fraser-Jenk.	atpB	KT289123	FTOL v1.7
Polypodiaceae	Selliguea rhynchophylla (Hook.) Fraser-Jenk.	rbcL	JQ685427	FTOL v1.7
Polypodiaceae	Selliguea rhynchophylla (Hook.) Fraser-Jenk.	rps4-trnS	JQ685492	FTOL v1.7
Polypodiaceae	Selliguea rhynchophylla (Hook.) Fraser-Jenk.	trnL-trnF	KT289329	FTOL v1.7
Polypodiaceae	Selliguea senanensis (Maxim.) S. G. Lu, Hovenkamp &	rbcL	KT289191	FTOL v1.7
Polypodiaceae	Selliguea senanensis (Maxim.) S. G. Lu, Hovenkamp &	trnL-trnF	KT289330	FTOL v1.7
Polypodiaceae	Selliguea soridens (Hook.) Hovenkamp	atpA	KT289113	FTOL v1.7
Polypodiaceae	Selliguea soridens (Hook.) Hovenkamp	atpB	KT289129	FTOL v1.7
Polypodiaceae	Selliguea soridens (Hook.) Hovenkamp	rbcL	KT289208	FTOL v1.7
Polypodiaceae	Selliguea soridens (Hook.) Hovenkamp	trnL-trnF	KT289348	FTOL v1.7
Polypodiaceae	Selliguea stenophylla (Blume) Parris	rbcL	KT289209	FTOL v1.7
Polypodiaceae	Selliguea stenophylla (Blume) Parris	trnL-trnF	KT289349	FTOL v1.7
Polypodiaceae	Selliguea stenosquamis Hovenkamp	atpA	KT289114	FTOL v1.7
Polypodiaceae	Selliguea stenosquamis Hovenkamp	atpB	KT289130	FTOL v1.7
Polypodiaceae	Selliguea stenosquamis Hovenkamp	rbcL	KT289210	FTOL v1.7
Polypodiaceae	Selliguea stenosquamis Hovenkamp	trnL-trnF	KT289350	FTOL v1.7
Polypodiaceae	Selliguea subsparsa (Baker) Hovenkamp	rps4	AY362620	FTOL v1.7
Polypodiaceae	Selliguea taeniata (Sw.) Parris	plastome	MW876349	FTOL v1.7
Polypodiaceae	Selliguea tatsienensis (Franch. & Bureau) X. C. Zhang &	plastome	MT130544	FTOL v1.7
Polypodiaceae	Selliguea tomentosa (W. M. Chu) X. C. Zhang & L. J. He	atpA	KT289101	FTOL v1.7
Polypodiaceae	Selliguea tomentosa (W. M. Chu) X. C. Zhang & L. J. He	atpB	KT289117	FTOL v1.7
Polypodiaceae	Selliguea tomentosa (W. M. Chu) X. C. Zhang & L. J. He	rbcL	KT289149	FTOL v1.7
Polypodiaceae	Selliguea tomentosa (W. M. Chu) X. C. Zhang & L. J. He	trnL-trnF	KT289290	FTOL v1.7
Polypodiaceae	Selliguea triloba (Houtt.) M. G. Price	rbcL	KT289214	FTOL v1.7
Polypodiaceae	Selliguea triloba (Houtt.) M. G. Price	trnL-trnF	KT289354	FTOL v1.7
Polypodiaceae	Selliguea trisecta (Baker) Fraser-Jenk.	rbcL	JQ685438	FTOL v1.7
Polypodiaceae	Selliguea wardii (Clarke) X. C. Zhang & L. J. He	rbcL	KT289158	FTOL v1.7
Polypodiaceae	Selliguea wardii (Clarke) X. C. Zhang & L. J. He	trnL-trnF	KT289299	FTOL v1.7
Polypodiaceae	Selliguea whitfordii (Copel.) Hovenkamp	atpA	KT289116	FTOL v1.7
Polypodiaceae	Selliguea whitfordii (Copel.) Hovenkamp	atpB	KT289132	FTOL v1.7
Polypodiaceae	Selliguea whitfordii (Copel.) Hovenkamp	rbcL	KT289216	FTOL v1.7
Polypodiaceae	Selliguea whitfordii (Copel.) Hovenkamp	trnL-trnF	KT289355	FTOL v1.7
Polypodiaceae	Selliguea wusugongii Liang Zhang, X. P. Fan & Li Bing Z	rbcL	MW462198	FTOL v1.7
Polypodiaceae	Selliguea wusugongii Liang Zhang, X. P. Fan & Li Bing Z	rps4	MW462197	FTOL v1.7
Polypodiaceae	Selliguea wusugongii Liang Zhang, X. P. Fan & Li Bing Z	rps4-trnS	MW462197	FTOL v1.7
Polypodiaceae	Selliguea wusugongii Liang Zhang, X. P. Fan & Li Bing Z	trnL-trnF	MW462196	FTOL v1.7
Polypodiaceae	Selliguea yakuinsularis (Masam.) H. Ohashi & K. Ohash	rbcL	AB575251	FTOL v1.7
Polypodiaceae	Selliguea yakushimensis (Makino) Fraser-Jenk.	plastome	MN623352	FTOL v1.7
Polypodiaceae	Serpocaulon adnatum (Kunze ex Klotzsch) A. R. Sm.	rbcL	AY362593	FTOL v1.7
Polypodiaceae	Serpocaulon adnatum (Kunze ex Klotzsch) A. R. Sm.	rps4-trnS	AY362666	FTOL v1.7
Polypodiaceae	Serpocaulon appressum (Copel.) A. R. Sm.	rbcL	DQ151905	FTOL v1.7
Polypodiaceae	Serpocaulon appressum (Copel.) A. R. Sm.	rps4	DQ151930	FTOL v1.7
Polypodiaceae	Serpocaulon appressum (Copel.) A. R. Sm.	rps4-trnS	DQ151930	FTOL v1.7
Polypodiaceae	Serpocaulon appressum (Copel.) A. R. Sm.	trnL-trnF	DQ151957	FTOL v1.7
Polypodiaceae	Serpocaulon attenuatum (C. Presl) A. R. Sm.	rbcL	DQ151912	FTOL v1.7
Polypodiaceae	Serpocaulon attenuatum (C. Presl) A. R. Sm.	rps4	DQ151937	FTOL v1.7
Polypodiaceae	Serpocaulon attenuatum (C. Presl) A. R. Sm.	rps4-trnS	DQ151937	FTOL v1.7
Polypodiaceae	Serpocaulon attenuatum (C. Presl) A. R. Sm.	trnL-trnF	DQ151964	FTOL v1.7
Polypodiaceae	Serpocaulon caceresii (Sodiro) A. R. Sm.	rbcL	EF551066	FTOL v1.7
Polypodiaceae	Serpocaulon caceresii (Sodiro) A. R. Sm.	rps4	EF551082	FTOL v1.7
Polypodiaceae	Serpocaulon caceresii (Sodiro) A. R. Sm.	rps4-trnS	EF551082	FTOL v1.7
Polypodiaceae	Serpocaulon caceresii (Sodiro) A. R. Sm.	trnL-trnF	EF551120	FTOL v1.7
Polypodiaceae	Serpocaulon catharinae (Langsd. & Fisch.) A. R. Sm.	rbcL	EF551068	FTOL v1.7
Polypodiaceae	Serpocaulon catharinae (Langsd. & Fisch.) A. R. Sm.	rps4	EF551086	FTOL v1.7
Polypodiaceae	Serpocaulon catharinae (Langsd. & Fisch.) A. R. Sm.	rps4-trnS	EF551086	FTOL v1.7
Polypodiaceae	Serpocaulon catharinae (Langsd. & Fisch.) A. R. Sm.	trnL-trnF	EF551124	FTOL v1.7
Polypodiaceae	Serpocaulon crystalloneuron (Rosenst.) A. R. Sm.	rbcL	DQ151906	FTOL v1.7
Polypodiaceae	Serpocaulon dasypleuron (Kunze) A. R. Sm.	rps4	EF551089	FTOL v1.7
Polypodiaceae	Serpocaulon dasypleuron (Kunze) A. R. Sm.	rps4-trnS	EF551089	FTOL v1.7
Polypodiaceae	Serpocaulon dasypleuron (Kunze) A. R. Sm.	trnL-trnF	EF551127	FTOL v1.7
Polypodiaceae	Serpocaulon dissimile (L.) A. R. Sm.	rbcL	DQ151908	FTOL v1.7
Polypodiaceae	Serpocaulon dissimile (L.) A. R. Sm.	rps4	EF551090	FTOL v1.7
Polypodiaceae	Serpocaulon dissimile (L.) A. R. Sm.	rps4-trnS	DQ151933	FTOL v1.7
Polypodiaceae	Serpocaulon dissimile (L.) A. R. Sm.	trnL-trnF	DQ151960	FTOL v1.7
Polypodiaceae	Serpocaulon eleutherophlebium (Fée) A. R. Sm.	trnL-trnF	DQ151983	FTOL v1.7
Polypodiaceae	Serpocaulon falcaria (Kunze) A. R. Sm.	rbcL	EF551069	FTOL v1.7
Polypodiaceae	Serpocaulon falcaria (Kunze) A. R. Sm.	rps4	EF551093	FTOL v1.7
Polypodiaceae	Serpocaulon falcaria (Kunze) A. R. Sm.	rps4-trnS	EF551093	FTOL v1.7
Polypodiaceae	Serpocaulon falcaria (Kunze) A. R. Sm.	trnL-trnF	EF551131	FTOL v1.7
Polypodiaceae	Serpocaulon fraxinifolium (Jacq.) A. R. Sm.	plastome	MW876370	FTOL v1.7
Polypodiaceae	Serpocaulon funckii (Mett.) A. R. Sm.	rbcL	AY362603	FTOL v1.7
Polypodiaceae	Serpocaulon funckii (Mett.) A. R. Sm.	rps4-trnS	AY362675	FTOL v1.7
Polypodiaceae	Serpocaulon gilliesii (C. Chr.) A. R. Sm.	rbcL	DQ151911	FTOL v1.7
Polypodiaceae	Serpocaulon gilliesii (C. Chr.) A. R. Sm.	rps4	DQ151936	FTOL v1.7
Polypodiaceae	Serpocaulon gilliesii (C. Chr.) A. R. Sm.	rps4-trnS	DQ151936	FTOL v1.7
Polypodiaceae	Serpocaulon gilliesii (C. Chr.) A. R. Sm.	trnL-trnF	DQ151963	FTOL v1.7
Polypodiaceae	Serpocaulon intricatum (M. Kessler & A. R. Sm.) A. R. S	rbcL	DQ151919	FTOL v1.7
Polypodiaceae	Serpocaulon intricatum (M. Kessler & A. R. Sm.) A. R. S	trnL-trnF	DQ151967	FTOL v1.7
Polypodiaceae	Serpocaulon lasiopus (Klotzsch) A. R. Sm.	rbcL	EF551072	FTOL v1.7
Polypodiaceae	Serpocaulon lasiopus (Klotzsch) A. R. Sm.	rps4	EF551100	FTOL v1.7
Polypodiaceae	Serpocaulon lasiopus (Klotzsch) A. R. Sm.	rps4-trnS	EF551100	FTOL v1.7
Polypodiaceae	Serpocaulon lasiopus (Klotzsch) A. R. Sm.	trnL-trnF	EF551138	FTOL v1.7
Polypodiaceae	Serpocaulon latipes (Langsd. & L. Fisch.) A. R. Sm.	rbcL	DQ151915	FTOL v1.7
Polypodiaceae	Serpocaulon latissimum (R. C. Moran & B. ?llg.) A. R. S	rbcL	DQ151916	FTOL v1.7
Polypodiaceae	Serpocaulon latissimum (R. C. Moran & B. ?llg.) A. R. S	rps4	DQ151941	FTOL v1.7
Polypodiaceae	Serpocaulon latissimum (R. C. Moran & B. ?llg.) A. R. S	rps4-trnS	DQ151941	FTOL v1.7
Polypodiaceae	Serpocaulon latissimum (R. C. Moran & B. ?llg.) A. R. S	trnL-trnF	DQ151968	FTOL v1.7
Polypodiaceae	Serpocaulon levigatum (Cav.) A. R. Sm.	plastome	MW876371	FTOL v1.7
Polypodiaceae	Serpocaulon loriceum (L.) A. R. Sm.	plastome	MW876372	FTOL v1.7
Polypodiaceae	Serpocaulon loriciforme (Rosenst.) A. R. Sm.	rbcL	EF551075	FTOL v1.7
Polypodiaceae	Serpocaulon loriciforme (Rosenst.) A. R. Sm.	rps4	EF551105	FTOL v1.7
Polypodiaceae	Serpocaulon loriciforme (Rosenst.) A. R. Sm.	rps4-trnS	EF551105	FTOL v1.7
Polypodiaceae	Serpocaulon loriciforme (Rosenst.) A. R. Sm.	trnL-trnF	EF551143	FTOL v1.7
Polypodiaceae	Serpocaulon maritimum (Hieron.) A. R. Sm.	rbcL	DQ151918	FTOL v1.7
Polypodiaceae	Serpocaulon maritimum (Hieron.) A. R. Sm.	rps4	DQ151943	FTOL v1.7
Polypodiaceae	Serpocaulon maritimum (Hieron.) A. R. Sm.	rps4-trnS	DQ151943	FTOL v1.7
Polypodiaceae	Serpocaulon maritimum (Hieron.) A. R. Sm.	trnL-trnF	DQ151970	FTOL v1.7
Polypodiaceae	Serpocaulon menisciifolium (Langsd. & Fisch.) A. R. Sm	rbcL	EF551076	FTOL v1.7
Polypodiaceae	Serpocaulon menisciifolium (Langsd. & Fisch.) A. R. Sm	rps4	EF551108	FTOL v1.7
Polypodiaceae	Serpocaulon menisciifolium (Langsd. & Fisch.) A. R. Sm	rps4-trnS	EF551108	FTOL v1.7
Polypodiaceae	Serpocaulon menisciifolium (Langsd. & Fisch.) A. R. Sm	trnL-trnF	EF551146	FTOL v1.7
Polypodiaceae	Serpocaulon patentissimum (Mett. ex Kuhn) A. R. Sm.	rbcL	AY362602	FTOL v1.7
Polypodiaceae	Serpocaulon patentissimum (Mett. ex Kuhn) A. R. Sm.	rps4-trnS	AY362674	FTOL v1.7
Polypodiaceae	Serpocaulon polystichum (Link) A. R. Sm.	rbcL	EF551077	FTOL v1.7
Polypodiaceae	Serpocaulon polystichum (Link) A. R. Sm.	rps4	EF551109	FTOL v1.7
Polypodiaceae	Serpocaulon polystichum (Link) A. R. Sm.	rps4-trnS	EF551109	FTOL v1.7
Polypodiaceae	Serpocaulon polystichum (Link) A. R. Sm.	trnL-trnF	EF551147	FTOL v1.7
Polypodiaceae	Serpocaulon ptilorhizon (Christ) A. R. Sm.	rbcL	AY362611	FTOL v1.7

Polypodiaceae	Serpocaulon richardii (Klotzsch) A. R. Sm.	rbcL	DQ227291	FTOL v1.7
Polypodiaceae	Serpocaulon richardii (Klotzsch) A. R. Sm.	rps4	DQ151929	FTOL v1.7
Polypodiaceae	Serpocaulon richardii (Klotzsch) A. R. Sm.	rps4-trnS	DQ151929	FTOL v1.7
Polypodiaceae	Serpocaulon richardii (Klotzsch) A. R. Sm.	trnL-trnF	DQ151956	FTOL v1.7
Polypodiaceae	Serpocaulon sessilifolium (Desv.) A. R. Sm.	rbcL	DQ151924	FTOL v1.7
Polypodiaceae	Serpocaulon sessilifolium (Desv.) A. R. Sm.	rps4	EF551112	FTOL v1.7
Polypodiaceae	Serpocaulon sessilifolium (Desv.) A. R. Sm.	rps4-trnS	EF551112	FTOL v1.7
Polypodiaceae	Serpocaulon sessilifolium (Desv.) A. R. Sm.	trnL-trnF	DQ151976	FTOL v1.7
Polypodiaceae	Serpocaulon silvulae (M. Kessler & A. R. Sm.) A. R. Sm	rbcL	DQ151925	FTOL v1.7
Polypodiaceae	Serpocaulon silvulae (M. Kessler & A. R. Sm.) A. R. Sm	rps4	DQ151952	FTOL v1.7
Polypodiaceae	Serpocaulon silvulae (M. Kessler & A. R. Sm.) A. R. Sm	rps4-trnS	DQ151952	FTOL v1.7
Polypodiaceae	Serpocaulon silvulae (M. Kessler & A. R. Sm.) A. R. Sm	trnL-trnF	DQ151979	FTOL v1.7
Polypodiaceae	Serpocaulon subandinum (Sodiro) A. R. Sm.	rbcL	DQ151921	FTOL v1.7
Polypodiaceae	Serpocaulon subandinum (Sodiro) A. R. Sm.	rps4	DQ151947	FTOL v1.7
Polypodiaceae	Serpocaulon subandinum (Sodiro) A. R. Sm.	rps4-trnS	DQ151946	FTOL v1.7
Polypodiaceae	Serpocaulon subandinum (Sodiro) A. R. Sm.	trnL-trnF	DQ151973	FTOL v1.7
Polypodiaceae	Serpocaulon triseriale (Sw.) A. R. Sm.	atpA	EF463850	FTOL v1.7
Polypodiaceae	Serpocaulon triseriale (Sw.) A. R. Sm.	atpB	EF463516	FTOL v1.7
Polypodiaceae	Serpocaulon triseriale (Sw.) A. R. Sm.	rbcL	EF463263	FTOL v1.7
Polypodiaceae	Serpocaulon triseriale (Sw.) A. R. Sm.	rps4	DQ151953	FTOL v1.7
Polypodiaceae	Serpocaulon triseriale (Sw.) A. R. Sm.	rps4-trnS	DQ151953	FTOL v1.7
Polypodiaceae	Serpocaulon triseriale (Sw.) A. R. Sm.	trnL-trnF	DQ151980	FTOL v1.7
Polypodiaceae	Serpocaulon vacillans (Link) A. R. Sm.	rbcL	DQ151927	FTOL v1.7
Polypodiaceae	Serpocaulon vacillans (Link) A. R. Sm.	rps4	DQ151954	FTOL v1.7
Polypodiaceae	Serpocaulon vacillans (Link) A. R. Sm.	rps4-trnS	DQ151954	FTOL v1.7
Polypodiaceae	Serpocaulon vacillans (Link) A. R. Sm.	trnL-trnF	DQ151981	FTOL v1.7
Polypodiaceae	Serpocaulon wagneri (Mett.) A. R. Sm.	rbcL	DQ151928	FTOL v1.7
Polypodiaceae	Serpocaulon wagneri (Mett.) A. R. Sm.	rps4	DQ151955	FTOL v1.7
Polypodiaceae	Serpocaulon wagneri (Mett.) A. R. Sm.	rps4-trnS	DQ151955	FTOL v1.7
Polypodiaceae	Serpocaulon wagneri (Mett.) A. R. Sm.	trnL-trnF	DQ151982	FTOL v1.7
Polypodiaceae	Stenogrammitis delitescens (Maxon) Labiak	rbcL	MK319134	FTOL v1.7
Polypodiaceae	Stenogrammitis delitescens (Maxon) Labiak	trnL-trnF	MK319035	FTOL v1.7
Polypodiaceae	Stenogrammitis hartii (Jenman) Labiak	atpB	GU376583	FTOL v1.7
Polypodiaceae	Stenogrammitis hartii (Jenman) Labiak	rbcL	GU386985	FTOL v1.7
Polypodiaceae	Stenogrammitis hartii (Jenman) Labiak	rps4-trnS	GU387057	FTOL v1.7
Polypodiaceae	Stenogrammitis hartii (Jenman) Labiak	trnL-trnF	GU387225	FTOL v1.7
Polypodiaceae	Stenogrammitis hellwigii (Mickel & Beitel) Labiak	atpB	GU376584	FTOL v1.7
Polypodiaceae	Stenogrammitis hellwigii (Mickel & Beitel) Labiak	rbcL	GU386990	FTOL v1.7
Polypodiaceae	Stenogrammitis hellwigii (Mickel & Beitel) Labiak	rps4-trnS	GU387058	FTOL v1.7
Polypodiaceae	Stenogrammitis hellwigii (Mickel & Beitel) Labiak	trnL-trnF	GU387226	FTOL v1.7
Polypodiaceae	Stenogrammitis hildebrandtii (Hieron.) Labiak	atpB	GU376585	FTOL v1.7
Polypodiaceae	Stenogrammitis hildebrandtii (Hieron.) Labiak	rbcL	GU386975	FTOL v1.7
Polypodiaceae	Stenogrammitis hildebrandtii (Hieron.) Labiak	rps4-trnS	GU387059	FTOL v1.7
Polypodiaceae	Stenogrammitis hildebrandtii (Hieron.) Labiak	trnL-trnF	GU387227	FTOL v1.7
Polypodiaceae	Stenogrammitis limula (Christ) Labiak	atpB	GU376591	FTOL v1.7
Polypodiaceae	Stenogrammitis limula (Christ) Labiak	rbcL	GU387023	FTOL v1.7
Polypodiaceae	Stenogrammitis limula (Christ) Labiak	rps4-trnS	GU387067	FTOL v1.7
Polypodiaceae	Stenogrammitis limula (Christ) Labiak	trnL-trnF	GU387232	FTOL v1.7
Polypodiaceae	Stenogrammitis myosuroides (Sw.) Labiak	atpB	GU376595	FTOL v1.7
Polypodiaceae	Stenogrammitis myosuroides (Sw.) Labiak	rbcL	GU386971	FTOL v1.7
Polypodiaceae	Stenogrammitis myosuroides (Sw.) Labiak	rps4-trnS	GU387072	FTOL v1.7
Polypodiaceae	Stenogrammitis myosuroides (Sw.) Labiak	trnL-trnF	GU387236	FTOL v1.7
Polypodiaceae	Stenogrammitis nutata (Jenman) Labiak	rbcL	MK319148	FTOL v1.7
Polypodiaceae	Stenogrammitis nutata (Jenman) Labiak	trnL-trnF	MK319041	FTOL v1.7
Polypodiaceae	Stenogrammitis oosora (Baker) Labiak	atpB	KY711846	FTOL v1.7
Polypodiaceae	Stenogrammitis oosora (Baker) Labiak	rbcL	KY712022	FTOL v1.7
Polypodiaceae	Stenogrammitis oosora (Baker) Labiak	rps4-trnS	KY712338	FTOL v1.7
Polypodiaceae	Stenogrammitis oosora (Baker) Labiak	trnL-trnF	KY711671	FTOL v1.7
Polypodiaceae	Stenogrammitis prionodes (Mickel & Beitel) Labiak	atpB	GU376605	FTOL v1.7
Polypodiaceae	Stenogrammitis prionodes (Mickel & Beitel) Labiak	rbcL	GU386991	FTOL v1.7
Polypodiaceae	Stenogrammitis prionodes (Mickel & Beitel) Labiak	rps4-trnS	GU387086	FTOL v1.7
Polypodiaceae	Stenogrammitis prionodes (Mickel & Beitel) Labiak	trnL-trnF	GU387249	FTOL v1.7
Polypodiaceae	Stenogrammitis pumila (Labiak) Labiak	atpB	GU376608	FTOL v1.7
Polypodiaceae	Stenogrammitis pumila (Labiak) Labiak	rbcL	GU387022	FTOL v1.7
Polypodiaceae	Stenogrammitis pumila (Labiak) Labiak	rps4-trnS	GU387089	FTOL v1.7
Polypodiaceae	Stenogrammitis pumila (Labiak) Labiak	trnL-trnF	GU387252	FTOL v1.7
Polypodiaceae	Stenogrammitis saffordii (Maxon) Labiak	atpB	EF178645	FTOL v1.7
Polypodiaceae	Stenogrammitis saffordii (Maxon) Labiak	rbcL	EF178628	FTOL v1.7
Polypodiaceae	Stenogrammitis saffordii (Maxon) Labiak	trnL-trnF	EF178662	FTOL v1.7
Polypodiaceae	Stenogrammitis subcoriacea (Copel.) Labiak	atpB	HQ599537	FTOL v1.7
Polypodiaceae	Stenogrammitis subcoriacea (Copel.) Labiak	rbcL	HQ599529	FTOL v1.7
Polypodiaceae	Stenogrammitis subcoriacea (Copel.) Labiak	trnL-trnF	HQ599519	FTOL v1.7
Polypodiaceae	Stenogrammitis wittigiana (Fée & Glaz.) Labiak	atpB	GU376624	FTOL v1.7
Polypodiaceae	Stenogrammitis wittigiana (Fée & Glaz.) Labiak	rbcL	GU386989	FTOL v1.7
Polypodiaceae	Stenogrammitis wittigiana (Fée & Glaz.) Labiak	rps4-trnS	GU387073	FTOL v1.7
Polypodiaceae	Stenogrammitis wittigiana (Fée & Glaz.) Labiak	trnL-trnF	GU387269	FTOL v1.7
Polypodiaceae	Synammia espinosae (Weath.) G. Kunkel	plastome	MW876373	FTOL v1.7
Polypodiaceae	Synammia feuillei (Bertero) Copel.	plastome	MW876374	FTOL v1.7
Polypodiaceae	Synammia intermedia (Colla) G. Kunkel	plastome	MW876375	FTOL v1.7
Polypodiaceae	Terpsichore asplenifolia (L.) A. R. Sm.	plastome	MZ357094	FTOL v1.7
Polypodiaceae	Terpsichore atroviridis (Copel.) A. R. Sm.	rbcL	MW138276	FTOL v1.7
Polypodiaceae	Terpsichore chrysleri (Copel.) A. R. Sm.	atpB	KM218859	FTOL v1.7
Polypodiaceae	Terpsichore chrysleri (Copel.) A. R. Sm.	rbcL	KM218813	FTOL v1.7
Polypodiaceae	Terpsichore eggersii (Baker ex Hook.) A. R. Sm.	atpB	AF469785	FTOL v1.7
Polypodiaceae	Terpsichore eggersii (Baker ex Hook.) A. R. Sm.	rbcL	AF468209	FTOL v1.7
Polypodiaceae	Terpsichore eggersii (Baker ex Hook.) A. R. Sm.	trnL-trnF	AF469798	FTOL v1.7
Polypodiaceae	Terpsichore hanekeana (Proctor) A. R. Sm.	atpB	AY459503	FTOL v1.7
Polypodiaceae	Terpsichore hanekeana (Proctor) A. R. Sm.	rbcL	AY460670	FTOL v1.7
Polypodiaceae	Terpsichore lehmanniana (Hieron.) A. R. Sm.	atpB	AY459506	FTOL v1.7
Polypodiaceae	Terpsichore lehmanniana (Hieron.) A. R. Sm.	rbcL	AY460673	FTOL v1.7
Polypodiaceae	Terpsichore staheliana (Posth.) A. R. Sm.	rbcL	MK319119	FTOL v1.7
Polypodiaceae	Thalassogrammitis deplanchei (Baker) Parris, Sundue,	atpB	KY711801	FTOL v1.7
Polypodiaceae	Thalassogrammitis deplanchei (Baker) Parris, Sundue,	rbcL	KY711976	FTOL v1.7
Polypodiaceae	Thalassogrammitis deplanchei (Baker) Parris, Sundue,	rps4-trnS	KY712299	FTOL v1.7
Polypodiaceae	Thalassogrammitis deplanchei (Baker) Parris, Sundue,	trnL-trnF	KY711625	FTOL v1.7
Polypodiaceae	Thylacopteris minuta K.Hori & Khine	rbcL	LC685476	FTOL v1.7
Polypodiaceae	Thylacopteris papillosa (Blume) Kunze ex J. Sm.	plastome	MW876376	FTOL v1.7
Polypodiaceae	Tomophyllum donianum (Spreng.) Fraser-Jenk. & Parri	plastome	MT130584	FTOL v1.7
Polypodiaceae	Tomophyllum macrum (Copel.) Parris	atpB	KM218830	FTOL v1.7
Polypodiaceae	Tomophyllum macrum (Copel.) Parris	rbcL	KM218780	FTOL v1.7
Polypodiaceae	Tomophyllum perplexum (Parris) Parris	atpB	KY711901	FTOL v1.7
Polypodiaceae	Tomophyllum perplexum (Parris) Parris	trnL-trnF	KY711725	FTOL v1.7
Polypodiaceae	Tomophyllum repandulum (Kunze ex Mett.) Parris	atpB	KY711900	FTOL v1.7
Polypodiaceae	Tomophyllum repandulum (Kunze ex Mett.) Parris	rbcL	KY712072	FTOL v1.7
Polypodiaceae	Tomophyllum repandulum (Kunze ex Mett.) Parris	trnL-trnF	KY711724	FTOL v1.7
Polypodiaceae	Tomophyllum sakaguchianum (Koidz.) Parris	rbcL	AB575254	FTOL v1.7
Polypodiaceae	Tomophyllum secundum (Ridl.) Parris	atpB	KM218832	FTOL v1.7
Polypodiaceae	Tomophyllum secundum (Ridl.) Parris	rbcL	KM218781	FTOL v1.7
Polypodiaceae	Tomophyllum secundum (Ridl.) Parris	rps4-trnS	KM106195	FTOL v1.7
Polypodiaceae	Tomophyllum secundum (Ridl.) Parris	trnL-trnF	KM106095	FTOL v1.7
Polypodiaceae	Tomophyllum subsecundodissectum (Zoll.) Parris	atpB	KY711895	FTOL v1.7
Polypodiaceae	Tomophyllum subsecundodissectum (Zoll.) Parris	trnL-trnF	KY711718	FTOL v1.7
Polypodiaceae	Tomophyllum walleri (Maiden & Betche) Parris	atpB	KM218831	FTOL v1.7
Polypodiaceae	Tomophyllum walleri (Maiden & Betche) Parris	rbcL	KM218782	FTOL v1.7
Polypodiaceae	Tomophyllum walleri (Maiden & Betche) Parris	rps4-trnS	KM106196	FTOL v1.7
Polypodiaceae	Tomophyllum walleri (Maiden & Betche) Parris	trnL-trnF	KM106096	FTOL v1.7
Polypodiaceae	Xiphopterella devolii S. J. Moore, Parris & W. L. Chiou	rps4	OQ717250	FTOL v1.7
Polypodiaceae	Xiphopterella devolii S. J. Moore, Parris & W. L. Chiou	rps4-trnS	OQ717250	FTOL v1.7
Polypodiaceae	Xiphopterella hieronymusii (C. Chr.) Parris	rbcL	KM218783	FTOL v1.7
Polypodiaceae	Xiphopterella hieronymusii (C. Chr.) Parris	rps4-trnS	KM106197	FTOL v1.7
Polypodiaceae	Xiphopterella hieronymusii (C. Chr.) Parris	trnL-trnF	KM106097	FTOL v1.7
Polypodiaceae	Xiphopterella sparsipilosa (Holttum) Parris	rbcL	KM218784	FTOL v1.7
Polypodiaceae	Xiphopterella sparsipilosa (Holttum) Parris	rps4-trnS	KM106198	FTOL v1.7
Polypodiaceae	Xiphopterella sparsipilosa (Holttum) Parris	trnL-trnF	KM106098	FTOL v1.7
Polypodiaceae	Zealandia novae-zealandiae (Baker) Testo & A. R. Fiel	rbcL	DQ401116	FTOL v1.7
Polypodiaceae	Zealandia novae-zealandiae (Baker) Testo & A. R. Fiel	rps4	DQ401126	FTOL v1.7
Polypodiaceae	Zealandia novae-zealandiae (Baker) Testo & A. R. Fiel	rps4-trnS	DQ401126	FTOL v1.7
Polypodiaceae	Zealandia novae-zealandiae (Baker) Testo & A. R. Fiel	trnL-trnF	DQ401124	FTOL v1.7
Polypodiaceae	Zealandia powellii (Baker) Testo & A. R. Field	rbcL	KY099831	FTOL v1.7
Polypodiaceae	Zealandia powellii (Baker) Testo & A. R. Field	trnL-trnF	MG427072	FTOL v1.7
Polypodiaceae	Zealandia pustulata (G. Forst.) Testo & A. R. Field	atpA	MH113546	FTOL v1.7
Polypodiaceae	Zealandia pustulata (G. Forst.) Testo & A. R. Field	atpB	MH113580	FTOL v1.7
Polypodiaceae	Zealandia pustulata (G. Forst.) Testo & A. R. Field	rbcL	MH051181	FTOL v1.7
Polypodiaceae	Zealandia pustulata (G. Forst.) Testo & A. R. Field	rps4	MH113480	FTOL v1.7
Polypodiaceae	Zealandia pustulata (G. Forst.) Testo & A. R. Field	rps4-trnS	MH113480	FTOL v1.7
Polypodiaceae	Zealandia pustulata (G. Forst.) Testo & A. R. Field	trnL-trnF	MH113513	FTOL v1.7
Polypodiaceae	Zealandia vieillardii (Mett.) Testo & A. R. Field	rbcL	DQ179635	FTOL v1.7
Polypodiaceae	Zealandia vieillardii (Mett.) Testo & A. R. Field	rps4	DQ179637	FTOL v1.7
Polypodiaceae	Zealandia vieillardii (Mett.) Testo & A. R. Field	rps4-trnS	DQ179638	FTOL v1.7
Polypodiaceae	Zealandia vieillardii (Mett.) Testo & A. R. Field	trnL-trnF	DQ179645	FTOL v1.7
Pteridryaceae	Draconopteris draconoptera (D. C. Eaton) Li Bing Zhang	atpA	KU605209	FTOL v1.7
Pteridryaceae	Draconopteris draconoptera (D. C. Eaton) Li Bing Zhang	atpB	MN781245	FTOL v1.7
Pteridryaceae	Draconopteris draconoptera (D. C. Eaton) Li Bing Zhang	rbcL	KU605195	FTOL v1.7
Pteridryaceae	Draconopteris draconoptera (D. C. Eaton) Li Bing Zhang	rps4	KU605088	FTOL v1.7
Pteridryaceae	Draconopteris draconoptera (D. C. Eaton) Li Bing Zhang	rps4-trnS	KU605088	FTOL v1.7
Pteridryaceae	Draconopteris draconoptera (D. C. Eaton) Li Bing Zhang	trnL-trnF	KU605111	FTOL v1.7
Pteridryaceae	Malaifilix grandidentata (Ces.) Li Bing Zhang & Schuett	atpA	KU605210	FTOL v1.7
Pteridryaceae	Malaifilix grandidentata (Ces.) Li Bing Zhang & Schuett	rbcL	KU605197	FTOL v1.7
Pteridryaceae	Malaifilix grandidentata (Ces.) Li Bing Zhang & Schuett	rps4	KU605090	FTOL v1.7
Pteridryaceae	Malaifilix grandidentata (Ces.) Li Bing Zhang & Schuett	rps4-trnS	KU605090	FTOL v1.7
Pteridryaceae	Malaifilix grandidentata (Ces.) Li Bing Zhang & Schuett	trnL-trnF	KU605113	FTOL v1.7
Pteridryaceae	Polydictyum menyanthidis C. Presl	atpB	MF623680	FTOL v1.7
Pteridryaceae	Polydictyum menyanthidis C. Presl	rbcL	MF623752	FTOL v1.7
Pteridryaceae	Polydictyum menyanthidis C. Presl	trnL-trnF	MF623776	FTOL v1.7
Pteridryaceae	Polydictyum ternatum (Baker) S. Y. Dong & C. W. Chen	atpB	MF623681	FTOL v1.7
Pteridryaceae	Polydictyum ternatum (Baker) S. Y. Dong & C. W. Chen	rbcL	MF623753	FTOL v1.7
Pteridryaceae	Polydictyum ternatum (Baker) S. Y. Dong & C. W. Chen	trnL-trnF	MF623777	FTOL v1.7
Pteridryaceae	Polydictyum variabile (Tardieu & Ching) S. Y. Dong & C.	atpA	MG517575	FTOL v1.7
Pteridryaceae	Polydictyum variabile (Tardieu & Ching) S. Y. Dong & C.	atpB	MF623682	FTOL v1.7
Pteridryaceae	Polydictyum variabile (Tardieu & Ching) S. Y. Dong & C.	rbcL	MG517578	FTOL v1.7
Pteridryaceae	Polydictyum variabile (Tardieu & Ching) S. Y. Dong & C.	rps4	OR087336	FTOL v1.7
Pteridryaceae	Polydictyum variabile (Tardieu & Ching) S. Y. Dong & C.	rps4-trnS	OR087335	FTOL v1.7
Pteridryaceae	Polydictyum variabile (Tardieu & Ching) S. Y. Dong & C.	trnL-trnF	OR087522	FTOL v1.7
Pteridryaceae	Pteridrys australis Ching ex C. Chr. & Ching	atpB	KJ196486	FTOL v1.7
Pteridryaceae	Pteridrys australis Ching ex C. Chr. & Ching	rbcL	KJ196892	FTOL v1.7
Pteridryaceae	Pteridrys australis Ching ex C. Chr. & Ching	trnL-trnF	KJ196678	FTOL v1.7
Pteridryaceae	Pteridrys cnemidaria (Christ) C. Chr.	plastome	MT130579	FTOL v1.7
Pteridryaceae	Pteridrys costularis Li Bing Zhang, Liang Zhang, N. T. Lu	rbcL	MG517594	FTOL v1.7
Pteridryaceae	Pteridrys costularis Li Bing Zhang, Liang Zhang, N. T. Lu	trnL-trnF	MG517603	FTOL v1.7
Pteridryaceae	Pteridrys hanoiensis Li Bing Zhang, Liang Zhang, N. T. L	atpA	OR087151	FTOL v1.7
Pteridryaceae	Pteridrys hanoiensis Li Bing Zhang, Liang Zhang, N. T. L	trnL-trnF	MG546824	FTOL v1.7
Pteridryaceae	Pteridrys lofouensis (Christ) C. Chr. & Ching	atpB	EF450527	FTOL v1.7
Pteridryaceae	Pteridrys lofouensis (Christ) C. Chr. & Ching	rbcL	EF460687	FTOL v1.7
Pteridryaceae	Pteridrys microthecia (Fée) C. Chr. & Ching	atpB	KJ196448	FTOL v1.7
Pteridryaceae	Pteridrys microthecia (Fée) C. Chr. & Ching	rbcL	KJ196848	FTOL v1.7
Pteridryaceae	Pteridrys microthecia (Fée) C. Chr. & Ching	trnL-trnF	KJ196630	FTOL v1.7
Pteridryaceae	Pteridrys syrmatica (Willd.) C. Chr. & Ching	atpB	KJ196475	FTOL v1.7
Pteridryaceae	Pteridrys syrmatica (Willd.) C. Chr. & Ching	rbcL	KJ196875	FTOL v1.7
Pteridryaceae	Pteridrys syrmatica (Willd.) C. Chr. & Ching	trnL-trnF	KJ196654	FTOL v1.7
Pteridryaceae	Pteridrys triangularis Li Bing Zhang & X. M. Zhou	trnL-trnF	MG517605	FTOL v1.7
Pteridryaceae	Pteridrys vietnamensis Li Bing Zhang, Liang Zhang, N. T	atpA	OR087152	FTOL v1.7
Pteridryaceae	Pteridrys vietnamensis Li Bing Zhang, Liang Zhang, N. T	rps4	OR087337	FTOL v1.7
Pteridryaceae	Pteridrys vietnamensis Li Bing Zhang, Liang Zhang, N. T	rps4-trnS	OR087337	FTOL v1.7
Tectariaceae	Hypoderris brauniana (H. Karst.) F. G. Wang & Christen	atpA	KF898007	FTOL v1.7
Tectariaceae	Hypoderris brauniana (H. Karst.) F. G. Wang & Christen	rbcL	KF667648	FTOL v1.7
Tectariaceae	Hypoderris brauniana (H. Karst.) F. G. Wang & Christen	trnL-trnF	OR087521	FTOL v1.7
Tectariaceae	Hypoderris brownii J. Sm.	atpA	KF898001	FTOL v1.7

Tectariaceae	Hypoderris brownii J. Sm.	rbcL	KF887164	FTOL v1.7
Tectariaceae	Hypoderris brownii J. Sm.	trnL-trnF	KF897949	FTOL v1.7
Tectariaceae	Hypoderris nicotianifolia (Baker) Moran et al.	rbcL	KF667654	FTOL v1.7
Tectariaceae	Hypoderris nicotianifolia (Baker) Moran et al.	rps4-trnS	KF667569	FTOL v1.7
Tectariaceae	Hypoderris nicotianifolia (Baker) Moran et al.	trnL-trnF	KF667627	FTOL v1.7
Tectariaceae	Tectaria acerifolia R. C. Moran	atpA	KF898006	FTOL v1.7
Tectariaceae	Tectaria acerifolia R. C. Moran	rbcL	KJ628842	FTOL v1.7
Tectariaceae	Tectaria acerifolia R. C. Moran	trnL-trnF	KF897954	FTOL v1.7
Tectariaceae	Tectaria aenigma C. W. Chen & C. J. Rothf.	rbcL	KY927533	FTOL v1.7
Tectariaceae	Tectaria aenigma C. W. Chen & C. J. Rothf.	trnL-trnF	KY927538	FTOL v1.7
Tectariaceae	Tectaria angelicifolia (Schum.) Copel.	rps4-trnS	KF667560	FTOL v1.7
Tectariaceae	Tectaria angelicifolia (Schum.) Copel.	trnL-trnF	KF667616	FTOL v1.7
Tectariaceae	Tectaria angulata (Willd.) Copel.	atpA	OR087155	FTOL v1.7
Tectariaceae	Tectaria angulata (Willd.) Copel.	rbcL	OR087023	FTOL v1.7
Tectariaceae	Tectaria angulata (Willd.) Copel.	rps4	OR087342	FTOL v1.7
Tectariaceae	Tectaria angulata (Willd.) Copel.	rps4-trnS	OR087342	FTOL v1.7
Tectariaceae	Tectaria angulata (Willd.) Copel.	trnL-trnF	OR087527	FTOL v1.7
Tectariaceae	Tectaria antioquoiana (Baker) C.Chr.	atpA	OR087156	FTOL v1.7
Tectariaceae	Tectaria antioquoiana (Baker) C.Chr.	rbcL	MW138146	FTOL v1.7
Tectariaceae	Tectaria antioquoiana (Baker) C.Chr.	rps4	OR087343	FTOL v1.7
Tectariaceae	Tectaria antioquoiana (Baker) C.Chr.	rps4-trnS	OR087343	FTOL v1.7
Tectariaceae	Tectaria antioquoiana (Baker) C.Chr.	trnL-trnF	OR087529	FTOL v1.7
Tectariaceae	Tectaria athyrioides (Baker) C. Chr.	rbcL	KJ628844	FTOL v1.7
Tectariaceae	Tectaria aurita (Sw.) S. Chandra	atpB	KJ196404	FTOL v1.7
Tectariaceae	Tectaria aurita (Sw.) S. Chandra	rbcL	KJ196849	FTOL v1.7
Tectariaceae	Tectaria aurita (Sw.) S. Chandra	trnL-trnF	KJ196631	FTOL v1.7
Tectariaceae	Tectaria balansae (C. Chr.) C. Chr.	atpA	KY937628	FTOL v1.7
Tectariaceae	Tectaria balansae (C. Chr.) C. Chr.	rbcL	KY937371	FTOL v1.7
Tectariaceae	Tectaria balansae (C. Chr.) C. Chr.	rps4	KY937457	FTOL v1.7
Tectariaceae	Tectaria balansae (C. Chr.) C. Chr.	rps4-trnS	KY937458	FTOL v1.7
Tectariaceae	Tectaria balansae (C. Chr.) C. Chr.	trnL-trnF	KY937562	FTOL v1.7
Tectariaceae	Tectaria barberi (Hook.) Copel.	atpB	KJ196445	FTOL v1.7
Tectariaceae	Tectaria barberi (Hook.) Copel.	rbcL	KJ196846	FTOL v1.7
Tectariaceae	Tectaria barberi (Hook.) Copel.	trnL-trnF	KJ196628	FTOL v1.7
Tectariaceae	Tectaria barteri (J. Sm.) C. Chr.	rps4-trnS	KF667561	FTOL v1.7
Tectariaceae	Tectaria barteri (J. Sm.) C. Chr.	trnL-trnF	KF667617	FTOL v1.7
Tectariaceae	Tectaria beccariana (Ces.) C. Chr.	atpB	OK480073	FTOL v1.7
Tectariaceae	Tectaria beccariana (Ces.) C. Chr.	rbcL	OK104174	FTOL v1.7
Tectariaceae	Tectaria beccariana (Ces.) C. Chr.	trnL-trnF	OK480194	FTOL v1.7
Tectariaceae	Tectaria borneensis S. Y. Dong	atpB	KJ196489	FTOL v1.7
Tectariaceae	Tectaria borneensis S. Y. Dong	rbcL	KJ196854	FTOL v1.7
Tectariaceae	Tectaria borneensis S. Y. Dong	trnL-trnF	KJ196642	FTOL v1.7
Tectariaceae	Tectaria christovalensis (C. Chr.) Alston	atpB	OK480080	FTOL v1.7
Tectariaceae	Tectaria christovalensis (C. Chr.) Alston	rbcL	MF623760	FTOL v1.7
Tectariaceae	Tectaria christovalensis (C. Chr.) Alston	trnL-trnF	OK480201	FTOL v1.7
Tectariaceae	Tectaria cicutaria (L.) Copel.	atpB	KJ196408	FTOL v1.7
Tectariaceae	Tectaria cicutaria (L.) Copel.	rbcL	KF667649	FTOL v1.7
Tectariaceae	Tectaria cicutaria (L.) Copel.	rps4-trnS	KF667562	FTOL v1.7
Tectariaceae	Tectaria cicutaria (L.) Copel.	trnL-trnF	KJ196696	FTOL v1.7
Tectariaceae	Tectaria coadunata (Wall. ex Hook. & Grev.) C. Chr.	plastome	MT130661	FTOL v1.7
Tectariaceae	Tectaria crenata Cav.	atpA	KY937584	FTOL v1.7
Tectariaceae	Tectaria crenata Cav.	rbcL	KY937326	FTOL v1.7
Tectariaceae	Tectaria crenata Cav.	rps4	OR087357	FTOL v1.7
Tectariaceae	Tectaria crenata Cav.	rps4-trnS	OR087357	FTOL v1.7
Tectariaceae	Tectaria crenata Cav.	trnL-trnF	KY937485	FTOL v1.7
Tectariaceae	Tectaria danangensis S.Y.Dong	atpB	OM671283	FTOL v1.7
Tectariaceae	Tectaria danangensis S.Y.Dong	rbcL	OM671282	FTOL v1.7
Tectariaceae	Tectaria danangensis S.Y.Dong	rps4	OR087359	FTOL v1.7
Tectariaceae	Tectaria danangensis S.Y.Dong	rps4-trnS	OR087359	FTOL v1.7
Tectariaceae	Tectaria danangensis S.Y.Dong	trnL-trnF	OM671286	FTOL v1.7
Tectariaceae	Tectaria darienensis A. Rojas	rbcL	KF667651	FTOL v1.7
Tectariaceae	Tectaria darienensis A. Rojas	rps4-trnS	KF667564	FTOL v1.7
Tectariaceae	Tectaria darienensis A. Rojas	trnL-trnF	KF667622	FTOL v1.7
Tectariaceae	Tectaria decurrens (C. Presl) Copel.	plastome	MN623363	FTOL v1.7
Tectariaceae	Tectaria dissecta (G. Forst.) Lellinger	atpB	MH542580	FTOL v1.7
Tectariaceae	Tectaria dissecta (G. Forst.) Lellinger	rbcL	MH542570	FTOL v1.7
Tectariaceae	Tectaria dissecta (G. Forst.) Lellinger	trnL-trnF	MH542614	FTOL v1.7
Tectariaceae	Tectaria dubia (C. B. Clarke & Baker) Ching	atpB	MF623690	FTOL v1.7
Tectariaceae	Tectaria dubia (C. B. Clarke & Baker) Ching	rbcL	MF623762	FTOL v1.7
Tectariaceae	Tectaria dubia (C. B. Clarke & Baker) Ching	trnL-trnF	MF623783	FTOL v1.7
Tectariaceae	Tectaria durvillei (Bory) Holttum	atpB	OK480091	FTOL v1.7
Tectariaceae	Tectaria durvillei (Bory) Holttum	rbcL	OK104187	FTOL v1.7
Tectariaceae	Tectaria durvillei (Bory) Holttum	trnL-trnF	OK480212	FTOL v1.7
Tectariaceae	Tectaria ebenina (C. Chr.) Ching	atpA	KY937589	FTOL v1.7

Tectariaceae	Tectaria ebenina (C. Chr.) Ching	rbcL	KY937333	FTOL v1.7
Tectariaceae	Tectaria ebenina (C. Chr.) Ching	rps4	KY937393	FTOL v1.7
Tectariaceae	Tectaria ebenina (C. Chr.) Ching	rps4-trnS	KY937393	FTOL v1.7
Tectariaceae	Tectaria ebenina (C. Chr.) Ching	trnL-trnF	KY937492	FTOL v1.7
Tectariaceae	Tectaria fauriei Tagawa	atpA	OR087182	FTOL v1.7
Tectariaceae	Tectaria fauriei Tagawa	rbcL	OR087037	FTOL v1.7
Tectariaceae	Tectaria fauriei Tagawa	rps4	OR087371	FTOL v1.7
Tectariaceae	Tectaria fauriei Tagawa	rps4-trnS	OR087371	FTOL v1.7
Tectariaceae	Tectaria fauriei Tagawa	trnL-trnF	OR087549	FTOL v1.7
Tectariaceae	Tectaria fernandensis (Baker) C. Chr.	atpA	KF898010	FTOL v1.7
Tectariaceae	Tectaria fernandensis (Baker) C. Chr.	rbcL	KF887174	FTOL v1.7
Tectariaceae	Tectaria fernandensis (Baker) C. Chr.	trnL-trnF	KF897958	FTOL v1.7
Tectariaceae	Tectaria ferruginea (Mett.) Copel.	atpB	OK480092	FTOL v1.7
Tectariaceae	Tectaria ferruginea (Mett.) Copel.	rbcL	OK104207	FTOL v1.7
Tectariaceae	Tectaria ferruginea (Mett.) Copel.	trnL-trnF	OK480213	FTOL v1.7
Tectariaceae	Tectaria fimbriata (Willd.) Proctor & Lourteig	atpA	EF463867	FTOL v1.7
Tectariaceae	Tectaria fimbriata (Willd.) Proctor & Lourteig	atpB	EF463527	FTOL v1.7
Tectariaceae	Tectaria fimbriata (Willd.) Proctor & Lourteig	rbcL	EF463271	FTOL v1.7
Tectariaceae	Tectaria fissa (Kunze) Holttum	atpB	MF623694	FTOL v1.7
Tectariaceae	Tectaria fissa (Kunze) Holttum	rbcL	MF623766	FTOL v1.7
Tectariaceae	Tectaria fissa (Kunze) Holttum	trnL-trnF	MF623787	FTOL v1.7
Tectariaceae	Tectaria fungii S.Y.Dong	atpB	MH542581	FTOL v1.7
Tectariaceae	Tectaria fungii S.Y.Dong	rbcL	MH542571	FTOL v1.7
Tectariaceae	Tectaria fungii S.Y.Dong	trnL-trnF	MH542615	FTOL v1.7
Tectariaceae	Tectaria fuscipes (Wall.) C. Chr.	plastome	MT130612	FTOL v1.7
Tectariaceae	Tectaria gaudichaudii (Mett.) Maxon	atpA	KF898012	FTOL v1.7
Tectariaceae	Tectaria gaudichaudii (Mett.) Maxon	rbcL	KF887176	FTOL v1.7
Tectariaceae	Tectaria gigantea (Blume) Copel.	atpB	KJ196403	FTOL v1.7
Tectariaceae	Tectaria gigantea (Blume) Copel.	rbcL	KJ196853	FTOL v1.7
Tectariaceae	Tectaria gigantea (Blume) Copel.	trnL-trnF	KJ196660	FTOL v1.7
Tectariaceae	Tectaria griffithii (Baker) C. Chr.	atpA	KY937596	FTOL v1.7
Tectariaceae	Tectaria griffithii (Baker) C. Chr.	rbcL	KY937337	FTOL v1.7
Tectariaceae	Tectaria griffithii (Baker) C. Chr.	rps4	KY937401	FTOL v1.7
Tectariaceae	Tectaria griffithii (Baker) C. Chr.	rps4-trnS	KY937401	FTOL v1.7
Tectariaceae	Tectaria griffithii (Baker) C. Chr.	trnL-trnF	KY937501	FTOL v1.7
Tectariaceae	Tectaria grossedentata Ching & Chu H. Wang	atpA	KY937601	FTOL v1.7
Tectariaceae	Tectaria grossedentata Ching & Chu H. Wang	rbcL	KY937338	FTOL v1.7
Tectariaceae	Tectaria grossedentata Ching & Chu H. Wang	rps4	KY937407	FTOL v1.7
Tectariaceae	Tectaria grossedentata Ching & Chu H. Wang	rps4-trnS	KY937407	FTOL v1.7
Tectariaceae	Tectaria grossedentata Ching & Chu H. Wang	trnL-trnF	KY937507	FTOL v1.7
Tectariaceae	Tectaria harlandii (Hook.) C. M. Kuo	atpA	KY937603	FTOL v1.7
Tectariaceae	Tectaria harlandii (Hook.) C. M. Kuo	rbcL	KY937339	FTOL v1.7
Tectariaceae	Tectaria harlandii (Hook.) C. M. Kuo	trnL-trnF	KY937511	FTOL v1.7
Tectariaceae	Tectaria heracleifolia (Willd.) Underw.	atpA	KF898016	FTOL v1.7
Tectariaceae	Tectaria heracleifolia (Willd.) Underw.	atpB	KJ196407	FTOL v1.7
Tectariaceae	Tectaria heracleifolia (Willd.) Underw.	rbcL	KY937341	FTOL v1.7
Tectariaceae	Tectaria heracleifolia (Willd.) Underw.	trnL-trnF	KY937515	FTOL v1.7
Tectariaceae	Tectaria herpetocaulos Holttum	atpA	OR087198	FTOL v1.7
Tectariaceae	Tectaria herpetocaulos Holttum	rbcL	OR087043	FTOL v1.7
Tectariaceae	Tectaria herpetocaulos Holttum	rps4	OR087384	FTOL v1.7
Tectariaceae	Tectaria herpetocaulos Holttum	rps4-trnS	OR087384	FTOL v1.7
Tectariaceae	Tectaria herpetocaulos Holttum	trnL-trnF	OR087560	FTOL v1.7
Tectariaceae	Tectaria heterocarpa (Bedd.) C. V. Morton	atpA	KF898017	FTOL v1.7
Tectariaceae	Tectaria heterocarpa (Bedd.) C. V. Morton	rbcL	KF887181	FTOL v1.7
Tectariaceae	Tectaria heterocarpa (Bedd.) C. V. Morton	trnL-trnF	KF897964	FTOL v1.7
Tectariaceae	Tectaria hilocarpa (Fée) M. G. Price	atpB	OK480094	FTOL v1.7
Tectariaceae	Tectaria hilocarpa (Fée) M. G. Price	rbcL	OK104188	FTOL v1.7
Tectariaceae	Tectaria hilocarpa (Fée) M. G. Price	trnL-trnF	OK480215	FTOL v1.7
Tectariaceae	Tectaria impressa (Fée) Holttum	plastome	MT130620	FTOL v1.7
Tectariaceae	Tectaria incisa Cav.	atpA	OR087154	FTOL v1.7
Tectariaceae	Tectaria incisa Cav.	rbcL	OR087022	FTOL v1.7
Tectariaceae	Tectaria incisa Cav.	rps4	OR087341	FTOL v1.7
Tectariaceae	Tectaria incisa Cav.	rps4-trnS	OR087341	FTOL v1.7
Tectariaceae	Tectaria incisa Cav.	trnL-trnF	OR087526	FTOL v1.7
Tectariaceae	Tectaria ingens (Atk. ex C. B. Clarke) Holttum	atpA	OR087206	FTOL v1.7
Tectariaceae	Tectaria ingens (Atk. ex C. B. Clarke) Holttum	rbcL	KY937344	FTOL v1.7
Tectariaceae	Tectaria ingens (Atk. ex C. B. Clarke) Holttum	rps4	KY937418	FTOL v1.7
Tectariaceae	Tectaria ingens (Atk. ex C. B. Clarke) Holttum	rps4-trnS	KY937418	FTOL v1.7
Tectariaceae	Tectaria ingens (Atk. ex C. B. Clarke) Holttum	trnL-trnF	KY937522	FTOL v1.7
Tectariaceae	Tectaria jardini (Mett.) E.D.Br.	rbcL	MT216133	FTOL v1.7
Tectariaceae	Tectaria jardinii (Mett.) E. D. Br.	rbcL	MT657543	FTOL v1.7
Tectariaceae	Tectaria keckii (Luerss.) C. Chr.	atpA	OR087207	FTOL v1.7
Tectariaceae	Tectaria keckii (Luerss.) C. Chr.	trnL-trnF	OR087569	FTOL v1.7
Tectariaceae	Tectaria kehdingiana (Kuhn ex Luerss.) M. G. Price	atpA	OR087208	FTOL v1.7
Tectariaceae	Tectaria kehdingiana (Kuhn ex Luerss.) M. G. Price	rbcL	OR087047	FTOL v1.7
Tectariaceae	Tectaria kehdingiana (Kuhn ex Luerss.) M. G. Price	rps4	OR087393	FTOL v1.7
Tectariaceae	Tectaria kehdingiana (Kuhn ex Luerss.) M. G. Price	rps4-trnS	OR087393	FTOL v1.7
Tectariaceae	Tectaria kehdingiana (Kuhn ex Luerss.) M. G. Price	trnL-trnF	OR087570	FTOL v1.7
Tectariaceae	Tectaria kusukusensis (Hayata) Lellinger	atpA	OR087209	FTOL v1.7
Tectariaceae	Tectaria kusukusensis (Hayata) Lellinger	rbcL	KY937346	FTOL v1.7
Tectariaceae	Tectaria kusukusensis (Hayata) Lellinger	rps4	OR087395	FTOL v1.7
Tectariaceae	Tectaria kusukusensis (Hayata) Lellinger	rps4-trnS	OR087395	FTOL v1.7
Tectariaceae	Tectaria kusukusensis (Hayata) Lellinger	trnL-trnF	OR087571	FTOL v1.7
Tectariaceae	Tectaria labrusca (Hook.) Copel.	atpB	KJ196499	FTOL v1.7
Tectariaceae	Tectaria labrusca (Hook.) Copel.	rbcL	KJ196818	FTOL v1.7
Tectariaceae	Tectaria labrusca (Hook.) Copel.	trnL-trnF	KJ196692	FTOL v1.7
Tectariaceae	Tectaria laotica Tardieu & C. Chr.	atpA	OR087213	FTOL v1.7
Tectariaceae	Tectaria laotica Tardieu & C. Chr.	rbcL	OR087049	FTOL v1.7
Tectariaceae	Tectaria laotica Tardieu & C. Chr.	rps4	KY937419	FTOL v1.7
Tectariaceae	Tectaria laotica Tardieu & C. Chr.	rps4-trnS	OR087396	FTOL v1.7
Tectariaceae	Tectaria laotica Tardieu & C. Chr.	trnL-trnF	OR087572	FTOL v1.7
Tectariaceae	Tectaria latifolia (G. Forst.) Copel.	atpB	OK480096	FTOL v1.7
Tectariaceae	Tectaria latifolia (G. Forst.) Copel.	rbcL	MT657535	FTOL v1.7
Tectariaceae	Tectaria latifolia (G. Forst.) Copel.	trnL-trnF	OK480217	FTOL v1.7
Tectariaceae	Tectaria lobbii (Hook.) Copel.	atpB	OK480097	FTOL v1.7
Tectariaceae	Tectaria lobbii (Hook.) Copel.	rbcL	OK104209	FTOL v1.7
Tectariaceae	Tectaria lobbii (Hook.) Copel.	trnL-trnF	OK480218	FTOL v1.7
Tectariaceae	Tectaria luchunensis S. K. Wu	atpB	KJ196444	FTOL v1.7
Tectariaceae	Tectaria luchunensis S. K. Wu	rbcL	KJ196891	FTOL v1.7
Tectariaceae	Tectaria luchunensis S. K. Wu	trnL-trnF	KJ196673	FTOL v1.7
Tectariaceae	Tectaria macleanii (Copel.) S. Y. Dong	atpB	KJ196511	FTOL v1.7
Tectariaceae	Tectaria macleanii (Copel.) S. Y. Dong	rbcL	KJ196810	FTOL v1.7
Tectariaceae	Tectaria macleanii (Copel.) S. Y. Dong	trnL-trnF	KJ196680	FTOL v1.7
Tectariaceae	Tectaria manilensis (C. Presl) Holttum	atpB	MF623678	FTOL v1.7
Tectariaceae	Tectaria manilensis (C. Presl) Holttum	rbcL	MF623750	FTOL v1.7
Tectariaceae	Tectaria media Ching	atpA	OR087218	FTOL v1.7
Tectariaceae	Tectaria media Ching	rbcL	OR087053	FTOL v1.7
Tectariaceae	Tectaria media Ching	rps4	OR087402	FTOL v1.7
Tectariaceae	Tectaria media Ching	rps4-trnS	OR087402	FTOL v1.7
Tectariaceae	Tectaria media Ching	trnL-trnF	OR087577	FTOL v1.7
Tectariaceae	Tectaria melanocaulos (Blume) Copel.	atpB	KJ196422	FTOL v1.7
Tectariaceae	Tectaria melanocaulos (Blume) Copel.	rbcL	KJ196832	FTOL v1.7
Tectariaceae	Tectaria melanocaulos (Blume) Copel.	trnL-trnF	KJ196709	FTOL v1.7
Tectariaceae	Tectaria membranacea (Hook.) Fraser-Jenk. & Kholia	atpA	KY937586	FTOL v1.7
Tectariaceae	Tectaria membranacea (Hook.) Fraser-Jenk. & Kholia	rbcL	KY937327	FTOL v1.7
Tectariaceae	Tectaria membranacea (Hook.) Fraser-Jenk. & Kholia	rps4	KY937388	FTOL v1.7
Tectariaceae	Tectaria membranacea (Hook.) Fraser-Jenk. & Kholia	rps4-trnS	KY937388	FTOL v1.7
Tectariaceae	Tectaria membranacea (Hook.) Fraser-Jenk. & Kholia	trnL-trnF	KY937486	FTOL v1.7
Tectariaceae	Tectaria mexicana (Fée) C. V. Morton	atpA	KU605212	FTOL v1.7
Tectariaceae	Tectaria mexicana (Fée) C. V. Morton	rbcL	KU605199	FTOL v1.7
Tectariaceae	Tectaria mexicana (Fée) C. V. Morton	trnL-trnF	KU605115	FTOL v1.7
Tectariaceae	Tectaria microsora A. R. Sm.	atpA	OR087280	FTOL v1.7
Tectariaceae	Tectaria microsora A. R. Sm.	rbcL	OR087086	FTOL v1.7
Tectariaceae	Tectaria microsora A. R. Sm.	rps4	OR087457	FTOL v1.7
Tectariaceae	Tectaria microsora A. R. Sm.	rps4-trnS	OR087457	FTOL v1.7
Tectariaceae	Tectaria microsora A. R. Sm.	trnL-trnF	OR087632	FTOL v1.7

Tectariaceae	Tectaria moorei (Hook.) C. Chr.	rbcL	KF887162	FTOL v1.7
Tectariaceae	Tectaria moorei (Hook.) C. Chr.	trnL-trnF	KF955995	FTOL v1.7
Tectariaceae	Tectaria moranii Li Bing Zhang & G. D. Tang	atpA	OR087297	FTOL v1.7
Tectariaceae	Tectaria multicaudata (C. B. Clarke) Ching	atpA	OR087215	FTOL v1.7
Tectariaceae	Tectaria multicaudata (C. B. Clarke) Ching	rbcL	OR087051	FTOL v1.7
Tectariaceae	Tectaria multicaudata (C. B. Clarke) Ching	rps4	OR087399	FTOL v1.7
Tectariaceae	Tectaria multicaudata (C. B. Clarke) Ching	rps4-trnS	OR087399	FTOL v1.7
Tectariaceae	Tectaria multicaudata (C. B. Clarke) Ching	trnL-trnF	OR087574	FTOL v1.7
Tectariaceae	Tectaria nayarii Mazumdar	atpA	OR087224	FTOL v1.7
Tectariaceae	Tectaria nayarii Mazumdar	rbcL	OR087057	FTOL v1.7
Tectariaceae	Tectaria nayarii Mazumdar	rps4	OR087405	FTOL v1.7
Tectariaceae	Tectaria nayarii Mazumdar	rps4-trnS	OR087405	FTOL v1.7
Tectariaceae	Tectaria nayarii Mazumdar	trnL-trnF	OR087583	FTOL v1.7
Tectariaceae	Tectaria pallescens S.Y.Dong & C.W.Chen	atpB	OK480100	FTOL v1.7
Tectariaceae	Tectaria pallescens S.Y.Dong & C.W.Chen	rbcL	OK104211	FTOL v1.7
Tectariaceae	Tectaria pallescens S.Y.Dong & C.W.Chen	trnL-trnF	OK480222	FTOL v1.7
Tectariaceae	Tectaria palmata (Mett.) Copel.	atpB	OK480102	FTOL v1.7
Tectariaceae	Tectaria palmata (Mett.) Copel.	rbcL	OK104193	FTOL v1.7
Tectariaceae	Tectaria palmata (Mett.) Copel.	trnL-trnF	OK480225	FTOL v1.7
Tectariaceae	Tectaria panamensis (Hook.) R. M. Tryon & A. F. Tryon	plastome	MK705757	FTOL v1.7
Tectariaceae	Tectaria paradoxa (Fée) Sledge	atpA	KF898025	FTOL v1.7
Tectariaceae	Tectaria paradoxa (Fée) Sledge	rbcL	KF887189	FTOL v1.7
Tectariaceae	Tectaria paradoxa (Fée) Sledge	trnL-trnF	KF897971	FTOL v1.7
Tectariaceae	Tectaria phaeocaulis (Rosenst.) C. Chr.	atpA	OR087226	FTOL v1.7
Tectariaceae	Tectaria phaeocaulis (Rosenst.) C. Chr.	rbcL	KY937354	FTOL v1.7
Tectariaceae	Tectaria phaeocaulis (Rosenst.) C. Chr.	rps4	KY937428	FTOL v1.7
Tectariaceae	Tectaria phaeocaulis (Rosenst.) C. Chr.	rps4-trnS	KY937428	FTOL v1.7
Tectariaceae	Tectaria phaeocaulis (Rosenst.) C. Chr.	trnL-trnF	KY937531	FTOL v1.7
Tectariaceae	Tectaria phanomensis S. Linds.	atpA	OR087230	FTOL v1.7
Tectariaceae	Tectaria phanomensis S. Linds.	rbcL	OR087059	FTOL v1.7
Tectariaceae	Tectaria phanomensis S. Linds.	rps4	OR087411	FTOL v1.7
Tectariaceae	Tectaria phanomensis S. Linds.	rps4-trnS	OR087410	FTOL v1.7
Tectariaceae	Tectaria phanomensis S. Linds.	trnL-trnF	OR087588	FTOL v1.7
Tectariaceae	Tectaria pica (L. fil.) C. Chr.	atpA	KF898027	FTOL v1.7
Tectariaceae	Tectaria pica (L. fil.) C. Chr.	rbcL	KF992520	FTOL v1.7
Tectariaceae	Tectaria pica (L. fil.) C. Chr.	trnL-trnF	KF897973	FTOL v1.7
Tectariaceae	Tectaria pilosa (Fée) R. C. Moran	atpA	OR087234	FTOL v1.7
Tectariaceae	Tectaria pilosa (Fée) R. C. Moran	rbcL	OR087064	FTOL v1.7
Tectariaceae	Tectaria pilosa (Fée) R. C. Moran	rps4	OR087416	FTOL v1.7
Tectariaceae	Tectaria pilosa (Fée) R. C. Moran	rps4-trnS	OR087416	FTOL v1.7
Tectariaceae	Tectaria pilosa (Fée) R. C. Moran	trnL-trnF	OR087593	FTOL v1.7
Tectariaceae	Tectaria pleiosora (Alderw.) C. Chr.	atpB	OK480103	FTOL v1.7
Tectariaceae	Tectaria pleiosora (Alderw.) C. Chr.	rbcL	OK104194	FTOL v1.7
Tectariaceae	Tectaria pleiosora (Alderw.) C. Chr.	trnL-trnF	OK480226	FTOL v1.7
Tectariaceae	Tectaria polymorpha (Wall. ex Hook.) Copel.	atpA	KY937610	FTOL v1.7
Tectariaceae	Tectaria polymorpha (Wall. ex Hook.) Copel.	rbcL	KY937351	FTOL v1.7
Tectariaceae	Tectaria polymorpha (Wall. ex Hook.) Copel.	rps4	KY937424	FTOL v1.7
Tectariaceae	Tectaria polymorpha (Wall. ex Hook.) Copel.	rps4-trnS	KY937424	FTOL v1.7
Tectariaceae	Tectaria polymorpha (Wall. ex Hook.) Copel.	trnL-trnF	KY937528	FTOL v1.7
Tectariaceae	Tectaria profereoides (Christ) S. Y. Dong	atpB	MW795608	FTOL v1.7
Tectariaceae	Tectaria profereoides (Christ) S. Y. Dong	rbcL	MW795601	FTOL v1.7
Tectariaceae	Tectaria profereoides (Christ) S. Y. Dong	trnL-trnF	MW795631	FTOL v1.7
Tectariaceae	Tectaria prolifera	atpA	EF463869	FTOL v1.7
Tectariaceae	Tectaria prolifera	atpB	EF463529	FTOL v1.7
Tectariaceae	Tectaria prolifera	rbcL	EF463273	FTOL v1.7
Tectariaceae	Tectaria pseudosiifolia Fraser-Jenk. & Wangdi	rbcL	MT635911	FTOL v1.7
Tectariaceae	Tectaria psomiocarpa S. Y. Dong	atpB	KJ196502	FTOL v1.7
Tectariaceae	Tectaria psomiocarpa S. Y. Dong	rbcL	KJ196822	FTOL v1.7
Tectariaceae	Tectaria psomiocarpa S. Y. Dong	trnL-trnF	KJ196698	FTOL v1.7
Tectariaceae	Tectaria pubens R. C. Moran	atpA	OR087245	FTOL v1.7
Tectariaceae	Tectaria pubens R. C. Moran	rbcL	OR087072	FTOL v1.7
Tectariaceae	Tectaria pubens R. C. Moran	rps4	OR087427	FTOL v1.7
Tectariaceae	Tectaria pubens R. C. Moran	rps4-trnS	OR087427	FTOL v1.7
Tectariaceae	Tectaria pubens R. C. Moran	trnL-trnF	OR087603	FTOL v1.7
Tectariaceae	Tectaria puberula (Desv.) C. Chr.	rbcL	KF992521	FTOL v1.7
Tectariaceae	Tectaria quinquefida (Baker) Ching	atpA	KY937606	FTOL v1.7
Tectariaceae	Tectaria quinquefida (Baker) Ching	rbcL	KY937340	FTOL v1.7
Tectariaceae	Tectaria quinquefida (Baker) Ching	rps4	KY937413	FTOL v1.7
Tectariaceae	Tectaria quinquefida (Baker) Ching	rps4-trnS	KY937413	FTOL v1.7
Tectariaceae	Tectaria quinquefida (Baker) Ching	trnL-trnF	KY937514	FTOL v1.7
Tectariaceae	Tectaria remotipinna Ching & Chu H. Wang	atpB	OK480105	FTOL v1.7
Tectariaceae	Tectaria remotipinna Ching & Chu H. Wang	rbcL	OK104196	FTOL v1.7
Tectariaceae	Tectaria remotipinna Ching & Chu H. Wang	trnL-trnF	OK480229	FTOL v1.7
Tectariaceae	Tectaria repanda (Willd.) Holttum	atpB	KJ196509	FTOL v1.7
Tectariaceae	Tectaria repanda (Willd.) Holttum	rbcL	KJ196831	FTOL v1.7
Tectariaceae	Tectaria repanda (Willd.) Holttum	trnL-trnF	KJ196707	FTOL v1.7
Tectariaceae	Tectaria rivalis (Mett. ex Kuhn) Maxon	rbcL	KJ628854	FTOL v1.7
Tectariaceae	Tectaria sabahensis C. W. Chen & C. J. Rothf.	rbcL	KY927534	FTOL v1.7
Tectariaceae	Tectaria sabahensis C. W. Chen & C. J. Rothf.	trnL-trnF	KY927537	FTOL v1.7
Tectariaceae	Tectaria sagenioides (Mett.) Christenh.	atpA	OR087255	FTOL v1.7
Tectariaceae	Tectaria sagenioides (Mett.) Christenh.	rbcL	OR087078	FTOL v1.7
Tectariaceae	Tectaria sagenioides (Mett.) Christenh.	trnL-trnF	OR087610	FTOL v1.7
Tectariaceae	Tectaria semipinnata (Roxb.) C. V. Morton	atpA	OR087258	FTOL v1.7
Tectariaceae	Tectaria semipinnata (Roxb.) C. V. Morton	atpB	KJ196498	FTOL v1.7
Tectariaceae	Tectaria semipinnata (Roxb.) C. V. Morton	rbcL	OR087079	FTOL v1.7
Tectariaceae	Tectaria semipinnata (Roxb.) C. V. Morton	rps4	OR087438	FTOL v1.7
Tectariaceae	Tectaria semipinnata (Roxb.) C. V. Morton	rps4-trnS	KU605097	FTOL v1.7
Tectariaceae	Tectaria semipinnata (Roxb.) C. V. Morton	trnL-trnF	OR087613	FTOL v1.7
Tectariaceae	Tectaria setulosa (Baker) Holttum	atpA	KU605220	FTOL v1.7
Tectariaceae	Tectaria setulosa (Baker) Holttum	rbcL	KU605191	FTOL v1.7
Tectariaceae	Tectaria setulosa (Baker) Holttum	rps4	KU605098	FTOL v1.7
Tectariaceae	Tectaria setulosa (Baker) Holttum	rps4-trnS	KU605098	FTOL v1.7
Tectariaceae	Tectaria setulosa (Baker) Holttum	trnL-trnF	KU605123	FTOL v1.7
Tectariaceae	Tectaria simonsii (Baker) Ching	plastome	MT130623	FTOL v1.7
Tectariaceae	Tectaria singaporeana (Wall. ex Hook. & Grev.) Copel.	atpA	OR087271	FTOL v1.7
Tectariaceae	Tectaria singaporeana (Wall. ex Hook. & Grev.) Copel.	atpB	MF623699	FTOL v1.7
Tectariaceae	Tectaria singaporeana (Wall. ex Hook. & Grev.) Copel.	rbcL	KF887196	FTOL v1.7
Tectariaceae	Tectaria singaporeana (Wall. ex Hook. & Grev.) Copel.	rps4	OR087445	FTOL v1.7
Tectariaceae	Tectaria singaporeana (Wall. ex Hook. & Grev.) Copel.	rps4-trnS	KY937444	FTOL v1.7
Tectariaceae	Tectaria singaporeana (Wall. ex Hook. & Grev.) Copel.	trnL-trnF	KY937548	FTOL v1.7
Tectariaceae	Tectaria subconfluens (Bedd.) Ching	atpA	OR087288	FTOL v1.7
Tectariaceae	Tectaria subconfluens (Bedd.) Ching	rbcL	OR087096	FTOL v1.7
Tectariaceae	Tectaria subconfluens (Bedd.) Ching	rps4	OR087469	FTOL v1.7
Tectariaceae	Tectaria subconfluens (Bedd.) Ching	rps4-trnS	OR087469	FTOL v1.7
Tectariaceae	Tectaria subconfluens (Bedd.) Ching	trnL-trnF	OR087641	FTOL v1.7
Tectariaceae	Tectaria subfuscipes (Tagawa) C. M. Kuo	atpA	KY937629	FTOL v1.7
Tectariaceae	Tectaria subfuscipes (Tagawa) C. M. Kuo	rbcL	KY937373	FTOL v1.7
Tectariaceae	Tectaria subfuscipes (Tagawa) C. M. Kuo	rps4	KY937459	FTOL v1.7
Tectariaceae	Tectaria subfuscipes (Tagawa) C. M. Kuo	rps4-trnS	KY937459	FTOL v1.7
Tectariaceae	Tectaria subfuscipes (Tagawa) C. M. Kuo	trnL-trnF	KY937563	FTOL v1.7
Tectariaceae	Tectaria subglabrata (Holttum) S. Y. Dong	atpB	KJ196406	FTOL v1.7
Tectariaceae	Tectaria subglabrata (Holttum) S. Y. Dong	rbcL	KJ196807	FTOL v1.7
Tectariaceae	Tectaria subglabrata (Holttum) S. Y. Dong	trnL-trnF	KJ196676	FTOL v1.7
Tectariaceae	Tectaria subsageniacea (Christ) Christenh.	atpA	OR087157	FTOL v1.7
Tectariaceae	Tectaria subsageniacea (Christ) Christenh.	rbcL	OR087025	FTOL v1.7

Tectariaceae	Tectaria subsageniacea (Christ) Christenh.	rps4	OR087344	FTOL v1.7
Tectariaceae	Tectaria subsageniacea (Christ) Christenh.	rps4-trnS	OR087344	FTOL v1.7
Tectariaceae	Tectaria subsageniacea (Christ) Christenh.	trnL-trnF	OR087530	FTOL v1.7
Tectariaceae	Tectaria subtriphylla (Hook. & Arn.) Copel.	atpA	KY937632	FTOL v1.7
Tectariaceae	Tectaria subtriphylla (Hook. & Arn.) Copel.	rbcL	KY937376	FTOL v1.7
Tectariaceae	Tectaria subtriphylla (Hook. & Arn.) Copel.	rps4	KY937463	FTOL v1.7
Tectariaceae	Tectaria subtriphylla (Hook. & Arn.) Copel.	rps4-trnS	KY937463	FTOL v1.7
Tectariaceae	Tectaria subtriphylla (Hook. & Arn.) Copel.	trnL-trnF	KY937567	FTOL v1.7
Tectariaceae	Tectaria subvariolosa	atpA	KU605224	FTOL v1.7
Tectariaceae	Tectaria subvariolosa	rbcL	KU605204	FTOL v1.7
Tectariaceae	Tectaria subvariolosa	rps4	KU605102	FTOL v1.7
Tectariaceae	Tectaria subvariolosa	rps4-trnS	KU605102	FTOL v1.7
Tectariaceae	Tectaria subvariolosa	trnL-trnF	KU605126	FTOL v1.7
Tectariaceae	Tectaria sulitii Copel.	atpB	MF623700	FTOL v1.7
Tectariaceae	Tectaria sulitii Copel.	rbcL	MF623772	FTOL v1.7
Tectariaceae	Tectaria sulitii Copel.	trnL-trnF	MF623792	FTOL v1.7
Tectariaceae	Tectaria tahitensis Maxon	rbcL	KY099871	FTOL v1.7
Tectariaceae	Tectaria trichotoma (Fée) Tagawa	atpB	OK480110	FTOL v1.7
Tectariaceae	Tectaria trichotoma (Fée) Tagawa	rbcL	OK104199	FTOL v1.7
Tectariaceae	Tectaria trichotoma (Fée) Tagawa	trnL-trnF	OK480233	FTOL v1.7
Tectariaceae	Tectaria tricuspis (Bedd.) Copel.	atpB	KJ196501	FTOL v1.7
Tectariaceae	Tectaria tricuspis (Bedd.) Copel.	rbcL	KJ196820	FTOL v1.7
Tectariaceae	Tectaria tricuspis (Bedd.) Copel.	trnL-trnF	KJ196694	FTOL v1.7
Tectariaceae	Tectaria trifoliata (L.) Cav.	atpA	EF463870	FTOL v1.7
Tectariaceae	Tectaria trifoliata (L.) Cav.	atpB	EF463530	FTOL v1.7
Tectariaceae	Tectaria trifoliata (L.) Cav.	rbcL	EF463274	FTOL v1.7
Tectariaceae	Tectaria trifoliata (L.) Cav.	trnL-trnF	KF897981	FTOL v1.7
Tectariaceae	Tectaria vanikoroensis S.Y.Dong & C.W.Chen	atpB	OK480111	FTOL v1.7
Tectariaceae	Tectaria vanikoroensis S.Y.Dong & C.W.Chen	rbcL	OK104200	FTOL v1.7
Tectariaceae	Tectaria vanikoroensis S.Y.Dong & C.W.Chen	trnL-trnF	OK480234	FTOL v1.7
Tectariaceae	Tectaria vasta (Blume) Copel.	atpA	KY937635	FTOL v1.7
Tectariaceae	Tectaria vasta (Blume) Copel.	rbcL	OR087100	FTOL v1.7
Tectariaceae	Tectaria vasta (Blume) Copel.	rps4	KY937468	FTOL v1.7
Tectariaceae	Tectaria vasta (Blume) Copel.	rps4-trnS	KY937468	FTOL v1.7
Tectariaceae	Tectaria vasta (Blume) Copel.	trnL-trnF	OR087647	FTOL v1.7
Tectariaceae	Tectaria wightii (C. B. Clarke) Ching	atpB	KJ196416	FTOL v1.7
Tectariaceae	Tectaria wightii (C. B. Clarke) Ching	rbcL	KJ196906	FTOL v1.7
Tectariaceae	Tectaria wightii (C. B. Clarke) Ching	trnL-trnF	KJ196710	FTOL v1.7
Tectariaceae	Tectaria zeilanica (Houtt.) Sledge	plastome	MT130702	FTOL v1.7
Tectariaceae	Triplophyllum crassifolium Holttum	atpA	KF898037	FTOL v1.7
Tectariaceae	Triplophyllum crassifolium Holttum	rbcL	OR087101	FTOL v1.7
Tectariaceae	Triplophyllum crassifolium Holttum	rps4	OR087476	FTOL v1.7
Tectariaceae	Triplophyllum crassifolium Holttum	rps4-trnS	OR087476	FTOL v1.7
Tectariaceae	Triplophyllum crassifolium Holttum	trnL-trnF	OR087649	FTOL v1.7
Tectariaceae	Triplophyllum dicksonioides (Fée) Holttum	atpA	OR087301	FTOL v1.7
Tectariaceae	Triplophyllum dicksonioides (Fée) Holttum	rbcL	OR087103	FTOL v1.7
Tectariaceae	Triplophyllum dicksonioides (Fée) Holttum	rps4	OR087479	FTOL v1.7
Tectariaceae	Triplophyllum dicksonioides (Fée) Holttum	rps4-trnS	OR087479	FTOL v1.7
Tectariaceae	Triplophyllum dicksonioides (Fée) Holttum	trnL-trnF	OR087652	FTOL v1.7
Tectariaceae	Triplophyllum fraternum (Mett.) Holttum	rbcL	KF667657	FTOL v1.7
Tectariaceae	Triplophyllum fraternum (Mett.) Holttum	rps4-trnS	KF667572	FTOL v1.7
Tectariaceae	Triplophyllum fraternum (Mett.) Holttum	trnL-trnF	KF667630	FTOL v1.7
Tectariaceae	Triplophyllum funestum (Kunze) Holttum	atpA	OR087307	FTOL v1.7
Tectariaceae	Triplophyllum funestum (Kunze) Holttum	rbcL	OR087110	FTOL v1.7
Tectariaceae	Triplophyllum funestum (Kunze) Holttum	rps4	OR087487	FTOL v1.7
Tectariaceae	Triplophyllum funestum (Kunze) Holttum	rps4-trnS	OR087487	FTOL v1.7
Tectariaceae	Triplophyllum funestum (Kunze) Holttum	trnL-trnF	OR087660	FTOL v1.7
Tectariaceae	Triplophyllum glabrum J. Prado & R. C. Moran	atpA	OR087313	FTOL v1.7
Tectariaceae	Triplophyllum glabrum J. Prado & R. C. Moran	rbcL	OR087120	FTOL v1.7
Tectariaceae	Triplophyllum glabrum J. Prado & R. C. Moran	rps4	OR087498	FTOL v1.7
Tectariaceae	Triplophyllum glabrum J. Prado & R. C. Moran	rps4-trnS	OR087498	FTOL v1.7
Tectariaceae	Triplophyllum glabrum J. Prado & R. C. Moran	trnL-trnF	OR087671	FTOL v1.7
Tectariaceae	Triplophyllum heudelotii Pic. Serm.	atpA	KF898042	FTOL v1.7
Tectariaceae	Triplophyllum heudelotii Pic. Serm.	rbcL	KF887208	FTOL v1.7
Tectariaceae	Triplophyllum heudelotii Pic. Serm.	trnL-trnF	KF897990	FTOL v1.7
Tectariaceae	Triplophyllum hirsutum (Holttum) J. Prado & R. C. Mora	atpA	OR087318	FTOL v1.7
Tectariaceae	Triplophyllum hirsutum (Holttum) J. Prado & R. C. Mora	rbcL	OR087129	FTOL v1.7
Tectariaceae	Triplophyllum hirsutum (Holttum) J. Prado & R. C. Mora	rps4	OR087514	FTOL v1.7
Tectariaceae	Triplophyllum hirsutum (Holttum) J. Prado & R. C. Mora	rps4-trnS	OR087514	FTOL v1.7
Tectariaceae	Triplophyllum hirsutum (Holttum) J. Prado & R. C. Mora	trnL-trnF	OR087679	FTOL v1.7
Tectariaceae	Triplophyllum jenseniae (C. Chr.) Holttum	rbcL	KF667660	FTOL v1.7
Tectariaceae	Triplophyllum jenseniae (C. Chr.) Holttum	rps4-trnS	KF667575	FTOL v1.7
Tectariaceae	Triplophyllum jenseniae (C. Chr.) Holttum	trnL-trnF	KF667633	FTOL v1.7
Tectariaceae	Triplophyllum pentagonum (Bonap.) Holttum	rbcL	KF667662	FTOL v1.7
Tectariaceae	Triplophyllum pentagonum (Bonap.) Holttum	rps4-trnS	KF667577	FTOL v1.7
Tectariaceae	Triplophyllum pentagonum (Bonap.) Holttum	trnL-trnF	KF667635	FTOL v1.7
Tectariaceae	Triplophyllum pilosissimum (J. Sm. ex T. Moore) Holttu	rps4	KU605103	FTOL v1.7
Tectariaceae	Triplophyllum pilosissimum (J. Sm. ex T. Moore) Holttu	rps4-trnS	KU605103	FTOL v1.7
Tectariaceae	Triplophyllum pilosissimum (J. Sm. ex T. Moore) Holttu	trnL-trnF	KU605127	FTOL v1.7
Tectariaceae	Triplophyllum securidiforme (Hook.) Holttum	trnL-trnF	KU605128	FTOL v1.7
Tectariaceae	Triplophyllum vogelii (Hook.) Holttum	rbcL	KF667661	FTOL v1.7
Tectariaceae	Triplophyllum vogelii (Hook.) Holttum	rps4-trnS	KF667576	FTOL v1.7
Tectariaceae	Triplophyllum vogelii (Hook.) Holttum	trnL-trnF	KF667634	FTOL v1.7

Table S3. Primer sets used in this study

DNA region	Primer name	Sequence (5'- 3')	Reference
rbcL	ESRBCL1F	ATGTCACCACAAACGGAGACTAAAGC	Schuettpelz and Pryer (2007)
	ESRBCL1361R	TCAGGACTCCACTTACTAGCTTCACG	
atpA	ESATPF412F	GARCARGTTCGACAGCAAGT	Schuettpelz et al. (2006)
	ESTRN46F	GTATAGGTTCRARTCCTATTGGACG	
	ESATPA877R*	CATCTCCCGGATATGCTTCTCG	
	ESATPA535F*	ACAGCAGTAGCTACAGATAC	
atpB	ESATPE45R	ATTCCAAACWATTCGATTWGGAG	Schuettpelz and Pryer (2007)
	ESATPB172F	AATGTTACTTGTGAAGTWCAACAAT	
rps4-trnS	F	ATGTCCCGTTATCGAGGACCT	Nadot et al. (1995)
	R	TACCGAGGGTTCGAATC	Smith and Cranfill (2002)
trnL-F	Fern-1	GGCAGCCCCCARATTCAGGGRAACC	Trewick et al. (2002)
	f	ATTTGAACTGGTGACACGAG	Taberlet et al. (1991)

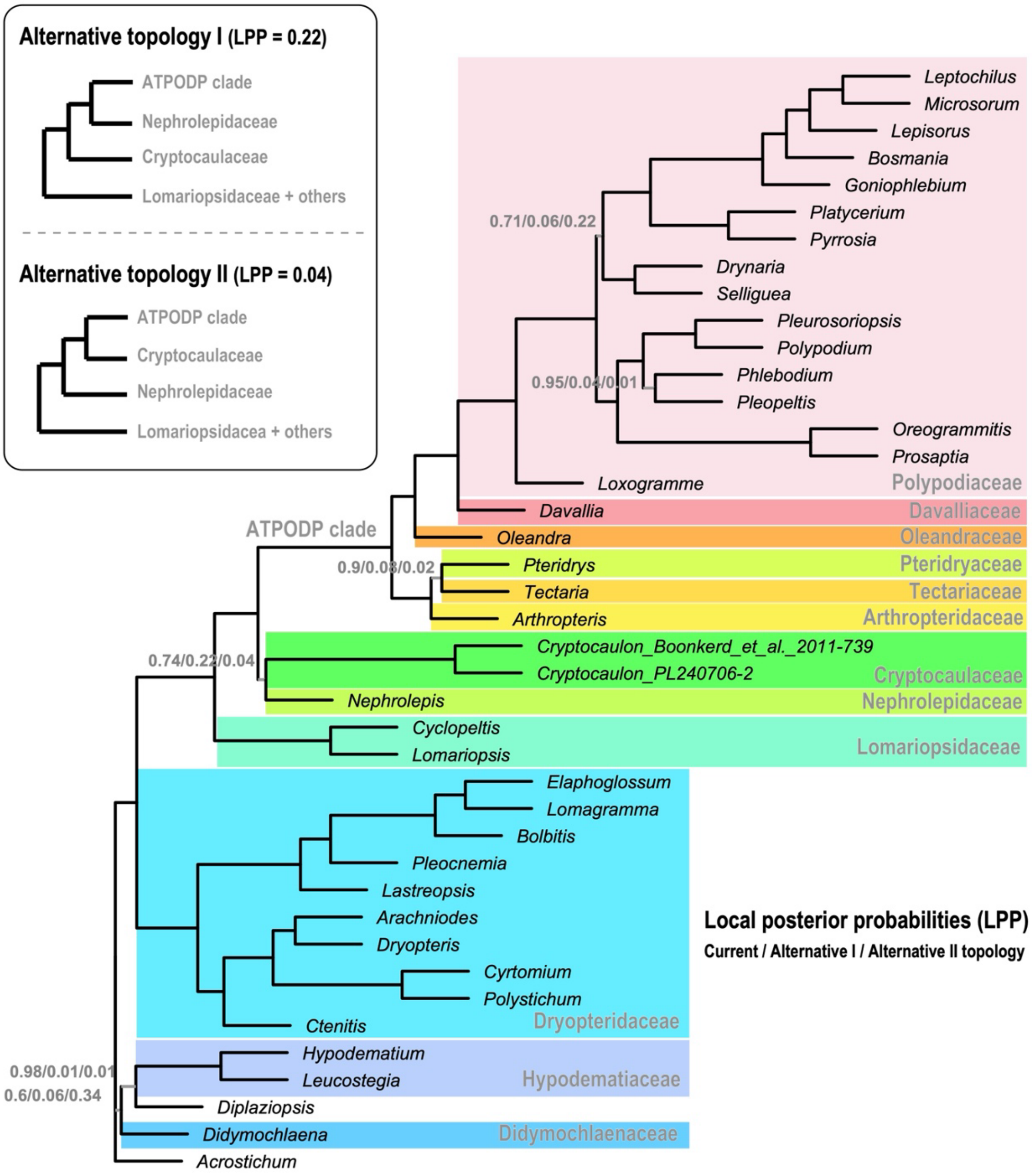

Supplementary Fig. 1. Species tree inferred from individual gene trees of 86 plastid CDSs. Only ASTRAL local posterior probabilities (LPP) of the current topology lower than 1 are shown. The upper-left panel illustrates alternative topologies, indicating the placements of Cryptocaulaceae along with their corresponding LPP values.

Supplementary Fig. S2. The full species-level plastid tree based on 6 plastid regions, atpA, atpB, rbcL, rps4-trnS, and trnL-F.

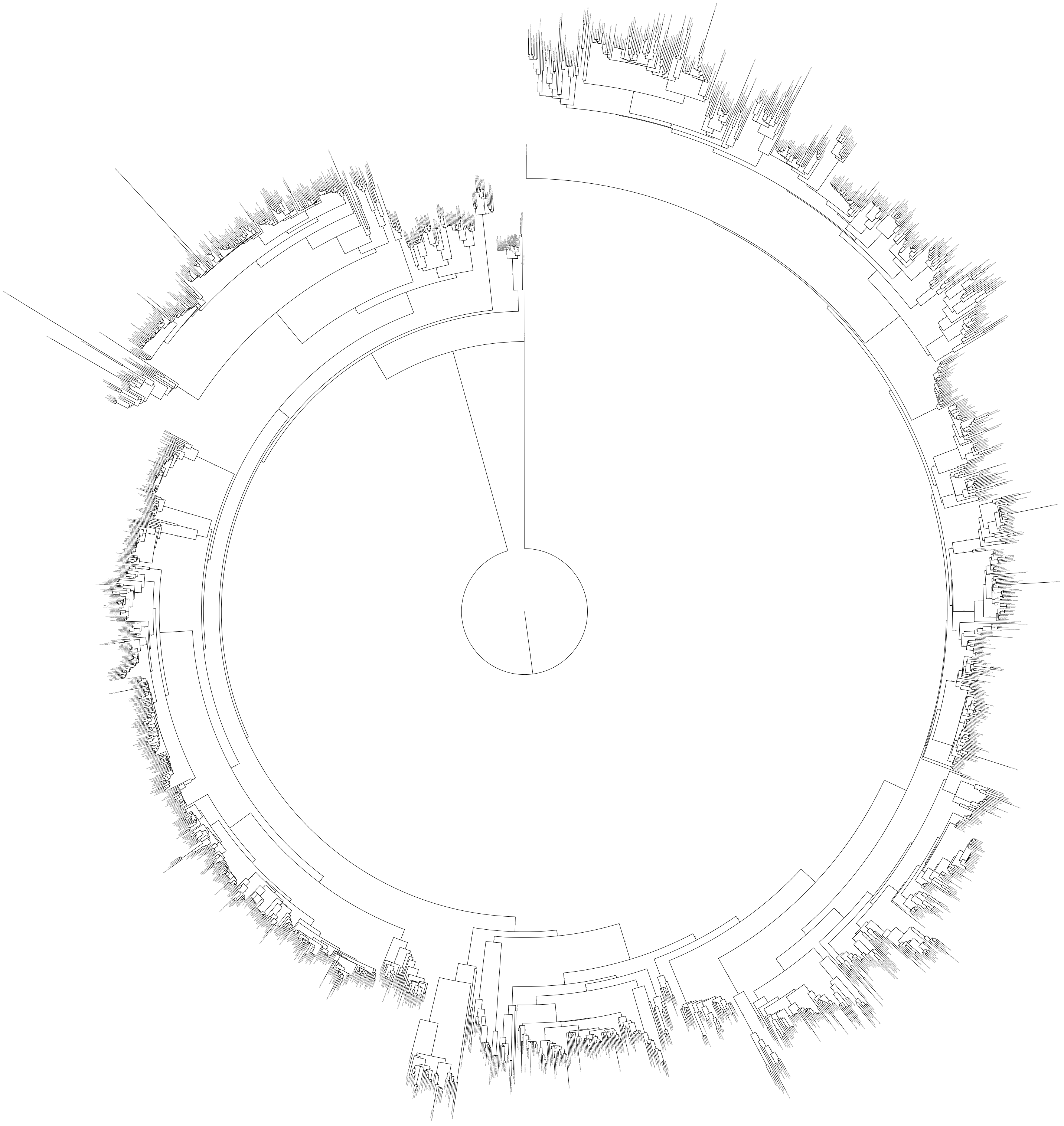

Supplement: Supplementary file 1 — Supplementary Material 1. [file 10265_2026_1698_MOESM1_ESM.pdf]
